# Improved Variable Selection Algorithm Using a LASSO-Type Penalty, with an Application to Assessing Hepatitis B Infection Relevant Factors in Community Residents

**DOI:** 10.1371/journal.pone.0134151

**Published:** 2015-07-27

**Authors:** Pi Guo, Fangfang Zeng, Xiaomin Hu, Dingmei Zhang, Shuming Zhu, Yu Deng, Yuantao Hao

**Affiliations:** 1 Department of Medical Statistics and Epidemiology and Health Information Research Center, School of Public Health, Sun Yat-sen University, Guangzhou, Guangdong, 510080, China; 2 Laboratory of Health Informatics, Guangdong Key Laboratory of Medicine, Sun Yat-sen University, Guangzhou, Guangdong, 510080, China; Queen's University Belfast, UNITED KINGDOM

## Abstract

**Objectives:**

In epidemiological studies, it is important to identify independent associations between collective exposures and a health outcome. The current stepwise selection technique ignores stochastic errors and suffers from a lack of stability. The alternative LASSO-penalized regression model can be applied to detect significant predictors from a pool of candidate variables. However, this technique is prone to false positives and tends to create excessive biases. It remains challenging to develop robust variable selection methods and enhance predictability.

**Material and methods:**

Two improved algorithms denoted the two-stage hybrid and bootstrap ranking procedures, both using a LASSO-type penalty, were developed for epidemiological association analysis. The performance of the proposed procedures and other methods including conventional LASSO, Bolasso, stepwise and stability selection models were evaluated using intensive simulation. In addition, methods were compared by using an empirical analysis based on large-scale survey data of hepatitis B infection-relevant factors among Guangdong residents.

**Results:**

The proposed procedures produced comparable or less biased selection results when compared to conventional variable selection models. In total, the two newly proposed procedures were stable with respect to various scenarios of simulation, demonstrating a higher power and a lower false positive rate during variable selection than the compared methods. In empirical analysis, the proposed procedures yielding a sparse set of hepatitis B infection-relevant factors gave the best predictive performance and showed that the procedures were able to select a more stringent set of factors. The individual history of hepatitis B vaccination, family and individual history of hepatitis B infection were associated with hepatitis B infection in the studied residents according to the proposed procedures.

**Conclusions:**

The newly proposed procedures improve the identification of significant variables and enable us to derive a new insight into epidemiological association analysis.

## Introduction

The variable selection technique is employed for epidemiologic analysis to identify independent associations between collective exposures and a health outcome [1]. Selection of the best variables is aimed at controlling confounders to obtain unbiased estimates of covariate effects and predicting probabilities with robust estimation [2–5]. In epidemiological studies, automatic variable selection using stepwise regression is the most widely used method. However, it is not always optimal when applied for identifying independent associations in large epidemiologic data sets with many predictors [6, 7].

Currently, due to the steady growth of epidemiological data resulting from large-scale cohort studies and routinely collected electronic surveillance information of disease, the collection of many predictors offers new challenges for developing advanced variable selection methods [8]. In practice, the stepwise selection method requires arbitrary definitions of thresholds that are used to decide which variables to include or exclude from the model, an inherent problem that has been discussed in previous studies [2–4, 9, 10]. For instance, the stepwise selection technique ignores stochastic errors inherited in the stages of variable selection and suffers from a lack of stability [11]. In this case, a model using univariate or non-penalized regression modeling approaches is likely to overfit the data and generates findings that will not generalize well when extended to new data. Instead, penalized regression models from the field of machine learning are more flexible than conventional statistical regression methods and have been proposed to deal with data sets involving many covariates [7]. The Least Absolute Shrinkage and Selection Operator (LASSO) [12] model is one such model, and has been developed to overcome the limitations when there are many predictors analyzed. By shrinking variables with very unstable estimates towards zero, the LASSO model can effectively exclude some irrelevant variables and produce sparse estimations.

For epidemiologic analysis, when the collected data sets involve many confounders, control of all measured confounders can lead to problems for conventional model-fitting methods. The LASSO shrinkage regression model has been increasingly used to adjust various confounders and investigate the associations between several exposures and a health outcome [13–16]. However, in practice, the LASSO model creates excessive biases when selecting significant variables and is not consistent in terms of variable selection [17, 18]. This means the group of variables, selected by LASSO, is not consistently comprised of the true set of important variables. It remains challenging to develop robust techniques of variable selection and enhance predictability for epidemiologic analysis. We therefore need a modeling strategy that can incorporate a shrinkage approach to avoid the overfitting of a regression model with various confounders, identify informative predictors from a pool of candidate variables, and estimate the model parameters with low variability for epidemiologic analysis.

This work addresses the gap by a) rigorously evaluating the performance of the stepwise selection technique, stability selection method [19] and LASSO-type shrinkage regression models, and b) introducing two improved algorithms denoted a two-stage hybrid and a bootstrap ranking procedure, that use a LASSO-type penalty for identifying informative variables. We initially analyze their respective characteristics of the different methods based on performance, and then put forward feasible improved schemes for robust variable selection. The specific algorithms of the two proposed procedures are compared with previously published methods, using intensive simulations. We further apply the newly proposed methods to a large-scale epidemiology survey data of hepatitis B virus (HBV) infection to assess relevant factors among community residents. The empirical research aims to investigate the detection efficiency of key variables by the different methods. Results indicate that the proposed procedures are capable of identifying informative variables with better predictive performance.

## Material and Methods

### Variable selection using the LASSO shrinkage regression model

Let ***y***_***i***_ = 0 or 1 denotes the binary outcome of the *i*th sample of *n* individuals. For example, ***y***_***i***_ denotes the presence or absence of HBV infection of individual *i* in this study. Let us define ***y*** = (***y***_1_,…,***y***_***n***_)^***T***^ as the binary outcomes for all the *n* individuals. The probability of observing ***y***_***i***_ = 1 is written as ***p***_***i***_ = Pr(***y***_***i***_ = 1), ***i*** = 1,…,***n***. Let ***x***_***i***_ = (***x***_***i***1_,…,***x***_***ip***_)^***T***^ denote a *p*-dimensional vector of predictors that may be associated with the outcome ***y***_***i***_. We model the relationship of ***y***_***i***_ with ***x***_***i***_ through a logistic regression model:

$$\mathrm{logit}(p_{i})=\beta_{0}+\sum_{j=1}^{p}\beta_{j}x_{ij},$$

where *β*_0_ and *β*_***j***_ are the intercept and regression coefficients, and **logit**(***p***_***i***_) is estimated by:

$$\mathrm{logit}(p_{i})=\log\left[\frac{\mathrm{Pr}(y_{i}=1)}{1-\mathrm{Pr}(y_{i}=1)}\right].$$

We define the parameter vector *β* = (*β*_0_,*β*_1_…,*β*_***p***_)^***T***^, and then the above logistic regression model can be written in a more compact form as: $\mathrm{logit}(p_{i})=x_{i}^{T}\beta$.

The LASSO is a regularization technique for simultaneous estimation and variable selection [12]. The LASSO estimates $\hat{\beta }$ are given by

$$\hat{\beta }=\underset{\beta }{\arg\;\min}\sum_{i=1}^{n}\left(-y_{i}(x_{i}^{T}\beta )+\log(1+e^{x_{i}^{T}\beta })\right)+\lambda \sum_{j=1}^{p}\left|\beta_{j}\right|,$$

where *λ* is a nonnegative tuning parameter. In LASSO, the penalty function continuously shrinks the coefficients toward zero; the larger the value of *λ*, the greater the amount of shrinkage. As *λ* increases, the LASSO model will shrink some of the coefficients to be exactly zero and obtain a sparse subset of variables with non-zero regression coefficients [20].

### Two improved variable selection algorithms using a LASSO-type penalty

#### Two-stage hybrid procedure

The conventional LASSO model tends to select many noisy features with high probability [21]. In this case, application of the adaptive LASSO has proposed to obtain consistent variable selection [22]. In the adaptive LASSO, weights are used for penalizing different coefficients to enjoy the oracle properties, which are related to identifying the right subset model and having the optimal estimation rate [22]. The adaptive LASSO model approximates the true underlying model with a higher probability compared to the conventional LASSO. The adaptive LASSO estimates are defined as:

$$\hat{\beta }=\underset{\beta }{\arg\;\min}\sum_{i=1}^{n}\left(-y_{i}(x_{i}^{T}\beta )+\log(1+e^{x_{i}^{T}\beta })\right)+\lambda \sum_{j=1}^{p}{\hat{w}}_{j}|\beta_{j}|,$$

where ***w*** is a data-dependent weight vector. The basic requirement for the weights is that the ***w***_***j***_ should be relatively large if the true value of *β*_***j***_ = 0, and ***w***_***j***_ should be relatively small if *β*_***j***_ ≠ 0 [22]. According to previous findings, the ordinary least squares (OLS) estimator $\hat{\beta }(\mathrm{ols})$ can be used for computing the weight vector $\hat{w}=1/|\hat{\beta }(\mathrm{ols}){|}^{\gamma }$ (*γ* > 0) in the adaptive LASSO model [22]. However, the adaptive LASSO suffers from the multicollinearity caused by large correlations among covariates due to the OLS estimates, used for computing the weights, being very unstable in this situation [23]. In reality, a large number of covariates usually are introduced at the initial stage of statistical modeling in practical data analysis, but only a small number of covariates are the true factors correlated with the outcome. Hence, we established a two-stage hybrid procedure for variable selection in this work. The LASSO was performed to obtain an initial estimator of the coefficients and to reduce the dimension of the model. The coefficient estimates of variables screened by the first stage were used for the weighting parameters of the adaptive LASSO in the second stage to select consistent variables. The pseudocode of the two-stage hybrid procedure is given in Algorithm 1.

**Algorithm 1.** Two-stage hybrid procedure.

Input:

- (*X*, *Y*): training set that contains *n* samples and a *p*-dimensional vector of predictors, and $(X,\;Y)\in {\mathbb{R}}^{n\times (p\times 1)}$.
- *λ*_1_: tuning parameter in the LASSO penalty.
- *γ*: parameter for computing the weight vector ***w*** in the adaptive LASSO penalty.
- *λ*_2_: tuning parameter in the adaptive LASSO penalty.

**Output:** optimal variable subset *S*.

/Stage 1:/

**1:** Compute the LASSO estimates

$$\hat{\beta }(\lambda_{1})=\underset{\beta }{\arg\;\min}\sum_{i=1}^{n}\left(-y_{i}(x_{i}^{T}\beta )+\log(1+e^{x_{i}^{T}\beta })\right)+\lambda_{1}\sum_{j=1}^{p}\left|\beta_{j}\right|$$

**2:** Compute the non-zero coefficients set $J_{1}=\{j,\;{\hat{\beta }}_{j}\neq 0\}$ in the LASSO regression
**3:** Compute the zero coefficients set $J_{{}_{1}}^{*}=\{j,\;{\hat{\beta }}_{j}=0\}$ in the LASSO regression

/Stage 2: /

**4:** Compute the weight vector $w=1/|\hat{\beta }(J_{1}){|}^{\gamma }$ of the adaptive LASSO penalty
**5:** Set $\hat{\beta }(J_{{}_{1}}^{*})=0$ in the adaptive LASSO regression
**6:** Compute the adaptive LASSO estimates

$$\hat{\beta }(\lambda_{2})=\underset{\beta }{\arg\;\min}\sum_{i=1}^{n}\left(-y_{i}(x_{i}^{T}\beta )+\log(1+e^{x_{i}^{T}\beta })\right)+\lambda_{2}\sum_{j=1}^{p}{\hat{w}}_{j}|\beta_{j}|$$

**7:** Compute the non-zero coefficients $J_{2}=\{j,\;{\hat{\beta }}_{j}\neq 0\}$ in the adaptive LASSO regression
**8:** Generate the optimal variable subset *S* = *X*(*J*_2_)

For the adaptive LASSO, the parameter *γ* was usually set equal to 1 to determine the initial weight vector [21, 22]. The coordinate descent algorithm was used for LASSO estimation and the optimal tuning parameter was selected via the *K*-fold cross-validation [24]. As presented in the first stage of Algorithm 1, a portion of the irrelevant covariates were eliminated by the LASSO model and a relatively sparse set of variables was obtained. In the second stage, we computed the coefficients of the adaptive LASSO using the local quadratic approximation algorithm, which was proposed to approximate the nonconvex penalty function in generalized linear models based on penalized likelihood inference [25]. The optimal *λ*_2_ of the adaptive LASSO penalty was selected in a similar fashion used in the LASSO-penalized model.

#### Bootstrap ranking procedure

We also proposed an alternative robust selection procedure based on bootstrap ranking for a comparison. The bootstrap ranking procedure generates a LASSO estimates matrix representing variable ranking according to importance, and runs the external intersection operation to extract a panel of informative variables. Bach [26] proposed to use several bootstrap samples of a given dataset for running LASSO and intersected several sets of the estimates of the non-zero coefficients from LASSO to get a consistent model selection. In the bootstrap ranking procedure, we performed the intersection operation in a similar way to extract relevant variables from sufficiently many different data sets using the sample bootstrapping method. However, instead of directly intersecting several sets of non-zero LASSO estimates, we generated a LASSO estimate matrix representing variable ranking according to their importance, and then intersected to obtain a robust result. During each internal loop, bootstrap samples were generated by randomly selecting *n*_*bootstrap*_ individuals with replacement from a given data set of *n* individuals. For each such sample, LASSO regression coefficients were estimated for all variables, and the average estimation across internal bootstraps was calculated as the measurement of variable importance. The importance score is given by

$$Score_{k}=|B_{2}{}^{-1}\sum_{j=1}^{B_{2}}J_{jk}|,$$

where *B*_2_ denotes the number of internal sampling and *J*_*jk*_ represents the estimate of the regression coefficient in LASSO. The scores of variables were sorted in a descending order of importance, and a segmented regression model was employed to detect the breakpoint on the score curve. The variables with the highest scores were identified as a panel of informative variables. The pseudocode of the bootstrap ranking procedure is given in Algorithm 2.

**Algorithm 2.** Bootstrap ranking procedure.

Input:

- (*X*, *Y*): training set that contains *n* samples and a *p*-dimensional vector of predictors, and $(X,\;Y)\in {\mathbb{R}}^{n\times (p\times 1)}$.
- *B*_1_: number of external samplings for the intersection operation.
- *B*_2_: number of internal samplings for the variable ranking operation.
- *n*_*bootstrap*_: size of bootstrap samples with replacement.
- *λ*: tuning parameter in the LASSO penalty.

**Output:** optimal variable subset *S*.

**1: for** *m* = 1 to *B*_1_ **do**
**2: for** *b* = 1 to *B*_2_ **do**
**3:** Generate bootstrap sample $(X^{b},Y^{b})\in {\mathbb{R}}^{n_{Bootstrap}\times (p+1)}$
**4:** Compute LASSO estimates

$$\hat{\beta }(\lambda )=\underset{\beta }{\arg\;\min}\sum_{i=1}^{n_{\mathrm{bootstrap}}}\left(-y_{i}(x_{i}^{T}\beta )+\log(1+e^{x_{i}^{T}\beta })\right)+\lambda \sum_{j=1}^{p}\left|\beta_{j}\right|$$

**5:** Compute coefficients set at $J_{b}=\{j,\;{\hat{\beta }}_{j}^{b}\}$ in the LASSO regression
**6:** end for
**7:** Generate the LASSO estimates matrix $I_{B_{2}\times p}=\left(\begin{array}{l} J_{1\times p}\\ J_{2\times p}\\ \cdot \cdot \cdot \\ J_{B_{2}\times p} \end{array}\right)$
**8:** Compute the variable importance scores by:

$$Score_{i}=|B_{2}{}^{-1}\sum_{j=1}^{B_{2}}J_{ji}|\;(i=1,2,\ldots ,p)$$

**9:** Sort variables in a descending order with rank ***r*** based on the important scores
**10:** Estimate the breakpoint *τ* using a segmented regression model with one breakpoint on the score curve ***E***[***Score***_***i***_ | ***r***_***i***_] = *β*_1_ + *δ*_1_ ***I***(***r***_***i***_ > *τ*) + *ε*_***i***_
**11:** Obtain the subset ***S***_***m***_ of relevant variables corresponding to ***r***_***i***_ > *τ*
**12:** end for
**13:** Perform the intersection operation on the *m* subsets of variables to construct the optimal set $S=\cap_{m=1}^{B_{1}}S_{m}$

To detect the breakpoint on the score curve, a segmented regression model [27] was performed. In the segmented regression model ***E***[***Score***_***i***_ | ***r***_***i***_] = *β*_1_ + *δ*_1_ ***I***(***r***_***i***_ > *τ*) + *ε*_***i***_, ***r***_***i***_ represents the rank for ***i*** = 1,2,…,***p*** variables, ***I***(⋅) is the indicator function being equal to one when its argument is true, *τ* denotes the breakpoint, *β*_1_ is the mean level for ***r***_***i***_ > *τ*, *δ*_1_ is the difference in the mean level at the breakpoint, and *ε* is the noise. In practice, we needed to choose the number of external samplings for the intersection operation *B*_1_, the number of internal samplings for variable ranking operation *B*_2_, the tuning parameter *λ* for LASSO and the size of the bootstrap samples *n*_*bootstrap*_. Based on our experience, the model performed similarly when *B*_2_ was large. One can take *B*_1_ = 5 and *B*_2_ = 100, for example. In the following simulation study and empirical analysis, we used the above-mentioned parameter settings. In the bootstrap ranking algorithm, the value of the parameter *n*_*bootstrap*_ was set to equal the size of the original data, and the setting was used in the following simulation study and empirical analysis. The tuning parameter was selected using a 10-fold cross-validation approach. Once the optimal variable set *S* was selected in the bootstrap ranking procedure, we estimated the regression coefficients by the unregularized least-square fit method, as proposed for constructing the Bolasso model [26]. In the simulation study and empirical analysis, the unregularized least-square fit method was applied to estimate the regression coefficients for both the Bolasso and bootstrap ranking methods. The R codes of the two proposed algorithms have been provided and made freely available for academic use (Table A and B in S1 File).

### Simulation study

We conducted a simulation study to demonstrate the validity of the two proposed methods, and compared them to other methods. We referred to the Monte Carlo simulation scheme established by Sabbe et al. [28], and extended it in this work. We considered a total number of *t* = 100 and *t* = 200 predictors. In the case of *t* = 100, 50 categorical and 50 continuous predictors were generated, respectively. First, we created 4 binary categorical predictor variables. In particular, *cat1* was drawn from a Bernoulli distribution with a success probability of 0.7, *cat2* equaled *cat1* with a probability of 0.8, *cat3* equaled *cat2* with a probability of 0.7, and *cat4* equaled *cat3* with a probability of 0.6. The predictor *cat5* equaled one with a probability of 0.95 if *cat1* and *cat2* were equal, and it equaled zero with a probability of 0.95 otherwise. The predictor *cat6* equaled one with a probability of 0.75 if *cat1* and *cat2* were equal, and it equaled zero with a probability of 0.75 otherwise. The variable *cat7* was drawn from a Bernoulli distribution with a success probability of 0.5, *cat8* equaled *cat7* with a probability of 0.90, *cat9* equaled *cat7* with a probability of 0.80, and *cat10* equaled *cat7* with a probability of 0.70. Next, we independently simulated 40 categorical predictor variables (*cat11* up to *cat50*) that were drawn from a Bernoulli distribution with a success probability of 0.5. We simulated observations for 5 continuous variables (*cnt1* up to *cnt5*) from a normal distribution with mean vector *μ*_0_ = (0, 0, 0, −3, −3). We set all variances to 1 and the correlation coefficient to 0.8. Finally, we independently drew 45 more continuous variables (*cnt6* up to *cnt50*) from standard normal distributions.

In the case of *t* = 200, the categorical variables of *cat1* up to *cat10* were generated as in the above case, and the left 90 categorical variables (*cat11 up to cat100*) were independently drawn from a Bernoulli distribution with probability 0.5. The continuous variables of *cnt1* up to *cnt5* were also generated as in the case of *t* = 100, and the remaining 95 continuous variables (*cnt6 up to cnt100*) were independently drawn from a standard normal distribution.

The main effects of non-zero predictors were simulated in the following scenarios:

1) Scenario 1: For the first scenario we defined a linear model with *r* = 8 non-zero coefficients defined by 4 categorical (*cat1*, *cat2*, *cat11*, *cat12*) and 4 continuous (*cnt1*, *cnt2*, *cnt11*, *cnt12*) predictors. The coefficients of non-zero predictors were set to

$$\beta_{\left\{cat\right\}}^{\left\{S1\right\}}=(3,\;2.5,\;2,\;1.5),$$

$$\beta_{\left\{cnt\right\}}^{\left\{S1\right\}}=(3,\;2.5,\;2,\;1.5).$$

2) Scenario 2: For the second scenario we defined a linear model with *r* = 12 non-zero coefficients defined by 6 categorical (*cat1*, *cat2*, *cat3*, *cat11*, *cat12*, *cat13*) and 6 continuous (*cnt1*, *cnt2*, *cnt3*, *cnt11*, *cnt12*, *cnt13*) predictors. The coefficients of non-zero predictors were set to

$$\beta_{\left\{cat\right\}}^{\left\{S2\right\}}=(3,\;2.2,\;1.2,\;3,\;2.2,\;1.2),$$

$$\beta_{\left\{cnt\right\}}^{\left\{S2\right\}}=(3,\;2.5,\;2,\;3,\;2.5,\;2).$$

3: Scenario 3: For the third scenario we defined a linear model with *r* = 16 non-zero coefficients defined by 8 categorical (*cat1*, *cat2*, *cat3*, *cat4*, *cat11*, *cat12*, *cat13*, *cat14*) and 8 continuous (*cnt1*, *cnt2*, *cnt3*, *cnt4*, *cnt11*, *cnt12*, *cnt13*, *cnt14*) predictors. The coefficients of non-zero predictors were set to

$$\beta_{\left\{cat\right\}}^{\left\{S3\right\}}=(3,\;2.5,\;2,\;1.5,\;3,\;2.5,\;2,\;1.5),$$

$$\beta_{\left\{cnt\right\}}^{\left\{S3\right\}}=(2.8,\;2.2,\;1.8,\;1.5,\;2.8,\;2.2,\;1.8,\;1.5).$$

4: Scenario 4: For the fourth scenario we defined a linear model with *r* = 20 non-zero coefficients defined by 10 categorical (*cat1*, *cat2*, *cat3*, *cat4*, *cat5*, *cat11*, *cat12*, *cat13*, *cat14*, *cat15*) and 10 continuous (*cnt1*, *cnt2*, *cnt3*, *cnt4*, *cnt5*, *cnt11*, *cnt12*, *cnt13*, *cnt14*, *cnt15*) predictors. The coefficients of non-zero predictors were set to

$$\beta_{\left\{cat\right\}}^{\left\{S4\right\}}=(3,\;2.5,\;2,\;1.5,\;1.3,\;3,\;2.5,\;2,\;1.5,\;1.3),$$

$$\beta_{\left\{cnt\right\}}^{\left\{S4\right\}}=(3,\;2.8,\;2.2,\;1.8,\;1.5,\;-3,\;-2.8,\;-2.2,\;-1.8,\;-1.5).$$

From the predictor sets, binary outcomes were generated from a linear logistic regression that contained an intercept (*β*_0_ = 2). The logistic regression equation was $P(Y=1|X=x)=\frac{e^{x\beta }}{1+e^{x\beta }}$. We calculated the probability and defined the target event as 1 with the probability larger than 0.5. We considered sample sizes: *n* = 50, 100, 200, 300 and 500, and calculated three evaluation metrics, including the true positive rate (TPR), false positive rate (FPR), and area under the ROC curve (AUC) score to evaluate the predictive performance of each approach. True positives (TP) were those variables that were correctly identified as significant variables with non-zero coefficients by the approach. False positives (FP) were those variables that were incorrectly identified as significant variables with non-zero coefficients by the approach. TPR and FPR are defined as

$$TPR=\frac{TP}{r}\;\text{and}\;FPR=\frac{FP}{t-r},$$

where *r* is the number of non-zero predictors and *t* is the total number of predictors in the simulated data. A larger AUC score reveals a good balance between TPR and FPR, and indicates a higher power in selecting significant variables. The simulation was repeated 100 times and the average of the statistical metric was calculated. We also recorded how frequently each variable is selected during the 100 simulations. We compared the two proposed procedures with four previously published methods including the stepwise selection, stability selection [19], LASSO and Bolasso [26] models using the TPR, FPR, AUC and variable selection frequency. The metrics evaluated model performance independent of any particular algorithm for variable selection. The stepwise selection technique was established using the stepAIC function in the MASS package within R 3.0.2 software [29]. This function used the "both" mode for search direction and computed the generalized Akaike Information Criterion for model selection. The default parameter settings in this package were used. The R package stabs was applied to establish the stability selection model. Two tuning parameters including a threshold value and a regularization parameter in this model influenced the identification of a set of stable variables. In [19] a threshold value for the tuning parameters between 0.6 and 0.9 was suggested. Our analysis results tended to be similar when using the threshold value at the suggested range. Therefore, a threshold value 0.75 was used and the optimal regularization parameter was determined using a cross-validation approach in this study.

### Sensitivity analysis

To observe the performance of different methods to detect small effect predictors, a sensitivity analysis was performed using two groups of predictors with smaller values of non-zero coefficients compared to the above simulation study. For each group 4 linear models were defined with 8, 12, 16 and 20 non-zero coefficients. In the first group, the coefficients of non-zero predictors were set as follows:

1. *r* = 8: $\beta_{\left\{cat\right\}}^{\left\{S1\right\}}=(2,\;1.5,\;1,\;0.5)$, $\beta_{\left\{cnt\right\}}^{\left\{S1\right\}}=(2,\;1.5,\;1,\;0.5)$;
2. *r* = 12: $\beta_{\left\{cat\right\}}^{\left\{S2\right\}}=(2,\;1.2,\;0.2,\;2,\;1.2,\;0.2)$, $\beta_{\left\{cnt\right\}}^{\left\{S2\right\}}=(2,\;1.5,\;1,\;2,\;1.5,\;1)$;
3. *r* = 16: $\beta_{\left\{cat\right\}}^{\left\{S3\right\}}=(2,\;1.5,\;1,\;0.5,\;2,\;1.5,\;1,\;0.5)$, $\beta_{\left\{cnt\right\}}^{\left\{S3\right\}}=(1.8,\;1.2,\;0.8,\;0.5,\;1.8,\;1.2,\;0.8,\;0.5)$;
4. *r* = 20: $\beta_{\left\{cat\right\}}^{\left\{S4\right\}}=(2,\;1.5,\;1,\;0.5,\;0.3,\;2,\;1.5,\;1,\;0.5,\;0.3)$,

$$\beta_{\left\{cnt\right\}}^{\left\{S4\right\}}=(2,\;1.8,\;1.2,\;0.8,\;0.5,\;-2,\;-1.8,\;-1.2,\;-0.8,\;-0.5).$$

In the second group, the non-zero coefficients were decreased further compared to the first group. The 4 linear models were defined as follows:

1. *r* = 8: $\beta_{\left\{cat\right\}}^{\left\{S1\right\}}=(1,\;0.5,\;1,\;0.5)$, $\beta_{\left\{cnt\right\}}^{\left\{S1\right\}}=(1,\;0.5,\;1,\;0.5)$;
2. *r* = 12: $\beta_{\left\{cat\right\}}^{\left\{S2\right\}}=(1,\;0.2,\;0.2,\;1,\;0.2,\;0.2)$, $\beta_{\left\{cnt\right\}}^{\left\{S2\right\}}=(1,\;0.5,\;1,\;1,\;0.5,\;1)$;
3. *r* = 16: $\beta_{\left\{cat\right\}}^{\left\{S3\right\}}=(1,\;0.5,\;1,\;0.5,\;1,\;0.5,\;1,\;0.5)$, $\beta_{\left\{cnt\right\}}^{\left\{S3\right\}}=(0.8,\;0.2,\;0.8,\;0.5,\;0.8,\;0.2,\;0.8,\;0.5)$;
4. *r* = 20: $\beta_{\left\{cat\right\}}^{\left\{S4\right\}}=(1,\;0.5,\;1,\;0.5,\;0.3,\;1,\;0.5,\;1,\;0.5,\;0.3)$,

$$\beta_{\left\{cnt\right\}}^{\left\{S4\right\}}=(1,\;0.8,\;0.2,\;0.8,\;0.5,\;-1,\;-0.8,\;-0.2,\;-0.8,\;-0.5).$$

In the sensitivity analysis, a total number of predictors *t* = 100 was simulated and the other parameter settings remain the same as the above Monte Carlo simulation. The AUC values for variable selection were applied to evaluate the overall performance of the compared methods.

### Application to identification of relevant epidemiological factors in HBV infection

#### Ethics Statement

This study was approved by the Human Ethics Committee of Sun Yat-Sen University.

#### Data

In the empirical analysis, data were available from a population-based study enrolling 5,357 residents of the Yuexiu district in Guangdong Province, China. This study investigated the relevant epidemiological factors of HBV infection in order to prevent primary HBV infection by promoting community based intervention methods involving health education. Eighteen sub-districts were randomly chosen from the Yuexiu district, and recruitment occurred between January 2013 and July 2014. Residents of the district were more widespread and the samples were representative of residents living in the study areas. The subjects were informed of the details of this study, and gave written informed consent, after receiving detailed explanations of the study and potential consequences, prior to enrollment. After receiving informed consent, we collected venous blood samples aseptically and separated the serum from blood by centrifugation, and transported the serum, in small vials packed in an ice-packed box, to the laboratory for storage at 2–8°C until analysis for hepatitis B surface antigen (HBsAg), using Pathozyme hepatitis B antigen ELISA test kits. The presence of HBsAg was used as an indicator of exposure to HBV. The physical growth indices of residents were measured and a lifestyle questionnaire was filled out to investigate the relevant factors for HBV infection. The questionnaire mainly included the residents’ demographic characteristics (age, sex, height, weight, career, marital status, nationality, education level), life habits (frequency of alcohol consumption, smoking habits, exercise frequency, staying abroad for more than three months per year), medical history (family history of HBV infection, personal history of HBV infection, personal surgical history, personal history of transfusion, history of hepatitis B vaccination). This analysis resulted in completion of 5,357 questionnaires after checking the validity of each questionnaire. Additional discussion of the data appears in the Results section. Data used in this work is available in Table C in S1 File.

#### Statistical analysis

Initially, data that was normally distributed was expressed as mean and standard deviation, and data with a skewed distribution was expressed as the median and interquartile range. Independent sample *t*-tests were used for testing the differences of numeric variables between residents infected with HBV and HBV-free residents, and chi-square tests were used for analysis of categorical data. SPSS software 13.0 (SPSS, Inc., Chicago, IL, USA) was used for the statistical analyses. A *P*-value < 0.05 was considered to be statistically significant.

We compared six different variable selection methods: (1) stepwise selection; (2) stability selection; (3) conventional LASSO shrinkage selection; (4) Bolasso method; (5) the two-stage hybrid procedure; (6) the bootstrap ranking procedure. We evaluated the performance of different variable selection methods for identifying key factors that can distinguish individuals infected with HBV from individuals free of HBV. The factors detected by different methods were applied to predict two groups of residents by using ensemble classification models, which were established using the ipred package within R software. A 10-fold stratified cross-validation approach was used for ensemble model building. To evaluate the overall predictive performance of these methods, both internal and external validation methods [30] were performed. We divided all the data into a training set including 80% of the original samples and a testing set including the remaining 20% of the samples. Prediction errors based on out-of-bag (OOB) samples and AUC values based on the 10-fold cross-validation were calculated for the internal and external validation set, respectively. Larger AUC and smaller OOB error indicate better predictive performance of model. The procedure was repeated 100 times and the average of the evaluation metrics and the corresponding standard deviation were reported.

## Results

### Performance evaluation based on simulation study

**Fig 1:**
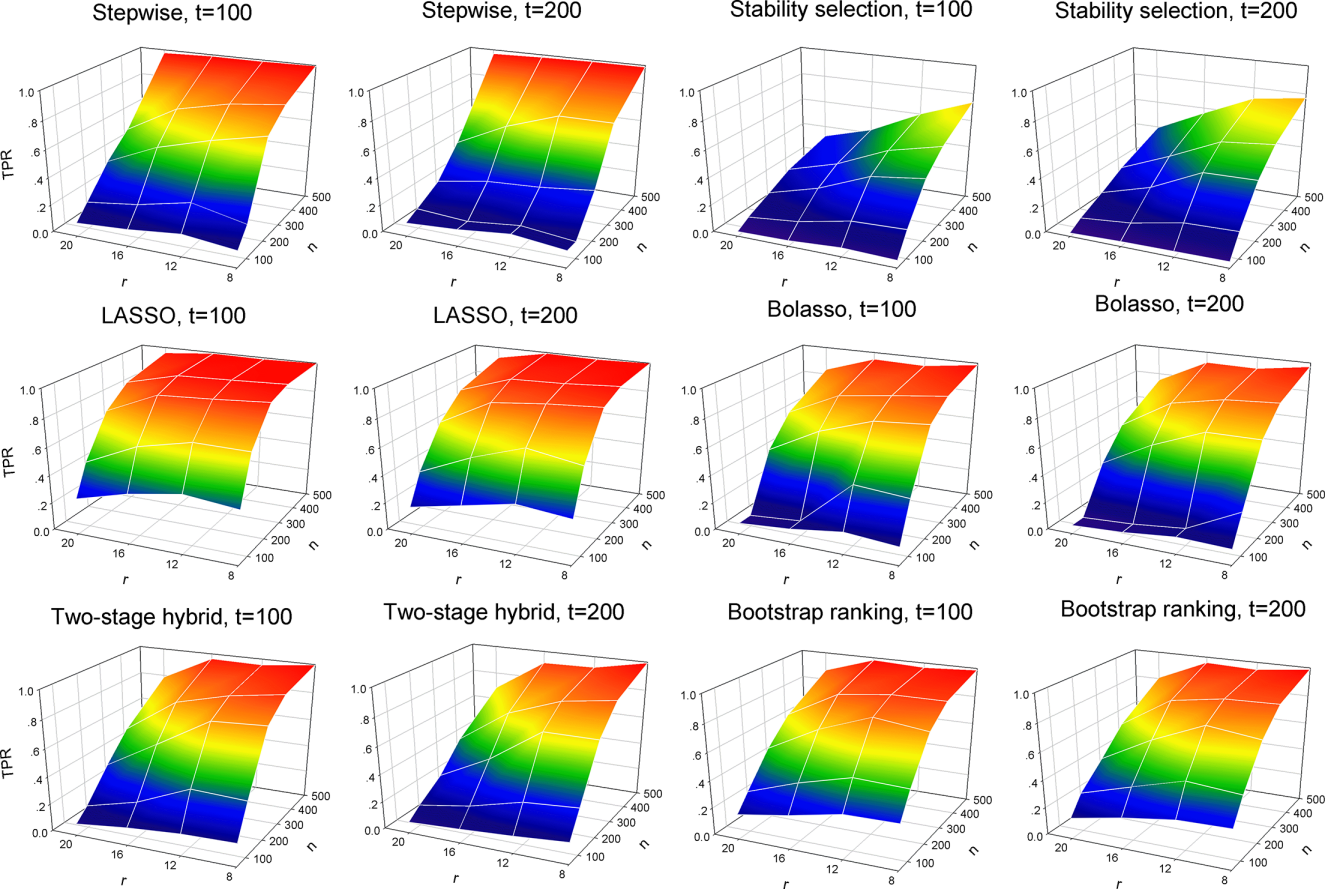
True positive rate (TPR) of the six variable selection methods with all simulated variables. 100 simulations were used to plot and evaluate the predictive performance of the six variable selection methods when the number of total predictors (*t* = 100 and 200), the number of true predictors (*r* = 8, 12, 16 and 20) and sample size (*n* = 50, 100, 200, 300 and 500) change. Left panel: the number of total predictors *t* = 100; right panel: the number of total predictors *t* = 200. Six compared variable selection methods: stepwise, stability selection, LASSO, Bolasso, two-stage hybrid and bootstrap ranking procedures.

Fig 1 presents the changing TPR of the six methods when the number of total predictors, the number of non-zero predictors and sample size vary. A higher TPR indicates better performance of identification of informative predictors from a pool of simulated variables. In the left panel corresponding to the number of total predictors *t* = 100, the TPR of the six methods increased continuously with increasing sample size and finally smoothly converged. In addition, with the increasing number of true predictors, the TPR significantly decreased. A similar pattern appeared in the right panel of Fig 1 when the number of total predictors *t* = 200. In fact, the results of the simulation study demonstrated that the identification of informative predictors became difficult when data with many covariates and small sample size was analyzed. The TPRs of variable selection using LASSO, Bolasso and the two proposed methods had a more clear rise than the stepwise selection and stability selection models when sample size reached a moderate scale, for example *n* = 200. Compared with the stepwise selection and stability selection models, the LASSO, Bolasso and the two newly proposed procedures presented a more favorable performance of correctly identifying influential predictors with non-zero coefficients.

**Fig 2:**
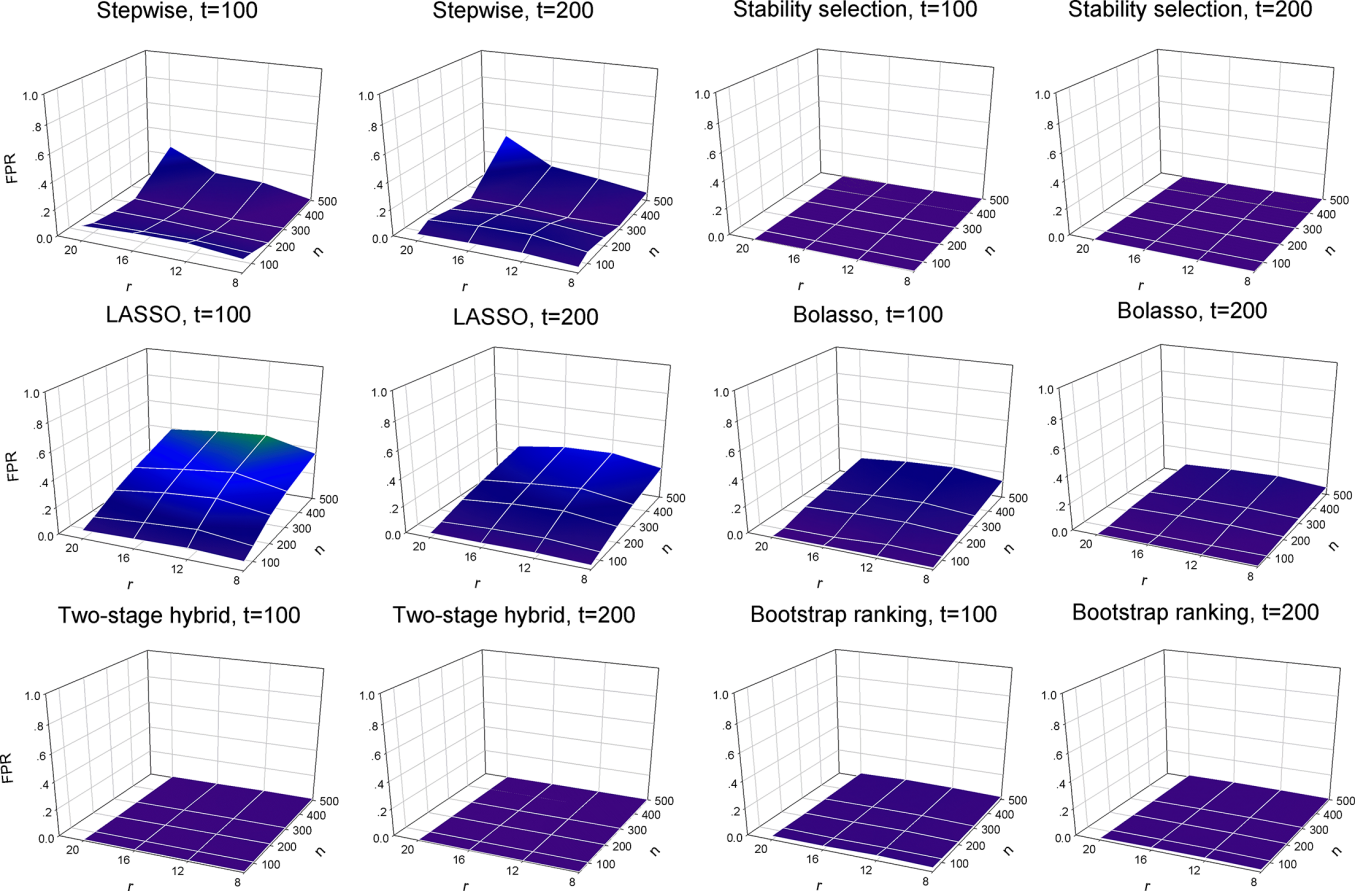
False positive rate (FPR) of the six variable selection methods with all simulated variables. 100 simulations were used to plot and evaluate the predictive performance of six variable selection methods when the number of total predictors (*t* = 100 and 200), the number of true predictors (*r* = 8, 12, 16 and 20) and sample size (*n* = 50, 100, 200, 300 and 500) change. Left panel: the number of total predictors *t* = 100; right panel: the number of total predictors *t* = 200. Six compared variable selection methods: stepwise, stability selection, LASSO, Bolasso, two-stage hybrid and bootstrap ranking procedures.

Fig 2 shows the changing FPR of the variable selection when the number of total predictors, the number of non-zero predictors and sample size vary. A higher FPR indicates a higher identification rate of irrelevant predictors (noise predictors) from a pool of simulated variables. For the stepwise selection technique, the FPR increased when the number of truly non-zero predictors was large (*r* = 20) in the cases of both *t* = 100 and *t* = 200. Based on the selection frequency of significant variables, a larger sample size increased the variable selection efficiency of the stepwise selection technique (Fig A in S1 File). The stability selection method selected non-zero predictors with significantly low FPR (Fig 2 and Fig B in S1 File), suggesting it could effectively eliminate noise variables during variable selection. For the conventional LASSO model, FPR remarkably increased with larger sample size (Fig 2). The increasing FPR indicated some noise variables were selected by the LASSO model as statistically significant predictors. This could be confirmed based on the frequency plot showing selection of each variable during the 100 simulations (Fig C in S1 File). The Bolasso model improved the problem of larger FPR to some degree in contrast to conventional LASSO (Fig 2 and Fig D in S1 File). More notably, the proposed procedures of the two-stage hybrid and bootstrap ranking provided efficient control over the increasing FPR during variable selection, when sample size became larger, to an extent comparable with the stability selection model (Fig 2). A larger sample size increased the variable selection efficiency of the two proposed procedures (Figs E and F in S1 File). When analyzing data with a large sample size, for example *n* = 500, the two proposed procedures were comparable to the stepwise selection technique in reducing the number of false positives. Specifically, they both outperformed the stepwise selection technique when sample size was relatively small, for example *n* = 100, in our simulation study.

**Fig 3:**
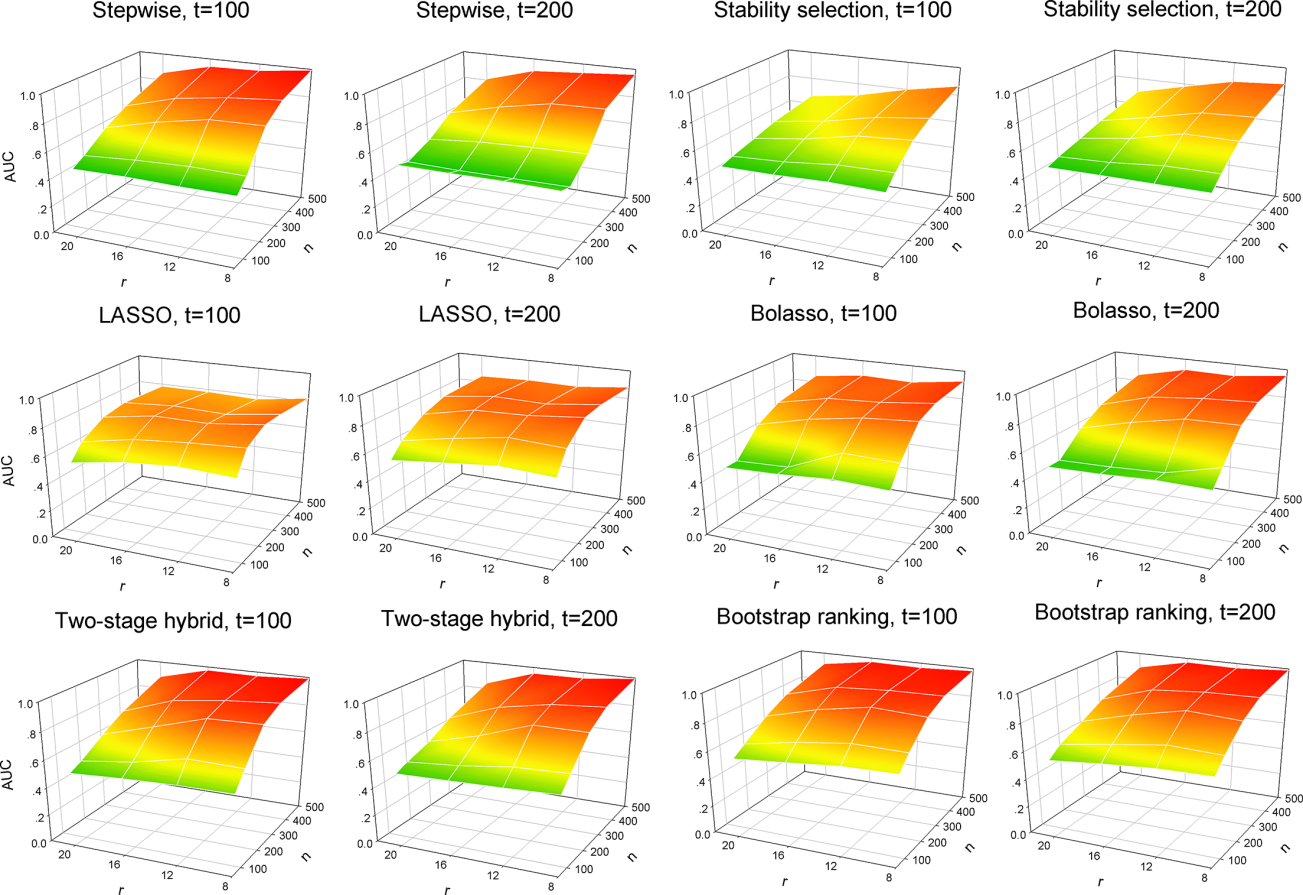
Areas under the ROC curve (AUC) of the six variable selection methods with all simulated variables. 100 simulations were used to plot and evaluate the predictive performance of six variable selection methods when the number of total predictors (*t* = 100 and 200), the number of true predictors (*r* = 8, 12, 16 and 20) and sample size (*n* = 50, 100, 200, 300 and 500) change. Left panel: the number of total predictors *t* = 100; right panel: the number of total predictors *t* = 200. Variable selection methods: stepwise, stability selection, LASSO, Bolasso, two-stage hybrid and bootstrap ranking procedures.

**Fig 4:**
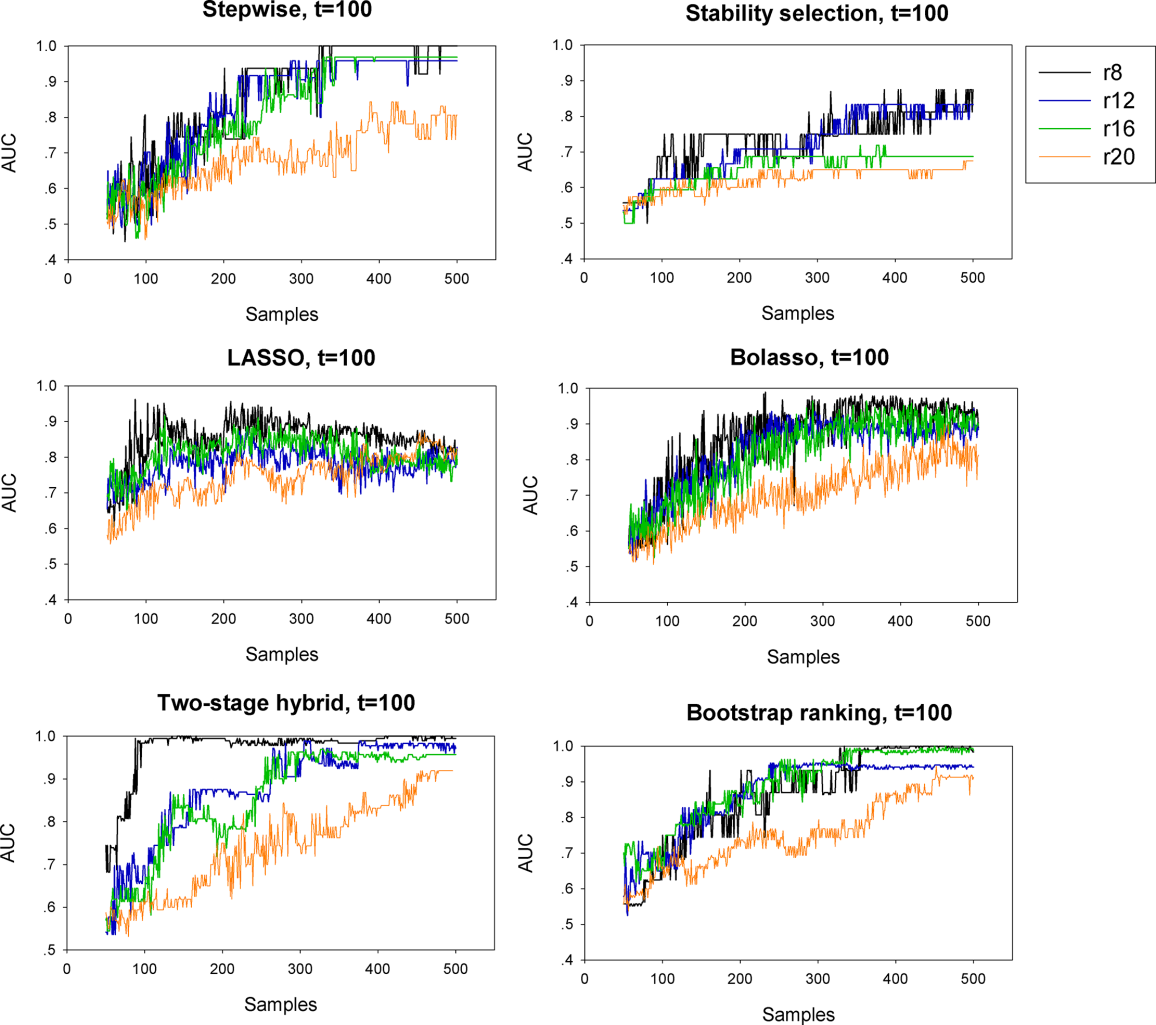
Predictive performance of the six variable selection methods. Asymptotic analysis of the metric AUC was used to evaluate the predictive performance of the six variable selection methods when sample size gradually increased from 50 to 500 when the total number of predictors *t* = 100. Variable selection methods: stepwise, stability selection, LASSO, Bolasso, two-stage hybrid and bootstrap ranking procedures.

The AUC was used for evaluating the overall performance of variable selection and a higher AUC indicated a good balance between the TPR and FPR. In practice, a model with high TPR and low FPR during variable selection is preferable. For the stepwise selection technique, stability selection, Bolasso, two-stage hybrid and bootstrap ranking methods, the AUC values increased continuously with larger sample size (Fig 3). For the LASSO, the AUC values showed a rising trend when sample size continuously increased, but tended to decline when sample size reached a large number. This was due to the fact that the LASSO selected variables with a high probability of false positives when sample size increased, which would reduce the overall power of selection. The two proposed procedures had better performance than the stepwise selection, stability selection and Bolasso methods, especially in cases with small sample size, for example *n* = 100 and 200. Fig 4 presents the continuously changing power in selecting truly non-zero variables with the sample size increasing when the total number of predictors *t* = 100. These results confirmed the findings above from the analysis of TPR and FPR. When the effects of relevant predictors turn smaller, for example in the sensitivity analysis corresponding to two groups of small values of non-zero coefficients, the AUC values had a similar variation pattern for each model when compared to the originally designed set of non-zero coefficients (Figs G and H in S1 File). It was observed that the AUC of variable selection using the LASSO model tended to decline along with increasing sample size. However, the increasing sample size enhanced the detection ability of relevant variables for the two proposed procedures. An underestimation of small effect predictors of the two newly proposed procedures was not observed. In total, the two procedures had competitive performance when compared to other methods, irrespective of sample size, the number of truly non-zero predictors and total predictors.

### Performance evaluation based on the studying of HBV infection in Yuexiu residents

Based on the univariate analysis in Table 1, factors including age, height, weight, marital status, education level, exercise frequency, family history of HBV infection, personal history of HBV infection and personal history of hepatitis B vaccination were associated with HBV infection (all *P*<0.05). Elder residents had a higher infection rate of HBV than the younger (*P*<0.05). Heavier and taller residents were more likely to be infected than those light weighted and less tall individuals (*P*<0.05). The higher infection rate of HBV was significantly associated with residents who were single, received a superior education, and got less exercise (all *P*<0.05). Residents with a family and personal history of HBV infection tended to have a high risk for HBV infection than those who had no history of the relevant disease (*P*<0.05). Note that residents who had been given the hepatitis B vaccination had less chance of being infected (*P*<0.05).

**Table 1:** Basic characteristics and investigated factors between residents infected with hepatitis B virus (HBV) and HBV-free residents in the Yuexiu district of Guangdong, China.

	HBV infected (N = 335)	HBV free (N = 5022)	*P*-value
Age (years, mean±sd)	54.33±13.43	48.88±22.34	0.000
Gender			0.595
*males*	107 (31.9%)	1675 (33.4%)	
*females*	228 (68.1%)	3347 (66.6%)	
Height	160.08±9.25	155.28±13.04	0.000
Weight	59.96±9.87	54.52±14.39	0.000
Career			0.439
*leaders of enterprise unit*	7 (2.1%)	134 (2.7%)	
*technical personnel*	27 (8.1%)	383 (7.6%)	
*handle affairs personnel*	23 (6.9%)	350 (7.0%)	
*commercial and service personnel*	41 (12.2%)	445 (8.9%)	
*agricultural*, *forestry and fishery production personnel*	2 (0.6%)	34 (0.7%)	
*transportation equipment operators*	21 (6.3%)	263 (5.2%)	
*others*	214 (63.9%)	3413 (68.0%)	
Marital status			0.000
*single*	16 (4.8%)	1097 (21.8%)	
*married*	299 (89.3%)	3536 (70.4%)	
*widowed*	18 (5.4%)	322 (6.4%)	
*divorce*	2 (0.6%)	67 (1.3%)	
Nationality			0.706
*Han nationality*	323 (96.4%)	4861 (96.8%)	
*minority nationality*	12 (3.6%)	161 (3.2%)	
Frequency of alcohol consumption			0.458
*no*	301 (89.9%)	4628 (92.2%)	
*occasionally*	26 (7.8%)	304 (6.1%)	
*often*	3 (0.9%)	42 (0.8%)	
*always*	5 (1.5%)	48 (1.0%)	
Smoking habit			0.298
*smoking*	29 (8.7%)	344 (6.8%)	
*give up smoking*	13 (3.9%)	153 (3.0%)	
*never smoking*	293 (87.5%)	4525 (90.1%)	
Education level			0.000
*illiteracy*	6 (1.8%)	175 (3.5%)	
*primary school*	42 (12.5%)	1201 (23.9%)	
*middle school*	107 (31.9%)	1246 (24.8%)	
*high and vocational school*	118 (35.2%)	1471 (29.3%)	
*undergraduate and above*	62 (18.5%)	929 (18.5%)	
Exercise frequency			0.001
*every day*	102 (30.4%)	1861 (37.1%)	
*more than once per week*	58 (17.3%)	1075 (21.4%)	
*occasionally*	74 (22.1%)	943 (18.8%)	
*never*	101 (30.1%)	1143 (22.8%)	
Family history of HBV infection			0.000
*no*	301 (89.9%)	4914 (97.8%)	
*yes*	34 (10.1%)	108 (2.2%)	
Stay abroad for more than three months			0.744
*no*	332 (99.1%)	4885 (99.3%)	
*yes*	3 (0.9%)	37 (0.7%)	
Personal history of HBV infection			0.000
*no*	213 (63.6%)	4968 (98.9%)	
*yes*	122 (36.4%)	54 (1.1%)	
Personal surgical history			0.915
*no*	287 (85.7%)	4313 (85.9%)	
*yes*	48 (14.3%)	709 (14.1%)	
Personal history of transfusion			0.915
*no*	329 (98.2%)	4936 (98.3%)	
*yes*	6 (1.8%)	86 (1.7%)	
Personal history of hepatitis B vaccination			0.000
*no*	296 (88.4%)	3424 (68.2%)	
*yes*	39 (11.6%)	1598 (31.8%)	

The six methods for identifying factors associate with HBV infection among residents were compared (Table 2). For LASSO, the optimal tuning parameter *λ* = 0.0017, corresponding to a minimal deviance of 0.2301, was chosen (Fig I in S1 File, A). The significant variables were estimated from the coefficient paths for the fitted LASSO model based on the optimal *λ* (Fig I in S1 File, B). Accordingly, the stepwise selection technique identified 9 significant factors, including gender, height, frequency of alcohol consumption, education level, exercise frequency, family and personal history of HBV infection, personal history of hepatitis B vaccination, and personal surgical history, whereas conventional LASSO selected the largest number of significant factors except for age, smoking habit and staying abroad for more than three months per year. This finding suggested that LASSO was less conservative compared to the other methods and that features including height, weight, career, nationality and education level with very small coefficients may represent noise features. The stability selection method identified a sparser subset of significant factors than the stepwise selection and LASSO models in this empirical analysis and presented a low detection rate of noise variables, which was consistent with the simulation study. Bolasso identified relatively fewer variables than conventional LASSO in this empirical analysis. The proposed two-stage hybrid procedure identified three variables including family and personal history of HBV infection and personal history of hepatitis B vaccination, resulting a similar subset of significant factors as the stability selection model. In particular, the bootstrap ranking procedure identified an optimum sparse subset of relevant factors among the compared models. This empirical analysis presented two proposed procedures that effectively detected the most informative predictors from a pool of candidate variables.

**Table 2:** Significant factors selected using the six variable selection models and the corresponding estimations of regression coefficients. The six compared variable selection methods: stepwise, stability selection, LASSO, Bolasso, two-stage hybrid and bootstrap ranking procedures.

Variable name	stepwise’s *β*	stability selection’s *β*	LASSO’s *β*	Bolasso’s *β*	two-stage hybrid’s *β*	bootstrap ranking’s *β*
Age	-	-	-	-	-	-
Gender	0.6044	-	0.3737	0.4855	-	-
Height	0.0399	-	0.0222	0.0362	-	-
Weight	-	-	0.0073	-	-	-
Career	-	-	0.0152	-	-	-
Marital status	-	-	0.0949	-	-	-
Nationality	-	-	0.0124	-	-	-
Frequency of alcohol consumption	0.2801	-	0.1962	-	-	-
Education level	-0.0977	-	-0.0217	-	-	-
Exercise frequency	0.1216	-	0.0956	0.1221	-	-
Smoking habit	-	-	-	-	-	-
Family history of HBV infection	0.7806	0.7632	0.6726	0.7406	0.2317	-
Personal history of HB vaccination	-0.889	-1.1157	-0.729	-0.9182	-0.5203	-1.127
Personal history of HBV infection	3.7437	3.7035	3.6597	3.6888	3.7164	3.766
Personal surgical history	-0.3707	-	-0.2323	-	-	-
Personal history of transfusion	-	-	-0.1813	-0.6444	-	-
Stay abroad for more than three months annually	-	-	-	-	-	-

In order to validate the predictive performance of the factors identified by the six methods to distinguish residents infected with HBV from HBV-free residents, we used the internal and external validation methods (Table 3). The prediction model with fewer predictors identified by the stability selection method and the two newly proposed procedures had the minimum OOB prediction errors and the maximal AUCs, demonstrating that these three methods outperformed the other methods with respect to the identification of the most informative factors. The standard deviations of the model evaluation metrics based on 100 replicates were consistently small for the compared models. However, it is worth noting that the bootstrap ranking procedure had the optimal predictive performance based on the least number of factors.

**Table 3:** Predictive performance of factors identified by the six variable selection methods to distinguish residents infected with hepatitis B virus (HBV) from the HBV-free residents. Internal and external validation methods were used to compare the variable selection methods. Internal validation: average out-of-bag (OOB) sample prediction error based on 100 replicates was used to evaluate the performance for the six methods by using training set (80% of the total samples), followed by a testing set (20% of the total samples). External validation: average 10-fold cross-validated area under the ROC curve (AUC) was used to evaluate the performance for the six methods. Variable selection methods: stepwise, stability selection, LASSO, Bolasso, two-stage hybrid and bootstrap ranking procedures. The mean of the evaluation metric and the corresponding standard deviation (SD) based on 100 replicates are presented as the mean (SD).

Validation	Metrics	stepwise	stability selection	LASSO	Bolasso	two-stage hybrid	bootstrap ranking
Internal	Average OOB error (Training set)	0.0652(0.0011)	0.0511(0.0000)	0.0607(0.0010)	0.0618(0.0008)	0.0511(0.0000)	0.0511(0.0000)
Internal	Average OOB error (Testing set)	0.0537(0.0020)	0.0448(0.0002)	0.0478(0.0014)	0.0551(0.0021)	0.0448(0.0002)	0.0448(0.0000)
External	Average AUC	0.6587(0.0081)	0.7045(0.0000)	0.6572(0.0089)	0.6689(0.0079)	0.7045(0.0000)	0.7045(0.0000)

## Discussion

Two newly proposed variable selection algorithms, the two-stage hybrid and bootstrap ranking procedures, were investigated in this work. Simulation studies revealed a high power and a low identification rate of irrelevant variables with the two proposed procedures during variable selection. Use of these algorithms in empirical analysis based on a large-scale epidemiology survey of HBV infection-relevant factors in community residents demonstrated that the procedures both were competitive or more favorable when compared with methods used in current practice.

The basis of the two-stage hybrid method is to establish a hybrid procedure for variable selection based on a LASSO-type penalized regression approach. This is achieved through sequentially combining the conventional LASSO and adaptive LASSO models, taking into consideration the optimal solution of the tuning parameter and weight vector for model penalization. We used the coordinate descent algorithm [23] for LASSO estimation because the algorithm was extremely efficient for fitting the entire LASSO regularization path in a pathwise fashion for generalized linear models. The optimal tuning parameter in the LASSO regression model can be selected using prediction error, and the *K*-fold cross-validation approach is an unbiased way to guide this choice [24]. In this study, a 10-fold cross-validation method was employed to select the optimal *λ*_1_ for the two-stage hybrid method. The principle of the 10-fold cross-validation method is to randomly partition the original sample into ten subsamples. Of these subsamples, one single subsample is retained as the validation set for testing the model, and the remaining subsamples are used for training data. This procedure is repeated 10 times, and the results are averaged to give a robust performance evaluation [31]. We evaluated the predictive performance at each value of the tuning parameter, chose the LASSO model corresponding to the best performance, and selected variables at the optimal tuning parameter. The parameter *λ*_2_ of the adaptive LASSO model was tuned in a manner similar to the LASSO model. For the proposed bootstrap ranking procedure, we used several bootstrap samples of the original data for estimating consistent coefficients in the LASSO model and intersected the non-zero coefficients according to Bolasso method [26]. However, instead of directly intersecting the non-zero estimates in the LASSO model, we generated a matrix of variable importance according to the estimate of coefficient for each variable, and intersected the selected variables which had the non-zero coefficients to obtain robust selection. By running the LASSO model in multiple bootstrap samples, the average estimation of coefficients was applied to detect a panel of the most significant variables in order to alleviate the over-selection problem of the conventional LASSO model.

Simulation studies and empirical analysis based on a large-scale epidemiology survey of relevant factors for HBV infection among community residents were performed to compare the two proposed procedures and other alternatives. The simulation studies revealed that conventional LASSO outperformed the stepwise selection and stability selection procedures in terms of the TPR metric, especially when analyzing data with more covariates (Fig 1). In addition, LASSO selected variables with slightly higher TPR than Bolasso and our two proposed procedures when sample size was relatively small, for example *n* = 100 and 200. However, when sample size increased the LASSO model tended to identify many truly zero coefficients as non-zero coefficients, resulting in a redundant set of noise variables (Fig 2), i.e. with a large sample size, a large number of irrelevant factors were identified to be significant by LASSO. The LASSO model usually selects the non-zero coefficients if they are not too small, and therefore tends to select many irrelevant covariates as having high probability [32–35]. This conclusion is supported by our simulation analysis. Although the performance of detecting truly relevant variables, using the stability selection method, was relatively inferior to the other methods used for comparison, stability selection had an obvious advantage in controlling the identification rate of false relevant variables. For eliminating noise variables, the two proposed procedures and the stability selection model were comparable, and they outperformed the stepwise selection technique and Bolasso model with respect to the FPR measurement (Fig 2). For the stepwise selection technique, the AIC selection criterion was used in this work because it can be widely extended to more generalized models. However, a wider variety of selection criteria to construct a stepwise variable selection model should be investigated and compared with the two proposed procedures in future studies. In total, the two-stage hybrid and bootstrap ranking procedures performed favorably when compared to other methods in terms of the AUC metric.

In the empirical analysis, the stepwise selection technique identified 9 potentially relevant factors whereas LASSO identified the largest number of factors. This finding was similar to the results of our simulation analysis, demonstrating that LASSO was less conservative compared to other methods in regard to practical data analysis. The proposed procedures effectively eliminated irrelevant variables to produce a sparse model and improve prediction based on the selected variables. The stability selection method had one significant advantage over the stepwise selection, LASSO and Bolasso models that stability selection identified relevant variables with lower FPR. Both the two-stage hybrid and the stability selection models identified three important factors associated with HBV infection in residents. Because the number of residents infected with HBV and that of HBV-free residents in the data was imbalanced, we used an ensemble classification model to investigate the significance of the factors detected by the six methods. In the data mining field, datasets that suffer from imbalanced class distributions occur when the number of samples that represent one class is much lower than the ones of the other classes, and ensemble-based methods have been proposed to address the class imbalance issue [36]. As assessed using the metrics of the OOB prediction error and AUC, the ensemble model with the least number of predictors identified by the proposed bootstrap ranking procedure had the optimal performance. Taking into consideration the results of both the simulation study and empirical analysis, our two newly proposed procedures can select the most informative predictors from a pool of variables, and are competitive with the other alternatives.

Because information on factors relevant to HBV infection in Yuexiu residents is not available, we attempted to fill this gap by using our models to evaluate HBV infection among community residents from Yuexiu and identify significant factors related to the infection of HBV. Using intensive simulations and an empirical analysis based on a study of HBV infection relevant factors in community residents, we identified three factors predominantly linked to HBV infection, including personal history of hepatitis B vaccination and family and personal history of HBV infection. The coefficient estimations (*β*s) for personal hepatitis B vaccination in the two-stage hybrid and bootstrap ranking procedures were -0.5203 and -1.1270, respectively. These results showed the important role of immunizing individuals with the hepatitis B vaccine to reduce the risk of HBV infection in residents [37, 38]. This study also found that the family and personal history of HBV infection exhibit a positive correlation with HBV infection of individual resident. In fact, HBV infection is a major public health problem and this type of virus is the leading cause of chronic hepatitis in the Asian population [39]. In China, the hepatitis B rate is particularly high and a majority of liver cancer and cirrhosis is hepatitis-B related [40, 41]. Risk factors for HBV infection including family history of HBV infection, personal history of vaccination, smoking, older age, male gender, poor sleep quality, occupation as a private small-businessman and history of surgical operations were found in Chinese population [42, 43]. Our results were consistent with previous findings supporting a significant association between family history and non vaccinated status and HBV infection. In contrast to other studies, known HBV risk factors such as a history of surgical operations, older age, male gender, occupation and smoking, which were also investigated in our study showed no significant difference between the HBV-infected residents and the HBV-free residents. Our two procedures had comparable predictive performance in the empirical analysis. However, the bootstrap ranking procedure yielded a sparser subset of factors, indicating that personal history of infection and non-vaccination were closely related to HBV infection in the studied population.

In summary, two improved variable selection algorithms using a LASSO-type penalty were proposed in this work. The proposed methods had a favorable performance in screening significant variables, and enhance our capability to derive new insights into epidemiological association analysis.

## Supporting Information

S1 File Contains the following files: Fig A. Frequency that each variable is selected for the stepwise variable selection method when sample size changes. Plot based on 100 simulations using various sample sizes (*n* = 100, 200, 300 and 500). Left panel: the number of true predictors *r* = 8; right panel: the number of true predictors *r* = 12. Red bars represent the selection frequency of significant variables (true non-zero predictors) and grey bars represent that of noise variables (true zero predictors) in the simulated data. **Fig B. Frequency that each variable is selected for the stability selection method when sample size changes.** Plot based on 100 simulations using various sample sizes (*n* = 100, 200, 300 and 500). Left panel: the number of true predictors *r* = 8; right panel: the number of true predictors *r* = 12. Red bars represent the selection frequency of significant variables (true non-zero predictors) and grey bars represent that of noise variables (true zero predictors) in the simulated data. **Fig C. Frequency that each variable is selected for the LASSO variable selection method when sample size changes.** Plot based on 100 simulations using various sample sizes (*n* = 100, 200, 300 and 500). Left panel: the number of true predictors *r* = 8; right panel: the number of true predictors *r* = 12. Red bars represent the selection frequency of significant variables (true non-zero predictors) and grey bars represent that of noise variables (true zero predictors) in the simulated data. **Fig D. Frequency that each variable is selected for the Bolasso variable selection method when sample size changes.** Plot based on 100 simulations using various sample sizes (*n* = 100, 200, 300 and 500). Left panel: the number of true predictors *r* = 8; right panel: the number of true predictors *r* = 12. Red bars represent the selection frequency of significant variables (true non-zero predictors) and grey bars represent that of noise variables (true zero predictors) in the simulated data. **Fig E. Frequency that each variable is selected for the two-stage hybrid variable selection method when sample size changes.** Plot based on 100 simulations using various sample sizes (*n* = 100, 200, 300 and 500). Left panel: the number of true predictors *r* = 8; right panel: the number of true predictors *r* = 12. Red bars represent the selection frequency of significant variables (true non-zero predictors) and grey bars represent that of noise variables (true zero predictors) in the simulated data. **Fig F. Frequency that each variable is selected for the bootstrap ranking variable selection method when sample size changes.** Plot based on 100 simulations using various sample sizes (*n* = 100, 200, 300 and 500). Left panel: the number of true predictors *r* = 8; right panel: the number of true predictors *r* = 12. Red bars represent the selection frequency of significant variables (true non-zero predictors) and grey bars represent that of noise variables (true zero predictors) in the simulated data. **Fig G. Sensitivity analysis based on the metric AUC to evaluate the performance of the compared methods when the number of true predictors (*r* = 8, 12, 16 and 20) and sample size (*n* = 50, 100, 200, 300 and 500) changes with respect to the small effect predictors of group 1.** A total number of variables *t* = 100 were simulated. Six compared methods: stepwise, stability selection, LASSO, Bolasso, two-stage hybrid and bootstrap ranking procedures. **Fig H. Sensitivity analysis based on the metric AUC to evaluate the performance of the compared methods when the number of true predictors (*r* = 8, 12, 16 and 20) and sample size (*n* = 50, 100, 200, 300 and 500) changes with respect to the small effect predictors of group 2.** A total number of variables *t* = 100 were simulated. Six compared methods: stepwise, stability selection, LASSO, Bolasso, two-stage hybrid and bootstrap ranking procedures. **Fig I. The estimation of tuning parameter *λ* and coefficients for the LASSO model.** (A): The deviance with error bar of the LASSO logistic regression model using a 10-fold cross-validation across different values of the tuning parameter (log-scale). The optimal model is the one with a deviance of 0.2301 when the tuning parameter reaches 0.0017. (B): The path of the estimated coefficients over a grid of values for *λ* and the selected variables corresponding to the optimal *λ*. **Table A. R codes of the two-stage hybrid procedure.** The R function TSLasso was used for establishing the two-stage hybrid procedure. **Table B. R codes of the bootstrap ranking procedure.** The R function Bootranking was used for establishing the bootstrap ranking procedure. **Table C. A de-identified dataset of this work was made publicly-available.**
(DOCX)
