# Supplementary material for: Improved Variable Selection Algorithm Using a LASSO-Type Penalty, with an Application to Assessing Hepatitis B Infection Relevant Factors in Community Residents
Source: PLoS One. 2015 Jul 27;10(7):e0134151. doi: 10.1371/journal.pone.0134151 (PMC4516242; doi:10.1371/journal.pone.0134151)
Supplement: S1 File — Plot based on 100 simulations using various sample sizes (n = 100, 200, 300 and 500). Left panel: the number of true predictors r = 8; right panel: the number of true predictors r = 12. Red bars represent the selection frequency of significant variables (true non-zero predictors) and grey bars represent that of noise variables (true zero predictors) in the simulated data. Fig B. Frequency that each variable is selected for the stability selection method when sample size changes. Plot based on 100 simulations using various sample sizes (n = 100, 200, 300 and 500). Left panel: the number of true predictors r = 8; right panel: the number of true predictors r = 12. Red bars represent the selection frequency of significant variables (true non-zero predictors) and grey bars represent that of noise variables (true zero predictors) in the simulated data. Fig C. Frequency that each variable is selected for the LASSO variable selection method when sample size changes. Plot based on 100 simulations using various sample sizes (n = 100, 200, 300 and 500). Left panel: the number of true predictors r = 8; right panel: the number of true predictors r = 12. Red bars represent the selection frequency of significant variables (true non-zero predictors) and grey bars represent that of noise variables (true zero predictors) in the simulated data. Fig D. Frequency that each variable is selected for the Bolasso variable selection method when sample size changes. Plot based on 100 simulations using various sample sizes (n = 100, 200, 300 and 500). Left panel: the number of true predictors r = 8; right panel: the number of true predictors r = 12. Red bars represent the selection frequency of significant variables (true non-zero predictors) and grey bars represent that of noise variables (true zero predictors) in the simulated data. Fig E. Frequency that each variable is selected for the two-stage hybrid variable selection method when sample size changes. Plot based on 100 simulations using [file pone.0134151.s001.docx]

Supporting Information for

**Improved variable selection algorithm using a LASSO-type penalty, with an application to assessing hepatitis B infection relevant factors in community residents**

Pi Guo^1, 2^, Fangfang Zeng^1, 2^, Xiaomin Hu^1, 2^, Dingmei Zhang^1, 2^, Shuming Zhu^1, 2^, Yu Deng^1, 2^, Yuantao Hao^1, 2, *^

^1^ Department of Medical Statistics and Epidemiology and Health Information Research Center, School of Public Health, Sun Yat-sen University, Guangzhou, Guangdong 510080, China

^2^ Laboratory of Health Informatics, Guangdong Key Laboratory of Medicine, Sun Yat-sen University, Guangzhou, Guangdong 510080, China

^*^ Corresponding author. E-mail: [haoyt@mail.sysu.edu.cn](mailto:haoyt@mail.sysu.edu.cn)

**Fig A. Frequency that each variable is selected for the stepwise variable selection method when sample size changes.** Plot based on 100 simulations using various sample sizes (*n* = 100, 200, 300 and 500). Left panel: the number of true predictors *r* = 8; right panel: the number of true predictors *r* = 12. Red bars represent the selection frequency of significant variables (true non-zero predictors) and grey bars represent that of noise variables (true zero predictors) in the simulated data.

**
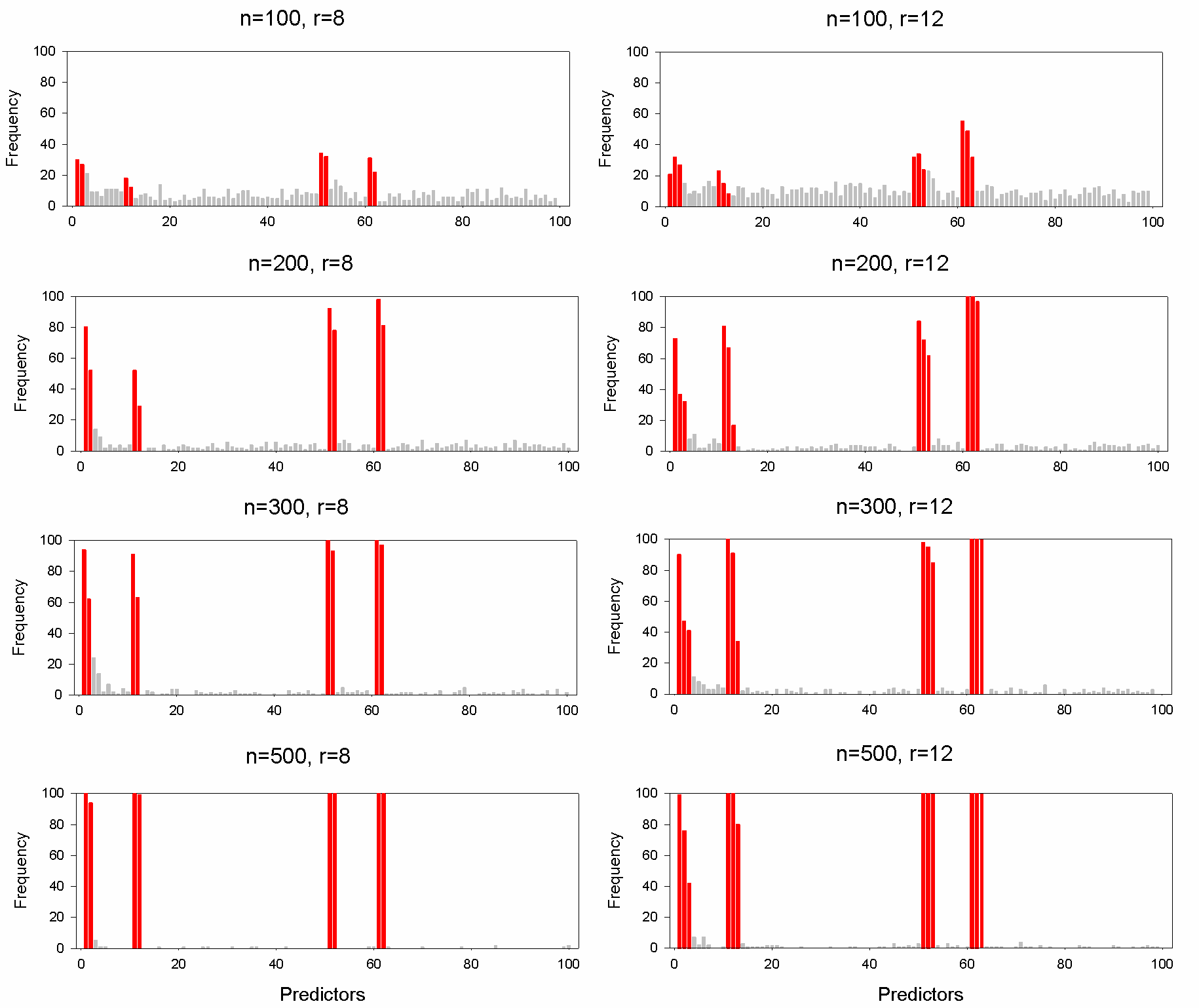
**

**Fig B. Frequency that each variable is selected for the stability selection method when sample size changes.** Plot based on 100 simulations using various sample sizes (*n* = 100, 200, 300 and 500). Left panel: the number of true predictors *r* = 8; right panel: the number of true predictors *r* = 12. Red bars represent the selection frequency of significant variables (true non-zero predictors) and grey bars represent that of noise variables (true zero predictors) in the simulated data.

**
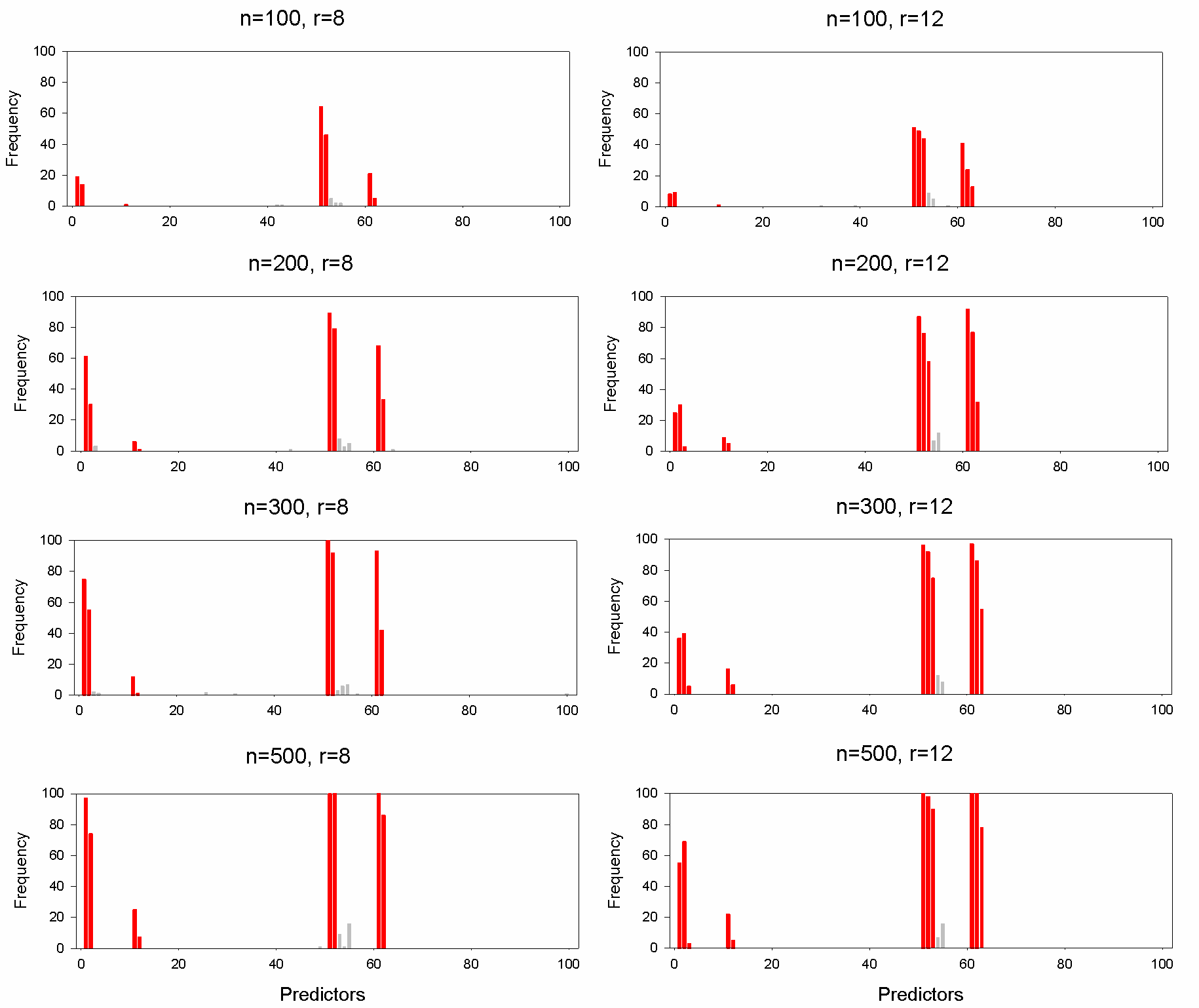
**

**Fig C. Frequency that each variable is selected for the LASSO variable selection method when sample size changes.** Plot based on 100 simulations using various sample sizes (*n* = 100, 200, 300 and 500). Left panel: the number of true predictors *r* = 8; right panel: the number of true predictors *r* = 12. Red bars represent the selection frequency of significant variables (true non-zero predictors) and grey bars represent that of noise variables (true zero predictors) in the simulated data.


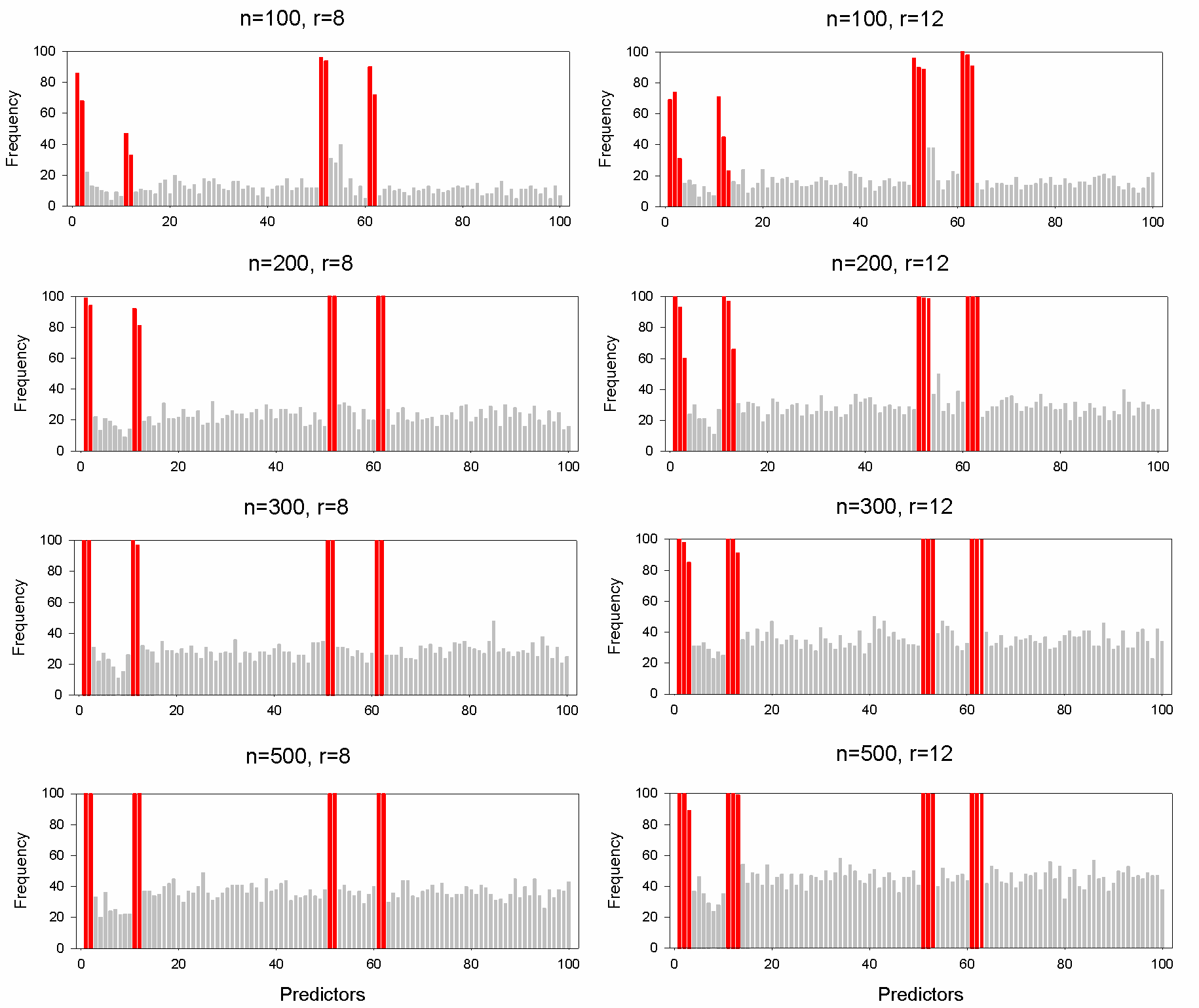


**Fig D. Frequency that each variable is selected for the Bolasso variable selection method when sample size changes.** Plot based on 100 simulations using various sample sizes (*n* = 100, 200, 300 and 500). Left panel: the number of true predictors *r* = 8; right panel: the number of true predictors *r* = 12. Red bars represent the selection frequency of significant variables (true non-zero predictors) and grey bars represent that of noise variables (true zero predictors) in the simulated data.


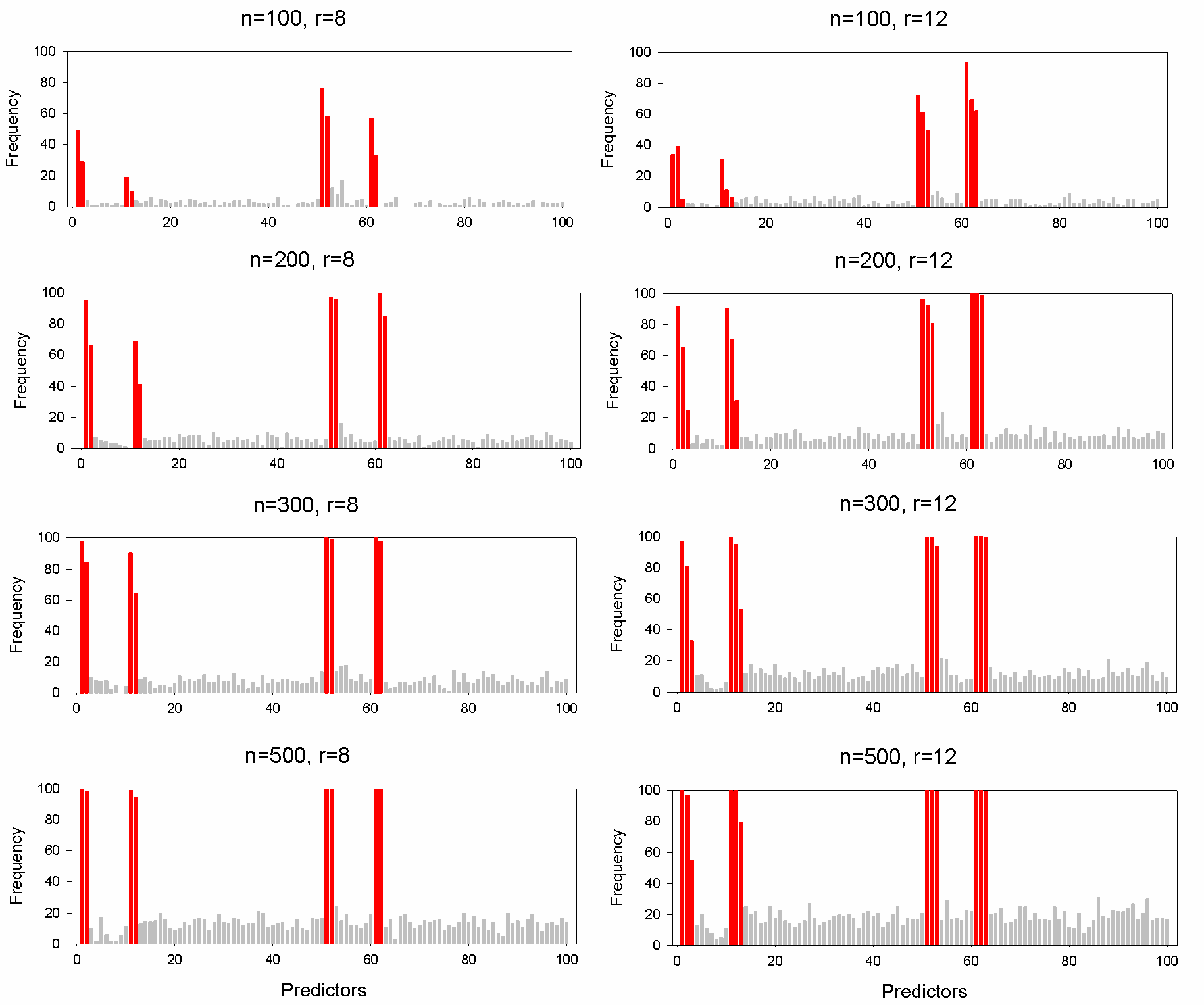


**Fig E. Frequency that each variable is selected for the two-stage hybrid variable selection method when sample size changes.** Plot based on 100 simulations using various sample sizes (*n* = 100, 200, 300 and 500). Left panel: the number of true predictors *r* = 8; right panel: the number of true predictors *r* = 12. Red bars represent the selection frequency of significant variables (true non-zero predictors) and grey bars represent that of noise variables (true zero predictors) in the simulated data.


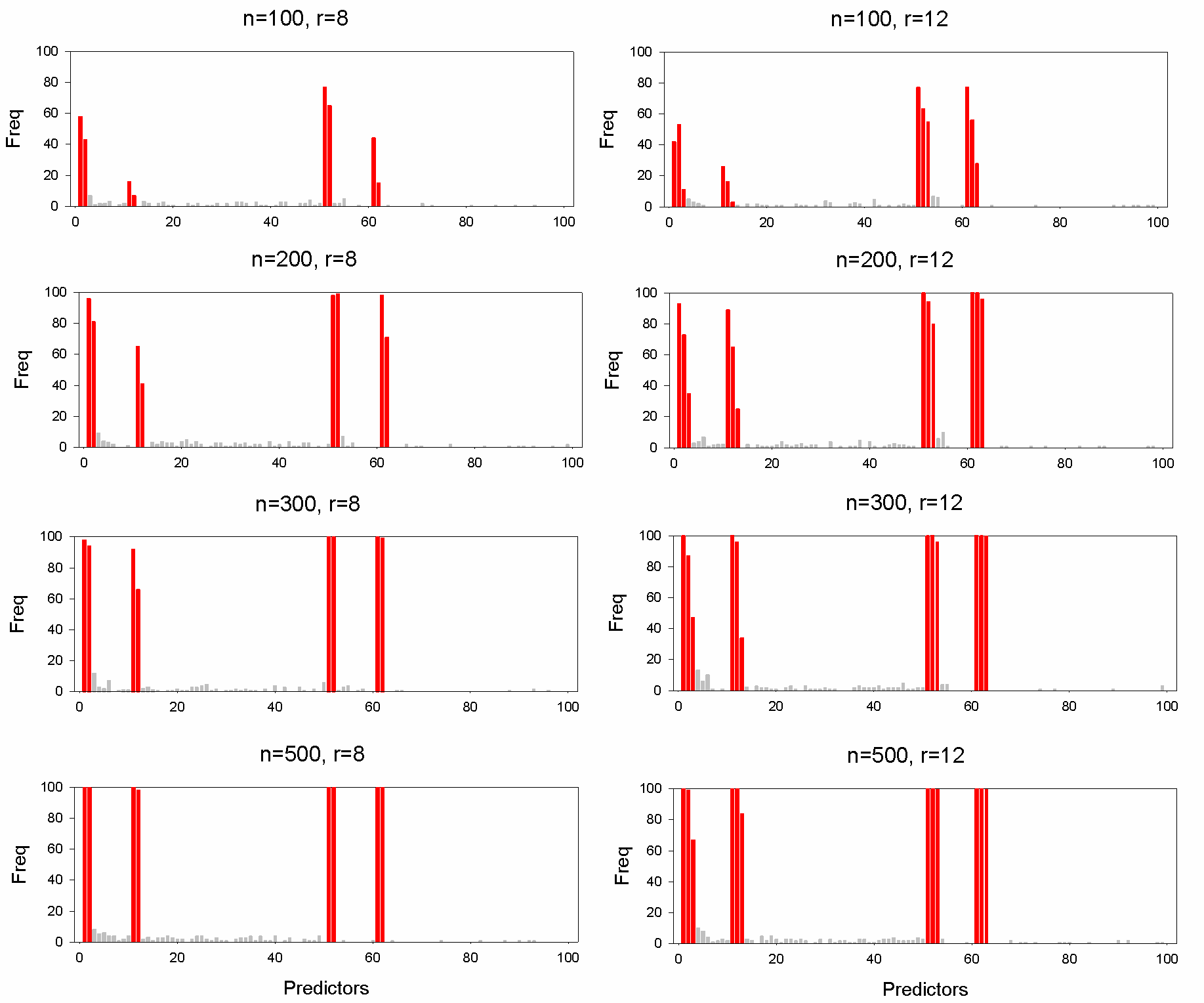


**Fig F. Frequency that each variable is selected for the bootstrap ranking variable selection method when sample size changes.** Plot based on 100 simulations using various sample sizes (*n* = 100, 200, 300 and 500). Left panel: the number of true predictors *r* = 8; right panel: the number of true predictors *r* = 12. Red bars represent the selection frequency of significant variables (true non-zero predictors) and grey bars represent that of noise variables (true zero predictors) in the simulated data.


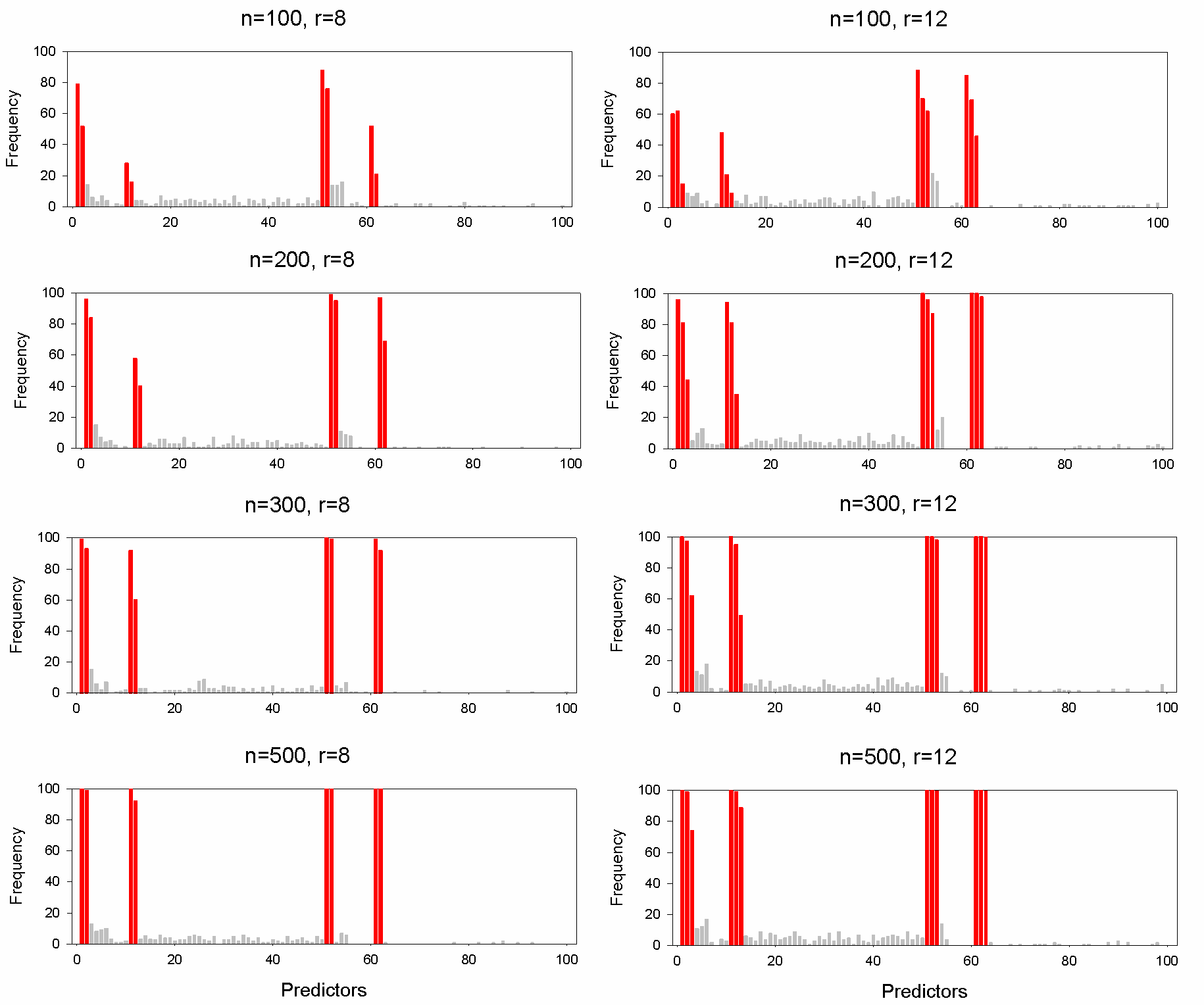


**Fig G. Sensitivity analysis based on the metric AUC to evaluate the performance of the compared methods when the number of true predictors (*r*=8, 12, 16 and 20) and sample size (*n*=50, 100, 200, 300 and 500) changes with respect to the small effect predictors of group 1.** A total number of variables *t*=100 were simulated. Six compared methods: stepwise, stability selection, LASSO, Bolasso, two-stage hybrid and bootstrap ranking procedures.


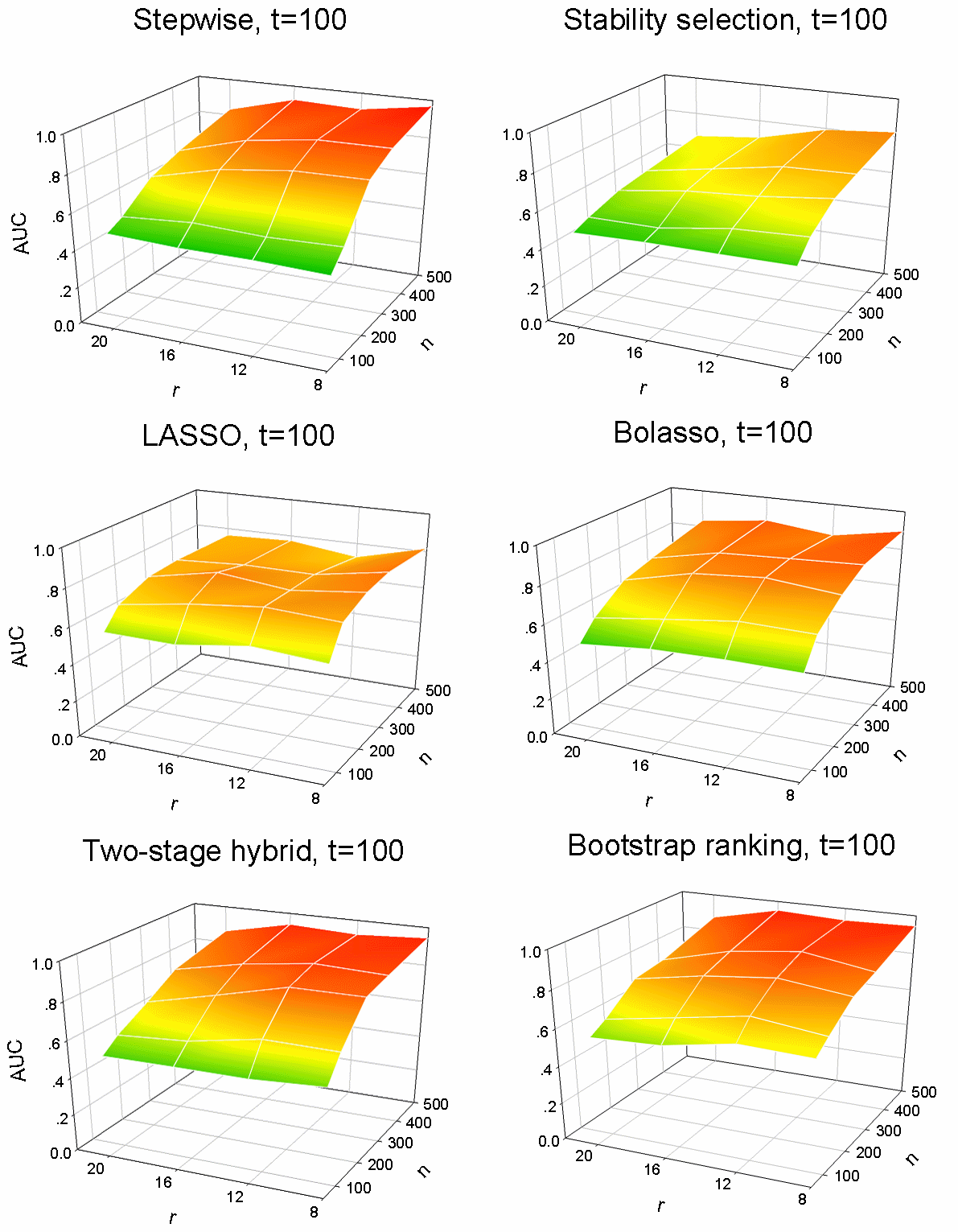


**Fig H. Sensitivity analysis based on the metric AUC to evaluate the performance of the compared methods when the number of true predictors (*r*=8, 12, 16 and 20) and sample size (*n*=50, 100, 200, 300 and 500) changes with respect to the small effect predictors of group 2.** A total number of variables *t*=100 were simulated. Six compared methods: stepwise, stability selection, LASSO, Bolasso, two-stage hybrid and bootstrap ranking procedures.


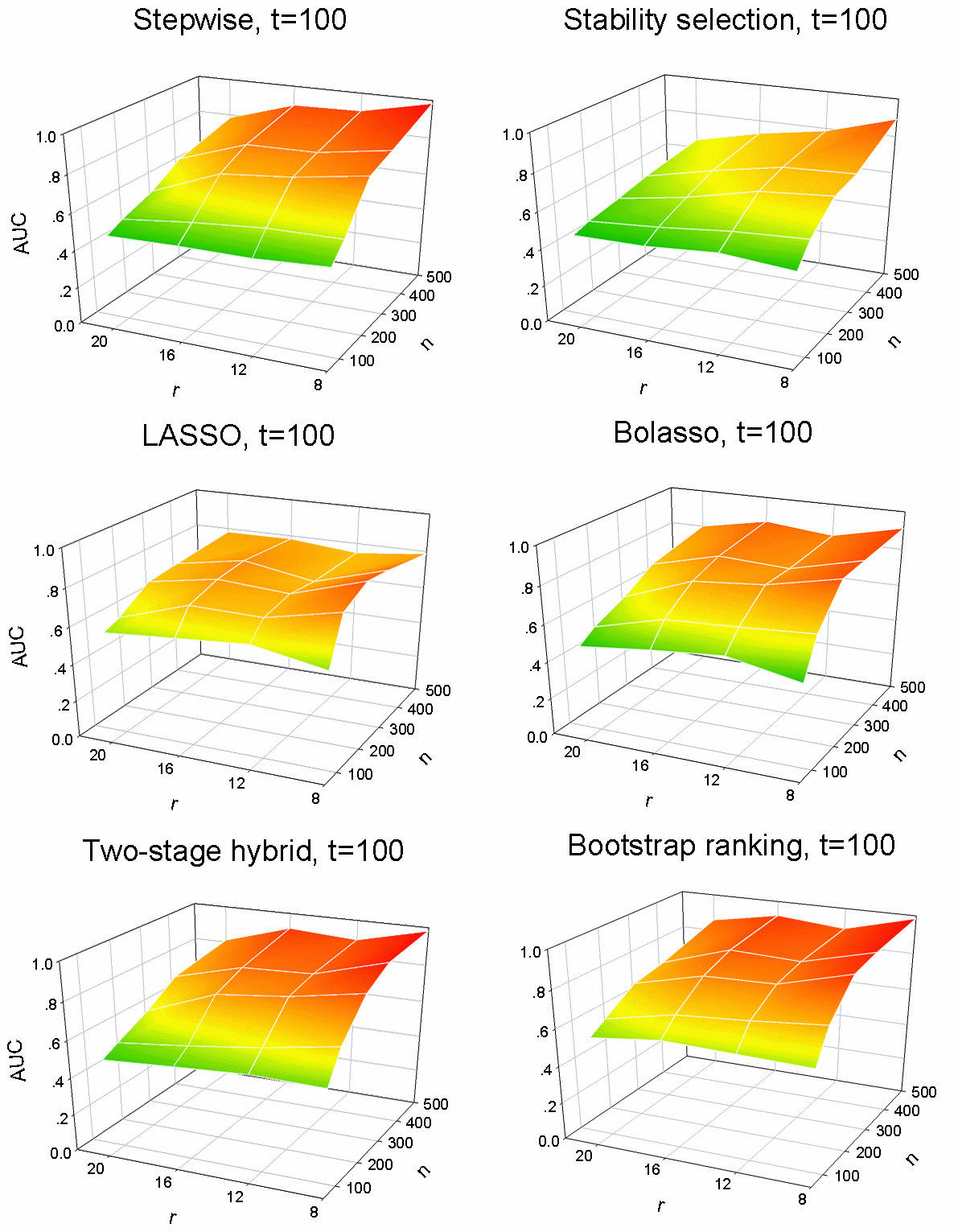


**Fig I. The estimation of tuning parameter $\lambda$ and coefficients for the LASSO model.** (A): The deviance with error bar of the LASSO logistic regression model using a 10-fold cross-validation across different values of the tuning parameter (log-scale). The optimal model is the one with a deviance of 0.2301 when the tuning parameter reaches 0.0017. (B): The path of the estimated coefficients over a grid of values for $\lambda$ and the selected variables corresponding to the optimal $\lambda$.


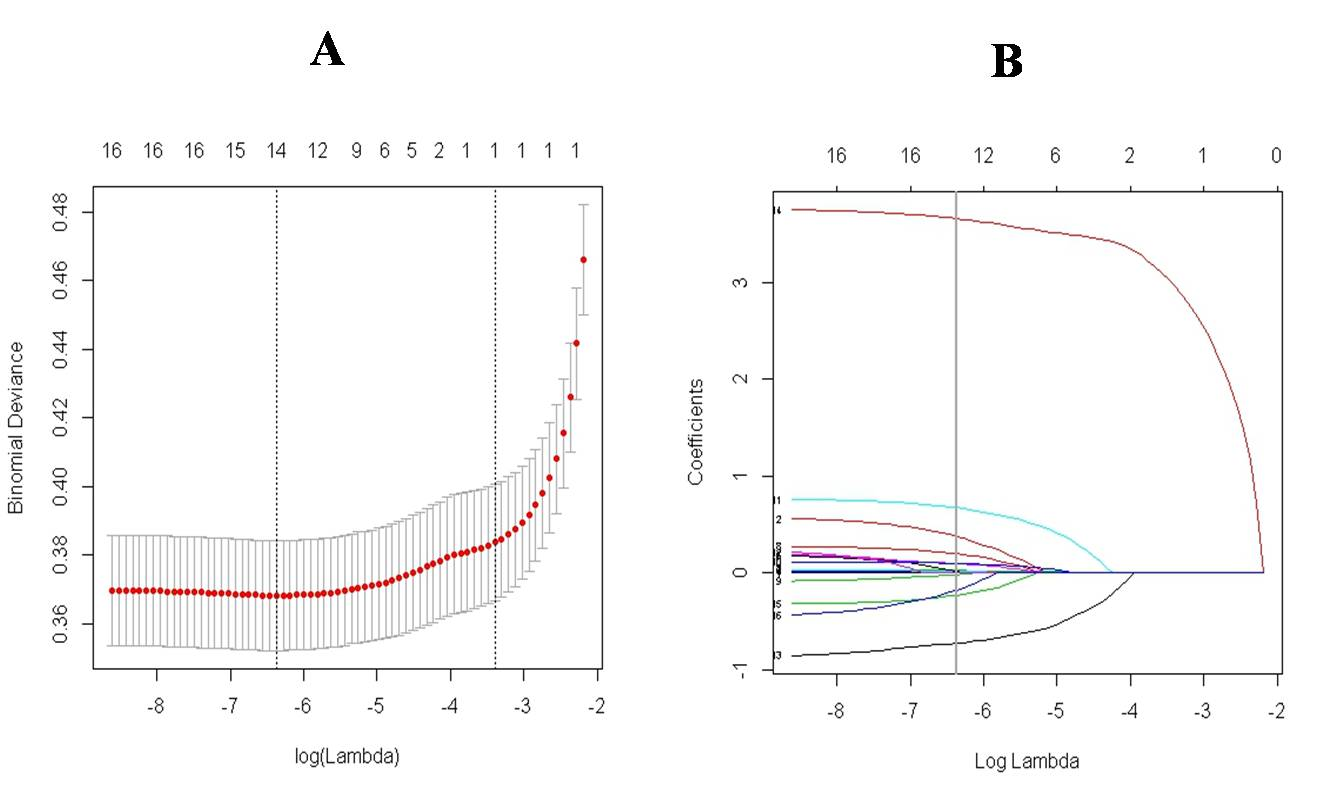


**Table A. R codes of the two-stage hybrid procedure.** The R function TSLasso was used for establishing the two-stage hybrid procedure.

| Line NO. | R codes and comments |
| --- | --- |
| 1  2  3  4  5  6  7  8  9  10  11  12  13  14  15  16  17  18  19  20  21  22  23  24  25  26  27  28  29  30  31  32  33  34  35  36  37  38  39  40  41  42  43  44  45  46  47 | # Installing the corresponding R packages for the first time  install.packages(c("glmnet", "lqa"))  # Loading R packages  library(glmnet)  library(lqa)  # Main function  # Input parameter: (x, y) for data; wfactor for computing weights; kfold for *K*-fold CV  TSLasso=function(x, y, wfactor=1, kfold=10, ...){  varx <- colnames(x)  rowx <- nrow(x)  n <- length(y)  if (rowx!=n){  stop("The number of rows in x is not equal to the length of y!")  }  # Stage 1: Set up the LASSO model  cvfit <- cv.glmnet(x=x, y=y, type.measure="deviance", nfold=kfold, family="binomial")  model.final <- cvfit$glmnet.fit  nzero <- as.matrix(coef(model.final, s=cvfit$lambda.min))  nzero <- as.matrix(nzero[rownames(nzero)!="(Intercept)",])  var_Lasso <- names(nzero[nzero[,1]!=0,])  cat("LASSO model finish!", "\n")  if(!is.null(var_Lasso)){  # Stage 2: Set up the adaptive LASSO model  x_Ada <- x[, var_Lasso]  y_Ada <- y  w <- (abs(as.vector(nzero[nzero[,1]!=0,])))^(-wfactor)  cvfit_Ada <- cv.lqa(y_Ada, x_Ada, lambda.candidates=list(seq(0.001,5,by=0.01)), family=binomial(), penalty.family=lasso, n.fold=5, loss.func="aic.loss", intercept=TRUE)  lamd <- cvfit_Ada$lambda.opt  fit_Ada <- lqa(y_Ada~x_Ada, family=binomial(), penalty=adaptive.lasso(lambda=lamd, al.weights=w))  var_Ada1 <- round(fit_Ada$coef, 4)  var_Ada2 <- names(round(fit_Ada$coef, 4))  var_Ada3 <- var_Ada1[var_Ada2!="(Intercept)"]  names(var_Ada3) <- var_Lasso  var_Ada4 <- names(var_Ada3[var_Ada3!=0])  cat("Adaptive LASSO model finish!", "\n")  }  else {  print("No variables are selected by the LASSO method!")  var_Ada4 <-c()  }  res <- list(var_Ada1=var_Ada1, var_Ada4=var_Ada4)  res  }  # The end |

**Table B. R codes of the bootstrap ranking procedure.** The R function Bootranking was used for establishing the bootstrap ranking procedure.

| Line NO. | R codes and comments |
| --- | --- |
| 1  2  3  4  5  6  7  8  9  10  11  12  13  14  15  16  17  18  19  20  21  22  23  24  25  26  27  28  29  30  31  32  33  34  35  36  37  38  39  40  41  42  43  44  45  46  47  48  49  50  51  52  53  54  55  56  57  58  59  60  61  62  63  64  65  66  67  68  69  70 | # Installing the corresponding R packages for the first time  install.packages(c("glmnet", "segmented"))  # Loading R packages  library(glmnet)  library(segmented)  # Main function  # Input parameter: (x, y) for data; B for external validation; Boots for internal validation  Bootranking=function(x, y, B=10, Boots=100, kfold=10, ...){  varx <- colnames(x)  varx0 <- varx  nvar <- length(varx)  rowx <- nrow(x)  n <- length(y)  var_list_all <- vector("list",B)  if (rowx!=n){  stop("The number of rows in x is not equal to the length of y!")  }  if (nvar==1){  stop("The x matrix has only one variable!")  }  for (ij in 1:B){  # Step 1-variable selection  Nboot <- n  RecordM=matrix(0, nrow=Boots, ncol=nvar)  rownames(RecordM)=paste("BSample", 1:Boots, sep="")  colnames(RecordM)=varx  # Loop for internal validation  for(i in 1:Boots){  #sampling with replacement  repeat{  s <- sample(Nboot, replace=TRUE)  if(length(table(y[s])) >= 2 & length(table(y[-s])) >= 2)  break  }  BoostrapX <- x[s, ]  BoostrapY <- y[s]  #Set up the LASSO model  cvfit <- cv.glmnet(x=BoostrapX, y=BoostrapY, type.measure="deviance", nfold=kfold, family="binomial")  model.final <- cvfit$glmnet.fit  nzero <- as.matrix(coef(model.final, s=cvfit$lambda.min))  var_nz <- names(nzero[nzero[,1]!=0,])  #Computing the variable importance measure  for(i0 in 1:dim(nzero)[1]){  for(j0 in 1:nvar){  if (dimnames(nzero)[[1]][i0]==colnames(RecordM)[j0]){RecordM[i, j0]=nzero[i0, ]}  }  }  cat("Step 1-Current iteration: ", i, "\n")  } # The end of the inner for loop  # Internal validation end  # Set up a segment spline analysis on log-scale variable score curve  Impor <- abs(apply(RecordM, 2, mean))  score <- sort(Impor[Impor!=0])  index <- seq(1:length(score))  lin.mod <- lm(score~index)  segmented.mod <- segmented(lin.mod, seg.Z=~index, psi=list(index=c(index[length(index)/5])))  break_p <- round(segmented.mod$psi[1,2])  var_list_all[[ij]] <- names(score[break_p:length(index)])  # Intersecting operation  if (length(var_list_all[[ij]])!=0){  varx0 <- intersect(var_list_all[[ij]], varx0)  cat("Step 2-Current iteration: ", ij, "\n")  }  } # The end of the outer for loop  varx0  }  # The end |

**Table C. A de-identified dataset of this work are made publicly-available.**

| No | AGE | GENDER | HEIGHT | WEIGHT | CAREER | MARITAL_STATUS | NATIONALITY | DRINKING_FREQUENCY | EDUCATION_LEVEL | EXERCISE_FREQUENCY | FAMILY_HISTORY_OF_HB | GO_OUT3MONTHS_PER_YEAR | HB_VACCINATION | HISTORY_OF_HB | HISTORY_OF_SURGERY | HISTORY_OF_TRANSFUSION | SMOKING_HABIT | HBSAG |
| --- | --- | --- | --- | --- | --- | --- | --- | --- | --- | --- | --- | --- | --- | --- | --- | --- | --- | --- |
| 1 | 2 | 2 | 89 | 12.5 | 8 | 1 | 1 | 1 | 1 | 1 | 1 | 1 | 2 | 1 | 1 | 1 | 1 | 1 |
| 2 | 3 | 2 | 90 | 15 | 8 | 1 | 1 | 1 | 1 | 4 | 1 | 1 | 2 | 1 | 1 | 1 | 1 | 1 |
| 3 | 3 | 2 | 90 | 12 | 8 | 1 | 1 | 1 | 1 | 4 | 1 | 1 | 2 | 1 | 1 | 1 | 1 | 1 |
| 4 | 3 | 2 | 90 | 11.5 | 8 | 1 | 1 | 1 | 6 | 3 | 1 | 1 | 2 | 1 | 1 | 1 | 1 | 1 |
| 5 | 3 | 1 | 92 | 14.5 | 8 | 1 | 1 | 1 | 1 | 4 | 1 | 1 | 2 | 1 | 1 | 1 | 1 | 1 |
| 6 | 3 | 1 | 96 | 14.5 | 8 | 1 | 1 | 1 | 1 | 4 | 1 | 1 | 2 | 1 | 1 | 1 | 1 | 1 |
| 7 | 3 | 1 | 97 | 14 | 8 | 1 | 1 | 1 | 1 | 4 | 1 | 1 | 2 | 1 | 1 | 1 | 1 | 1 |
| 8 | 3 | 1 | 98 | 14.4 | 8 | 1 | 1 | 1 | 1 | 4 | 1 | 1 | 2 | 1 | 1 | 1 | 1 | 1 |
| 9 | 3 | 1 | 102 | 16.2 | 8 | 1 | 1 | 1 | 1 | 2 | 1 | 1 | 2 | 1 | 1 | 1 | 1 | 1 |
| 10 | 4 | 1 | 100 | 15 | 8 | 1 | 1 | 1 | 1 | 2 | 1 | 1 | 2 | 1 | 1 | 1 | 1 | 1 |
| 11 | 4 | 1 | 102 | 15 | 8 | 1 | 1 | 1 | 3 | 4 | 2 | 1 | 2 | 1 | 1 | 1 | 1 | 1 |
| 12 | 4 | 1 | 104 | 14.5 | 8 | 1 | 1 | 1 | 6 | 2 | 1 | 1 | 2 | 1 | 1 | 1 | 1 | 1 |
| 13 | 4 | 1 | 107.1 | 18.5 | 8 | 1 | 1 | 1 | 1 | 1 | 2 | 1 | 2 | 1 | 1 | 1 | 1 | 1 |
| 14 | 5 | 2 | 102 | 15 | 8 | 1 | 1 | 1 | 1 | 4 | 1 | 1 | 2 | 1 | 1 | 1 | 1 | 1 |
| 15 | 5 | 2 | 107.5 | 15.8 | 8 | 1 | 1 | 1 | 3 | 4 | 1 | 1 | 1 | 1 | 1 | 1 | 1 | 1 |
| 16 | 5 | 1 | 102 | 20 | 8 | 1 | 1 | 1 | 1 | 2 | 1 | 1 | 2 | 1 | 1 | 1 | 1 | 1 |
| 17 | 5 | 1 | 110 | 24 | 8 | 1 | 1 | 1 | 1 | 4 | 1 | 1 | 2 | 1 | 1 | 1 | 1 | 1 |
| 18 | 6 | 2 | 110 | 18 | 8 | 1 | 1 | 1 | 1 | 1 | 1 | 1 | 2 | 1 | 1 | 1 | 1 | 1 |
| 19 | 6 | 2 | 110 | 17 | 8 | 1 | 1 | 1 | 1 | 4 | 1 | 1 | 2 | 1 | 1 | 1 | 1 | 1 |
| 20 | 6 | 2 | 119 | 19 | 8 | 1 | 1 | 1 | 1 | 2 | 1 | 1 | 1 | 1 | 1 | 1 | 1 | 1 |
| 21 | 6 | 2 | 126.5 | 26 | 8 | 1 | 1 | 1 | 2 | 2 | 1 | 1 | 2 | 1 | 1 | 1 | 1 | 1 |
| 22 | 6 | 1 | 105 | 15 | 8 | 1 | 1 | 1 | 1 | 1 | 1 | 1 | 2 | 1 | 1 | 1 | 1 | 1 |
| 23 | 6 | 1 | 107 | 19 | 8 | 1 | 1 | 1 | 6 | 2 | 1 | 1 | 2 | 1 | 1 | 1 | 1 | 1 |
| 24 | 6 | 1 | 110 | 18 | 8 | 1 | 1 | 1 | 1 | 2 | 1 | 1 | 2 | 1 | 1 | 1 | 1 | 1 |
| 25 | 6 | 1 | 114.5 | 18.8 | 8 | 1 | 1 | 1 | 1 | 2 | 1 | 1 | 1 | 1 | 1 | 1 | 1 | 1 |
| 26 | 6 | 1 | 121 | 22.6 | 8 | 1 | 1 | 1 | 2 | 1 | 1 | 1 | 2 | 1 | 1 | 1 | 1 | 1 |
| 27 | 7 | 2 | 103.4 | 16.5 | 8 | 1 | 1 | 1 | 3 | 1 | 1 | 1 | 2 | 1 | 1 | 1 | 1 | 1 |
| 28 | 7 | 2 | 110.5 | 17 | 8 | 1 | 1 | 1 | 2 | 3 | 1 | 1 | 1 | 1 | 1 | 1 | 1 | 1 |
| 29 | 7 | 2 | 111 | 19 | 8 | 1 | 1 | 1 | 2 | 2 | 1 | 1 | 2 | 1 | 1 | 1 | 1 | 1 |
| 30 | 7 | 2 | 111.5 | 16.5 | 8 | 1 | 1 | 1 | 2 | 3 | 1 | 1 | 2 | 1 | 1 | 1 | 1 | 1 |
| 31 | 7 | 2 | 112 | 20 | 8 | 1 | 1 | 1 | 3 | 1 | 1 | 1 | 2 | 1 | 1 | 1 | 1 | 1 |
| 32 | 7 | 2 | 112.5 | 20 | 8 | 1 | 1 | 1 | 2 | 1 | 1 | 1 | 2 | 1 | 1 | 1 | 1 | 1 |
| 33 | 7 | 2 | 113.5 | 20.5 | 8 | 1 | 1 | 1 | 3 | 1 | 1 | 1 | 2 | 1 | 1 | 1 | 1 | 1 |
| 34 | 7 | 2 | 113.5 | 19 | 8 | 1 | 1 | 1 | 2 | 1 | 1 | 1 | 2 | 1 | 1 | 1 | 1 | 1 |
| 35 | 7 | 2 | 113.5 | 18 | 8 | 1 | 1 | 1 | 2 | 3 | 1 | 1 | 2 | 1 | 1 | 1 | 1 | 1 |
| 36 | 7 | 2 | 114 | 20 | 8 | 1 | 1 | 1 | 2 | 1 | 1 | 1 | 2 | 1 | 1 | 1 | 1 | 1 |
| 37 | 7 | 2 | 114 | 18 | 8 | 1 | 1 | 1 | 2 | 1 | 1 | 1 | 2 | 1 | 1 | 1 | 1 | 1 |
| 38 | 7 | 2 | 117 | 22 | 8 | 2 | 1 | 1 | 2 | 3 | 1 | 1 | 2 | 1 | 1 | 1 | 1 | 1 |
| 39 | 7 | 2 | 117 | 20 | 8 | 1 | 1 | 1 | 2 | 1 | 2 | 1 | 2 | 1 | 1 | 1 | 1 | 1 |
| 40 | 7 | 2 | 117.5 | 23 | 8 | 1 | 1 | 1 | 2 | 1 | 1 | 1 | 2 | 1 | 1 | 1 | 1 | 1 |
| 41 | 7 | 2 | 118 | 25 | 8 | 1 | 1 | 1 | 2 | 2 | 1 | 1 | 2 | 1 | 1 | 1 | 1 | 1 |
| 42 | 7 | 2 | 118 | 24.5 | 8 | 1 | 1 | 1 | 2 | 1 | 1 | 1 | 2 | 1 | 1 | 1 | 1 | 1 |
| 43 | 7 | 2 | 118 | 24 | 8 | 1 | 1 | 1 | 2 | 1 | 1 | 1 | 2 | 1 | 1 | 1 | 1 | 1 |
| 44 | 7 | 2 | 118 | 23 | 8 | 1 | 1 | 1 | 2 | 1 | 1 | 1 | 2 | 1 | 1 | 1 | 1 | 1 |
| 45 | 7 | 2 | 118 | 22.5 | 8 | 1 | 1 | 1 | 2 | 1 | 1 | 1 | 2 | 1 | 1 | 1 | 1 | 1 |
| 46 | 7 | 2 | 118 | 22.5 | 8 | 1 | 1 | 1 | 2 | 1 | 1 | 1 | 2 | 1 | 1 | 1 | 1 | 1 |
| 47 | 7 | 2 | 118 | 18 | 8 | 1 | 1 | 1 | 2 | 4 | 1 | 1 | 2 | 1 | 1 | 1 | 1 | 1 |
| 48 | 7 | 2 | 118 | 17 | 8 | 1 | 1 | 1 | 2 | 1 | 1 | 1 | 2 | 1 | 1 | 1 | 1 | 1 |
| 49 | 7 | 2 | 118.5 | 20 | 8 | 1 | 1 | 1 | 2 | 1 | 1 | 1 | 2 | 1 | 1 | 1 | 1 | 1 |
| 50 | 7 | 2 | 119 | 24 | 8 | 1 | 1 | 1 | 2 | 2 | 1 | 1 | 2 | 1 | 1 | 1 | 1 | 1 |
| 51 | 7 | 2 | 119.5 | 25 | 8 | 1 | 1 | 1 | 2 | 3 | 1 | 1 | 2 | 1 | 1 | 1 | 1 | 1 |
| 52 | 7 | 2 | 119.6 | 23.6 | 8 | 1 | 1 | 1 | 2 | 1 | 1 | 1 | 2 | 1 | 1 | 1 | 1 | 1 |
| 53 | 7 | 2 | 120 | 26 | 8 | 1 | 1 | 1 | 2 | 2 | 1 | 1 | 2 | 1 | 1 | 1 | 1 | 1 |
| 54 | 7 | 2 | 120 | 24 | 8 | 1 | 1 | 1 | 3 | 1 | 1 | 1 | 2 | 1 | 1 | 1 | 1 | 1 |
| 55 | 7 | 2 | 120 | 23 | 8 | 1 | 1 | 1 | 2 | 4 | 1 | 1 | 2 | 1 | 1 | 1 | 1 | 1 |
| 56 | 7 | 2 | 120 | 23 | 4 | 2 | 1 | 1 | 3 | 2 | 1 | 1 | 2 | 1 | 1 | 1 | 1 | 1 |
| 57 | 7 | 2 | 120 | 21 | 8 | 1 | 1 | 1 | 2 | 3 | 1 | 1 | 2 | 1 | 1 | 1 | 1 | 1 |
| 58 | 7 | 2 | 120.5 | 24.5 | 8 | 1 | 1 | 1 | 2 | 1 | 1 | 1 | 2 | 1 | 1 | 1 | 1 | 1 |
| 59 | 7 | 2 | 120.5 | 23 | 8 | 1 | 1 | 1 | 2 | 3 | 1 | 1 | 2 | 1 | 1 | 1 | 1 | 1 |
| 60 | 7 | 2 | 120.6 | 25.5 | 8 | 1 | 1 | 1 | 2 | 1 | 1 | 1 | 2 | 1 | 1 | 1 | 1 | 1 |
| 61 | 7 | 2 | 120.8 | 23 | 8 | 1 | 1 | 1 | 2 | 1 | 1 | 1 | 2 | 1 | 1 | 1 | 1 | 1 |
| 62 | 7 | 2 | 122 | 27 | 8 | 1 | 1 | 1 | 2 | 1 | 1 | 1 | 2 | 1 | 1 | 1 | 1 | 1 |
| 63 | 7 | 2 | 122 | 26 | 8 | 1 | 1 | 1 | 2 | 1 | 1 | 1 | 2 | 1 | 1 | 1 | 1 | 1 |
| 64 | 7 | 2 | 122 | 24.5 | 8 | 1 | 1 | 1 | 2 | 1 | 1 | 1 | 2 | 1 | 1 | 1 | 1 | 1 |
| 65 | 7 | 2 | 122 | 23 | 8 | 1 | 1 | 1 | 2 | 2 | 1 | 1 | 2 | 1 | 1 | 1 | 1 | 1 |
| 66 | 7 | 2 | 122 | 23 | 8 | 1 | 1 | 1 | 2 | 2 | 1 | 1 | 2 | 1 | 1 | 1 | 1 | 1 |
| 67 | 7 | 2 | 122 | 21 | 8 | 1 | 1 | 1 | 2 | 1 | 1 | 1 | 2 | 1 | 1 | 1 | 1 | 1 |
| 68 | 7 | 2 | 122 | 18 | 8 | 1 | 1 | 1 | 2 | 4 | 1 | 1 | 2 | 1 | 1 | 1 | 1 | 1 |
| 69 | 7 | 2 | 122.5 | 26.5 | 8 | 1 | 1 | 1 | 2 | 1 | 1 | 1 | 2 | 1 | 2 | 1 | 1 | 1 |
| 70 | 7 | 2 | 123 | 28 | 8 | 1 | 1 | 1 | 2 | 4 | 1 | 1 | 2 | 1 | 1 | 1 | 1 | 1 |
| 71 | 7 | 2 | 123 | 24 | 8 | 1 | 1 | 1 | 2 | 2 | 1 | 1 | 2 | 1 | 1 | 1 | 1 | 1 |
| 72 | 7 | 2 | 123 | 22 | 8 | 1 | 1 | 1 | 2 | 4 | 1 | 1 | 2 | 1 | 1 | 1 | 1 | 1 |
| 73 | 7 | 2 | 123 | 21.5 | 3 | 1 | 1 | 1 | 2 | 2 | 1 | 1 | 2 | 1 | 1 | 1 | 1 | 1 |
| 74 | 7 | 2 | 123 | 20 | 8 | 1 | 1 | 1 | 2 | 2 | 1 | 1 | 2 | 1 | 1 | 1 | 1 | 1 |
| 75 | 7 | 2 | 123 | 19 | 8 | 1 | 1 | 1 | 2 | 1 | 1 | 1 | 2 | 1 | 1 | 1 | 1 | 1 |
| 76 | 7 | 2 | 124 | 26 | 8 | 1 | 1 | 1 | 2 | 1 | 1 | 1 | 2 | 1 | 1 | 1 | 1 | 1 |
| 77 | 7 | 2 | 124 | 25 | 8 | 1 | 1 | 1 | 2 | 1 | 1 | 1 | 2 | 1 | 1 | 1 | 1 | 1 |
| 78 | 7 | 2 | 124.8 | 33.4 | 8 | 1 | 1 | 1 | 2 | 1 | 1 | 1 | 2 | 1 | 1 | 1 | 1 | 1 |
| 79 | 7 | 2 | 125 | 27 | 8 | 1 | 1 | 1 | 2 | 4 | 1 | 1 | 2 | 1 | 1 | 1 | 1 | 1 |
| 80 | 7 | 2 | 125 | 20 | 8 | 1 | 1 | 1 | 2 | 4 | 1 | 1 | 2 | 1 | 1 | 1 | 1 | 1 |
| 81 | 7 | 2 | 125.2 | 25.5 | 8 | 1 | 2 | 1 | 3 | 1 | 1 | 1 | 2 | 1 | 1 | 1 | 1 | 1 |
| 82 | 7 | 2 | 125.5 | 27 | 8 | 1 | 1 | 1 | 2 | 1 | 1 | 1 | 2 | 1 | 1 | 1 | 1 | 1 |
| 83 | 7 | 2 | 125.5 | 22.5 | 8 | 1 | 1 | 1 | 2 | 1 | 1 | 1 | 2 | 1 | 1 | 1 | 1 | 1 |
| 84 | 7 | 2 | 127 | 24.5 | 8 | 1 | 2 | 1 | 2 | 2 | 1 | 1 | 2 | 1 | 1 | 1 | 1 | 1 |
| 85 | 7 | 2 | 127 | 23.5 | 8 | 1 | 1 | 1 | 2 | 1 | 1 | 1 | 2 | 1 | 1 | 1 | 1 | 1 |
| 86 | 7 | 2 | 127 | 22 | 8 | 1 | 1 | 1 | 2 | 3 | 1 | 1 | 2 | 1 | 1 | 1 | 1 | 1 |
| 87 | 7 | 2 | 127.2 | 26.5 | 8 | 1 | 1 | 1 | 2 | 1 | 1 | 1 | 2 | 1 | 1 | 1 | 1 | 1 |
| 88 | 7 | 2 | 127.5 | 24 | 8 | 1 | 1 | 1 | 2 | 1 | 1 | 1 | 2 | 1 | 1 | 1 | 1 | 1 |
| 89 | 7 | 2 | 128 | 37 | 8 | 1 | 1 | 1 | 2 | 1 | 1 | 1 | 2 | 1 | 1 | 1 | 1 | 1 |
| 90 | 7 | 2 | 128 | 26.5 | 8 | 1 | 1 | 2 | 2 | 1 | 1 | 1 | 2 | 1 | 1 | 1 | 1 | 1 |
| 91 | 7 | 2 | 129.5 | 26 | 8 | 1 | 1 | 1 | 2 | 1 | 1 | 1 | 2 | 1 | 1 | 1 | 1 | 1 |
| 92 | 7 | 2 | 132 | 26 | 8 | 1 | 1 | 1 | 2 | 2 | 1 | 1 | 2 | 1 | 1 | 1 | 1 | 1 |
| 93 | 7 | 2 | 152 | 25.5 | 8 | 1 | 1 | 1 | 2 | 1 | 1 | 1 | 2 | 1 | 1 | 1 | 1 | 1 |
| 94 | 7 | 1 | 110 | 15 | 8 | 1 | 1 | 1 | 2 | 1 | 1 | 1 | 2 | 1 | 1 | 1 | 1 | 1 |
| 95 | 7 | 1 | 112 | 17 | 8 | 1 | 1 | 1 | 2 | 2 | 1 | 1 | 2 | 1 | 1 | 1 | 1 | 1 |
| 96 | 7 | 1 | 113 | 21 | 8 | 1 | 1 | 1 | 2 | 1 | 1 | 1 | 2 | 1 | 1 | 1 | 1 | 1 |
| 97 | 7 | 1 | 113 | 19 | 8 | 1 | 1 | 1 | 2 | 3 | 1 | 1 | 2 | 1 | 1 | 1 | 1 | 1 |
| 98 | 7 | 1 | 114 | 26.5 | 8 | 1 | 1 | 1 | 2 | 1 | 1 | 1 | 2 | 1 | 1 | 1 | 1 | 1 |
| 99 | 7 | 1 | 114 | 21 | 8 | 1 | 1 | 1 | 2 | 1 | 1 | 1 | 2 | 1 | 1 | 1 | 1 | 1 |
| 100 | 7 | 1 | 114.5 | 17.2 | 8 | 1 | 1 | 1 | 2 | 1 | 1 | 1 | 2 | 1 | 1 | 1 | 1 | 1 |
| 101 | 7 | 1 | 115 | 21 | 8 | 1 | 1 | 1 | 2 | 2 | 1 | 1 | 2 | 1 | 1 | 1 | 1 | 1 |
| 102 | 7 | 1 | 115 | 20 | 8 | 1 | 1 | 1 | 1 | 2 | 1 | 1 | 2 | 1 | 1 | 1 | 1 | 1 |
| 103 | 7 | 1 | 116 | 26 | 8 | 1 | 1 | 1 | 2 | 1 | 1 | 1 | 2 | 1 | 1 | 1 | 1 | 1 |
| 104 | 7 | 1 | 116 | 20.5 | 8 | 1 | 1 | 1 | 2 | 1 | 1 | 1 | 2 | 1 | 1 | 1 | 1 | 1 |
| 105 | 7 | 1 | 116.9 | 21 | 8 | 1 | 1 | 1 | 2 | 3 | 1 | 1 | 2 | 1 | 1 | 1 | 1 | 1 |
| 106 | 7 | 1 | 117 | 22 | 8 | 1 | 1 | 1 | 2 | 1 | 1 | 1 | 2 | 1 | 1 | 1 | 1 | 1 |
| 107 | 7 | 1 | 117 | 22 | 8 | 1 | 1 | 1 | 2 | 1 | 1 | 1 | 2 | 1 | 1 | 1 | 1 | 1 |
| 108 | 7 | 1 | 118 | 23 | 8 | 1 | 1 | 1 | 2 | 3 | 1 | 1 | 2 | 1 | 1 | 1 | 1 | 1 |
| 109 | 7 | 1 | 118 | 21.5 | 8 | 1 | 1 | 1 | 2 | 1 | 1 | 1 | 2 | 1 | 1 | 1 | 1 | 1 |
| 110 | 7 | 1 | 118 | 21 | 8 | 1 | 1 | 1 | 2 | 2 | 1 | 1 | 2 | 1 | 1 | 1 | 1 | 1 |
| 111 | 7 | 1 | 118 | 18 | 8 | 1 | 1 | 1 | 2 | 4 | 1 | 1 | 2 | 1 | 1 | 1 | 1 | 1 |
| 112 | 7 | 1 | 118.5 | 22 | 8 | 1 | 1 | 1 | 2 | 1 | 1 | 1 | 2 | 1 | 1 | 1 | 1 | 1 |
| 113 | 7 | 1 | 119 | 24.5 | 8 | 1 | 1 | 1 | 2 | 1 | 1 | 1 | 2 | 1 | 1 | 1 | 1 | 1 |
| 114 | 7 | 1 | 119 | 22 | 8 | 1 | 1 | 1 | 2 | 2 | 1 | 1 | 2 | 1 | 1 | 1 | 1 | 1 |
| 115 | 7 | 1 | 119 | 22 | 8 | 1 | 1 | 1 | 2 | 2 | 1 | 1 | 2 | 1 | 1 | 1 | 1 | 1 |
| 116 | 7 | 1 | 119 | 22 | 8 | 1 | 1 | 1 | 2 | 1 | 1 | 1 | 2 | 1 | 1 | 1 | 1 | 1 |
| 117 | 7 | 1 | 119 | 20 | 8 | 1 | 1 | 1 | 2 | 1 | 1 | 1 | 2 | 1 | 1 | 1 | 1 | 1 |
| 118 | 7 | 1 | 120 | 23 | 8 | 1 | 1 | 1 | 2 | 1 | 1 | 1 | 2 | 1 | 1 | 1 | 1 | 1 |
| 119 | 7 | 1 | 120 | 23 | 8 | 1 | 1 | 1 | 2 | 1 | 1 | 1 | 2 | 1 | 1 | 1 | 1 | 1 |
| 120 | 7 | 1 | 120 | 23 | 8 | 1 | 1 | 1 | 2 | 1 | 1 | 1 | 2 | 1 | 1 | 1 | 1 | 1 |
| 121 | 7 | 1 | 120 | 22 | 8 | 1 | 1 | 1 | 2 | 2 | 1 | 1 | 2 | 1 | 1 | 1 | 1 | 1 |
| 122 | 7 | 1 | 121 | 31 | 8 | 1 | 1 | 1 | 2 | 2 | 1 | 1 | 2 | 1 | 1 | 1 | 1 | 1 |
| 123 | 7 | 1 | 121 | 25 | 8 | 1 | 1 | 1 | 2 | 4 | 1 | 1 | 2 | 1 | 1 | 1 | 1 | 1 |
| 124 | 7 | 1 | 121 | 22.6 | 8 | 1 | 1 | 1 | 2 | 1 | 1 | 1 | 2 | 1 | 1 | 1 | 1 | 1 |
| 125 | 7 | 1 | 121 | 22.5 | 8 | 1 | 1 | 1 | 2 | 2 | 1 | 1 | 2 | 1 | 1 | 1 | 1 | 1 |
| 126 | 7 | 1 | 121.5 | 24 | 8 | 2 | 1 | 1 | 2 | 2 | 1 | 1 | 2 | 1 | 1 | 1 | 1 | 1 |
| 127 | 7 | 1 | 121.8 | 24.6 | 8 | 1 | 1 | 1 | 2 | 2 | 1 | 1 | 2 | 1 | 1 | 1 | 1 | 1 |
| 128 | 7 | 1 | 122 | 27 | 8 | 1 | 1 | 1 | 2 | 1 | 1 | 1 | 2 | 1 | 1 | 1 | 1 | 1 |
| 129 | 7 | 1 | 122 | 24 | 8 | 1 | 1 | 1 | 2 | 1 | 1 | 1 | 2 | 1 | 1 | 1 | 1 | 1 |
| 130 | 7 | 1 | 122 | 24 | 8 | 1 | 1 | 1 | 2 | 1 | 1 | 1 | 2 | 1 | 1 | 1 | 1 | 1 |
| 131 | 7 | 1 | 122 | 23 | 8 | 1 | 1 | 1 | 2 | 2 | 1 | 1 | 2 | 1 | 1 | 1 | 1 | 1 |
| 132 | 7 | 1 | 122 | 23 | 8 | 1 | 1 | 1 | 2 | 1 | 1 | 1 | 2 | 1 | 1 | 1 | 1 | 1 |
| 133 | 7 | 1 | 122 | 22 | 8 | 1 | 1 | 1 | 2 | 1 | 1 | 1 | 2 | 1 | 1 | 1 | 1 | 1 |
| 134 | 7 | 1 | 122.2 | 25 | 8 | 1 | 1 | 1 | 2 | 1 | 1 | 1 | 1 | 1 | 1 | 1 | 1 | 1 |
| 135 | 7 | 1 | 122.3 | 29.8 | 8 | 1 | 1 | 1 | 2 | 2 | 1 | 1 | 2 | 1 | 1 | 1 | 1 | 1 |
| 136 | 7 | 1 | 122.5 | 27 | 8 | 1 | 1 | 1 | 2 | 2 | 1 | 1 | 2 | 1 | 1 | 1 | 1 | 1 |
| 137 | 7 | 1 | 122.8 | 22 | 8 | 1 | 1 | 1 | 2 | 1 | 1 | 1 | 2 | 1 | 1 | 1 | 1 | 1 |
| 138 | 7 | 1 | 123 | 24.5 | 8 | 1 | 1 | 1 | 2 | 2 | 1 | 1 | 2 | 1 | 1 | 1 | 1 | 1 |
| 139 | 7 | 1 | 123 | 23.5 | 8 | 1 | 1 | 1 | 4 | 3 | 1 | 1 | 2 | 1 | 1 | 1 | 1 | 1 |
| 140 | 7 | 1 | 123 | 22 | 8 | 1 | 1 | 1 | 2 | 1 | 1 | 1 | 2 | 1 | 1 | 1 | 1 | 1 |
| 141 | 7 | 1 | 123.1 | 26.5 | 8 | 1 | 2 | 1 | 2 | 1 | 1 | 1 | 2 | 1 | 1 | 1 | 1 | 1 |
| 142 | 7 | 1 | 123.4 | 25 | 8 | 1 | 1 | 1 | 2 | 1 | 1 | 1 | 2 | 1 | 1 | 1 | 1 | 1 |
| 143 | 7 | 1 | 123.5 | 21.5 | 8 | 1 | 1 | 1 | 2 | 1 | 1 | 1 | 2 | 1 | 1 | 1 | 1 | 1 |
| 144 | 7 | 1 | 123.6 | 27 | 8 | 1 | 1 | 1 | 2 | 1 | 1 | 1 | 2 | 1 | 1 | 1 | 1 | 1 |
| 145 | 7 | 1 | 124 | 25 | 8 | 1 | 1 | 1 | 6 | 3 | 1 | 1 | 2 | 1 | 1 | 1 | 1 | 1 |
| 146 | 7 | 1 | 124 | 24.5 | 8 | 1 | 1 | 1 | 2 | 2 | 1 | 1 | 2 | 1 | 1 | 1 | 1 | 1 |
| 147 | 7 | 1 | 124 | 24 | 8 | 1 | 1 | 1 | 2 | 4 | 1 | 1 | 2 | 1 | 1 | 1 | 1 | 1 |
| 148 | 7 | 1 | 124 | 22 | 8 | 1 | 1 | 1 | 2 | 4 | 1 | 1 | 2 | 1 | 1 | 1 | 1 | 1 |
| 149 | 7 | 1 | 125 | 26.5 | 8 | 1 | 1 | 1 | 2 | 1 | 1 | 1 | 2 | 1 | 1 | 1 | 1 | 1 |
| 150 | 7 | 1 | 125 | 25 | 8 | 1 | 1 | 1 | 2 | 1 | 1 | 1 | 2 | 1 | 1 | 1 | 1 | 1 |
| 151 | 7 | 1 | 125 | 22.5 | 8 | 1 | 1 | 1 | 2 | 4 | 1 | 1 | 1 | 1 | 1 | 1 | 1 | 1 |
| 152 | 7 | 1 | 125.4 | 34 | 8 | 1 | 1 | 1 | 2 | 1 | 1 | 1 | 2 | 1 | 1 | 1 | 1 | 1 |
| 153 | 7 | 1 | 125.5 | 23 | 8 | 1 | 1 | 1 | 2 | 2 | 1 | 1 | 2 | 1 | 1 | 1 | 1 | 1 |
| 154 | 7 | 1 | 126 | 26 | 8 | 1 | 1 | 1 | 2 | 1 | 1 | 1 | 2 | 1 | 1 | 1 | 1 | 1 |
| 155 | 7 | 1 | 126 | 23 | 8 | 1 | 1 | 1 | 2 | 3 | 1 | 1 | 2 | 1 | 1 | 1 | 1 | 1 |
| 156 | 7 | 1 | 126.3 | 24.5 | 8 | 1 | 1 | 1 | 2 | 1 | 1 | 1 | 2 | 1 | 1 | 1 | 1 | 1 |
| 157 | 7 | 1 | 126.7 | 25 | 8 | 1 | 1 | 1 | 2 | 2 | 1 | 1 | 2 | 1 | 1 | 1 | 1 | 1 |
| 158 | 7 | 1 | 127 | 29 | 8 | 1 | 1 | 1 | 2 | 4 | 1 | 1 | 2 | 1 | 1 | 1 | 1 | 1 |
| 159 | 7 | 1 | 127 | 28 | 8 | 1 | 1 | 1 | 2 | 2 | 1 | 1 | 2 | 1 | 1 | 1 | 1 | 1 |
| 160 | 7 | 1 | 127 | 21.5 | 8 | 1 | 1 | 1 | 2 | 1 | 1 | 1 | 2 | 1 | 1 | 1 | 1 | 1 |
| 161 | 7 | 1 | 127.2 | 33.5 | 8 | 1 | 1 | 1 | 2 | 1 | 1 | 1 | 2 | 1 | 1 | 1 | 1 | 1 |
| 162 | 7 | 1 | 127.5 | 27 | 8 | 1 | 1 | 1 | 2 | 3 | 1 | 1 | 2 | 1 | 2 | 1 | 1 | 1 |
| 163 | 7 | 1 | 128 | 24 | 8 | 1 | 1 | 1 | 2 | 1 | 1 | 1 | 2 | 1 | 1 | 1 | 1 | 1 |
| 164 | 7 | 1 | 129 | 28 | 8 | 1 | 1 | 1 | 2 | 1 | 1 | 1 | 2 | 1 | 2 | 1 | 1 | 1 |
| 165 | 7 | 1 | 129 | 23.4 | 4 | 2 | 1 | 1 | 4 | 2 | 1 | 1 | 2 | 1 | 1 | 1 | 1 | 1 |
| 166 | 7 | 1 | 129.5 | 33 | 8 | 1 | 1 | 1 | 2 | 1 | 1 | 1 | 2 | 1 | 1 | 1 | 1 | 1 |
| 167 | 7 | 1 | 129.5 | 31.5 | 8 | 1 | 1 | 1 | 3 | 1 | 1 | 1 | 2 | 1 | 1 | 1 | 1 | 1 |
| 168 | 7 | 1 | 130 | 30 | 8 | 1 | 1 | 1 | 2 | 4 | 1 | 1 | 2 | 1 | 1 | 1 | 1 | 1 |
| 169 | 7 | 1 | 130 | 30 | 8 | 1 | 1 | 1 | 2 | 1 | 1 | 1 | 2 | 1 | 1 | 1 | 1 | 1 |
| 170 | 7 | 1 | 130.6 | 40 | 8 | 1 | 1 | 1 | 2 | 2 | 1 | 1 | 2 | 1 | 1 | 1 | 1 | 1 |
| 171 | 7 | 1 | 131 | 33.5 | 8 | 1 | 1 | 1 | 2 | 1 | 1 | 1 | 2 | 1 | 1 | 1 | 1 | 1 |
| 172 | 7 | 1 | 131 | 28 | 8 | 1 | 1 | 1 | 2 | 1 | 1 | 1 | 2 | 1 | 1 | 1 | 1 | 1 |
| 173 | 7 | 1 | 131 | 28 | 8 | 1 | 1 | 1 | 2 | 1 | 1 | 1 | 2 | 1 | 1 | 1 | 1 | 1 |
| 174 | 7 | 1 | 131.8 | 27.4 | 8 | 1 | 1 | 1 | 2 | 1 | 1 | 1 | 2 | 1 | 1 | 1 | 1 | 1 |
| 175 | 7 | 1 | 132 | 36 | 8 | 1 | 2 | 1 | 2 | 2 | 1 | 1 | 2 | 1 | 1 | 1 | 1 | 1 |
| 176 | 7 | 1 | 132 | 27 | 8 | 1 | 1 | 1 | 2 | 1 | 1 | 1 | 2 | 1 | 1 | 1 | 1 | 1 |
| 177 | 7 | 1 | 133.5 | 27 | 8 | 1 | 1 | 1 | 2 | 2 | 1 | 1 | 2 | 1 | 2 | 1 | 1 | 1 |
| 178 | 7 | 1 | 134 | 33 | 8 | 1 | 1 | 1 | 2 | 4 | 1 | 1 | 2 | 1 | 1 | 1 | 1 | 1 |
| 179 | 7 | 1 | 134 | 32 | 8 | 1 | 1 | 1 | 2 | 3 | 1 | 1 | 2 | 1 | 1 | 1 | 1 | 1 |
| 180 | 8 | 2 | 111 | 20 | 8 | 1 | 1 | 1 | 2 | 2 | 1 | 1 | 2 | 1 | 1 | 1 | 1 | 1 |
| 181 | 8 | 2 | 115.5 | 17 | 8 | 1 | 1 | 1 | 2 | 1 | 1 | 1 | 2 | 1 | 1 | 1 | 1 | 1 |
| 182 | 8 | 2 | 117 | 29 | 8 | 1 | 1 | 1 | 2 | 1 | 1 | 1 | 2 | 1 | 1 | 1 | 1 | 1 |
| 183 | 8 | 2 | 118 | 23 | 8 | 1 | 1 | 1 | 2 | 1 | 1 | 1 | 2 | 1 | 1 | 1 | 1 | 1 |
| 184 | 8 | 2 | 119.2 | 23.5 | 8 | 1 | 1 | 1 | 2 | 4 | 1 | 1 | 2 | 1 | 1 | 1 | 1 | 1 |
| 185 | 8 | 2 | 120 | 30 | 8 | 1 | 1 | 1 | 2 | 2 | 1 | 1 | 2 | 1 | 1 | 1 | 1 | 1 |
| 186 | 8 | 2 | 120 | 30 | 8 | 1 | 1 | 1 | 2 | 1 | 1 | 1 | 2 | 1 | 1 | 1 | 1 | 1 |
| 187 | 8 | 2 | 120 | 21 | 8 | 1 | 1 | 1 | 2 | 2 | 1 | 1 | 2 | 1 | 1 | 1 | 1 | 1 |
| 188 | 8 | 2 | 120.1 | 21.5 | 8 | 1 | 1 | 1 | 2 | 3 | 1 | 1 | 2 | 1 | 1 | 1 | 1 | 1 |
| 189 | 8 | 2 | 120.2 | 22 | 8 | 1 | 1 | 1 | 2 | 1 | 1 | 1 | 2 | 1 | 1 | 1 | 1 | 1 |
| 190 | 8 | 2 | 120.3 | 22.5 | 8 | 1 | 1 | 1 | 2 | 1 | 1 | 1 | 2 | 1 | 1 | 1 | 1 | 1 |
| 191 | 8 | 2 | 121 | 23.1 | 8 | 1 | 1 | 1 | 2 | 2 | 1 | 1 | 2 | 1 | 1 | 1 | 1 | 1 |
| 192 | 8 | 2 | 121 | 23 | 8 | 1 | 1 | 1 | 2 | 1 | 1 | 1 | 2 | 1 | 1 | 1 | 1 | 1 |
| 193 | 8 | 2 | 121.5 | 25.1 | 8 | 1 | 1 | 1 | 2 | 1 | 1 | 1 | 2 | 1 | 1 | 1 | 1 | 1 |
| 194 | 8 | 2 | 121.7 | 21.5 | 8 | 1 | 1 | 1 | 2 | 1 | 1 | 1 | 2 | 1 | 1 | 1 | 1 | 1 |
| 195 | 8 | 2 | 122 | 24 | 8 | 1 | 1 | 1 | 2 | 1 | 1 | 1 | 2 | 1 | 1 | 1 | 1 | 1 |
| 196 | 8 | 2 | 122 | 23.5 | 8 | 1 | 1 | 1 | 2 | 2 | 1 | 1 | 2 | 1 | 1 | 1 | 1 | 1 |
| 197 | 8 | 2 | 122 | 20 | 8 | 1 | 1 | 1 | 2 | 4 | 1 | 1 | 2 | 1 | 1 | 1 | 1 | 1 |
| 198 | 8 | 2 | 122.3 | 22.1 | 8 | 1 | 1 | 1 | 2 | 1 | 1 | 1 | 2 | 1 | 1 | 1 | 1 | 1 |
| 199 | 8 | 2 | 122.3 | 20 | 8 | 1 | 1 | 1 | 2 | 2 | 1 | 1 | 2 | 1 | 1 | 1 | 1 | 1 |
| 200 | 8 | 2 | 122.5 | 20 | 8 | 1 | 1 | 1 | 2 | 1 | 1 | 1 | 2 | 1 | 1 | 1 | 1 | 1 |
| 201 | 8 | 2 | 123 | 32 | 8 | 1 | 1 | 1 | 2 | 2 | 1 | 1 | 2 | 1 | 1 | 1 | 1 | 1 |
| 202 | 8 | 2 | 123 | 26 | 8 | 1 | 1 | 1 | 2 | 1 | 1 | 1 | 2 | 1 | 1 | 1 | 1 | 1 |
| 203 | 8 | 2 | 123 | 25 | 8 | 1 | 1 | 1 | 2 | 1 | 1 | 1 | 2 | 1 | 1 | 1 | 1 | 1 |
| 204 | 8 | 2 | 123.3 | 23 | 8 | 1 | 1 | 1 | 2 | 1 | 1 | 1 | 2 | 1 | 1 | 1 | 1 | 1 |
| 205 | 8 | 2 | 123.3 | 22.5 | 8 | 1 | 1 | 1 | 2 | 3 | 1 | 1 | 2 | 1 | 1 | 1 | 1 | 1 |
| 206 | 8 | 2 | 124 | 27 | 8 | 1 | 1 | 1 | 2 | 2 | 1 | 1 | 2 | 1 | 1 | 1 | 1 | 1 |
| 207 | 8 | 2 | 125 | 25 | 8 | 1 | 1 | 1 | 2 | 1 | 1 | 1 | 2 | 1 | 1 | 1 | 1 | 1 |
| 208 | 8 | 2 | 125 | 24.5 | 8 | 1 | 1 | 1 | 2 | 1 | 1 | 1 | 2 | 1 | 1 | 1 | 1 | 1 |
| 209 | 8 | 2 | 125 | 23 | 8 | 1 | 1 | 1 | 2 | 1 | 1 | 1 | 2 | 1 | 1 | 1 | 1 | 1 |
| 210 | 8 | 2 | 125 | 23 | 8 | 1 | 1 | 1 | 2 | 1 | 1 | 1 | 2 | 1 | 1 | 1 | 1 | 1 |
| 211 | 8 | 2 | 125.3 | 30.5 | 8 | 1 | 1 | 1 | 2 | 1 | 1 | 1 | 2 | 1 | 1 | 1 | 1 | 1 |
| 212 | 8 | 2 | 125.6 | 25.5 | 8 | 1 | 1 | 1 | 3 | 1 | 1 | 1 | 2 | 1 | 1 | 1 | 1 | 1 |
| 213 | 8 | 2 | 125.6 | 24.8 | 8 | 1 | 1 | 1 | 2 | 1 | 1 | 1 | 2 | 1 | 1 | 1 | 1 | 1 |
| 214 | 8 | 2 | 126 | 26 | 8 | 1 | 1 | 1 | 2 | 1 | 1 | 1 | 2 | 1 | 1 | 1 | 1 | 1 |
| 215 | 8 | 2 | 126 | 21 | 8 | 1 | 2 | 1 | 2 | 1 | 1 | 1 | 2 | 1 | 1 | 1 | 1 | 1 |
| 216 | 8 | 2 | 126 | 21 | 8 | 1 | 1 | 1 | 2 | 1 | 1 | 1 | 2 | 1 | 1 | 1 | 1 | 1 |
| 217 | 8 | 2 | 126.3 | 23.5 | 8 | 1 | 1 | 1 | 2 | 3 | 1 | 1 | 2 | 1 | 1 | 1 | 1 | 1 |
| 218 | 8 | 2 | 126.5 | 22 | 8 | 1 | 1 | 1 | 2 | 2 | 1 | 1 | 2 | 1 | 1 | 1 | 1 | 1 |
| 219 | 8 | 2 | 127.7 | 20 | 8 | 1 | 1 | 1 | 2 | 1 | 1 | 1 | 2 | 1 | 1 | 1 | 1 | 1 |
| 220 | 8 | 2 | 128 | 26.5 | 8 | 1 | 1 | 1 | 2 | 1 | 1 | 1 | 2 | 1 | 1 | 1 | 1 | 1 |
| 221 | 8 | 2 | 128 | 24 | 8 | 1 | 1 | 1 | 2 | 4 | 1 | 1 | 2 | 1 | 1 | 1 | 1 | 1 |
| 222 | 8 | 2 | 128.5 | 27 | 8 | 1 | 1 | 1 | 2 | 3 | 1 | 1 | 2 | 1 | 1 | 1 | 1 | 1 |
| 223 | 8 | 2 | 129 | 26 | 8 | 1 | 1 | 1 | 2 | 2 | 1 | 1 | 2 | 1 | 1 | 1 | 1 | 1 |
| 224 | 8 | 2 | 129 | 26 | 8 | 1 | 1 | 1 | 2 | 1 | 1 | 1 | 2 | 1 | 1 | 1 | 1 | 1 |
| 225 | 8 | 2 | 129 | 24 | 8 | 1 | 1 | 1 | 2 | 1 | 1 | 1 | 2 | 1 | 1 | 1 | 1 | 1 |
| 226 | 8 | 2 | 129 | 22.5 | 8 | 1 | 1 | 1 | 2 | 2 | 1 | 1 | 2 | 1 | 1 | 1 | 1 | 1 |
| 227 | 8 | 2 | 129 | 22 | 8 | 1 | 1 | 1 | 2 | 2 | 1 | 1 | 2 | 1 | 1 | 1 | 1 | 1 |
| 228 | 8 | 2 | 129.3 | 27.5 | 8 | 1 | 1 | 1 | 2 | 1 | 1 | 1 | 2 | 1 | 1 | 1 | 1 | 1 |
| 229 | 8 | 2 | 129.5 | 30.1 | 8 | 1 | 1 | 1 | 2 | 1 | 1 | 1 | 2 | 1 | 1 | 1 | 1 | 1 |
| 230 | 8 | 2 | 130 | 30 | 8 | 1 | 1 | 1 | 2 | 1 | 1 | 1 | 2 | 1 | 1 | 1 | 1 | 1 |
| 231 | 8 | 2 | 130 | 26.5 | 8 | 1 | 1 | 1 | 2 | 3 | 1 | 1 | 2 | 1 | 1 | 1 | 1 | 1 |
| 232 | 8 | 2 | 130 | 25 | 8 | 1 | 1 | 1 | 2 | 3 | 1 | 1 | 2 | 1 | 1 | 1 | 1 | 1 |
| 233 | 8 | 2 | 130.5 | 27 | 8 | 1 | 1 | 1 | 2 | 2 | 1 | 1 | 2 | 1 | 1 | 1 | 1 | 1 |
| 234 | 8 | 2 | 131 | 25.5 | 8 | 1 | 1 | 1 | 2 | 1 | 1 | 1 | 2 | 1 | 1 | 1 | 1 | 1 |
| 235 | 8 | 2 | 131.5 | 32 | 8 | 1 | 1 | 1 | 2 | 1 | 1 | 1 | 2 | 1 | 1 | 1 | 1 | 1 |
| 236 | 8 | 2 | 132 | 40 | 8 | 1 | 1 | 1 | 2 | 3 | 1 | 1 | 2 | 1 | 1 | 1 | 1 | 1 |
| 237 | 8 | 2 | 132 | 30 | 8 | 1 | 1 | 1 | 2 | 1 | 1 | 1 | 2 | 1 | 1 | 1 | 1 | 1 |
| 238 | 8 | 2 | 132 | 29.9 | 8 | 1 | 1 | 1 | 2 | 1 | 1 | 1 | 2 | 1 | 1 | 1 | 1 | 1 |
| 239 | 8 | 2 | 132 | 26.5 | 8 | 1 | 1 | 2 | 2 | 1 | 1 | 1 | 2 | 1 | 1 | 1 | 1 | 1 |
| 240 | 8 | 2 | 134.3 | 34.1 | 8 | 1 | 1 | 1 | 2 | 2 | 1 | 1 | 2 | 1 | 1 | 1 | 1 | 1 |
| 241 | 8 | 2 | 134.3 | 31.2 | 8 | 1 | 1 | 1 | 2 | 1 | 1 | 1 | 2 | 1 | 1 | 1 | 1 | 1 |
| 242 | 8 | 2 | 135 | 28 | 8 | 1 | 1 | 1 | 2 | 1 | 1 | 1 | 2 | 1 | 1 | 1 | 1 | 1 |
| 243 | 8 | 2 | 135.5 | 42.5 | 8 | 1 | 1 | 1 | 2 | 1 | 1 | 1 | 2 | 1 | 1 | 1 | 1 | 1 |
| 244 | 8 | 2 | 136 | 41.5 | 8 | 1 | 1 | 1 | 2 | 4 | 1 | 1 | 2 | 1 | 1 | 1 | 1 | 1 |
| 245 | 8 | 2 | 136 | 30 | 8 | 1 | 1 | 1 | 2 | 4 | 1 | 1 | 2 | 1 | 1 | 1 | 1 | 1 |
| 246 | 8 | 2 | 138.7 | 44.1 | 8 | 1 | 1 | 1 | 2 | 1 | 1 | 1 | 2 | 1 | 1 | 1 | 1 | 1 |
| 247 | 8 | 2 | 142.5 | 30.5 | 8 | 1 | 1 | 1 | 2 | 1 | 1 | 1 | 2 | 1 | 1 | 1 | 1 | 1 |
| 248 | 8 | 2 | 148.5 | 36 | 8 | 1 | 1 | 1 | 2 | 1 | 1 | 1 | 2 | 1 | 1 | 1 | 1 | 1 |
| 249 | 8 | 1 | 115 | 26 | 8 | 1 | 1 | 1 | 2 | 2 | 1 | 1 | 2 | 1 | 1 | 1 | 1 | 1 |
| 250 | 8 | 1 | 115 | 20 | 8 | 1 | 1 | 1 | 2 | 1 | 1 | 1 | 2 | 1 | 1 | 1 | 1 | 1 |
| 251 | 8 | 1 | 115 | 18.6 | 8 | 1 | 1 | 1 | 2 | 2 | 1 | 1 | 2 | 1 | 1 | 1 | 1 | 1 |
| 252 | 8 | 1 | 117 | 24 | 8 | 1 | 1 | 1 | 2 | 3 | 1 | 1 | 2 | 1 | 1 | 1 | 1 | 1 |
| 253 | 8 | 1 | 117 | 19.5 | 8 | 1 | 1 | 1 | 2 | 3 | 1 | 1 | 2 | 1 | 1 | 1 | 1 | 1 |
| 254 | 8 | 1 | 117.8 | 23.7 | 8 | 1 | 1 | 1 | 2 | 1 | 1 | 1 | 2 | 1 | 1 | 1 | 1 | 1 |
| 255 | 8 | 1 | 118 | 25 | 8 | 1 | 1 | 1 | 2 | 1 | 1 | 1 | 2 | 1 | 1 | 1 | 1 | 1 |
| 256 | 8 | 1 | 118 | 24.8 | 8 | 1 | 1 | 1 | 2 | 2 | 1 | 1 | 2 | 1 | 1 | 1 | 1 | 1 |
| 257 | 8 | 1 | 120 | 23.5 | 8 | 1 | 1 | 1 | 2 | 3 | 1 | 1 | 2 | 1 | 1 | 1 | 1 | 1 |
| 258 | 8 | 1 | 120 | 22 | 8 | 1 | 1 | 1 | 2 | 1 | 1 | 1 | 2 | 1 | 1 | 1 | 1 | 1 |
| 259 | 8 | 1 | 120 | 22 | 8 | 1 | 1 | 1 | 1 | 4 | 1 | 1 | 2 | 1 | 1 | 1 | 1 | 1 |
| 260 | 8 | 1 | 120 | 21 | 8 | 1 | 1 | 1 | 2 | 2 | 1 | 1 | 2 | 1 | 1 | 1 | 1 | 1 |
| 261 | 8 | 1 | 121 | 26 | 8 | 1 | 1 | 1 | 2 | 1 | 1 | 1 | 2 | 1 | 1 | 1 | 1 | 1 |
| 262 | 8 | 1 | 121 | 25 | 8 | 1 | 1 | 1 | 2 | 1 | 1 | 1 | 2 | 1 | 1 | 1 | 1 | 1 |
| 263 | 8 | 1 | 121 | 23.5 | 8 | 1 | 1 | 1 | 3 | 1 | 1 | 1 | 2 | 1 | 1 | 1 | 1 | 1 |
| 264 | 8 | 1 | 121 | 22 | 8 | 1 | 1 | 1 | 2 | 2 | 1 | 1 | 2 | 1 | 1 | 1 | 1 | 1 |
| 265 | 8 | 1 | 121.1 | 23 | 8 | 1 | 1 | 1 | 2 | 1 | 1 | 1 | 2 | 1 | 1 | 1 | 1 | 1 |
| 266 | 8 | 1 | 121.5 | 23 | 8 | 1 | 1 | 1 | 2 | 1 | 1 | 1 | 2 | 1 | 1 | 1 | 1 | 1 |
| 267 | 8 | 1 | 122 | 24 | 8 | 1 | 1 | 1 | 2 | 1 | 1 | 1 | 2 | 1 | 1 | 1 | 1 | 1 |
| 268 | 8 | 1 | 122 | 23 | 8 | 1 | 1 | 1 | 2 | 1 | 1 | 1 | 2 | 1 | 1 | 1 | 1 | 1 |
| 269 | 8 | 1 | 122.8 | 25 | 8 | 1 | 1 | 1 | 2 | 1 | 1 | 1 | 2 | 1 | 1 | 1 | 1 | 1 |
| 270 | 8 | 1 | 123 | 27.5 | 8 | 1 | 1 | 1 | 2 | 2 | 1 | 1 | 2 | 1 | 1 | 1 | 1 | 1 |
| 271 | 8 | 1 | 123 | 25 | 8 | 1 | 1 | 1 | 2 | 2 | 1 | 1 | 2 | 1 | 1 | 1 | 1 | 1 |
| 272 | 8 | 1 | 123.1 | 24.5 | 8 | 1 | 1 | 1 | 2 | 2 | 1 | 1 | 2 | 1 | 1 | 1 | 1 | 1 |
| 273 | 8 | 1 | 123.5 | 29.5 | 8 | 1 | 1 | 1 | 2 | 1 | 1 | 1 | 2 | 1 | 1 | 1 | 1 | 1 |
| 274 | 8 | 1 | 123.5 | 26 | 8 | 1 | 1 | 1 | 2 | 1 | 1 | 1 | 2 | 1 | 1 | 1 | 1 | 1 |
| 275 | 8 | 1 | 124 | 29.5 | 8 | 1 | 1 | 1 | 2 | 1 | 1 | 1 | 2 | 1 | 1 | 1 | 1 | 1 |
| 276 | 8 | 1 | 124 | 29 | 8 | 1 | 1 | 1 | 2 | 1 | 1 | 1 | 2 | 1 | 1 | 1 | 1 | 1 |
| 277 | 8 | 1 | 124 | 25 | 8 | 1 | 1 | 1 | 2 | 1 | 1 | 1 | 2 | 1 | 1 | 1 | 1 | 1 |
| 278 | 8 | 1 | 124 | 24 | 8 | 1 | 1 | 1 | 2 | 4 | 1 | 1 | 2 | 1 | 1 | 1 | 1 | 1 |
| 279 | 8 | 1 | 124.1 | 26.5 | 8 | 1 | 1 | 1 | 2 | 1 | 1 | 1 | 2 | 1 | 1 | 1 | 1 | 1 |
| 280 | 8 | 1 | 124.3 | 24.9 | 8 | 1 | 1 | 1 | 2 | 1 | 1 | 1 | 2 | 1 | 1 | 1 | 1 | 1 |
| 281 | 8 | 1 | 125 | 26.5 | 8 | 1 | 1 | 1 | 2 | 1 | 1 | 1 | 2 | 1 | 1 | 1 | 1 | 1 |
| 282 | 8 | 1 | 125 | 25 | 8 | 1 | 1 | 1 | 2 | 2 | 1 | 1 | 2 | 1 | 1 | 1 | 1 | 1 |
| 283 | 8 | 1 | 125 | 24 | 8 | 1 | 1 | 1 | 2 | 1 | 1 | 1 | 2 | 1 | 1 | 1 | 1 | 1 |
| 284 | 8 | 1 | 125 | 23 | 8 | 1 | 1 | 1 | 2 | 3 | 1 | 1 | 1 | 1 | 1 | 1 | 1 | 1 |
| 285 | 8 | 1 | 125 | 23 | 8 | 1 | 1 | 1 | 2 | 2 | 1 | 1 | 2 | 1 | 1 | 1 | 1 | 1 |
| 286 | 8 | 1 | 125.5 | 28 | 8 | 1 | 1 | 1 | 2 | 1 | 1 | 1 | 2 | 1 | 1 | 1 | 1 | 1 |
| 287 | 8 | 1 | 125.5 | 25 | 8 | 1 | 1 | 1 | 2 | 1 | 1 | 1 | 2 | 1 | 1 | 1 | 1 | 1 |
| 288 | 8 | 1 | 125.5 | 24.5 | 8 | 1 | 1 | 1 | 2 | 2 | 1 | 1 | 2 | 1 | 1 | 1 | 1 | 1 |
| 289 | 8 | 1 | 125.5 | 23.5 | 8 | 1 | 1 | 1 | 2 | 4 | 1 | 1 | 2 | 1 | 1 | 1 | 1 | 1 |
| 290 | 8 | 1 | 125.7 | 24.7 | 8 | 1 | 1 | 1 | 2 | 1 | 1 | 1 | 2 | 1 | 1 | 1 | 1 | 1 |
| 291 | 8 | 1 | 125.8 | 28 | 8 | 1 | 1 | 1 | 2 | 2 | 1 | 1 | 2 | 1 | 1 | 1 | 1 | 1 |
| 292 | 8 | 1 | 126 | 26 | 8 | 1 | 1 | 1 | 2 | 1 | 1 | 1 | 2 | 1 | 1 | 1 | 1 | 1 |
| 293 | 8 | 1 | 126 | 23 | 8 | 1 | 1 | 1 | 2 | 1 | 1 | 1 | 2 | 1 | 1 | 1 | 1 | 1 |
| 294 | 8 | 1 | 126.5 | 25.5 | 8 | 1 | 1 | 1 | 2 | 1 | 1 | 1 | 2 | 1 | 1 | 1 | 1 | 1 |
| 295 | 8 | 1 | 127 | 25.5 | 8 | 1 | 1 | 1 | 2 | 2 | 1 | 1 | 2 | 1 | 1 | 1 | 1 | 1 |
| 296 | 8 | 1 | 127 | 25 | 8 | 1 | 1 | 1 | 2 | 1 | 1 | 1 | 2 | 1 | 1 | 1 | 1 | 1 |
| 297 | 8 | 1 | 127 | 24 | 8 | 1 | 1 | 1 | 2 | 4 | 1 | 1 | 2 | 1 | 1 | 1 | 1 | 1 |
| 298 | 8 | 1 | 127 | 23 | 8 | 1 | 1 | 1 | 2 | 3 | 1 | 1 | 2 | 1 | 1 | 1 | 1 | 1 |
| 299 | 8 | 1 | 127 | 19.5 | 8 | 1 | 1 | 1 | 2 | 2 | 1 | 1 | 2 | 1 | 1 | 1 | 1 | 1 |
| 300 | 8 | 1 | 127.1 | 27 | 8 | 1 | 1 | 1 | 2 | 3 | 1 | 1 | 2 | 1 | 2 | 1 | 1 | 1 |
| 301 | 8 | 1 | 127.1 | 27 | 8 | 1 | 1 | 1 | 2 | 2 | 1 | 1 | 2 | 1 | 1 | 1 | 1 | 1 |
| 302 | 8 | 1 | 127.5 | 29.5 | 8 | 1 | 1 | 1 | 2 | 1 | 1 | 1 | 2 | 1 | 1 | 1 | 1 | 1 |
| 303 | 8 | 1 | 127.6 | 31.5 | 8 | 1 | 1 | 1 | 2 | 1 | 1 | 1 | 2 | 1 | 1 | 1 | 1 | 1 |
| 304 | 8 | 1 | 128 | 29 | 8 | 1 | 2 | 1 | 2 | 4 | 1 | 1 | 2 | 1 | 1 | 1 | 1 | 1 |
| 305 | 8 | 1 | 128 | 27 | 8 | 1 | 2 | 1 | 2 | 1 | 1 | 1 | 2 | 1 | 1 | 1 | 1 | 1 |
| 306 | 8 | 1 | 128 | 27 | 8 | 1 | 1 | 1 | 2 | 1 | 1 | 1 | 2 | 1 | 1 | 1 | 1 | 1 |
| 307 | 8 | 1 | 128 | 22.5 | 8 | 1 | 1 | 1 | 2 | 3 | 1 | 1 | 2 | 1 | 1 | 1 | 1 | 1 |
| 308 | 8 | 1 | 128.4 | 28.1 | 8 | 1 | 1 | 1 | 2 | 3 | 1 | 1 | 2 | 1 | 1 | 1 | 1 | 1 |
| 309 | 8 | 1 | 128.5 | 28 | 8 | 1 | 1 | 1 | 2 | 1 | 1 | 1 | 2 | 1 | 1 | 1 | 1 | 1 |
| 310 | 8 | 1 | 128.5 | 27.5 | 8 | 1 | 1 | 1 | 2 | 2 | 1 | 1 | 2 | 1 | 1 | 1 | 1 | 1 |
| 311 | 8 | 1 | 129 | 28.8 | 8 | 1 | 1 | 1 | 2 | 2 | 1 | 1 | 2 | 1 | 1 | 1 | 1 | 1 |
| 312 | 8 | 1 | 129 | 24 | 8 | 1 | 1 | 1 | 2 | 2 | 1 | 1 | 2 | 1 | 2 | 1 | 1 | 1 |
| 313 | 8 | 1 | 129 | 24 | 8 | 1 | 1 | 1 | 2 | 1 | 1 | 1 | 2 | 1 | 1 | 1 | 1 | 1 |
| 314 | 8 | 1 | 129.4 | 28.5 | 8 | 1 | 1 | 1 | 2 | 2 | 1 | 1 | 2 | 1 | 1 | 1 | 1 | 1 |
| 315 | 8 | 1 | 129.4 | 27 | 8 | 1 | 1 | 1 | 2 | 1 | 1 | 1 | 2 | 1 | 1 | 1 | 1 | 1 |
| 316 | 8 | 1 | 130 | 31 | 8 | 1 | 1 | 1 | 2 | 1 | 1 | 1 | 2 | 1 | 1 | 1 | 1 | 1 |
| 317 | 8 | 1 | 130 | 30 | 8 | 1 | 1 | 1 | 2 | 1 | 1 | 1 | 2 | 1 | 1 | 1 | 1 | 1 |
| 318 | 8 | 1 | 130 | 27 | 8 | 1 | 1 | 1 | 2 | 2 | 1 | 1 | 2 | 1 | 1 | 1 | 1 | 1 |
| 319 | 8 | 1 | 130.3 | 28.7 | 8 | 1 | 1 | 1 | 2 | 2 | 1 | 1 | 2 | 1 | 1 | 1 | 1 | 1 |
| 320 | 8 | 1 | 130.5 | 30 | 8 | 1 | 1 | 1 | 2 | 1 | 1 | 1 | 2 | 1 | 1 | 1 | 1 | 1 |
| 321 | 8 | 1 | 131 | 28 | 8 | 1 | 1 | 1 | 2 | 4 | 1 | 1 | 2 | 1 | 1 | 1 | 1 | 1 |
| 322 | 8 | 1 | 131 | 27.8 | 8 | 1 | 1 | 1 | 2 | 1 | 1 | 1 | 2 | 1 | 1 | 1 | 1 | 1 |
| 323 | 8 | 1 | 131 | 25 | 8 | 1 | 1 | 1 | 2 | 3 | 1 | 1 | 2 | 1 | 1 | 1 | 1 | 1 |
| 324 | 8 | 1 | 131.5 | 29 | 8 | 1 | 1 | 1 | 2 | 1 | 2 | 1 | 1 | 1 | 1 | 1 | 1 | 1 |
| 325 | 8 | 1 | 131.5 | 28.5 | 8 | 2 | 1 | 1 | 2 | 1 | 1 | 1 | 2 | 1 | 1 | 1 | 1 | 1 |
| 326 | 8 | 1 | 131.9 | 30.1 | 8 | 1 | 1 | 1 | 2 | 1 | 1 | 1 | 2 | 1 | 1 | 1 | 1 | 1 |
| 327 | 8 | 1 | 132 | 31 | 8 | 1 | 1 | 1 | 2 | 1 | 1 | 1 | 2 | 1 | 1 | 1 | 1 | 1 |
| 328 | 8 | 1 | 133 | 37 | 8 | 1 | 1 | 1 | 2 | 2 | 1 | 1 | 2 | 1 | 1 | 1 | 1 | 1 |
| 329 | 8 | 1 | 133.1 | 32.4 | 8 | 1 | 1 | 1 | 2 | 1 | 1 | 1 | 2 | 1 | 1 | 1 | 1 | 1 |
| 330 | 8 | 1 | 134 | 41 | 8 | 1 | 1 | 1 | 2 | 1 | 1 | 1 | 2 | 1 | 1 | 1 | 1 | 1 |
| 331 | 8 | 1 | 134 | 27.5 | 8 | 1 | 1 | 1 | 2 | 3 | 1 | 1 | 2 | 1 | 1 | 1 | 1 | 1 |
| 332 | 8 | 1 | 134 | 27 | 8 | 1 | 1 | 1 | 2 | 3 | 1 | 1 | 2 | 1 | 1 | 1 | 1 | 1 |
| 333 | 8 | 1 | 135 | 42 | 8 | 1 | 2 | 1 | 2 | 3 | 1 | 1 | 2 | 1 | 2 | 1 | 1 | 1 |
| 334 | 8 | 1 | 135 | 38 | 8 | 1 | 2 | 1 | 2 | 1 | 1 | 1 | 2 | 1 | 1 | 1 | 1 | 1 |
| 335 | 8 | 1 | 135 | 32 | 8 | 1 | 1 | 1 | 2 | 2 | 1 | 1 | 2 | 1 | 1 | 1 | 1 | 1 |
| 336 | 8 | 1 | 135 | 28.5 | 8 | 1 | 1 | 1 | 2 | 2 | 1 | 1 | 2 | 1 | 1 | 1 | 1 | 1 |
| 337 | 8 | 1 | 135 | 20 | 8 | 1 | 1 | 1 | 2 | 2 | 1 | 1 | 2 | 1 | 1 | 1 | 1 | 1 |
| 338 | 8 | 1 | 136 | 36.5 | 8 | 1 | 1 | 1 | 2 | 3 | 1 | 1 | 2 | 1 | 1 | 1 | 1 | 1 |
| 339 | 8 | 1 | 136 | 30 | 8 | 1 | 1 | 1 | 2 | 1 | 2 | 1 | 2 | 1 | 1 | 1 | 1 | 1 |
| 340 | 8 | 1 | 137.1 | 44.5 | 8 | 1 | 1 | 1 | 2 | 2 | 1 | 1 | 2 | 1 | 1 | 1 | 1 | 1 |
| 341 | 8 | 1 | 138.6 | 37.6 | 8 | 1 | 1 | 1 | 2 | 1 | 1 | 1 | 2 | 1 | 1 | 1 | 1 | 1 |
| 342 | 8 | 1 | 140 | 38 | 8 | 1 | 1 | 1 | 2 | 2 | 1 | 1 | 2 | 1 | 1 | 1 | 1 | 1 |
| 343 | 8 | 1 | 142.5 | 45.6 | 8 | 1 | 1 | 1 | 2 | 1 | 1 | 1 | 2 | 1 | 1 | 1 | 1 | 1 |
| 344 | 8 | 1 | 143 | 25 | 8 | 1 | 1 | 1 | 2 | 2 | 1 | 1 | 2 | 1 | 1 | 1 | 1 | 1 |
| 345 | 8 | 1 | 150 | 20 | 8 | 1 | 1 | 1 | 2 | 2 | 1 | 1 | 2 | 1 | 1 | 1 | 1 | 1 |
| 346 | 9 | 2 | 115 | 27 | 8 | 1 | 1 | 1 | 6 | 1 | 1 | 1 | 2 | 1 | 1 | 1 | 1 | 1 |
| 347 | 9 | 2 | 119 | 20 | 8 | 1 | 1 | 1 | 2 | 1 | 1 | 1 | 1 | 1 | 1 | 1 | 1 | 1 |
| 348 | 9 | 2 | 119 | 18 | 8 | 1 | 1 | 1 | 2 | 1 | 1 | 1 | 2 | 1 | 1 | 1 | 1 | 1 |
| 349 | 9 | 2 | 120.5 | 19 | 8 | 1 | 1 | 1 | 2 | 1 | 1 | 1 | 2 | 1 | 1 | 1 | 1 | 1 |
| 350 | 9 | 2 | 122 | 32 | 8 | 1 | 1 | 1 | 2 | 2 | 1 | 1 | 2 | 1 | 1 | 1 | 1 | 1 |
| 351 | 9 | 2 | 122 | 23 | 8 | 1 | 1 | 1 | 2 | 1 | 1 | 1 | 2 | 1 | 1 | 1 | 1 | 1 |
| 352 | 9 | 2 | 122.5 | 23 | 8 | 1 | 1 | 1 | 2 | 2 | 1 | 1 | 2 | 1 | 1 | 1 | 1 | 1 |
| 353 | 9 | 2 | 125 | 28 | 8 | 1 | 1 | 1 | 2 | 3 | 1 | 1 | 2 | 1 | 1 | 1 | 1 | 1 |
| 354 | 9 | 2 | 125 | 21.5 | 8 | 1 | 1 | 1 | 2 | 4 | 1 | 1 | 2 | 1 | 1 | 1 | 1 | 1 |
| 355 | 9 | 2 | 125.1 | 27 | 8 | 1 | 1 | 1 | 3 | 2 | 1 | 1 | 2 | 1 | 1 | 1 | 1 | 1 |
| 356 | 9 | 2 | 126 | 20.5 | 8 | 1 | 1 | 1 | 2 | 2 | 1 | 1 | 2 | 1 | 1 | 1 | 1 | 1 |
| 357 | 9 | 2 | 127 | 23 | 8 | 1 | 1 | 1 | 2 | 1 | 1 | 1 | 2 | 1 | 1 | 1 | 1 | 1 |
| 358 | 9 | 2 | 127.5 | 28 | 8 | 1 | 1 | 1 | 2 | 2 | 1 | 1 | 2 | 1 | 1 | 1 | 1 | 1 |
| 359 | 9 | 2 | 127.5 | 25 | 8 | 1 | 1 | 1 | 2 | 1 | 1 | 1 | 2 | 1 | 1 | 1 | 1 | 1 |
| 360 | 9 | 2 | 127.7 | 31.2 | 8 | 1 | 1 | 1 | 2 | 1 | 1 | 1 | 2 | 1 | 1 | 1 | 1 | 1 |
| 361 | 9 | 2 | 128 | 20.5 | 8 | 1 | 1 | 1 | 2 | 1 | 1 | 1 | 2 | 1 | 1 | 1 | 1 | 1 |
| 362 | 9 | 2 | 128.1 | 25 | 8 | 1 | 1 | 1 | 2 | 1 | 1 | 1 | 2 | 1 | 1 | 1 | 1 | 1 |
| 363 | 9 | 2 | 129 | 32 | 8 | 1 | 1 | 1 | 2 | 3 | 1 | 1 | 2 | 1 | 1 | 1 | 1 | 1 |
| 364 | 9 | 2 | 129 | 28 | 8 | 1 | 1 | 1 | 2 | 1 | 1 | 1 | 2 | 1 | 1 | 1 | 1 | 1 |
| 365 | 9 | 2 | 129 | 26 | 8 | 1 | 1 | 1 | 2 | 1 | 1 | 1 | 2 | 1 | 1 | 1 | 1 | 1 |
| 366 | 9 | 2 | 130 | 22 | 8 | 1 | 1 | 1 | 2 | 2 | 1 | 1 | 2 | 1 | 1 | 1 | 1 | 1 |
| 367 | 9 | 2 | 130 | 21 | 8 | 1 | 1 | 1 | 2 | 1 | 1 | 1 | 2 | 1 | 1 | 1 | 1 | 1 |
| 368 | 9 | 2 | 130.2 | 27 | 8 | 1 | 1 | 1 | 2 | 1 | 1 | 1 | 2 | 1 | 1 | 1 | 1 | 1 |
| 369 | 9 | 2 | 130.5 | 26.5 | 8 | 1 | 1 | 1 | 2 | 3 | 1 | 1 | 2 | 1 | 1 | 1 | 1 | 1 |
| 370 | 9 | 2 | 131 | 32 | 8 | 1 | 1 | 1 | 2 | 2 | 1 | 1 | 2 | 1 | 1 | 1 | 1 | 1 |
| 371 | 9 | 2 | 131 | 28 | 8 | 1 | 1 | 1 | 3 | 1 | 1 | 1 | 2 | 1 | 1 | 1 | 1 | 1 |
| 372 | 9 | 2 | 131 | 26 | 8 | 1 | 1 | 1 | 2 | 1 | 1 | 1 | 2 | 1 | 1 | 1 | 1 | 1 |
| 373 | 9 | 2 | 131 | 26 | 8 | 2 | 1 | 1 | 2 | 1 | 1 | 1 | 2 | 1 | 1 | 1 | 1 | 1 |
| 374 | 9 | 2 | 131 | 24.5 | 8 | 1 | 1 | 1 | 2 | 2 | 1 | 1 | 2 | 1 | 1 | 1 | 1 | 1 |
| 375 | 9 | 2 | 131.2 | 25.5 | 8 | 1 | 1 | 1 | 2 | 1 | 1 | 1 | 2 | 1 | 1 | 1 | 1 | 1 |
| 376 | 9 | 2 | 131.4 | 25.5 | 8 | 1 | 1 | 1 | 2 | 1 | 1 | 1 | 2 | 1 | 1 | 1 | 1 | 1 |
| 377 | 9 | 2 | 132 | 29.5 | 8 | 1 | 1 | 1 | 2 | 1 | 1 | 1 | 2 | 1 | 1 | 1 | 1 | 1 |
| 378 | 9 | 2 | 132.1 | 27.8 | 8 | 1 | 1 | 1 | 3 | 4 | 1 | 1 | 1 | 1 | 1 | 1 | 1 | 1 |
| 379 | 9 | 2 | 132.5 | 28 | 8 | 1 | 1 | 1 | 2 | 1 | 1 | 1 | 2 | 1 | 1 | 1 | 1 | 1 |
| 380 | 9 | 2 | 133 | 28 | 8 | 1 | 1 | 1 | 2 | 2 | 1 | 1 | 2 | 1 | 1 | 1 | 1 | 1 |
| 381 | 9 | 2 | 133 | 26 | 8 | 1 | 1 | 1 | 2 | 2 | 1 | 1 | 2 | 1 | 1 | 1 | 1 | 1 |
| 382 | 9 | 2 | 133 | 24 | 8 | 1 | 1 | 1 | 2 | 1 | 1 | 1 | 2 | 1 | 1 | 1 | 1 | 1 |
| 383 | 9 | 2 | 133.5 | 11.5 | 8 | 1 | 1 | 1 | 2 | 1 | 1 | 1 | 2 | 1 | 1 | 1 | 1 | 1 |
| 384 | 9 | 2 | 134 | 33 | 8 | 1 | 1 | 1 | 2 | 1 | 1 | 1 | 2 | 1 | 1 | 1 | 1 | 1 |
| 385 | 9 | 2 | 134 | 32 | 8 | 1 | 1 | 1 | 2 | 2 | 1 | 1 | 2 | 1 | 1 | 1 | 1 | 1 |
| 386 | 9 | 2 | 134 | 29.5 | 8 | 1 | 1 | 1 | 2 | 1 | 1 | 1 | 2 | 1 | 1 | 1 | 1 | 1 |
| 387 | 9 | 2 | 134 | 26.5 | 8 | 1 | 1 | 1 | 2 | 2 | 1 | 1 | 2 | 1 | 1 | 1 | 1 | 1 |
| 388 | 9 | 2 | 134 | 26 | 8 | 1 | 1 | 1 | 2 | 1 | 1 | 1 | 2 | 1 | 1 | 1 | 1 | 1 |
| 389 | 9 | 2 | 134 | 23 | 8 | 1 | 1 | 1 | 2 | 4 | 1 | 1 | 2 | 1 | 1 | 1 | 1 | 1 |
| 390 | 9 | 2 | 134.5 | 28 | 8 | 1 | 1 | 1 | 2 | 2 | 1 | 1 | 2 | 1 | 1 | 1 | 1 | 1 |
| 391 | 9 | 2 | 135 | 46 | 8 | 1 | 1 | 1 | 2 | 2 | 1 | 1 | 2 | 1 | 1 | 1 | 1 | 1 |
| 392 | 9 | 2 | 135 | 32 | 8 | 1 | 1 | 1 | 2 | 2 | 1 | 1 | 2 | 1 | 1 | 1 | 1 | 1 |
| 393 | 9 | 2 | 135 | 28.7 | 8 | 1 | 1 | 1 | 2 | 1 | 1 | 1 | 2 | 1 | 1 | 1 | 1 | 1 |
| 394 | 9 | 2 | 136 | 34 | 8 | 1 | 1 | 1 | 2 | 1 | 1 | 1 | 2 | 1 | 1 | 1 | 1 | 1 |
| 395 | 9 | 2 | 136 | 33 | 8 | 2 | 1 | 1 | 2 | 1 | 1 | 1 | 2 | 1 | 1 | 1 | 1 | 1 |
| 396 | 9 | 2 | 136 | 30 | 8 | 1 | 1 | 1 | 2 | 1 | 1 | 1 | 2 | 1 | 1 | 1 | 1 | 1 |
| 397 | 9 | 2 | 136 | 29 | 8 | 1 | 1 | 1 | 2 | 2 | 1 | 1 | 2 | 1 | 1 | 1 | 1 | 1 |
| 398 | 9 | 2 | 136 | 28 | 8 | 1 | 1 | 1 | 2 | 2 | 1 | 1 | 1 | 1 | 1 | 1 | 1 | 1 |
| 399 | 9 | 2 | 137 | 33 | 8 | 1 | 1 | 1 | 2 | 2 | 1 | 1 | 2 | 1 | 1 | 1 | 1 | 1 |
| 400 | 9 | 2 | 137 | 32.5 | 8 | 1 | 1 | 1 | 2 | 2 | 1 | 1 | 2 | 1 | 1 | 1 | 1 | 1 |
| 401 | 9 | 2 | 137.1 | 31.5 | 8 | 1 | 1 | 1 | 2 | 3 | 1 | 1 | 2 | 1 | 1 | 1 | 1 | 1 |
| 402 | 9 | 2 | 137.4 | 32 | 8 | 1 | 1 | 1 | 2 | 2 | 1 | 1 | 2 | 1 | 1 | 1 | 1 | 1 |
| 403 | 9 | 2 | 138 | 42 | 8 | 1 | 1 | 1 | 2 | 1 | 1 | 1 | 2 | 1 | 1 | 1 | 1 | 1 |
| 404 | 9 | 2 | 138 | 31.5 | 8 | 1 | 1 | 1 | 2 | 2 | 1 | 1 | 2 | 1 | 1 | 1 | 1 | 1 |
| 405 | 9 | 2 | 138.1 | 31 | 8 | 1 | 1 | 1 | 2 | 2 | 1 | 1 | 2 | 1 | 1 | 1 | 1 | 1 |
| 406 | 9 | 2 | 138.2 | 29.5 | 8 | 1 | 1 | 1 | 2 | 1 | 1 | 1 | 2 | 1 | 1 | 1 | 1 | 1 |
| 407 | 9 | 2 | 138.5 | 32.5 | 8 | 1 | 1 | 1 | 2 | 1 | 1 | 1 | 2 | 1 | 1 | 1 | 1 | 1 |
| 408 | 9 | 2 | 139 | 32 | 8 | 1 | 1 | 1 | 2 | 1 | 1 | 1 | 2 | 1 | 1 | 1 | 1 | 1 |
| 409 | 9 | 2 | 139.4 | 27 | 8 | 1 | 1 | 2 | 2 | 1 | 1 | 1 | 2 | 1 | 1 | 1 | 1 | 1 |
| 410 | 9 | 2 | 140 | 37 | 8 | 1 | 1 | 1 | 2 | 2 | 1 | 1 | 2 | 1 | 1 | 1 | 1 | 1 |
| 411 | 9 | 2 | 140 | 29 | 8 | 1 | 1 | 1 | 2 | 1 | 1 | 1 | 2 | 1 | 1 | 1 | 1 | 1 |
| 412 | 9 | 2 | 140 | 25.5 | 8 | 1 | 1 | 1 | 2 | 1 | 1 | 1 | 2 | 1 | 1 | 1 | 1 | 1 |
| 413 | 9 | 2 | 140.5 | 57 | 8 | 1 | 1 | 1 | 2 | 1 | 1 | 1 | 2 | 1 | 1 | 1 | 1 | 1 |
| 414 | 9 | 2 | 141 | 52 | 8 | 1 | 1 | 1 | 2 | 2 | 1 | 1 | 2 | 1 | 1 | 1 | 1 | 1 |
| 415 | 9 | 2 | 141.5 | 52 | 8 | 1 | 1 | 1 | 2 | 3 | 1 | 1 | 2 | 1 | 1 | 1 | 1 | 1 |
| 416 | 9 | 2 | 143.5 | 33 | 8 | 1 | 1 | 1 | 2 | 3 | 3 | 1 | 2 | 1 | 1 | 1 | 1 | 1 |
| 417 | 9 | 2 | 143.7 | 37 | 8 | 1 | 1 | 1 | 2 | 1 | 2 | 1 | 2 | 1 | 1 | 1 | 1 | 1 |
| 418 | 9 | 2 | 149.5 | 42 | 8 | 1 | 1 | 1 | 2 | 3 | 1 | 1 | 2 | 1 | 1 | 1 | 1 | 1 |
| 419 | 9 | 1 | 120 | 24 | 8 | 1 | 1 | 1 | 2 | 4 | 1 | 1 | 2 | 1 | 1 | 1 | 1 | 1 |
| 420 | 9 | 1 | 120.8 | 22.7 | 8 | 1 | 1 | 1 | 2 | 1 | 1 | 1 | 2 | 1 | 1 | 1 | 1 | 1 |
| 421 | 9 | 1 | 122 | 30 | 8 | 1 | 1 | 1 | 2 | 4 | 1 | 1 | 2 | 1 | 1 | 1 | 1 | 1 |
| 422 | 9 | 1 | 122 | 21 | 8 | 1 | 1 | 1 | 2 | 2 | 1 | 1 | 2 | 1 | 1 | 1 | 1 | 1 |
| 423 | 9 | 1 | 125.7 | 23 | 8 | 1 | 1 | 1 | 2 | 1 | 1 | 1 | 2 | 1 | 1 | 1 | 1 | 1 |
| 424 | 9 | 1 | 126 | 26 | 8 | 1 | 1 | 1 | 2 | 1 | 1 | 1 | 2 | 1 | 1 | 1 | 1 | 1 |
| 425 | 9 | 1 | 126 | 24 | 8 | 1 | 1 | 1 | 2 | 2 | 1 | 1 | 2 | 1 | 1 | 1 | 1 | 1 |
| 426 | 9 | 1 | 126 | 24 | 8 | 1 | 1 | 1 | 2 | 1 | 1 | 1 | 2 | 1 | 1 | 1 | 1 | 1 |
| 427 | 9 | 1 | 126 | 23 | 8 | 1 | 1 | 1 | 2 | 1 | 1 | 1 | 1 | 1 | 1 | 1 | 1 | 1 |
| 428 | 9 | 1 | 126.1 | 26 | 8 | 1 | 1 | 1 | 2 | 3 | 1 | 1 | 2 | 1 | 1 | 1 | 1 | 1 |
| 429 | 9 | 1 | 128 | 31 | 8 | 1 | 1 | 1 | 2 | 2 | 1 | 1 | 2 | 1 | 1 | 1 | 1 | 1 |
| 430 | 9 | 1 | 128 | 28 | 8 | 1 | 1 | 1 | 2 | 2 | 1 | 1 | 2 | 1 | 1 | 1 | 1 | 1 |
| 431 | 9 | 1 | 128 | 23.4 | 8 | 1 | 1 | 1 | 2 | 1 | 1 | 1 | 1 | 1 | 1 | 1 | 1 | 1 |
| 432 | 9 | 1 | 128 | 22 | 8 | 1 | 1 | 1 | 2 | 2 | 1 | 1 | 2 | 1 | 1 | 1 | 1 | 1 |
| 433 | 9 | 1 | 128.5 | 28 | 8 | 1 | 1 | 1 | 2 | 3 | 1 | 1 | 2 | 1 | 1 | 1 | 1 | 1 |
| 434 | 9 | 1 | 128.7 | 31 | 8 | 1 | 1 | 1 | 2 | 3 | 1 | 1 | 2 | 1 | 1 | 1 | 1 | 1 |
| 435 | 9 | 1 | 129 | 27 | 8 | 1 | 2 | 1 | 2 | 3 | 1 | 1 | 2 | 1 | 1 | 1 | 1 | 1 |
| 436 | 9 | 1 | 129.1 | 25 | 8 | 1 | 1 | 1 | 2 | 3 | 1 | 1 | 2 | 1 | 1 | 1 | 1 | 1 |
| 437 | 9 | 1 | 129.7 | 27 | 8 | 1 | 1 | 1 | 2 | 1 | 1 | 1 | 1 | 1 | 1 | 1 | 1 | 1 |
| 438 | 9 | 1 | 129.8 | 41.8 | 8 | 1 | 1 | 1 | 3 | 1 | 1 | 1 | 2 | 1 | 1 | 1 | 1 | 1 |
| 439 | 9 | 1 | 130 | 34 | 8 | 1 | 1 | 1 | 2 | 4 | 1 | 1 | 2 | 1 | 1 | 1 | 1 | 1 |
| 440 | 9 | 1 | 130 | 29 | 8 | 1 | 1 | 1 | 2 | 1 | 1 | 1 | 2 | 1 | 1 | 1 | 1 | 1 |
| 441 | 9 | 1 | 130 | 27 | 8 | 1 | 1 | 1 | 3 | 1 | 1 | 1 | 2 | 1 | 1 | 1 | 1 | 1 |
| 442 | 9 | 1 | 130 | 22 | 8 | 1 | 1 | 1 | 2 | 1 | 1 | 1 | 2 | 1 | 1 | 1 | 1 | 1 |
| 443 | 9 | 1 | 131 | 40 | 8 | 1 | 1 | 1 | 2 | 1 | 1 | 1 | 2 | 1 | 1 | 1 | 1 | 1 |
| 444 | 9 | 1 | 131 | 28 | 8 | 1 | 1 | 1 | 2 | 1 | 1 | 1 | 2 | 1 | 1 | 1 | 1 | 1 |
| 445 | 9 | 1 | 131.1 | 24 | 8 | 1 | 1 | 1 | 2 | 3 | 1 | 1 | 2 | 1 | 1 | 1 | 1 | 1 |
| 446 | 9 | 1 | 131.4 | 23 | 8 | 1 | 1 | 1 | 2 | 1 | 1 | 1 | 2 | 1 | 1 | 1 | 1 | 1 |
| 447 | 9 | 1 | 132 | 42 | 8 | 1 | 1 | 1 | 2 | 2 | 1 | 1 | 2 | 1 | 1 | 1 | 1 | 1 |
| 448 | 9 | 1 | 132 | 35 | 8 | 1 | 1 | 1 | 2 | 2 | 1 | 1 | 2 | 1 | 1 | 1 | 1 | 1 |
| 449 | 9 | 1 | 132 | 32 | 8 | 1 | 1 | 1 | 2 | 1 | 1 | 1 | 2 | 1 | 1 | 1 | 1 | 1 |
| 450 | 9 | 1 | 132 | 29 | 8 | 1 | 1 | 1 | 2 | 1 | 1 | 1 | 2 | 1 | 1 | 1 | 1 | 1 |
| 451 | 9 | 1 | 132 | 28.5 | 8 | 1 | 1 | 1 | 2 | 2 | 1 | 1 | 2 | 1 | 1 | 1 | 1 | 1 |
| 452 | 9 | 1 | 132 | 21 | 8 | 1 | 1 | 1 | 2 | 4 | 1 | 1 | 2 | 1 | 1 | 1 | 1 | 1 |
| 453 | 9 | 1 | 132.5 | 29.5 | 8 | 1 | 1 | 1 | 2 | 1 | 1 | 1 | 2 | 1 | 1 | 1 | 1 | 1 |
| 454 | 9 | 1 | 132.6 | 27.5 | 8 | 1 | 1 | 1 | 2 | 1 | 1 | 1 | 2 | 1 | 1 | 1 | 1 | 1 |
| 455 | 9 | 1 | 133 | 37 | 8 | 1 | 1 | 1 | 2 | 2 | 1 | 1 | 2 | 1 | 1 | 1 | 1 | 1 |
| 456 | 9 | 1 | 133 | 34 | 8 | 1 | 1 | 1 | 2 | 1 | 1 | 1 | 2 | 1 | 1 | 1 | 1 | 1 |
| 457 | 9 | 1 | 133 | 27 | 8 | 1 | 1 | 1 | 2 | 1 | 1 | 1 | 2 | 1 | 1 | 1 | 1 | 1 |
| 458 | 9 | 1 | 133 | 25 | 6 | 1 | 1 | 1 | 4 | 2 | 1 | 1 | 2 | 1 | 1 | 1 | 1 | 1 |
| 459 | 9 | 1 | 133.3 | 48.5 | 8 | 1 | 1 | 1 | 2 | 1 | 1 | 1 | 2 | 1 | 1 | 1 | 1 | 1 |
| 460 | 9 | 1 | 133.4 | 27.5 | 8 | 1 | 1 | 1 | 2 | 1 | 1 | 1 | 2 | 1 | 1 | 1 | 1 | 1 |
| 461 | 9 | 1 | 133.5 | 32.5 | 8 | 1 | 1 | 1 | 2 | 4 | 1 | 1 | 2 | 1 | 1 | 1 | 1 | 1 |
| 462 | 9 | 1 | 133.5 | 28 | 8 | 1 | 1 | 1 | 2 | 2 | 1 | 1 | 2 | 1 | 1 | 1 | 1 | 1 |
| 463 | 9 | 1 | 134 | 46 | 8 | 1 | 1 | 1 | 2 | 3 | 1 | 1 | 2 | 1 | 1 | 1 | 1 | 1 |
| 464 | 9 | 1 | 134 | 27 | 8 | 1 | 1 | 1 | 2 | 3 | 1 | 1 | 2 | 1 | 1 | 1 | 1 | 1 |
| 465 | 9 | 1 | 134 | 25 | 8 | 1 | 1 | 1 | 2 | 2 | 1 | 1 | 2 | 1 | 1 | 1 | 1 | 1 |
| 466 | 9 | 1 | 134.5 | 30.5 | 8 | 1 | 1 | 1 | 2 | 1 | 1 | 1 | 2 | 1 | 1 | 1 | 1 | 1 |
| 467 | 9 | 1 | 135 | 32.5 | 8 | 1 | 1 | 1 | 2 | 4 | 1 | 1 | 2 | 1 | 1 | 1 | 1 | 1 |
| 468 | 9 | 1 | 135 | 28 | 8 | 1 | 1 | 1 | 2 | 1 | 1 | 1 | 2 | 1 | 1 | 1 | 1 | 1 |
| 469 | 9 | 1 | 135 | 22 | 8 | 1 | 1 | 1 | 2 | 1 | 1 | 1 | 2 | 1 | 1 | 1 | 1 | 1 |
| 470 | 9 | 1 | 135.5 | 27.5 | 8 | 1 | 1 | 1 | 2 | 3 | 1 | 1 | 2 | 1 | 1 | 1 | 1 | 1 |
| 471 | 9 | 1 | 136 | 43 | 8 | 1 | 1 | 1 | 2 | 2 | 1 | 1 | 2 | 1 | 1 | 1 | 1 | 1 |
| 472 | 9 | 1 | 136 | 32.5 | 8 | 1 | 1 | 1 | 2 | 3 | 1 | 1 | 2 | 1 | 1 | 1 | 1 | 1 |
| 473 | 9 | 1 | 137 | 43 | 8 | 1 | 1 | 1 | 2 | 1 | 1 | 1 | 2 | 1 | 1 | 1 | 1 | 1 |
| 474 | 9 | 1 | 137 | 38 | 8 | 1 | 1 | 1 | 2 | 1 | 1 | 1 | 2 | 1 | 1 | 1 | 1 | 1 |
| 475 | 9 | 1 | 137 | 30 | 8 | 1 | 1 | 1 | 2 | 2 | 1 | 1 | 2 | 1 | 1 | 1 | 1 | 1 |
| 476 | 9 | 1 | 137.5 | 36 | 8 | 1 | 1 | 1 | 2 | 1 | 1 | 1 | 2 | 1 | 1 | 1 | 1 | 1 |
| 477 | 9 | 1 | 137.5 | 33 | 8 | 1 | 1 | 1 | 2 | 1 | 1 | 1 | 2 | 1 | 1 | 1 | 1 | 1 |
| 478 | 9 | 1 | 138 | 35.5 | 8 | 1 | 1 | 1 | 2 | 1 | 1 | 1 | 2 | 1 | 1 | 1 | 1 | 1 |
| 479 | 9 | 1 | 138 | 33 | 8 | 1 | 1 | 1 | 2 | 2 | 1 | 1 | 2 | 1 | 1 | 1 | 1 | 1 |
| 480 | 9 | 1 | 138.5 | 30 | 8 | 1 | 1 | 1 | 2 | 3 | 1 | 1 | 2 | 1 | 1 | 1 | 1 | 1 |
| 481 | 9 | 1 | 139 | 37 | 8 | 1 | 1 | 1 | 2 | 1 | 1 | 1 | 2 | 1 | 1 | 1 | 1 | 1 |
| 482 | 9 | 1 | 140 | 43 | 8 | 1 | 1 | 1 | 2 | 1 | 1 | 1 | 2 | 1 | 1 | 1 | 1 | 1 |
| 483 | 9 | 1 | 140 | 36 | 8 | 1 | 1 | 1 | 2 | 1 | 1 | 1 | 2 | 1 | 1 | 1 | 1 | 1 |
| 484 | 9 | 1 | 140 | 35 | 8 | 1 | 1 | 1 | 2 | 2 | 1 | 1 | 2 | 1 | 1 | 1 | 1 | 1 |
| 485 | 9 | 1 | 140 | 31 | 8 | 1 | 1 | 1 | 2 | 4 | 1 | 1 | 2 | 1 | 1 | 1 | 1 | 1 |
| 486 | 9 | 1 | 140 | 25 | 8 | 1 | 1 | 1 | 2 | 2 | 1 | 1 | 2 | 1 | 1 | 1 | 1 | 1 |
| 487 | 9 | 1 | 140.5 | 34 | 8 | 1 | 1 | 1 | 2 | 1 | 1 | 1 | 2 | 1 | 1 | 1 | 1 | 1 |
| 488 | 9 | 1 | 141 | 49.1 | 8 | 1 | 1 | 1 | 2 | 1 | 1 | 1 | 2 | 1 | 1 | 1 | 1 | 1 |
| 489 | 9 | 1 | 141 | 35 | 8 | 1 | 1 | 1 | 2 | 3 | 1 | 1 | 2 | 1 | 1 | 1 | 1 | 1 |
| 490 | 9 | 1 | 141.2 | 33 | 8 | 1 | 1 | 1 | 2 | 1 | 1 | 1 | 2 | 1 | 1 | 1 | 1 | 1 |
| 491 | 9 | 1 | 142 | 40 | 8 | 1 | 1 | 1 | 2 | 2 | 1 | 1 | 2 | 1 | 1 | 1 | 1 | 1 |
| 492 | 9 | 1 | 142 | 31 | 8 | 1 | 1 | 1 | 2 | 4 | 1 | 1 | 2 | 1 | 1 | 1 | 1 | 1 |
| 493 | 9 | 1 | 143 | 49.5 | 8 | 1 | 1 | 1 | 2 | 2 | 1 | 1 | 2 | 1 | 1 | 1 | 1 | 1 |
| 494 | 9 | 1 | 147.5 | 48 | 8 | 1 | 1 | 1 | 2 | 2 | 1 | 1 | 2 | 1 | 1 | 1 | 1 | 1 |
| 495 | 9 | 1 | 149 | 44 | 8 | 1 | 1 | 1 | 2 | 2 | 1 | 1 | 2 | 1 | 1 | 1 | 1 | 1 |
| 496 | 10 | 2 | 120 | 21 | 8 | 1 | 2 | 1 | 2 | 1 | 1 | 1 | 2 | 1 | 1 | 1 | 1 | 1 |
| 497 | 10 | 2 | 125.5 | 23 | 8 | 2 | 1 | 1 | 2 | 2 | 1 | 1 | 2 | 1 | 1 | 1 | 1 | 1 |
| 498 | 10 | 2 | 128 | 24 | 8 | 1 | 1 | 1 | 2 | 3 | 1 | 1 | 2 | 1 | 1 | 1 | 1 | 1 |
| 499 | 10 | 2 | 129 | 28 | 8 | 1 | 1 | 1 | 2 | 1 | 1 | 1 | 2 | 1 | 1 | 1 | 1 | 1 |
| 500 | 10 | 2 | 129 | 22.5 | 8 | 1 | 1 | 1 | 2 | 2 | 1 | 1 | 2 | 1 | 1 | 1 | 1 | 1 |
| 501 | 10 | 2 | 129.5 | 24 | 8 | 1 | 1 | 1 | 2 | 1 | 1 | 1 | 2 | 1 | 1 | 1 | 1 | 1 |
| 502 | 10 | 2 | 130 | 40 | 8 | 1 | 1 | 1 | 2 | 1 | 1 | 1 | 2 | 1 | 1 | 1 | 1 | 1 |
| 503 | 10 | 2 | 132 | 30 | 8 | 1 | 1 | 1 | 2 | 1 | 1 | 1 | 2 | 1 | 1 | 1 | 1 | 1 |
| 504 | 10 | 2 | 132 | 26.5 | 8 | 1 | 1 | 1 | 2 | 1 | 1 | 1 | 2 | 1 | 1 | 1 | 1 | 1 |
| 505 | 10 | 2 | 132 | 24.5 | 8 | 1 | 1 | 1 | 2 | 2 | 1 | 1 | 2 | 1 | 1 | 1 | 1 | 1 |
| 506 | 10 | 2 | 132.5 | 27.5 | 8 | 1 | 1 | 1 | 2 | 1 | 1 | 1 | 2 | 1 | 1 | 1 | 1 | 1 |
| 507 | 10 | 2 | 134 | 34 | 8 | 1 | 1 | 1 | 2 | 2 | 1 | 1 | 2 | 1 | 1 | 1 | 1 | 1 |
| 508 | 10 | 2 | 134 | 31.5 | 8 | 1 | 1 | 1 | 2 | 2 | 1 | 1 | 2 | 1 | 1 | 1 | 1 | 1 |
| 509 | 10 | 2 | 136 | 27 | 8 | 1 | 1 | 1 | 1 | 1 | 1 | 1 | 2 | 1 | 1 | 1 | 1 | 1 |
| 510 | 10 | 2 | 136 | 25 | 8 | 1 | 1 | 1 | 2 | 1 | 1 | 1 | 2 | 1 | 1 | 1 | 1 | 1 |
| 511 | 10 | 2 | 137 | 28 | 8 | 1 | 1 | 1 | 2 | 4 | 1 | 1 | 2 | 1 | 1 | 1 | 1 | 1 |
| 512 | 10 | 2 | 137.5 | 34.8 | 8 | 1 | 1 | 1 | 2 | 1 | 1 | 1 | 2 | 1 | 1 | 1 | 1 | 1 |
| 513 | 10 | 2 | 138 | 30 | 8 | 1 | 1 | 1 | 2 | 2 | 1 | 1 | 2 | 1 | 1 | 1 | 1 | 1 |
| 514 | 10 | 2 | 138 | 30 | 8 | 1 | 1 | 1 | 2 | 1 | 1 | 1 | 2 | 1 | 1 | 1 | 1 | 1 |
| 515 | 10 | 2 | 138 | 26.5 | 8 | 1 | 1 | 1 | 2 | 1 | 1 | 1 | 2 | 1 | 1 | 1 | 1 | 1 |
| 516 | 10 | 2 | 138 | 26 | 8 | 1 | 1 | 1 | 2 | 2 | 1 | 1 | 2 | 1 | 1 | 1 | 1 | 1 |
| 517 | 10 | 2 | 138 | 25 | 8 | 1 | 1 | 1 | 2 | 3 | 1 | 1 | 2 | 1 | 1 | 1 | 1 | 1 |
| 518 | 10 | 2 | 139 | 35 | 8 | 1 | 1 | 1 | 2 | 2 | 1 | 1 | 2 | 1 | 1 | 1 | 1 | 1 |
| 519 | 10 | 2 | 139 | 34.5 | 8 | 1 | 1 | 1 | 2 | 1 | 1 | 1 | 2 | 1 | 1 | 1 | 1 | 1 |
| 520 | 10 | 2 | 139.5 | 28.5 | 8 | 1 | 1 | 1 | 2 | 1 | 1 | 1 | 2 | 1 | 1 | 1 | 1 | 1 |
| 521 | 10 | 2 | 139.7 | 36.5 | 8 | 1 | 1 | 1 | 2 | 1 | 1 | 1 | 2 | 1 | 1 | 1 | 1 | 1 |
| 522 | 10 | 2 | 140 | 40 | 8 | 1 | 1 | 1 | 2 | 2 | 1 | 1 | 2 | 1 | 1 | 1 | 1 | 1 |
| 523 | 10 | 2 | 140 | 27 | 8 | 1 | 1 | 1 | 2 | 1 | 1 | 1 | 2 | 1 | 1 | 1 | 1 | 1 |
| 524 | 10 | 2 | 141 | 40.5 | 8 | 1 | 1 | 1 | 2 | 1 | 1 | 1 | 2 | 1 | 1 | 1 | 1 | 1 |
| 525 | 10 | 2 | 141 | 37 | 8 | 1 | 1 | 1 | 2 | 1 | 1 | 1 | 2 | 1 | 1 | 1 | 1 | 1 |
| 526 | 10 | 2 | 141.5 | 40 | 8 | 1 | 1 | 1 | 2 | 2 | 1 | 1 | 2 | 1 | 1 | 1 | 1 | 1 |
| 527 | 10 | 2 | 142 | 35 | 8 | 1 | 1 | 1 | 2 | 2 | 1 | 1 | 2 | 1 | 1 | 1 | 1 | 1 |
| 528 | 10 | 2 | 145 | 50 | 8 | 1 | 1 | 1 | 2 | 1 | 1 | 1 | 2 | 1 | 1 | 1 | 1 | 1 |
| 529 | 10 | 2 | 145 | 36 | 8 | 1 | 1 | 1 | 2 | 1 | 1 | 1 | 2 | 1 | 1 | 1 | 1 | 1 |
| 530 | 10 | 2 | 145 | 32 | 8 | 1 | 1 | 1 | 2 | 2 | 1 | 1 | 2 | 1 | 1 | 1 | 1 | 1 |
| 531 | 10 | 2 | 146 | 39 | 8 | 1 | 2 | 1 | 2 | 3 | 1 | 1 | 1 | 1 | 1 | 1 | 1 | 1 |
| 532 | 10 | 2 | 147 | 34 | 8 | 1 | 1 | 1 | 2 | 1 | 1 | 1 | 2 | 1 | 1 | 1 | 1 | 1 |
| 533 | 10 | 2 | 147.5 | 46 | 8 | 1 | 1 | 1 | 2 | 2 | 1 | 1 | 2 | 1 | 1 | 1 | 1 | 1 |
| 534 | 10 | 2 | 151 | 42.5 | 8 | 1 | 1 | 1 | 2 | 2 | 1 | 1 | 2 | 1 | 1 | 1 | 1 | 1 |
| 535 | 10 | 1 | 129 | 24 | 8 | 1 | 1 | 1 | 2 | 1 | 1 | 1 | 2 | 1 | 1 | 1 | 1 | 1 |
| 536 | 10 | 1 | 130.2 | 29 | 8 | 1 | 1 | 1 | 2 | 2 | 1 | 1 | 2 | 1 | 1 | 1 | 1 | 1 |
| 537 | 10 | 1 | 131 | 34 | 8 | 1 | 1 | 1 | 2 | 2 | 1 | 1 | 2 | 1 | 1 | 1 | 1 | 1 |
| 538 | 10 | 1 | 132 | 36 | 8 | 1 | 1 | 1 | 2 | 1 | 1 | 1 | 2 | 1 | 1 | 1 | 1 | 1 |
| 539 | 10 | 1 | 132 | 32 | 8 | 1 | 1 | 1 | 2 | 4 | 1 | 1 | 2 | 1 | 1 | 1 | 1 | 1 |
| 540 | 10 | 1 | 132 | 26 | 8 | 1 | 1 | 1 | 2 | 1 | 1 | 1 | 2 | 1 | 1 | 1 | 1 | 1 |
| 541 | 10 | 1 | 133 | 30.5 | 8 | 1 | 1 | 1 | 2 | 2 | 1 | 1 | 2 | 1 | 1 | 1 | 1 | 1 |
| 542 | 10 | 1 | 133 | 30 | 8 | 1 | 1 | 1 | 2 | 1 | 1 | 1 | 2 | 1 | 1 | 1 | 1 | 1 |
| 543 | 10 | 1 | 133 | 29 | 8 | 1 | 1 | 1 | 2 | 1 | 1 | 1 | 2 | 1 | 1 | 1 | 1 | 1 |
| 544 | 10 | 1 | 133 | 26 | 8 | 1 | 1 | 1 | 2 | 2 | 1 | 1 | 2 | 1 | 1 | 1 | 1 | 1 |
| 545 | 10 | 1 | 133 | 25 | 8 | 1 | 1 | 1 | 2 | 1 | 1 | 1 | 2 | 1 | 1 | 1 | 1 | 1 |
| 546 | 10 | 1 | 134 | 34 | 8 | 1 | 1 | 1 | 2 | 4 | 1 | 1 | 2 | 1 | 1 | 1 | 1 | 1 |
| 547 | 10 | 1 | 134 | 34 | 8 | 1 | 1 | 1 | 2 | 1 | 1 | 1 | 2 | 1 | 1 | 1 | 1 | 1 |
| 548 | 10 | 1 | 134 | 30 | 8 | 1 | 1 | 1 | 2 | 1 | 1 | 1 | 2 | 1 | 1 | 1 | 1 | 1 |
| 549 | 10 | 1 | 134.5 | 29 | 8 | 1 | 1 | 1 | 2 | 1 | 1 | 1 | 2 | 1 | 1 | 1 | 1 | 1 |
| 550 | 10 | 1 | 135.5 | 37.5 | 8 | 1 | 1 | 1 | 2 | 3 | 1 | 1 | 2 | 1 | 1 | 1 | 1 | 1 |
| 551 | 10 | 1 | 136 | 32 | 8 | 1 | 1 | 1 | 2 | 1 | 1 | 1 | 1 | 1 | 1 | 1 | 1 | 1 |
| 552 | 10 | 1 | 136.5 | 30.5 | 8 | 1 | 1 | 1 | 2 | 1 | 1 | 1 | 2 | 1 | 1 | 1 | 1 | 1 |
| 553 | 10 | 1 | 136.5 | 27.5 | 8 | 1 | 1 | 1 | 2 | 2 | 1 | 1 | 2 | 1 | 1 | 1 | 1 | 1 |
| 554 | 10 | 1 | 137 | 27 | 8 | 1 | 1 | 1 | 2 | 2 | 1 | 1 | 2 | 1 | 1 | 1 | 1 | 1 |
| 555 | 10 | 1 | 137.5 | 25 | 8 | 1 | 1 | 1 | 2 | 3 | 1 | 1 | 2 | 1 | 1 | 1 | 1 | 1 |
| 556 | 10 | 1 | 138 | 37 | 8 | 1 | 1 | 1 | 2 | 3 | 1 | 1 | 2 | 1 | 1 | 1 | 1 | 1 |
| 557 | 10 | 1 | 138.5 | 34.5 | 8 | 1 | 1 | 1 | 2 | 1 | 1 | 1 | 2 | 1 | 1 | 1 | 1 | 1 |
| 558 | 10 | 1 | 139 | 41 | 8 | 1 | 1 | 1 | 2 | 1 | 1 | 1 | 2 | 1 | 1 | 1 | 1 | 1 |
| 559 | 10 | 1 | 140 | 38 | 8 | 1 | 1 | 1 | 2 | 4 | 1 | 1 | 2 | 1 | 1 | 1 | 1 | 1 |
| 560 | 10 | 1 | 140 | 38 | 8 | 1 | 1 | 1 | 2 | 4 | 1 | 1 | 2 | 1 | 1 | 1 | 1 | 1 |
| 561 | 10 | 1 | 140 | 35 | 8 | 1 | 1 | 1 | 2 | 2 | 1 | 1 | 2 | 1 | 1 | 1 | 1 | 1 |
| 562 | 10 | 1 | 140 | 31.5 | 8 | 1 | 1 | 1 | 2 | 2 | 1 | 1 | 2 | 1 | 1 | 1 | 1 | 1 |
| 563 | 10 | 1 | 140 | 27 | 8 | 1 | 1 | 1 | 2 | 1 | 1 | 1 | 2 | 1 | 1 | 1 | 1 | 1 |
| 564 | 10 | 1 | 140.5 | 48.5 | 8 | 1 | 1 | 1 | 2 | 1 | 1 | 1 | 2 | 1 | 2 | 1 | 1 | 1 |
| 565 | 10 | 1 | 140.5 | 27 | 8 | 1 | 1 | 1 | 2 | 2 | 1 | 1 | 2 | 1 | 1 | 1 | 1 | 1 |
| 566 | 10 | 1 | 141 | 44.5 | 8 | 1 | 1 | 1 | 2 | 1 | 1 | 1 | 2 | 1 | 1 | 1 | 1 | 1 |
| 567 | 10 | 1 | 141 | 42 | 8 | 1 | 1 | 1 | 2 | 1 | 1 | 1 | 2 | 1 | 1 | 1 | 1 | 1 |
| 568 | 10 | 1 | 141 | 29.8 | 8 | 1 | 1 | 1 | 2 | 2 | 2 | 1 | 2 | 1 | 1 | 1 | 1 | 1 |
| 569 | 10 | 1 | 142 | 35.5 | 8 | 1 | 1 | 1 | 2 | 2 | 1 | 1 | 2 | 1 | 1 | 1 | 1 | 1 |
| 570 | 10 | 1 | 143.2 | 35 | 8 | 1 | 1 | 1 | 2 | 1 | 1 | 1 | 2 | 1 | 1 | 1 | 1 | 1 |
| 571 | 10 | 1 | 145 | 30 | 8 | 1 | 1 | 1 | 2 | 2 | 1 | 1 | 2 | 1 | 1 | 1 | 1 | 1 |
| 572 | 10 | 1 | 148 | 51 | 8 | 1 | 1 | 1 | 2 | 1 | 1 | 1 | 2 | 1 | 1 | 1 | 1 | 1 |
| 573 | 10 | 1 | 148 | 44 | 8 | 1 | 1 | 1 | 5 | 4 | 1 | 1 | 2 | 1 | 1 | 1 | 1 | 1 |
| 574 | 10 | 1 | 152 | 35 | 8 | 1 | 1 | 1 | 2 | 1 | 1 | 1 | 2 | 1 | 1 | 1 | 1 | 1 |
| 575 | 10 | 1 | 156 | 51 | 8 | 1 | 1 | 1 | 2 | 4 | 1 | 1 | 2 | 1 | 1 | 1 | 1 | 1 |
| 576 | 10 | 1 | 158 | 55 | 8 | 1 | 1 | 1 | 2 | 1 | 1 | 1 | 2 | 1 | 1 | 1 | 1 | 1 |
| 577 | 11 | 2 | 130 | 32 | 8 | 1 | 1 | 1 | 2 | 1 | 1 | 1 | 2 | 1 | 1 | 1 | 1 | 1 |
| 578 | 11 | 2 | 138 | 30 | 8 | 1 | 1 | 1 | 2 | 4 | 1 | 1 | 1 | 1 | 1 | 1 | 1 | 1 |
| 579 | 11 | 2 | 140 | 33.5 | 8 | 1 | 1 | 1 | 2 | 3 | 1 | 1 | 2 | 1 | 1 | 1 | 1 | 1 |
| 580 | 11 | 2 | 140 | 30 | 8 | 1 | 1 | 1 | 3 | 2 | 1 | 1 | 2 | 1 | 1 | 1 | 1 | 1 |
| 581 | 11 | 2 | 140 | 26 | 8 | 1 | 1 | 1 | 2 | 4 | 1 | 1 | 2 | 1 | 1 | 1 | 1 | 1 |
| 582 | 11 | 2 | 140.5 | 26 | 8 | 1 | 1 | 1 | 2 | 1 | 1 | 1 | 2 | 1 | 1 | 1 | 1 | 1 |
| 583 | 11 | 2 | 142 | 27.5 | 8 | 1 | 1 | 1 | 2 | 2 | 1 | 1 | 2 | 1 | 1 | 1 | 1 | 1 |
| 584 | 11 | 2 | 145 | 50 | 8 | 1 | 1 | 1 | 2 | 3 | 1 | 1 | 2 | 1 | 1 | 1 | 1 | 1 |
| 585 | 11 | 2 | 145 | 31.5 | 8 | 1 | 1 | 1 | 2 | 3 | 1 | 1 | 2 | 1 | 1 | 1 | 1 | 1 |
| 586 | 11 | 2 | 146 | 36.5 | 8 | 1 | 1 | 1 | 2 | 1 | 1 | 1 | 2 | 1 | 2 | 1 | 1 | 1 |
| 587 | 11 | 2 | 146 | 34 | 8 | 1 | 1 | 1 | 2 | 4 | 1 | 1 | 2 | 1 | 1 | 1 | 1 | 1 |
| 588 | 11 | 2 | 146 | 34 | 8 | 1 | 1 | 1 | 2 | 1 | 1 | 1 | 2 | 1 | 1 | 1 | 1 | 1 |
| 589 | 11 | 2 | 147 | 37.5 | 8 | 1 | 1 | 1 | 2 | 2 | 1 | 1 | 1 | 1 | 1 | 1 | 1 | 1 |
| 590 | 11 | 2 | 147 | 36 | 8 | 1 | 1 | 1 | 2 | 1 | 1 | 1 | 2 | 1 | 1 | 1 | 1 | 1 |
| 591 | 11 | 2 | 148 | 37.5 | 8 | 1 | 1 | 1 | 2 | 2 | 1 | 1 | 2 | 1 | 1 | 1 | 1 | 1 |
| 592 | 11 | 2 | 148 | 35 | 8 | 1 | 1 | 1 | 2 | 3 | 1 | 1 | 2 | 1 | 1 | 1 | 1 | 1 |
| 593 | 11 | 2 | 149 | 40 | 8 | 1 | 1 | 1 | 2 | 2 | 1 | 1 | 2 | 1 | 1 | 1 | 1 | 1 |
| 594 | 11 | 2 | 149 | 39 | 8 | 1 | 1 | 1 | 3 | 2 | 1 | 1 | 2 | 1 | 1 | 1 | 1 | 1 |
| 595 | 11 | 2 | 150 | 32 | 8 | 1 | 1 | 1 | 2 | 3 | 1 | 1 | 2 | 1 | 1 | 1 | 1 | 1 |
| 596 | 11 | 2 | 150 | 32 | 8 | 1 | 1 | 1 | 2 | 1 | 1 | 1 | 2 | 1 | 1 | 1 | 1 | 1 |
| 597 | 11 | 2 | 151 | 42 | 8 | 1 | 1 | 1 | 2 | 1 | 1 | 1 | 2 | 1 | 1 | 1 | 1 | 1 |
| 598 | 11 | 2 | 151 | 34.5 | 8 | 1 | 1 | 1 | 2 | 1 | 1 | 1 | 2 | 1 | 1 | 1 | 1 | 1 |
| 599 | 11 | 2 | 151 | 32 | 8 | 1 | 1 | 1 | 2 | 1 | 1 | 1 | 2 | 1 | 1 | 1 | 1 | 1 |
| 600 | 11 | 2 | 151.5 | 35.5 | 8 | 1 | 1 | 1 | 2 | 1 | 1 | 1 | 2 | 1 | 1 | 1 | 1 | 1 |
| 601 | 11 | 2 | 152 | 39.5 | 8 | 1 | 1 | 1 | 2 | 2 | 1 | 1 | 2 | 1 | 1 | 1 | 1 | 1 |
| 602 | 11 | 2 | 153 | 45 | 8 | 1 | 1 | 1 | 2 | 1 | 1 | 1 | 2 | 1 | 1 | 1 | 1 | 1 |
| 603 | 11 | 2 | 153 | 34 | 8 | 1 | 2 | 1 | 2 | 1 | 1 | 1 | 2 | 1 | 1 | 1 | 1 | 1 |
| 604 | 11 | 2 | 155 | 44.5 | 8 | 1 | 1 | 1 | 2 | 1 | 1 | 1 | 2 | 1 | 1 | 1 | 1 | 1 |
| 605 | 11 | 2 | 163 | 48 | 8 | 1 | 1 | 1 | 2 | 1 | 1 | 1 | 2 | 1 | 1 | 1 | 1 | 1 |
| 606 | 11 | 2 | 166 | 51 | 8 | 1 | 2 | 1 | 2 | 1 | 1 | 1 | 2 | 1 | 1 | 1 | 1 | 1 |
| 607 | 11 | 1 | 121 | 40.5 | 8 | 1 | 1 | 1 | 2 | 1 | 1 | 1 | 2 | 1 | 1 | 1 | 1 | 1 |
| 608 | 11 | 1 | 130.5 | 25 | 8 | 1 | 1 | 1 | 2 | 2 | 1 | 1 | 2 | 1 | 1 | 1 | 1 | 1 |
| 609 | 11 | 1 | 131 | 26 | 8 | 1 | 1 | 1 | 2 | 3 | 1 | 1 | 2 | 1 | 1 | 1 | 1 | 1 |
| 610 | 11 | 1 | 136 | 32 | 8 | 1 | 1 | 1 | 2 | 4 | 1 | 1 | 2 | 1 | 1 | 1 | 1 | 1 |
| 611 | 11 | 1 | 136 | 31 | 8 | 1 | 1 | 1 | 2 | 1 | 1 | 1 | 2 | 1 | 1 | 1 | 1 | 1 |
| 612 | 11 | 1 | 137 | 32.5 | 8 | 1 | 1 | 1 | 2 | 1 | 1 | 1 | 2 | 1 | 1 | 1 | 1 | 1 |
| 613 | 11 | 1 | 138 | 34 | 8 | 1 | 1 | 1 | 2 | 3 | 1 | 1 | 2 | 1 | 1 | 1 | 1 | 1 |
| 614 | 11 | 1 | 138 | 26 | 8 | 1 | 1 | 1 | 2 | 1 | 1 | 1 | 2 | 1 | 1 | 1 | 1 | 1 |
| 615 | 11 | 1 | 138 | 25 | 8 | 1 | 1 | 1 | 2 | 4 | 1 | 1 | 1 | 1 | 1 | 1 | 1 | 1 |
| 616 | 11 | 1 | 138.5 | 36.5 | 8 | 1 | 1 | 1 | 2 | 3 | 1 | 1 | 2 | 1 | 1 | 1 | 1 | 1 |
| 617 | 11 | 1 | 140 | 35 | 8 | 1 | 1 | 1 | 2 | 2 | 1 | 1 | 2 | 1 | 1 | 1 | 1 | 1 |
| 618 | 11 | 1 | 140 | 28 | 8 | 1 | 1 | 1 | 2 | 1 | 1 | 1 | 2 | 1 | 1 | 1 | 1 | 1 |
| 619 | 11 | 1 | 141 | 47 | 8 | 1 | 1 | 1 | 2 | 1 | 1 | 1 | 2 | 1 | 1 | 1 | 1 | 1 |
| 620 | 11 | 1 | 141 | 30 | 8 | 1 | 1 | 1 | 2 | 1 | 1 | 1 | 2 | 1 | 1 | 1 | 1 | 1 |
| 621 | 11 | 1 | 143.5 | 45 | 8 | 1 | 1 | 1 | 2 | 1 | 1 | 1 | 2 | 1 | 2 | 1 | 1 | 1 |
| 622 | 11 | 1 | 144 | 32.5 | 8 | 1 | 1 | 1 | 2 | 1 | 1 | 1 | 2 | 1 | 1 | 1 | 1 | 1 |
| 623 | 11 | 1 | 145 | 35 | 8 | 1 | 1 | 1 | 2 | 1 | 1 | 1 | 2 | 1 | 1 | 1 | 1 | 1 |
| 624 | 11 | 1 | 145 | 23 | 8 | 1 | 1 | 1 | 2 | 4 | 1 | 1 | 2 | 1 | 1 | 1 | 1 | 1 |
| 625 | 11 | 1 | 146 | 46.5 | 8 | 1 | 1 | 1 | 2 | 2 | 1 | 1 | 2 | 1 | 1 | 1 | 1 | 1 |
| 626 | 11 | 1 | 146 | 43.5 | 8 | 1 | 1 | 1 | 2 | 1 | 1 | 1 | 2 | 1 | 1 | 1 | 1 | 1 |
| 627 | 11 | 1 | 147 | 41.5 | 8 | 1 | 1 | 1 | 2 | 2 | 1 | 1 | 2 | 1 | 1 | 1 | 1 | 1 |
| 628 | 11 | 1 | 147 | 38.5 | 8 | 1 | 1 | 1 | 2 | 1 | 1 | 1 | 2 | 1 | 1 | 1 | 1 | 1 |
| 629 | 11 | 1 | 147 | 38 | 8 | 1 | 1 | 1 | 2 | 2 | 1 | 1 | 2 | 1 | 2 | 2 | 1 | 1 |
| 630 | 11 | 1 | 147 | 31.5 | 8 | 1 | 1 | 1 | 2 | 4 | 1 | 1 | 2 | 1 | 1 | 1 | 1 | 1 |
| 631 | 11 | 1 | 149 | 45.5 | 8 | 1 | 2 | 1 | 2 | 1 | 1 | 1 | 2 | 1 | 1 | 1 | 1 | 1 |
| 632 | 11 | 1 | 150 | 40 | 8 | 1 | 1 | 1 | 2 | 1 | 1 | 1 | 2 | 1 | 1 | 1 | 1 | 1 |
| 633 | 11 | 1 | 150 | 35 | 8 | 1 | 1 | 1 | 2 | 4 | 1 | 1 | 2 | 1 | 1 | 1 | 1 | 1 |
| 634 | 11 | 1 | 150 | 33 | 8 | 1 | 1 | 1 | 2 | 1 | 1 | 1 | 2 | 1 | 1 | 1 | 1 | 1 |
| 635 | 11 | 1 | 150 | 30 | 8 | 1 | 1 | 1 | 2 | 2 | 1 | 1 | 2 | 1 | 1 | 1 | 1 | 1 |
| 636 | 11 | 1 | 150.5 | 38 | 8 | 1 | 2 | 1 | 2 | 1 | 1 | 1 | 2 | 1 | 1 | 1 | 1 | 1 |
| 637 | 11 | 1 | 154 | 57 | 8 | 1 | 1 | 1 | 2 | 1 | 1 | 1 | 2 | 1 | 1 | 1 | 1 | 1 |
| 638 | 11 | 1 | 154.5 | 36 | 8 | 1 | 1 | 1 | 2 | 2 | 1 | 1 | 2 | 1 | 1 | 1 | 1 | 1 |
| 639 | 11 | 1 | 157 | 49.5 | 8 | 1 | 1 | 1 | 2 | 1 | 1 | 1 | 2 | 1 | 1 | 1 | 1 | 1 |
| 640 | 11 | 1 | 157 | 40 | 8 | 1 | 1 | 1 | 2 | 1 | 1 | 1 | 2 | 1 | 1 | 1 | 1 | 1 |
| 641 | 11 | 1 | 159 | 53 | 8 | 1 | 1 | 1 | 2 | 2 | 1 | 1 | 2 | 1 | 1 | 1 | 1 | 1 |
| 642 | 11 | 1 | 159.5 | 40 | 8 | 1 | 1 | 1 | 2 | 3 | 1 | 1 | 2 | 1 | 1 | 1 | 1 | 1 |
| 643 | 12 | 2 | 124 | 23 | 8 | 1 | 1 | 1 | 2 | 3 | 1 | 1 | 2 | 1 | 1 | 1 | 1 | 1 |
| 644 | 12 | 2 | 140 | 35 | 8 | 1 | 1 | 1 | 2 | 2 | 1 | 1 | 2 | 1 | 1 | 1 | 1 | 1 |
| 645 | 12 | 2 | 141 | 29 | 8 | 1 | 1 | 1 | 2 | 1 | 1 | 1 | 2 | 1 | 1 | 1 | 1 | 1 |
| 646 | 12 | 2 | 143 | 30 | 8 | 1 | 1 | 1 | 2 | 4 | 1 | 1 | 2 | 1 | 1 | 1 | 1 | 1 |
| 647 | 12 | 2 | 144 | 33.5 | 8 | 1 | 1 | 1 | 2 | 1 | 1 | 1 | 2 | 1 | 1 | 1 | 1 | 1 |
| 648 | 12 | 2 | 146 | 27 | 8 | 1 | 1 | 1 | 2 | 1 | 1 | 1 | 2 | 1 | 1 | 1 | 1 | 1 |
| 649 | 12 | 2 | 147 | 37 | 8 | 1 | 1 | 1 | 2 | 2 | 1 | 1 | 2 | 1 | 1 | 1 | 1 | 1 |
| 650 | 12 | 2 | 148 | 34.5 | 8 | 1 | 1 | 1 | 2 | 2 | 1 | 1 | 2 | 1 | 1 | 1 | 1 | 1 |
| 651 | 12 | 2 | 149 | 36.5 | 8 | 1 | 1 | 1 | 2 | 1 | 1 | 1 | 2 | 1 | 1 | 1 | 1 | 1 |
| 652 | 12 | 2 | 150 | 46 | 8 | 1 | 1 | 1 | 2 | 2 | 1 | 1 | 2 | 1 | 1 | 1 | 1 | 1 |
| 653 | 12 | 2 | 150 | 34.5 | 8 | 1 | 1 | 1 | 2 | 3 | 1 | 1 | 2 | 1 | 1 | 1 | 1 | 1 |
| 654 | 12 | 2 | 152 | 52.5 | 8 | 1 | 1 | 1 | 2 | 4 | 1 | 1 | 2 | 1 | 1 | 1 | 1 | 1 |
| 655 | 12 | 2 | 152.5 | 47 | 8 | 1 | 1 | 1 | 2 | 2 | 1 | 1 | 2 | 1 | 1 | 1 | 1 | 1 |
| 656 | 12 | 2 | 155 | 49.5 | 8 | 2 | 1 | 1 | 2 | 1 | 1 | 1 | 2 | 1 | 1 | 1 | 1 | 1 |
| 657 | 12 | 2 | 155 | 41 | 8 | 1 | 1 | 1 | 2 | 3 | 1 | 1 | 2 | 1 | 1 | 1 | 1 | 1 |
| 658 | 12 | 2 | 155 | 37 | 8 | 1 | 1 | 1 | 2 | 3 | 1 | 1 | 2 | 1 | 1 | 1 | 1 | 1 |
| 659 | 12 | 2 | 156 | 53 | 8 | 1 | 1 | 1 | 4 | 4 | 1 | 1 | 1 | 1 | 1 | 1 | 1 | 1 |
| 660 | 12 | 2 | 156 | 42 | 8 | 1 | 1 | 1 | 2 | 1 | 1 | 1 | 2 | 1 | 1 | 1 | 1 | 1 |
| 661 | 12 | 2 | 160 | 45 | 8 | 1 | 1 | 1 | 2 | 3 | 1 | 1 | 2 | 1 | 1 | 1 | 1 | 1 |
| 662 | 12 | 2 | 162 | 80.5 | 8 | 1 | 1 | 1 | 2 | 4 | 1 | 1 | 2 | 1 | 1 | 1 | 1 | 1 |
| 663 | 12 | 2 | 166 | 65 | 8 | 1 | 1 | 1 | 2 | 3 | 1 | 1 | 2 | 1 | 1 | 1 | 1 | 1 |
| 664 | 12 | 2 | 166 | 50.5 | 8 | 1 | 1 | 1 | 2 | 2 | 1 | 1 | 2 | 1 | 1 | 1 | 1 | 1 |
| 665 | 12 | 2 | 167 | 64 | 8 | 1 | 1 | 1 | 2 | 2 | 1 | 1 | 2 | 1 | 1 | 1 | 1 | 1 |
| 666 | 12 | 1 | 136 | 28 | 8 | 1 | 1 | 1 | 2 | 4 | 1 | 1 | 2 | 1 | 1 | 1 | 1 | 1 |
| 667 | 12 | 1 | 138 | 40 | 8 | 1 | 1 | 1 | 3 | 1 | 1 | 1 | 2 | 1 | 1 | 1 | 1 | 1 |
| 668 | 12 | 1 | 139 | 25 | 8 | 1 | 1 | 1 | 2 | 1 | 1 | 1 | 2 | 1 | 1 | 1 | 1 | 1 |
| 669 | 12 | 1 | 144.5 | 40 | 8 | 1 | 1 | 1 | 2 | 4 | 1 | 1 | 2 | 1 | 1 | 1 | 1 | 1 |
| 670 | 12 | 1 | 145 | 30 | 8 | 1 | 1 | 1 | 2 | 2 | 1 | 1 | 2 | 1 | 1 | 1 | 1 | 1 |
| 671 | 12 | 1 | 148 | 54.5 | 8 | 1 | 1 | 1 | 2 | 2 | 1 | 1 | 1 | 1 | 1 | 1 | 1 | 1 |
| 672 | 12 | 1 | 148 | 45 | 8 | 1 | 1 | 1 | 2 | 3 | 1 | 1 | 2 | 1 | 1 | 1 | 1 | 1 |
| 673 | 12 | 1 | 148 | 43.5 | 8 | 1 | 1 | 1 | 2 | 1 | 1 | 1 | 2 | 1 | 1 | 1 | 1 | 1 |
| 674 | 12 | 1 | 148 | 42 | 8 | 1 | 2 | 1 | 2 | 1 | 1 | 1 | 2 | 2 | 1 | 1 | 1 | 2 |
| 675 | 12 | 1 | 148 | 38 | 8 | 1 | 1 | 1 | 2 | 2 | 1 | 1 | 2 | 1 | 1 | 1 | 1 | 1 |
| 676 | 12 | 1 | 148 | 37 | 8 | 1 | 1 | 1 | 2 | 3 | 1 | 1 | 2 | 1 | 1 | 1 | 1 | 1 |
| 677 | 12 | 1 | 148.5 | 35 | 8 | 1 | 1 | 1 | 2 | 2 | 1 | 1 | 2 | 1 | 1 | 1 | 1 | 1 |
| 678 | 12 | 1 | 149.5 | 35 | 8 | 1 | 1 | 1 | 2 | 1 | 1 | 1 | 2 | 1 | 1 | 1 | 1 | 1 |
| 679 | 12 | 1 | 150 | 50 | 8 | 1 | 1 | 1 | 2 | 3 | 1 | 1 | 2 | 1 | 1 | 1 | 1 | 1 |
| 680 | 12 | 1 | 150 | 46 | 8 | 1 | 1 | 1 | 2 | 4 | 1 | 1 | 2 | 1 | 1 | 1 | 1 | 1 |
| 681 | 12 | 1 | 151 | 38.5 | 8 | 1 | 1 | 1 | 2 | 2 | 1 | 1 | 2 | 1 | 1 | 1 | 1 | 1 |
| 682 | 12 | 1 | 152 | 67.5 | 8 | 1 | 1 | 1 | 2 | 3 | 1 | 1 | 2 | 1 | 1 | 1 | 1 | 1 |
| 683 | 12 | 1 | 154 | 44 | 8 | 1 | 1 | 1 | 2 | 2 | 1 | 1 | 2 | 1 | 1 | 1 | 1 | 1 |
| 684 | 12 | 1 | 155 | 49.5 | 8 | 1 | 1 | 1 | 2 | 3 | 3 | 1 | 2 | 1 | 1 | 1 | 1 | 1 |
| 685 | 12 | 1 | 155.5 | 60 | 8 | 1 | 1 | 1 | 2 | 2 | 1 | 1 | 2 | 1 | 1 | 1 | 1 | 1 |
| 686 | 12 | 1 | 158 | 79.5 | 8 | 1 | 1 | 1 | 2 | 4 | 1 | 1 | 2 | 1 | 1 | 1 | 1 | 1 |
| 687 | 12 | 1 | 159.5 | 43.5 | 8 | 1 | 1 | 1 | 2 | 2 | 1 | 1 | 2 | 1 | 1 | 1 | 1 | 1 |
| 688 | 12 | 1 | 160 | 50 | 8 | 1 | 1 | 1 | 2 | 1 | 1 | 1 | 2 | 1 | 1 | 1 | 1 | 1 |
| 689 | 12 | 1 | 161 | 38.5 | 8 | 1 | 1 | 1 | 2 | 2 | 1 | 1 | 2 | 1 | 1 | 1 | 1 | 1 |
| 690 | 12 | 1 | 163 | 52.5 | 8 | 1 | 1 | 1 | 2 | 3 | 1 | 1 | 2 | 1 | 1 | 1 | 1 | 1 |
| 691 | 12 | 1 | 166 | 60 | 8 | 1 | 1 | 1 | 2 | 2 | 1 | 1 | 2 | 1 | 2 | 1 | 1 | 1 |
| 692 | 12 | 1 | 175 | 60.5 | 8 | 1 | 1 | 1 | 3 | 2 | 1 | 1 | 2 | 1 | 1 | 1 | 1 | 1 |
| 693 | 13 | 2 | 136.5 | 30.5 | 8 | 1 | 1 | 1 | 3 | 1 | 1 | 1 | 2 | 1 | 1 | 1 | 1 | 1 |
| 694 | 13 | 2 | 143.5 | 35 | 8 | 1 | 1 | 1 | 3 | 3 | 1 | 1 | 2 | 1 | 1 | 1 | 1 | 1 |
| 695 | 13 | 2 | 148 | 32.5 | 8 | 1 | 1 | 1 | 3 | 2 | 1 | 1 | 2 | 1 | 1 | 1 | 1 | 1 |
| 696 | 13 | 2 | 150 | 45 | 8 | 1 | 1 | 1 | 3 | 2 | 1 | 1 | 2 | 1 | 1 | 1 | 1 | 1 |
| 697 | 13 | 2 | 150 | 41.2 | 8 | 2 | 1 | 1 | 3 | 1 | 1 | 1 | 2 | 1 | 1 | 1 | 1 | 1 |
| 698 | 13 | 2 | 150 | 40.5 | 8 | 1 | 1 | 1 | 3 | 1 | 1 | 2 | 2 | 1 | 1 | 1 | 1 | 1 |
| 699 | 13 | 2 | 150.2 | 39.5 | 8 | 1 | 1 | 1 | 3 | 2 | 1 | 1 | 2 | 1 | 1 | 1 | 1 | 1 |
| 700 | 13 | 2 | 150.5 | 46.5 | 8 | 1 | 1 | 1 | 3 | 2 | 1 | 1 | 2 | 1 | 1 | 1 | 1 | 1 |
| 701 | 13 | 2 | 151 | 36.5 | 8 | 1 | 1 | 1 | 2 | 1 | 1 | 1 | 2 | 1 | 1 | 1 | 1 | 1 |
| 702 | 13 | 2 | 151 | 35 | 1 | 2 | 1 | 1 | 3 | 1 | 1 | 1 | 2 | 1 | 1 | 1 | 1 | 1 |
| 703 | 13 | 2 | 152 | 48.5 | 8 | 1 | 1 | 1 | 3 | 2 | 1 | 1 | 2 | 1 | 1 | 1 | 1 | 1 |
| 704 | 13 | 2 | 152 | 43.5 | 8 | 1 | 1 | 1 | 2 | 3 | 1 | 1 | 2 | 1 | 1 | 1 | 1 | 1 |
| 705 | 13 | 2 | 152.5 | 50.5 | 8 | 1 | 1 | 1 | 3 | 1 | 1 | 1 | 2 | 1 | 1 | 1 | 1 | 1 |
| 706 | 13 | 2 | 153 | 42 | 8 | 1 | 1 | 1 | 2 | 4 | 1 | 1 | 2 | 1 | 1 | 1 | 1 | 1 |
| 707 | 13 | 2 | 153.2 | 58.5 | 8 | 1 | 1 | 1 | 3 | 2 | 1 | 1 | 2 | 1 | 1 | 1 | 1 | 1 |
| 708 | 13 | 2 | 153.8 | 39 | 8 | 1 | 1 | 1 | 3 | 2 | 1 | 1 | 2 | 1 | 1 | 1 | 1 | 1 |
| 709 | 13 | 2 | 154.5 | 47 | 8 | 1 | 1 | 1 | 3 | 2 | 1 | 1 | 2 | 1 | 1 | 1 | 1 | 1 |
| 710 | 13 | 2 | 155 | 47 | 8 | 1 | 1 | 1 | 3 | 2 | 1 | 1 | 2 | 1 | 1 | 1 | 1 | 1 |
| 711 | 13 | 2 | 155 | 45 | 8 | 1 | 1 | 1 | 3 | 2 | 1 | 1 | 2 | 1 | 1 | 1 | 1 | 1 |
| 712 | 13 | 2 | 155 | 41 | 8 | 1 | 1 | 1 | 3 | 2 | 1 | 1 | 2 | 1 | 1 | 1 | 1 | 1 |
| 713 | 13 | 2 | 155 | 36.4 | 8 | 1 | 1 | 1 | 3 | 2 | 1 | 1 | 2 | 1 | 1 | 1 | 1 | 1 |
| 714 | 13 | 2 | 156 | 43.5 | 8 | 1 | 1 | 1 | 3 | 2 | 1 | 1 | 2 | 1 | 1 | 1 | 1 | 1 |
| 715 | 13 | 2 | 156.5 | 44.5 | 8 | 1 | 1 | 1 | 3 | 3 | 1 | 1 | 2 | 1 | 1 | 1 | 1 | 1 |
| 716 | 13 | 2 | 157 | 46.5 | 8 | 1 | 1 | 1 | 3 | 2 | 1 | 1 | 2 | 1 | 1 | 1 | 1 | 1 |
| 717 | 13 | 2 | 157.5 | 46.3 | 8 | 1 | 1 | 1 | 3 | 1 | 1 | 1 | 2 | 1 | 1 | 1 | 1 | 1 |
| 718 | 13 | 2 | 158 | 46 | 8 | 1 | 1 | 1 | 3 | 1 | 1 | 1 | 2 | 1 | 1 | 1 | 1 | 1 |
| 719 | 13 | 2 | 158 | 43 | 8 | 1 | 1 | 1 | 3 | 1 | 1 | 1 | 2 | 1 | 1 | 1 | 1 | 1 |
| 720 | 13 | 2 | 158.5 | 48 | 8 | 1 | 1 | 1 | 3 | 1 | 1 | 1 | 2 | 1 | 1 | 1 | 1 | 1 |
| 721 | 13 | 2 | 159 | 57 | 8 | 1 | 1 | 1 | 3 | 2 | 1 | 1 | 2 | 1 | 1 | 1 | 1 | 1 |
| 722 | 13 | 2 | 159 | 45 | 8 | 1 | 1 | 1 | 3 | 1 | 1 | 1 | 2 | 1 | 1 | 1 | 1 | 1 |
| 723 | 13 | 2 | 159.5 | 49.5 | 8 | 1 | 1 | 1 | 3 | 1 | 1 | 1 | 2 | 1 | 1 | 1 | 1 | 1 |
| 724 | 13 | 2 | 159.5 | 46 | 8 | 1 | 1 | 1 | 3 | 1 | 1 | 1 | 2 | 1 | 1 | 1 | 1 | 1 |
| 725 | 13 | 2 | 160 | 51 | 8 | 1 | 1 | 1 | 3 | 2 | 1 | 1 | 1 | 1 | 1 | 1 | 1 | 1 |
| 726 | 13 | 2 | 160.5 | 51 | 8 | 1 | 1 | 1 | 3 | 1 | 1 | 1 | 2 | 1 | 1 | 1 | 1 | 1 |
| 727 | 13 | 2 | 161 | 50 | 8 | 1 | 1 | 1 | 3 | 2 | 1 | 1 | 2 | 1 | 1 | 1 | 1 | 1 |
| 728 | 13 | 2 | 161 | 48.5 | 8 | 1 | 1 | 1 | 3 | 1 | 1 | 1 | 2 | 1 | 2 | 1 | 1 | 1 |
| 729 | 13 | 2 | 161 | 47.5 | 8 | 1 | 1 | 1 | 3 | 2 | 1 | 1 | 2 | 1 | 1 | 1 | 1 | 1 |
| 730 | 13 | 2 | 162 | 60 | 8 | 1 | 1 | 1 | 3 | 1 | 1 | 1 | 2 | 1 | 1 | 1 | 1 | 1 |
| 731 | 13 | 2 | 162 | 44 | 8 | 1 | 1 | 1 | 3 | 4 | 1 | 1 | 2 | 1 | 1 | 1 | 1 | 1 |
| 732 | 13 | 2 | 166.5 | 65 | 8 | 1 | 1 | 1 | 3 | 2 | 1 | 1 | 2 | 1 | 1 | 1 | 1 | 1 |
| 733 | 13 | 2 | 167.5 | 44.5 | 8 | 1 | 1 | 1 | 3 | 2 | 1 | 1 | 2 | 1 | 1 | 1 | 1 | 1 |
| 734 | 13 | 2 | 168 | 48.8 | 8 | 1 | 1 | 1 | 3 | 1 | 1 | 1 | 2 | 1 | 1 | 1 | 1 | 1 |
| 735 | 13 | 2 | 169 | 79 | 8 | 1 | 1 | 1 | 3 | 2 | 1 | 1 | 2 | 1 | 1 | 1 | 1 | 1 |
| 736 | 13 | 2 | 169.5 | 60.5 | 8 | 1 | 1 | 1 | 3 | 2 | 1 | 1 | 2 | 1 | 1 | 1 | 1 | 1 |
| 737 | 13 | 2 | 171 | 56 | 8 | 1 | 1 | 1 | 3 | 3 | 1 | 1 | 2 | 1 | 1 | 1 | 1 | 1 |
| 738 | 13 | 2 | 172 | 54 | 8 | 1 | 1 | 1 | 3 | 3 | 1 | 1 | 2 | 1 | 1 | 1 | 1 | 1 |
| 739 | 13 | 1 | 144 | 42 | 8 | 1 | 1 | 1 | 3 | 3 | 1 | 1 | 1 | 1 | 1 | 1 | 1 | 2 |
| 740 | 13 | 1 | 145 | 30 | 8 | 1 | 1 | 1 | 2 | 4 | 1 | 1 | 2 | 1 | 1 | 1 | 1 | 1 |
| 741 | 13 | 1 | 148 | 35 | 8 | 1 | 1 | 1 | 3 | 1 | 1 | 1 | 2 | 1 | 1 | 1 | 1 | 1 |
| 742 | 13 | 1 | 148.3 | 38.5 | 8 | 1 | 1 | 1 | 3 | 2 | 1 | 1 | 2 | 1 | 1 | 1 | 1 | 1 |
| 743 | 13 | 1 | 150 | 45 | 8 | 1 | 1 | 1 | 3 | 1 | 1 | 1 | 2 | 1 | 1 | 1 | 1 | 1 |
| 744 | 13 | 1 | 152 | 59 | 8 | 1 | 1 | 1 | 3 | 1 | 1 | 1 | 2 | 1 | 1 | 1 | 1 | 1 |
| 745 | 13 | 1 | 152 | 38 | 8 | 1 | 1 | 1 | 2 | 1 | 1 | 1 | 2 | 1 | 1 | 1 | 1 | 1 |
| 746 | 13 | 1 | 153 | 44 | 8 | 1 | 1 | 1 | 3 | 2 | 1 | 1 | 2 | 1 | 1 | 1 | 1 | 1 |
| 747 | 13 | 1 | 153.4 | 46.5 | 8 | 1 | 2 | 1 | 3 | 3 | 1 | 1 | 2 | 1 | 1 | 1 | 1 | 1 |
| 748 | 13 | 1 | 154 | 48.5 | 8 | 1 | 1 | 1 | 3 | 2 | 1 | 1 | 2 | 1 | 1 | 1 | 1 | 1 |
| 749 | 13 | 1 | 155 | 46 | 8 | 1 | 1 | 1 | 3 | 2 | 1 | 1 | 2 | 1 | 1 | 1 | 1 | 1 |
| 750 | 13 | 1 | 155 | 45 | 8 | 1 | 1 | 1 | 3 | 1 | 1 | 1 | 2 | 1 | 1 | 1 | 1 | 1 |
| 751 | 13 | 1 | 155 | 43 | 8 | 1 | 1 | 1 | 3 | 2 | 1 | 1 | 2 | 1 | 1 | 1 | 1 | 1 |
| 752 | 13 | 1 | 155.8 | 47 | 8 | 1 | 1 | 1 | 3 | 2 | 2 | 1 | 2 | 1 | 1 | 1 | 1 | 1 |
| 753 | 13 | 1 | 157 | 49.8 | 8 | 1 | 1 | 1 | 3 | 2 | 1 | 1 | 2 | 1 | 1 | 1 | 1 | 1 |
| 754 | 13 | 1 | 157 | 46.9 | 8 | 1 | 1 | 1 | 3 | 2 | 1 | 1 | 2 | 1 | 1 | 1 | 1 | 1 |
| 755 | 13 | 1 | 157 | 38.1 | 8 | 1 | 1 | 1 | 3 | 2 | 1 | 1 | 1 | 1 | 1 | 1 | 1 | 1 |
| 756 | 13 | 1 | 158.5 | 40 | 8 | 1 | 1 | 1 | 3 | 2 | 1 | 1 | 2 | 1 | 1 | 1 | 1 | 1 |
| 757 | 13 | 1 | 159 | 57.4 | 8 | 1 | 1 | 1 | 3 | 3 | 1 | 1 | 2 | 1 | 1 | 1 | 1 | 1 |
| 758 | 13 | 1 | 159 | 41.8 | 8 | 1 | 1 | 1 | 3 | 3 | 1 | 1 | 2 | 1 | 1 | 1 | 1 | 1 |
| 759 | 13 | 1 | 159.5 | 51.5 | 8 | 1 | 1 | 1 | 3 | 1 | 1 | 1 | 2 | 1 | 1 | 1 | 1 | 1 |
| 760 | 13 | 1 | 160 | 51 | 8 | 1 | 1 | 1 | 3 | 1 | 1 | 1 | 2 | 1 | 1 | 1 | 1 | 1 |
| 761 | 13 | 1 | 160 | 44.2 | 8 | 1 | 1 | 1 | 3 | 2 | 1 | 1 | 2 | 1 | 1 | 1 | 1 | 1 |
| 762 | 13 | 1 | 161.5 | 50.5 | 8 | 1 | 1 | 1 | 3 | 2 | 1 | 1 | 2 | 1 | 1 | 1 | 1 | 1 |
| 763 | 13 | 1 | 162 | 46.5 | 8 | 1 | 1 | 1 | 3 | 1 | 1 | 1 | 2 | 1 | 1 | 1 | 1 | 1 |
| 764 | 13 | 1 | 162 | 42.5 | 8 | 1 | 1 | 1 | 3 | 2 | 1 | 1 | 2 | 1 | 1 | 1 | 1 | 1 |
| 765 | 13 | 1 | 162.5 | 49.5 | 8 | 1 | 1 | 1 | 3 | 2 | 1 | 1 | 2 | 1 | 1 | 1 | 1 | 1 |
| 766 | 13 | 1 | 163 | 52 | 8 | 1 | 1 | 1 | 3 | 1 | 1 | 1 | 2 | 1 | 1 | 1 | 1 | 1 |
| 767 | 13 | 1 | 163.5 | 46 | 8 | 1 | 1 | 1 | 3 | 2 | 1 | 1 | 2 | 1 | 1 | 1 | 1 | 1 |
| 768 | 13 | 1 | 164.5 | 59 | 8 | 1 | 1 | 1 | 3 | 1 | 1 | 1 | 1 | 1 | 1 | 1 | 1 | 1 |
| 769 | 13 | 1 | 166 | 70 | 8 | 1 | 2 | 1 | 3 | 1 | 1 | 1 | 2 | 1 | 1 | 1 | 1 | 1 |
| 770 | 13 | 1 | 166 | 44.3 | 8 | 1 | 1 | 1 | 3 | 2 | 1 | 1 | 2 | 1 | 1 | 1 | 1 | 1 |
| 771 | 13 | 1 | 167 | 40 | 8 | 1 | 1 | 1 | 3 | 1 | 1 | 1 | 2 | 1 | 1 | 1 | 1 | 1 |
| 772 | 13 | 1 | 168 | 76.5 | 8 | 1 | 2 | 1 | 3 | 2 | 1 | 1 | 2 | 1 | 1 | 1 | 1 | 1 |
| 773 | 13 | 1 | 168 | 49.4 | 8 | 1 | 1 | 1 | 3 | 2 | 1 | 1 | 2 | 1 | 1 | 1 | 1 | 1 |
| 774 | 13 | 1 | 169 | 65 | 8 | 1 | 1 | 1 | 3 | 1 | 1 | 1 | 2 | 1 | 1 | 1 | 1 | 1 |
| 775 | 13 | 1 | 170 | 44 | 8 | 1 | 1 | 1 | 2 | 1 | 1 | 1 | 2 | 1 | 1 | 1 | 1 | 1 |
| 776 | 13 | 1 | 170.5 | 54 | 8 | 1 | 1 | 1 | 3 | 2 | 1 | 1 | 2 | 1 | 1 | 1 | 1 | 1 |
| 777 | 13 | 1 | 171 | 50 | 8 | 1 | 1 | 1 | 3 | 2 | 1 | 1 | 2 | 1 | 1 | 1 | 1 | 1 |
| 778 | 14 | 2 | 146.3 | 35 | 8 | 1 | 1 | 1 | 3 | 2 | 1 | 1 | 2 | 1 | 1 | 1 | 1 | 1 |
| 779 | 14 | 2 | 150 | 34 | 8 | 1 | 1 | 1 | 3 | 2 | 1 | 1 | 2 | 1 | 1 | 1 | 1 | 1 |
| 780 | 14 | 2 | 153.5 | 44.5 | 8 | 1 | 1 | 1 | 3 | 1 | 1 | 1 | 2 | 1 | 1 | 1 | 1 | 1 |
| 781 | 14 | 2 | 155 | 45 | 8 | 1 | 1 | 1 | 3 | 3 | 1 | 1 | 1 | 1 | 1 | 1 | 1 | 1 |
| 782 | 14 | 2 | 155 | 45 | 8 | 1 | 1 | 1 | 3 | 1 | 1 | 1 | 2 | 1 | 1 | 1 | 1 | 1 |
| 783 | 14 | 2 | 156 | 45 | 8 | 1 | 1 | 1 | 3 | 1 | 1 | 1 | 1 | 1 | 1 | 1 | 1 | 1 |
| 784 | 14 | 2 | 157 | 50.5 | 8 | 1 | 1 | 1 | 3 | 2 | 1 | 1 | 2 | 1 | 1 | 1 | 1 | 1 |
| 785 | 14 | 2 | 157 | 40 | 8 | 1 | 1 | 1 | 3 | 4 | 1 | 1 | 2 | 1 | 1 | 1 | 1 | 1 |
| 786 | 14 | 2 | 157.5 | 47 | 8 | 1 | 1 | 1 | 3 | 2 | 1 | 1 | 2 | 1 | 1 | 1 | 1 | 1 |
| 787 | 14 | 2 | 160 | 62 | 8 | 1 | 1 | 1 | 3 | 1 | 1 | 1 | 2 | 1 | 1 | 1 | 1 | 1 |
| 788 | 14 | 2 | 160 | 49.5 | 8 | 1 | 1 | 1 | 3 | 3 | 1 | 1 | 2 | 1 | 1 | 1 | 1 | 1 |
| 789 | 14 | 2 | 161 | 45 | 8 | 1 | 1 | 1 | 3 | 4 | 1 | 1 | 1 | 1 | 1 | 1 | 1 | 1 |
| 790 | 14 | 2 | 163 | 45 | 8 | 1 | 1 | 1 | 3 | 2 | 1 | 1 | 2 | 1 | 1 | 1 | 1 | 1 |
| 791 | 14 | 2 | 168 | 55 | 8 | 1 | 1 | 1 | 3 | 2 | 1 | 1 | 2 | 1 | 1 | 1 | 1 | 1 |
| 792 | 14 | 1 | 150 | 40.5 | 8 | 1 | 1 | 1 | 3 | 1 | 1 | 1 | 2 | 1 | 1 | 1 | 1 | 1 |
| 793 | 14 | 1 | 156.5 | 45 | 8 | 1 | 1 | 1 | 3 | 1 | 1 | 1 | 2 | 1 | 1 | 1 | 1 | 1 |
| 794 | 14 | 1 | 159 | 65.5 | 8 | 1 | 1 | 1 | 3 | 3 | 1 | 1 | 2 | 1 | 1 | 1 | 1 | 1 |
| 795 | 14 | 1 | 159.5 | 40 | 8 | 1 | 1 | 1 | 3 | 1 | 1 | 1 | 2 | 1 | 1 | 1 | 1 | 1 |
| 796 | 14 | 1 | 160.3 | 49.5 | 8 | 1 | 2 | 1 | 3 | 3 | 1 | 1 | 2 | 1 | 1 | 1 | 1 | 1 |
| 797 | 14 | 1 | 165 | 51.5 | 8 | 1 | 1 | 1 | 3 | 1 | 1 | 1 | 2 | 1 | 1 | 1 | 1 | 1 |
| 798 | 14 | 1 | 165.5 | 40.5 | 8 | 1 | 1 | 1 | 3 | 2 | 1 | 1 | 2 | 1 | 1 | 1 | 1 | 1 |
| 799 | 14 | 1 | 167 | 51.8 | 8 | 1 | 1 | 1 | 3 | 2 | 1 | 1 | 2 | 1 | 1 | 1 | 1 | 1 |
| 800 | 14 | 1 | 167.5 | 53 | 8 | 1 | 1 | 1 | 3 | 2 | 1 | 1 | 2 | 1 | 1 | 1 | 1 | 1 |
| 801 | 14 | 1 | 167.5 | 52.5 | 8 | 1 | 1 | 1 | 3 | 2 | 1 | 1 | 2 | 1 | 1 | 1 | 1 | 1 |
| 802 | 14 | 1 | 170 | 62 | 8 | 1 | 1 | 1 | 3 | 2 | 1 | 1 | 2 | 1 | 2 | 1 | 1 | 1 |
| 803 | 14 | 1 | 170.5 | 82 | 8 | 1 | 1 | 1 | 3 | 2 | 1 | 1 | 2 | 1 | 1 | 1 | 1 | 1 |
| 804 | 14 | 1 | 172 | 50 | 8 | 1 | 1 | 1 | 3 | 1 | 1 | 1 | 2 | 1 | 1 | 1 | 1 | 1 |
| 805 | 14 | 1 | 176 | 85 | 8 | 1 | 1 | 1 | 3 | 2 | 1 | 1 | 2 | 1 | 1 | 1 | 1 | 1 |
| 806 | 14 | 1 | 179 | 84.5 | 8 | 1 | 1 | 1 | 3 | 1 | 1 | 1 | 2 | 1 | 1 | 1 | 1 | 1 |
| 807 | 15 | 2 | 153 | 42 | 8 | 1 | 1 | 1 | 3 | 1 | 1 | 1 | 2 | 1 | 1 | 1 | 1 | 1 |
| 808 | 15 | 2 | 153 | 40 | 8 | 1 | 1 | 1 | 3 | 1 | 1 | 1 | 2 | 1 | 1 | 1 | 1 | 1 |
| 809 | 15 | 2 | 156 | 43 | 8 | 1 | 1 | 1 | 3 | 1 | 1 | 1 | 2 | 1 | 1 | 1 | 1 | 1 |
| 810 | 15 | 2 | 162 | 45 | 8 | 1 | 2 | 1 | 3 | 1 | 1 | 1 | 2 | 1 | 1 | 1 | 1 | 1 |
| 811 | 15 | 1 | 155 | 50 | 8 | 1 | 1 | 1 | 3 | 1 | 1 | 1 | 1 | 1 | 1 | 1 | 1 | 1 |
| 812 | 15 | 1 | 158 | 38 | 8 | 1 | 1 | 1 | 3 | 2 | 1 | 1 | 2 | 1 | 1 | 1 | 1 | 1 |
| 813 | 15 | 1 | 169 | 67 | 8 | 1 | 1 | 1 | 3 | 2 | 2 | 1 | 2 | 1 | 1 | 1 | 1 | 1 |
| 814 | 15 | 1 | 170 | 56 | 8 | 1 | 1 | 1 | 3 | 2 | 1 | 1 | 2 | 1 | 1 | 1 | 1 | 1 |
| 815 | 15 | 1 | 170 | 45 | 8 | 1 | 1 | 1 | 3 | 1 | 1 | 1 | 2 | 1 | 1 | 1 | 1 | 1 |
| 816 | 15 | 1 | 173.5 | 56.7 | 8 | 1 | 1 | 1 | 3 | 2 | 1 | 1 | 2 | 1 | 1 | 1 | 1 | 1 |
| 817 | 15 | 1 | 174 | 82 | 8 | 1 | 1 | 1 | 3 | 2 | 1 | 1 | 1 | 1 | 1 | 1 | 1 | 1 |
| 818 | 16 | 2 | 158.2 | 43.2 | 8 | 1 | 1 | 1 | 2 | 2 | 1 | 1 | 2 | 1 | 1 | 1 | 1 | 1 |
| 819 | 16 | 1 | 170 | 60 | 8 | 1 | 1 | 1 | 4 | 1 | 1 | 1 | 2 | 1 | 1 | 1 | 1 | 1 |
| 820 | 16 | 1 | 175 | 65 | 8 | 1 | 1 | 1 | 3 | 1 | 1 | 1 | 2 | 1 | 2 | 1 | 1 | 1 |
| 821 | 16 | 1 | 176 | 78 | 8 | 1 | 1 | 1 | 3 | 1 | 1 | 1 | 2 | 1 | 1 | 1 | 1 | 1 |
| 822 | 17 | 2 | 153 | 50 | 8 | 1 | 1 | 1 | 4 | 4 | 1 | 1 | 1 | 1 | 1 | 1 | 1 | 1 |
| 823 | 17 | 1 | 172 | 66 | 8 | 1 | 2 | 1 | 4 | 1 | 2 | 1 | 2 | 1 | 1 | 1 | 1 | 1 |
| 824 | 17 | 1 | 173 | 50 | 8 | 1 | 1 | 1 | 3 | 1 | 1 | 1 | 2 | 1 | 1 | 1 | 1 | 1 |
| 825 | 17 | 1 | 177 | 60 | 8 | 1 | 1 | 1 | 3 | 1 | 1 | 1 | 2 | 1 | 1 | 1 | 1 | 1 |
| 826 | 17 | 1 | 177 | 53 | 8 | 1 | 1 | 1 | 3 | 2 | 1 | 1 | 2 | 1 | 1 | 1 | 1 | 1 |
| 827 | 17 | 1 | 180 | 57 | 8 | 1 | 2 | 1 | 3 | 1 | 1 | 1 | 1 | 1 | 1 | 1 | 1 | 1 |
| 828 | 18 | 2 | 152 | 46 | 2 | 1 | 1 | 1 | 4 | 3 | 1 | 1 | 1 | 1 | 1 | 1 | 1 | 1 |
| 829 | 18 | 2 | 155 | 50 | 8 | 1 | 1 | 1 | 3 | 1 | 1 | 1 | 2 | 1 | 1 | 1 | 1 | 1 |
| 830 | 18 | 2 | 156 | 41.5 | 4 | 1 | 1 | 1 | 3 | 1 | 1 | 1 | 1 | 1 | 1 | 1 | 1 | 1 |
| 831 | 18 | 2 | 160 | 44 | 8 | 1 | 1 | 1 | 4 | 2 | 1 | 1 | 2 | 1 | 1 | 1 | 1 | 1 |
| 832 | 18 | 2 | 163 | 49 | 8 | 1 | 1 | 1 | 4 | 4 | 1 | 1 | 1 | 1 | 1 | 1 | 1 | 1 |
| 833 | 19 | 2 | 153 | 40 | 8 | 1 | 1 | 1 | 4 | 3 | 1 | 1 | 1 | 1 | 1 | 1 | 1 | 1 |
| 834 | 19 | 2 | 157 | 51.6 | 3 | 1 | 1 | 1 | 4 | 4 | 1 | 1 | 2 | 1 | 1 | 1 | 1 | 1 |
| 835 | 19 | 2 | 159 | 49 | 8 | 1 | 1 | 1 | 4 | 3 | 1 | 1 | 2 | 1 | 1 | 1 | 1 | 1 |
| 836 | 19 | 2 | 160 | 54 | 8 | 1 | 1 | 1 | 5 | 3 | 1 | 1 | 2 | 1 | 1 | 1 | 1 | 1 |
| 837 | 19 | 2 | 163 | 54 | 3 | 2 | 1 | 1 | 4 | 4 | 1 | 1 | 2 | 1 | 1 | 1 | 1 | 1 |
| 838 | 19 | 2 | 170 | 64 | 8 | 2 | 1 | 1 | 4 | 2 | 1 | 1 | 2 | 1 | 1 | 1 | 1 | 1 |
| 839 | 19 | 1 | 175 | 63 | 8 | 1 | 1 | 1 | 4 | 1 | 1 | 1 | 1 | 1 | 1 | 1 | 1 | 1 |
| 840 | 20 | 2 | 153 | 46 | 2 | 1 | 1 | 1 | 4 | 4 | 1 | 1 | 1 | 1 | 1 | 1 | 1 | 1 |
| 841 | 20 | 2 | 158 | 44 | 8 | 1 | 1 | 1 | 4 | 4 | 1 | 1 | 2 | 1 | 1 | 1 | 1 | 1 |
| 842 | 20 | 2 | 159 | 51 | 8 | 1 | 1 | 1 | 5 | 2 | 1 | 1 | 2 | 1 | 1 | 1 | 1 | 1 |
| 843 | 20 | 2 | 164 | 58 | 8 | 1 | 1 | 1 | 5 | 4 | 1 | 1 | 2 | 1 | 1 | 1 | 1 | 1 |
| 844 | 20 | 2 | 167 | 56 | 2 | 2 | 1 | 2 | 5 | 3 | 1 | 1 | 2 | 1 | 1 | 1 | 1 | 1 |
| 845 | 20 | 1 | 165 | 65 | 2 | 1 | 1 | 1 | 4 | 2 | 1 | 1 | 1 | 1 | 2 | 1 | 1 | 1 |
| 846 | 20 | 1 | 173 | 60 | 3 | 1 | 1 | 1 | 5 | 2 | 1 | 1 | 1 | 1 | 1 | 1 | 1 | 1 |
| 847 | 20 | 1 | 179 | 75 | 8 | 1 | 1 | 1 | 5 | 2 | 1 | 1 | 2 | 1 | 1 | 1 | 1 | 1 |
| 848 | 21 | 2 | 153 | 55 | 8 | 1 | 1 | 1 | 5 | 2 | 2 | 1 | 2 | 1 | 1 | 1 | 1 | 1 |
| 849 | 21 | 2 | 153 | 40 | 2 | 1 | 1 | 1 | 5 | 3 | 1 | 1 | 1 | 1 | 1 | 1 | 1 | 1 |
| 850 | 21 | 2 | 157 | 50 | 8 | 1 | 1 | 2 | 5 | 3 | 1 | 1 | 1 | 1 | 1 | 1 | 1 | 1 |
| 851 | 21 | 2 | 160 | 55 | 8 | 1 | 1 | 1 | 5 | 2 | 1 | 1 | 2 | 1 | 1 | 1 | 1 | 1 |
| 852 | 21 | 2 | 162 | 49 | 8 | 1 | 1 | 1 | 4 | 4 | 1 | 1 | 1 | 1 | 1 | 1 | 1 | 1 |
| 853 | 21 | 2 | 167 | 71.6 | 4 | 1 | 1 | 1 | 5 | 3 | 1 | 1 | 2 | 1 | 1 | 1 | 1 | 1 |
| 854 | 21 | 2 | 171 | 60 | 2 | 1 | 1 | 1 | 4 | 3 | 1 | 1 | 1 | 1 | 1 | 1 | 1 | 1 |
| 855 | 21 | 1 | 170 | 70 | 3 | 1 | 1 | 1 | 6 | 4 | 1 | 1 | 1 | 1 | 1 | 1 | 1 | 2 |
| 856 | 21 | 1 | 178 | 75 | 8 | 1 | 1 | 1 | 5 | 3 | 1 | 1 | 2 | 1 | 1 | 1 | 1 | 1 |
| 857 | 22 | 2 | 155 | 48 | 8 | 1 | 1 | 1 | 5 | 2 | 1 | 1 | 2 | 1 | 1 | 1 | 1 | 1 |
| 858 | 22 | 2 | 159 | 96 | 4 | 2 | 1 | 1 | 5 | 4 | 1 | 1 | 2 | 1 | 1 | 1 | 1 | 1 |
| 859 | 22 | 2 | 159 | 49 | 8 | 1 | 1 | 1 | 5 | 3 | 1 | 1 | 2 | 1 | 1 | 1 | 1 | 1 |
| 860 | 22 | 2 | 160 | 53 | 2 | 1 | 1 | 1 | 4 | 1 | 1 | 1 | 1 | 1 | 1 | 1 | 1 | 1 |
| 861 | 22 | 2 | 162 | 67 | 2 | 2 | 1 | 1 | 4 | 4 | 1 | 1 | 1 | 1 | 1 | 1 | 1 | 1 |
| 862 | 22 | 2 | 164.5 | 47.5 | 8 | 1 | 1 | 1 | 5 | 1 | 1 | 1 | 1 | 1 | 1 | 1 | 1 | 1 |
| 863 | 22 | 2 | 165 | 52 | 8 | 1 | 1 | 1 | 5 | 3 | 1 | 1 | 2 | 1 | 1 | 1 | 1 | 1 |
| 864 | 22 | 1 | 163.5 | 55.4 | 8 | 1 | 1 | 1 | 4 | 4 | 1 | 1 | 1 | 1 | 1 | 1 | 1 | 1 |
| 865 | 22 | 1 | 165 | 50 | 8 | 1 | 1 | 1 | 5 | 4 | 1 | 1 | 2 | 1 | 1 | 1 | 1 | 1 |
| 866 | 22 | 1 | 169 | 54 | 8 | 1 | 1 | 1 | 4 | 3 | 1 | 1 | 1 | 1 | 1 | 1 | 1 | 1 |
| 867 | 22 | 1 | 172 | 69 | 8 | 1 | 1 | 1 | 5 | 2 | 1 | 1 | 1 | 1 | 1 | 1 | 1 | 1 |
| 868 | 22 | 1 | 179 | 67.5 | 8 | 2 | 1 | 1 | 4 | 4 | 1 | 1 | 2 | 1 | 1 | 1 | 1 | 1 |
| 869 | 23 | 2 | 155 | 60.8 | 8 | 1 | 1 | 1 | 4 | 4 | 1 | 1 | 2 | 1 | 1 | 1 | 1 | 1 |
| 870 | 23 | 2 | 155 | 47 | 2 | 1 | 1 | 1 | 4 | 2 | 1 | 1 | 1 | 1 | 1 | 1 | 1 | 1 |
| 871 | 23 | 2 | 155 | 43.5 | 4 | 1 | 1 | 1 | 4 | 1 | 1 | 1 | 2 | 1 | 1 | 1 | 1 | 2 |
| 872 | 23 | 2 | 157 | 57 | 8 | 1 | 1 | 1 | 5 | 4 | 1 | 1 | 2 | 1 | 1 | 1 | 1 | 1 |
| 873 | 23 | 2 | 160 | 55 | 8 | 1 | 1 | 1 | 5 | 3 | 1 | 1 | 2 | 1 | 1 | 1 | 1 | 1 |
| 874 | 23 | 2 | 160 | 55 | 4 | 1 | 1 | 1 | 5 | 3 | 1 | 1 | 2 | 1 | 1 | 1 | 1 | 1 |
| 875 | 23 | 2 | 160 | 52.5 | 2 | 2 | 1 | 1 | 5 | 2 | 1 | 1 | 1 | 1 | 1 | 1 | 1 | 1 |
| 876 | 23 | 2 | 160 | 51 | 2 | 1 | 2 | 1 | 3 | 4 | 1 | 1 | 1 | 1 | 1 | 1 | 1 | 1 |
| 877 | 23 | 2 | 160 | 48 | 8 | 1 | 1 | 1 | 5 | 1 | 1 | 1 | 2 | 1 | 1 | 1 | 1 | 1 |
| 878 | 23 | 2 | 163 | 50 | 8 | 1 | 1 | 1 | 5 | 2 | 1 | 1 | 1 | 1 | 1 | 1 | 1 | 1 |
| 879 | 23 | 2 | 163 | 50 | 2 | 1 | 1 | 1 | 5 | 4 | 1 | 1 | 2 | 1 | 1 | 1 | 1 | 1 |
| 880 | 23 | 2 | 164 | 55 | 8 | 1 | 1 | 1 | 1 | 4 | 1 | 1 | 1 | 1 | 1 | 1 | 1 | 1 |
| 881 | 23 | 2 | 165 | 60 | 8 | 1 | 1 | 1 | 5 | 4 | 1 | 1 | 2 | 1 | 1 | 1 | 1 | 1 |
| 882 | 23 | 2 | 178 | 59 | 8 | 2 | 1 | 1 | 5 | 4 | 1 | 1 | 1 | 1 | 1 | 1 | 1 | 1 |
| 883 | 23 | 1 | 159 | 47 | 8 | 1 | 1 | 1 | 4 | 4 | 1 | 1 | 1 | 1 | 1 | 1 | 1 | 1 |
| 884 | 23 | 1 | 172 | 65 | 4 | 2 | 2 | 1 | 5 | 4 | 1 | 1 | 1 | 1 | 1 | 1 | 1 | 1 |
| 885 | 23 | 1 | 173 | 89 | 2 | 1 | 1 | 1 | 5 | 4 | 1 | 1 | 1 | 1 | 1 | 1 | 1 | 1 |
| 886 | 24 | 2 | 151 | 49.5 | 3 | 1 | 1 | 1 | 5 | 4 | 1 | 1 | 1 | 1 | 1 | 1 | 1 | 1 |
| 887 | 24 | 2 | 152 | 45 | 2 | 1 | 1 | 1 | 4 | 3 | 1 | 1 | 2 | 1 | 1 | 1 | 1 | 1 |
| 888 | 24 | 2 | 156 | 54 | 8 | 1 | 2 | 1 | 5 | 4 | 1 | 1 | 1 | 1 | 1 | 1 | 1 | 1 |
| 889 | 24 | 2 | 156 | 43 | 8 | 1 | 1 | 1 | 5 | 2 | 1 | 1 | 2 | 1 | 1 | 1 | 1 | 1 |
| 890 | 24 | 2 | 158 | 56 | 8 | 2 | 1 | 1 | 4 | 4 | 1 | 1 | 2 | 1 | 1 | 1 | 1 | 1 |
| 891 | 24 | 2 | 160 | 43 | 2 | 1 | 1 | 1 | 4 | 2 | 1 | 1 | 2 | 1 | 1 | 1 | 1 | 1 |
| 892 | 24 | 2 | 162 | 60 | 8 | 1 | 1 | 1 | 5 | 2 | 1 | 1 | 2 | 1 | 1 | 1 | 1 | 1 |
| 893 | 24 | 2 | 163 | 51 | 2 | 1 | 1 | 1 | 5 | 1 | 1 | 1 | 1 | 1 | 1 | 1 | 1 | 1 |
| 894 | 24 | 2 | 165 | 49 | 2 | 1 | 1 | 1 | 4 | 2 | 1 | 1 | 1 | 1 | 1 | 1 | 1 | 1 |
| 895 | 24 | 2 | 175 | 84.5 | 4 | 1 | 1 | 1 | 5 | 3 | 1 | 1 | 1 | 1 | 1 | 1 | 1 | 1 |
| 896 | 24 | 1 | 163 | 59 | 3 | 1 | 1 | 1 | 5 | 1 | 1 | 1 | 1 | 1 | 1 | 1 | 3 | 1 |
| 897 | 24 | 1 | 164 | 50.5 | 4 | 1 | 1 | 1 | 5 | 3 | 1 | 1 | 1 | 1 | 1 | 1 | 1 | 1 |
| 898 | 24 | 1 | 166 | 78 | 8 | 1 | 1 | 1 | 4 | 1 | 1 | 1 | 1 | 1 | 1 | 1 | 1 | 1 |
| 899 | 24 | 1 | 166 | 60 | 3 | 1 | 1 | 1 | 4 | 1 | 1 | 1 | 1 | 1 | 1 | 1 | 1 | 2 |
| 900 | 24 | 1 | 168 | 95 | 8 | 1 | 1 | 1 | 5 | 2 | 1 | 1 | 2 | 1 | 1 | 1 | 1 | 1 |
| 901 | 24 | 1 | 170 | 80 | 4 | 1 | 1 | 1 | 5 | 3 | 1 | 1 | 2 | 1 | 1 | 1 | 1 | 1 |
| 902 | 24 | 1 | 170 | 70 | 8 | 1 | 1 | 1 | 5 | 4 | 1 | 1 | 1 | 1 | 1 | 1 | 1 | 1 |
| 903 | 24 | 1 | 171 | 67 | 2 | 1 | 1 | 2 | 5 | 2 | 1 | 1 | 1 | 1 | 1 | 1 | 1 | 1 |
| 904 | 25 | 2 | 150 | 58 | 2 | 1 | 1 | 1 | 5 | 3 | 1 | 1 | 2 | 1 | 1 | 1 | 1 | 1 |
| 905 | 25 | 2 | 150 | 53.5 | 4 | 1 | 1 | 2 | 5 | 4 | 1 | 1 | 2 | 1 | 1 | 1 | 1 | 1 |
| 906 | 25 | 2 | 150 | 50 | 8 | 2 | 1 | 1 | 5 | 1 | 1 | 1 | 1 | 1 | 1 | 1 | 1 | 1 |
| 907 | 25 | 2 | 154.5 | 44 | 8 | 1 | 1 | 1 | 5 | 4 | 1 | 1 | 1 | 1 | 1 | 1 | 1 | 1 |
| 908 | 25 | 2 | 155 | 52.5 | 4 | 1 | 1 | 2 | 5 | 3 | 1 | 1 | 2 | 1 | 1 | 1 | 1 | 1 |
| 909 | 25 | 2 | 155 | 41.5 | 2 | 1 | 1 | 1 | 4 | 2 | 1 | 1 | 2 | 1 | 1 | 1 | 1 | 1 |
| 910 | 25 | 2 | 156 | 47 | 4 | 2 | 1 | 1 | 4 | 4 | 1 | 1 | 2 | 1 | 1 | 1 | 1 | 1 |
| 911 | 25 | 2 | 157 | 49 | 3 | 1 | 1 | 1 | 5 | 2 | 1 | 1 | 1 | 1 | 1 | 1 | 1 | 1 |
| 912 | 25 | 2 | 157 | 45 | 8 | 1 | 1 | 1 | 4 | 3 | 1 | 1 | 2 | 1 | 1 | 1 | 1 | 1 |
| 913 | 25 | 2 | 158 | 58 | 4 | 1 | 1 | 1 | 5 | 2 | 1 | 1 | 2 | 1 | 1 | 1 | 1 | 1 |
| 914 | 25 | 2 | 159 | 57 | 8 | 2 | 1 | 1 | 5 | 4 | 1 | 1 | 1 | 1 | 1 | 1 | 1 | 1 |
| 915 | 25 | 2 | 159 | 44 | 4 | 2 | 1 | 1 | 5 | 3 | 1 | 1 | 2 | 1 | 1 | 1 | 1 | 1 |
| 916 | 25 | 2 | 160 | 48.5 | 8 | 1 | 2 | 2 | 4 | 2 | 1 | 1 | 2 | 1 | 1 | 1 | 1 | 1 |
| 917 | 25 | 2 | 160 | 47 | 2 | 1 | 1 | 1 | 5 | 2 | 1 | 1 | 1 | 1 | 1 | 1 | 1 | 1 |
| 918 | 25 | 2 | 162 | 60 | 8 | 2 | 1 | 1 | 3 | 3 | 1 | 1 | 1 | 1 | 1 | 1 | 1 | 1 |
| 919 | 25 | 2 | 162 | 55 | 8 | 2 | 1 | 1 | 5 | 3 | 1 | 1 | 2 | 1 | 1 | 1 | 1 | 1 |
| 920 | 25 | 2 | 162 | 50 | 4 | 2 | 1 | 1 | 5 | 2 | 1 | 1 | 1 | 1 | 1 | 1 | 1 | 1 |
| 921 | 25 | 2 | 162 | 43 | 8 | 1 | 1 | 2 | 5 | 4 | 1 | 1 | 2 | 1 | 1 | 1 | 1 | 1 |
| 922 | 25 | 2 | 163 | 60 | 4 | 2 | 1 | 1 | 5 | 2 | 1 | 1 | 2 | 1 | 1 | 1 | 1 | 1 |
| 923 | 25 | 2 | 163 | 50 | 4 | 2 | 1 | 1 | 5 | 4 | 1 | 1 | 1 | 1 | 1 | 1 | 1 | 1 |
| 924 | 25 | 2 | 164 | 51 | 2 | 2 | 1 | 1 | 5 | 1 | 1 | 1 | 1 | 1 | 1 | 1 | 1 | 1 |
| 925 | 25 | 2 | 164.2 | 64.5 | 4 | 2 | 1 | 1 | 5 | 1 | 1 | 1 | 1 | 1 | 1 | 1 | 1 | 1 |
| 926 | 25 | 2 | 165 | 55 | 8 | 2 | 1 | 1 | 3 | 2 | 1 | 1 | 1 | 1 | 1 | 1 | 1 | 1 |
| 927 | 25 | 2 | 165 | 53 | 2 | 1 | 1 | 1 | 5 | 2 | 1 | 1 | 1 | 1 | 1 | 1 | 1 | 1 |
| 928 | 25 | 2 | 167 | 50 | 2 | 1 | 1 | 1 | 4 | 2 | 1 | 1 | 2 | 1 | 1 | 1 | 1 | 1 |
| 929 | 25 | 2 | 168 | 67 | 4 | 2 | 1 | 1 | 4 | 3 | 1 | 1 | 1 | 1 | 1 | 1 | 1 | 1 |
| 930 | 25 | 2 | 168 | 52 | 8 | 2 | 1 | 1 | 6 | 4 | 1 | 1 | 1 | 1 | 1 | 1 | 1 | 1 |
| 931 | 25 | 1 | 164 | 74.5 | 2 | 1 | 1 | 1 | 5 | 4 | 1 | 2 | 1 | 1 | 1 | 1 | 1 | 1 |
| 932 | 25 | 1 | 165 | 63 | 2 | 1 | 1 | 2 | 5 | 2 | 3 | 1 | 1 | 1 | 1 | 1 | 2 | 1 |
| 933 | 25 | 1 | 167 | 63.5 | 4 | 1 | 1 | 3 | 5 | 2 | 1 | 1 | 2 | 1 | 1 | 1 | 1 | 1 |
| 934 | 25 | 1 | 167 | 58 | 2 | 1 | 1 | 2 | 6 | 2 | 1 | 1 | 1 | 1 | 1 | 1 | 1 | 1 |
| 935 | 25 | 1 | 168 | 65 | 4 | 1 | 1 | 3 | 5 | 3 | 1 | 1 | 2 | 1 | 1 | 1 | 1 | 1 |
| 936 | 25 | 1 | 171 | 59 | 2 | 1 | 1 | 2 | 5 | 3 | 1 | 1 | 1 | 1 | 1 | 1 | 3 | 1 |
| 937 | 25 | 1 | 177.5 | 79.5 | 4 | 1 | 1 | 1 | 5 | 3 | 1 | 1 | 1 | 1 | 1 | 1 | 1 | 1 |
| 938 | 25 | 1 | 178 | 62 | 8 | 1 | 1 | 1 | 5 | 2 | 1 | 1 | 1 | 1 | 1 | 1 | 1 | 1 |
| 939 | 26 | 2 | 150 | 42 | 6 | 1 | 1 | 1 | 4 | 1 | 1 | 1 | 1 | 1 | 1 | 1 | 1 | 1 |
| 940 | 26 | 2 | 151 | 40 | 8 | 1 | 1 | 1 | 5 | 4 | 1 | 1 | 1 | 1 | 1 | 1 | 1 | 1 |
| 941 | 26 | 2 | 153.5 | 44 | 2 | 2 | 1 | 1 | 4 | 4 | 1 | 1 | 1 | 1 | 1 | 1 | 1 | 1 |
| 942 | 26 | 2 | 155 | 51 | 2 | 1 | 1 | 2 | 4 | 3 | 1 | 1 | 1 | 1 | 1 | 1 | 1 | 1 |
| 943 | 26 | 2 | 155 | 47.5 | 8 | 2 | 1 | 1 | 4 | 1 | 1 | 1 | 1 | 1 | 2 | 1 | 1 | 1 |
| 944 | 26 | 2 | 155 | 43 | 3 | 1 | 1 | 1 | 5 | 3 | 1 | 1 | 2 | 1 | 2 | 1 | 1 | 1 |
| 945 | 26 | 2 | 156 | 60 | 4 | 2 | 1 | 1 | 6 | 4 | 1 | 1 | 1 | 1 | 1 | 1 | 1 | 1 |
| 946 | 26 | 2 | 156 | 52 | 2 | 1 | 1 | 1 | 5 | 4 | 1 | 1 | 1 | 1 | 1 | 1 | 1 | 1 |
| 947 | 26 | 2 | 156 | 47 | 8 | 1 | 1 | 1 | 5 | 1 | 1 | 1 | 1 | 1 | 1 | 1 | 1 | 1 |
| 948 | 26 | 2 | 156 | 45 | 8 | 2 | 1 | 3 | 2 | 4 | 1 | 1 | 1 | 1 | 1 | 1 | 3 | 1 |
| 949 | 26 | 2 | 157 | 60 | 1 | 2 | 1 | 1 | 5 | 3 | 1 | 1 | 2 | 1 | 1 | 1 | 1 | 1 |
| 950 | 26 | 2 | 158 | 47 | 8 | 2 | 1 | 1 | 3 | 2 | 1 | 1 | 1 | 1 | 1 | 1 | 1 | 1 |
| 951 | 26 | 2 | 158 | 47 | 4 | 2 | 1 | 1 | 5 | 3 | 1 | 1 | 2 | 1 | 1 | 1 | 1 | 1 |
| 952 | 26 | 2 | 158 | 42 | 8 | 1 | 1 | 1 | 4 | 3 | 1 | 1 | 1 | 1 | 1 | 1 | 1 | 1 |
| 953 | 26 | 2 | 159 | 56 | 8 | 2 | 1 | 1 | 3 | 1 | 1 | 1 | 1 | 1 | 1 | 1 | 1 | 1 |
| 954 | 26 | 2 | 160 | 57 | 3 | 2 | 1 | 1 | 5 | 3 | 1 | 1 | 1 | 1 | 1 | 1 | 1 | 1 |
| 955 | 26 | 2 | 160 | 52.5 | 8 | 2 | 1 | 1 | 5 | 3 | 1 | 1 | 2 | 1 | 1 | 1 | 1 | 1 |
| 956 | 26 | 2 | 160 | 48 | 8 | 1 | 1 | 1 | 6 | 4 | 1 | 1 | 1 | 1 | 1 | 1 | 1 | 1 |
| 957 | 26 | 2 | 160 | 47 | 8 | 2 | 1 | 1 | 5 | 3 | 1 | 1 | 1 | 1 | 1 | 1 | 1 | 1 |
| 958 | 26 | 2 | 161 | 53.2 | 8 | 2 | 1 | 1 | 3 | 4 | 1 | 1 | 2 | 1 | 2 | 1 | 1 | 1 |
| 959 | 26 | 2 | 162 | 51.5 | 4 | 2 | 1 | 1 | 5 | 3 | 1 | 1 | 2 | 1 | 1 | 1 | 1 | 1 |
| 960 | 26 | 2 | 163 | 49 | 3 | 2 | 1 | 1 | 5 | 1 | 1 | 1 | 2 | 1 | 1 | 1 | 1 | 1 |
| 961 | 26 | 2 | 163 | 48 | 4 | 2 | 1 | 1 | 4 | 3 | 1 | 1 | 2 | 1 | 1 | 1 | 1 | 1 |
| 962 | 26 | 2 | 165 | 51 | 8 | 2 | 1 | 1 | 6 | 4 | 1 | 1 | 2 | 1 | 1 | 1 | 1 | 1 |
| 963 | 26 | 2 | 169 | 60 | 8 | 2 | 1 | 1 | 4 | 4 | 1 | 1 | 1 | 1 | 1 | 1 | 1 | 1 |
| 964 | 26 | 2 | 172 | 54.6 | 8 | 2 | 1 | 1 | 4 | 4 | 1 | 1 | 1 | 1 | 1 | 1 | 1 | 1 |
| 965 | 26 | 1 | 160 | 69.5 | 4 | 2 | 1 | 1 | 5 | 4 | 1 | 1 | 2 | 1 | 1 | 1 | 3 | 1 |
| 966 | 26 | 1 | 167 | 58 | 2 | 1 | 1 | 2 | 5 | 4 | 1 | 1 | 1 | 1 | 1 | 1 | 1 | 1 |
| 967 | 26 | 1 | 169 | 50 | 2 | 1 | 1 | 1 | 5 | 2 | 1 | 1 | 1 | 1 | 1 | 1 | 1 | 1 |
| 968 | 26 | 1 | 174 | 68 | 2 | 1 | 1 | 1 | 5 | 4 | 1 | 1 | 2 | 1 | 1 | 1 | 1 | 1 |
| 969 | 26 | 1 | 180 | 75 | 8 | 1 | 1 | 1 | 5 | 2 | 1 | 1 | 2 | 1 | 1 | 1 | 1 | 1 |
| 970 | 27 | 2 | 142 | 93 | 3 | 1 | 1 | 1 | 5 | 4 | 1 | 1 | 2 | 1 | 1 | 1 | 1 | 1 |
| 971 | 27 | 2 | 150 | 55 | 2 | 2 | 1 | 1 | 5 | 3 | 1 | 1 | 1 | 1 | 1 | 1 | 1 | 1 |
| 972 | 27 | 2 | 153 | 41 | 8 | 2 | 1 | 1 | 4 | 3 | 1 | 1 | 1 | 1 | 1 | 1 | 1 | 1 |
| 973 | 27 | 2 | 155 | 49 | 2 | 2 | 1 | 2 | 5 | 1 | 1 | 1 | 2 | 1 | 1 | 1 | 1 | 1 |
| 974 | 27 | 2 | 155 | 44 | 2 | 2 | 1 | 1 | 5 | 4 | 1 | 1 | 1 | 1 | 1 | 1 | 1 | 1 |
| 975 | 27 | 2 | 156 | 53 | 2 | 1 | 1 | 2 | 5 | 3 | 1 | 1 | 1 | 1 | 1 | 1 | 1 | 1 |
| 976 | 27 | 2 | 156 | 48 | 4 | 2 | 1 | 1 | 4 | 4 | 1 | 1 | 1 | 1 | 1 | 1 | 1 | 1 |
| 977 | 27 | 2 | 156 | 45 | 2 | 2 | 1 | 1 | 5 | 3 | 1 | 1 | 2 | 1 | 1 | 1 | 1 | 1 |
| 978 | 27 | 2 | 158 | 55 | 6 | 2 | 1 | 1 | 5 | 2 | 1 | 1 | 2 | 1 | 1 | 1 | 1 | 1 |
| 979 | 27 | 2 | 158 | 53 | 2 | 2 | 1 | 2 | 5 | 3 | 1 | 1 | 1 | 1 | 1 | 1 | 1 | 1 |
| 980 | 27 | 2 | 158 | 46.8 | 8 | 2 | 1 | 1 | 5 | 1 | 1 | 1 | 1 | 1 | 1 | 1 | 1 | 1 |
| 981 | 27 | 2 | 158 | 45 | 3 | 2 | 1 | 1 | 5 | 3 | 1 | 1 | 2 | 1 | 1 | 1 | 1 | 1 |
| 982 | 27 | 2 | 159 | 60 | 4 | 2 | 1 | 1 | 4 | 3 | 1 | 1 | 1 | 1 | 1 | 1 | 1 | 1 |
| 983 | 27 | 2 | 159 | 41 | 2 | 2 | 1 | 1 | 5 | 3 | 1 | 1 | 1 | 1 | 1 | 1 | 1 | 1 |
| 984 | 27 | 2 | 160 | 55 | 4 | 2 | 1 | 1 | 5 | 4 | 1 | 1 | 2 | 1 | 1 | 1 | 1 | 1 |
| 985 | 27 | 2 | 160 | 55 | 4 | 2 | 1 | 1 | 3 | 3 | 1 | 1 | 1 | 1 | 1 | 1 | 1 | 1 |
| 986 | 27 | 2 | 160 | 53.5 | 8 | 1 | 1 | 1 | 5 | 3 | 1 | 1 | 1 | 1 | 1 | 1 | 1 | 1 |
| 987 | 27 | 2 | 160 | 45 | 2 | 1 | 1 | 2 | 5 | 2 | 1 | 1 | 2 | 1 | 1 | 1 | 1 | 1 |
| 988 | 27 | 2 | 161 | 44 | 2 | 1 | 1 | 1 | 4 | 4 | 1 | 1 | 2 | 1 | 1 | 1 | 1 | 1 |
| 989 | 27 | 2 | 162 | 55 | 4 | 2 | 1 | 1 | 5 | 2 | 1 | 1 | 1 | 1 | 2 | 1 | 1 | 1 |
| 990 | 27 | 2 | 162 | 53 | 8 | 2 | 1 | 1 | 5 | 2 | 1 | 1 | 1 | 1 | 2 | 1 | 1 | 1 |
| 991 | 27 | 2 | 163 | 50 | 2 | 2 | 1 | 1 | 5 | 3 | 1 | 1 | 1 | 2 | 1 | 1 | 1 | 2 |
| 992 | 27 | 2 | 165 | 50 | 8 | 2 | 1 | 1 | 5 | 4 | 1 | 1 | 2 | 1 | 1 | 1 | 1 | 1 |
| 993 | 27 | 2 | 167 | 53 | 3 | 2 | 1 | 1 | 5 | 3 | 1 | 1 | 1 | 1 | 1 | 1 | 1 | 1 |
| 994 | 27 | 2 | 168 | 70 | 8 | 2 | 1 | 1 | 4 | 4 | 1 | 1 | 1 | 1 | 1 | 1 | 1 | 1 |
| 995 | 27 | 2 | 168 | 47 | 4 | 1 | 1 | 1 | 5 | 3 | 1 | 1 | 2 | 1 | 1 | 1 | 1 | 1 |
| 996 | 27 | 2 | 169 | 68 | 2 | 1 | 1 | 1 | 5 | 2 | 1 | 1 | 2 | 1 | 2 | 1 | 1 | 1 |
| 997 | 27 | 2 | 170 | 64 | 4 | 2 | 1 | 1 | 5 | 3 | 1 | 1 | 2 | 1 | 1 | 1 | 1 | 1 |
| 998 | 27 | 2 | 170 | 43 | 8 | 2 | 1 | 1 | 5 | 4 | 1 | 1 | 1 | 1 | 1 | 1 | 1 | 1 |
| 999 | 27 | 1 | 166 | 59.5 | 2 | 1 | 1 | 1 | 5 | 2 | 1 | 1 | 1 | 1 | 1 | 1 | 1 | 1 |
| 1000 | 28 | 2 | 150 | 50 | 4 | 2 | 1 | 1 | 5 | 2 | 1 | 1 | 1 | 1 | 1 | 1 | 1 | 1 |
| 1001 | 28 | 2 | 150 | 44 | 4 | 2 | 1 | 1 | 5 | 3 | 1 | 1 | 2 | 1 | 1 | 1 | 1 | 1 |
| 1002 | 28 | 2 | 152 | 42 | 3 | 2 | 1 | 1 | 4 | 3 | 1 | 1 | 2 | 1 | 1 | 1 | 1 | 1 |
| 1003 | 28 | 2 | 153 | 60 | 2 | 2 | 1 | 1 | 5 | 2 | 1 | 1 | 2 | 1 | 1 | 1 | 1 | 1 |
| 1004 | 28 | 2 | 153 | 42 | 1 | 2 | 1 | 1 | 5 | 3 | 1 | 1 | 1 | 1 | 1 | 1 | 1 | 1 |
| 1005 | 28 | 2 | 155 | 46 | 1 | 1 | 1 | 1 | 5 | 2 | 1 | 1 | 2 | 1 | 1 | 1 | 1 | 1 |
| 1006 | 28 | 2 | 155 | 45 | 1 | 2 | 1 | 1 | 5 | 1 | 1 | 1 | 2 | 1 | 1 | 1 | 1 | 1 |
| 1007 | 28 | 2 | 156 | 58 | 1 | 2 | 1 | 1 | 4 | 4 | 1 | 1 | 2 | 1 | 1 | 1 | 1 | 1 |
| 1008 | 28 | 2 | 156 | 48 | 3 | 2 | 1 | 1 | 5 | 3 | 1 | 1 | 2 | 1 | 1 | 1 | 1 | 1 |
| 1009 | 28 | 2 | 156 | 44 | 4 | 2 | 1 | 1 | 5 | 3 | 1 | 1 | 1 | 1 | 1 | 1 | 1 | 1 |
| 1010 | 28 | 2 | 157 | 48 | 8 | 2 | 1 | 1 | 5 | 2 | 1 | 1 | 1 | 1 | 1 | 1 | 1 | 1 |
| 1011 | 28 | 2 | 157 | 46 | 4 | 2 | 1 | 1 | 4 | 4 | 1 | 1 | 1 | 1 | 1 | 1 | 1 | 1 |
| 1012 | 28 | 2 | 159 | 44 | 4 | 2 | 1 | 1 | 4 | 2 | 1 | 1 | 1 | 1 | 1 | 1 | 1 | 1 |
| 1013 | 28 | 2 | 160 | 58 | 4 | 2 | 1 | 1 | 4 | 4 | 1 | 1 | 1 | 1 | 1 | 1 | 1 | 1 |
| 1014 | 28 | 2 | 160 | 54.5 | 8 | 2 | 1 | 1 | 4 | 3 | 1 | 1 | 1 | 1 | 1 | 1 | 1 | 1 |
| 1015 | 28 | 2 | 160 | 50 | 3 | 1 | 1 | 1 | 5 | 2 | 1 | 1 | 1 | 1 | 1 | 1 | 1 | 1 |
| 1016 | 28 | 2 | 160 | 50 | 2 | 2 | 1 | 1 | 5 | 2 | 1 | 1 | 1 | 1 | 1 | 1 | 1 | 1 |
| 1017 | 28 | 2 | 160 | 48 | 2 | 1 | 1 | 1 | 5 | 3 | 1 | 1 | 1 | 1 | 1 | 1 | 1 | 1 |
| 1018 | 28 | 2 | 160 | 45 | 8 | 1 | 1 | 2 | 5 | 2 | 1 | 1 | 2 | 1 | 1 | 1 | 1 | 1 |
| 1019 | 28 | 2 | 161 | 57 | 4 | 2 | 1 | 1 | 5 | 2 | 1 | 2 | 2 | 1 | 1 | 1 | 1 | 1 |
| 1020 | 28 | 2 | 162 | 85 | 8 | 2 | 1 | 1 | 5 | 4 | 1 | 1 | 2 | 1 | 1 | 1 | 3 | 1 |
| 1021 | 28 | 2 | 162 | 57.5 | 8 | 2 | 1 | 1 | 5 | 4 | 1 | 1 | 2 | 1 | 2 | 1 | 1 | 1 |
| 1022 | 28 | 2 | 162.5 | 65 | 4 | 2 | 1 | 1 | 5 | 2 | 1 | 1 | 2 | 1 | 1 | 1 | 1 | 1 |
| 1023 | 28 | 2 | 163 | 53.5 | 8 | 2 | 1 | 1 | 3 | 4 | 1 | 1 | 1 | 1 | 1 | 1 | 1 | 1 |
| 1024 | 28 | 2 | 163 | 52 | 3 | 2 | 1 | 2 | 5 | 3 | 1 | 1 | 1 | 1 | 1 | 1 | 1 | 1 |
| 1025 | 28 | 2 | 163 | 50 | 2 | 1 | 1 | 1 | 5 | 3 | 1 | 1 | 2 | 1 | 1 | 1 | 1 | 1 |
| 1026 | 28 | 2 | 165 | 71.5 | 3 | 2 | 1 | 1 | 4 | 3 | 1 | 1 | 1 | 1 | 1 | 1 | 2 | 1 |
| 1027 | 28 | 2 | 170 | 58 | 2 | 2 | 1 | 1 | 5 | 2 | 1 | 1 | 1 | 1 | 1 | 1 | 1 | 1 |
| 1028 | 28 | 1 | 162 | 75 | 2 | 1 | 1 | 1 | 2 | 3 | 1 | 1 | 1 | 1 | 1 | 1 | 3 | 1 |
| 1029 | 28 | 1 | 164 | 52 | 2 | 1 | 1 | 2 | 5 | 3 | 3 | 1 | 1 | 1 | 1 | 1 | 1 | 1 |
| 1030 | 28 | 1 | 170 | 60 | 2 | 2 | 1 | 2 | 5 | 3 | 1 | 1 | 2 | 1 | 1 | 1 | 2 | 1 |
| 1031 | 28 | 1 | 170.5 | 75 | 4 | 1 | 1 | 1 | 5 | 4 | 1 | 1 | 1 | 1 | 1 | 1 | 1 | 1 |
| 1032 | 28 | 1 | 172 | 60 | 8 | 2 | 1 | 1 | 3 | 4 | 1 | 1 | 2 | 1 | 1 | 1 | 1 | 1 |
| 1033 | 28 | 1 | 175 | 76 | 8 | 1 | 1 | 2 | 5 | 2 | 1 | 1 | 2 | 1 | 1 | 1 | 3 | 1 |
| 1034 | 28 | 1 | 179 | 76 | 3 | 1 | 1 | 1 | 5 | 2 | 1 | 1 | 2 | 1 | 1 | 1 | 1 | 1 |
| 1035 | 28 | 1 | 180 | 56 | 8 | 1 | 1 | 1 | 4 | 3 | 1 | 1 | 2 | 1 | 1 | 1 | 1 | 1 |
| 1036 | 29 | 2 | 143 | 46 | 4 | 1 | 2 | 1 | 4 | 2 | 1 | 1 | 1 | 1 | 1 | 1 | 1 | 1 |
| 1037 | 29 | 2 | 148 | 61 | 8 | 2 | 1 | 1 | 5 | 3 | 1 | 1 | 1 | 2 | 1 | 1 | 1 | 2 |
| 1038 | 29 | 2 | 150 | 52.5 | 4 | 1 | 1 | 1 | 4 | 1 | 1 | 1 | 2 | 1 | 1 | 1 | 1 | 1 |
| 1039 | 29 | 2 | 150 | 48 | 8 | 2 | 1 | 1 | 3 | 2 | 1 | 1 | 2 | 1 | 1 | 1 | 1 | 1 |
| 1040 | 29 | 2 | 153 | 60 | 8 | 2 | 1 | 3 | 5 | 2 | 1 | 1 | 2 | 1 | 1 | 1 | 1 | 1 |
| 1041 | 29 | 2 | 153 | 53.8 | 3 | 2 | 1 | 1 | 5 | 3 | 1 | 1 | 2 | 1 | 1 | 1 | 1 | 1 |
| 1042 | 29 | 2 | 155 | 58 | 8 | 2 | 1 | 1 | 5 | 3 | 1 | 1 | 1 | 1 | 1 | 1 | 1 | 1 |
| 1043 | 29 | 2 | 155 | 49 | 8 | 2 | 1 | 1 | 4 | 3 | 1 | 1 | 1 | 1 | 1 | 1 | 1 | 1 |
| 1044 | 29 | 2 | 155 | 49 | 2 | 2 | 1 | 1 | 4 | 4 | 1 | 1 | 1 | 1 | 1 | 1 | 1 | 1 |
| 1045 | 29 | 2 | 155 | 45 | 4 | 2 | 1 | 1 | 5 | 3 | 1 | 1 | 1 | 1 | 1 | 1 | 1 | 1 |
| 1046 | 29 | 2 | 157 | 54 | 3 | 2 | 1 | 1 | 5 | 3 | 1 | 1 | 1 | 1 | 1 | 1 | 1 | 1 |
| 1047 | 29 | 2 | 157 | 52.5 | 8 | 2 | 1 | 1 | 5 | 4 | 1 | 1 | 1 | 1 | 1 | 1 | 1 | 1 |
| 1048 | 29 | 2 | 157 | 40.5 | 2 | 2 | 1 | 1 | 5 | 2 | 1 | 1 | 1 | 1 | 1 | 1 | 1 | 1 |
| 1049 | 29 | 2 | 158 | 60 | 8 | 2 | 1 | 1 | 3 | 4 | 1 | 1 | 2 | 1 | 1 | 1 | 1 | 1 |
| 1050 | 29 | 2 | 158 | 50 | 2 | 2 | 1 | 1 | 5 | 4 | 1 | 1 | 1 | 1 | 2 | 1 | 1 | 1 |
| 1051 | 29 | 2 | 158 | 50 | 2 | 2 | 1 | 1 | 5 | 4 | 1 | 1 | 1 | 1 | 1 | 1 | 1 | 1 |
| 1052 | 29 | 2 | 158 | 50 | 2 | 2 | 1 | 1 | 5 | 4 | 1 | 1 | 1 | 1 | 1 | 1 | 1 | 1 |
| 1053 | 29 | 2 | 158 | 42 | 3 | 2 | 1 | 1 | 5 | 3 | 1 | 1 | 2 | 1 | 1 | 1 | 1 | 1 |
| 1054 | 29 | 2 | 159 | 68 | 8 | 1 | 1 | 2 | 5 | 3 | 1 | 2 | 2 | 1 | 1 | 1 | 1 | 1 |
| 1055 | 29 | 2 | 160 | 71 | 2 | 2 | 1 | 1 | 5 | 3 | 1 | 1 | 1 | 1 | 1 | 1 | 1 | 1 |
| 1056 | 29 | 2 | 160 | 66 | 8 | 2 | 2 | 1 | 5 | 4 | 1 | 1 | 1 | 1 | 2 | 1 | 1 | 1 |
| 1057 | 29 | 2 | 160 | 63 | 4 | 2 | 2 | 1 | 4 | 4 | 1 | 1 | 1 | 1 | 1 | 1 | 1 | 1 |
| 1058 | 29 | 2 | 160 | 55 | 8 | 1 | 1 | 2 | 5 | 3 | 1 | 1 | 1 | 1 | 1 | 1 | 3 | 1 |
| 1059 | 29 | 2 | 160 | 53.5 | 4 | 1 | 1 | 1 | 5 | 2 | 1 | 1 | 1 | 1 | 1 | 1 | 1 | 1 |
| 1060 | 29 | 2 | 160 | 52 | 2 | 2 | 1 | 1 | 5 | 3 | 1 | 1 | 2 | 1 | 1 | 1 | 1 | 1 |
| 1061 | 29 | 2 | 160 | 50 | 4 | 2 | 1 | 1 | 5 | 4 | 1 | 1 | 1 | 1 | 1 | 1 | 1 | 1 |
| 1062 | 29 | 2 | 161 | 55 | 2 | 1 | 1 | 2 | 5 | 3 | 1 | 1 | 2 | 1 | 2 | 1 | 1 | 1 |
| 1063 | 29 | 2 | 161 | 51 | 2 | 2 | 1 | 2 | 5 | 2 | 1 | 1 | 2 | 1 | 1 | 1 | 1 | 1 |
| 1064 | 29 | 2 | 162 | 73 | 3 | 2 | 1 | 1 | 5 | 3 | 1 | 1 | 1 | 1 | 1 | 1 | 1 | 1 |
| 1065 | 29 | 2 | 162 | 55 | 3 | 2 | 1 | 1 | 5 | 2 | 1 | 1 | 2 | 1 | 1 | 1 | 1 | 1 |
| 1066 | 29 | 2 | 162 | 49 | 1 | 2 | 1 | 1 | 5 | 3 | 1 | 1 | 2 | 1 | 1 | 1 | 1 | 1 |
| 1067 | 29 | 2 | 162 | 46 | 4 | 2 | 1 | 1 | 5 | 4 | 1 | 1 | 1 | 1 | 2 | 1 | 1 | 1 |
| 1068 | 29 | 2 | 163 | 70 | 1 | 2 | 1 | 1 | 5 | 1 | 1 | 1 | 2 | 1 | 1 | 1 | 1 | 1 |
| 1069 | 29 | 2 | 163 | 52 | 4 | 2 | 1 | 1 | 5 | 3 | 1 | 1 | 2 | 1 | 1 | 1 | 1 | 1 |
| 1070 | 29 | 2 | 163 | 45 | 8 | 2 | 1 | 1 | 3 | 4 | 1 | 1 | 1 | 1 | 1 | 1 | 1 | 1 |
| 1071 | 29 | 2 | 165 | 81.5 | 8 | 4 | 1 | 1 | 5 | 4 | 1 | 1 | 2 | 1 | 1 | 1 | 3 | 1 |
| 1072 | 29 | 2 | 165 | 60 | 8 | 2 | 2 | 1 | 3 | 4 | 1 | 1 | 2 | 1 | 1 | 1 | 1 | 1 |
| 1073 | 29 | 2 | 165 | 60 | 8 | 2 | 1 | 1 | 5 | 1 | 1 | 1 | 2 | 1 | 1 | 1 | 1 | 1 |
| 1074 | 29 | 2 | 165 | 50 | 8 | 2 | 1 | 1 | 4 | 3 | 1 | 1 | 1 | 1 | 1 | 1 | 1 | 1 |
| 1075 | 29 | 2 | 168 | 65 | 3 | 2 | 1 | 1 | 5 | 2 | 1 | 1 | 2 | 1 | 1 | 1 | 1 | 1 |
| 1076 | 29 | 2 | 169 | 69 | 8 | 2 | 1 | 1 | 5 | 3 | 1 | 1 | 1 | 1 | 1 | 1 | 1 | 1 |
| 1077 | 29 | 2 | 170 | 60 | 8 | 2 | 2 | 1 | 4 | 3 | 1 | 1 | 1 | 1 | 1 | 1 | 1 | 1 |
| 1078 | 29 | 2 | 170 | 55 | 4 | 2 | 1 | 1 | 5 | 2 | 1 | 1 | 2 | 1 | 1 | 1 | 1 | 1 |
| 1079 | 29 | 1 | 166 | 55 | 4 | 2 | 1 | 1 | 5 | 4 | 1 | 1 | 1 | 1 | 1 | 1 | 1 | 1 |
| 1080 | 29 | 1 | 169 | 80 | 8 | 1 | 1 | 1 | 5 | 4 | 1 | 1 | 2 | 1 | 1 | 1 | 1 | 1 |
| 1081 | 29 | 1 | 170 | 52 | 8 | 2 | 1 | 1 | 4 | 4 | 1 | 1 | 1 | 1 | 1 | 1 | 1 | 1 |
| 1082 | 29 | 1 | 171 | 68 | 8 | 2 | 1 | 1 | 4 | 4 | 1 | 1 | 1 | 1 | 1 | 1 | 1 | 1 |
| 1083 | 29 | 1 | 173 | 73 | 8 | 2 | 2 | 1 | 4 | 2 | 1 | 1 | 1 | 1 | 1 | 1 | 1 | 1 |
| 1084 | 29 | 1 | 179 | 81 | 2 | 1 | 1 | 1 | 5 | 2 | 1 | 1 | 2 | 1 | 1 | 1 | 1 | 1 |
| 1085 | 29 | 1 | 187 | 88.8 | 4 | 1 | 1 | 1 | 5 | 2 | 1 | 1 | 1 | 1 | 1 | 1 | 1 | 1 |
| 1086 | 30 | 2 | 150 | 79 | 6 | 2 | 1 | 1 | 5 | 4 | 1 | 1 | 2 | 1 | 2 | 1 | 1 | 1 |
| 1087 | 30 | 2 | 150 | 58 | 8 | 2 | 1 | 1 | 6 | 4 | 1 | 1 | 1 | 1 | 1 | 1 | 1 | 1 |
| 1088 | 30 | 2 | 152 | 52.5 | 8 | 2 | 1 | 1 | 5 | 3 | 1 | 1 | 1 | 1 | 1 | 1 | 1 | 1 |
| 1089 | 30 | 2 | 153 | 54 | 3 | 2 | 1 | 1 | 5 | 1 | 2 | 1 | 2 | 1 | 2 | 1 | 1 | 1 |
| 1090 | 30 | 2 | 153 | 48 | 4 | 2 | 1 | 1 | 4 | 3 | 1 | 1 | 2 | 1 | 1 | 1 | 1 | 1 |
| 1091 | 30 | 2 | 153 | 46 | 3 | 2 | 1 | 1 | 5 | 3 | 1 | 1 | 1 | 1 | 1 | 1 | 1 | 1 |
| 1092 | 30 | 2 | 153 | 45 | 2 | 2 | 1 | 1 | 5 | 3 | 1 | 1 | 2 | 1 | 1 | 1 | 1 | 2 |
| 1093 | 30 | 2 | 155 | 50 | 8 | 2 | 1 | 1 | 3 | 3 | 1 | 1 | 2 | 1 | 1 | 1 | 1 | 2 |
| 1094 | 30 | 2 | 155.5 | 53.5 | 8 | 2 | 1 | 1 | 3 | 1 | 1 | 1 | 1 | 1 | 1 | 1 | 1 | 2 |
| 1095 | 30 | 2 | 156 | 57.5 | 4 | 2 | 1 | 1 | 5 | 4 | 1 | 1 | 1 | 1 | 1 | 1 | 1 | 1 |
| 1096 | 30 | 2 | 156 | 53 | 2 | 2 | 1 | 2 | 5 | 1 | 1 | 1 | 1 | 1 | 1 | 1 | 1 | 1 |
| 1097 | 30 | 2 | 156 | 48 | 2 | 2 | 1 | 2 | 5 | 3 | 1 | 1 | 1 | 1 | 1 | 1 | 1 | 1 |
| 1098 | 30 | 2 | 156 | 47 | 8 | 1 | 1 | 1 | 5 | 2 | 1 | 1 | 1 | 1 | 1 | 1 | 1 | 1 |
| 1099 | 30 | 2 | 157 | 45 | 2 | 1 | 1 | 1 | 5 | 2 | 1 | 1 | 2 | 1 | 1 | 1 | 1 | 1 |
| 1100 | 30 | 2 | 158 | 57.5 | 8 | 2 | 1 | 1 | 4 | 4 | 1 | 1 | 1 | 1 | 1 | 1 | 1 | 1 |
| 1101 | 30 | 2 | 158 | 50 | 3 | 2 | 1 | 1 | 5 | 2 | 1 | 1 | 2 | 1 | 1 | 1 | 1 | 1 |
| 1102 | 30 | 2 | 159 | 48 | 3 | 2 | 1 | 1 | 5 | 3 | 1 | 1 | 2 | 1 | 1 | 1 | 1 | 1 |
| 1103 | 30 | 2 | 160 | 68 | 3 | 2 | 1 | 1 | 5 | 3 | 1 | 1 | 2 | 1 | 1 | 1 | 1 | 1 |
| 1104 | 30 | 2 | 160 | 65 | 4 | 2 | 1 | 1 | 5 | 2 | 1 | 1 | 1 | 1 | 2 | 1 | 1 | 1 |
| 1105 | 30 | 2 | 160 | 55 | 8 | 2 | 1 | 1 | 5 | 4 | 1 | 1 | 2 | 1 | 1 | 1 | 1 | 1 |
| 1106 | 30 | 2 | 160 | 53 | 3 | 2 | 1 | 2 | 3 | 3 | 1 | 1 | 1 | 1 | 2 | 1 | 3 | 1 |
| 1107 | 30 | 2 | 160 | 50 | 8 | 2 | 1 | 1 | 5 | 1 | 1 | 1 | 1 | 1 | 1 | 1 | 1 | 1 |
| 1108 | 30 | 2 | 161 | 57 | 8 | 2 | 1 | 1 | 5 | 3 | 1 | 1 | 2 | 1 | 1 | 1 | 1 | 1 |
| 1109 | 30 | 2 | 161 | 47 | 8 | 2 | 1 | 1 | 6 | 4 | 1 | 1 | 2 | 1 | 1 | 1 | 1 | 1 |
| 1110 | 30 | 2 | 162 | 50 | 1 | 2 | 1 | 1 | 5 | 3 | 1 | 1 | 2 | 1 | 1 | 1 | 1 | 1 |
| 1111 | 30 | 2 | 162 | 45 | 1 | 2 | 1 | 1 | 5 | 1 | 1 | 1 | 1 | 1 | 1 | 1 | 1 | 1 |
| 1112 | 30 | 2 | 163 | 59 | 4 | 1 | 1 | 1 | 5 | 2 | 1 | 1 | 1 | 1 | 1 | 1 | 1 | 1 |
| 1113 | 30 | 2 | 163 | 56 | 8 | 2 | 1 | 2 | 5 | 3 | 1 | 1 | 2 | 1 | 1 | 1 | 1 | 1 |
| 1114 | 30 | 2 | 164 | 55 | 2 | 2 | 1 | 1 | 3 | 1 | 1 | 1 | 2 | 1 | 1 | 1 | 1 | 1 |
| 1115 | 30 | 2 | 165 | 60 | 8 | 2 | 1 | 2 | 3 | 3 | 1 | 1 | 2 | 1 | 1 | 1 | 1 | 1 |
| 1116 | 30 | 2 | 165 | 57 | 2 | 2 | 1 | 1 | 5 | 3 | 1 | 1 | 2 | 1 | 2 | 1 | 1 | 1 |
| 1117 | 30 | 2 | 165 | 55 | 8 | 2 | 1 | 1 | 5 | 2 | 1 | 1 | 1 | 1 | 1 | 1 | 1 | 2 |
| 1118 | 30 | 2 | 165 | 52.5 | 4 | 2 | 1 | 1 | 5 | 2 | 1 | 1 | 2 | 1 | 1 | 1 | 1 | 1 |
| 1119 | 30 | 2 | 166 | 58.5 | 4 | 1 | 1 | 1 | 5 | 3 | 1 | 1 | 2 | 1 | 1 | 1 | 1 | 1 |
| 1120 | 30 | 1 | 155 | 52 | 8 | 2 | 1 | 1 | 2 | 4 | 1 | 1 | 1 | 1 | 1 | 1 | 1 | 1 |
| 1121 | 30 | 1 | 161 | 55 | 1 | 1 | 1 | 1 | 5 | 4 | 1 | 1 | 1 | 1 | 1 | 1 | 1 | 1 |
| 1122 | 30 | 1 | 164 | 65 | 2 | 2 | 1 | 1 | 5 | 3 | 1 | 1 | 2 | 1 | 1 | 1 | 1 | 1 |
| 1123 | 30 | 1 | 166.5 | 80 | 3 | 2 | 1 | 1 | 5 | 2 | 1 | 1 | 2 | 1 | 2 | 1 | 1 | 1 |
| 1124 | 30 | 1 | 167 | 70 | 8 | 2 | 1 | 1 | 5 | 2 | 1 | 1 | 2 | 1 | 2 | 1 | 1 | 1 |
| 1125 | 30 | 1 | 170 | 80 | 3 | 2 | 1 | 1 | 5 | 4 | 1 | 1 | 1 | 1 | 1 | 1 | 1 | 1 |
| 1126 | 30 | 1 | 170 | 78 | 4 | 2 | 1 | 1 | 5 | 4 | 1 | 1 | 2 | 1 | 1 | 1 | 3 | 1 |
| 1127 | 30 | 1 | 170 | 68 | 8 | 2 | 1 | 1 | 5 | 1 | 1 | 1 | 1 | 1 | 1 | 1 | 1 | 1 |
| 1128 | 30 | 1 | 172 | 67 | 2 | 2 | 1 | 1 | 5 | 2 | 1 | 1 | 2 | 1 | 1 | 1 | 1 | 1 |
| 1129 | 30 | 1 | 173 | 62 | 2 | 2 | 1 | 1 | 5 | 3 | 2 | 1 | 2 | 1 | 1 | 1 | 1 | 1 |
| 1130 | 30 | 1 | 178 | 81 | 4 | 2 | 1 | 2 | 5 | 2 | 1 | 1 | 2 | 1 | 1 | 1 | 3 | 1 |
| 1131 | 31 | 2 | 148 | 49 | 3 | 1 | 1 | 1 | 5 | 1 | 1 | 1 | 1 | 1 | 1 | 1 | 1 | 1 |
| 1132 | 31 | 2 | 148 | 40 | 3 | 2 | 1 | 1 | 5 | 4 | 1 | 1 | 2 | 1 | 1 | 1 | 1 | 1 |
| 1133 | 31 | 2 | 150 | 52 | 8 | 2 | 1 | 1 | 4 | 4 | 1 | 1 | 2 | 1 | 1 | 1 | 1 | 1 |
| 1134 | 31 | 2 | 150 | 43 | 4 | 2 | 1 | 1 | 5 | 2 | 1 | 1 | 1 | 1 | 1 | 1 | 1 | 1 |
| 1135 | 31 | 2 | 151 | 50 | 8 | 2 | 1 | 1 | 5 | 3 | 1 | 1 | 1 | 2 | 2 | 1 | 1 | 2 |
| 1136 | 31 | 2 | 153 | 50 | 2 | 2 | 2 | 1 | 5 | 4 | 1 | 1 | 2 | 1 | 1 | 1 | 1 | 2 |
| 1137 | 31 | 2 | 154 | 47.5 | 3 | 2 | 1 | 1 | 5 | 2 | 1 | 1 | 2 | 1 | 1 | 1 | 1 | 1 |
| 1138 | 31 | 2 | 155 | 60 | 2 | 2 | 1 | 1 | 5 | 3 | 1 | 1 | 1 | 1 | 1 | 1 | 1 | 1 |
| 1139 | 31 | 2 | 155 | 47 | 2 | 4 | 1 | 1 | 5 | 2 | 1 | 1 | 2 | 1 | 1 | 1 | 1 | 1 |
| 1140 | 31 | 2 | 155 | 44 | 4 | 1 | 1 | 1 | 5 | 4 | 1 | 1 | 1 | 1 | 1 | 1 | 1 | 1 |
| 1141 | 31 | 2 | 155 | 40 | 8 | 1 | 1 | 1 | 4 | 3 | 1 | 1 | 2 | 1 | 1 | 1 | 1 | 1 |
| 1142 | 31 | 2 | 156 | 55 | 3 | 2 | 1 | 1 | 5 | 3 | 1 | 1 | 2 | 1 | 1 | 1 | 1 | 1 |
| 1143 | 31 | 2 | 156 | 52 | 2 | 2 | 1 | 1 | 5 | 2 | 1 | 1 | 1 | 1 | 2 | 1 | 1 | 1 |
| 1144 | 31 | 2 | 156 | 49 | 4 | 2 | 1 | 1 | 5 | 2 | 1 | 1 | 1 | 1 | 1 | 1 | 1 | 1 |
| 1145 | 31 | 2 | 156 | 48 | 8 | 2 | 1 | 1 | 3 | 1 | 1 | 1 | 1 | 1 | 1 | 1 | 1 | 1 |
| 1146 | 31 | 2 | 156 | 45 | 4 | 2 | 1 | 1 | 5 | 4 | 1 | 1 | 2 | 1 | 1 | 1 | 1 | 1 |
| 1147 | 31 | 2 | 156 | 45 | 3 | 2 | 1 | 1 | 5 | 3 | 1 | 1 | 2 | 1 | 1 | 1 | 1 | 1 |
| 1148 | 31 | 2 | 157 | 50 | 2 | 2 | 1 | 1 | 5 | 3 | 1 | 1 | 2 | 1 | 1 | 1 | 1 | 1 |
| 1149 | 31 | 2 | 158 | 66.5 | 3 | 2 | 1 | 1 | 5 | 2 | 1 | 1 | 1 | 2 | 1 | 1 | 1 | 2 |
| 1150 | 31 | 2 | 158 | 55 | 1 | 2 | 1 | 1 | 5 | 3 | 1 | 1 | 1 | 1 | 2 | 1 | 1 | 1 |
| 1151 | 31 | 2 | 158 | 52 | 2 | 2 | 1 | 1 | 5 | 1 | 1 | 1 | 2 | 1 | 1 | 1 | 1 | 1 |
| 1152 | 31 | 2 | 158 | 51 | 8 | 2 | 1 | 1 | 5 | 4 | 1 | 1 | 2 | 1 | 1 | 1 | 1 | 1 |
| 1153 | 31 | 2 | 158 | 42 | 4 | 2 | 1 | 1 | 5 | 2 | 1 | 1 | 1 | 1 | 1 | 1 | 1 | 1 |
| 1154 | 31 | 2 | 159 | 56 | 1 | 2 | 1 | 1 | 5 | 3 | 1 | 1 | 2 | 1 | 1 | 1 | 1 | 1 |
| 1155 | 31 | 2 | 159 | 55 | 2 | 2 | 1 | 1 | 5 | 4 | 1 | 1 | 2 | 1 | 1 | 1 | 1 | 1 |
| 1156 | 31 | 2 | 159 | 50 | 2 | 2 | 1 | 1 | 5 | 4 | 1 | 1 | 2 | 1 | 1 | 1 | 1 | 1 |
| 1157 | 31 | 2 | 160 | 62.5 | 3 | 2 | 1 | 1 | 5 | 3 | 2 | 1 | 2 | 1 | 1 | 1 | 1 | 1 |
| 1158 | 31 | 2 | 160 | 58 | 8 | 2 | 1 | 1 | 4 | 2 | 1 | 1 | 1 | 1 | 1 | 1 | 1 | 1 |
| 1159 | 31 | 2 | 160 | 50 | 8 | 2 | 1 | 1 | 4 | 2 | 1 | 1 | 1 | 1 | 1 | 1 | 1 | 1 |
| 1160 | 31 | 2 | 160 | 49.5 | 8 | 2 | 1 | 1 | 5 | 2 | 1 | 1 | 1 | 1 | 1 | 1 | 1 | 1 |
| 1161 | 31 | 2 | 161 | 46 | 1 | 2 | 1 | 1 | 5 | 3 | 1 | 1 | 1 | 1 | 1 | 1 | 1 | 1 |
| 1162 | 31 | 2 | 162 | 59 | 2 | 2 | 1 | 1 | 5 | 3 | 1 | 1 | 1 | 1 | 1 | 1 | 1 | 1 |
| 1163 | 31 | 2 | 162 | 56 | 3 | 2 | 1 | 1 | 5 | 4 | 1 | 1 | 1 | 1 | 1 | 1 | 1 | 1 |
| 1164 | 31 | 2 | 163 | 50 | 4 | 2 | 1 | 1 | 4 | 4 | 1 | 1 | 1 | 1 | 2 | 1 | 1 | 1 |
| 1165 | 31 | 2 | 164 | 58 | 3 | 2 | 1 | 1 | 5 | 4 | 1 | 1 | 2 | 1 | 1 | 1 | 1 | 1 |
| 1166 | 31 | 2 | 165 | 80 | 3 | 2 | 1 | 1 | 5 | 3 | 1 | 1 | 2 | 1 | 1 | 1 | 1 | 1 |
| 1167 | 31 | 2 | 165 | 60 | 4 | 2 | 1 | 1 | 5 | 4 | 1 | 1 | 2 | 1 | 1 | 1 | 1 | 1 |
| 1168 | 31 | 2 | 165 | 60 | 4 | 2 | 1 | 1 | 5 | 3 | 1 | 1 | 2 | 1 | 1 | 1 | 1 | 1 |
| 1169 | 31 | 2 | 165 | 58 | 4 | 2 | 1 | 1 | 4 | 4 | 1 | 1 | 1 | 1 | 1 | 1 | 1 | 1 |
| 1170 | 31 | 2 | 165 | 49 | 4 | 2 | 1 | 1 | 5 | 3 | 1 | 1 | 1 | 1 | 1 | 1 | 1 | 1 |
| 1171 | 31 | 2 | 165 | 44 | 3 | 2 | 1 | 1 | 5 | 3 | 1 | 1 | 2 | 1 | 1 | 1 | 1 | 1 |
| 1172 | 31 | 2 | 166 | 55 | 3 | 2 | 1 | 1 | 5 | 3 | 1 | 1 | 1 | 1 | 1 | 1 | 1 | 1 |
| 1173 | 31 | 2 | 167 | 50 | 8 | 2 | 1 | 1 | 5 | 2 | 2 | 1 | 1 | 1 | 1 | 1 | 1 | 1 |
| 1174 | 31 | 2 | 168 | 65 | 4 | 2 | 1 | 1 | 5 | 1 | 1 | 1 | 1 | 1 | 1 | 1 | 1 | 1 |
| 1175 | 31 | 1 | 163 | 58.1 | 8 | 1 | 1 | 1 | 4 | 1 | 1 | 1 | 2 | 1 | 1 | 1 | 3 | 1 |
| 1176 | 31 | 1 | 175 | 72 | 1 | 2 | 1 | 1 | 5 | 2 | 1 | 1 | 2 | 1 | 1 | 1 | 1 | 1 |
| 1177 | 31 | 1 | 175 | 70 | 6 | 1 | 1 | 1 | 4 | 1 | 1 | 1 | 1 | 1 | 1 | 1 | 1 | 1 |
| 1178 | 31 | 1 | 175 | 70 | 1 | 2 | 1 | 1 | 1 | 3 | 1 | 1 | 2 | 1 | 1 | 1 | 1 | 1 |
| 1179 | 31 | 1 | 175 | 61 | 8 | 2 | 1 | 1 | 5 | 2 | 1 | 1 | 1 | 2 | 1 | 1 | 1 | 2 |
| 1180 | 32 | 2 | 152 | 58 | 8 | 2 | 1 | 1 | 3 | 3 | 1 | 1 | 1 | 1 | 1 | 1 | 1 | 1 |
| 1181 | 32 | 2 | 152 | 48 | 4 | 2 | 1 | 1 | 4 | 4 | 1 | 1 | 2 | 1 | 1 | 1 | 1 | 1 |
| 1182 | 32 | 2 | 154 | 76 | 8 | 2 | 1 | 1 | 4 | 3 | 1 | 1 | 1 | 1 | 1 | 1 | 1 | 1 |
| 1183 | 32 | 2 | 154 | 65 | 8 | 2 | 1 | 1 | 5 | 2 | 1 | 1 | 2 | 1 | 1 | 1 | 1 | 1 |
| 1184 | 32 | 2 | 154 | 52 | 2 | 1 | 1 | 1 | 5 | 3 | 1 | 1 | 2 | 1 | 1 | 1 | 1 | 1 |
| 1185 | 32 | 2 | 154 | 44 | 8 | 2 | 1 | 1 | 3 | 1 | 1 | 1 | 1 | 1 | 1 | 1 | 1 | 1 |
| 1186 | 32 | 2 | 155 | 48 | 4 | 2 | 1 | 1 | 5 | 3 | 1 | 1 | 2 | 1 | 1 | 1 | 1 | 1 |
| 1187 | 32 | 2 | 155 | 45 | 4 | 2 | 1 | 2 | 5 | 4 | 1 | 1 | 2 | 1 | 2 | 1 | 2 | 1 |
| 1188 | 32 | 2 | 157 | 64 | 2 | 2 | 1 | 1 | 5 | 4 | 2 | 1 | 1 | 2 | 1 | 1 | 1 | 2 |
| 1189 | 32 | 2 | 157 | 57 | 8 | 2 | 1 | 1 | 5 | 2 | 1 | 1 | 2 | 1 | 1 | 1 | 1 | 1 |
| 1190 | 32 | 2 | 158 | 54 | 8 | 2 | 1 | 1 | 4 | 4 | 1 | 1 | 1 | 1 | 1 | 1 | 1 | 1 |
| 1191 | 32 | 2 | 158 | 52 | 3 | 2 | 1 | 1 | 5 | 3 | 1 | 1 | 2 | 1 | 1 | 1 | 1 | 1 |
| 1192 | 32 | 2 | 158 | 50 | 8 | 2 | 1 | 1 | 5 | 1 | 1 | 1 | 1 | 1 | 1 | 1 | 1 | 1 |
| 1193 | 32 | 2 | 158 | 45 | 8 | 2 | 1 | 1 | 4 | 4 | 1 | 1 | 1 | 1 | 1 | 1 | 1 | 1 |
| 1194 | 32 | 2 | 159 | 55 | 6 | 2 | 1 | 1 | 5 | 4 | 1 | 1 | 1 | 1 | 1 | 1 | 1 | 1 |
| 1195 | 32 | 2 | 160 | 72 | 8 | 2 | 1 | 1 | 3 | 1 | 1 | 1 | 1 | 1 | 1 | 1 | 1 | 1 |
| 1196 | 32 | 2 | 160 | 67.5 | 4 | 2 | 1 | 1 | 5 | 4 | 1 | 1 | 1 | 1 | 1 | 1 | 1 | 1 |
| 1197 | 32 | 2 | 160 | 61 | 4 | 2 | 1 | 1 | 5 | 4 | 1 | 1 | 1 | 1 | 2 | 1 | 1 | 1 |
| 1198 | 32 | 2 | 160 | 58 | 3 | 2 | 1 | 1 | 5 | 3 | 1 | 1 | 2 | 1 | 1 | 1 | 1 | 1 |
| 1199 | 32 | 2 | 160 | 55 | 8 | 2 | 1 | 1 | 5 | 2 | 1 | 1 | 2 | 1 | 1 | 1 | 1 | 1 |
| 1200 | 32 | 2 | 160 | 55 | 2 | 2 | 1 | 1 | 1 | 3 | 1 | 1 | 2 | 1 | 1 | 1 | 1 | 1 |
| 1201 | 32 | 2 | 160 | 49 | 4 | 2 | 1 | 1 | 5 | 3 | 1 | 1 | 2 | 1 | 1 | 1 | 1 | 1 |
| 1202 | 32 | 2 | 160 | 48 | 2 | 2 | 1 | 1 | 5 | 4 | 1 | 1 | 2 | 1 | 1 | 1 | 1 | 1 |
| 1203 | 32 | 2 | 161 | 54.5 | 4 | 2 | 1 | 1 | 5 | 4 | 1 | 1 | 1 | 1 | 1 | 1 | 1 | 1 |
| 1204 | 32 | 2 | 161 | 52.5 | 4 | 2 | 1 | 1 | 5 | 2 | 1 | 1 | 1 | 1 | 2 | 1 | 1 | 1 |
| 1205 | 32 | 2 | 161 | 49 | 3 | 2 | 1 | 1 | 4 | 3 | 1 | 1 | 1 | 1 | 1 | 1 | 1 | 1 |
| 1206 | 32 | 2 | 162 | 50 | 8 | 2 | 1 | 1 | 4 | 4 | 1 | 1 | 2 | 1 | 1 | 1 | 1 | 1 |
| 1207 | 32 | 2 | 162 | 50 | 4 | 2 | 1 | 1 | 4 | 4 | 1 | 1 | 2 | 1 | 1 | 1 | 1 | 1 |
| 1208 | 32 | 2 | 163 | 72 | 8 | 2 | 1 | 1 | 5 | 2 | 1 | 1 | 1 | 1 | 1 | 1 | 1 | 1 |
| 1209 | 32 | 2 | 163 | 60 | 8 | 2 | 1 | 1 | 5 | 1 | 1 | 1 | 2 | 1 | 1 | 1 | 1 | 1 |
| 1210 | 32 | 2 | 163 | 58 | 3 | 2 | 1 | 1 | 5 | 4 | 1 | 1 | 2 | 1 | 2 | 1 | 1 | 1 |
| 1211 | 32 | 2 | 163 | 55 | 8 | 2 | 1 | 1 | 3 | 3 | 1 | 1 | 2 | 1 | 1 | 1 | 1 | 1 |
| 1212 | 32 | 2 | 163 | 55 | 1 | 2 | 1 | 1 | 5 | 3 | 1 | 1 | 2 | 1 | 1 | 1 | 1 | 1 |
| 1213 | 32 | 2 | 164 | 67 | 2 | 2 | 1 | 1 | 5 | 4 | 1 | 1 | 2 | 1 | 1 | 1 | 1 | 1 |
| 1214 | 32 | 2 | 165 | 53 | 3 | 2 | 1 | 2 | 3 | 3 | 1 | 1 | 2 | 1 | 1 | 1 | 1 | 1 |
| 1215 | 32 | 2 | 167 | 47.5 | 8 | 2 | 1 | 1 | 4 | 4 | 1 | 1 | 1 | 2 | 1 | 1 | 1 | 1 |
| 1216 | 32 | 2 | 169 | 55 | 4 | 2 | 1 | 2 | 5 | 4 | 1 | 1 | 1 | 1 | 2 | 1 | 2 | 1 |
| 1217 | 32 | 2 | 170 | 69 | 2 | 1 | 1 | 1 | 5 | 3 | 1 | 1 | 2 | 1 | 1 | 1 | 1 | 1 |
| 1218 | 32 | 2 | 170 | 62.5 | 3 | 2 | 1 | 1 | 5 | 2 | 1 | 1 | 2 | 1 | 1 | 1 | 1 | 1 |
| 1219 | 32 | 1 | 160 | 61.5 | 1 | 2 | 1 | 1 | 5 | 1 | 1 | 1 | 2 | 1 | 1 | 1 | 1 | 1 |
| 1220 | 32 | 1 | 168 | 65 | 4 | 1 | 1 | 1 | 5 | 3 | 1 | 1 | 1 | 1 | 1 | 1 | 1 | 1 |
| 1221 | 32 | 1 | 175 | 67.5 | 8 | 1 | 1 | 1 | 4 | 1 | 1 | 1 | 2 | 1 | 1 | 1 | 1 | 1 |
| 1222 | 32 | 1 | 178 | 77 | 8 | 2 | 1 | 1 | 5 | 3 | 1 | 1 | 2 | 1 | 1 | 1 | 1 | 1 |
| 1223 | 33 | 2 | 137 | 48 | 2 | 2 | 1 | 1 | 5 | 3 | 1 | 1 | 2 | 1 | 1 | 1 | 1 | 1 |
| 1224 | 33 | 2 | 142 | 46 | 4 | 1 | 1 | 2 | 5 | 1 | 1 | 1 | 1 | 1 | 1 | 1 | 1 | 1 |
| 1225 | 33 | 2 | 150 | 54 | 6 | 2 | 1 | 1 | 4 | 4 | 1 | 1 | 2 | 1 | 1 | 1 | 1 | 1 |
| 1226 | 33 | 2 | 150 | 48 | 4 | 2 | 1 | 1 | 6 | 3 | 1 | 1 | 1 | 1 | 1 | 1 | 1 | 2 |
| 1227 | 33 | 2 | 150 | 42 | 4 | 2 | 1 | 1 | 5 | 4 | 1 | 1 | 2 | 1 | 1 | 1 | 1 | 1 |
| 1228 | 33 | 2 | 153 | 50 | 8 | 2 | 1 | 1 | 5 | 3 | 1 | 1 | 1 | 1 | 1 | 1 | 1 | 1 |
| 1229 | 33 | 2 | 153 | 48 | 4 | 1 | 1 | 1 | 4 | 3 | 1 | 1 | 2 | 1 | 1 | 1 | 1 | 1 |
| 1230 | 33 | 2 | 154 | 52 | 3 | 2 | 1 | 2 | 5 | 3 | 1 | 1 | 2 | 1 | 1 | 1 | 1 | 1 |
| 1231 | 33 | 2 | 154 | 39 | 3 | 2 | 1 | 2 | 5 | 4 | 1 | 1 | 1 | 2 | 1 | 1 | 1 | 2 |
| 1232 | 33 | 2 | 155 | 105 | 8 | 2 | 1 | 1 | 3 | 4 | 1 | 1 | 1 | 1 | 1 | 1 | 1 | 1 |
| 1233 | 33 | 2 | 155 | 63 | 3 | 2 | 2 | 1 | 5 | 3 | 2 | 1 | 2 | 1 | 1 | 1 | 1 | 1 |
| 1234 | 33 | 2 | 155 | 55 | 2 | 2 | 1 | 1 | 5 | 3 | 1 | 1 | 2 | 1 | 1 | 1 | 1 | 1 |
| 1235 | 33 | 2 | 155 | 52 | 8 | 2 | 1 | 1 | 6 | 4 | 1 | 1 | 2 | 1 | 1 | 1 | 1 | 1 |
| 1236 | 33 | 2 | 155 | 48 | 2 | 2 | 1 | 2 | 5 | 3 | 1 | 1 | 1 | 1 | 2 | 1 | 1 | 1 |
| 1237 | 33 | 2 | 155 | 45.6 | 3 | 2 | 1 | 1 | 5 | 3 | 1 | 1 | 1 | 1 | 1 | 1 | 1 | 1 |
| 1238 | 33 | 2 | 157 | 57.5 | 4 | 2 | 1 | 1 | 4 | 4 | 1 | 1 | 1 | 1 | 1 | 1 | 1 | 1 |
| 1239 | 33 | 2 | 157 | 56 | 8 | 2 | 1 | 1 | 3 | 3 | 1 | 1 | 2 | 1 | 1 | 1 | 1 | 1 |
| 1240 | 33 | 2 | 157 | 52.5 | 8 | 2 | 1 | 1 | 2 | 4 | 1 | 1 | 2 | 1 | 1 | 1 | 1 | 1 |
| 1241 | 33 | 2 | 157 | 50 | 4 | 2 | 1 | 1 | 5 | 4 | 1 | 1 | 2 | 1 | 1 | 1 | 1 | 1 |
| 1242 | 33 | 2 | 158 | 75 | 8 | 2 | 1 | 1 | 5 | 1 | 1 | 1 | 2 | 1 | 1 | 1 | 1 | 1 |
| 1243 | 33 | 2 | 158 | 59 | 3 | 2 | 1 | 1 | 5 | 2 | 1 | 1 | 1 | 1 | 2 | 1 | 1 | 1 |
| 1244 | 33 | 2 | 158 | 58 | 2 | 2 | 1 | 1 | 5 | 2 | 1 | 1 | 1 | 1 | 1 | 1 | 1 | 1 |
| 1245 | 33 | 2 | 158 | 57.6 | 4 | 2 | 1 | 1 | 5 | 2 | 1 | 1 | 2 | 1 | 1 | 1 | 1 | 1 |
| 1246 | 33 | 2 | 158 | 52.5 | 8 | 2 | 1 | 1 | 5 | 4 | 1 | 1 | 2 | 1 | 1 | 1 | 1 | 1 |
| 1247 | 33 | 2 | 158 | 48 | 8 | 2 | 1 | 1 | 3 | 3 | 1 | 1 | 2 | 1 | 1 | 1 | 1 | 1 |
| 1248 | 33 | 2 | 158 | 40 | 8 | 2 | 1 | 1 | 5 | 4 | 1 | 1 | 2 | 1 | 1 | 1 | 1 | 1 |
| 1249 | 33 | 2 | 160 | 60 | 8 | 2 | 1 | 1 | 4 | 4 | 1 | 1 | 1 | 1 | 1 | 1 | 1 | 1 |
| 1250 | 33 | 2 | 160 | 60 | 2 | 2 | 1 | 1 | 5 | 3 | 1 | 1 | 2 | 1 | 1 | 1 | 1 | 1 |
| 1251 | 33 | 2 | 160 | 58.5 | 1 | 2 | 1 | 1 | 5 | 2 | 1 | 1 | 1 | 1 | 1 | 1 | 1 | 1 |
| 1252 | 33 | 2 | 160 | 55 | 3 | 2 | 1 | 1 | 5 | 2 | 1 | 1 | 1 | 1 | 1 | 1 | 1 | 1 |
| 1253 | 33 | 2 | 160 | 50 | 2 | 2 | 1 | 1 | 5 | 1 | 1 | 1 | 1 | 1 | 1 | 1 | 1 | 1 |
| 1254 | 33 | 2 | 160 | 47.5 | 4 | 2 | 1 | 1 | 5 | 2 | 1 | 1 | 1 | 1 | 2 | 1 | 1 | 1 |
| 1255 | 33 | 2 | 162 | 65 | 8 | 2 | 1 | 1 | 4 | 2 | 1 | 1 | 2 | 1 | 1 | 1 | 1 | 1 |
| 1256 | 33 | 2 | 163 | 57.5 | 8 | 1 | 1 | 1 | 5 | 2 | 1 | 1 | 1 | 1 | 1 | 1 | 1 | 1 |
| 1257 | 33 | 2 | 164 | 66 | 2 | 2 | 1 | 1 | 5 | 2 | 1 | 1 | 1 | 1 | 1 | 1 | 1 | 1 |
| 1258 | 33 | 2 | 164 | 48 | 2 | 1 | 1 | 1 | 5 | 3 | 1 | 1 | 1 | 1 | 1 | 1 | 1 | 1 |
| 1259 | 33 | 2 | 165 | 50 | 4 | 2 | 1 | 1 | 4 | 2 | 1 | 2 | 1 | 1 | 2 | 1 | 1 | 1 |
| 1260 | 33 | 2 | 167 | 66 | 8 | 2 | 1 | 1 | 3 | 3 | 1 | 1 | 1 | 2 | 1 | 1 | 1 | 2 |
| 1261 | 33 | 2 | 168 | 72.5 | 8 | 2 | 1 | 1 | 5 | 3 | 1 | 1 | 1 | 1 | 1 | 1 | 1 | 1 |
| 1262 | 33 | 2 | 168 | 58 | 3 | 2 | 1 | 1 | 6 | 3 | 1 | 1 | 2 | 1 | 1 | 1 | 1 | 1 |
| 1263 | 33 | 2 | 168 | 49 | 8 | 2 | 1 | 1 | 4 | 4 | 1 | 1 | 2 | 1 | 1 | 1 | 1 | 1 |
| 1264 | 33 | 1 | 160 | 52 | 8 | 2 | 1 | 1 | 5 | 4 | 1 | 1 | 1 | 1 | 1 | 1 | 1 | 2 |
| 1265 | 33 | 1 | 163 | 65 | 2 | 2 | 1 | 1 | 5 | 3 | 1 | 1 | 2 | 1 | 1 | 1 | 1 | 1 |
| 1266 | 33 | 1 | 164 | 62 | 2 | 2 | 1 | 1 | 5 | 2 | 1 | 1 | 2 | 1 | 1 | 1 | 1 | 1 |
| 1267 | 33 | 1 | 168 | 65 | 8 | 2 | 1 | 1 | 5 | 1 | 1 | 1 | 1 | 1 | 1 | 1 | 1 | 1 |
| 1268 | 33 | 1 | 168 | 60 | 8 | 2 | 1 | 1 | 5 | 2 | 1 | 1 | 1 | 1 | 1 | 1 | 1 | 1 |
| 1269 | 33 | 1 | 170 | 60 | 4 | 2 | 1 | 1 | 3 | 3 | 1 | 1 | 1 | 2 | 1 | 1 | 1 | 2 |
| 1270 | 33 | 1 | 170 | 56 | 8 | 2 | 1 | 1 | 4 | 4 | 1 | 1 | 1 | 1 | 1 | 1 | 1 | 1 |
| 1271 | 33 | 1 | 177 | 95 | 8 | 2 | 1 | 1 | 5 | 3 | 1 | 1 | 1 | 1 | 1 | 1 | 1 | 1 |
| 1272 | 34 | 2 | 148 | 45.5 | 4 | 2 | 1 | 1 | 5 | 3 | 2 | 1 | 1 | 1 | 1 | 1 | 1 | 1 |
| 1273 | 34 | 2 | 150 | 40 | 3 | 2 | 1 | 1 | 4 | 3 | 1 | 1 | 2 | 1 | 1 | 1 | 1 | 1 |
| 1274 | 34 | 2 | 153 | 50 | 8 | 2 | 1 | 1 | 5 | 4 | 1 | 1 | 2 | 1 | 1 | 1 | 1 | 1 |
| 1275 | 34 | 2 | 153 | 40 | 8 | 2 | 1 | 1 | 4 | 1 | 1 | 1 | 1 | 2 | 1 | 1 | 1 | 1 |
| 1276 | 34 | 2 | 155 | 60 | 3 | 2 | 1 | 1 | 4 | 4 | 1 | 1 | 1 | 1 | 1 | 1 | 1 | 1 |
| 1277 | 34 | 2 | 156 | 42.5 | 2 | 2 | 1 | 1 | 5 | 3 | 1 | 1 | 1 | 1 | 1 | 1 | 1 | 1 |
| 1278 | 34 | 2 | 157 | 55 | 8 | 2 | 1 | 1 | 4 | 3 | 1 | 1 | 2 | 1 | 1 | 1 | 1 | 2 |
| 1279 | 34 | 2 | 158 | 74 | 8 | 2 | 1 | 1 | 4 | 3 | 1 | 1 | 2 | 1 | 1 | 1 | 1 | 1 |
| 1280 | 34 | 2 | 158 | 60 | 8 | 2 | 1 | 1 | 4 | 4 | 1 | 1 | 1 | 1 | 1 | 1 | 1 | 1 |
| 1281 | 34 | 2 | 158 | 60 | 2 | 4 | 1 | 1 | 5 | 2 | 1 | 1 | 1 | 1 | 1 | 1 | 1 | 1 |
| 1282 | 34 | 2 | 158 | 57 | 3 | 1 | 1 | 1 | 4 | 1 | 1 | 1 | 1 | 1 | 1 | 1 | 1 | 1 |
| 1283 | 34 | 2 | 158 | 53 | 8 | 2 | 1 | 1 | 5 | 2 | 1 | 1 | 1 | 1 | 2 | 1 | 1 | 1 |
| 1284 | 34 | 2 | 159 | 55 | 8 | 2 | 1 | 1 | 4 | 4 | 1 | 1 | 1 | 1 | 1 | 1 | 1 | 1 |
| 1285 | 34 | 2 | 160 | 90 | 8 | 2 | 1 | 1 | 5 | 4 | 1 | 1 | 1 | 1 | 1 | 1 | 1 | 1 |
| 1286 | 34 | 2 | 160 | 70 | 4 | 2 | 1 | 1 | 5 | 3 | 1 | 1 | 2 | 1 | 1 | 1 | 1 | 1 |
| 1287 | 34 | 2 | 160 | 60 | 8 | 2 | 1 | 1 | 5 | 1 | 1 | 1 | 1 | 1 | 1 | 1 | 1 | 1 |
| 1288 | 34 | 2 | 160 | 55 | 8 | 2 | 1 | 1 | 3 | 3 | 1 | 1 | 1 | 1 | 1 | 1 | 1 | 1 |
| 1289 | 34 | 2 | 160 | 54 | 4 | 2 | 1 | 1 | 4 | 3 | 1 | 1 | 1 | 1 | 1 | 1 | 1 | 1 |
| 1290 | 34 | 2 | 160 | 54 | 3 | 2 | 1 | 1 | 4 | 4 | 1 | 1 | 2 | 1 | 1 | 1 | 1 | 1 |
| 1291 | 34 | 2 | 160 | 53 | 8 | 2 | 1 | 1 | 5 | 4 | 1 | 1 | 1 | 1 | 1 | 1 | 1 | 1 |
| 1292 | 34 | 2 | 160 | 51 | 8 | 2 | 1 | 1 | 4 | 3 | 1 | 1 | 1 | 2 | 1 | 1 | 1 | 2 |
| 1293 | 34 | 2 | 160 | 44 | 8 | 2 | 1 | 1 | 5 | 4 | 1 | 1 | 1 | 1 | 1 | 1 | 1 | 1 |
| 1294 | 34 | 2 | 160 | 40 | 3 | 2 | 1 | 1 | 5 | 2 | 1 | 1 | 1 | 1 | 1 | 1 | 1 | 1 |
| 1295 | 34 | 2 | 161 | 54 | 1 | 2 | 1 | 1 | 5 | 4 | 1 | 1 | 1 | 1 | 1 | 1 | 1 | 1 |
| 1296 | 34 | 2 | 162 | 55.8 | 8 | 2 | 1 | 1 | 4 | 1 | 1 | 1 | 2 | 1 | 2 | 1 | 1 | 1 |
| 1297 | 34 | 2 | 163 | 93.2 | 8 | 2 | 1 | 1 | 4 | 2 | 1 | 1 | 2 | 1 | 1 | 1 | 1 | 1 |
| 1298 | 34 | 2 | 163 | 60 | 8 | 2 | 1 | 1 | 3 | 2 | 1 | 1 | 1 | 1 | 1 | 1 | 1 | 1 |
| 1299 | 34 | 2 | 163 | 60 | 3 | 2 | 1 | 1 | 5 | 2 | 1 | 1 | 2 | 1 | 2 | 1 | 1 | 1 |
| 1300 | 34 | 2 | 163 | 55 | 8 | 1 | 1 | 1 | 5 | 3 | 1 | 1 | 2 | 1 | 2 | 1 | 1 | 1 |
| 1301 | 34 | 2 | 164 | 50 | 1 | 2 | 1 | 1 | 5 | 4 | 1 | 1 | 2 | 1 | 1 | 1 | 1 | 1 |
| 1302 | 34 | 2 | 164 | 46 | 4 | 2 | 1 | 1 | 4 | 1 | 1 | 1 | 2 | 2 | 1 | 1 | 1 | 2 |
| 1303 | 34 | 2 | 164.5 | 69 | 4 | 2 | 1 | 1 | 3 | 3 | 1 | 1 | 1 | 1 | 1 | 1 | 1 | 1 |
| 1304 | 34 | 1 | 163 | 70 | 8 | 2 | 2 | 2 | 4 | 2 | 1 | 1 | 1 | 1 | 1 | 1 | 1 | 1 |
| 1305 | 34 | 1 | 171 | 70 | 2 | 2 | 1 | 1 | 5 | 1 | 1 | 1 | 2 | 1 | 1 | 1 | 1 | 1 |
| 1306 | 34 | 1 | 172 | 65 | 4 | 2 | 1 | 1 | 5 | 4 | 1 | 1 | 1 | 1 | 1 | 1 | 3 | 1 |
| 1307 | 35 | 2 | 145 | 48 | 2 | 2 | 1 | 1 | 5 | 3 | 1 | 1 | 2 | 1 | 1 | 1 | 1 | 1 |
| 1308 | 35 | 2 | 148 | 44 | 8 | 2 | 1 | 1 | 5 | 4 | 1 | 1 | 1 | 1 | 1 | 1 | 1 | 1 |
| 1309 | 35 | 2 | 150 | 40 | 4 | 2 | 1 | 1 | 4 | 3 | 1 | 1 | 1 | 1 | 1 | 1 | 1 | 1 |
| 1310 | 35 | 2 | 153 | 43 | 8 | 2 | 1 | 1 | 5 | 3 | 1 | 1 | 2 | 1 | 1 | 1 | 1 | 1 |
| 1311 | 35 | 2 | 154 | 42 | 4 | 2 | 1 | 1 | 3 | 4 | 1 | 1 | 1 | 1 | 1 | 1 | 1 | 1 |
| 1312 | 35 | 2 | 155 | 60 | 8 | 2 | 1 | 1 | 5 | 2 | 1 | 1 | 1 | 1 | 2 | 1 | 1 | 1 |
| 1313 | 35 | 2 | 155 | 51.5 | 2 | 2 | 1 | 1 | 5 | 4 | 1 | 1 | 1 | 1 | 1 | 1 | 1 | 1 |
| 1314 | 35 | 2 | 155 | 50 | 8 | 2 | 1 | 1 | 3 | 4 | 1 | 1 | 1 | 1 | 1 | 1 | 1 | 1 |
| 1315 | 35 | 2 | 155 | 40 | 8 | 2 | 1 | 1 | 5 | 3 | 1 | 1 | 2 | 1 | 1 | 1 | 1 | 1 |
| 1316 | 35 | 2 | 156 | 53 | 8 | 2 | 1 | 1 | 5 | 1 | 1 | 1 | 2 | 1 | 1 | 1 | 1 | 1 |
| 1317 | 35 | 2 | 156 | 44 | 8 | 2 | 2 | 1 | 6 | 4 | 1 | 1 | 2 | 1 | 1 | 1 | 1 | 1 |
| 1318 | 35 | 2 | 157 | 61 | 2 | 2 | 1 | 2 | 4 | 4 | 1 | 1 | 1 | 1 | 1 | 1 | 1 | 1 |
| 1319 | 35 | 2 | 157 | 52 | 2 | 2 | 1 | 1 | 4 | 1 | 1 | 1 | 1 | 1 | 1 | 1 | 1 | 1 |
| 1320 | 35 | 2 | 157 | 50 | 4 | 2 | 1 | 1 | 5 | 2 | 1 | 1 | 1 | 1 | 1 | 1 | 1 | 1 |
| 1321 | 35 | 2 | 158 | 70 | 8 | 2 | 1 | 1 | 4 | 4 | 1 | 1 | 2 | 1 | 1 | 1 | 1 | 1 |
| 1322 | 35 | 2 | 158 | 60 | 8 | 2 | 1 | 2 | 5 | 2 | 1 | 1 | 1 | 1 | 1 | 1 | 1 | 1 |
| 1323 | 35 | 2 | 158 | 60 | 4 | 2 | 1 | 1 | 4 | 3 | 1 | 1 | 2 | 1 | 1 | 1 | 1 | 1 |
| 1324 | 35 | 2 | 158 | 59.5 | 4 | 2 | 2 | 1 | 3 | 3 | 1 | 1 | 2 | 1 | 1 | 1 | 1 | 1 |
| 1325 | 35 | 2 | 158 | 57 | 8 | 2 | 1 | 2 | 5 | 3 | 1 | 1 | 2 | 1 | 1 | 1 | 1 | 1 |
| 1326 | 35 | 2 | 158 | 57 | 2 | 2 | 1 | 1 | 5 | 4 | 1 | 1 | 1 | 1 | 1 | 1 | 1 | 1 |
| 1327 | 35 | 2 | 158 | 55.2 | 3 | 2 | 1 | 1 | 5 | 3 | 1 | 1 | 1 | 1 | 1 | 1 | 1 | 1 |
| 1328 | 35 | 2 | 158 | 55 | 2 | 2 | 1 | 1 | 5 | 4 | 1 | 1 | 1 | 1 | 1 | 1 | 1 | 1 |
| 1329 | 35 | 2 | 159 | 60 | 8 | 2 | 1 | 1 | 3 | 4 | 1 | 1 | 2 | 1 | 1 | 1 | 1 | 1 |
| 1330 | 35 | 2 | 159 | 48 | 8 | 2 | 1 | 1 | 5 | 3 | 1 | 1 | 2 | 1 | 2 | 1 | 1 | 1 |
| 1331 | 35 | 2 | 160 | 56.5 | 2 | 2 | 1 | 1 | 5 | 1 | 2 | 1 | 2 | 1 | 1 | 1 | 1 | 1 |
| 1332 | 35 | 2 | 160 | 52 | 4 | 1 | 1 | 1 | 4 | 4 | 1 | 1 | 2 | 1 | 2 | 2 | 1 | 1 |
| 1333 | 35 | 2 | 160 | 52 | 2 | 2 | 1 | 1 | 5 | 4 | 1 | 1 | 1 | 1 | 1 | 1 | 1 | 1 |
| 1334 | 35 | 2 | 160 | 50 | 4 | 2 | 1 | 1 | 4 | 2 | 1 | 1 | 1 | 1 | 1 | 1 | 1 | 1 |
| 1335 | 35 | 2 | 160 | 49 | 8 | 2 | 1 | 1 | 3 | 3 | 1 | 1 | 2 | 1 | 1 | 1 | 1 | 1 |
| 1336 | 35 | 2 | 161 | 50 | 8 | 2 | 1 | 1 | 4 | 4 | 1 | 1 | 1 | 1 | 1 | 1 | 1 | 1 |
| 1337 | 35 | 2 | 162 | 58 | 8 | 2 | 1 | 1 | 4 | 3 | 1 | 1 | 1 | 1 | 1 | 1 | 1 | 1 |
| 1338 | 35 | 2 | 162 | 53 | 4 | 1 | 1 | 1 | 5 | 2 | 1 | 1 | 1 | 1 | 1 | 1 | 1 | 1 |
| 1339 | 35 | 2 | 163 | 48 | 3 | 2 | 1 | 1 | 5 | 3 | 1 | 1 | 2 | 1 | 1 | 1 | 1 | 1 |
| 1340 | 35 | 2 | 164 | 65 | 8 | 2 | 1 | 1 | 5 | 3 | 1 | 1 | 1 | 1 | 2 | 1 | 1 | 1 |
| 1341 | 35 | 2 | 164 | 60 | 8 | 2 | 1 | 1 | 3 | 2 | 1 | 1 | 2 | 1 | 1 | 1 | 1 | 1 |
| 1342 | 35 | 2 | 165 | 60 | 8 | 2 | 1 | 1 | 5 | 3 | 1 | 1 | 1 | 1 | 1 | 1 | 1 | 1 |
| 1343 | 35 | 2 | 165 | 60 | 1 | 2 | 1 | 1 | 3 | 2 | 1 | 1 | 2 | 1 | 1 | 1 | 1 | 1 |
| 1344 | 35 | 2 | 165 | 58 | 8 | 1 | 1 | 1 | 2 | 1 | 1 | 1 | 1 | 1 | 1 | 1 | 1 | 2 |
| 1345 | 35 | 2 | 165 | 54 | 4 | 2 | 1 | 1 | 3 | 4 | 1 | 1 | 1 | 1 | 1 | 1 | 1 | 1 |
| 1346 | 35 | 2 | 165 | 45 | 8 | 2 | 1 | 1 | 3 | 4 | 1 | 1 | 2 | 1 | 1 | 1 | 1 | 1 |
| 1347 | 35 | 2 | 166 | 57 | 8 | 2 | 1 | 1 | 4 | 1 | 2 | 1 | 1 | 2 | 2 | 1 | 1 | 2 |
| 1348 | 35 | 1 | 164 | 65.5 | 8 | 2 | 2 | 2 | 5 | 4 | 1 | 2 | 2 | 1 | 1 | 1 | 1 | 1 |
| 1349 | 35 | 1 | 168 | 80 | 4 | 2 | 1 | 2 | 5 | 3 | 1 | 1 | 1 | 1 | 1 | 1 | 3 | 1 |
| 1350 | 35 | 1 | 168 | 66.5 | 4 | 2 | 1 | 3 | 5 | 4 | 1 | 1 | 1 | 1 | 1 | 1 | 1 | 1 |
| 1351 | 35 | 1 | 168 | 66 | 8 | 1 | 1 | 1 | 5 | 3 | 1 | 1 | 1 | 1 | 1 | 1 | 1 | 1 |
| 1352 | 35 | 1 | 170 | 100 | 2 | 1 | 1 | 1 | 5 | 2 | 1 | 1 | 2 | 1 | 1 | 1 | 3 | 1 |
| 1353 | 35 | 1 | 170 | 89 | 8 | 1 | 1 | 1 | 2 | 4 | 1 | 1 | 1 | 1 | 1 | 1 | 1 | 1 |
| 1354 | 35 | 1 | 170 | 76 | 4 | 2 | 1 | 1 | 5 | 4 | 1 | 1 | 2 | 1 | 1 | 1 | 1 | 1 |
| 1355 | 35 | 1 | 170 | 75 | 1 | 2 | 1 | 1 | 4 | 3 | 1 | 1 | 2 | 1 | 1 | 1 | 1 | 1 |
| 1356 | 35 | 1 | 170 | 72.5 | 8 | 2 | 1 | 1 | 3 | 4 | 1 | 1 | 1 | 1 | 1 | 1 | 1 | 2 |
| 1357 | 35 | 1 | 172 | 74 | 4 | 2 | 1 | 2 | 5 | 2 | 1 | 1 | 1 | 1 | 1 | 1 | 1 | 1 |
| 1358 | 35 | 1 | 173 | 60 | 2 | 2 | 1 | 1 | 5 | 3 | 1 | 1 | 2 | 1 | 1 | 1 | 3 | 1 |
| 1359 | 35 | 1 | 176 | 87.5 | 8 | 1 | 1 | 1 | 5 | 2 | 1 | 1 | 2 | 1 | 1 | 1 | 1 | 1 |
| 1360 | 36 | 2 | 150 | 45 | 8 | 2 | 1 | 1 | 5 | 4 | 1 | 1 | 2 | 1 | 1 | 1 | 1 | 1 |
| 1361 | 36 | 2 | 150 | 45 | 8 | 2 | 1 | 1 | 3 | 4 | 1 | 1 | 1 | 1 | 1 | 1 | 1 | 1 |
| 1362 | 36 | 2 | 150.5 | 50.2 | 2 | 2 | 1 | 1 | 5 | 4 | 1 | 1 | 1 | 1 | 1 | 1 | 1 | 1 |
| 1363 | 36 | 2 | 151 | 56.2 | 2 | 2 | 1 | 1 | 5 | 4 | 1 | 1 | 2 | 1 | 2 | 1 | 1 | 1 |
| 1364 | 36 | 2 | 152 | 52 | 8 | 2 | 1 | 1 | 4 | 2 | 1 | 1 | 1 | 1 | 1 | 1 | 1 | 1 |
| 1365 | 36 | 2 | 153 | 47.5 | 8 | 2 | 1 | 1 | 4 | 4 | 1 | 1 | 1 | 1 | 2 | 1 | 1 | 1 |
| 1366 | 36 | 2 | 154 | 48 | 3 | 2 | 1 | 1 | 5 | 4 | 1 | 1 | 1 | 1 | 1 | 1 | 1 | 1 |
| 1367 | 36 | 2 | 154 | 47 | 4 | 2 | 1 | 1 | 4 | 4 | 2 | 1 | 1 | 1 | 1 | 1 | 1 | 1 |
| 1368 | 36 | 2 | 155 | 65 | 8 | 2 | 1 | 1 | 4 | 4 | 1 | 1 | 1 | 1 | 1 | 1 | 1 | 1 |
| 1369 | 36 | 2 | 155 | 58 | 4 | 2 | 1 | 1 | 4 | 4 | 1 | 1 | 1 | 1 | 1 | 1 | 1 | 1 |
| 1370 | 36 | 2 | 155 | 47 | 8 | 2 | 1 | 1 | 4 | 2 | 1 | 1 | 1 | 2 | 1 | 1 | 1 | 2 |
| 1371 | 36 | 2 | 155 | 44.6 | 8 | 2 | 1 | 1 | 5 | 4 | 1 | 1 | 2 | 1 | 1 | 1 | 1 | 1 |
| 1372 | 36 | 2 | 157 | 50 | 8 | 2 | 1 | 1 | 4 | 1 | 1 | 1 | 1 | 1 | 1 | 1 | 1 | 1 |
| 1373 | 36 | 2 | 158 | 58 | 4 | 2 | 1 | 2 | 3 | 2 | 1 | 1 | 2 | 1 | 1 | 1 | 1 | 2 |
| 1374 | 36 | 2 | 158 | 55 | 8 | 2 | 1 | 1 | 5 | 4 | 1 | 1 | 2 | 1 | 1 | 1 | 1 | 1 |
| 1375 | 36 | 2 | 158 | 55 | 3 | 2 | 1 | 2 | 4 | 3 | 2 | 1 | 2 | 1 | 1 | 1 | 1 | 1 |
| 1376 | 36 | 2 | 158 | 52 | 8 | 2 | 1 | 1 | 5 | 3 | 1 | 1 | 1 | 1 | 1 | 1 | 1 | 1 |
| 1377 | 36 | 2 | 158 | 52 | 8 | 2 | 1 | 1 | 4 | 4 | 1 | 1 | 2 | 1 | 1 | 1 | 1 | 1 |
| 1378 | 36 | 2 | 158 | 51 | 8 | 2 | 1 | 1 | 5 | 3 | 1 | 1 | 2 | 1 | 1 | 1 | 1 | 1 |
| 1379 | 36 | 2 | 158 | 50.5 | 8 | 2 | 1 | 1 | 4 | 4 | 1 | 1 | 1 | 1 | 1 | 1 | 1 | 1 |
| 1380 | 36 | 2 | 159 | 60 | 8 | 2 | 1 | 1 | 4 | 3 | 1 | 1 | 2 | 1 | 1 | 1 | 1 | 1 |
| 1381 | 36 | 2 | 159 | 51.5 | 3 | 2 | 1 | 1 | 5 | 4 | 1 | 1 | 1 | 1 | 1 | 1 | 1 | 1 |
| 1382 | 36 | 2 | 159.5 | 44 | 8 | 2 | 1 | 1 | 5 | 4 | 1 | 1 | 2 | 1 | 2 | 1 | 1 | 1 |
| 1383 | 36 | 2 | 160 | 56 | 3 | 2 | 1 | 1 | 5 | 2 | 1 | 1 | 1 | 1 | 2 | 1 | 1 | 1 |
| 1384 | 36 | 2 | 160 | 54 | 8 | 2 | 1 | 1 | 5 | 2 | 1 | 1 | 1 | 1 | 1 | 1 | 1 | 1 |
| 1385 | 36 | 2 | 160 | 45 | 3 | 2 | 1 | 1 | 5 | 3 | 1 | 1 | 2 | 1 | 1 | 1 | 1 | 1 |
| 1386 | 36 | 2 | 161 | 65 | 8 | 3 | 1 | 1 | 4 | 4 | 1 | 1 | 2 | 1 | 1 | 1 | 1 | 1 |
| 1387 | 36 | 2 | 162 | 79 | 4 | 2 | 1 | 1 | 5 | 1 | 1 | 1 | 2 | 1 | 1 | 1 | 1 | 1 |
| 1388 | 36 | 2 | 162 | 65 | 3 | 1 | 1 | 1 | 4 | 3 | 1 | 2 | 1 | 1 | 1 | 1 | 1 | 1 |
| 1389 | 36 | 2 | 162 | 55 | 8 | 2 | 1 | 1 | 3 | 3 | 1 | 1 | 1 | 1 | 1 | 1 | 1 | 1 |
| 1390 | 36 | 2 | 162 | 54 | 8 | 2 | 2 | 1 | 3 | 2 | 1 | 1 | 1 | 1 | 1 | 1 | 1 | 2 |
| 1391 | 36 | 2 | 162 | 52 | 3 | 2 | 1 | 1 | 4 | 1 | 1 | 1 | 1 | 1 | 1 | 1 | 1 | 1 |
| 1392 | 36 | 2 | 163 | 53 | 3 | 2 | 1 | 1 | 5 | 3 | 1 | 1 | 2 | 1 | 1 | 1 | 1 | 1 |
| 1393 | 36 | 2 | 163 | 52 | 4 | 2 | 2 | 1 | 5 | 3 | 1 | 1 | 1 | 1 | 1 | 1 | 1 | 1 |
| 1394 | 36 | 2 | 163 | 47 | 8 | 2 | 1 | 1 | 4 | 3 | 1 | 1 | 2 | 1 | 2 | 1 | 1 | 1 |
| 1395 | 36 | 2 | 165 | 64 | 3 | 2 | 1 | 1 | 5 | 4 | 1 | 1 | 1 | 1 | 1 | 1 | 1 | 1 |
| 1396 | 36 | 2 | 165 | 58 | 8 | 2 | 1 | 1 | 5 | 1 | 1 | 1 | 2 | 1 | 1 | 1 | 1 | 1 |
| 1397 | 36 | 2 | 166 | 64 | 2 | 2 | 1 | 1 | 5 | 2 | 1 | 1 | 2 | 2 | 2 | 1 | 1 | 2 |
| 1398 | 36 | 1 | 153 | 45 | 2 | 1 | 1 | 1 | 5 | 1 | 1 | 1 | 1 | 1 | 1 | 1 | 1 | 2 |
| 1399 | 36 | 1 | 160 | 65 | 4 | 2 | 1 | 1 | 4 | 4 | 1 | 1 | 1 | 1 | 1 | 1 | 1 | 1 |
| 1400 | 36 | 1 | 160 | 54 | 8 | 2 | 1 | 1 | 5 | 2 | 1 | 1 | 1 | 1 | 1 | 1 | 1 | 1 |
| 1401 | 36 | 1 | 166 | 82 | 4 | 2 | 1 | 1 | 3 | 3 | 1 | 1 | 1 | 2 | 1 | 1 | 1 | 2 |
| 1402 | 36 | 1 | 168 | 70 | 8 | 2 | 1 | 1 | 4 | 1 | 1 | 1 | 1 | 1 | 1 | 1 | 1 | 1 |
| 1403 | 36 | 1 | 170 | 66 | 1 | 2 | 1 | 1 | 5 | 1 | 1 | 1 | 1 | 1 | 1 | 1 | 1 | 1 |
| 1404 | 36 | 1 | 170 | 65 | 8 | 2 | 1 | 1 | 3 | 4 | 1 | 1 | 1 | 2 | 1 | 1 | 1 | 2 |
| 1405 | 36 | 1 | 173 | 65 | 4 | 2 | 1 | 1 | 5 | 3 | 1 | 1 | 2 | 1 | 1 | 1 | 3 | 1 |
| 1406 | 37 | 2 | 150 | 45 | 8 | 2 | 1 | 1 | 4 | 4 | 1 | 1 | 2 | 1 | 1 | 1 | 1 | 1 |
| 1407 | 37 | 2 | 153 | 42 | 1 | 2 | 1 | 2 | 6 | 2 | 1 | 1 | 1 | 1 | 1 | 1 | 1 | 1 |
| 1408 | 37 | 2 | 155 | 54 | 8 | 2 | 1 | 1 | 4 | 1 | 2 | 1 | 1 | 1 | 1 | 1 | 1 | 1 |
| 1409 | 37 | 2 | 155 | 44 | 8 | 2 | 1 | 1 | 5 | 1 | 1 | 1 | 2 | 1 | 1 | 1 | 1 | 1 |
| 1410 | 37 | 2 | 156 | 65 | 8 | 2 | 1 | 1 | 4 | 3 | 1 | 1 | 1 | 1 | 1 | 1 | 1 | 1 |
| 1411 | 37 | 2 | 156 | 55 | 3 | 2 | 1 | 1 | 5 | 2 | 1 | 1 | 1 | 1 | 1 | 1 | 1 | 1 |
| 1412 | 37 | 2 | 156 | 54 | 4 | 2 | 1 | 1 | 5 | 3 | 2 | 1 | 1 | 1 | 1 | 1 | 1 | 2 |
| 1413 | 37 | 2 | 156 | 50 | 8 | 2 | 1 | 1 | 5 | 4 | 1 | 1 | 1 | 1 | 2 | 1 | 1 | 1 |
| 1414 | 37 | 2 | 156 | 50 | 4 | 2 | 1 | 1 | 5 | 3 | 1 | 1 | 2 | 1 | 1 | 1 | 1 | 1 |
| 1415 | 37 | 2 | 156 | 50 | 3 | 1 | 1 | 2 | 5 | 2 | 1 | 1 | 2 | 1 | 1 | 1 | 1 | 1 |
| 1416 | 37 | 2 | 156 | 46 | 2 | 2 | 1 | 1 | 5 | 3 | 1 | 1 | 2 | 1 | 1 | 1 | 1 | 1 |
| 1417 | 37 | 2 | 156 | 45 | 8 | 2 | 1 | 1 | 5 | 2 | 1 | 1 | 1 | 1 | 1 | 1 | 1 | 1 |
| 1418 | 37 | 2 | 157 | 48 | 2 | 2 | 1 | 1 | 5 | 4 | 1 | 1 | 1 | 1 | 2 | 1 | 1 | 1 |
| 1419 | 37 | 2 | 158 | 60 | 8 | 2 | 2 | 1 | 5 | 4 | 1 | 1 | 1 | 1 | 1 | 1 | 1 | 1 |
| 1420 | 37 | 2 | 158 | 60 | 8 | 2 | 1 | 1 | 3 | 4 | 1 | 2 | 1 | 1 | 1 | 1 | 1 | 1 |
| 1421 | 37 | 2 | 158 | 50 | 4 | 2 | 1 | 2 | 3 | 3 | 1 | 1 | 1 | 1 | 1 | 1 | 1 | 1 |
| 1422 | 37 | 2 | 158 | 48 | 8 | 2 | 1 | 1 | 4 | 3 | 1 | 1 | 1 | 1 | 1 | 1 | 1 | 1 |
| 1423 | 37 | 2 | 159 | 49 | 4 | 2 | 1 | 1 | 5 | 4 | 1 | 1 | 2 | 1 | 2 | 1 | 1 | 1 |
| 1424 | 37 | 2 | 159 | 48 | 4 | 2 | 1 | 1 | 4 | 3 | 1 | 1 | 2 | 1 | 1 | 1 | 3 | 1 |
| 1425 | 37 | 2 | 160 | 68 | 8 | 2 | 1 | 1 | 5 | 3 | 1 | 1 | 2 | 1 | 2 | 1 | 1 | 1 |
| 1426 | 37 | 2 | 160 | 61.5 | 8 | 2 | 1 | 1 | 5 | 4 | 1 | 1 | 1 | 1 | 1 | 1 | 1 | 1 |
| 1427 | 37 | 2 | 160 | 58 | 2 | 2 | 1 | 1 | 5 | 4 | 1 | 2 | 1 | 1 | 1 | 1 | 1 | 1 |
| 1428 | 37 | 2 | 160 | 55 | 8 | 2 | 1 | 1 | 5 | 4 | 1 | 1 | 2 | 1 | 1 | 1 | 1 | 1 |
| 1429 | 37 | 2 | 160 | 55 | 8 | 1 | 1 | 1 | 1 | 4 | 1 | 1 | 1 | 1 | 1 | 1 | 1 | 1 |
| 1430 | 37 | 2 | 160 | 52 | 4 | 2 | 1 | 1 | 3 | 2 | 1 | 1 | 2 | 1 | 1 | 1 | 1 | 1 |
| 1431 | 37 | 2 | 160 | 50 | 8 | 2 | 1 | 1 | 5 | 4 | 1 | 1 | 2 | 1 | 1 | 1 | 1 | 1 |
| 1432 | 37 | 2 | 161 | 57 | 3 | 2 | 1 | 1 | 5 | 2 | 1 | 1 | 2 | 1 | 1 | 1 | 1 | 1 |
| 1433 | 37 | 2 | 161 | 55 | 2 | 2 | 1 | 1 | 5 | 2 | 1 | 1 | 2 | 1 | 1 | 1 | 1 | 1 |
| 1434 | 37 | 2 | 162 | 68 | 8 | 2 | 1 | 1 | 5 | 4 | 1 | 1 | 2 | 1 | 1 | 1 | 1 | 2 |
| 1435 | 37 | 2 | 163 | 65 | 4 | 2 | 1 | 1 | 5 | 3 | 2 | 1 | 1 | 1 | 2 | 1 | 1 | 2 |
| 1436 | 37 | 2 | 163 | 62.5 | 4 | 2 | 1 | 1 | 4 | 4 | 1 | 1 | 2 | 1 | 1 | 1 | 1 | 1 |
| 1437 | 37 | 2 | 163 | 58 | 1 | 2 | 1 | 1 | 5 | 3 | 1 | 1 | 1 | 1 | 1 | 1 | 1 | 1 |
| 1438 | 37 | 2 | 163 | 50 | 4 | 2 | 1 | 1 | 4 | 3 | 1 | 1 | 2 | 1 | 1 | 1 | 1 | 1 |
| 1439 | 37 | 2 | 164 | 70 | 8 | 2 | 1 | 1 | 6 | 2 | 1 | 1 | 2 | 1 | 1 | 1 | 1 | 1 |
| 1440 | 37 | 2 | 164 | 67 | 8 | 2 | 1 | 1 | 6 | 3 | 1 | 1 | 1 | 2 | 1 | 1 | 1 | 2 |
| 1441 | 37 | 2 | 165 | 64 | 3 | 2 | 1 | 1 | 4 | 2 | 1 | 1 | 1 | 1 | 1 | 1 | 1 | 1 |
| 1442 | 37 | 2 | 165 | 52 | 8 | 2 | 1 | 1 | 4 | 3 | 1 | 1 | 1 | 1 | 1 | 1 | 1 | 1 |
| 1443 | 37 | 2 | 166 | 60 | 1 | 2 | 1 | 1 | 5 | 4 | 1 | 1 | 1 | 1 | 1 | 1 | 1 | 1 |
| 1444 | 37 | 2 | 166.5 | 57.1 | 3 | 2 | 1 | 1 | 5 | 4 | 1 | 1 | 1 | 1 | 1 | 1 | 1 | 1 |
| 1445 | 37 | 2 | 172 | 59 | 2 | 2 | 1 | 1 | 4 | 3 | 1 | 1 | 1 | 1 | 1 | 1 | 1 | 2 |
| 1446 | 37 | 2 | 182 | 65 | 4 | 2 | 1 | 1 | 4 | 1 | 1 | 1 | 1 | 1 | 1 | 1 | 1 | 1 |
| 1447 | 37 | 1 | 160 | 50 | 4 | 2 | 1 | 2 | 5 | 1 | 1 | 1 | 1 | 1 | 1 | 1 | 1 | 2 |
| 1448 | 37 | 1 | 161 | 58 | 8 | 2 | 1 | 2 | 4 | 1 | 1 | 1 | 1 | 1 | 1 | 1 | 2 | 1 |
| 1449 | 37 | 1 | 163 | 65 | 8 | 1 | 1 | 2 | 3 | 3 | 1 | 1 | 1 | 1 | 1 | 1 | 3 | 1 |
| 1450 | 37 | 1 | 168 | 65 | 3 | 2 | 1 | 1 | 5 | 3 | 1 | 1 | 1 | 1 | 1 | 1 | 1 | 1 |
| 1451 | 37 | 1 | 178 | 78 | 3 | 2 | 1 | 1 | 5 | 2 | 1 | 1 | 2 | 1 | 1 | 1 | 1 | 1 |
| 1452 | 37 | 1 | 178 | 76 | 4 | 2 | 1 | 1 | 5 | 4 | 1 | 1 | 2 | 1 | 1 | 1 | 1 | 1 |
| 1453 | 38 | 2 | 150 | 70 | 8 | 2 | 1 | 1 | 4 | 4 | 1 | 1 | 2 | 1 | 1 | 1 | 1 | 1 |
| 1454 | 38 | 2 | 150 | 55 | 8 | 2 | 1 | 1 | 4 | 4 | 1 | 1 | 2 | 1 | 1 | 1 | 1 | 1 |
| 1455 | 38 | 2 | 151 | 51.7 | 4 | 4 | 1 | 1 | 2 | 3 | 1 | 1 | 1 | 1 | 1 | 1 | 1 | 1 |
| 1456 | 38 | 2 | 154 | 59 | 8 | 2 | 1 | 1 | 2 | 1 | 2 | 1 | 1 | 1 | 1 | 1 | 1 | 1 |
| 1457 | 38 | 2 | 154 | 43 | 8 | 2 | 1 | 1 | 4 | 4 | 1 | 1 | 1 | 1 | 1 | 1 | 1 | 1 |
| 1458 | 38 | 2 | 155 | 50 | 8 | 2 | 1 | 1 | 5 | 4 | 1 | 1 | 1 | 1 | 1 | 1 | 1 | 1 |
| 1459 | 38 | 2 | 155 | 48 | 2 | 2 | 1 | 1 | 5 | 1 | 1 | 1 | 2 | 1 | 1 | 1 | 1 | 1 |
| 1460 | 38 | 2 | 155 | 45 | 8 | 2 | 1 | 1 | 5 | 3 | 1 | 1 | 2 | 1 | 1 | 1 | 1 | 1 |
| 1461 | 38 | 2 | 156 | 70 | 8 | 2 | 1 | 2 | 5 | 2 | 1 | 1 | 1 | 1 | 1 | 1 | 1 | 1 |
| 1462 | 38 | 2 | 156 | 70 | 3 | 2 | 1 | 1 | 4 | 4 | 1 | 1 | 2 | 1 | 1 | 1 | 1 | 1 |
| 1463 | 38 | 2 | 156 | 49.2 | 8 | 2 | 1 | 1 | 4 | 3 | 1 | 1 | 2 | 1 | 1 | 1 | 1 | 1 |
| 1464 | 38 | 2 | 157 | 65 | 8 | 2 | 2 | 1 | 4 | 3 | 1 | 1 | 1 | 1 | 1 | 1 | 1 | 1 |
| 1465 | 38 | 2 | 157 | 58 | 8 | 2 | 1 | 1 | 4 | 3 | 1 | 1 | 2 | 1 | 1 | 1 | 1 | 1 |
| 1466 | 38 | 2 | 158 | 56 | 4 | 2 | 1 | 1 | 4 | 3 | 1 | 1 | 2 | 1 | 1 | 1 | 1 | 1 |
| 1467 | 38 | 2 | 158 | 48 | 8 | 2 | 1 | 1 | 5 | 3 | 1 | 1 | 2 | 1 | 1 | 1 | 1 | 1 |
| 1468 | 38 | 2 | 158 | 45 | 1 | 2 | 1 | 1 | 5 | 1 | 1 | 1 | 2 | 1 | 1 | 1 | 1 | 1 |
| 1469 | 38 | 2 | 159 | 55 | 8 | 2 | 1 | 1 | 3 | 4 | 1 | 1 | 1 | 1 | 1 | 1 | 1 | 1 |
| 1470 | 38 | 2 | 160 | 90 | 8 | 2 | 1 | 1 | 5 | 1 | 1 | 1 | 1 | 1 | 1 | 1 | 1 | 1 |
| 1471 | 38 | 2 | 160 | 75 | 2 | 2 | 1 | 1 | 5 | 2 | 1 | 1 | 1 | 1 | 1 | 1 | 1 | 1 |
| 1472 | 38 | 2 | 160 | 65 | 8 | 2 | 1 | 1 | 5 | 4 | 1 | 1 | 2 | 1 | 1 | 1 | 1 | 1 |
| 1473 | 38 | 2 | 160 | 61 | 8 | 2 | 1 | 1 | 3 | 4 | 1 | 1 | 1 | 1 | 2 | 1 | 1 | 1 |
| 1474 | 38 | 2 | 160 | 55 | 8 | 3 | 1 | 1 | 5 | 4 | 1 | 1 | 1 | 1 | 1 | 1 | 1 | 1 |
| 1475 | 38 | 2 | 160 | 54 | 8 | 2 | 1 | 1 | 5 | 3 | 1 | 1 | 1 | 1 | 1 | 1 | 1 | 1 |
| 1476 | 38 | 2 | 160 | 52 | 8 | 2 | 1 | 1 | 4 | 4 | 1 | 1 | 1 | 1 | 1 | 1 | 1 | 1 |
| 1477 | 38 | 2 | 161 | 55 | 6 | 2 | 1 | 1 | 5 | 4 | 1 | 1 | 2 | 1 | 2 | 1 | 1 | 1 |
| 1478 | 38 | 2 | 162 | 65 | 8 | 2 | 1 | 1 | 5 | 4 | 1 | 1 | 2 | 1 | 1 | 1 | 1 | 1 |
| 1479 | 38 | 2 | 163 | 67.5 | 8 | 2 | 1 | 1 | 3 | 1 | 1 | 1 | 1 | 1 | 1 | 1 | 1 | 1 |
| 1480 | 38 | 2 | 163 | 64 | 8 | 2 | 2 | 1 | 3 | 3 | 1 | 1 | 1 | 1 | 1 | 1 | 1 | 1 |
| 1481 | 38 | 2 | 163 | 54 | 3 | 2 | 1 | 1 | 5 | 4 | 1 | 1 | 2 | 1 | 1 | 1 | 1 | 1 |
| 1482 | 38 | 2 | 164 | 49 | 8 | 2 | 1 | 1 | 3 | 1 | 1 | 1 | 2 | 1 | 1 | 1 | 1 | 1 |
| 1483 | 38 | 2 | 167 | 45 | 2 | 2 | 1 | 1 | 5 | 3 | 1 | 1 | 1 | 1 | 1 | 1 | 1 | 1 |
| 1484 | 38 | 2 | 172 | 65 | 2 | 2 | 1 | 1 | 5 | 2 | 1 | 1 | 2 | 1 | 1 | 1 | 1 | 1 |
| 1485 | 38 | 1 | 157 | 73.9 | 8 | 2 | 1 | 1 | 4 | 1 | 1 | 1 | 1 | 1 | 1 | 1 | 1 | 1 |
| 1486 | 38 | 1 | 160 | 95 | 8 | 1 | 2 | 1 | 4 | 4 | 3 | 1 | 1 | 1 | 1 | 1 | 1 | 2 |
| 1487 | 38 | 1 | 163 | 70 | 2 | 2 | 1 | 1 | 4 | 1 | 1 | 1 | 2 | 2 | 1 | 1 | 1 | 2 |
| 1488 | 38 | 1 | 170 | 68 | 3 | 2 | 1 | 2 | 5 | 2 | 1 | 1 | 1 | 1 | 2 | 1 | 1 | 1 |
| 1489 | 38 | 1 | 170 | 62 | 2 | 2 | 1 | 1 | 5 | 4 | 1 | 1 | 1 | 1 | 1 | 1 | 1 | 1 |
| 1490 | 38 | 1 | 172 | 84 | 3 | 2 | 1 | 1 | 5 | 3 | 1 | 1 | 1 | 1 | 1 | 1 | 1 | 1 |
| 1491 | 38 | 1 | 173 | 65 | 8 | 2 | 1 | 1 | 4 | 2 | 1 | 1 | 2 | 1 | 1 | 1 | 1 | 1 |
| 1492 | 38 | 1 | 176 | 75 | 2 | 2 | 1 | 2 | 5 | 3 | 1 | 1 | 1 | 2 | 1 | 1 | 1 | 2 |
| 1493 | 38 | 1 | 181 | 78.5 | 1 | 2 | 1 | 1 | 5 | 2 | 1 | 1 | 1 | 1 | 1 | 1 | 2 | 1 |
| 1494 | 39 | 2 | 150 | 49 | 1 | 2 | 1 | 1 | 5 | 2 | 1 | 1 | 1 | 1 | 1 | 1 | 1 | 1 |
| 1495 | 39 | 2 | 153 | 55 | 1 | 2 | 1 | 1 | 4 | 2 | 1 | 1 | 2 | 1 | 1 | 1 | 1 | 1 |
| 1496 | 39 | 2 | 153 | 51 | 4 | 2 | 2 | 1 | 5 | 1 | 1 | 1 | 1 | 1 | 1 | 1 | 1 | 1 |
| 1497 | 39 | 2 | 154 | 42 | 4 | 4 | 1 | 1 | 4 | 1 | 1 | 1 | 1 | 1 | 1 | 1 | 1 | 1 |
| 1498 | 39 | 2 | 155 | 66.5 | 4 | 2 | 1 | 1 | 5 | 3 | 1 | 1 | 2 | 1 | 1 | 1 | 1 | 1 |
| 1499 | 39 | 2 | 155 | 60 | 4 | 2 | 1 | 1 | 3 | 4 | 1 | 1 | 2 | 1 | 1 | 1 | 1 | 1 |
| 1500 | 39 | 2 | 155 | 55 | 8 | 2 | 1 | 1 | 5 | 3 | 1 | 1 | 1 | 1 | 1 | 1 | 1 | 1 |
| 1501 | 39 | 2 | 155 | 53 | 4 | 2 | 1 | 1 | 5 | 3 | 1 | 1 | 2 | 1 | 1 | 1 | 1 | 1 |
| 1502 | 39 | 2 | 155 | 46 | 8 | 2 | 1 | 1 | 4 | 2 | 1 | 1 | 2 | 1 | 1 | 1 | 1 | 1 |
| 1503 | 39 | 2 | 155 | 44 | 8 | 2 | 1 | 1 | 3 | 4 | 1 | 1 | 1 | 1 | 1 | 1 | 1 | 1 |
| 1504 | 39 | 2 | 156 | 55 | 2 | 2 | 1 | 1 | 5 | 4 | 1 | 1 | 2 | 1 | 1 | 1 | 1 | 1 |
| 1505 | 39 | 2 | 156 | 53 | 3 | 2 | 1 | 1 | 5 | 4 | 1 | 1 | 1 | 1 | 1 | 1 | 1 | 1 |
| 1506 | 39 | 2 | 156 | 45 | 4 | 2 | 1 | 1 | 3 | 3 | 1 | 1 | 1 | 1 | 1 | 1 | 1 | 1 |
| 1507 | 39 | 2 | 157 | 55 | 8 | 2 | 1 | 1 | 3 | 3 | 1 | 1 | 1 | 1 | 1 | 1 | 1 | 1 |
| 1508 | 39 | 2 | 157 | 46 | 8 | 2 | 1 | 1 | 3 | 3 | 1 | 1 | 1 | 1 | 1 | 1 | 1 | 1 |
| 1509 | 39 | 2 | 158 | 55 | 8 | 2 | 1 | 1 | 4 | 1 | 1 | 1 | 2 | 1 | 1 | 1 | 1 | 1 |
| 1510 | 39 | 2 | 158 | 44 | 8 | 2 | 1 | 1 | 4 | 4 | 1 | 1 | 1 | 1 | 1 | 1 | 1 | 1 |
| 1511 | 39 | 2 | 158 | 38.5 | 8 | 1 | 1 | 1 | 5 | 3 | 1 | 1 | 2 | 1 | 1 | 1 | 1 | 1 |
| 1512 | 39 | 2 | 159 | 53.5 | 8 | 2 | 1 | 1 | 3 | 4 | 1 | 1 | 2 | 1 | 2 | 1 | 1 | 1 |
| 1513 | 39 | 2 | 160 | 55 | 8 | 2 | 1 | 1 | 3 | 4 | 1 | 1 | 1 | 1 | 1 | 1 | 1 | 1 |
| 1514 | 39 | 2 | 160 | 47 | 4 | 2 | 2 | 1 | 5 | 3 | 1 | 1 | 1 | 1 | 1 | 1 | 1 | 1 |
| 1515 | 39 | 2 | 161 | 60 | 8 | 2 | 1 | 1 | 3 | 4 | 2 | 1 | 1 | 1 | 2 | 1 | 1 | 2 |
| 1516 | 39 | 2 | 163 | 59 | 8 | 2 | 1 | 1 | 5 | 2 | 1 | 1 | 2 | 1 | 1 | 1 | 1 | 1 |
| 1517 | 39 | 2 | 163 | 56 | 8 | 2 | 1 | 1 | 4 | 1 | 2 | 1 | 2 | 1 | 2 | 1 | 1 | 1 |
| 1518 | 39 | 2 | 164 | 70 | 8 | 2 | 1 | 1 | 4 | 3 | 1 | 1 | 1 | 1 | 1 | 1 | 1 | 1 |
| 1519 | 39 | 2 | 165 | 50 | 8 | 2 | 1 | 1 | 3 | 4 | 1 | 1 | 2 | 1 | 1 | 1 | 1 | 1 |
| 1520 | 39 | 2 | 165 | 47 | 4 | 1 | 1 | 1 | 4 | 3 | 1 | 1 | 1 | 1 | 1 | 1 | 1 | 1 |
| 1521 | 39 | 2 | 171 | 61.6 | 8 | 2 | 1 | 1 | 4 | 3 | 1 | 1 | 1 | 1 | 1 | 1 | 1 | 2 |
| 1522 | 39 | 1 | 163 | 62 | 8 | 1 | 1 | 1 | 2 | 4 | 1 | 1 | 1 | 1 | 1 | 1 | 1 | 1 |
| 1523 | 39 | 1 | 164 | 61.5 | 3 | 2 | 1 | 1 | 5 | 4 | 1 | 1 | 2 | 1 | 1 | 1 | 1 | 1 |
| 1524 | 39 | 1 | 165 | 51 | 2 | 2 | 1 | 1 | 2 | 2 | 1 | 1 | 2 | 1 | 1 | 1 | 1 | 1 |
| 1525 | 39 | 1 | 166 | 65 | 5 | 2 | 1 | 1 | 6 | 2 | 1 | 1 | 2 | 1 | 1 | 1 | 1 | 1 |
| 1526 | 39 | 1 | 167 | 57 | 3 | 2 | 1 | 2 | 5 | 2 | 1 | 1 | 2 | 1 | 1 | 1 | 3 | 1 |
| 1527 | 39 | 1 | 168 | 75 | 8 | 2 | 1 | 1 | 3 | 4 | 1 | 1 | 1 | 1 | 1 | 1 | 1 | 1 |
| 1528 | 39 | 1 | 170 | 75 | 8 | 2 | 1 | 1 | 5 | 1 | 1 | 1 | 2 | 1 | 1 | 1 | 1 | 1 |
| 1529 | 39 | 1 | 170 | 60 | 2 | 2 | 1 | 1 | 5 | 2 | 1 | 1 | 1 | 2 | 1 | 1 | 1 | 2 |
| 1530 | 39 | 1 | 170.5 | 77.2 | 4 | 1 | 1 | 1 | 5 | 4 | 1 | 1 | 1 | 1 | 1 | 1 | 1 | 1 |
| 1531 | 39 | 1 | 172 | 60 | 1 | 2 | 1 | 1 | 3 | 4 | 1 | 1 | 2 | 1 | 1 | 1 | 1 | 1 |
| 1532 | 39 | 1 | 173 | 80 | 2 | 2 | 1 | 2 | 5 | 3 | 1 | 1 | 1 | 1 | 1 | 1 | 1 | 1 |
| 1533 | 39 | 1 | 173 | 68 | 3 | 2 | 1 | 1 | 5 | 2 | 1 | 1 | 2 | 1 | 1 | 1 | 1 | 1 |
| 1534 | 39 | 1 | 173.5 | 76 | 2 | 2 | 1 | 2 | 5 | 2 | 1 | 1 | 1 | 1 | 1 | 1 | 3 | 1 |
| 1535 | 39 | 1 | 179 | 74 | 8 | 2 | 1 | 3 | 5 | 2 | 1 | 1 | 1 | 1 | 1 | 1 | 3 | 1 |
| 1536 | 39 | 1 | 179.5 | 68.2 | 8 | 2 | 1 | 1 | 5 | 4 | 1 | 1 | 1 | 1 | 1 | 1 | 1 | 2 |
| 1537 | 40 | 2 | 147 | 40 | 8 | 3 | 1 | 1 | 4 | 4 | 1 | 1 | 1 | 1 | 1 | 1 | 1 | 1 |
| 1538 | 40 | 2 | 150 | 50 | 8 | 2 | 1 | 1 | 4 | 4 | 1 | 1 | 1 | 2 | 1 | 1 | 1 | 2 |
| 1539 | 40 | 2 | 150 | 49 | 8 | 2 | 1 | 1 | 3 | 3 | 1 | 1 | 1 | 1 | 1 | 1 | 1 | 1 |
| 1540 | 40 | 2 | 150 | 42 | 8 | 2 | 1 | 1 | 5 | 3 | 1 | 1 | 1 | 1 | 1 | 1 | 1 | 1 |
| 1541 | 40 | 2 | 154 | 52 | 8 | 2 | 1 | 1 | 3 | 1 | 1 | 1 | 1 | 1 | 2 | 1 | 1 | 1 |
| 1542 | 40 | 2 | 155 | 51.5 | 4 | 2 | 1 | 1 | 4 | 3 | 1 | 1 | 2 | 1 | 1 | 1 | 1 | 1 |
| 1543 | 40 | 2 | 155 | 45 | 8 | 2 | 1 | 1 | 3 | 3 | 1 | 1 | 1 | 1 | 1 | 1 | 1 | 1 |
| 1544 | 40 | 2 | 156 | 74 | 3 | 2 | 1 | 1 | 5 | 2 | 1 | 1 | 1 | 1 | 1 | 1 | 1 | 1 |
| 1545 | 40 | 2 | 156 | 65 | 3 | 2 | 1 | 1 | 5 | 4 | 1 | 1 | 1 | 1 | 1 | 1 | 1 | 1 |
| 1546 | 40 | 2 | 156 | 62 | 3 | 2 | 1 | 1 | 4 | 4 | 1 | 1 | 2 | 1 | 1 | 1 | 1 | 1 |
| 1547 | 40 | 2 | 157 | 74 | 4 | 2 | 1 | 1 | 3 | 4 | 1 | 1 | 1 | 1 | 1 | 1 | 1 | 1 |
| 1548 | 40 | 2 | 157 | 55 | 8 | 2 | 1 | 1 | 6 | 4 | 1 | 1 | 1 | 1 | 1 | 1 | 1 | 1 |
| 1549 | 40 | 2 | 157 | 54 | 3 | 2 | 1 | 1 | 4 | 2 | 1 | 1 | 2 | 1 | 2 | 1 | 1 | 1 |
| 1550 | 40 | 2 | 158 | 75 | 8 | 2 | 1 | 1 | 6 | 4 | 1 | 1 | 2 | 1 | 1 | 1 | 1 | 1 |
| 1551 | 40 | 2 | 158 | 65 | 8 | 2 | 1 | 1 | 5 | 2 | 1 | 1 | 1 | 1 | 1 | 1 | 1 | 1 |
| 1552 | 40 | 2 | 158 | 58 | 3 | 2 | 1 | 1 | 5 | 2 | 1 | 1 | 1 | 1 | 1 | 1 | 1 | 1 |
| 1553 | 40 | 2 | 158 | 58 | 8 | 2 | 1 | 1 | 4 | 3 | 1 | 1 | 1 | 1 | 1 | 1 | 1 | 1 |
| 1554 | 40 | 2 | 158 | 57 | 4 | 2 | 1 | 1 | 4 | 3 | 1 | 1 | 2 | 1 | 1 | 1 | 1 | 1 |
| 1555 | 40 | 2 | 158 | 55 | 2 | 2 | 1 | 1 | 5 | 2 | 1 | 1 | 1 | 1 | 1 | 1 | 1 | 1 |
| 1556 | 40 | 2 | 158 | 50 | 8 | 2 | 1 | 1 | 4 | 1 | 1 | 1 | 2 | 1 | 1 | 1 | 1 | 1 |
| 1557 | 40 | 2 | 158 | 46 | 3 | 2 | 1 | 2 | 4 | 3 | 1 | 1 | 2 | 1 | 1 | 1 | 1 | 1 |
| 1558 | 40 | 2 | 159 | 55 | 3 | 2 | 1 | 1 | 4 | 4 | 1 | 1 | 1 | 1 | 1 | 1 | 1 | 1 |
| 1559 | 40 | 2 | 159 | 53 | 8 | 2 | 1 | 1 | 4 | 2 | 1 | 1 | 2 | 1 | 1 | 1 | 1 | 1 |
| 1560 | 40 | 2 | 159 | 53 | 2 | 2 | 1 | 1 | 5 | 4 | 1 | 1 | 2 | 1 | 1 | 1 | 1 | 1 |
| 1561 | 40 | 2 | 160 | 70 | 8 | 2 | 1 | 1 | 6 | 4 | 1 | 1 | 1 | 1 | 1 | 1 | 1 | 1 |
| 1562 | 40 | 2 | 160 | 65 | 8 | 2 | 1 | 1 | 4 | 4 | 1 | 1 | 2 | 1 | 1 | 1 | 1 | 2 |
| 1563 | 40 | 2 | 160 | 55 | 4 | 2 | 1 | 1 | 3 | 4 | 3 | 1 | 1 | 1 | 1 | 1 | 1 | 1 |
| 1564 | 40 | 2 | 160 | 51 | 4 | 2 | 1 | 1 | 4 | 4 | 1 | 1 | 1 | 1 | 1 | 1 | 1 | 1 |
| 1565 | 40 | 2 | 160 | 50 | 8 | 2 | 1 | 1 | 3 | 4 | 1 | 1 | 1 | 1 | 1 | 1 | 1 | 1 |
| 1566 | 40 | 2 | 160 | 48 | 8 | 2 | 1 | 1 | 4 | 4 | 1 | 1 | 1 | 1 | 1 | 1 | 1 | 1 |
| 1567 | 40 | 2 | 161 | 58 | 8 | 1 | 2 | 1 | 4 | 3 | 1 | 1 | 1 | 1 | 1 | 1 | 1 | 2 |
| 1568 | 40 | 2 | 161 | 52 | 1 | 2 | 1 | 1 | 5 | 4 | 1 | 1 | 1 | 1 | 1 | 1 | 1 | 1 |
| 1569 | 40 | 2 | 163 | 65 | 8 | 2 | 1 | 1 | 3 | 1 | 2 | 1 | 2 | 2 | 1 | 1 | 1 | 1 |
| 1570 | 40 | 2 | 163 | 64 | 8 | 2 | 1 | 1 | 4 | 3 | 1 | 1 | 2 | 1 | 1 | 1 | 1 | 1 |
| 1571 | 40 | 2 | 163 | 54 | 8 | 2 | 1 | 1 | 4 | 1 | 1 | 1 | 1 | 1 | 1 | 1 | 1 | 1 |
| 1572 | 40 | 2 | 165 | 65 | 8 | 2 | 1 | 1 | 4 | 3 | 1 | 1 | 1 | 1 | 1 | 1 | 1 | 1 |
| 1573 | 40 | 2 | 165 | 65 | 4 | 2 | 1 | 1 | 5 | 3 | 1 | 1 | 1 | 1 | 1 | 1 | 1 | 1 |
| 1574 | 40 | 2 | 165 | 63.5 | 8 | 2 | 1 | 2 | 5 | 3 | 2 | 1 | 1 | 1 | 1 | 1 | 1 | 1 |
| 1575 | 40 | 2 | 167 | 52.6 | 8 | 2 | 1 | 1 | 4 | 1 | 1 | 1 | 2 | 1 | 1 | 1 | 1 | 1 |
| 1576 | 40 | 1 | 165 | 50.9 | 2 | 2 | 1 | 2 | 5 | 3 | 1 | 1 | 2 | 1 | 1 | 1 | 1 | 1 |
| 1577 | 40 | 1 | 167 | 78 | 8 | 2 | 1 | 1 | 4 | 4 | 1 | 1 | 2 | 1 | 1 | 1 | 1 | 1 |
| 1578 | 40 | 1 | 168 | 80 | 1 | 2 | 1 | 1 | 5 | 4 | 1 | 1 | 2 | 1 | 1 | 1 | 1 | 1 |
| 1579 | 40 | 1 | 168 | 54 | 6 | 4 | 1 | 1 | 4 | 3 | 1 | 1 | 2 | 1 | 1 | 1 | 1 | 1 |
| 1580 | 40 | 1 | 170 | 60 | 8 | 2 | 1 | 1 | 6 | 3 | 1 | 1 | 1 | 1 | 1 | 1 | 1 | 1 |
| 1581 | 40 | 1 | 170 | 60 | 4 | 2 | 1 | 1 | 5 | 1 | 1 | 1 | 1 | 1 | 1 | 1 | 1 | 1 |
| 1582 | 40 | 1 | 172 | 64 | 6 | 2 | 1 | 1 | 3 | 1 | 1 | 1 | 1 | 2 | 1 | 1 | 1 | 2 |
| 1583 | 40 | 1 | 175 | 60 | 2 | 2 | 1 | 2 | 5 | 2 | 1 | 1 | 1 | 1 | 1 | 1 | 1 | 1 |
| 1584 | 40 | 1 | 177 | 85 | 3 | 2 | 1 | 1 | 5 | 2 | 1 | 1 | 1 | 1 | 1 | 1 | 1 | 1 |
| 1585 | 40 | 1 | 180 | 84 | 3 | 2 | 1 | 1 | 5 | 2 | 1 | 1 | 2 | 2 | 2 | 1 | 1 | 1 |
| 1586 | 41 | 2 | 147 | 57.5 | 8 | 2 | 1 | 1 | 4 | 3 | 1 | 1 | 1 | 1 | 1 | 1 | 1 | 1 |
| 1587 | 41 | 2 | 150 | 56.5 | 8 | 2 | 1 | 1 | 4 | 4 | 2 | 1 | 1 | 2 | 1 | 1 | 1 | 2 |
| 1588 | 41 | 2 | 150 | 50 | 4 | 2 | 2 | 2 | 4 | 3 | 1 | 1 | 2 | 1 | 1 | 1 | 1 | 1 |
| 1589 | 41 | 2 | 150 | 44 | 8 | 2 | 1 | 1 | 4 | 3 | 1 | 1 | 1 | 1 | 1 | 1 | 1 | 1 |
| 1590 | 41 | 2 | 153 | 61.5 | 2 | 2 | 1 | 1 | 5 | 4 | 1 | 1 | 1 | 1 | 1 | 1 | 1 | 1 |
| 1591 | 41 | 2 | 153 | 56.2 | 4 | 2 | 1 | 1 | 4 | 3 | 2 | 1 | 1 | 1 | 1 | 1 | 1 | 2 |
| 1592 | 41 | 2 | 154 | 53 | 8 | 2 | 1 | 1 | 5 | 1 | 2 | 1 | 1 | 2 | 1 | 1 | 1 | 2 |
| 1593 | 41 | 2 | 154 | 53 | 6 | 2 | 1 | 1 | 4 | 2 | 1 | 1 | 1 | 1 | 1 | 1 | 1 | 1 |
| 1594 | 41 | 2 | 155 | 60 | 4 | 2 | 2 | 1 | 5 | 2 | 1 | 1 | 2 | 1 | 1 | 1 | 1 | 1 |
| 1595 | 41 | 2 | 155 | 59 | 3 | 2 | 1 | 1 | 4 | 3 | 1 | 1 | 2 | 1 | 1 | 1 | 1 | 1 |
| 1596 | 41 | 2 | 155 | 53 | 8 | 2 | 1 | 1 | 5 | 2 | 1 | 1 | 2 | 1 | 1 | 1 | 1 | 1 |
| 1597 | 41 | 2 | 155 | 48 | 8 | 2 | 1 | 1 | 3 | 4 | 1 | 1 | 1 | 1 | 1 | 1 | 1 | 1 |
| 1598 | 41 | 2 | 155 | 45 | 8 | 2 | 1 | 1 | 3 | 4 | 1 | 1 | 2 | 1 | 1 | 1 | 1 | 1 |
| 1599 | 41 | 2 | 155 | 42.5 | 8 | 2 | 1 | 1 | 4 | 4 | 1 | 1 | 1 | 1 | 1 | 1 | 1 | 1 |
| 1600 | 41 | 2 | 156 | 55 | 4 | 2 | 1 | 1 | 4 | 3 | 1 | 1 | 1 | 1 | 1 | 1 | 1 | 1 |
| 1601 | 41 | 2 | 156 | 47.5 | 4 | 2 | 1 | 1 | 4 | 4 | 1 | 1 | 1 | 1 | 1 | 1 | 1 | 1 |
| 1602 | 41 | 2 | 157 | 50 | 4 | 2 | 1 | 1 | 5 | 4 | 1 | 1 | 1 | 1 | 1 | 1 | 1 | 2 |
| 1603 | 41 | 2 | 158 | 75 | 8 | 2 | 1 | 1 | 3 | 3 | 1 | 1 | 2 | 1 | 2 | 1 | 1 | 2 |
| 1604 | 41 | 2 | 158 | 60 | 8 | 2 | 1 | 1 | 2 | 3 | 1 | 1 | 1 | 1 | 1 | 1 | 1 | 1 |
| 1605 | 41 | 2 | 158 | 50 | 2 | 2 | 1 | 2 | 5 | 2 | 1 | 1 | 2 | 1 | 1 | 1 | 1 | 1 |
| 1606 | 41 | 2 | 158 | 48.5 | 8 | 2 | 1 | 1 | 2 | 2 | 1 | 1 | 1 | 1 | 1 | 1 | 1 | 1 |
| 1607 | 41 | 2 | 158 | 47 | 8 | 2 | 1 | 1 | 3 | 4 | 1 | 1 | 2 | 1 | 1 | 1 | 1 | 1 |
| 1608 | 41 | 2 | 158 | 46.5 | 4 | 2 | 1 | 1 | 5 | 3 | 1 | 1 | 2 | 1 | 1 | 1 | 1 | 1 |
| 1609 | 41 | 2 | 160 | 65 | 8 | 2 | 1 | 1 | 4 | 3 | 1 | 1 | 1 | 1 | 1 | 1 | 1 | 1 |
| 1610 | 41 | 2 | 160 | 62 | 8 | 2 | 1 | 1 | 5 | 3 | 2 | 1 | 1 | 1 | 2 | 1 | 1 | 2 |
| 1611 | 41 | 2 | 160 | 60 | 8 | 2 | 1 | 1 | 4 | 4 | 1 | 1 | 1 | 1 | 1 | 1 | 1 | 1 |
| 1612 | 41 | 2 | 160 | 58 | 4 | 2 | 1 | 1 | 3 | 4 | 1 | 1 | 1 | 1 | 1 | 1 | 1 | 1 |
| 1613 | 41 | 2 | 160 | 58 | 1 | 2 | 1 | 1 | 5 | 1 | 1 | 1 | 2 | 1 | 1 | 1 | 1 | 1 |
| 1614 | 41 | 2 | 160 | 50 | 4 | 2 | 1 | 1 | 4 | 1 | 1 | 1 | 1 | 1 | 1 | 1 | 1 | 1 |
| 1615 | 41 | 2 | 160 | 49 | 8 | 2 | 1 | 1 | 4 | 4 | 1 | 1 | 2 | 1 | 1 | 1 | 1 | 1 |
| 1616 | 41 | 2 | 160 | 47 | 4 | 2 | 1 | 1 | 5 | 2 | 1 | 1 | 2 | 1 | 1 | 1 | 1 | 1 |
| 1617 | 41 | 2 | 161 | 64.5 | 8 | 2 | 1 | 1 | 5 | 3 | 1 | 1 | 1 | 1 | 1 | 1 | 1 | 1 |
| 1618 | 41 | 2 | 161.5 | 82.2 | 2 | 1 | 1 | 1 | 2 | 2 | 1 | 1 | 2 | 1 | 1 | 1 | 1 | 1 |
| 1619 | 41 | 2 | 162 | 70 | 8 | 2 | 1 | 2 | 3 | 3 | 1 | 1 | 2 | 1 | 1 | 1 | 1 | 2 |
| 1620 | 41 | 2 | 162 | 68 | 8 | 1 | 1 | 1 | 4 | 1 | 1 | 1 | 2 | 1 | 1 | 1 | 1 | 1 |
| 1621 | 41 | 2 | 162 | 57 | 3 | 2 | 2 | 1 | 4 | 2 | 1 | 1 | 2 | 1 | 1 | 1 | 1 | 1 |
| 1622 | 41 | 2 | 162 | 53 | 2 | 2 | 1 | 1 | 5 | 2 | 1 | 1 | 1 | 1 | 1 | 1 | 1 | 1 |
| 1623 | 41 | 2 | 163 | 65 | 8 | 2 | 2 | 1 | 2 | 4 | 1 | 1 | 1 | 1 | 1 | 1 | 1 | 1 |
| 1624 | 41 | 2 | 163 | 64 | 2 | 2 | 2 | 2 | 5 | 3 | 1 | 1 | 2 | 1 | 1 | 1 | 1 | 1 |
| 1625 | 41 | 2 | 163 | 60 | 8 | 2 | 1 | 1 | 3 | 1 | 1 | 1 | 1 | 2 | 1 | 1 | 1 | 2 |
| 1626 | 41 | 2 | 163 | 59 | 2 | 2 | 1 | 1 | 4 | 2 | 1 | 1 | 2 | 1 | 1 | 1 | 1 | 1 |
| 1627 | 41 | 2 | 163 | 55 | 8 | 2 | 1 | 1 | 5 | 4 | 1 | 1 | 1 | 1 | 1 | 1 | 1 | 2 |
| 1628 | 41 | 2 | 163 | 55 | 2 | 2 | 1 | 1 | 4 | 4 | 1 | 1 | 1 | 1 | 1 | 1 | 1 | 1 |
| 1629 | 41 | 2 | 165 | 57 | 8 | 2 | 1 | 1 | 5 | 4 | 1 | 1 | 2 | 1 | 1 | 1 | 1 | 2 |
| 1630 | 41 | 2 | 165 | 52.5 | 3 | 2 | 1 | 1 | 5 | 4 | 1 | 1 | 1 | 1 | 1 | 1 | 1 | 2 |
| 1631 | 41 | 2 | 165 | 52 | 3 | 2 | 1 | 1 | 5 | 3 | 1 | 1 | 1 | 1 | 1 | 1 | 1 | 1 |
| 1632 | 41 | 2 | 165.5 | 66 | 2 | 2 | 1 | 1 | 4 | 2 | 2 | 1 | 2 | 1 | 1 | 1 | 1 | 1 |
| 1633 | 41 | 2 | 168 | 65 | 8 | 3 | 1 | 1 | 4 | 4 | 1 | 1 | 1 | 1 | 1 | 1 | 1 | 1 |
| 1634 | 41 | 2 | 168 | 60 | 8 | 2 | 1 | 1 | 5 | 4 | 2 | 1 | 1 | 1 | 1 | 1 | 1 | 2 |
| 1635 | 41 | 1 | 170 | 65 | 8 | 4 | 1 | 1 | 4 | 2 | 1 | 1 | 1 | 1 | 1 | 1 | 1 | 1 |
| 1636 | 41 | 1 | 171 | 64 | 8 | 2 | 1 | 2 | 3 | 4 | 1 | 1 | 1 | 1 | 1 | 1 | 3 | 1 |
| 1637 | 41 | 1 | 172 | 84 | 4 | 2 | 1 | 1 | 4 | 3 | 1 | 1 | 1 | 1 | 1 | 1 | 3 | 1 |
| 1638 | 41 | 1 | 172 | 64 | 8 | 2 | 1 | 2 | 5 | 2 | 1 | 1 | 2 | 1 | 1 | 1 | 1 | 1 |
| 1639 | 41 | 1 | 173 | 57.5 | 6 | 2 | 1 | 1 | 4 | 3 | 1 | 1 | 1 | 1 | 1 | 1 | 1 | 1 |
| 1640 | 41 | 1 | 175 | 61 | 8 | 1 | 1 | 1 | 4 | 1 | 1 | 1 | 1 | 1 | 1 | 1 | 1 | 1 |
| 1641 | 41 | 1 | 176 | 53.2 | 2 | 2 | 1 | 1 | 4 | 1 | 1 | 1 | 2 | 1 | 1 | 1 | 1 | 1 |
| 1642 | 41 | 1 | 180 | 90 | 2 | 2 | 1 | 2 | 5 | 3 | 1 | 1 | 2 | 1 | 1 | 1 | 3 | 1 |
| 1643 | 42 | 2 | 150 | 61.8 | 8 | 2 | 2 | 1 | 3 | 2 | 1 | 1 | 1 | 1 | 2 | 1 | 1 | 1 |
| 1644 | 42 | 2 | 150 | 54 | 8 | 2 | 1 | 1 | 4 | 4 | 1 | 1 | 1 | 1 | 1 | 1 | 1 | 1 |
| 1645 | 42 | 2 | 150 | 50 | 4 | 2 | 1 | 1 | 4 | 4 | 1 | 1 | 1 | 1 | 1 | 1 | 1 | 1 |
| 1646 | 42 | 2 | 150.5 | 43.2 | 4 | 2 | 1 | 1 | 4 | 4 | 1 | 1 | 2 | 2 | 1 | 1 | 1 | 2 |
| 1647 | 42 | 2 | 152 | 48 | 8 | 2 | 1 | 1 | 4 | 4 | 1 | 1 | 1 | 1 | 1 | 1 | 1 | 1 |
| 1648 | 42 | 2 | 152 | 46.5 | 8 | 2 | 1 | 1 | 6 | 4 | 1 | 1 | 1 | 1 | 1 | 1 | 1 | 1 |
| 1649 | 42 | 2 | 152 | 40 | 8 | 2 | 1 | 1 | 6 | 4 | 1 | 1 | 2 | 1 | 1 | 1 | 1 | 1 |
| 1650 | 42 | 2 | 154 | 60 | 1 | 4 | 1 | 1 | 3 | 4 | 1 | 1 | 1 | 1 | 1 | 1 | 1 | 1 |
| 1651 | 42 | 2 | 154 | 58.5 | 5 | 2 | 1 | 1 | 3 | 3 | 1 | 1 | 1 | 1 | 2 | 1 | 1 | 1 |
| 1652 | 42 | 2 | 154 | 58 | 8 | 2 | 1 | 1 | 3 | 4 | 1 | 1 | 1 | 1 | 1 | 1 | 1 | 2 |
| 1653 | 42 | 2 | 154 | 55 | 8 | 2 | 1 | 1 | 5 | 4 | 1 | 1 | 1 | 1 | 1 | 1 | 1 | 1 |
| 1654 | 42 | 2 | 154 | 51 | 8 | 2 | 1 | 1 | 4 | 3 | 1 | 1 | 1 | 1 | 1 | 1 | 1 | 1 |
| 1655 | 42 | 2 | 154 | 50 | 3 | 2 | 1 | 1 | 5 | 4 | 1 | 1 | 2 | 1 | 1 | 1 | 1 | 1 |
| 1656 | 42 | 2 | 154.5 | 56.1 | 8 | 2 | 1 | 1 | 3 | 1 | 1 | 1 | 2 | 1 | 1 | 1 | 1 | 1 |
| 1657 | 42 | 2 | 155 | 65 | 8 | 1 | 1 | 1 | 4 | 3 | 1 | 1 | 1 | 1 | 1 | 1 | 1 | 1 |
| 1658 | 42 | 2 | 155 | 55 | 8 | 2 | 1 | 1 | 3 | 1 | 1 | 1 | 1 | 1 | 1 | 1 | 1 | 1 |
| 1659 | 42 | 2 | 155 | 52 | 8 | 2 | 1 | 2 | 5 | 3 | 1 | 1 | 2 | 1 | 1 | 1 | 1 | 1 |
| 1660 | 42 | 2 | 156 | 54 | 8 | 2 | 1 | 1 | 5 | 4 | 1 | 1 | 2 | 1 | 1 | 1 | 1 | 1 |
| 1661 | 42 | 2 | 156 | 52 | 8 | 1 | 1 | 1 | 4 | 2 | 1 | 1 | 1 | 1 | 1 | 1 | 1 | 1 |
| 1662 | 42 | 2 | 156 | 51 | 8 | 2 | 1 | 1 | 4 | 4 | 1 | 1 | 1 | 1 | 1 | 1 | 1 | 1 |
| 1663 | 42 | 2 | 156 | 48 | 4 | 2 | 1 | 1 | 4 | 2 | 1 | 1 | 2 | 1 | 1 | 1 | 1 | 1 |
| 1664 | 42 | 2 | 157 | 53 | 8 | 2 | 1 | 1 | 4 | 3 | 1 | 1 | 1 | 1 | 1 | 1 | 1 | 1 |
| 1665 | 42 | 2 | 157 | 52 | 8 | 2 | 1 | 1 | 5 | 4 | 1 | 1 | 1 | 1 | 1 | 1 | 1 | 1 |
| 1666 | 42 | 2 | 157 | 47 | 8 | 2 | 1 | 2 | 4 | 2 | 1 | 1 | 2 | 1 | 1 | 1 | 1 | 1 |
| 1667 | 42 | 2 | 158 | 70 | 6 | 2 | 1 | 1 | 4 | 4 | 1 | 1 | 1 | 1 | 1 | 1 | 1 | 1 |
| 1668 | 42 | 2 | 158 | 62.5 | 4 | 2 | 1 | 1 | 4 | 3 | 1 | 1 | 1 | 1 | 1 | 1 | 1 | 1 |
| 1669 | 42 | 2 | 158 | 59.5 | 2 | 2 | 1 | 1 | 4 | 2 | 1 | 1 | 2 | 1 | 1 | 1 | 1 | 1 |
| 1670 | 42 | 2 | 158 | 57.5 | 8 | 2 | 1 | 1 | 5 | 1 | 1 | 1 | 1 | 1 | 1 | 1 | 1 | 1 |
| 1671 | 42 | 2 | 158 | 55 | 8 | 2 | 1 | 1 | 6 | 4 | 1 | 1 | 1 | 1 | 1 | 1 | 1 | 1 |
| 1672 | 42 | 2 | 158 | 55 | 8 | 2 | 1 | 1 | 6 | 3 | 1 | 1 | 2 | 1 | 1 | 1 | 1 | 1 |
| 1673 | 42 | 2 | 158 | 54 | 4 | 2 | 1 | 1 | 4 | 4 | 1 | 1 | 1 | 2 | 1 | 1 | 1 | 2 |
| 1674 | 42 | 2 | 158 | 53 | 6 | 2 | 1 | 1 | 4 | 4 | 1 | 1 | 2 | 1 | 1 | 1 | 1 | 1 |
| 1675 | 42 | 2 | 158 | 52 | 8 | 2 | 1 | 1 | 3 | 2 | 1 | 1 | 1 | 1 | 1 | 1 | 1 | 1 |
| 1676 | 42 | 2 | 158 | 49 | 1 | 2 | 1 | 1 | 4 | 1 | 1 | 1 | 2 | 1 | 1 | 1 | 1 | 1 |
| 1677 | 42 | 2 | 159 | 65 | 8 | 2 | 1 | 1 | 6 | 4 | 1 | 1 | 2 | 1 | 1 | 1 | 1 | 1 |
| 1678 | 42 | 2 | 160 | 58 | 8 | 2 | 1 | 1 | 5 | 3 | 1 | 1 | 1 | 1 | 1 | 1 | 1 | 1 |
| 1679 | 42 | 2 | 160 | 56 | 8 | 2 | 1 | 1 | 4 | 4 | 1 | 1 | 2 | 1 | 1 | 1 | 1 | 2 |
| 1680 | 42 | 2 | 160 | 56 | 4 | 2 | 1 | 1 | 4 | 4 | 1 | 1 | 1 | 1 | 1 | 1 | 1 | 1 |
| 1681 | 42 | 2 | 160 | 55 | 3 | 2 | 1 | 1 | 5 | 3 | 1 | 1 | 1 | 1 | 1 | 1 | 1 | 1 |
| 1682 | 42 | 2 | 160 | 47.5 | 3 | 2 | 1 | 1 | 5 | 2 | 1 | 1 | 1 | 1 | 1 | 1 | 1 | 1 |
| 1683 | 42 | 2 | 160 | 45 | 4 | 1 | 1 | 1 | 4 | 4 | 1 | 1 | 1 | 1 | 1 | 1 | 1 | 1 |
| 1684 | 42 | 2 | 160 | 45 | 3 | 2 | 1 | 1 | 5 | 2 | 1 | 1 | 2 | 1 | 1 | 1 | 1 | 1 |
| 1685 | 42 | 2 | 161 | 55 | 8 | 2 | 1 | 1 | 5 | 4 | 1 | 1 | 1 | 1 | 1 | 1 | 1 | 1 |
| 1686 | 42 | 2 | 161 | 46 | 4 | 2 | 1 | 1 | 4 | 3 | 1 | 1 | 2 | 1 | 1 | 1 | 1 | 1 |
| 1687 | 42 | 2 | 162 | 77 | 8 | 2 | 1 | 2 | 2 | 2 | 1 | 1 | 1 | 1 | 1 | 1 | 3 | 2 |
| 1688 | 42 | 2 | 162 | 60 | 8 | 2 | 1 | 1 | 3 | 4 | 1 | 1 | 1 | 1 | 1 | 1 | 1 | 1 |
| 1689 | 42 | 2 | 163 | 60 | 4 | 1 | 1 | 1 | 5 | 3 | 1 | 1 | 1 | 2 | 1 | 1 | 1 | 2 |
| 1690 | 42 | 2 | 163 | 50 | 4 | 2 | 1 | 1 | 5 | 3 | 1 | 1 | 1 | 1 | 1 | 1 | 1 | 1 |
| 1691 | 42 | 2 | 165 | 75 | 3 | 2 | 1 | 1 | 4 | 4 | 1 | 1 | 2 | 1 | 1 | 1 | 1 | 1 |
| 1692 | 42 | 2 | 165 | 57 | 8 | 2 | 1 | 1 | 4 | 2 | 2 | 1 | 1 | 1 | 1 | 1 | 1 | 1 |
| 1693 | 42 | 2 | 165 | 50 | 4 | 2 | 1 | 1 | 3 | 3 | 1 | 1 | 2 | 2 | 1 | 1 | 1 | 2 |
| 1694 | 42 | 2 | 166 | 70 | 3 | 2 | 1 | 1 | 4 | 4 | 1 | 1 | 1 | 1 | 1 | 1 | 1 | 1 |
| 1695 | 42 | 1 | 167 | 81 | 3 | 1 | 1 | 2 | 5 | 4 | 1 | 1 | 1 | 1 | 1 | 1 | 1 | 1 |
| 1696 | 42 | 1 | 167 | 70 | 8 | 2 | 1 | 1 | 5 | 4 | 1 | 1 | 1 | 1 | 1 | 1 | 1 | 1 |
| 1697 | 42 | 1 | 168 | 65 | 8 | 2 | 1 | 1 | 4 | 4 | 1 | 1 | 2 | 1 | 1 | 1 | 1 | 1 |
| 1698 | 42 | 1 | 168 | 57 | 8 | 2 | 1 | 1 | 3 | 3 | 1 | 1 | 1 | 1 | 1 | 1 | 1 | 1 |
| 1699 | 42 | 1 | 170 | 65 | 8 | 2 | 1 | 2 | 5 | 3 | 1 | 1 | 2 | 1 | 1 | 1 | 1 | 1 |
| 1700 | 42 | 1 | 170 | 60 | 4 | 2 | 2 | 1 | 3 | 1 | 1 | 2 | 1 | 1 | 1 | 1 | 1 | 1 |
| 1701 | 42 | 1 | 172 | 74.5 | 1 | 2 | 1 | 1 | 5 | 2 | 1 | 1 | 1 | 1 | 1 | 1 | 1 | 1 |
| 1702 | 42 | 1 | 172 | 73 | 4 | 2 | 1 | 1 | 5 | 2 | 2 | 1 | 2 | 1 | 1 | 1 | 1 | 1 |
| 1703 | 42 | 1 | 175 | 76 | 2 | 2 | 1 | 1 | 5 | 3 | 1 | 1 | 2 | 1 | 1 | 1 | 1 | 1 |
| 1704 | 42 | 1 | 175 | 72 | 8 | 2 | 1 | 2 | 5 | 1 | 1 | 1 | 1 | 1 | 1 | 1 | 2 | 1 |
| 1705 | 42 | 1 | 176 | 90 | 6 | 2 | 1 | 1 | 2 | 4 | 1 | 1 | 1 | 1 | 2 | 1 | 1 | 1 |
| 1706 | 42 | 1 | 176 | 66 | 4 | 2 | 1 | 1 | 4 | 3 | 1 | 1 | 1 | 1 | 1 | 1 | 1 | 1 |
| 1707 | 42 | 1 | 178 | 54 | 8 | 2 | 1 | 1 | 4 | 4 | 1 | 1 | 1 | 1 | 1 | 1 | 3 | 2 |
| 1708 | 43 | 2 | 145 | 45 | 8 | 2 | 1 | 1 | 5 | 3 | 1 | 1 | 2 | 1 | 1 | 1 | 1 | 1 |
| 1709 | 43 | 2 | 148 | 65 | 8 | 2 | 1 | 1 | 3 | 3 | 1 | 1 | 1 | 1 | 1 | 1 | 1 | 1 |
| 1710 | 43 | 2 | 148 | 48 | 2 | 2 | 1 | 1 | 5 | 3 | 1 | 1 | 1 | 1 | 1 | 1 | 1 | 1 |
| 1711 | 43 | 2 | 150 | 65 | 8 | 2 | 1 | 1 | 5 | 3 | 1 | 1 | 2 | 1 | 1 | 1 | 1 | 1 |
| 1712 | 43 | 2 | 150 | 46 | 8 | 2 | 1 | 1 | 3 | 4 | 1 | 1 | 1 | 1 | 1 | 1 | 1 | 1 |
| 1713 | 43 | 2 | 150 | 42 | 4 | 1 | 1 | 1 | 3 | 4 | 1 | 1 | 2 | 1 | 1 | 1 | 1 | 1 |
| 1714 | 43 | 2 | 152.5 | 56 | 3 | 2 | 1 | 1 | 5 | 4 | 1 | 1 | 1 | 1 | 2 | 1 | 1 | 1 |
| 1715 | 43 | 2 | 153 | 54 | 8 | 2 | 1 | 1 | 4 | 4 | 1 | 1 | 2 | 1 | 1 | 1 | 1 | 1 |
| 1716 | 43 | 2 | 153 | 52.5 | 8 | 2 | 1 | 1 | 4 | 3 | 1 | 1 | 1 | 1 | 1 | 1 | 1 | 1 |
| 1717 | 43 | 2 | 153 | 44 | 2 | 2 | 1 | 1 | 5 | 4 | 1 | 1 | 1 | 1 | 1 | 1 | 1 | 2 |
| 1718 | 43 | 2 | 154 | 26.5 | 8 | 1 | 1 | 1 | 2 | 1 | 1 | 1 | 2 | 1 | 1 | 1 | 1 | 1 |
| 1719 | 43 | 2 | 155 | 54 | 8 | 2 | 1 | 1 | 3 | 4 | 1 | 1 | 2 | 1 | 1 | 1 | 1 | 1 |
| 1720 | 43 | 2 | 155 | 54 | 8 | 1 | 1 | 1 | 4 | 1 | 1 | 1 | 1 | 1 | 1 | 1 | 1 | 1 |
| 1721 | 43 | 2 | 155 | 52 | 8 | 2 | 1 | 1 | 3 | 4 | 1 | 1 | 1 | 1 | 1 | 1 | 1 | 1 |
| 1722 | 43 | 2 | 155 | 51 | 8 | 2 | 1 | 1 | 4 | 3 | 1 | 1 | 1 | 1 | 1 | 1 | 1 | 1 |
| 1723 | 43 | 2 | 156 | 46.5 | 8 | 2 | 1 | 1 | 4 | 4 | 1 | 1 | 1 | 1 | 1 | 1 | 1 | 1 |
| 1724 | 43 | 2 | 156 | 44 | 2 | 2 | 1 | 1 | 5 | 2 | 1 | 1 | 1 | 1 | 1 | 1 | 1 | 1 |
| 1725 | 43 | 2 | 158 | 85 | 8 | 2 | 1 | 1 | 3 | 4 | 1 | 1 | 1 | 1 | 1 | 1 | 1 | 1 |
| 1726 | 43 | 2 | 158 | 72 | 2 | 2 | 1 | 1 | 5 | 3 | 1 | 1 | 1 | 1 | 1 | 1 | 1 | 1 |
| 1727 | 43 | 2 | 158 | 65 | 8 | 2 | 1 | 1 | 4 | 4 | 1 | 1 | 2 | 1 | 1 | 1 | 1 | 1 |
| 1728 | 43 | 2 | 158 | 65 | 4 | 2 | 1 | 1 | 4 | 4 | 1 | 1 | 1 | 1 | 1 | 1 | 1 | 1 |
| 1729 | 43 | 2 | 158 | 55 | 6 | 2 | 1 | 1 | 4 | 3 | 1 | 1 | 2 | 1 | 1 | 1 | 1 | 1 |
| 1730 | 43 | 2 | 158 | 48 | 8 | 2 | 1 | 1 | 4 | 3 | 1 | 1 | 2 | 1 | 1 | 1 | 1 | 1 |
| 1731 | 43 | 2 | 159 | 65 | 8 | 2 | 1 | 1 | 4 | 4 | 1 | 1 | 1 | 1 | 1 | 1 | 1 | 2 |
| 1732 | 43 | 2 | 159 | 55 | 8 | 2 | 1 | 1 | 3 | 2 | 1 | 1 | 2 | 1 | 1 | 1 | 1 | 1 |
| 1733 | 43 | 2 | 159 | 54.8 | 8 | 2 | 1 | 1 | 4 | 1 | 1 | 1 | 1 | 1 | 1 | 1 | 1 | 1 |
| 1734 | 43 | 2 | 160 | 60 | 8 | 2 | 1 | 1 | 3 | 2 | 1 | 1 | 2 | 1 | 1 | 1 | 1 | 1 |
| 1735 | 43 | 2 | 160 | 55 | 8 | 2 | 1 | 1 | 4 | 3 | 1 | 1 | 1 | 1 | 1 | 1 | 1 | 1 |
| 1736 | 43 | 2 | 160 | 55 | 3 | 2 | 1 | 1 | 5 | 4 | 1 | 1 | 2 | 1 | 1 | 1 | 1 | 1 |
| 1737 | 43 | 2 | 160 | 54 | 8 | 2 | 1 | 1 | 4 | 3 | 1 | 1 | 1 | 1 | 1 | 1 | 1 | 1 |
| 1738 | 43 | 2 | 160 | 52.5 | 3 | 2 | 1 | 1 | 3 | 3 | 1 | 1 | 1 | 1 | 1 | 1 | 1 | 1 |
| 1739 | 43 | 2 | 162 | 58 | 8 | 2 | 1 | 1 | 4 | 2 | 1 | 1 | 1 | 1 | 1 | 1 | 1 | 1 |
| 1740 | 43 | 2 | 162 | 50 | 4 | 2 | 1 | 1 | 5 | 4 | 1 | 1 | 1 | 1 | 1 | 1 | 1 | 1 |
| 1741 | 43 | 2 | 163 | 55 | 8 | 2 | 1 | 1 | 4 | 3 | 1 | 1 | 2 | 1 | 1 | 1 | 1 | 1 |
| 1742 | 43 | 2 | 163 | 52 | 8 | 2 | 1 | 1 | 5 | 3 | 2 | 1 | 1 | 2 | 1 | 1 | 1 | 2 |
| 1743 | 43 | 2 | 163 | 51 | 8 | 2 | 1 | 1 | 6 | 4 | 1 | 1 | 2 | 2 | 1 | 1 | 1 | 2 |
| 1744 | 43 | 2 | 164 | 65 | 8 | 2 | 1 | 1 | 4 | 2 | 1 | 1 | 2 | 1 | 1 | 1 | 1 | 1 |
| 1745 | 43 | 2 | 164 | 62 | 8 | 2 | 1 | 1 | 4 | 3 | 1 | 1 | 1 | 1 | 1 | 1 | 1 | 1 |
| 1746 | 43 | 2 | 164 | 59 | 8 | 2 | 1 | 2 | 5 | 1 | 1 | 1 | 1 | 1 | 1 | 1 | 1 | 1 |
| 1747 | 43 | 2 | 164 | 59 | 8 | 2 | 1 | 1 | 4 | 4 | 1 | 1 | 2 | 1 | 1 | 1 | 1 | 1 |
| 1748 | 43 | 2 | 165 | 60.1 | 2 | 1 | 1 | 1 | 5 | 3 | 1 | 1 | 2 | 1 | 1 | 1 | 1 | 1 |
| 1749 | 43 | 2 | 165 | 55 | 8 | 2 | 1 | 1 | 5 | 1 | 1 | 1 | 2 | 1 | 1 | 1 | 1 | 1 |
| 1750 | 43 | 2 | 165 | 40 | 4 | 2 | 1 | 1 | 3 | 3 | 1 | 1 | 1 | 1 | 1 | 1 | 1 | 1 |
| 1751 | 43 | 2 | 166 | 62 | 8 | 2 | 1 | 1 | 4 | 4 | 1 | 1 | 1 | 1 | 1 | 1 | 1 | 1 |
| 1752 | 43 | 2 | 168 | 85 | 4 | 2 | 1 | 1 | 4 | 2 | 1 | 1 | 1 | 1 | 1 | 1 | 1 | 1 |
| 1753 | 43 | 2 | 170 | 60 | 8 | 2 | 2 | 1 | 2 | 2 | 1 | 1 | 1 | 1 | 2 | 1 | 1 | 1 |
| 1754 | 43 | 2 | 170 | 55 | 2 | 2 | 1 | 1 | 4 | 4 | 1 | 1 | 1 | 1 | 1 | 1 | 1 | 1 |
| 1755 | 43 | 1 | 158 | 48 | 8 | 2 | 1 | 1 | 4 | 4 | 1 | 1 | 1 | 2 | 1 | 1 | 1 | 1 |
| 1756 | 43 | 1 | 165 | 66 | 3 | 2 | 1 | 1 | 3 | 4 | 1 | 1 | 1 | 1 | 2 | 1 | 1 | 2 |
| 1757 | 43 | 1 | 165 | 58 | 8 | 2 | 1 | 2 | 4 | 3 | 1 | 1 | 1 | 1 | 1 | 1 | 3 | 1 |
| 1758 | 43 | 1 | 165 | 58 | 3 | 2 | 1 | 1 | 5 | 1 | 1 | 1 | 1 | 1 | 1 | 1 | 1 | 1 |
| 1759 | 43 | 1 | 166 | 60 | 8 | 2 | 1 | 1 | 4 | 3 | 1 | 1 | 1 | 1 | 1 | 1 | 1 | 1 |
| 1760 | 43 | 1 | 167 | 52 | 8 | 2 | 1 | 1 | 5 | 4 | 2 | 1 | 2 | 1 | 1 | 1 | 3 | 1 |
| 1761 | 43 | 1 | 168 | 61.5 | 4 | 2 | 1 | 1 | 3 | 4 | 1 | 1 | 1 | 1 | 1 | 1 | 2 | 1 |
| 1762 | 43 | 1 | 170 | 70 | 8 | 2 | 1 | 2 | 5 | 4 | 1 | 1 | 2 | 1 | 1 | 1 | 3 | 1 |
| 1763 | 43 | 1 | 170 | 60.5 | 2 | 2 | 1 | 2 | 5 | 2 | 1 | 1 | 2 | 1 | 1 | 1 | 1 | 1 |
| 1764 | 43 | 1 | 173 | 65 | 8 | 2 | 1 | 1 | 5 | 4 | 1 | 1 | 1 | 1 | 1 | 1 | 1 | 1 |
| 1765 | 43 | 1 | 176 | 87 | 4 | 2 | 1 | 1 | 5 | 3 | 1 | 1 | 2 | 1 | 1 | 1 | 1 | 1 |
| 1766 | 44 | 2 | 55 | 74 | 8 | 2 | 1 | 1 | 5 | 4 | 1 | 1 | 1 | 2 | 1 | 1 | 1 | 2 |
| 1767 | 44 | 2 | 145 | 48 | 3 | 2 | 1 | 1 | 5 | 2 | 1 | 1 | 1 | 1 | 1 | 1 | 1 | 1 |
| 1768 | 44 | 2 | 148 | 60.2 | 8 | 2 | 1 | 1 | 4 | 2 | 1 | 1 | 1 | 1 | 1 | 1 | 1 | 1 |
| 1769 | 44 | 2 | 148 | 56 | 8 | 3 | 1 | 1 | 4 | 4 | 1 | 1 | 2 | 1 | 1 | 1 | 1 | 1 |
| 1770 | 44 | 2 | 150 | 60 | 8 | 2 | 2 | 2 | 4 | 1 | 1 | 1 | 1 | 1 | 1 | 1 | 1 | 1 |
| 1771 | 44 | 2 | 150 | 50 | 3 | 2 | 2 | 1 | 4 | 2 | 1 | 1 | 1 | 1 | 1 | 1 | 1 | 1 |
| 1772 | 44 | 2 | 150 | 45 | 3 | 2 | 1 | 1 | 4 | 4 | 1 | 1 | 1 | 1 | 1 | 1 | 1 | 1 |
| 1773 | 44 | 2 | 151 | 50 | 8 | 2 | 1 | 1 | 4 | 4 | 1 | 1 | 1 | 1 | 1 | 1 | 1 | 1 |
| 1774 | 44 | 2 | 153 | 60 | 4 | 2 | 2 | 1 | 5 | 4 | 1 | 1 | 2 | 1 | 1 | 1 | 1 | 1 |
| 1775 | 44 | 2 | 154 | 65 | 8 | 2 | 1 | 2 | 3 | 2 | 1 | 1 | 1 | 1 | 1 | 1 | 1 | 1 |
| 1776 | 44 | 2 | 154 | 55 | 4 | 2 | 1 | 1 | 3 | 1 | 1 | 1 | 1 | 1 | 1 | 1 | 1 | 1 |
| 1777 | 44 | 2 | 154 | 45 | 4 | 2 | 1 | 1 | 5 | 3 | 1 | 1 | 1 | 1 | 1 | 1 | 1 | 1 |
| 1778 | 44 | 2 | 155 | 56 | 8 | 2 | 1 | 1 | 3 | 3 | 1 | 1 | 2 | 1 | 2 | 1 | 1 | 1 |
| 1779 | 44 | 2 | 155 | 50.5 | 8 | 2 | 1 | 1 | 4 | 2 | 1 | 1 | 1 | 1 | 1 | 1 | 1 | 1 |
| 1780 | 44 | 2 | 155 | 50 | 6 | 2 | 1 | 1 | 4 | 2 | 1 | 1 | 1 | 1 | 1 | 1 | 1 | 1 |
| 1781 | 44 | 2 | 156 | 64 | 8 | 1 | 1 | 1 | 2 | 2 | 1 | 1 | 1 | 1 | 1 | 1 | 1 | 1 |
| 1782 | 44 | 2 | 156 | 61 | 4 | 2 | 1 | 1 | 3 | 4 | 1 | 1 | 2 | 1 | 1 | 1 | 1 | 1 |
| 1783 | 44 | 2 | 157 | 69 | 8 | 2 | 1 | 1 | 3 | 3 | 1 | 1 | 1 | 1 | 1 | 1 | 1 | 1 |
| 1784 | 44 | 2 | 157 | 50 | 8 | 4 | 1 | 1 | 4 | 2 | 1 | 1 | 1 | 1 | 1 | 1 | 1 | 1 |
| 1785 | 44 | 2 | 157.5 | 57.6 | 2 | 2 | 1 | 1 | 5 | 2 | 1 | 1 | 1 | 1 | 1 | 1 | 1 | 1 |
| 1786 | 44 | 2 | 158 | 69 | 8 | 2 | 1 | 1 | 3 | 1 | 1 | 1 | 2 | 1 | 1 | 1 | 1 | 1 |
| 1787 | 44 | 2 | 158 | 60 | 4 | 2 | 2 | 1 | 3 | 3 | 1 | 1 | 2 | 1 | 1 | 1 | 1 | 1 |
| 1788 | 44 | 2 | 158 | 59 | 4 | 2 | 1 | 3 | 5 | 3 | 1 | 1 | 2 | 1 | 1 | 1 | 1 | 1 |
| 1789 | 44 | 2 | 158 | 56 | 8 | 2 | 1 | 1 | 5 | 4 | 1 | 1 | 2 | 1 | 1 | 1 | 1 | 1 |
| 1790 | 44 | 2 | 158 | 56 | 4 | 2 | 1 | 1 | 4 | 3 | 1 | 1 | 1 | 1 | 1 | 1 | 1 | 1 |
| 1791 | 44 | 2 | 158 | 55.5 | 8 | 2 | 1 | 1 | 4 | 3 | 1 | 1 | 2 | 1 | 1 | 1 | 1 | 1 |
| 1792 | 44 | 2 | 158 | 54 | 8 | 2 | 1 | 1 | 4 | 4 | 1 | 1 | 1 | 1 | 1 | 1 | 1 | 1 |
| 1793 | 44 | 2 | 158 | 53 | 3 | 2 | 2 | 1 | 5 | 2 | 1 | 1 | 1 | 1 | 1 | 1 | 1 | 1 |
| 1794 | 44 | 2 | 159 | 55 | 8 | 2 | 1 | 1 | 3 | 4 | 1 | 1 | 1 | 1 | 1 | 1 | 1 | 1 |
| 1795 | 44 | 2 | 160 | 70 | 8 | 3 | 1 | 1 | 4 | 4 | 1 | 1 | 2 | 1 | 1 | 1 | 1 | 1 |
| 1796 | 44 | 2 | 160 | 70 | 8 | 2 | 1 | 1 | 2 | 4 | 1 | 1 | 1 | 1 | 1 | 1 | 1 | 2 |
| 1797 | 44 | 2 | 160 | 68 | 4 | 2 | 1 | 1 | 4 | 2 | 1 | 1 | 1 | 1 | 1 | 1 | 1 | 1 |
| 1798 | 44 | 2 | 160 | 61.5 | 8 | 2 | 1 | 1 | 5 | 3 | 1 | 1 | 2 | 1 | 1 | 1 | 1 | 1 |
| 1799 | 44 | 2 | 160 | 56.5 | 2 | 2 | 2 | 1 | 4 | 3 | 1 | 1 | 1 | 1 | 1 | 1 | 1 | 1 |
| 1800 | 44 | 2 | 160 | 55 | 8 | 2 | 1 | 1 | 3 | 3 | 1 | 1 | 1 | 1 | 2 | 1 | 1 | 1 |
| 1801 | 44 | 2 | 160 | 55 | 4 | 2 | 1 | 1 | 4 | 4 | 1 | 1 | 1 | 1 | 1 | 1 | 1 | 1 |
| 1802 | 44 | 2 | 160 | 54.2 | 8 | 2 | 1 | 1 | 4 | 4 | 1 | 1 | 1 | 2 | 1 | 1 | 1 | 2 |
| 1803 | 44 | 2 | 160 | 50 | 8 | 2 | 1 | 1 | 4 | 4 | 1 | 1 | 1 | 1 | 1 | 1 | 1 | 1 |
| 1804 | 44 | 2 | 160 | 46 | 3 | 2 | 1 | 1 | 4 | 3 | 1 | 1 | 2 | 1 | 2 | 1 | 1 | 1 |
| 1805 | 44 | 2 | 161 | 52 | 2 | 4 | 1 | 1 | 5 | 3 | 1 | 1 | 2 | 1 | 1 | 1 | 1 | 1 |
| 1806 | 44 | 2 | 162 | 62 | 8 | 2 | 1 | 1 | 4 | 4 | 1 | 1 | 1 | 1 | 1 | 1 | 1 | 1 |
| 1807 | 44 | 2 | 162 | 62 | 2 | 2 | 1 | 1 | 4 | 1 | 1 | 1 | 2 | 1 | 2 | 1 | 1 | 1 |
| 1808 | 44 | 2 | 162 | 60 | 2 | 2 | 1 | 2 | 4 | 1 | 1 | 1 | 2 | 1 | 2 | 1 | 1 | 1 |
| 1809 | 44 | 2 | 162 | 56 | 8 | 2 | 1 | 1 | 5 | 4 | 1 | 1 | 2 | 1 | 1 | 1 | 1 | 1 |
| 1810 | 44 | 2 | 163 | 65 | 8 | 2 | 2 | 1 | 3 | 3 | 1 | 1 | 2 | 1 | 1 | 1 | 1 | 1 |
| 1811 | 44 | 2 | 164 | 55 | 6 | 2 | 1 | 1 | 4 | 3 | 1 | 1 | 2 | 2 | 1 | 1 | 1 | 2 |
| 1812 | 44 | 2 | 165 | 65 | 3 | 2 | 1 | 1 | 5 | 3 | 1 | 1 | 1 | 1 | 1 | 1 | 1 | 1 |
| 1813 | 44 | 2 | 165 | 60 | 8 | 3 | 1 | 1 | 2 | 3 | 1 | 1 | 1 | 1 | 2 | 1 | 1 | 1 |
| 1814 | 44 | 2 | 165 | 55 | 4 | 2 | 1 | 1 | 5 | 2 | 1 | 1 | 2 | 1 | 1 | 1 | 1 | 1 |
| 1815 | 44 | 1 | 161.5 | 60.7 | 4 | 2 | 1 | 1 | 5 | 2 | 1 | 1 | 1 | 1 | 1 | 1 | 1 | 1 |
| 1816 | 44 | 1 | 164 | 49 | 1 | 4 | 1 | 2 | 5 | 1 | 1 | 1 | 2 | 1 | 2 | 1 | 1 | 1 |
| 1817 | 44 | 1 | 165 | 69 | 8 | 2 | 1 | 2 | 4 | 3 | 1 | 1 | 1 | 1 | 1 | 1 | 3 | 1 |
| 1818 | 44 | 1 | 168 | 63.5 | 1 | 2 | 1 | 1 | 5 | 1 | 1 | 1 | 1 | 1 | 1 | 1 | 3 | 1 |
| 1819 | 44 | 1 | 170 | 100 | 4 | 2 | 1 | 1 | 5 | 1 | 1 | 1 | 1 | 1 | 1 | 1 | 1 | 1 |
| 1820 | 44 | 1 | 170 | 60 | 2 | 2 | 1 | 1 | 5 | 3 | 1 | 1 | 1 | 1 | 1 | 1 | 1 | 1 |
| 1821 | 44 | 1 | 172 | 80 | 8 | 2 | 1 | 1 | 2 | 1 | 1 | 1 | 1 | 2 | 1 | 1 | 3 | 2 |
| 1822 | 44 | 1 | 172 | 80 | 3 | 2 | 1 | 1 | 5 | 3 | 1 | 1 | 2 | 1 | 1 | 1 | 1 | 1 |
| 1823 | 44 | 1 | 172 | 55 | 2 | 2 | 1 | 1 | 4 | 1 | 1 | 1 | 2 | 1 | 1 | 1 | 1 | 1 |
| 1824 | 44 | 1 | 173 | 75 | 8 | 2 | 1 | 1 | 5 | 4 | 1 | 1 | 1 | 1 | 1 | 1 | 1 | 1 |
| 1825 | 44 | 1 | 173 | 75 | 2 | 2 | 1 | 1 | 3 | 4 | 1 | 1 | 1 | 1 | 1 | 1 | 3 | 1 |
| 1826 | 44 | 1 | 175 | 82 | 8 | 2 | 1 | 1 | 4 | 2 | 1 | 1 | 2 | 1 | 2 | 1 | 1 | 1 |
| 1827 | 44 | 1 | 179 | 66 | 1 | 2 | 1 | 1 | 5 | 4 | 1 | 1 | 1 | 1 | 1 | 1 | 1 | 1 |
| 1828 | 45 | 2 | 140 | 45 | 8 | 2 | 1 | 1 | 4 | 4 | 1 | 1 | 1 | 1 | 1 | 1 | 1 | 1 |
| 1829 | 45 | 2 | 141 | 53.8 | 8 | 4 | 2 | 1 | 3 | 1 | 1 | 1 | 1 | 1 | 1 | 1 | 1 | 1 |
| 1830 | 45 | 2 | 146 | 54.5 | 8 | 2 | 1 | 1 | 4 | 2 | 1 | 1 | 1 | 1 | 1 | 1 | 1 | 1 |
| 1831 | 45 | 2 | 151.5 | 49.4 | 8 | 2 | 1 | 1 | 3 | 3 | 1 | 1 | 2 | 1 | 2 | 1 | 1 | 1 |
| 1832 | 45 | 2 | 153 | 60 | 1 | 2 | 1 | 1 | 5 | 3 | 1 | 1 | 1 | 1 | 2 | 1 | 1 | 1 |
| 1833 | 45 | 2 | 153 | 46 | 8 | 2 | 1 | 1 | 3 | 2 | 1 | 1 | 2 | 1 | 1 | 1 | 1 | 1 |
| 1834 | 45 | 2 | 154 | 50 | 6 | 2 | 1 | 1 | 3 | 3 | 1 | 1 | 1 | 1 | 1 | 1 | 1 | 1 |
| 1835 | 45 | 2 | 154 | 44 | 8 | 1 | 1 | 1 | 4 | 3 | 1 | 1 | 1 | 1 | 1 | 1 | 1 | 1 |
| 1836 | 45 | 2 | 155 | 60 | 8 | 2 | 1 | 1 | 4 | 3 | 1 | 1 | 2 | 1 | 1 | 1 | 1 | 1 |
| 1837 | 45 | 2 | 155 | 60 | 8 | 2 | 1 | 1 | 5 | 3 | 1 | 1 | 1 | 1 | 1 | 1 | 1 | 1 |
| 1838 | 45 | 2 | 155 | 57 | 8 | 2 | 1 | 1 | 3 | 4 | 1 | 1 | 1 | 1 | 1 | 1 | 2 | 1 |
| 1839 | 45 | 2 | 155 | 55 | 8 | 2 | 1 | 1 | 3 | 4 | 1 | 1 | 1 | 1 | 1 | 1 | 1 | 1 |
| 1840 | 45 | 2 | 155 | 53 | 4 | 2 | 1 | 1 | 3 | 4 | 1 | 1 | 2 | 1 | 1 | 1 | 1 | 1 |
| 1841 | 45 | 2 | 155 | 51 | 8 | 2 | 1 | 1 | 4 | 4 | 1 | 1 | 1 | 1 | 1 | 1 | 1 | 1 |
| 1842 | 45 | 2 | 155 | 45 | 4 | 2 | 1 | 1 | 3 | 4 | 1 | 1 | 1 | 1 | 1 | 1 | 1 | 1 |
| 1843 | 45 | 2 | 156 | 68 | 4 | 2 | 1 | 1 | 4 | 4 | 1 | 1 | 1 | 1 | 1 | 1 | 1 | 1 |
| 1844 | 45 | 2 | 156 | 54 | 3 | 2 | 1 | 1 | 4 | 4 | 1 | 1 | 2 | 1 | 1 | 1 | 1 | 1 |
| 1845 | 45 | 2 | 156 | 52.5 | 8 | 2 | 1 | 1 | 4 | 4 | 1 | 1 | 1 | 1 | 1 | 1 | 1 | 1 |
| 1846 | 45 | 2 | 156 | 44 | 8 | 2 | 1 | 1 | 3 | 3 | 1 | 1 | 1 | 1 | 1 | 1 | 1 | 1 |
| 1847 | 45 | 2 | 157 | 61 | 8 | 2 | 1 | 1 | 6 | 4 | 1 | 1 | 1 | 1 | 1 | 1 | 1 | 1 |
| 1848 | 45 | 2 | 157 | 60 | 8 | 2 | 1 | 1 | 3 | 4 | 1 | 1 | 1 | 1 | 1 | 1 | 1 | 1 |
| 1849 | 45 | 2 | 158 | 60 | 8 | 2 | 1 | 1 | 3 | 3 | 1 | 1 | 1 | 1 | 2 | 1 | 1 | 1 |
| 1850 | 45 | 2 | 158 | 55 | 8 | 2 | 1 | 1 | 3 | 3 | 1 | 1 | 2 | 1 | 1 | 1 | 1 | 1 |
| 1851 | 45 | 2 | 158 | 52 | 8 | 2 | 1 | 1 | 3 | 4 | 1 | 1 | 1 | 1 | 1 | 1 | 2 | 1 |
| 1852 | 45 | 2 | 158.5 | 61.6 | 8 | 2 | 1 | 2 | 3 | 3 | 1 | 1 | 1 | 1 | 1 | 1 | 1 | 1 |
| 1853 | 45 | 2 | 159 | 64 | 8 | 1 | 1 | 1 | 3 | 3 | 1 | 1 | 1 | 1 | 1 | 1 | 1 | 1 |
| 1854 | 45 | 2 | 159 | 52 | 8 | 2 | 1 | 1 | 4 | 1 | 1 | 1 | 1 | 1 | 1 | 1 | 1 | 1 |
| 1855 | 45 | 2 | 159.5 | 57 | 8 | 2 | 1 | 1 | 4 | 4 | 1 | 1 | 1 | 1 | 2 | 1 | 1 | 1 |
| 1856 | 45 | 2 | 160 | 71 | 8 | 2 | 1 | 1 | 3 | 1 | 2 | 1 | 1 | 2 | 2 | 1 | 1 | 2 |
| 1857 | 45 | 2 | 160 | 60 | 6 | 2 | 1 | 1 | 4 | 3 | 1 | 1 | 2 | 1 | 1 | 1 | 1 | 1 |
| 1858 | 45 | 2 | 160 | 54 | 1 | 2 | 1 | 1 | 4 | 3 | 1 | 1 | 1 | 1 | 1 | 1 | 1 | 1 |
| 1859 | 45 | 2 | 160 | 52 | 4 | 2 | 1 | 1 | 5 | 4 | 1 | 1 | 1 | 1 | 1 | 1 | 1 | 1 |
| 1860 | 45 | 2 | 160 | 51 | 1 | 2 | 1 | 1 | 4 | 3 | 1 | 1 | 1 | 1 | 1 | 1 | 1 | 1 |
| 1861 | 45 | 2 | 160 | 50 | 4 | 2 | 1 | 2 | 3 | 2 | 1 | 1 | 1 | 1 | 1 | 1 | 1 | 1 |
| 1862 | 45 | 2 | 160 | 40 | 4 | 2 | 1 | 1 | 4 | 4 | 1 | 1 | 2 | 2 | 1 | 1 | 1 | 2 |
| 1863 | 45 | 2 | 161 | 100 | 8 | 2 | 1 | 1 | 5 | 4 | 1 | 1 | 1 | 1 | 1 | 1 | 1 | 1 |
| 1864 | 45 | 2 | 162 | 65 | 4 | 2 | 1 | 1 | 4 | 3 | 1 | 1 | 2 | 1 | 1 | 1 | 1 | 1 |
| 1865 | 45 | 2 | 162 | 65 | 3 | 2 | 1 | 1 | 4 | 4 | 1 | 1 | 1 | 1 | 1 | 1 | 1 | 1 |
| 1866 | 45 | 2 | 162 | 60 | 8 | 2 | 1 | 1 | 4 | 2 | 1 | 1 | 1 | 1 | 1 | 1 | 1 | 1 |
| 1867 | 45 | 2 | 163 | 77 | 8 | 2 | 1 | 1 | 3 | 1 | 1 | 1 | 1 | 1 | 1 | 1 | 1 | 1 |
| 1868 | 45 | 2 | 163 | 63 | 3 | 2 | 1 | 1 | 5 | 3 | 1 | 1 | 2 | 1 | 2 | 1 | 1 | 1 |
| 1869 | 45 | 2 | 163 | 58 | 8 | 2 | 1 | 1 | 6 | 4 | 1 | 1 | 2 | 1 | 1 | 1 | 1 | 1 |
| 1870 | 45 | 2 | 163 | 56 | 4 | 2 | 1 | 1 | 5 | 2 | 1 | 1 | 1 | 1 | 1 | 1 | 1 | 1 |
| 1871 | 45 | 2 | 164 | 53 | 3 | 2 | 1 | 1 | 5 | 4 | 1 | 1 | 1 | 1 | 1 | 1 | 1 | 1 |
| 1872 | 45 | 2 | 165 | 73 | 3 | 2 | 1 | 1 | 4 | 1 | 1 | 1 | 1 | 1 | 1 | 1 | 1 | 1 |
| 1873 | 45 | 2 | 165 | 65 | 3 | 2 | 1 | 1 | 5 | 1 | 1 | 1 | 2 | 1 | 1 | 1 | 1 | 1 |
| 1874 | 45 | 2 | 165 | 56 | 2 | 2 | 1 | 1 | 5 | 2 | 1 | 1 | 1 | 1 | 1 | 1 | 1 | 1 |
| 1875 | 45 | 2 | 167.5 | 63.5 | 3 | 2 | 1 | 1 | 3 | 1 | 1 | 1 | 1 | 1 | 1 | 1 | 1 | 1 |
| 1876 | 45 | 2 | 168 | 70 | 8 | 1 | 1 | 1 | 5 | 4 | 1 | 1 | 1 | 1 | 1 | 1 | 1 | 1 |
| 1877 | 45 | 2 | 168 | 51 | 4 | 2 | 2 | 1 | 5 | 1 | 1 | 1 | 1 | 1 | 1 | 1 | 1 | 1 |
| 1878 | 45 | 1 | 160 | 60 | 2 | 2 | 1 | 1 | 5 | 3 | 1 | 1 | 1 | 1 | 1 | 1 | 1 | 1 |
| 1879 | 45 | 1 | 161 | 37.5 | 8 | 2 | 1 | 2 | 4 | 3 | 1 | 1 | 1 | 1 | 2 | 2 | 1 | 1 |
| 1880 | 45 | 1 | 162 | 84 | 8 | 2 | 1 | 1 | 6 | 3 | 1 | 1 | 1 | 1 | 1 | 1 | 1 | 1 |
| 1881 | 45 | 1 | 168 | 72 | 8 | 1 | 1 | 1 | 3 | 2 | 1 | 1 | 1 | 1 | 1 | 1 | 1 | 1 |
| 1882 | 45 | 1 | 168 | 72 | 4 | 2 | 2 | 1 | 5 | 1 | 1 | 1 | 2 | 1 | 1 | 1 | 1 | 1 |
| 1883 | 45 | 1 | 170 | 68 | 1 | 2 | 1 | 2 | 5 | 2 | 1 | 1 | 2 | 1 | 1 | 1 | 1 | 1 |
| 1884 | 45 | 1 | 170 | 58 | 8 | 2 | 1 | 1 | 4 | 2 | 1 | 1 | 1 | 1 | 1 | 1 | 1 | 1 |
| 1885 | 45 | 1 | 170 | 55 | 8 | 2 | 1 | 1 | 4 | 4 | 1 | 1 | 1 | 1 | 1 | 1 | 1 | 2 |
| 1886 | 45 | 1 | 173 | 66 | 8 | 2 | 1 | 1 | 5 | 1 | 1 | 1 | 2 | 1 | 1 | 1 | 3 | 1 |
| 1887 | 45 | 1 | 175 | 73 | 3 | 2 | 1 | 1 | 5 | 4 | 1 | 1 | 1 | 1 | 1 | 1 | 1 | 1 |
| 1888 | 45 | 1 | 179 | 63 | 4 | 2 | 1 | 1 | 4 | 1 | 1 | 1 | 1 | 1 | 1 | 1 | 1 | 1 |
| 1889 | 46 | 2 | 138 | 49 | 3 | 2 | 1 | 1 | 3 | 2 | 1 | 1 | 1 | 1 | 1 | 1 | 1 | 1 |
| 1890 | 46 | 2 | 150 | 57 | 8 | 2 | 1 | 1 | 3 | 4 | 1 | 1 | 1 | 1 | 1 | 1 | 1 | 1 |
| 1891 | 46 | 2 | 150 | 55 | 8 | 4 | 1 | 1 | 4 | 3 | 1 | 1 | 1 | 1 | 1 | 1 | 1 | 1 |
| 1892 | 46 | 2 | 150 | 46 | 8 | 2 | 1 | 1 | 5 | 2 | 1 | 1 | 1 | 1 | 1 | 1 | 1 | 1 |
| 1893 | 46 | 2 | 152 | 54 | 8 | 2 | 1 | 1 | 4 | 3 | 1 | 1 | 1 | 1 | 1 | 2 | 1 | 1 |
| 1894 | 46 | 2 | 153 | 48 | 4 | 2 | 1 | 1 | 4 | 3 | 1 | 1 | 1 | 2 | 1 | 1 | 1 | 2 |
| 1895 | 46 | 2 | 155 | 44 | 8 | 2 | 1 | 1 | 4 | 2 | 1 | 1 | 1 | 1 | 1 | 1 | 1 | 1 |
| 1896 | 46 | 2 | 158 | 65 | 8 | 2 | 2 | 1 | 4 | 4 | 1 | 1 | 1 | 1 | 1 | 1 | 1 | 2 |
| 1897 | 46 | 2 | 158 | 65 | 8 | 2 | 1 | 1 | 3 | 3 | 1 | 1 | 1 | 1 | 1 | 1 | 1 | 1 |
| 1898 | 46 | 2 | 158 | 65 | 3 | 2 | 1 | 1 | 4 | 4 | 1 | 1 | 1 | 1 | 1 | 1 | 1 | 1 |
| 1899 | 46 | 2 | 158 | 64 | 8 | 2 | 1 | 1 | 3 | 1 | 1 | 1 | 1 | 1 | 1 | 1 | 1 | 1 |
| 1900 | 46 | 2 | 158 | 60 | 8 | 2 | 1 | 1 | 3 | 3 | 1 | 1 | 1 | 1 | 1 | 1 | 1 | 1 |
| 1901 | 46 | 2 | 158 | 56 | 4 | 2 | 1 | 2 | 3 | 2 | 1 | 1 | 1 | 1 | 1 | 1 | 1 | 1 |
| 1902 | 46 | 2 | 158 | 55 | 8 | 2 | 1 | 1 | 5 | 3 | 1 | 1 | 1 | 1 | 1 | 1 | 1 | 1 |
| 1903 | 46 | 2 | 158 | 55 | 8 | 2 | 1 | 1 | 3 | 4 | 1 | 1 | 1 | 1 | 1 | 1 | 1 | 1 |
| 1904 | 46 | 2 | 158 | 54 | 8 | 2 | 1 | 1 | 3 | 3 | 1 | 1 | 1 | 1 | 1 | 1 | 1 | 2 |
| 1905 | 46 | 2 | 158 | 53 | 8 | 2 | 1 | 1 | 4 | 3 | 1 | 1 | 1 | 1 | 1 | 1 | 1 | 1 |
| 1906 | 46 | 2 | 158 | 49 | 8 | 2 | 1 | 1 | 3 | 4 | 1 | 1 | 1 | 1 | 1 | 1 | 1 | 1 |
| 1907 | 46 | 2 | 158 | 48 | 8 | 2 | 1 | 1 | 4 | 2 | 1 | 1 | 1 | 1 | 1 | 1 | 1 | 1 |
| 1908 | 46 | 2 | 158 | 46 | 8 | 2 | 1 | 1 | 4 | 2 | 1 | 1 | 1 | 2 | 1 | 1 | 1 | 2 |
| 1909 | 46 | 2 | 159 | 59 | 8 | 2 | 1 | 1 | 4 | 3 | 3 | 1 | 1 | 1 | 2 | 1 | 1 | 1 |
| 1910 | 46 | 2 | 159 | 57 | 8 | 2 | 1 | 1 | 4 | 4 | 1 | 1 | 2 | 1 | 1 | 1 | 1 | 1 |
| 1911 | 46 | 2 | 159 | 54 | 6 | 1 | 1 | 1 | 3 | 3 | 1 | 1 | 1 | 1 | 1 | 1 | 1 | 1 |
| 1912 | 46 | 2 | 159 | 50 | 8 | 2 | 1 | 1 | 3 | 2 | 1 | 1 | 1 | 1 | 2 | 1 | 1 | 1 |
| 1913 | 46 | 2 | 160 | 69 | 8 | 2 | 1 | 1 | 3 | 2 | 1 | 1 | 1 | 2 | 2 | 2 | 1 | 2 |
| 1914 | 46 | 2 | 160 | 62 | 3 | 2 | 1 | 1 | 5 | 4 | 1 | 1 | 2 | 1 | 1 | 1 | 1 | 1 |
| 1915 | 46 | 2 | 160 | 60 | 2 | 3 | 1 | 1 | 4 | 4 | 1 | 1 | 2 | 1 | 1 | 1 | 1 | 1 |
| 1916 | 46 | 2 | 160 | 53 | 4 | 2 | 1 | 1 | 5 | 3 | 1 | 1 | 2 | 1 | 1 | 1 | 1 | 1 |
| 1917 | 46 | 2 | 160 | 52.5 | 4 | 2 | 1 | 1 | 3 | 4 | 1 | 1 | 1 | 1 | 2 | 1 | 1 | 1 |
| 1918 | 46 | 2 | 160 | 52.5 | 3 | 2 | 1 | 1 | 5 | 2 | 1 | 1 | 1 | 1 | 1 | 1 | 1 | 1 |
| 1919 | 46 | 2 | 160 | 51 | 8 | 2 | 1 | 1 | 4 | 1 | 1 | 1 | 1 | 1 | 1 | 1 | 1 | 1 |
| 1920 | 46 | 2 | 161 | 78 | 2 | 1 | 1 | 1 | 5 | 3 | 2 | 1 | 1 | 2 | 2 | 1 | 1 | 2 |
| 1921 | 46 | 2 | 162 | 58 | 8 | 2 | 1 | 1 | 3 | 3 | 1 | 1 | 1 | 1 | 1 | 1 | 1 | 1 |
| 1922 | 46 | 2 | 162.5 | 61.2 | 8 | 2 | 1 | 1 | 3 | 4 | 1 | 1 | 2 | 1 | 1 | 1 | 1 | 1 |
| 1923 | 46 | 2 | 163 | 67 | 5 | 2 | 1 | 1 | 3 | 4 | 1 | 1 | 2 | 1 | 2 | 1 | 1 | 1 |
| 1924 | 46 | 2 | 164 | 51 | 8 | 2 | 1 | 1 | 4 | 3 | 1 | 1 | 1 | 1 | 1 | 1 | 1 | 1 |
| 1925 | 46 | 1 | 162 | 60 | 8 | 2 | 1 | 1 | 3 | 2 | 1 | 1 | 1 | 1 | 1 | 1 | 1 | 1 |
| 1926 | 46 | 1 | 164 | 59 | 4 | 2 | 1 | 1 | 5 | 2 | 1 | 1 | 1 | 1 | 1 | 1 | 1 | 1 |
| 1927 | 46 | 1 | 168 | 70 | 8 | 2 | 1 | 1 | 4 | 2 | 1 | 1 | 1 | 1 | 1 | 1 | 1 | 1 |
| 1928 | 46 | 1 | 168 | 56 | 8 | 1 | 1 | 1 | 2 | 3 | 1 | 1 | 1 | 1 | 2 | 1 | 3 | 1 |
| 1929 | 46 | 1 | 170 | 85 | 2 | 2 | 1 | 1 | 4 | 1 | 1 | 1 | 1 | 1 | 1 | 1 | 1 | 1 |
| 1930 | 46 | 1 | 171 | 93.2 | 8 | 2 | 1 | 2 | 4 | 4 | 1 | 1 | 1 | 1 | 1 | 1 | 3 | 1 |
| 1931 | 46 | 1 | 172 | 67.6 | 8 | 2 | 1 | 1 | 3 | 4 | 1 | 1 | 2 | 1 | 1 | 1 | 3 | 1 |
| 1932 | 46 | 1 | 172 | 58 | 8 | 2 | 1 | 1 | 4 | 4 | 1 | 1 | 1 | 1 | 1 | 1 | 1 | 1 |
| 1933 | 46 | 1 | 175 | 80 | 2 | 2 | 1 | 1 | 5 | 2 | 1 | 1 | 2 | 1 | 1 | 1 | 1 | 1 |
| 1934 | 46 | 1 | 175 | 65 | 3 | 2 | 1 | 1 | 5 | 2 | 1 | 1 | 1 | 1 | 1 | 1 | 1 | 1 |
| 1935 | 46 | 1 | 176 | 75 | 8 | 2 | 1 | 1 | 6 | 4 | 1 | 1 | 1 | 1 | 1 | 1 | 1 | 1 |
| 1936 | 46 | 1 | 183 | 69 | 8 | 2 | 1 | 1 | 6 | 4 | 1 | 1 | 1 | 1 | 1 | 1 | 1 | 1 |
| 1937 | 46 | 1 | 185 | 97 | 8 | 2 | 1 | 1 | 6 | 2 | 1 | 1 | 1 | 1 | 1 | 1 | 1 | 1 |
| 1938 | 47 | 2 | 150.2 | 55.1 | 8 | 2 | 1 | 1 | 3 | 3 | 1 | 1 | 1 | 1 | 1 | 1 | 1 | 1 |
| 1939 | 47 | 2 | 152 | 55 | 4 | 2 | 1 | 1 | 2 | 4 | 1 | 1 | 1 | 1 | 2 | 1 | 1 | 1 |
| 1940 | 47 | 2 | 152 | 52 | 2 | 2 | 1 | 1 | 4 | 1 | 1 | 1 | 1 | 1 | 1 | 1 | 1 | 1 |
| 1941 | 47 | 2 | 153 | 50 | 2 | 2 | 1 | 2 | 5 | 2 | 1 | 1 | 1 | 1 | 1 | 1 | 1 | 1 |
| 1942 | 47 | 2 | 153 | 44.5 | 8 | 2 | 1 | 1 | 3 | 3 | 1 | 1 | 2 | 1 | 1 | 1 | 1 | 1 |
| 1943 | 47 | 2 | 154 | 57 | 8 | 2 | 1 | 1 | 4 | 3 | 1 | 1 | 1 | 2 | 1 | 1 | 1 | 1 |
| 1944 | 47 | 2 | 154.5 | 61 | 3 | 2 | 1 | 1 | 4 | 2 | 2 | 1 | 2 | 1 | 2 | 1 | 1 | 1 |
| 1945 | 47 | 2 | 155 | 62 | 8 | 2 | 1 | 1 | 4 | 4 | 1 | 1 | 1 | 1 | 1 | 1 | 1 | 1 |
| 1946 | 47 | 2 | 155 | 58 | 4 | 4 | 1 | 1 | 3 | 1 | 1 | 1 | 1 | 1 | 1 | 1 | 1 | 1 |
| 1947 | 47 | 2 | 155 | 55 | 8 | 2 | 1 | 1 | 4 | 4 | 1 | 1 | 1 | 1 | 1 | 1 | 1 | 1 |
| 1948 | 47 | 2 | 155 | 52 | 8 | 2 | 1 | 1 | 3 | 3 | 1 | 1 | 1 | 1 | 1 | 1 | 1 | 2 |
| 1949 | 47 | 2 | 155 | 50 | 8 | 2 | 1 | 1 | 6 | 4 | 1 | 1 | 1 | 1 | 1 | 1 | 1 | 1 |
| 1950 | 47 | 2 | 155 | 45 | 8 | 2 | 1 | 1 | 4 | 3 | 2 | 1 | 2 | 1 | 1 | 1 | 1 | 1 |
| 1951 | 47 | 2 | 156 | 60 | 8 | 1 | 1 | 1 | 3 | 4 | 1 | 1 | 1 | 1 | 1 | 1 | 1 | 1 |
| 1952 | 47 | 2 | 156 | 60 | 8 | 1 | 1 | 1 | 3 | 3 | 1 | 1 | 1 | 1 | 1 | 1 | 1 | 1 |
| 1953 | 47 | 2 | 156 | 56 | 8 | 2 | 1 | 1 | 4 | 4 | 1 | 1 | 1 | 1 | 1 | 1 | 1 | 1 |
| 1954 | 47 | 2 | 156 | 55 | 8 | 2 | 1 | 1 | 4 | 3 | 1 | 1 | 2 | 1 | 1 | 1 | 1 | 1 |
| 1955 | 47 | 2 | 156 | 50 | 8 | 2 | 1 | 1 | 4 | 4 | 1 | 1 | 1 | 1 | 1 | 1 | 1 | 2 |
| 1956 | 47 | 2 | 156 | 49 | 8 | 1 | 1 | 1 | 3 | 4 | 1 | 1 | 1 | 1 | 1 | 1 | 1 | 1 |
| 1957 | 47 | 2 | 157 | 63 | 4 | 2 | 1 | 1 | 3 | 4 | 1 | 1 | 1 | 1 | 1 | 1 | 1 | 1 |
| 1958 | 47 | 2 | 157 | 54.5 | 3 | 2 | 1 | 1 | 3 | 4 | 1 | 1 | 1 | 1 | 1 | 1 | 3 | 1 |
| 1959 | 47 | 2 | 158 | 56 | 4 | 2 | 1 | 1 | 4 | 4 | 1 | 1 | 2 | 1 | 1 | 1 | 1 | 1 |
| 1960 | 47 | 2 | 158 | 55 | 1 | 1 | 1 | 1 | 1 | 3 | 1 | 1 | 1 | 1 | 1 | 1 | 1 | 1 |
| 1961 | 47 | 2 | 158 | 50.7 | 4 | 2 | 1 | 1 | 3 | 4 | 1 | 1 | 2 | 1 | 1 | 1 | 1 | 1 |
| 1962 | 47 | 2 | 158 | 50 | 8 | 2 | 1 | 1 | 2 | 4 | 1 | 1 | 2 | 1 | 1 | 1 | 1 | 1 |
| 1963 | 47 | 2 | 158 | 48 | 4 | 2 | 1 | 1 | 4 | 2 | 1 | 1 | 1 | 1 | 2 | 1 | 1 | 1 |
| 1964 | 47 | 2 | 159 | 70 | 4 | 2 | 1 | 1 | 5 | 3 | 1 | 1 | 1 | 1 | 1 | 1 | 1 | 1 |
| 1965 | 47 | 2 | 159 | 60 | 8 | 2 | 1 | 1 | 4 | 4 | 1 | 1 | 1 | 1 | 1 | 1 | 1 | 1 |
| 1966 | 47 | 2 | 159 | 59 | 8 | 2 | 1 | 1 | 4 | 2 | 1 | 1 | 1 | 1 | 1 | 1 | 1 | 1 |
| 1967 | 47 | 2 | 159 | 44 | 4 | 2 | 1 | 1 | 4 | 2 | 1 | 1 | 1 | 1 | 2 | 1 | 1 | 1 |
| 1968 | 47 | 2 | 160 | 80 | 8 | 2 | 1 | 1 | 6 | 4 | 2 | 1 | 1 | 2 | 1 | 1 | 1 | 2 |
| 1969 | 47 | 2 | 160 | 80 | 8 | 2 | 1 | 1 | 3 | 4 | 1 | 1 | 1 | 1 | 1 | 1 | 1 | 1 |
| 1970 | 47 | 2 | 160 | 65 | 2 | 2 | 1 | 1 | 5 | 4 | 1 | 1 | 2 | 1 | 1 | 1 | 1 | 1 |
| 1971 | 47 | 2 | 160 | 64 | 8 | 2 | 1 | 1 | 4 | 4 | 1 | 1 | 1 | 1 | 1 | 1 | 1 | 1 |
| 1972 | 47 | 2 | 160 | 60 | 8 | 2 | 1 | 1 | 5 | 4 | 1 | 1 | 1 | 1 | 1 | 1 | 1 | 1 |
| 1973 | 47 | 2 | 160 | 60 | 8 | 2 | 1 | 1 | 4 | 4 | 1 | 1 | 2 | 1 | 1 | 1 | 1 | 2 |
| 1974 | 47 | 2 | 160 | 60 | 8 | 1 | 1 | 1 | 4 | 3 | 1 | 1 | 2 | 1 | 1 | 1 | 1 | 1 |
| 1975 | 47 | 2 | 160 | 60 | 8 | 2 | 1 | 1 | 2 | 4 | 1 | 1 | 2 | 1 | 1 | 1 | 1 | 2 |
| 1976 | 47 | 2 | 160 | 59 | 8 | 2 | 1 | 1 | 4 | 2 | 1 | 1 | 1 | 1 | 1 | 1 | 1 | 1 |
| 1977 | 47 | 2 | 160 | 55 | 8 | 2 | 1 | 1 | 4 | 1 | 1 | 1 | 1 | 1 | 1 | 1 | 1 | 1 |
| 1978 | 47 | 2 | 161 | 56 | 8 | 2 | 1 | 1 | 3 | 3 | 1 | 1 | 1 | 1 | 1 | 1 | 1 | 1 |
| 1979 | 47 | 2 | 162 | 69 | 4 | 2 | 1 | 1 | 6 | 3 | 1 | 1 | 1 | 1 | 1 | 1 | 1 | 1 |
| 1980 | 47 | 2 | 162 | 65 | 8 | 2 | 1 | 1 | 4 | 4 | 1 | 1 | 2 | 1 | 1 | 1 | 1 | 1 |
| 1981 | 47 | 2 | 162 | 60 | 3 | 2 | 1 | 1 | 4 | 4 | 1 | 1 | 1 | 1 | 1 | 1 | 1 | 1 |
| 1982 | 47 | 2 | 162 | 55 | 8 | 2 | 1 | 1 | 4 | 3 | 1 | 1 | 1 | 1 | 1 | 1 | 1 | 1 |
| 1983 | 47 | 2 | 162 | 50 | 8 | 2 | 1 | 1 | 3 | 4 | 1 | 1 | 1 | 1 | 1 | 1 | 1 | 2 |
| 1984 | 47 | 2 | 162 | 50 | 8 | 2 | 1 | 1 | 3 | 2 | 2 | 1 | 2 | 1 | 1 | 1 | 3 | 1 |
| 1985 | 47 | 2 | 165 | 55 | 3 | 2 | 1 | 1 | 4 | 4 | 1 | 1 | 1 | 1 | 1 | 1 | 1 | 2 |
| 1986 | 47 | 2 | 167 | 60 | 5 | 2 | 1 | 1 | 3 | 4 | 1 | 1 | 1 | 1 | 1 | 1 | 1 | 2 |
| 1987 | 47 | 2 | 171 | 69 | 8 | 2 | 1 | 1 | 3 | 1 | 1 | 1 | 1 | 1 | 1 | 1 | 1 | 1 |
| 1988 | 47 | 1 | 158 | 46 | 8 | 2 | 1 | 1 | 4 | 4 | 1 | 1 | 2 | 1 | 1 | 1 | 1 | 2 |
| 1989 | 47 | 1 | 161.5 | 52.5 | 2 | 2 | 1 | 1 | 2 | 4 | 1 | 1 | 1 | 1 | 1 | 1 | 1 | 1 |
| 1990 | 47 | 1 | 163 | 65 | 8 | 2 | 1 | 1 | 4 | 3 | 1 | 1 | 1 | 1 | 1 | 1 | 1 | 1 |
| 1991 | 47 | 1 | 163 | 60 | 4 | 2 | 1 | 1 | 4 | 1 | 1 | 1 | 1 | 1 | 1 | 1 | 1 | 2 |
| 1992 | 47 | 1 | 165 | 70 | 8 | 2 | 1 | 1 | 3 | 1 | 1 | 1 | 1 | 1 | 1 | 1 | 1 | 1 |
| 1993 | 47 | 1 | 168 | 55 | 8 | 2 | 1 | 2 | 2 | 3 | 1 | 1 | 1 | 1 | 1 | 1 | 3 | 2 |
| 1994 | 47 | 1 | 170 | 67.5 | 8 | 2 | 1 | 1 | 4 | 4 | 1 | 1 | 1 | 1 | 1 | 1 | 1 | 1 |
| 1995 | 47 | 1 | 170 | 65 | 8 | 2 | 1 | 1 | 4 | 1 | 1 | 1 | 2 | 1 | 1 | 1 | 1 | 1 |
| 1996 | 47 | 1 | 170 | 65 | 8 | 1 | 1 | 1 | 4 | 1 | 1 | 1 | 1 | 1 | 1 | 1 | 3 | 1 |
| 1997 | 47 | 1 | 173 | 65 | 4 | 2 | 1 | 1 | 6 | 3 | 1 | 1 | 1 | 1 | 1 | 1 | 3 | 1 |
| 1998 | 47 | 1 | 175 | 70 | 8 | 2 | 1 | 1 | 3 | 4 | 1 | 1 | 1 | 1 | 1 | 1 | 1 | 1 |
| 1999 | 47 | 1 | 175 | 68 | 4 | 2 | 1 | 1 | 5 | 1 | 1 | 1 | 1 | 1 | 1 | 1 | 1 | 1 |
| 2000 | 47 | 1 | 178 | 88 | 8 | 2 | 1 | 1 | 5 | 3 | 1 | 1 | 1 | 2 | 1 | 1 | 1 | 2 |
| 2001 | 47 | 1 | 184.5 | 73.1 | 8 | 2 | 1 | 2 | 4 | 1 | 1 | 1 | 2 | 1 | 1 | 1 | 3 | 1 |
| 2002 | 48 | 2 | 148 | 50.5 | 5 | 2 | 1 | 1 | 3 | 4 | 1 | 1 | 1 | 1 | 1 | 1 | 1 | 1 |
| 2003 | 48 | 2 | 148.5 | 55 | 8 | 2 | 1 | 1 | 4 | 1 | 1 | 1 | 1 | 1 | 1 | 1 | 1 | 1 |
| 2004 | 48 | 2 | 150 | 56 | 4 | 2 | 1 | 1 | 3 | 4 | 1 | 1 | 1 | 1 | 1 | 1 | 1 | 2 |
| 2005 | 48 | 2 | 150 | 50 | 4 | 2 | 1 | 1 | 3 | 2 | 1 | 1 | 1 | 1 | 1 | 1 | 1 | 1 |
| 2006 | 48 | 2 | 152 | 57.8 | 3 | 1 | 1 | 1 | 5 | 2 | 1 | 1 | 2 | 1 | 2 | 1 | 1 | 1 |
| 2007 | 48 | 2 | 153 | 67 | 4 | 4 | 1 | 1 | 2 | 3 | 2 | 1 | 1 | 1 | 1 | 1 | 1 | 1 |
| 2008 | 48 | 2 | 153 | 62 | 8 | 2 | 1 | 1 | 3 | 3 | 1 | 1 | 1 | 2 | 1 | 1 | 1 | 1 |
| 2009 | 48 | 2 | 153 | 48 | 2 | 2 | 1 | 1 | 3 | 1 | 1 | 1 | 1 | 1 | 1 | 1 | 1 | 1 |
| 2010 | 48 | 2 | 155 | 65 | 8 | 1 | 2 | 1 | 4 | 4 | 1 | 1 | 1 | 1 | 1 | 1 | 1 | 1 |
| 2011 | 48 | 2 | 155 | 63 | 3 | 2 | 1 | 1 | 3 | 1 | 1 | 1 | 1 | 1 | 1 | 1 | 1 | 1 |
| 2012 | 48 | 2 | 155 | 54 | 8 | 2 | 1 | 1 | 4 | 4 | 1 | 1 | 1 | 1 | 1 | 1 | 1 | 1 |
| 2013 | 48 | 2 | 155 | 53 | 4 | 2 | 1 | 1 | 4 | 4 | 1 | 1 | 1 | 1 | 1 | 1 | 1 | 1 |
| 2014 | 48 | 2 | 155 | 46 | 4 | 2 | 1 | 1 | 3 | 3 | 1 | 1 | 1 | 1 | 1 | 1 | 1 | 1 |
| 2015 | 48 | 2 | 156 | 58 | 8 | 2 | 1 | 1 | 4 | 3 | 2 | 1 | 1 | 1 | 1 | 1 | 1 | 2 |
| 2016 | 48 | 2 | 156 | 57 | 8 | 2 | 1 | 1 | 4 | 1 | 1 | 1 | 1 | 1 | 1 | 1 | 1 | 1 |
| 2017 | 48 | 2 | 156 | 51 | 3 | 2 | 1 | 1 | 3 | 3 | 1 | 1 | 1 | 1 | 1 | 1 | 1 | 1 |
| 2018 | 48 | 2 | 156.5 | 65 | 8 | 2 | 1 | 1 | 2 | 4 | 1 | 1 | 1 | 1 | 2 | 1 | 1 | 2 |
| 2019 | 48 | 2 | 156.5 | 62 | 6 | 2 | 1 | 1 | 3 | 1 | 1 | 1 | 1 | 1 | 1 | 1 | 1 | 1 |
| 2020 | 48 | 2 | 157 | 51 | 8 | 2 | 1 | 1 | 6 | 4 | 1 | 1 | 1 | 1 | 1 | 1 | 1 | 1 |
| 2021 | 48 | 2 | 157.5 | 62.8 | 8 | 2 | 1 | 2 | 5 | 3 | 1 | 1 | 2 | 1 | 2 | 2 | 1 | 1 |
| 2022 | 48 | 2 | 158 | 56 | 6 | 2 | 1 | 1 | 4 | 4 | 1 | 1 | 1 | 1 | 1 | 1 | 1 | 1 |
| 2023 | 48 | 2 | 158 | 55 | 8 | 2 | 1 | 1 | 5 | 4 | 1 | 1 | 1 | 1 | 1 | 1 | 1 | 1 |
| 2024 | 48 | 2 | 158 | 54 | 8 | 2 | 1 | 1 | 4 | 4 | 1 | 1 | 1 | 1 | 1 | 1 | 1 | 1 |
| 2025 | 48 | 2 | 158 | 54 | 8 | 2 | 1 | 1 | 3 | 4 | 1 | 1 | 1 | 2 | 1 | 1 | 1 | 2 |
| 2026 | 48 | 2 | 159 | 61.5 | 8 | 2 | 1 | 1 | 3 | 2 | 1 | 1 | 1 | 1 | 1 | 1 | 1 | 1 |
| 2027 | 48 | 2 | 159 | 58 | 8 | 2 | 1 | 1 | 4 | 1 | 3 | 1 | 2 | 1 | 2 | 1 | 1 | 1 |
| 2028 | 48 | 2 | 159 | 45 | 2 | 2 | 1 | 1 | 5 | 4 | 1 | 1 | 2 | 1 | 1 | 1 | 1 | 1 |
| 2029 | 48 | 2 | 160 | 54 | 8 | 2 | 1 | 1 | 3 | 1 | 1 | 1 | 2 | 1 | 1 | 1 | 1 | 1 |
| 2030 | 48 | 2 | 160 | 50 | 6 | 1 | 1 | 1 | 3 | 3 | 1 | 1 | 1 | 1 | 1 | 1 | 1 | 1 |
| 2031 | 48 | 2 | 160 | 45 | 8 | 2 | 1 | 1 | 4 | 4 | 1 | 1 | 1 | 1 | 1 | 1 | 1 | 1 |
| 2032 | 48 | 2 | 161 | 75 | 8 | 2 | 1 | 1 | 4 | 3 | 1 | 1 | 2 | 1 | 1 | 1 | 1 | 1 |
| 2033 | 48 | 2 | 161 | 63 | 8 | 2 | 1 | 1 | 3 | 1 | 1 | 1 | 1 | 1 | 1 | 1 | 1 | 1 |
| 2034 | 48 | 2 | 161 | 60 | 8 | 2 | 1 | 1 | 3 | 3 | 1 | 1 | 1 | 1 | 2 | 1 | 1 | 1 |
| 2035 | 48 | 2 | 162 | 78 | 8 | 2 | 1 | 1 | 3 | 2 | 1 | 1 | 1 | 1 | 1 | 1 | 1 | 1 |
| 2036 | 48 | 2 | 162 | 72 | 8 | 2 | 1 | 1 | 2 | 3 | 1 | 1 | 1 | 1 | 1 | 1 | 1 | 2 |
| 2037 | 48 | 2 | 162 | 63 | 4 | 4 | 1 | 1 | 4 | 3 | 1 | 1 | 1 | 1 | 2 | 1 | 1 | 1 |
| 2038 | 48 | 2 | 162 | 61 | 8 | 2 | 1 | 1 | 4 | 4 | 1 | 1 | 1 | 1 | 1 | 1 | 1 | 1 |
| 2039 | 48 | 2 | 162 | 60 | 8 | 2 | 1 | 1 | 3 | 1 | 1 | 1 | 1 | 1 | 1 | 1 | 1 | 1 |
| 2040 | 48 | 2 | 162 | 52 | 4 | 2 | 1 | 1 | 3 | 1 | 1 | 1 | 1 | 1 | 1 | 1 | 1 | 1 |
| 2041 | 48 | 2 | 163 | 67.5 | 8 | 2 | 1 | 1 | 4 | 4 | 1 | 1 | 1 | 1 | 1 | 1 | 1 | 1 |
| 2042 | 48 | 2 | 165 | 60 | 8 | 2 | 1 | 1 | 5 | 3 | 1 | 1 | 2 | 1 | 1 | 1 | 1 | 1 |
| 2043 | 48 | 2 | 168 | 64 | 2 | 2 | 1 | 1 | 4 | 4 | 1 | 1 | 1 | 1 | 1 | 1 | 1 | 1 |
| 2044 | 48 | 1 | 160 | 45 | 8 | 1 | 1 | 2 | 3 | 1 | 1 | 1 | 1 | 1 | 1 | 1 | 3 | 1 |
| 2045 | 48 | 1 | 168 | 74 | 8 | 2 | 1 | 1 | 4 | 2 | 1 | 1 | 1 | 1 | 1 | 1 | 1 | 1 |
| 2046 | 48 | 1 | 169 | 65.5 | 8 | 2 | 1 | 1 | 3 | 1 | 1 | 1 | 1 | 1 | 2 | 1 | 1 | 2 |
| 2047 | 48 | 1 | 172 | 65 | 8 | 2 | 1 | 1 | 3 | 4 | 1 | 1 | 1 | 1 | 1 | 1 | 3 | 1 |
| 2048 | 48 | 1 | 172 | 65 | 3 | 2 | 1 | 1 | 5 | 2 | 1 | 1 | 1 | 1 | 1 | 1 | 1 | 1 |
| 2049 | 49 | 2 | 146.5 | 49 | 8 | 2 | 1 | 1 | 3 | 3 | 1 | 1 | 1 | 1 | 1 | 1 | 1 | 1 |
| 2050 | 49 | 2 | 148.5 | 61.5 | 8 | 2 | 1 | 1 | 3 | 2 | 1 | 1 | 1 | 1 | 1 | 1 | 1 | 1 |
| 2051 | 49 | 2 | 151 | 57 | 8 | 2 | 1 | 1 | 4 | 4 | 1 | 1 | 2 | 1 | 1 | 1 | 1 | 1 |
| 2052 | 49 | 2 | 152 | 58 | 4 | 2 | 1 | 1 | 2 | 1 | 1 | 1 | 1 | 1 | 1 | 1 | 1 | 1 |
| 2053 | 49 | 2 | 153 | 64.8 | 6 | 2 | 1 | 1 | 3 | 4 | 1 | 1 | 1 | 1 | 1 | 1 | 1 | 1 |
| 2054 | 49 | 2 | 153 | 63.8 | 8 | 2 | 1 | 1 | 3 | 3 | 1 | 1 | 1 | 1 | 1 | 1 | 1 | 1 |
| 2055 | 49 | 2 | 153 | 62.5 | 8 | 3 | 1 | 1 | 3 | 2 | 1 | 1 | 1 | 1 | 1 | 1 | 1 | 1 |
| 2056 | 49 | 2 | 153 | 52 | 8 | 2 | 1 | 1 | 4 | 4 | 1 | 1 | 2 | 1 | 2 | 1 | 1 | 1 |
| 2057 | 49 | 2 | 153 | 42 | 4 | 2 | 1 | 1 | 4 | 4 | 1 | 1 | 2 | 1 | 1 | 1 | 1 | 1 |
| 2058 | 49 | 2 | 154 | 64 | 4 | 2 | 1 | 1 | 4 | 4 | 1 | 1 | 1 | 1 | 1 | 1 | 1 | 1 |
| 2059 | 49 | 2 | 154 | 56 | 8 | 2 | 1 | 1 | 3 | 1 | 1 | 1 | 1 | 1 | 1 | 1 | 1 | 1 |
| 2060 | 49 | 2 | 155 | 57 | 8 | 1 | 1 | 1 | 3 | 4 | 1 | 1 | 1 | 1 | 1 | 1 | 1 | 1 |
| 2061 | 49 | 2 | 155 | 50 | 8 | 2 | 1 | 1 | 3 | 4 | 1 | 1 | 1 | 2 | 1 | 1 | 1 | 2 |
| 2062 | 49 | 2 | 155 | 47 | 8 | 2 | 1 | 1 | 2 | 4 | 1 | 1 | 1 | 1 | 1 | 1 | 1 | 1 |
| 2063 | 49 | 2 | 156 | 62 | 8 | 2 | 1 | 1 | 4 | 4 | 1 | 1 | 1 | 1 | 1 | 1 | 1 | 1 |
| 2064 | 49 | 2 | 156 | 59 | 8 | 2 | 1 | 1 | 3 | 2 | 1 | 1 | 1 | 1 | 1 | 1 | 1 | 1 |
| 2065 | 49 | 2 | 156 | 58.5 | 8 | 2 | 1 | 1 | 4 | 1 | 1 | 1 | 1 | 1 | 1 | 1 | 1 | 1 |
| 2066 | 49 | 2 | 156 | 57 | 8 | 2 | 1 | 1 | 5 | 2 | 1 | 1 | 1 | 2 | 2 | 1 | 1 | 2 |
| 2067 | 49 | 2 | 156 | 55 | 4 | 2 | 1 | 1 | 4 | 3 | 1 | 1 | 2 | 1 | 1 | 1 | 1 | 1 |
| 2068 | 49 | 2 | 156 | 53 | 8 | 3 | 1 | 1 | 2 | 4 | 1 | 1 | 1 | 1 | 1 | 1 | 1 | 1 |
| 2069 | 49 | 2 | 157 | 48.8 | 8 | 2 | 1 | 1 | 3 | 1 | 1 | 1 | 1 | 1 | 1 | 1 | 1 | 1 |
| 2070 | 49 | 2 | 158 | 55 | 3 | 2 | 1 | 2 | 4 | 1 | 1 | 1 | 1 | 1 | 1 | 1 | 1 | 1 |
| 2071 | 49 | 2 | 158 | 52 | 2 | 2 | 1 | 1 | 5 | 3 | 1 | 1 | 1 | 1 | 1 | 1 | 1 | 1 |
| 2072 | 49 | 2 | 159 | 64.7 | 8 | 2 | 1 | 1 | 2 | 4 | 1 | 1 | 1 | 1 | 1 | 1 | 1 | 1 |
| 2073 | 49 | 2 | 159 | 64 | 8 | 2 | 1 | 1 | 4 | 4 | 1 | 1 | 1 | 1 | 1 | 1 | 1 | 1 |
| 2074 | 49 | 2 | 160 | 73 | 2 | 2 | 1 | 1 | 5 | 2 | 1 | 1 | 2 | 1 | 1 | 1 | 1 | 1 |
| 2075 | 49 | 2 | 160 | 70 | 8 | 2 | 1 | 1 | 4 | 4 | 1 | 1 | 1 | 1 | 1 | 1 | 1 | 1 |
| 2076 | 49 | 2 | 160 | 65 | 8 | 2 | 1 | 2 | 3 | 4 | 1 | 1 | 1 | 1 | 1 | 1 | 1 | 1 |
| 2077 | 49 | 2 | 160 | 60 | 8 | 2 | 2 | 2 | 3 | 2 | 1 | 1 | 1 | 1 | 1 | 1 | 1 | 1 |
| 2078 | 49 | 2 | 160 | 58 | 8 | 2 | 1 | 1 | 4 | 1 | 1 | 1 | 1 | 1 | 2 | 1 | 1 | 1 |
| 2079 | 49 | 2 | 160 | 57 | 4 | 2 | 1 | 1 | 3 | 4 | 1 | 1 | 1 | 1 | 1 | 1 | 1 | 1 |
| 2080 | 49 | 2 | 161 | 75 | 8 | 4 | 1 | 1 | 4 | 4 | 1 | 1 | 2 | 1 | 1 | 1 | 1 | 1 |
| 2081 | 49 | 2 | 162 | 58 | 8 | 2 | 1 | 1 | 3 | 3 | 1 | 1 | 1 | 1 | 1 | 1 | 1 | 1 |
| 2082 | 49 | 2 | 162 | 55 | 4 | 2 | 1 | 1 | 4 | 3 | 1 | 1 | 1 | 2 | 1 | 2 | 1 | 2 |
| 2083 | 49 | 2 | 163 | 71 | 8 | 2 | 1 | 1 | 3 | 1 | 1 | 1 | 1 | 1 | 1 | 1 | 1 | 1 |
| 2084 | 49 | 2 | 164 | 67 | 8 | 2 | 1 | 1 | 4 | 1 | 1 | 1 | 1 | 1 | 1 | 1 | 1 | 1 |
| 2085 | 49 | 2 | 164 | 63 | 2 | 2 | 1 | 1 | 3 | 1 | 1 | 1 | 1 | 1 | 1 | 1 | 1 | 1 |
| 2086 | 49 | 2 | 164.5 | 39 | 8 | 1 | 1 | 1 | 1 | 1 | 1 | 1 | 1 | 1 | 1 | 1 | 3 | 1 |
| 2087 | 49 | 2 | 167 | 68 | 3 | 2 | 1 | 1 | 3 | 4 | 1 | 1 | 1 | 1 | 1 | 1 | 1 | 1 |
| 2088 | 49 | 1 | 160 | 55 | 8 | 2 | 1 | 1 | 5 | 3 | 1 | 1 | 2 | 1 | 1 | 1 | 1 | 1 |
| 2089 | 49 | 1 | 161 | 67 | 4 | 2 | 1 | 1 | 5 | 3 | 1 | 1 | 1 | 1 | 1 | 1 | 3 | 1 |
| 2090 | 49 | 1 | 162 | 70 | 5 | 2 | 2 | 1 | 3 | 1 | 1 | 1 | 1 | 1 | 1 | 1 | 1 | 1 |
| 2091 | 49 | 1 | 165 | 70 | 6 | 2 | 1 | 2 | 3 | 2 | 1 | 1 | 1 | 1 | 1 | 1 | 1 | 1 |
| 2092 | 49 | 1 | 165 | 63.6 | 6 | 3 | 1 | 2 | 3 | 3 | 1 | 1 | 1 | 1 | 1 | 1 | 3 | 1 |
| 2093 | 49 | 1 | 171 | 64 | 2 | 2 | 1 | 1 | 4 | 1 | 1 | 1 | 2 | 1 | 1 | 1 | 1 | 1 |
| 2094 | 49 | 1 | 173 | 68 | 8 | 2 | 1 | 1 | 2 | 3 | 1 | 1 | 1 | 1 | 1 | 1 | 1 | 1 |
| 2095 | 49 | 1 | 176 | 59.7 | 8 | 2 | 1 | 1 | 3 | 4 | 1 | 1 | 1 | 1 | 1 | 1 | 3 | 1 |
| 2096 | 49 | 1 | 178 | 82 | 4 | 2 | 1 | 2 | 3 | 4 | 1 | 1 | 1 | 1 | 1 | 1 | 3 | 1 |
| 2097 | 50 | 2 | 148 | 50 | 3 | 1 | 1 | 1 | 4 | 3 | 1 | 1 | 1 | 1 | 1 | 1 | 1 | 1 |
| 2098 | 50 | 2 | 148.5 | 55.5 | 4 | 2 | 1 | 1 | 3 | 4 | 1 | 1 | 1 | 1 | 1 | 1 | 1 | 1 |
| 2099 | 50 | 2 | 150 | 57 | 8 | 4 | 2 | 1 | 4 | 4 | 1 | 1 | 1 | 1 | 1 | 1 | 1 | 2 |
| 2100 | 50 | 2 | 150 | 56 | 8 | 1 | 2 | 1 | 3 | 3 | 1 | 1 | 1 | 1 | 1 | 1 | 1 | 1 |
| 2101 | 50 | 2 | 150 | 55 | 8 | 2 | 1 | 1 | 6 | 4 | 1 | 1 | 1 | 1 | 1 | 1 | 1 | 1 |
| 2102 | 50 | 2 | 150 | 55 | 8 | 2 | 1 | 1 | 3 | 4 | 1 | 1 | 1 | 1 | 1 | 1 | 1 | 1 |
| 2103 | 50 | 2 | 150 | 55 | 4 | 2 | 1 | 1 | 6 | 3 | 1 | 1 | 1 | 1 | 1 | 1 | 1 | 1 |
| 2104 | 50 | 2 | 150 | 45 | 8 | 2 | 1 | 1 | 6 | 2 | 1 | 1 | 1 | 1 | 1 | 1 | 1 | 1 |
| 2105 | 50 | 2 | 150 | 45 | 8 | 2 | 1 | 1 | 4 | 4 | 1 | 1 | 1 | 1 | 1 | 1 | 1 | 1 |
| 2106 | 50 | 2 | 150 | 41 | 8 | 1 | 1 | 1 | 4 | 4 | 1 | 1 | 1 | 1 | 1 | 1 | 1 | 1 |
| 2107 | 50 | 2 | 150 | 40 | 8 | 2 | 1 | 1 | 3 | 2 | 1 | 1 | 1 | 1 | 1 | 2 | 1 | 1 |
| 2108 | 50 | 2 | 152 | 46 | 8 | 2 | 1 | 1 | 4 | 3 | 1 | 1 | 1 | 1 | 1 | 1 | 1 | 1 |
| 2109 | 50 | 2 | 152 | 45 | 4 | 4 | 1 | 1 | 4 | 2 | 1 | 1 | 1 | 1 | 1 | 1 | 1 | 1 |
| 2110 | 50 | 2 | 153 | 58 | 8 | 2 | 1 | 1 | 3 | 1 | 1 | 1 | 1 | 1 | 2 | 1 | 1 | 1 |
| 2111 | 50 | 2 | 154 | 62 | 4 | 2 | 1 | 1 | 4 | 4 | 1 | 1 | 1 | 1 | 1 | 1 | 1 | 1 |
| 2112 | 50 | 2 | 154 | 53 | 8 | 2 | 1 | 1 | 4 | 4 | 1 | 1 | 1 | 1 | 1 | 1 | 1 | 1 |
| 2113 | 50 | 2 | 155 | 59 | 4 | 2 | 1 | 1 | 3 | 4 | 1 | 1 | 2 | 1 | 1 | 1 | 1 | 2 |
| 2114 | 50 | 2 | 155 | 51.5 | 8 | 2 | 1 | 1 | 4 | 4 | 1 | 1 | 1 | 1 | 1 | 1 | 1 | 1 |
| 2115 | 50 | 2 | 155 | 51 | 6 | 2 | 1 | 1 | 4 | 3 | 1 | 1 | 1 | 1 | 1 | 1 | 1 | 2 |
| 2116 | 50 | 2 | 155 | 48 | 8 | 2 | 1 | 1 | 3 | 4 | 1 | 1 | 1 | 1 | 1 | 1 | 1 | 1 |
| 2117 | 50 | 2 | 155 | 47 | 8 | 2 | 1 | 1 | 5 | 4 | 1 | 1 | 1 | 1 | 1 | 1 | 1 | 1 |
| 2118 | 50 | 2 | 155 | 45 | 2 | 2 | 1 | 1 | 4 | 2 | 1 | 1 | 2 | 1 | 1 | 1 | 1 | 1 |
| 2119 | 50 | 2 | 156 | 65 | 8 | 2 | 1 | 1 | 3 | 1 | 1 | 1 | 1 | 1 | 1 | 1 | 1 | 2 |
| 2120 | 50 | 2 | 156 | 63 | 4 | 2 | 1 | 1 | 4 | 3 | 1 | 1 | 1 | 1 | 1 | 1 | 1 | 1 |
| 2121 | 50 | 2 | 156 | 61 | 8 | 2 | 1 | 1 | 5 | 4 | 1 | 1 | 1 | 1 | 1 | 1 | 1 | 1 |
| 2122 | 50 | 2 | 156 | 60 | 4 | 2 | 1 | 1 | 3 | 2 | 1 | 1 | 1 | 1 | 1 | 1 | 1 | 1 |
| 2123 | 50 | 2 | 156 | 59 | 8 | 2 | 2 | 1 | 3 | 3 | 1 | 1 | 2 | 1 | 1 | 1 | 1 | 2 |
| 2124 | 50 | 2 | 156 | 55 | 8 | 2 | 1 | 1 | 5 | 4 | 1 | 1 | 1 | 1 | 1 | 1 | 1 | 2 |
| 2125 | 50 | 2 | 156 | 53 | 8 | 2 | 1 | 1 | 3 | 4 | 1 | 1 | 1 | 1 | 1 | 1 | 1 | 2 |
| 2126 | 50 | 2 | 156.8 | 64 | 3 | 2 | 1 | 1 | 4 | 1 | 1 | 1 | 1 | 1 | 1 | 1 | 1 | 1 |
| 2127 | 50 | 2 | 157 | 54 | 8 | 2 | 1 | 1 | 3 | 3 | 1 | 1 | 1 | 1 | 1 | 1 | 1 | 1 |
| 2128 | 50 | 2 | 157 | 52.5 | 8 | 2 | 1 | 1 | 5 | 2 | 1 | 1 | 2 | 1 | 1 | 1 | 1 | 1 |
| 2129 | 50 | 2 | 158 | 62 | 8 | 2 | 1 | 1 | 2 | 4 | 1 | 1 | 1 | 1 | 2 | 1 | 1 | 1 |
| 2130 | 50 | 2 | 158 | 61.5 | 8 | 1 | 1 | 1 | 4 | 2 | 1 | 1 | 1 | 1 | 1 | 1 | 1 | 1 |
| 2131 | 50 | 2 | 158 | 61 | 8 | 2 | 1 | 1 | 4 | 2 | 1 | 1 | 1 | 1 | 2 | 1 | 1 | 1 |
| 2132 | 50 | 2 | 158 | 60 | 8 | 2 | 1 | 1 | 4 | 4 | 1 | 1 | 1 | 1 | 1 | 1 | 1 | 1 |
| 2133 | 50 | 2 | 158 | 60 | 8 | 2 | 1 | 1 | 2 | 4 | 1 | 1 | 1 | 1 | 1 | 1 | 1 | 2 |
| 2134 | 50 | 2 | 158 | 55 | 3 | 2 | 1 | 1 | 3 | 3 | 1 | 1 | 1 | 1 | 1 | 1 | 1 | 1 |
| 2135 | 50 | 2 | 159 | 69.5 | 8 | 2 | 1 | 1 | 3 | 3 | 1 | 1 | 1 | 1 | 2 | 1 | 1 | 1 |
| 2136 | 50 | 2 | 159 | 60 | 8 | 2 | 1 | 1 | 3 | 1 | 1 | 1 | 1 | 1 | 1 | 1 | 1 | 2 |
| 2137 | 50 | 2 | 159 | 59 | 8 | 2 | 1 | 1 | 4 | 1 | 1 | 1 | 1 | 1 | 1 | 1 | 1 | 1 |
| 2138 | 50 | 2 | 159 | 58 | 2 | 2 | 1 | 1 | 3 | 3 | 1 | 1 | 1 | 1 | 1 | 1 | 1 | 1 |
| 2139 | 50 | 2 | 159 | 49 | 4 | 2 | 1 | 1 | 4 | 2 | 1 | 1 | 1 | 2 | 1 | 1 | 1 | 2 |
| 2140 | 50 | 2 | 159 | 46 | 3 | 2 | 1 | 1 | 4 | 1 | 1 | 1 | 1 | 1 | 2 | 1 | 1 | 1 |
| 2141 | 50 | 2 | 160 | 72.5 | 8 | 2 | 1 | 1 | 3 | 3 | 1 | 1 | 1 | 1 | 1 | 1 | 1 | 1 |
| 2142 | 50 | 2 | 160 | 70 | 8 | 2 | 1 | 1 | 4 | 2 | 1 | 1 | 2 | 1 | 1 | 1 | 1 | 1 |
| 2143 | 50 | 2 | 160 | 70 | 8 | 2 | 1 | 1 | 3 | 3 | 1 | 1 | 1 | 1 | 2 | 1 | 1 | 1 |
| 2144 | 50 | 2 | 160 | 64 | 8 | 2 | 1 | 1 | 5 | 1 | 1 | 1 | 1 | 1 | 1 | 1 | 1 | 1 |
| 2145 | 50 | 2 | 160 | 62.5 | 4 | 2 | 1 | 1 | 3 | 3 | 1 | 1 | 1 | 1 | 1 | 1 | 1 | 1 |
| 2146 | 50 | 2 | 160 | 62 | 8 | 2 | 1 | 1 | 4 | 4 | 1 | 1 | 1 | 1 | 1 | 1 | 1 | 1 |
| 2147 | 50 | 2 | 160 | 61.5 | 8 | 1 | 1 | 1 | 3 | 4 | 1 | 1 | 1 | 1 | 1 | 1 | 1 | 1 |
| 2148 | 50 | 2 | 160 | 61 | 8 | 2 | 1 | 1 | 3 | 1 | 1 | 1 | 1 | 1 | 1 | 1 | 1 | 1 |
| 2149 | 50 | 2 | 161 | 66 | 8 | 4 | 1 | 1 | 3 | 1 | 1 | 1 | 1 | 1 | 1 | 1 | 1 | 1 |
| 2150 | 50 | 2 | 161 | 61.5 | 4 | 2 | 1 | 1 | 3 | 4 | 1 | 1 | 1 | 1 | 1 | 1 | 1 | 1 |
| 2151 | 50 | 2 | 161 | 61 | 8 | 2 | 2 | 1 | 3 | 2 | 1 | 1 | 1 | 1 | 1 | 1 | 1 | 1 |
| 2152 | 50 | 2 | 161.5 | 55 | 8 | 2 | 1 | 1 | 4 | 1 | 1 | 1 | 1 | 1 | 1 | 1 | 1 | 1 |
| 2153 | 50 | 2 | 162 | 57 | 8 | 2 | 1 | 1 | 4 | 4 | 1 | 1 | 1 | 1 | 1 | 1 | 1 | 2 |
| 2154 | 50 | 2 | 162 | 55 | 8 | 2 | 1 | 1 | 4 | 1 | 1 | 1 | 1 | 1 | 1 | 1 | 1 | 1 |
| 2155 | 50 | 2 | 162.5 | 57 | 8 | 2 | 1 | 1 | 5 | 4 | 1 | 1 | 1 | 1 | 2 | 1 | 1 | 1 |
| 2156 | 50 | 2 | 163 | 73.5 | 8 | 3 | 1 | 1 | 3 | 3 | 1 | 1 | 1 | 1 | 1 | 1 | 1 | 1 |
| 2157 | 50 | 2 | 163 | 70 | 8 | 2 | 1 | 1 | 4 | 1 | 1 | 1 | 2 | 1 | 1 | 1 | 1 | 1 |
| 2158 | 50 | 2 | 164 | 73 | 8 | 2 | 1 | 1 | 4 | 3 | 1 | 1 | 1 | 1 | 1 | 1 | 1 | 2 |
| 2159 | 50 | 2 | 164 | 70.5 | 1 | 2 | 1 | 1 | 4 | 2 | 1 | 1 | 1 | 1 | 1 | 1 | 1 | 1 |
| 2160 | 50 | 2 | 164 | 50 | 3 | 2 | 1 | 1 | 4 | 4 | 1 | 1 | 2 | 1 | 1 | 1 | 1 | 1 |
| 2161 | 50 | 2 | 165 | 65 | 8 | 2 | 2 | 1 | 5 | 4 | 1 | 1 | 1 | 1 | 1 | 1 | 1 | 1 |
| 2162 | 50 | 2 | 165.5 | 51.6 | 8 | 2 | 1 | 1 | 3 | 4 | 1 | 1 | 1 | 1 | 1 | 1 | 1 | 1 |
| 2163 | 50 | 2 | 166 | 55 | 8 | 2 | 1 | 2 | 3 | 2 | 1 | 1 | 1 | 1 | 1 | 1 | 1 | 1 |
| 2164 | 50 | 2 | 167 | 70 | 2 | 1 | 1 | 1 | 2 | 4 | 1 | 1 | 1 | 1 | 1 | 1 | 1 | 2 |
| 2165 | 50 | 2 | 168 | 80 | 8 | 2 | 1 | 1 | 2 | 4 | 1 | 1 | 1 | 1 | 2 | 1 | 1 | 1 |
| 2166 | 50 | 2 | 168 | 57 | 8 | 2 | 1 | 1 | 3 | 3 | 1 | 1 | 2 | 1 | 1 | 1 | 1 | 1 |
| 2167 | 50 | 2 | 169 | 64 | 8 | 2 | 1 | 1 | 4 | 4 | 1 | 1 | 1 | 1 | 1 | 1 | 1 | 1 |
| 2168 | 50 | 2 | 169 | 62 | 8 | 2 | 1 | 1 | 5 | 4 | 1 | 1 | 1 | 1 | 1 | 1 | 1 | 1 |
| 2169 | 50 | 2 | 173 | 70 | 8 | 2 | 1 | 1 | 3 | 4 | 1 | 1 | 2 | 1 | 1 | 1 | 1 | 1 |
| 2170 | 50 | 2 | 186 | 68 | 8 | 2 | 1 | 1 | 4 | 3 | 1 | 1 | 1 | 1 | 1 | 1 | 1 | 1 |
| 2171 | 50 | 1 | 165 | 67 | 8 | 2 | 1 | 2 | 4 | 3 | 1 | 1 | 1 | 1 | 1 | 1 | 3 | 1 |
| 2172 | 50 | 1 | 168 | 60 | 8 | 2 | 1 | 1 | 5 | 4 | 1 | 1 | 1 | 1 | 2 | 1 | 1 | 1 |
| 2173 | 50 | 1 | 169 | 75 | 6 | 2 | 1 | 2 | 3 | 1 | 1 | 1 | 1 | 2 | 1 | 1 | 2 | 1 |
| 2174 | 50 | 1 | 169 | 58.2 | 8 | 2 | 1 | 1 | 3 | 1 | 1 | 1 | 1 | 1 | 1 | 1 | 3 | 1 |
| 2175 | 50 | 1 | 170 | 90 | 8 | 2 | 1 | 1 | 4 | 3 | 1 | 1 | 1 | 1 | 1 | 1 | 3 | 1 |
| 2176 | 50 | 1 | 170 | 73.2 | 8 | 4 | 1 | 1 | 5 | 2 | 1 | 1 | 1 | 1 | 1 | 1 | 1 | 1 |
| 2177 | 50 | 1 | 170 | 68 | 4 | 2 | 1 | 1 | 4 | 4 | 1 | 1 | 1 | 2 | 1 | 1 | 1 | 1 |
| 2178 | 50 | 1 | 170 | 68 | 3 | 2 | 1 | 3 | 4 | 1 | 1 | 1 | 1 | 1 | 1 | 1 | 3 | 1 |
| 2179 | 50 | 1 | 170 | 55 | 8 | 2 | 1 | 1 | 3 | 4 | 1 | 1 | 1 | 1 | 1 | 1 | 1 | 1 |
| 2180 | 50 | 1 | 171 | 60 | 8 | 2 | 1 | 1 | 5 | 3 | 1 | 1 | 1 | 1 | 1 | 1 | 1 | 1 |
| 2181 | 50 | 1 | 173 | 70 | 8 | 2 | 1 | 2 | 3 | 4 | 1 | 1 | 1 | 1 | 1 | 1 | 1 | 1 |
| 2182 | 50 | 1 | 173 | 55 | 8 | 2 | 1 | 1 | 5 | 1 | 1 | 1 | 1 | 2 | 2 | 1 | 2 | 2 |
| 2183 | 50 | 1 | 175 | 59 | 8 | 2 | 1 | 1 | 3 | 3 | 1 | 1 | 1 | 1 | 1 | 1 | 1 | 1 |
| 2184 | 50 | 1 | 176 | 60 | 8 | 2 | 1 | 1 | 3 | 4 | 1 | 1 | 1 | 1 | 1 | 1 | 1 | 1 |
| 2185 | 50 | 1 | 177 | 73.5 | 8 | 2 | 1 | 2 | 4 | 3 | 1 | 1 | 1 | 1 | 1 | 1 | 1 | 1 |
| 2186 | 50 | 1 | 182 | 88 | 8 | 2 | 1 | 1 | 4 | 3 | 1 | 1 | 1 | 1 | 1 | 1 | 3 | 1 |
| 2187 | 51 | 2 | 148 | 49 | 8 | 2 | 1 | 1 | 4 | 3 | 1 | 1 | 2 | 1 | 1 | 1 | 1 | 1 |
| 2188 | 51 | 2 | 149 | 52.7 | 6 | 2 | 1 | 1 | 3 | 1 | 1 | 1 | 1 | 1 | 1 | 1 | 1 | 1 |
| 2189 | 51 | 2 | 149.5 | 61 | 8 | 2 | 1 | 1 | 2 | 4 | 1 | 1 | 1 | 1 | 1 | 1 | 1 | 1 |
| 2190 | 51 | 2 | 150 | 52 | 8 | 2 | 1 | 1 | 3 | 3 | 1 | 1 | 1 | 1 | 2 | 1 | 1 | 1 |
| 2191 | 51 | 2 | 150 | 51 | 8 | 2 | 1 | 1 | 4 | 4 | 1 | 1 | 1 | 1 | 1 | 1 | 1 | 1 |
| 2192 | 51 | 2 | 150 | 48 | 8 | 2 | 1 | 1 | 3 | 4 | 1 | 1 | 1 | 1 | 1 | 1 | 1 | 1 |
| 2193 | 51 | 2 | 150 | 46 | 8 | 2 | 1 | 1 | 4 | 3 | 1 | 1 | 1 | 1 | 1 | 1 | 1 | 1 |
| 2194 | 51 | 2 | 150 | 46 | 8 | 2 | 1 | 1 | 3 | 4 | 1 | 1 | 1 | 1 | 1 | 1 | 1 | 1 |
| 2195 | 51 | 2 | 150 | 46 | 8 | 2 | 1 | 1 | 3 | 1 | 1 | 1 | 1 | 1 | 1 | 1 | 1 | 1 |
| 2196 | 51 | 2 | 150 | 43 | 8 | 2 | 1 | 1 | 5 | 2 | 1 | 1 | 2 | 1 | 1 | 1 | 1 | 1 |
| 2197 | 51 | 2 | 151 | 67 | 8 | 2 | 1 | 1 | 4 | 4 | 1 | 1 | 1 | 1 | 1 | 1 | 1 | 1 |
| 2198 | 51 | 2 | 151 | 60 | 3 | 2 | 1 | 1 | 4 | 4 | 1 | 1 | 1 | 1 | 1 | 1 | 1 | 1 |
| 2199 | 51 | 2 | 151 | 50 | 8 | 2 | 1 | 1 | 2 | 2 | 1 | 1 | 1 | 1 | 1 | 1 | 1 | 1 |
| 2200 | 51 | 2 | 151 | 48.5 | 8 | 2 | 1 | 1 | 3 | 1 | 1 | 1 | 2 | 1 | 1 | 1 | 1 | 1 |
| 2201 | 51 | 2 | 151 | 45 | 8 | 2 | 1 | 1 | 4 | 4 | 1 | 1 | 1 | 1 | 1 | 1 | 1 | 1 |
| 2202 | 51 | 2 | 152 | 68 | 6 | 2 | 1 | 1 | 3 | 2 | 1 | 1 | 1 | 1 | 1 | 1 | 1 | 1 |
| 2203 | 51 | 2 | 152 | 55 | 8 | 2 | 1 | 1 | 4 | 4 | 1 | 1 | 1 | 1 | 1 | 1 | 1 | 1 |
| 2204 | 51 | 2 | 152 | 50 | 8 | 2 | 1 | 1 | 4 | 1 | 1 | 1 | 1 | 1 | 1 | 1 | 1 | 1 |
| 2205 | 51 | 2 | 152 | 50 | 3 | 2 | 1 | 1 | 3 | 4 | 1 | 1 | 2 | 1 | 1 | 1 | 1 | 2 |
| 2206 | 51 | 2 | 153 | 61 | 1 | 2 | 1 | 1 | 3 | 1 | 1 | 1 | 1 | 1 | 1 | 1 | 1 | 1 |
| 2207 | 51 | 2 | 153 | 54 | 8 | 2 | 1 | 1 | 4 | 1 | 2 | 1 | 1 | 1 | 1 | 1 | 1 | 2 |
| 2208 | 51 | 2 | 153 | 51.5 | 8 | 2 | 1 | 1 | 4 | 2 | 1 | 1 | 1 | 1 | 1 | 1 | 1 | 1 |
| 2209 | 51 | 2 | 154 | 81.8 | 8 | 2 | 1 | 1 | 3 | 2 | 1 | 1 | 1 | 1 | 1 | 1 | 1 | 1 |
| 2210 | 51 | 2 | 154 | 60 | 8 | 2 | 1 | 1 | 4 | 4 | 1 | 1 | 1 | 1 | 1 | 1 | 1 | 1 |
| 2211 | 51 | 2 | 154 | 58 | 8 | 2 | 1 | 1 | 3 | 1 | 1 | 1 | 1 | 1 | 1 | 1 | 1 | 1 |
| 2212 | 51 | 2 | 154.5 | 61.9 | 6 | 2 | 1 | 1 | 3 | 1 | 1 | 1 | 1 | 1 | 1 | 1 | 1 | 1 |
| 2213 | 51 | 2 | 155 | 67 | 8 | 2 | 1 | 1 | 4 | 1 | 1 | 1 | 1 | 1 | 1 | 1 | 1 | 1 |
| 2214 | 51 | 2 | 155 | 65 | 8 | 2 | 1 | 1 | 4 | 1 | 1 | 2 | 1 | 1 | 1 | 2 | 1 | 1 |
| 2215 | 51 | 2 | 155 | 65 | 8 | 2 | 1 | 1 | 4 | 1 | 1 | 1 | 2 | 1 | 1 | 1 | 1 | 1 |
| 2216 | 51 | 2 | 155 | 61.5 | 8 | 2 | 1 | 1 | 3 | 1 | 1 | 1 | 2 | 1 | 1 | 1 | 1 | 1 |
| 2217 | 51 | 2 | 155 | 56 | 3 | 2 | 1 | 1 | 6 | 4 | 1 | 1 | 1 | 1 | 1 | 1 | 1 | 1 |
| 2218 | 51 | 2 | 155 | 55 | 8 | 2 | 1 | 1 | 6 | 4 | 1 | 1 | 1 | 1 | 1 | 1 | 1 | 1 |
| 2219 | 51 | 2 | 155 | 50 | 8 | 2 | 1 | 1 | 4 | 4 | 1 | 1 | 1 | 1 | 1 | 1 | 1 | 1 |
| 2220 | 51 | 2 | 155 | 48 | 8 | 1 | 1 | 1 | 5 | 3 | 1 | 1 | 1 | 1 | 1 | 1 | 1 | 1 |
| 2221 | 51 | 2 | 156 | 66 | 6 | 2 | 1 | 2 | 4 | 1 | 1 | 1 | 1 | 1 | 1 | 1 | 1 | 1 |
| 2222 | 51 | 2 | 156 | 55 | 8 | 2 | 1 | 1 | 5 | 1 | 1 | 1 | 2 | 1 | 1 | 1 | 1 | 1 |
| 2223 | 51 | 2 | 156 | 54 | 8 | 2 | 1 | 1 | 2 | 4 | 1 | 1 | 1 | 1 | 1 | 1 | 1 | 2 |
| 2224 | 51 | 2 | 156 | 54 | 4 | 2 | 1 | 1 | 4 | 4 | 1 | 1 | 1 | 1 | 1 | 1 | 1 | 1 |
| 2225 | 51 | 2 | 156 | 52 | 8 | 2 | 2 | 1 | 4 | 1 | 1 | 1 | 1 | 1 | 1 | 1 | 1 | 1 |
| 2226 | 51 | 2 | 157 | 58 | 8 | 2 | 1 | 1 | 4 | 1 | 1 | 1 | 1 | 2 | 2 | 1 | 1 | 1 |
| 2227 | 51 | 2 | 157 | 55 | 3 | 2 | 1 | 1 | 4 | 3 | 1 | 1 | 1 | 1 | 1 | 1 | 1 | 1 |
| 2228 | 51 | 2 | 157 | 50 | 1 | 2 | 1 | 1 | 5 | 3 | 1 | 1 | 1 | 1 | 1 | 1 | 1 | 1 |
| 2229 | 51 | 2 | 157.5 | 59.9 | 8 | 2 | 1 | 1 | 4 | 1 | 1 | 1 | 1 | 1 | 1 | 1 | 1 | 1 |
| 2230 | 51 | 2 | 158 | 84 | 8 | 2 | 1 | 1 | 4 | 4 | 1 | 1 | 1 | 1 | 1 | 1 | 1 | 1 |
| 2231 | 51 | 2 | 158 | 72 | 4 | 2 | 1 | 1 | 3 | 3 | 1 | 1 | 1 | 1 | 2 | 1 | 1 | 1 |
| 2232 | 51 | 2 | 158 | 63 | 8 | 2 | 1 | 1 | 4 | 1 | 1 | 1 | 2 | 1 | 1 | 1 | 1 | 1 |
| 2233 | 51 | 2 | 158 | 60 | 8 | 2 | 2 | 1 | 4 | 1 | 1 | 1 | 1 | 1 | 1 | 1 | 1 | 1 |
| 2234 | 51 | 2 | 158 | 60 | 8 | 2 | 1 | 1 | 6 | 3 | 1 | 1 | 2 | 1 | 1 | 1 | 1 | 1 |
| 2235 | 51 | 2 | 158 | 59 | 4 | 2 | 1 | 1 | 3 | 1 | 1 | 1 | 1 | 1 | 1 | 1 | 1 | 1 |
| 2236 | 51 | 2 | 158 | 55 | 8 | 2 | 1 | 1 | 4 | 3 | 1 | 1 | 1 | 1 | 1 | 1 | 1 | 1 |
| 2237 | 51 | 2 | 158 | 55 | 8 | 2 | 1 | 1 | 4 | 2 | 1 | 1 | 1 | 1 | 2 | 1 | 1 | 1 |
| 2238 | 51 | 2 | 158 | 52 | 8 | 2 | 1 | 1 | 4 | 3 | 1 | 1 | 1 | 1 | 1 | 1 | 1 | 1 |
| 2239 | 51 | 2 | 158 | 49 | 2 | 2 | 1 | 1 | 4 | 1 | 1 | 1 | 1 | 1 | 1 | 1 | 1 | 1 |
| 2240 | 51 | 2 | 158 | 49 | 1 | 2 | 1 | 1 | 4 | 1 | 1 | 1 | 2 | 1 | 1 | 1 | 1 | 1 |
| 2241 | 51 | 2 | 159 | 130 | 8 | 2 | 1 | 1 | 3 | 4 | 1 | 1 | 1 | 1 | 1 | 1 | 1 | 1 |
| 2242 | 51 | 2 | 159 | 60 | 8 | 2 | 1 | 1 | 6 | 4 | 1 | 1 | 1 | 1 | 1 | 1 | 1 | 1 |
| 2243 | 51 | 2 | 159 | 56 | 8 | 1 | 1 | 1 | 3 | 4 | 1 | 1 | 2 | 1 | 2 | 1 | 1 | 1 |
| 2244 | 51 | 2 | 160 | 82 | 8 | 2 | 1 | 1 | 4 | 3 | 1 | 1 | 1 | 1 | 1 | 1 | 1 | 1 |
| 2245 | 51 | 2 | 160 | 70 | 6 | 2 | 1 | 1 | 3 | 1 | 1 | 1 | 1 | 2 | 1 | 1 | 1 | 2 |
| 2246 | 51 | 2 | 160 | 69 | 2 | 1 | 1 | 1 | 4 | 4 | 1 | 1 | 1 | 1 | 1 | 1 | 1 | 1 |
| 2247 | 51 | 2 | 160 | 66 | 8 | 2 | 1 | 1 | 4 | 4 | 1 | 1 | 1 | 1 | 1 | 1 | 1 | 1 |
| 2248 | 51 | 2 | 160 | 63 | 8 | 2 | 2 | 1 | 6 | 4 | 1 | 1 | 1 | 1 | 1 | 1 | 1 | 1 |
| 2249 | 51 | 2 | 160 | 60 | 8 | 2 | 1 | 1 | 4 | 4 | 1 | 1 | 2 | 1 | 1 | 1 | 1 | 1 |
| 2250 | 51 | 2 | 160 | 60 | 8 | 2 | 1 | 1 | 4 | 4 | 1 | 1 | 1 | 1 | 1 | 1 | 1 | 1 |
| 2251 | 51 | 2 | 160 | 60 | 3 | 2 | 1 | 1 | 4 | 4 | 1 | 1 | 1 | 1 | 1 | 1 | 1 | 1 |
| 2252 | 51 | 2 | 160 | 59 | 8 | 2 | 1 | 1 | 3 | 1 | 1 | 1 | 1 | 1 | 1 | 1 | 1 | 1 |
| 2253 | 51 | 2 | 160 | 57 | 8 | 2 | 1 | 1 | 4 | 4 | 1 | 1 | 1 | 1 | 1 | 1 | 1 | 1 |
| 2254 | 51 | 2 | 160 | 55 | 8 | 3 | 1 | 1 | 5 | 2 | 1 | 1 | 1 | 1 | 1 | 1 | 1 | 1 |
| 2255 | 51 | 2 | 160 | 55 | 8 | 2 | 1 | 1 | 6 | 4 | 1 | 1 | 2 | 1 | 1 | 1 | 1 | 1 |
| 2256 | 51 | 2 | 160 | 55 | 8 | 2 | 1 | 1 | 4 | 4 | 1 | 1 | 1 | 1 | 1 | 1 | 1 | 2 |
| 2257 | 51 | 2 | 160 | 55 | 8 | 2 | 1 | 1 | 3 | 1 | 1 | 1 | 1 | 1 | 1 | 1 | 1 | 1 |
| 2258 | 51 | 2 | 160 | 52 | 3 | 2 | 1 | 1 | 4 | 3 | 1 | 1 | 2 | 1 | 2 | 1 | 1 | 1 |
| 2259 | 51 | 2 | 160 | 50 | 4 | 2 | 1 | 1 | 6 | 1 | 1 | 1 | 1 | 1 | 1 | 1 | 1 | 1 |
| 2260 | 51 | 2 | 160 | 46 | 8 | 2 | 1 | 1 | 3 | 3 | 1 | 1 | 2 | 1 | 1 | 1 | 1 | 1 |
| 2261 | 51 | 2 | 160 | 45 | 8 | 2 | 1 | 1 | 3 | 4 | 1 | 1 | 1 | 1 | 1 | 1 | 1 | 1 |
| 2262 | 51 | 2 | 161 | 68 | 8 | 2 | 1 | 1 | 3 | 4 | 1 | 1 | 1 | 1 | 1 | 1 | 1 | 1 |
| 2263 | 51 | 2 | 161 | 67 | 8 | 2 | 1 | 1 | 4 | 4 | 1 | 1 | 1 | 1 | 2 | 1 | 1 | 1 |
| 2264 | 51 | 2 | 161 | 60 | 8 | 2 | 1 | 1 | 4 | 3 | 1 | 1 | 2 | 1 | 1 | 1 | 1 | 1 |
| 2265 | 51 | 2 | 161 | 53 | 8 | 2 | 1 | 1 | 4 | 1 | 1 | 1 | 1 | 1 | 1 | 1 | 1 | 1 |
| 2266 | 51 | 2 | 161 | 53 | 8 | 2 | 1 | 1 | 3 | 3 | 1 | 1 | 2 | 1 | 1 | 1 | 1 | 1 |
| 2267 | 51 | 2 | 162 | 80 | 8 | 2 | 1 | 1 | 4 | 1 | 1 | 1 | 1 | 1 | 1 | 1 | 1 | 1 |
| 2268 | 51 | 2 | 162 | 65 | 8 | 2 | 1 | 1 | 4 | 4 | 1 | 1 | 1 | 1 | 1 | 1 | 1 | 1 |
| 2269 | 51 | 2 | 162 | 60 | 8 | 2 | 1 | 1 | 4 | 4 | 1 | 1 | 1 | 1 | 1 | 1 | 1 | 1 |
| 2270 | 51 | 2 | 162 | 59 | 8 | 2 | 1 | 1 | 4 | 4 | 1 | 1 | 1 | 1 | 1 | 1 | 1 | 1 |
| 2271 | 51 | 2 | 162 | 50 | 8 | 2 | 1 | 1 | 4 | 3 | 1 | 1 | 1 | 1 | 1 | 1 | 1 | 1 |
| 2272 | 51 | 2 | 163 | 67 | 8 | 2 | 1 | 1 | 3 | 3 | 1 | 1 | 1 | 1 | 2 | 1 | 1 | 1 |
| 2273 | 51 | 2 | 163 | 64 | 1 | 2 | 1 | 1 | 4 | 3 | 1 | 1 | 1 | 1 | 1 | 1 | 1 | 1 |
| 2274 | 51 | 2 | 163 | 62 | 4 | 1 | 1 | 1 | 4 | 4 | 1 | 1 | 1 | 1 | 2 | 1 | 1 | 1 |
| 2275 | 51 | 2 | 163 | 55 | 8 | 2 | 1 | 1 | 3 | 3 | 1 | 1 | 1 | 1 | 1 | 1 | 1 | 1 |
| 2276 | 51 | 2 | 163 | 53 | 8 | 2 | 1 | 1 | 4 | 4 | 1 | 1 | 1 | 1 | 1 | 1 | 1 | 1 |
| 2277 | 51 | 2 | 163.5 | 78 | 8 | 2 | 1 | 1 | 4 | 1 | 1 | 1 | 1 | 1 | 1 | 1 | 1 | 1 |
| 2278 | 51 | 2 | 164 | 64.5 | 8 | 2 | 1 | 1 | 4 | 4 | 1 | 1 | 1 | 1 | 1 | 1 | 1 | 1 |
| 2279 | 51 | 2 | 164 | 47 | 8 | 2 | 1 | 2 | 3 | 3 | 1 | 1 | 2 | 1 | 2 | 1 | 1 | 1 |
| 2280 | 51 | 2 | 165 | 66 | 4 | 2 | 1 | 1 | 4 | 4 | 1 | 1 | 1 | 1 | 1 | 1 | 1 | 1 |
| 2281 | 51 | 2 | 165 | 65 | 8 | 2 | 1 | 1 | 6 | 4 | 1 | 1 | 1 | 1 | 1 | 1 | 1 | 1 |
| 2282 | 51 | 2 | 166 | 52 | 8 | 1 | 1 | 1 | 4 | 2 | 1 | 1 | 1 | 1 | 1 | 1 | 1 | 1 |
| 2283 | 51 | 2 | 169 | 67 | 4 | 3 | 1 | 1 | 4 | 1 | 1 | 1 | 1 | 1 | 1 | 1 | 1 | 1 |
| 2284 | 51 | 2 | 170 | 71 | 8 | 2 | 1 | 1 | 4 | 4 | 1 | 1 | 1 | 1 | 1 | 1 | 1 | 1 |
| 2285 | 51 | 2 | 170 | 65 | 8 | 2 | 1 | 1 | 6 | 4 | 1 | 1 | 2 | 1 | 1 | 1 | 1 | 1 |
| 2286 | 51 | 1 | 160 | 68.8 | 4 | 2 | 1 | 1 | 3 | 4 | 1 | 1 | 1 | 1 | 1 | 1 | 1 | 1 |
| 2287 | 51 | 1 | 160 | 60 | 8 | 2 | 1 | 1 | 3 | 4 | 1 | 1 | 1 | 1 | 1 | 1 | 1 | 1 |
| 2288 | 51 | 1 | 161.5 | 63.5 | 3 | 2 | 1 | 1 | 4 | 4 | 1 | 1 | 1 | 1 | 1 | 1 | 1 | 1 |
| 2289 | 51 | 1 | 162 | 78 | 6 | 2 | 1 | 1 | 4 | 1 | 1 | 1 | 1 | 1 | 1 | 1 | 1 | 1 |
| 2290 | 51 | 1 | 163 | 67 | 8 | 2 | 1 | 3 | 3 | 3 | 1 | 1 | 1 | 1 | 1 | 1 | 3 | 2 |
| 2291 | 51 | 1 | 165 | 57.5 | 8 | 2 | 1 | 1 | 3 | 1 | 1 | 1 | 1 | 1 | 1 | 1 | 3 | 1 |
| 2292 | 51 | 1 | 166 | 72 | 8 | 2 | 1 | 1 | 4 | 1 | 2 | 1 | 2 | 1 | 1 | 1 | 1 | 1 |
| 2293 | 51 | 1 | 166 | 68.5 | 8 | 3 | 1 | 2 | 3 | 3 | 1 | 1 | 1 | 2 | 1 | 1 | 3 | 1 |
| 2294 | 51 | 1 | 167 | 59 | 8 | 2 | 1 | 2 | 4 | 4 | 1 | 1 | 2 | 1 | 1 | 1 | 3 | 1 |
| 2295 | 51 | 1 | 167 | 56 | 8 | 2 | 1 | 1 | 3 | 4 | 1 | 1 | 1 | 1 | 1 | 1 | 1 | 1 |
| 2296 | 51 | 1 | 167.5 | 63.5 | 2 | 2 | 1 | 2 | 5 | 2 | 1 | 1 | 1 | 1 | 1 | 1 | 1 | 1 |
| 2297 | 51 | 1 | 168 | 60 | 8 | 4 | 1 | 1 | 3 | 1 | 1 | 1 | 1 | 1 | 1 | 1 | 3 | 2 |
| 2298 | 51 | 1 | 168 | 55 | 2 | 2 | 1 | 1 | 5 | 2 | 1 | 1 | 1 | 1 | 1 | 1 | 1 | 2 |
| 2299 | 51 | 1 | 169 | 76 | 2 | 2 | 1 | 3 | 4 | 1 | 1 | 1 | 2 | 1 | 2 | 1 | 2 | 1 |
| 2300 | 51 | 1 | 169 | 75 | 8 | 1 | 1 | 1 | 4 | 4 | 1 | 1 | 1 | 1 | 1 | 1 | 1 | 1 |
| 2301 | 51 | 1 | 169 | 73 | 4 | 2 | 1 | 1 | 3 | 4 | 1 | 1 | 1 | 2 | 2 | 2 | 3 | 2 |
| 2302 | 51 | 1 | 169 | 72 | 6 | 2 | 1 | 1 | 4 | 1 | 1 | 1 | 1 | 1 | 1 | 1 | 1 | 2 |
| 2303 | 51 | 1 | 170 | 82 | 8 | 2 | 1 | 1 | 4 | 3 | 1 | 1 | 1 | 1 | 1 | 1 | 1 | 1 |
| 2304 | 51 | 1 | 170 | 75 | 8 | 2 | 1 | 1 | 5 | 3 | 1 | 1 | 1 | 1 | 1 | 1 | 1 | 1 |
| 2305 | 51 | 1 | 170 | 74.2 | 8 | 2 | 1 | 1 | 4 | 2 | 1 | 1 | 2 | 1 | 1 | 1 | 1 | 1 |
| 2306 | 51 | 1 | 170 | 74 | 8 | 2 | 1 | 2 | 4 | 3 | 1 | 1 | 1 | 1 | 1 | 1 | 3 | 1 |
| 2307 | 51 | 1 | 170 | 70 | 5 | 2 | 1 | 1 | 3 | 1 | 1 | 1 | 1 | 1 | 1 | 1 | 3 | 2 |
| 2308 | 51 | 1 | 170 | 64 | 3 | 2 | 1 | 1 | 4 | 4 | 1 | 1 | 1 | 1 | 1 | 1 | 1 | 1 |
| 2309 | 51 | 1 | 172 | 78 | 8 | 2 | 1 | 2 | 4 | 2 | 1 | 1 | 1 | 1 | 2 | 1 | 2 | 1 |
| 2310 | 51 | 1 | 173 | 80 | 3 | 2 | 1 | 2 | 4 | 2 | 1 | 1 | 1 | 1 | 1 | 1 | 2 | 1 |
| 2311 | 51 | 1 | 173 | 74 | 1 | 2 | 1 | 1 | 3 | 3 | 1 | 1 | 1 | 2 | 1 | 1 | 1 | 1 |
| 2312 | 51 | 1 | 173 | 52 | 8 | 2 | 1 | 1 | 4 | 4 | 1 | 1 | 1 | 1 | 1 | 1 | 1 | 1 |
| 2313 | 51 | 1 | 177 | 74 | 8 | 2 | 1 | 1 | 4 | 1 | 1 | 1 | 2 | 1 | 2 | 2 | 1 | 1 |
| 2314 | 52 | 2 | 149 | 54 | 2 | 2 | 1 | 1 | 4 | 1 | 1 | 1 | 1 | 1 | 1 | 1 | 1 | 1 |
| 2315 | 52 | 2 | 150 | 69 | 8 | 2 | 1 | 1 | 4 | 2 | 1 | 1 | 1 | 1 | 1 | 1 | 1 | 1 |
| 2316 | 52 | 2 | 150 | 69 | 4 | 2 | 1 | 1 | 3 | 4 | 1 | 1 | 1 | 1 | 1 | 1 | 1 | 1 |
| 2317 | 52 | 2 | 150 | 52.6 | 3 | 2 | 1 | 1 | 4 | 1 | 1 | 1 | 1 | 1 | 1 | 1 | 1 | 1 |
| 2318 | 52 | 2 | 150 | 50 | 8 | 2 | 1 | 1 | 4 | 3 | 1 | 1 | 2 | 1 | 1 | 1 | 1 | 1 |
| 2319 | 52 | 2 | 150 | 49 | 2 | 2 | 1 | 1 | 4 | 3 | 1 | 1 | 1 | 1 | 1 | 1 | 1 | 1 |
| 2320 | 52 | 2 | 150 | 44 | 8 | 2 | 1 | 1 | 4 | 3 | 1 | 1 | 1 | 1 | 1 | 1 | 1 | 1 |
| 2321 | 52 | 2 | 150 | 44 | 4 | 2 | 1 | 1 | 4 | 4 | 1 | 1 | 1 | 1 | 1 | 1 | 1 | 1 |
| 2322 | 52 | 2 | 150.5 | 45.5 | 3 | 2 | 1 | 1 | 4 | 2 | 1 | 1 | 1 | 2 | 1 | 1 | 1 | 2 |
| 2323 | 52 | 2 | 151 | 47 | 1 | 2 | 1 | 1 | 3 | 3 | 1 | 1 | 1 | 1 | 1 | 1 | 1 | 1 |
| 2324 | 52 | 2 | 151 | 43 | 8 | 2 | 1 | 1 | 5 | 1 | 1 | 1 | 1 | 2 | 1 | 1 | 1 | 1 |
| 2325 | 52 | 2 | 151.5 | 61.9 | 8 | 2 | 1 | 1 | 2 | 2 | 1 | 1 | 2 | 1 | 2 | 1 | 1 | 1 |
| 2326 | 52 | 2 | 152 | 75 | 8 | 2 | 1 | 1 | 6 | 4 | 1 | 1 | 1 | 1 | 1 | 1 | 1 | 1 |
| 2327 | 52 | 2 | 152 | 59 | 4 | 2 | 1 | 1 | 4 | 1 | 1 | 1 | 2 | 1 | 1 | 1 | 1 | 1 |
| 2328 | 52 | 2 | 152 | 54 | 6 | 2 | 1 | 1 | 3 | 2 | 1 | 1 | 1 | 1 | 1 | 1 | 1 | 1 |
| 2329 | 52 | 2 | 152 | 47.5 | 3 | 2 | 1 | 1 | 4 | 1 | 1 | 1 | 2 | 1 | 1 | 1 | 1 | 1 |
| 2330 | 52 | 2 | 153 | 43 | 4 | 2 | 1 | 1 | 4 | 4 | 1 | 1 | 1 | 1 | 1 | 1 | 1 | 1 |
| 2331 | 52 | 2 | 154 | 75 | 3 | 2 | 1 | 1 | 3 | 3 | 1 | 1 | 1 | 2 | 1 | 1 | 1 | 1 |
| 2332 | 52 | 2 | 154 | 60 | 8 | 2 | 1 | 1 | 4 | 1 | 1 | 1 | 2 | 1 | 1 | 1 | 1 | 1 |
| 2333 | 52 | 2 | 154 | 55 | 8 | 2 | 1 | 1 | 4 | 1 | 1 | 1 | 2 | 1 | 2 | 1 | 1 | 1 |
| 2334 | 52 | 2 | 154 | 50 | 6 | 4 | 1 | 1 | 3 | 1 | 1 | 1 | 1 | 1 | 2 | 1 | 1 | 1 |
| 2335 | 52 | 2 | 155 | 72.5 | 4 | 2 | 1 | 1 | 4 | 3 | 1 | 1 | 1 | 1 | 2 | 1 | 1 | 1 |
| 2336 | 52 | 2 | 155 | 58 | 4 | 2 | 1 | 1 | 4 | 1 | 1 | 1 | 1 | 1 | 1 | 1 | 1 | 1 |
| 2337 | 52 | 2 | 155 | 55 | 2 | 2 | 1 | 1 | 5 | 3 | 1 | 1 | 1 | 1 | 1 | 1 | 1 | 1 |
| 2338 | 52 | 2 | 155 | 52 | 8 | 2 | 1 | 1 | 4 | 2 | 1 | 1 | 1 | 1 | 1 | 1 | 1 | 1 |
| 2339 | 52 | 2 | 155 | 41.5 | 8 | 2 | 1 | 1 | 3 | 2 | 1 | 1 | 1 | 1 | 1 | 1 | 1 | 1 |
| 2340 | 52 | 2 | 155.5 | 67.9 | 8 | 2 | 1 | 1 | 4 | 1 | 1 | 1 | 1 | 1 | 1 | 1 | 1 | 1 |
| 2341 | 52 | 2 | 156 | 60 | 8 | 2 | 1 | 1 | 4 | 3 | 1 | 1 | 2 | 1 | 1 | 1 | 1 | 1 |
| 2342 | 52 | 2 | 156 | 60 | 8 | 2 | 1 | 1 | 4 | 3 | 1 | 1 | 1 | 1 | 1 | 1 | 1 | 1 |
| 2343 | 52 | 2 | 156 | 55 | 8 | 2 | 1 | 1 | 3 | 2 | 3 | 1 | 1 | 1 | 1 | 1 | 1 | 1 |
| 2344 | 52 | 2 | 156 | 52.5 | 1 | 2 | 1 | 1 | 1 | 4 | 1 | 1 | 1 | 1 | 1 | 1 | 2 | 1 |
| 2345 | 52 | 2 | 156 | 52 | 4 | 2 | 1 | 1 | 4 | 1 | 1 | 1 | 1 | 1 | 1 | 1 | 1 | 1 |
| 2346 | 52 | 2 | 156 | 50 | 4 | 2 | 2 | 2 | 4 | 2 | 1 | 1 | 1 | 1 | 2 | 1 | 1 | 1 |
| 2347 | 52 | 2 | 156 | 49 | 8 | 2 | 1 | 1 | 4 | 4 | 1 | 1 | 2 | 1 | 1 | 1 | 1 | 1 |
| 2348 | 52 | 2 | 156 | 47.5 | 8 | 2 | 1 | 1 | 4 | 1 | 1 | 1 | 1 | 1 | 1 | 1 | 1 | 1 |
| 2349 | 52 | 2 | 156 | 43 | 8 | 2 | 2 | 1 | 4 | 3 | 1 | 1 | 1 | 1 | 1 | 1 | 1 | 1 |
| 2350 | 52 | 2 | 157 | 52 | 8 | 2 | 1 | 1 | 3 | 1 | 1 | 1 | 1 | 1 | 1 | 1 | 1 | 1 |
| 2351 | 52 | 2 | 157 | 50 | 8 | 2 | 1 | 1 | 3 | 3 | 1 | 1 | 1 | 1 | 1 | 1 | 1 | 1 |
| 2352 | 52 | 2 | 158 | 66 | 8 | 3 | 1 | 1 | 4 | 1 | 1 | 1 | 1 | 1 | 1 | 1 | 1 | 1 |
| 2353 | 52 | 2 | 158 | 65 | 8 | 2 | 1 | 1 | 4 | 1 | 1 | 1 | 1 | 1 | 1 | 1 | 1 | 1 |
| 2354 | 52 | 2 | 158 | 63 | 8 | 2 | 1 | 1 | 4 | 3 | 1 | 1 | 2 | 1 | 1 | 1 | 1 | 1 |
| 2355 | 52 | 2 | 158 | 61.6 | 4 | 2 | 1 | 1 | 4 | 4 | 1 | 1 | 1 | 1 | 1 | 1 | 1 | 1 |
| 2356 | 52 | 2 | 158 | 57 | 8 | 2 | 1 | 1 | 4 | 3 | 1 | 1 | 1 | 1 | 1 | 1 | 1 | 1 |
| 2357 | 52 | 2 | 158 | 54 | 8 | 2 | 1 | 1 | 4 | 2 | 1 | 1 | 1 | 1 | 1 | 1 | 1 | 1 |
| 2358 | 52 | 2 | 158 | 51 | 8 | 2 | 1 | 1 | 4 | 4 | 1 | 1 | 1 | 1 | 1 | 1 | 1 | 1 |
| 2359 | 52 | 2 | 159 | 85 | 8 | 1 | 1 | 1 | 2 | 1 | 1 | 1 | 1 | 1 | 1 | 1 | 1 | 1 |
| 2360 | 52 | 2 | 159 | 62 | 8 | 2 | 1 | 1 | 4 | 3 | 1 | 1 | 1 | 1 | 2 | 1 | 1 | 1 |
| 2361 | 52 | 2 | 159 | 60 | 8 | 2 | 1 | 1 | 4 | 4 | 1 | 1 | 1 | 1 | 1 | 1 | 1 | 1 |
| 2362 | 52 | 2 | 159 | 60 | 8 | 2 | 1 | 1 | 4 | 3 | 1 | 1 | 1 | 1 | 1 | 1 | 1 | 1 |
| 2363 | 52 | 2 | 159 | 58 | 8 | 2 | 1 | 1 | 4 | 1 | 1 | 1 | 1 | 1 | 1 | 1 | 1 | 1 |
| 2364 | 52 | 2 | 159 | 56 | 3 | 2 | 1 | 1 | 4 | 3 | 1 | 1 | 2 | 1 | 1 | 1 | 1 | 1 |
| 2365 | 52 | 2 | 159 | 55 | 8 | 2 | 1 | 1 | 4 | 4 | 1 | 1 | 1 | 1 | 1 | 1 | 1 | 1 |
| 2366 | 52 | 2 | 160 | 84 | 8 | 2 | 1 | 1 | 4 | 1 | 1 | 1 | 1 | 1 | 2 | 1 | 1 | 2 |
| 2367 | 52 | 2 | 160 | 70 | 8 | 2 | 1 | 1 | 4 | 4 | 1 | 1 | 1 | 1 | 1 | 1 | 1 | 2 |
| 2368 | 52 | 2 | 160 | 70 | 8 | 2 | 1 | 1 | 3 | 4 | 1 | 1 | 1 | 1 | 1 | 1 | 1 | 1 |
| 2369 | 52 | 2 | 160 | 68 | 8 | 2 | 1 | 1 | 3 | 4 | 1 | 1 | 1 | 1 | 1 | 1 | 1 | 1 |
| 2370 | 52 | 2 | 160 | 65 | 8 | 3 | 1 | 1 | 4 | 4 | 1 | 1 | 1 | 1 | 1 | 1 | 1 | 1 |
| 2371 | 52 | 2 | 160 | 65 | 8 | 2 | 1 | 1 | 4 | 3 | 1 | 1 | 2 | 1 | 1 | 1 | 1 | 2 |
| 2372 | 52 | 2 | 160 | 64 | 4 | 4 | 1 | 1 | 4 | 4 | 1 | 1 | 1 | 1 | 2 | 1 | 1 | 1 |
| 2373 | 52 | 2 | 160 | 60.5 | 6 | 2 | 1 | 1 | 3 | 2 | 1 | 1 | 2 | 1 | 1 | 1 | 1 | 1 |
| 2374 | 52 | 2 | 160 | 60 | 8 | 2 | 1 | 1 | 3 | 1 | 1 | 1 | 1 | 1 | 1 | 1 | 1 | 1 |
| 2375 | 52 | 2 | 160 | 54 | 8 | 2 | 1 | 1 | 4 | 2 | 1 | 1 | 1 | 1 | 2 | 1 | 1 | 1 |
| 2376 | 52 | 2 | 160 | 51 | 8 | 2 | 2 | 1 | 4 | 1 | 1 | 1 | 2 | 1 | 2 | 1 | 1 | 1 |
| 2377 | 52 | 2 | 160 | 50 | 4 | 2 | 1 | 1 | 4 | 4 | 1 | 1 | 1 | 1 | 1 | 1 | 1 | 1 |
| 2378 | 52 | 2 | 160 | 49 | 8 | 1 | 1 | 1 | 4 | 4 | 1 | 1 | 1 | 1 | 2 | 1 | 1 | 1 |
| 2379 | 52 | 2 | 162 | 73.5 | 8 | 2 | 1 | 1 | 4 | 1 | 2 | 1 | 2 | 1 | 1 | 1 | 1 | 1 |
| 2380 | 52 | 2 | 162 | 67 | 8 | 2 | 1 | 1 | 4 | 3 | 1 | 1 | 2 | 1 | 2 | 1 | 1 | 1 |
| 2381 | 52 | 2 | 162 | 65 | 6 | 2 | 1 | 1 | 4 | 1 | 1 | 1 | 2 | 1 | 1 | 1 | 1 | 1 |
| 2382 | 52 | 2 | 162 | 59 | 8 | 2 | 2 | 1 | 4 | 4 | 1 | 1 | 1 | 2 | 1 | 1 | 1 | 2 |
| 2383 | 52 | 2 | 162 | 51 | 8 | 2 | 1 | 1 | 4 | 2 | 1 | 1 | 2 | 1 | 1 | 1 | 1 | 1 |
| 2384 | 52 | 2 | 163 | 63 | 8 | 1 | 1 | 1 | 4 | 2 | 1 | 1 | 2 | 1 | 1 | 1 | 1 | 1 |
| 2385 | 52 | 2 | 163 | 60 | 8 | 2 | 1 | 1 | 4 | 3 | 1 | 1 | 1 | 1 | 1 | 1 | 1 | 1 |
| 2386 | 52 | 2 | 163 | 57.5 | 1 | 2 | 1 | 1 | 4 | 1 | 1 | 1 | 1 | 1 | 1 | 1 | 1 | 1 |
| 2387 | 52 | 2 | 163 | 45 | 4 | 2 | 1 | 1 | 3 | 1 | 1 | 1 | 1 | 1 | 1 | 1 | 1 | 1 |
| 2388 | 52 | 2 | 164 | 63.5 | 4 | 4 | 1 | 1 | 4 | 1 | 1 | 1 | 1 | 1 | 1 | 1 | 1 | 1 |
| 2389 | 52 | 2 | 164 | 60 | 8 | 2 | 1 | 1 | 3 | 3 | 1 | 1 | 1 | 1 | 1 | 1 | 1 | 2 |
| 2390 | 52 | 2 | 164 | 57 | 8 | 2 | 1 | 1 | 4 | 3 | 1 | 1 | 1 | 1 | 1 | 1 | 1 | 1 |
| 2391 | 52 | 2 | 165 | 60.5 | 8 | 2 | 1 | 1 | 4 | 4 | 1 | 1 | 1 | 1 | 1 | 1 | 1 | 1 |
| 2392 | 52 | 2 | 165 | 53 | 4 | 2 | 1 | 1 | 4 | 1 | 1 | 1 | 1 | 1 | 1 | 1 | 1 | 1 |
| 2393 | 52 | 2 | 166 | 61 | 8 | 2 | 1 | 1 | 3 | 2 | 1 | 1 | 1 | 1 | 2 | 1 | 1 | 1 |
| 2394 | 52 | 2 | 168 | 78 | 8 | 2 | 1 | 1 | 4 | 3 | 1 | 1 | 1 | 1 | 1 | 1 | 1 | 1 |
| 2395 | 52 | 2 | 169 | 55 | 1 | 4 | 1 | 2 | 4 | 4 | 1 | 1 | 1 | 1 | 1 | 1 | 1 | 1 |
| 2396 | 52 | 1 | 157 | 64 | 8 | 2 | 1 | 1 | 3 | 3 | 1 | 1 | 1 | 1 | 1 | 1 | 1 | 1 |
| 2397 | 52 | 1 | 157.5 | 70.4 | 8 | 2 | 1 | 1 | 4 | 4 | 1 | 1 | 1 | 1 | 1 | 1 | 1 | 1 |
| 2398 | 52 | 1 | 158 | 73.5 | 4 | 2 | 1 | 1 | 5 | 3 | 1 | 1 | 2 | 1 | 1 | 1 | 1 | 1 |
| 2399 | 52 | 1 | 159 | 50 | 8 | 1 | 1 | 1 | 2 | 4 | 1 | 1 | 1 | 1 | 2 | 1 | 3 | 1 |
| 2400 | 52 | 1 | 165 | 63 | 8 | 2 | 1 | 1 | 3 | 4 | 1 | 1 | 1 | 1 | 1 | 1 | 1 | 2 |
| 2401 | 52 | 1 | 167 | 73.5 | 8 | 2 | 1 | 1 | 4 | 3 | 1 | 1 | 1 | 1 | 1 | 1 | 1 | 1 |
| 2402 | 52 | 1 | 167.5 | 68 | 8 | 2 | 1 | 1 | 4 | 1 | 1 | 1 | 1 | 1 | 1 | 1 | 1 | 1 |
| 2403 | 52 | 1 | 167.6 | 76 | 8 | 2 | 1 | 1 | 5 | 2 | 1 | 1 | 1 | 2 | 1 | 1 | 3 | 2 |
| 2404 | 52 | 1 | 168 | 67 | 1 | 2 | 1 | 1 | 4 | 4 | 1 | 1 | 1 | 1 | 1 | 1 | 1 | 1 |
| 2405 | 52 | 1 | 168 | 59 | 8 | 2 | 1 | 1 | 4 | 2 | 1 | 1 | 1 | 2 | 1 | 1 | 3 | 1 |
| 2406 | 52 | 1 | 170 | 70 | 8 | 2 | 1 | 1 | 5 | 2 | 1 | 1 | 1 | 1 | 1 | 1 | 3 | 2 |
| 2407 | 52 | 1 | 171 | 72.3 | 8 | 2 | 2 | 2 | 4 | 2 | 1 | 1 | 1 | 1 | 1 | 1 | 3 | 1 |
| 2408 | 52 | 1 | 171 | 71.5 | 1 | 2 | 1 | 1 | 5 | 1 | 1 | 1 | 2 | 1 | 1 | 1 | 1 | 1 |
| 2409 | 52 | 1 | 171 | 68 | 8 | 2 | 1 | 4 | 5 | 1 | 1 | 1 | 1 | 1 | 2 | 2 | 2 | 1 |
| 2410 | 52 | 1 | 171 | 61 | 8 | 2 | 1 | 1 | 5 | 1 | 1 | 1 | 1 | 1 | 1 | 1 | 1 | 1 |
| 2411 | 52 | 1 | 176 | 55 | 8 | 2 | 1 | 1 | 5 | 2 | 1 | 1 | 2 | 1 | 1 | 1 | 1 | 1 |
| 2412 | 52 | 1 | 177 | 74 | 8 | 2 | 2 | 1 | 3 | 4 | 1 | 1 | 1 | 1 | 1 | 1 | 1 | 1 |
| 2413 | 53 | 2 | 63 | 76 | 8 | 2 | 1 | 1 | 4 | 1 | 1 | 1 | 1 | 1 | 1 | 1 | 1 | 1 |
| 2414 | 53 | 2 | 145 | 43 | 4 | 2 | 1 | 1 | 4 | 1 | 1 | 1 | 2 | 1 | 2 | 1 | 1 | 1 |
| 2415 | 53 | 2 | 145.5 | 48 | 8 | 4 | 1 | 1 | 4 | 2 | 1 | 1 | 1 | 1 | 1 | 1 | 1 | 1 |
| 2416 | 53 | 2 | 148 | 56 | 8 | 2 | 1 | 1 | 4 | 2 | 1 | 1 | 1 | 1 | 1 | 1 | 1 | 1 |
| 2417 | 53 | 2 | 148 | 47 | 8 | 2 | 1 | 1 | 4 | 2 | 1 | 1 | 2 | 1 | 2 | 1 | 1 | 1 |
| 2418 | 53 | 2 | 150 | 70 | 8 | 2 | 1 | 1 | 4 | 4 | 1 | 1 | 1 | 1 | 1 | 1 | 1 | 1 |
| 2419 | 53 | 2 | 150 | 52 | 4 | 2 | 1 | 1 | 4 | 3 | 1 | 1 | 1 | 1 | 1 | 1 | 1 | 1 |
| 2420 | 53 | 2 | 150 | 45 | 8 | 2 | 1 | 1 | 4 | 1 | 1 | 1 | 1 | 1 | 1 | 1 | 1 | 1 |
| 2421 | 53 | 2 | 150 | 40 | 8 | 2 | 1 | 1 | 6 | 4 | 1 | 1 | 2 | 1 | 1 | 1 | 1 | 1 |
| 2422 | 53 | 2 | 151 | 51.4 | 8 | 2 | 1 | 1 | 4 | 1 | 1 | 1 | 1 | 1 | 1 | 1 | 1 | 1 |
| 2423 | 53 | 2 | 151 | 48 | 8 | 2 | 1 | 1 | 4 | 3 | 1 | 1 | 1 | 1 | 1 | 1 | 1 | 1 |
| 2424 | 53 | 2 | 152 | 55 | 8 | 1 | 1 | 1 | 4 | 2 | 1 | 1 | 2 | 1 | 1 | 1 | 1 | 1 |
| 2425 | 53 | 2 | 152 | 45 | 8 | 2 | 1 | 1 | 4 | 4 | 1 | 1 | 1 | 1 | 1 | 1 | 1 | 1 |
| 2426 | 53 | 2 | 153 | 61.4 | 2 | 2 | 1 | 1 | 2 | 1 | 1 | 1 | 2 | 1 | 1 | 1 | 1 | 1 |
| 2427 | 53 | 2 | 153 | 58 | 8 | 2 | 1 | 1 | 4 | 2 | 1 | 1 | 1 | 1 | 2 | 1 | 1 | 1 |
| 2428 | 53 | 2 | 153 | 57 | 8 | 2 | 1 | 1 | 3 | 3 | 1 | 1 | 1 | 1 | 1 | 1 | 1 | 1 |
| 2429 | 53 | 2 | 153 | 54.4 | 8 | 2 | 1 | 1 | 4 | 1 | 1 | 1 | 1 | 1 | 1 | 1 | 1 | 1 |
| 2430 | 53 | 2 | 153 | 53 | 2 | 2 | 1 | 1 | 3 | 1 | 1 | 1 | 1 | 1 | 1 | 1 | 1 | 1 |
| 2431 | 53 | 2 | 153 | 50 | 8 | 2 | 1 | 1 | 3 | 4 | 1 | 1 | 1 | 1 | 1 | 1 | 1 | 1 |
| 2432 | 53 | 2 | 154 | 67.5 | 8 | 2 | 1 | 1 | 4 | 2 | 1 | 1 | 1 | 1 | 2 | 1 | 1 | 1 |
| 2433 | 53 | 2 | 154 | 67.5 | 8 | 2 | 1 | 1 | 3 | 3 | 1 | 1 | 1 | 1 | 1 | 1 | 1 | 1 |
| 2434 | 53 | 2 | 154 | 66 | 2 | 2 | 1 | 1 | 5 | 4 | 1 | 1 | 2 | 1 | 1 | 1 | 1 | 1 |
| 2435 | 53 | 2 | 154 | 62 | 8 | 2 | 1 | 1 | 4 | 1 | 1 | 1 | 1 | 1 | 1 | 1 | 1 | 1 |
| 2436 | 53 | 2 | 154 | 53 | 8 | 2 | 2 | 1 | 4 | 2 | 1 | 1 | 2 | 1 | 1 | 1 | 1 | 1 |
| 2437 | 53 | 2 | 154 | 52.5 | 6 | 2 | 1 | 1 | 4 | 1 | 1 | 1 | 2 | 1 | 1 | 1 | 1 | 1 |
| 2438 | 53 | 2 | 154 | 49 | 4 | 2 | 1 | 1 | 4 | 2 | 1 | 1 | 2 | 1 | 1 | 1 | 1 | 1 |
| 2439 | 53 | 2 | 154.5 | 54.2 | 6 | 2 | 1 | 1 | 5 | 1 | 1 | 1 | 1 | 1 | 1 | 1 | 1 | 1 |
| 2440 | 53 | 2 | 155 | 62 | 8 | 2 | 1 | 1 | 5 | 2 | 1 | 1 | 1 | 1 | 1 | 1 | 1 | 1 |
| 2441 | 53 | 2 | 155 | 58.4 | 8 | 2 | 1 | 1 | 3 | 4 | 1 | 1 | 1 | 1 | 1 | 1 | 1 | 1 |
| 2442 | 53 | 2 | 155 | 54 | 1 | 2 | 1 | 1 | 6 | 3 | 1 | 1 | 1 | 1 | 1 | 1 | 1 | 1 |
| 2443 | 53 | 2 | 155 | 45 | 6 | 2 | 1 | 1 | 4 | 1 | 1 | 1 | 2 | 1 | 1 | 1 | 1 | 1 |
| 2444 | 53 | 2 | 156 | 67 | 8 | 2 | 1 | 1 | 3 | 1 | 1 | 1 | 1 | 1 | 1 | 1 | 1 | 1 |
| 2445 | 53 | 2 | 156 | 64.5 | 4 | 2 | 1 | 1 | 3 | 4 | 1 | 1 | 1 | 1 | 1 | 1 | 1 | 2 |
| 2446 | 53 | 2 | 156 | 60 | 8 | 2 | 1 | 1 | 4 | 4 | 1 | 1 | 1 | 1 | 1 | 1 | 1 | 1 |
| 2447 | 53 | 2 | 156 | 56 | 8 | 2 | 1 | 1 | 4 | 4 | 1 | 1 | 1 | 1 | 1 | 1 | 1 | 1 |
| 2448 | 53 | 2 | 156 | 55 | 8 | 2 | 1 | 1 | 4 | 2 | 1 | 1 | 1 | 1 | 2 | 1 | 1 | 1 |
| 2449 | 53 | 2 | 156 | 55 | 8 | 2 | 1 | 1 | 3 | 3 | 1 | 1 | 1 | 1 | 2 | 1 | 1 | 1 |
| 2450 | 53 | 2 | 156 | 46 | 8 | 2 | 1 | 1 | 4 | 1 | 1 | 1 | 1 | 1 | 1 | 1 | 1 | 1 |
| 2451 | 53 | 2 | 156.5 | 46 | 8 | 2 | 1 | 1 | 4 | 1 | 1 | 1 | 1 | 1 | 1 | 1 | 1 | 1 |
| 2452 | 53 | 2 | 157 | 57 | 8 | 2 | 1 | 1 | 3 | 2 | 1 | 1 | 1 | 1 | 1 | 1 | 1 | 1 |
| 2453 | 53 | 2 | 157 | 56.5 | 8 | 2 | 1 | 1 | 1 | 2 | 1 | 1 | 1 | 1 | 1 | 1 | 1 | 2 |
| 2454 | 53 | 2 | 158 | 71 | 8 | 2 | 1 | 1 | 3 | 4 | 1 | 1 | 1 | 1 | 1 | 1 | 1 | 1 |
| 2455 | 53 | 2 | 158 | 65.5 | 8 | 2 | 1 | 1 | 4 | 1 | 1 | 1 | 1 | 1 | 1 | 1 | 1 | 1 |
| 2456 | 53 | 2 | 158 | 65 | 8 | 2 | 1 | 1 | 4 | 2 | 1 | 1 | 1 | 1 | 1 | 1 | 1 | 1 |
| 2457 | 53 | 2 | 158 | 64 | 8 | 2 | 1 | 1 | 4 | 4 | 1 | 1 | 1 | 1 | 1 | 1 | 1 | 1 |
| 2458 | 53 | 2 | 158 | 60 | 8 | 2 | 1 | 1 | 4 | 1 | 1 | 1 | 1 | 1 | 1 | 1 | 1 | 1 |
| 2459 | 53 | 2 | 158 | 59.5 | 8 | 2 | 1 | 1 | 4 | 4 | 1 | 1 | 1 | 1 | 1 | 1 | 1 | 1 |
| 2460 | 53 | 2 | 158 | 56 | 8 | 2 | 1 | 1 | 4 | 2 | 1 | 1 | 1 | 1 | 1 | 1 | 1 | 1 |
| 2461 | 53 | 2 | 158 | 53 | 8 | 2 | 1 | 1 | 3 | 1 | 2 | 1 | 1 | 2 | 1 | 1 | 1 | 2 |
| 2462 | 53 | 2 | 158 | 45 | 8 | 2 | 1 | 1 | 2 | 1 | 1 | 1 | 1 | 1 | 1 | 1 | 1 | 1 |
| 2463 | 53 | 2 | 159 | 58 | 8 | 2 | 1 | 1 | 5 | 4 | 1 | 1 | 1 | 1 | 1 | 1 | 1 | 1 |
| 2464 | 53 | 2 | 159 | 54 | 4 | 2 | 1 | 1 | 4 | 2 | 1 | 1 | 1 | 1 | 2 | 2 | 1 | 1 |
| 2465 | 53 | 2 | 159 | 52 | 8 | 2 | 1 | 2 | 6 | 1 | 1 | 1 | 1 | 1 | 1 | 1 | 1 | 1 |
| 2466 | 53 | 2 | 159 | 52 | 8 | 2 | 1 | 1 | 4 | 3 | 1 | 1 | 1 | 1 | 1 | 1 | 1 | 1 |
| 2467 | 53 | 2 | 160 | 108 | 2 | 2 | 1 | 1 | 6 | 4 | 1 | 1 | 1 | 1 | 2 | 1 | 1 | 1 |
| 2468 | 53 | 2 | 160 | 67 | 6 | 2 | 1 | 1 | 4 | 2 | 1 | 1 | 1 | 1 | 1 | 1 | 1 | 1 |
| 2469 | 53 | 2 | 160 | 59 | 1 | 2 | 1 | 1 | 4 | 1 | 1 | 1 | 1 | 1 | 1 | 1 | 1 | 1 |
| 2470 | 53 | 2 | 160 | 55 | 1 | 2 | 1 | 1 | 5 | 1 | 1 | 1 | 1 | 2 | 1 | 1 | 1 | 1 |
| 2471 | 53 | 2 | 160 | 50.5 | 8 | 2 | 1 | 1 | 3 | 1 | 1 | 1 | 1 | 1 | 1 | 1 | 1 | 1 |
| 2472 | 53 | 2 | 160.5 | 67.5 | 8 | 2 | 1 | 1 | 3 | 4 | 1 | 1 | 1 | 1 | 1 | 1 | 1 | 1 |
| 2473 | 53 | 2 | 160.5 | 62.5 | 4 | 2 | 1 | 2 | 4 | 1 | 1 | 1 | 2 | 1 | 1 | 2 | 1 | 1 |
| 2474 | 53 | 2 | 161 | 65.5 | 8 | 2 | 1 | 1 | 4 | 1 | 1 | 1 | 1 | 1 | 2 | 1 | 1 | 1 |
| 2475 | 53 | 2 | 162 | 70 | 3 | 2 | 1 | 1 | 4 | 2 | 1 | 1 | 1 | 1 | 2 | 1 | 1 | 1 |
| 2476 | 53 | 2 | 162 | 54 | 8 | 2 | 2 | 1 | 4 | 2 | 1 | 1 | 2 | 1 | 2 | 1 | 1 | 1 |
| 2477 | 53 | 2 | 163 | 66 | 1 | 2 | 1 | 1 | 5 | 1 | 1 | 1 | 1 | 1 | 1 | 1 | 1 | 1 |
| 2478 | 53 | 2 | 163 | 50 | 4 | 2 | 1 | 1 | 4 | 2 | 1 | 1 | 1 | 2 | 1 | 1 | 1 | 2 |
| 2479 | 53 | 2 | 164 | 60 | 8 | 2 | 1 | 1 | 4 | 3 | 1 | 1 | 1 | 1 | 1 | 1 | 1 | 1 |
| 2480 | 53 | 2 | 164 | 55 | 8 | 1 | 1 | 1 | 3 | 1 | 1 | 1 | 1 | 1 | 1 | 1 | 1 | 1 |
| 2481 | 53 | 2 | 164 | 51 | 8 | 2 | 1 | 1 | 4 | 2 | 1 | 1 | 1 | 1 | 1 | 1 | 1 | 1 |
| 2482 | 53 | 2 | 165 | 90 | 8 | 2 | 1 | 1 | 4 | 2 | 1 | 1 | 1 | 1 | 1 | 1 | 1 | 1 |
| 2483 | 53 | 2 | 165 | 70 | 8 | 2 | 1 | 1 | 4 | 4 | 1 | 1 | 1 | 2 | 1 | 1 | 1 | 2 |
| 2484 | 53 | 2 | 165 | 58.5 | 8 | 2 | 1 | 1 | 4 | 1 | 1 | 1 | 1 | 1 | 1 | 1 | 1 | 1 |
| 2485 | 53 | 2 | 166 | 74 | 8 | 2 | 2 | 1 | 4 | 2 | 1 | 1 | 1 | 1 | 1 | 1 | 1 | 1 |
| 2486 | 53 | 2 | 168 | 60 | 8 | 2 | 1 | 1 | 4 | 1 | 1 | 1 | 1 | 1 | 1 | 1 | 1 | 1 |
| 2487 | 53 | 1 | 155 | 69 | 8 | 2 | 2 | 1 | 3 | 1 | 1 | 1 | 1 | 1 | 1 | 1 | 1 | 1 |
| 2488 | 53 | 1 | 162 | 58 | 8 | 2 | 1 | 1 | 4 | 4 | 1 | 1 | 1 | 1 | 1 | 1 | 1 | 1 |
| 2489 | 53 | 1 | 162.5 | 63.5 | 4 | 2 | 1 | 1 | 5 | 4 | 2 | 1 | 1 | 2 | 2 | 1 | 1 | 2 |
| 2490 | 53 | 1 | 164 | 78.8 | 8 | 1 | 1 | 2 | 4 | 1 | 1 | 1 | 1 | 1 | 1 | 1 | 1 | 1 |
| 2491 | 53 | 1 | 164 | 55 | 8 | 2 | 1 | 1 | 4 | 2 | 1 | 1 | 1 | 1 | 1 | 1 | 1 | 1 |
| 2492 | 53 | 1 | 165 | 81 | 8 | 2 | 1 | 1 | 5 | 1 | 1 | 1 | 1 | 1 | 1 | 1 | 3 | 1 |
| 2493 | 53 | 1 | 165 | 80 | 2 | 2 | 1 | 1 | 3 | 2 | 1 | 1 | 2 | 1 | 1 | 1 | 3 | 1 |
| 2494 | 53 | 1 | 165 | 64 | 8 | 4 | 1 | 1 | 3 | 2 | 1 | 1 | 1 | 1 | 1 | 1 | 1 | 1 |
| 2495 | 53 | 1 | 165.3 | 87.5 | 8 | 2 | 1 | 1 | 4 | 4 | 1 | 1 | 1 | 1 | 1 | 1 | 1 | 1 |
| 2496 | 53 | 1 | 166 | 68.5 | 8 | 2 | 1 | 2 | 5 | 2 | 1 | 1 | 2 | 1 | 1 | 1 | 1 | 1 |
| 2497 | 53 | 1 | 167 | 85 | 8 | 2 | 1 | 1 | 4 | 4 | 1 | 1 | 1 | 1 | 1 | 1 | 3 | 1 |
| 2498 | 53 | 1 | 167 | 76 | 8 | 2 | 1 | 1 | 4 | 2 | 1 | 1 | 1 | 1 | 1 | 1 | 2 | 1 |
| 2499 | 53 | 1 | 167 | 65 | 6 | 2 | 1 | 1 | 4 | 4 | 1 | 1 | 1 | 1 | 1 | 1 | 1 | 1 |
| 2500 | 53 | 1 | 167 | 60 | 5 | 2 | 1 | 1 | 4 | 3 | 1 | 1 | 1 | 1 | 1 | 1 | 2 | 1 |
| 2501 | 53 | 1 | 168 | 54 | 8 | 2 | 1 | 2 | 5 | 2 | 2 | 1 | 2 | 1 | 2 | 1 | 2 | 1 |
| 2502 | 53 | 1 | 170 | 72 | 8 | 1 | 1 | 1 | 4 | 1 | 1 | 1 | 1 | 1 | 1 | 1 | 1 | 1 |
| 2503 | 53 | 1 | 170 | 65 | 2 | 2 | 1 | 4 | 4 | 1 | 1 | 1 | 2 | 1 | 2 | 1 | 3 | 1 |
| 2504 | 53 | 1 | 170 | 61 | 4 | 2 | 1 | 2 | 4 | 3 | 1 | 1 | 1 | 1 | 1 | 1 | 3 | 1 |
| 2505 | 53 | 1 | 171 | 72 | 8 | 2 | 1 | 2 | 2 | 2 | 1 | 1 | 1 | 1 | 2 | 1 | 2 | 1 |
| 2506 | 53 | 1 | 172 | 65 | 8 | 2 | 1 | 1 | 3 | 4 | 1 | 1 | 1 | 1 | 1 | 1 | 1 | 1 |
| 2507 | 53 | 1 | 174 | 65 | 8 | 2 | 1 | 1 | 3 | 4 | 1 | 1 | 1 | 1 | 1 | 1 | 3 | 1 |
| 2508 | 53 | 1 | 176 | 61 | 8 | 2 | 1 | 1 | 5 | 2 | 1 | 1 | 1 | 1 | 1 | 1 | 1 | 1 |
| 2509 | 53 | 1 | 180 | 82 | 8 | 2 | 1 | 1 | 5 | 2 | 1 | 1 | 1 | 1 | 1 | 1 | 1 | 1 |
| 2510 | 54 | 2 | 82 | 64 | 8 | 2 | 1 | 1 | 4 | 2 | 1 | 1 | 1 | 1 | 1 | 1 | 1 | 1 |
| 2511 | 54 | 2 | 144 | 42 | 8 | 1 | 1 | 1 | 3 | 2 | 1 | 1 | 1 | 1 | 1 | 1 | 1 | 1 |
| 2512 | 54 | 2 | 145 | 45 | 6 | 2 | 1 | 1 | 4 | 1 | 1 | 1 | 1 | 1 | 1 | 1 | 1 | 1 |
| 2513 | 54 | 2 | 147 | 65 | 8 | 2 | 1 | 1 | 4 | 4 | 1 | 1 | 2 | 1 | 1 | 1 | 1 | 1 |
| 2514 | 54 | 2 | 149 | 60 | 8 | 2 | 1 | 1 | 2 | 3 | 1 | 1 | 1 | 1 | 1 | 1 | 1 | 1 |
| 2515 | 54 | 2 | 149 | 51 | 8 | 2 | 1 | 1 | 2 | 2 | 1 | 1 | 1 | 2 | 1 | 1 | 1 | 2 |
| 2516 | 54 | 2 | 150 | 65 | 8 | 1 | 1 | 1 | 4 | 3 | 1 | 1 | 2 | 1 | 1 | 1 | 1 | 1 |
| 2517 | 54 | 2 | 150 | 62 | 8 | 2 | 1 | 1 | 4 | 4 | 1 | 1 | 1 | 1 | 1 | 1 | 1 | 1 |
| 2518 | 54 | 2 | 150 | 52.5 | 8 | 2 | 2 | 1 | 3 | 1 | 1 | 1 | 1 | 1 | 1 | 1 | 1 | 1 |
| 2519 | 54 | 2 | 150 | 48 | 8 | 2 | 1 | 1 | 3 | 4 | 1 | 1 | 1 | 1 | 1 | 1 | 1 | 1 |
| 2520 | 54 | 2 | 150 | 45 | 1 | 2 | 1 | 1 | 3 | 2 | 1 | 1 | 1 | 2 | 2 | 1 | 1 | 2 |
| 2521 | 54 | 2 | 150 | 44 | 8 | 2 | 1 | 1 | 4 | 4 | 1 | 1 | 1 | 1 | 1 | 1 | 1 | 1 |
| 2522 | 54 | 2 | 150.5 | 59.5 | 6 | 2 | 2 | 1 | 4 | 1 | 1 | 1 | 1 | 1 | 1 | 1 | 1 | 1 |
| 2523 | 54 | 2 | 152 | 55 | 8 | 2 | 1 | 1 | 4 | 1 | 1 | 1 | 1 | 1 | 1 | 1 | 1 | 1 |
| 2524 | 54 | 2 | 152 | 53 | 8 | 2 | 1 | 1 | 4 | 2 | 1 | 1 | 2 | 1 | 1 | 1 | 1 | 2 |
| 2525 | 54 | 2 | 152 | 48 | 8 | 2 | 1 | 1 | 4 | 2 | 1 | 1 | 1 | 1 | 1 | 1 | 1 | 1 |
| 2526 | 54 | 2 | 153 | 70 | 8 | 2 | 1 | 1 | 2 | 4 | 1 | 1 | 1 | 1 | 1 | 1 | 1 | 1 |
| 2527 | 54 | 2 | 153 | 59 | 8 | 2 | 1 | 1 | 4 | 2 | 1 | 1 | 1 | 1 | 1 | 1 | 1 | 1 |
| 2528 | 54 | 2 | 153 | 50 | 8 | 2 | 1 | 1 | 5 | 1 | 1 | 1 | 1 | 1 | 1 | 1 | 2 | 1 |
| 2529 | 54 | 2 | 153 | 47 | 8 | 2 | 1 | 1 | 4 | 2 | 1 | 1 | 1 | 1 | 1 | 1 | 1 | 1 |
| 2530 | 54 | 2 | 153 | 46 | 2 | 2 | 1 | 1 | 4 | 3 | 1 | 1 | 1 | 1 | 1 | 1 | 1 | 1 |
| 2531 | 54 | 2 | 153 | 45 | 8 | 2 | 1 | 1 | 4 | 1 | 1 | 1 | 1 | 1 | 2 | 1 | 1 | 1 |
| 2532 | 54 | 2 | 153 | 44 | 1 | 1 | 1 | 1 | 4 | 2 | 1 | 1 | 1 | 1 | 1 | 1 | 1 | 1 |
| 2533 | 54 | 2 | 154 | 52 | 8 | 2 | 1 | 1 | 1 | 2 | 1 | 1 | 1 | 1 | 1 | 1 | 1 | 1 |
| 2534 | 54 | 2 | 155 | 62.5 | 8 | 2 | 1 | 1 | 4 | 3 | 1 | 1 | 1 | 1 | 1 | 1 | 1 | 1 |
| 2535 | 54 | 2 | 155 | 60 | 8 | 2 | 1 | 1 | 4 | 3 | 1 | 1 | 1 | 1 | 2 | 1 | 1 | 1 |
| 2536 | 54 | 2 | 155 | 59 | 8 | 2 | 1 | 1 | 3 | 1 | 1 | 1 | 1 | 1 | 1 | 1 | 1 | 1 |
| 2537 | 54 | 2 | 155 | 55 | 8 | 2 | 1 | 1 | 2 | 1 | 1 | 1 | 2 | 1 | 1 | 1 | 1 | 1 |
| 2538 | 54 | 2 | 155 | 54 | 4 | 2 | 1 | 1 | 4 | 2 | 1 | 1 | 1 | 1 | 1 | 1 | 1 | 1 |
| 2539 | 54 | 2 | 155 | 50 | 8 | 2 | 1 | 1 | 4 | 2 | 1 | 1 | 1 | 1 | 1 | 1 | 1 | 1 |
| 2540 | 54 | 2 | 155 | 50 | 8 | 2 | 1 | 1 | 3 | 4 | 1 | 1 | 1 | 1 | 1 | 1 | 1 | 1 |
| 2541 | 54 | 2 | 155 | 48 | 4 | 2 | 1 | 1 | 5 | 4 | 1 | 1 | 1 | 1 | 1 | 1 | 1 | 1 |
| 2542 | 54 | 2 | 155 | 46.5 | 8 | 2 | 1 | 1 | 4 | 1 | 1 | 1 | 1 | 1 | 1 | 1 | 1 | 1 |
| 2543 | 54 | 2 | 156 | 66 | 8 | 2 | 2 | 1 | 2 | 3 | 1 | 1 | 1 | 1 | 1 | 1 | 1 | 1 |
| 2544 | 54 | 2 | 156 | 57 | 3 | 2 | 1 | 1 | 4 | 2 | 1 | 1 | 1 | 1 | 1 | 1 | 1 | 1 |
| 2545 | 54 | 2 | 156 | 55 | 8 | 2 | 1 | 1 | 4 | 1 | 1 | 1 | 1 | 1 | 1 | 1 | 1 | 1 |
| 2546 | 54 | 2 | 156 | 55 | 8 | 2 | 1 | 1 | 4 | 1 | 1 | 1 | 1 | 1 | 1 | 1 | 1 | 1 |
| 2547 | 54 | 2 | 156 | 55 | 8 | 2 | 1 | 1 | 4 | 2 | 1 | 1 | 1 | 1 | 1 | 1 | 1 | 1 |
| 2548 | 54 | 2 | 156 | 55 | 8 | 2 | 1 | 1 | 4 | 2 | 1 | 1 | 1 | 1 | 1 | 1 | 1 | 1 |
| 2549 | 54 | 2 | 156 | 53 | 8 | 2 | 1 | 1 | 3 | 3 | 1 | 1 | 1 | 1 | 1 | 1 | 1 | 1 |
| 2550 | 54 | 2 | 156 | 53 | 4 | 2 | 1 | 1 | 4 | 1 | 1 | 1 | 1 | 1 | 1 | 1 | 1 | 1 |
| 2551 | 54 | 2 | 156 | 52 | 3 | 2 | 1 | 1 | 3 | 1 | 1 | 1 | 1 | 1 | 1 | 1 | 1 | 1 |
| 2552 | 54 | 2 | 156 | 48 | 8 | 2 | 1 | 1 | 2 | 3 | 1 | 1 | 1 | 1 | 1 | 1 | 1 | 1 |
| 2553 | 54 | 2 | 157 | 65 | 2 | 2 | 1 | 1 | 4 | 4 | 3 | 1 | 1 | 2 | 1 | 1 | 1 | 2 |
| 2554 | 54 | 2 | 157 | 52 | 8 | 2 | 1 | 1 | 3 | 3 | 1 | 1 | 1 | 2 | 1 | 1 | 1 | 2 |
| 2555 | 54 | 2 | 157 | 49 | 8 | 2 | 1 | 1 | 2 | 1 | 1 | 1 | 1 | 1 | 1 | 1 | 1 | 1 |
| 2556 | 54 | 2 | 157 | 38 | 8 | 2 | 1 | 1 | 4 | 2 | 1 | 1 | 1 | 1 | 1 | 1 | 1 | 1 |
| 2557 | 54 | 2 | 157.5 | 71.2 | 8 | 2 | 1 | 1 | 4 | 1 | 1 | 1 | 1 | 1 | 1 | 1 | 1 | 1 |
| 2558 | 54 | 2 | 157.5 | 54 | 8 | 2 | 1 | 1 | 4 | 1 | 1 | 1 | 1 | 1 | 2 | 1 | 1 | 1 |
| 2559 | 54 | 2 | 158 | 70.5 | 8 | 2 | 1 | 1 | 4 | 2 | 2 | 1 | 1 | 1 | 1 | 1 | 1 | 1 |
| 2560 | 54 | 2 | 158 | 68.8 | 8 | 2 | 1 | 1 | 4 | 1 | 1 | 1 | 2 | 1 | 1 | 1 | 1 | 1 |
| 2561 | 54 | 2 | 158 | 67 | 8 | 2 | 1 | 1 | 6 | 4 | 2 | 1 | 1 | 1 | 1 | 1 | 1 | 1 |
| 2562 | 54 | 2 | 158 | 63 | 8 | 3 | 1 | 1 | 4 | 1 | 1 | 1 | 1 | 1 | 2 | 1 | 1 | 1 |
| 2563 | 54 | 2 | 158 | 62 | 1 | 2 | 1 | 1 | 5 | 4 | 1 | 1 | 1 | 1 | 1 | 1 | 1 | 1 |
| 2564 | 54 | 2 | 158 | 60 | 8 | 4 | 1 | 1 | 4 | 1 | 1 | 1 | 1 | 1 | 2 | 1 | 1 | 1 |
| 2565 | 54 | 2 | 158 | 60 | 8 | 2 | 1 | 1 | 4 | 4 | 1 | 1 | 1 | 1 | 1 | 1 | 1 | 1 |
| 2566 | 54 | 2 | 158 | 54.5 | 8 | 2 | 1 | 1 | 4 | 2 | 1 | 1 | 1 | 1 | 2 | 1 | 1 | 1 |
| 2567 | 54 | 2 | 158 | 54.5 | 8 | 2 | 1 | 1 | 3 | 2 | 1 | 1 | 1 | 1 | 1 | 1 | 1 | 1 |
| 2568 | 54 | 2 | 158 | 52 | 8 | 2 | 1 | 1 | 4 | 3 | 1 | 1 | 1 | 1 | 1 | 1 | 1 | 1 |
| 2569 | 54 | 2 | 158 | 52 | 6 | 2 | 1 | 1 | 4 | 4 | 1 | 1 | 1 | 1 | 1 | 1 | 1 | 1 |
| 2570 | 54 | 2 | 158 | 50 | 8 | 2 | 1 | 1 | 6 | 2 | 1 | 1 | 1 | 1 | 1 | 1 | 1 | 1 |
| 2571 | 54 | 2 | 158.5 | 69 | 4 | 2 | 1 | 1 | 4 | 2 | 1 | 1 | 1 | 1 | 2 | 1 | 1 | 1 |
| 2572 | 54 | 2 | 158.5 | 51 | 4 | 2 | 1 | 1 | 4 | 1 | 1 | 1 | 1 | 1 | 1 | 1 | 1 | 1 |
| 2573 | 54 | 2 | 159 | 60 | 8 | 2 | 1 | 1 | 4 | 4 | 1 | 1 | 2 | 1 | 1 | 1 | 1 | 1 |
| 2574 | 54 | 2 | 159 | 58 | 8 | 2 | 1 | 1 | 4 | 3 | 1 | 1 | 2 | 1 | 1 | 1 | 1 | 1 |
| 2575 | 54 | 2 | 159 | 50 | 3 | 3 | 1 | 1 | 4 | 3 | 1 | 1 | 1 | 1 | 1 | 1 | 1 | 1 |
| 2576 | 54 | 2 | 160 | 100 | 8 | 2 | 1 | 1 | 4 | 3 | 1 | 1 | 1 | 1 | 2 | 1 | 1 | 1 |
| 2577 | 54 | 2 | 160 | 80 | 8 | 2 | 1 | 1 | 4 | 3 | 1 | 1 | 1 | 1 | 1 | 1 | 1 | 1 |
| 2578 | 54 | 2 | 160 | 67 | 6 | 2 | 1 | 1 | 4 | 1 | 1 | 1 | 2 | 1 | 2 | 1 | 1 | 1 |
| 2579 | 54 | 2 | 160 | 65 | 8 | 2 | 1 | 1 | 3 | 1 | 1 | 1 | 1 | 1 | 1 | 1 | 1 | 1 |
| 2580 | 54 | 2 | 160 | 62 | 8 | 2 | 1 | 1 | 4 | 4 | 1 | 1 | 1 | 1 | 1 | 1 | 1 | 1 |
| 2581 | 54 | 2 | 160 | 60 | 2 | 2 | 1 | 2 | 4 | 3 | 1 | 1 | 1 | 1 | 1 | 1 | 1 | 1 |
| 2582 | 54 | 2 | 160 | 58 | 8 | 2 | 1 | 1 | 4 | 4 | 1 | 1 | 1 | 1 | 1 | 1 | 1 | 2 |
| 2583 | 54 | 2 | 160 | 55 | 8 | 2 | 1 | 1 | 4 | 1 | 1 | 1 | 1 | 1 | 1 | 1 | 1 | 1 |
| 2584 | 54 | 2 | 160 | 52 | 8 | 2 | 1 | 1 | 4 | 3 | 1 | 1 | 1 | 1 | 1 | 1 | 1 | 1 |
| 2585 | 54 | 2 | 160 | 52 | 8 | 1 | 1 | 1 | 3 | 3 | 1 | 1 | 1 | 2 | 1 | 1 | 1 | 1 |
| 2586 | 54 | 2 | 160 | 52 | 6 | 2 | 1 | 2 | 4 | 1 | 1 | 1 | 1 | 1 | 1 | 1 | 1 | 1 |
| 2587 | 54 | 2 | 160 | 52 | 4 | 2 | 1 | 1 | 4 | 3 | 1 | 1 | 1 | 1 | 2 | 2 | 1 | 1 |
| 2588 | 54 | 2 | 160 | 46.5 | 8 | 4 | 1 | 1 | 4 | 1 | 1 | 1 | 2 | 1 | 2 | 1 | 1 | 1 |
| 2589 | 54 | 2 | 160.3 | 53.4 | 1 | 1 | 1 | 1 | 4 | 1 | 1 | 1 | 1 | 2 | 2 | 1 | 1 | 2 |
| 2590 | 54 | 2 | 161 | 68 | 8 | 2 | 1 | 2 | 4 | 1 | 1 | 1 | 1 | 2 | 1 | 1 | 2 | 2 |
| 2591 | 54 | 2 | 161 | 55 | 4 | 2 | 1 | 1 | 6 | 2 | 1 | 1 | 1 | 1 | 1 | 1 | 1 | 1 |
| 2592 | 54 | 2 | 161.5 | 63.1 | 8 | 2 | 1 | 1 | 3 | 1 | 1 | 1 | 1 | 1 | 1 | 1 | 1 | 1 |
| 2593 | 54 | 2 | 162 | 77.5 | 8 | 2 | 1 | 1 | 4 | 2 | 1 | 1 | 1 | 1 | 2 | 1 | 1 | 1 |
| 2594 | 54 | 2 | 162 | 65 | 8 | 2 | 1 | 1 | 4 | 3 | 1 | 1 | 1 | 1 | 1 | 1 | 1 | 1 |
| 2595 | 54 | 2 | 162 | 61 | 3 | 2 | 1 | 1 | 4 | 2 | 1 | 1 | 1 | 1 | 1 | 1 | 1 | 1 |
| 2596 | 54 | 2 | 162 | 55 | 3 | 2 | 1 | 1 | 4 | 1 | 1 | 1 | 2 | 1 | 1 | 1 | 1 | 1 |
| 2597 | 54 | 2 | 162 | 41 | 1 | 2 | 1 | 1 | 4 | 4 | 1 | 1 | 1 | 1 | 1 | 1 | 1 | 1 |
| 2598 | 54 | 2 | 163 | 70 | 8 | 4 | 1 | 1 | 4 | 2 | 1 | 1 | 1 | 1 | 1 | 1 | 1 | 1 |
| 2599 | 54 | 2 | 163 | 64.5 | 8 | 2 | 1 | 1 | 5 | 1 | 1 | 1 | 1 | 1 | 2 | 2 | 1 | 1 |
| 2600 | 54 | 2 | 163.5 | 70.8 | 8 | 4 | 1 | 1 | 4 | 3 | 1 | 1 | 1 | 1 | 1 | 1 | 1 | 1 |
| 2601 | 54 | 2 | 164 | 65 | 8 | 2 | 1 | 1 | 4 | 1 | 1 | 1 | 1 | 1 | 1 | 1 | 1 | 1 |
| 2602 | 54 | 2 | 164 | 63 | 8 | 2 | 1 | 1 | 4 | 2 | 1 | 1 | 1 | 1 | 1 | 1 | 1 | 1 |
| 2603 | 54 | 2 | 164.4 | 54 | 6 | 2 | 1 | 1 | 4 | 1 | 1 | 1 | 2 | 1 | 2 | 1 | 1 | 1 |
| 2604 | 54 | 2 | 165 | 60 | 6 | 2 | 1 | 1 | 4 | 1 | 1 | 1 | 1 | 1 | 2 | 2 | 1 | 1 |
| 2605 | 54 | 2 | 165 | 45 | 8 | 2 | 1 | 1 | 5 | 1 | 1 | 1 | 1 | 1 | 1 | 1 | 1 | 1 |
| 2606 | 54 | 2 | 166 | 69 | 8 | 2 | 1 | 1 | 4 | 4 | 1 | 1 | 1 | 1 | 2 | 1 | 1 | 1 |
| 2607 | 54 | 2 | 167 | 52 | 8 | 2 | 1 | 1 | 4 | 3 | 1 | 1 | 1 | 1 | 2 | 1 | 1 | 1 |
| 2608 | 54 | 2 | 167.5 | 65 | 1 | 1 | 1 | 1 | 5 | 4 | 1 | 1 | 1 | 1 | 1 | 1 | 1 | 1 |
| 2609 | 54 | 2 | 169 | 60 | 8 | 2 | 1 | 1 | 4 | 1 | 1 | 1 | 1 | 1 | 1 | 1 | 1 | 1 |
| 2610 | 54 | 2 | 170 | 77 | 8 | 2 | 1 | 1 | 3 | 4 | 1 | 1 | 1 | 1 | 1 | 1 | 1 | 1 |
| 2611 | 54 | 1 | 156 | 65 | 8 | 2 | 1 | 1 | 6 | 4 | 1 | 1 | 1 | 2 | 1 | 1 | 1 | 1 |
| 2612 | 54 | 1 | 157 | 54 | 4 | 2 | 1 | 1 | 4 | 1 | 1 | 1 | 1 | 1 | 1 | 1 | 1 | 1 |
| 2613 | 54 | 1 | 157 | 46 | 8 | 2 | 1 | 1 | 3 | 3 | 1 | 1 | 1 | 1 | 1 | 1 | 1 | 1 |
| 2614 | 54 | 1 | 158 | 61 | 8 | 2 | 1 | 1 | 3 | 3 | 1 | 1 | 1 | 1 | 1 | 1 | 1 | 2 |
| 2615 | 54 | 1 | 160 | 65 | 4 | 2 | 1 | 1 | 4 | 4 | 1 | 1 | 1 | 1 | 1 | 1 | 3 | 1 |
| 2616 | 54 | 1 | 161 | 60 | 6 | 1 | 1 | 1 | 4 | 1 | 1 | 1 | 1 | 1 | 1 | 1 | 1 | 1 |
| 2617 | 54 | 1 | 162 | 82 | 8 | 2 | 1 | 1 | 3 | 4 | 1 | 1 | 1 | 1 | 1 | 1 | 1 | 1 |
| 2618 | 54 | 1 | 163 | 63 | 8 | 2 | 1 | 1 | 4 | 4 | 1 | 1 | 1 | 1 | 1 | 1 | 2 | 1 |
| 2619 | 54 | 1 | 164 | 50 | 1 | 2 | 1 | 1 | 4 | 4 | 1 | 1 | 1 | 1 | 1 | 1 | 1 | 1 |
| 2620 | 54 | 1 | 165 | 63 | 8 | 2 | 1 | 1 | 5 | 4 | 1 | 1 | 2 | 1 | 1 | 1 | 1 | 1 |
| 2621 | 54 | 1 | 168 | 72 | 4 | 2 | 1 | 3 | 3 | 1 | 1 | 1 | 1 | 1 | 2 | 2 | 3 | 1 |
| 2622 | 54 | 1 | 168 | 70 | 4 | 2 | 1 | 1 | 4 | 4 | 1 | 1 | 1 | 1 | 1 | 1 | 3 | 1 |
| 2623 | 54 | 1 | 168 | 65 | 8 | 2 | 1 | 1 | 5 | 3 | 2 | 1 | 1 | 2 | 1 | 1 | 1 | 1 |
| 2624 | 54 | 1 | 170 | 63 | 8 | 4 | 1 | 2 | 4 | 3 | 1 | 1 | 1 | 1 | 1 | 1 | 3 | 1 |
| 2625 | 54 | 1 | 170 | 60 | 8 | 2 | 1 | 1 | 6 | 4 | 1 | 1 | 1 | 1 | 1 | 1 | 1 | 1 |
| 2626 | 54 | 1 | 171 | 91.5 | 2 | 2 | 1 | 4 | 4 | 3 | 2 | 2 | 1 | 1 | 1 | 1 | 3 | 1 |
| 2627 | 54 | 1 | 171 | 64.2 | 6 | 2 | 1 | 2 | 4 | 1 | 1 | 1 | 1 | 1 | 1 | 1 | 1 | 1 |
| 2628 | 54 | 1 | 173 | 76.5 | 8 | 2 | 1 | 1 | 3 | 4 | 1 | 1 | 1 | 1 | 1 | 1 | 3 | 1 |
| 2629 | 54 | 1 | 173 | 54.3 | 8 | 2 | 1 | 2 | 4 | 3 | 1 | 1 | 1 | 1 | 1 | 1 | 3 | 1 |
| 2630 | 54 | 1 | 175 | 70 | 8 | 2 | 1 | 1 | 5 | 3 | 1 | 1 | 1 | 1 | 1 | 1 | 1 | 1 |
| 2631 | 54 | 1 | 176 | 81.5 | 8 | 2 | 1 | 2 | 4 | 3 | 1 | 1 | 1 | 1 | 1 | 1 | 1 | 1 |
| 2632 | 54 | 1 | 176 | 77 | 8 | 2 | 1 | 1 | 5 | 2 | 1 | 1 | 1 | 1 | 1 | 1 | 1 | 1 |
| 2633 | 54 | 1 | 176 | 62 | 3 | 2 | 1 | 1 | 5 | 4 | 1 | 1 | 1 | 1 | 1 | 1 | 1 | 1 |
| 2634 | 55 | 2 | 142 | 55 | 8 | 2 | 1 | 1 | 3 | 1 | 1 | 1 | 2 | 1 | 1 | 1 | 1 | 1 |
| 2635 | 55 | 2 | 144.8 | 45 | 8 | 2 | 1 | 1 | 2 | 3 | 1 | 1 | 1 | 1 | 2 | 1 | 1 | 1 |
| 2636 | 55 | 2 | 147.5 | 51.8 | 8 | 2 | 1 | 1 | 4 | 1 | 1 | 1 | 2 | 1 | 1 | 1 | 1 | 1 |
| 2637 | 55 | 2 | 149 | 60 | 8 | 2 | 1 | 1 | 4 | 3 | 1 | 1 | 1 | 1 | 2 | 1 | 1 | 1 |
| 2638 | 55 | 2 | 149 | 55 | 8 | 2 | 1 | 1 | 3 | 4 | 1 | 1 | 1 | 1 | 1 | 1 | 1 | 1 |
| 2639 | 55 | 2 | 149 | 46.4 | 2 | 2 | 1 | 1 | 3 | 1 | 1 | 1 | 2 | 1 | 1 | 1 | 1 | 1 |
| 2640 | 55 | 2 | 149 | 43 | 8 | 1 | 1 | 1 | 2 | 4 | 1 | 1 | 1 | 1 | 1 | 1 | 1 | 1 |
| 2641 | 55 | 2 | 149.5 | 67 | 8 | 2 | 1 | 1 | 4 | 4 | 1 | 1 | 1 | 1 | 1 | 1 | 1 | 1 |
| 2642 | 55 | 2 | 150 | 65 | 8 | 2 | 1 | 1 | 3 | 3 | 1 | 1 | 1 | 1 | 1 | 1 | 1 | 1 |
| 2643 | 55 | 2 | 150 | 63 | 1 | 1 | 1 | 1 | 4 | 1 | 1 | 1 | 1 | 1 | 1 | 1 | 1 | 1 |
| 2644 | 55 | 2 | 150 | 55 | 8 | 4 | 1 | 2 | 4 | 3 | 1 | 1 | 1 | 1 | 1 | 1 | 1 | 1 |
| 2645 | 55 | 2 | 150 | 50 | 8 | 3 | 1 | 1 | 4 | 1 | 1 | 1 | 1 | 1 | 1 | 1 | 1 | 1 |
| 2646 | 55 | 2 | 150 | 50 | 8 | 3 | 1 | 1 | 3 | 1 | 1 | 1 | 1 | 1 | 1 | 1 | 1 | 1 |
| 2647 | 55 | 2 | 151 | 56.5 | 8 | 2 | 1 | 1 | 4 | 4 | 1 | 1 | 1 | 1 | 2 | 1 | 1 | 1 |
| 2648 | 55 | 2 | 151 | 48 | 4 | 2 | 1 | 1 | 4 | 1 | 1 | 1 | 2 | 1 | 2 | 1 | 1 | 1 |
| 2649 | 55 | 2 | 151.5 | 64.1 | 8 | 2 | 1 | 2 | 4 | 1 | 1 | 1 | 2 | 1 | 1 | 1 | 1 | 1 |
| 2650 | 55 | 2 | 151.5 | 50.8 | 8 | 2 | 1 | 1 | 4 | 1 | 1 | 1 | 1 | 1 | 1 | 1 | 1 | 1 |
| 2651 | 55 | 2 | 152 | 62.7 | 4 | 2 | 1 | 1 | 4 | 2 | 1 | 1 | 1 | 1 | 1 | 1 | 1 | 1 |
| 2652 | 55 | 2 | 152.5 | 56.5 | 1 | 2 | 1 | 1 | 4 | 1 | 1 | 1 | 1 | 1 | 1 | 1 | 1 | 2 |
| 2653 | 55 | 2 | 153 | 67 | 8 | 2 | 1 | 1 | 4 | 2 | 1 | 1 | 1 | 1 | 1 | 1 | 1 | 1 |
| 2654 | 55 | 2 | 153 | 65.5 | 4 | 2 | 1 | 1 | 4 | 3 | 1 | 1 | 1 | 1 | 1 | 1 | 1 | 1 |
| 2655 | 55 | 2 | 153 | 60 | 8 | 2 | 1 | 1 | 4 | 1 | 1 | 1 | 1 | 1 | 1 | 1 | 1 | 1 |
| 2656 | 55 | 2 | 153 | 55 | 4 | 2 | 1 | 1 | 3 | 4 | 1 | 1 | 2 | 1 | 2 | 1 | 1 | 1 |
| 2657 | 55 | 2 | 153 | 54.6 | 8 | 2 | 1 | 1 | 4 | 3 | 1 | 1 | 2 | 1 | 2 | 1 | 1 | 1 |
| 2658 | 55 | 2 | 153 | 52.5 | 8 | 2 | 1 | 1 | 4 | 1 | 1 | 1 | 1 | 1 | 1 | 1 | 1 | 1 |
| 2659 | 55 | 2 | 153 | 52.4 | 8 | 3 | 1 | 3 | 4 | 3 | 1 | 1 | 1 | 1 | 1 | 1 | 1 | 1 |
| 2660 | 55 | 2 | 153 | 51.5 | 3 | 2 | 1 | 1 | 4 | 2 | 1 | 1 | 1 | 1 | 2 | 1 | 1 | 1 |
| 2661 | 55 | 2 | 153 | 50 | 8 | 2 | 1 | 1 | 4 | 1 | 1 | 1 | 1 | 1 | 1 | 1 | 1 | 1 |
| 2662 | 55 | 2 | 153 | 50 | 8 | 2 | 1 | 1 | 3 | 3 | 1 | 1 | 2 | 1 | 1 | 1 | 1 | 1 |
| 2663 | 55 | 2 | 153 | 49.6 | 2 | 1 | 1 | 1 | 4 | 1 | 1 | 1 | 1 | 1 | 1 | 1 | 1 | 1 |
| 2664 | 55 | 2 | 153 | 46.5 | 3 | 2 | 1 | 1 | 4 | 1 | 1 | 1 | 1 | 1 | 2 | 2 | 1 | 1 |
| 2665 | 55 | 2 | 153 | 45 | 8 | 2 | 1 | 1 | 4 | 3 | 1 | 1 | 1 | 1 | 1 | 1 | 1 | 1 |
| 2666 | 55 | 2 | 154 | 66 | 2 | 2 | 1 | 1 | 4 | 3 | 1 | 1 | 2 | 1 | 1 | 1 | 1 | 1 |
| 2667 | 55 | 2 | 154 | 64.5 | 1 | 2 | 1 | 1 | 6 | 3 | 1 | 1 | 1 | 1 | 1 | 1 | 1 | 2 |
| 2668 | 55 | 2 | 154 | 64 | 8 | 2 | 1 | 2 | 4 | 2 | 1 | 1 | 1 | 1 | 1 | 1 | 1 | 1 |
| 2669 | 55 | 2 | 154 | 64 | 8 | 2 | 1 | 1 | 4 | 1 | 1 | 1 | 1 | 1 | 2 | 1 | 1 | 1 |
| 2670 | 55 | 2 | 154 | 56 | 8 | 2 | 1 | 1 | 5 | 4 | 1 | 1 | 1 | 1 | 1 | 1 | 1 | 1 |
| 2671 | 55 | 2 | 154 | 52 | 8 | 2 | 1 | 1 | 4 | 2 | 1 | 1 | 1 | 1 | 2 | 1 | 1 | 1 |
| 2672 | 55 | 2 | 154 | 52 | 6 | 2 | 1 | 1 | 4 | 1 | 1 | 1 | 1 | 1 | 1 | 1 | 1 | 1 |
| 2673 | 55 | 2 | 154 | 49 | 8 | 4 | 1 | 1 | 4 | 4 | 1 | 1 | 1 | 1 | 1 | 1 | 1 | 1 |
| 2674 | 55 | 2 | 154 | 45 | 8 | 2 | 1 | 1 | 4 | 3 | 1 | 1 | 1 | 1 | 1 | 1 | 1 | 1 |
| 2675 | 55 | 2 | 154.5 | 57.2 | 6 | 2 | 1 | 1 | 4 | 1 | 1 | 1 | 1 | 1 | 1 | 1 | 1 | 1 |
| 2676 | 55 | 2 | 155 | 66 | 8 | 2 | 1 | 1 | 3 | 1 | 1 | 1 | 1 | 1 | 1 | 1 | 1 | 1 |
| 2677 | 55 | 2 | 155 | 58 | 8 | 2 | 1 | 1 | 4 | 2 | 1 | 1 | 1 | 1 | 1 | 1 | 1 | 1 |
| 2678 | 55 | 2 | 155 | 55 | 8 | 2 | 1 | 1 | 4 | 1 | 1 | 1 | 1 | 1 | 1 | 1 | 1 | 1 |
| 2679 | 55 | 2 | 155 | 52 | 8 | 2 | 1 | 1 | 4 | 1 | 1 | 1 | 1 | 1 | 1 | 1 | 1 | 1 |
| 2680 | 55 | 2 | 155 | 51 | 8 | 2 | 1 | 1 | 4 | 1 | 1 | 1 | 1 | 1 | 1 | 1 | 1 | 1 |
| 2681 | 55 | 2 | 155 | 50 | 8 | 2 | 1 | 1 | 3 | 1 | 1 | 1 | 1 | 1 | 1 | 1 | 1 | 1 |
| 2682 | 55 | 2 | 155 | 49 | 8 | 2 | 1 | 1 | 3 | 2 | 1 | 1 | 1 | 1 | 1 | 1 | 1 | 1 |
| 2683 | 55 | 2 | 155 | 40 | 8 | 2 | 1 | 1 | 4 | 1 | 1 | 1 | 1 | 1 | 1 | 1 | 1 | 1 |
| 2684 | 55 | 2 | 155.3 | 56.5 | 8 | 2 | 1 | 1 | 3 | 1 | 1 | 1 | 1 | 1 | 1 | 1 | 1 | 1 |
| 2685 | 55 | 2 | 155.5 | 50.6 | 8 | 2 | 1 | 1 | 4 | 1 | 1 | 1 | 2 | 2 | 1 | 1 | 1 | 2 |
| 2686 | 55 | 2 | 156 | 70 | 8 | 2 | 1 | 1 | 4 | 1 | 1 | 1 | 1 | 1 | 1 | 1 | 1 | 1 |
| 2687 | 55 | 2 | 156 | 60 | 8 | 2 | 1 | 1 | 4 | 4 | 1 | 1 | 1 | 1 | 1 | 1 | 1 | 1 |
| 2688 | 55 | 2 | 156 | 60 | 8 | 2 | 1 | 1 | 4 | 4 | 1 | 1 | 1 | 1 | 1 | 1 | 1 | 1 |
| 2689 | 55 | 2 | 156 | 59 | 8 | 2 | 1 | 1 | 4 | 1 | 1 | 1 | 2 | 1 | 1 | 1 | 1 | 1 |
| 2690 | 55 | 2 | 156 | 53 | 8 | 2 | 1 | 1 | 4 | 3 | 1 | 1 | 1 | 1 | 1 | 1 | 1 | 1 |
| 2691 | 55 | 2 | 156 | 53 | 6 | 2 | 1 | 1 | 3 | 1 | 1 | 1 | 1 | 1 | 1 | 1 | 1 | 1 |
| 2692 | 55 | 2 | 156 | 52 | 8 | 3 | 1 | 1 | 4 | 1 | 1 | 1 | 1 | 1 | 1 | 1 | 1 | 1 |
| 2693 | 55 | 2 | 156 | 49 | 8 | 2 | 1 | 1 | 4 | 1 | 1 | 1 | 1 | 1 | 1 | 1 | 1 | 1 |
| 2694 | 55 | 2 | 156 | 46 | 3 | 1 | 1 | 1 | 4 | 1 | 1 | 1 | 2 | 1 | 1 | 1 | 1 | 1 |
| 2695 | 55 | 2 | 156 | 41 | 8 | 2 | 1 | 1 | 4 | 4 | 1 | 1 | 1 | 1 | 1 | 1 | 1 | 1 |
| 2696 | 55 | 2 | 156.6 | 56.9 | 8 | 2 | 1 | 1 | 4 | 1 | 1 | 1 | 1 | 1 | 1 | 1 | 1 | 1 |
| 2697 | 55 | 2 | 157 | 68 | 4 | 2 | 1 | 1 | 3 | 1 | 1 | 1 | 1 | 1 | 1 | 1 | 1 | 1 |
| 2698 | 55 | 2 | 157 | 65 | 8 | 2 | 1 | 1 | 4 | 4 | 1 | 1 | 1 | 1 | 1 | 1 | 1 | 1 |
| 2699 | 55 | 2 | 157 | 62 | 8 | 2 | 1 | 1 | 3 | 2 | 1 | 1 | 1 | 1 | 1 | 1 | 1 | 1 |
| 2700 | 55 | 2 | 157 | 54 | 8 | 2 | 1 | 1 | 3 | 2 | 1 | 1 | 1 | 1 | 1 | 1 | 1 | 1 |
| 2701 | 55 | 2 | 158 | 62 | 8 | 2 | 1 | 1 | 2 | 3 | 1 | 1 | 2 | 1 | 1 | 1 | 1 | 1 |
| 2702 | 55 | 2 | 158 | 58 | 8 | 2 | 1 | 1 | 4 | 4 | 1 | 1 | 2 | 1 | 2 | 1 | 1 | 1 |
| 2703 | 55 | 2 | 158 | 57.5 | 8 | 2 | 1 | 1 | 4 | 2 | 1 | 1 | 1 | 1 | 1 | 1 | 1 | 1 |
| 2704 | 55 | 2 | 158 | 55 | 8 | 2 | 1 | 1 | 4 | 3 | 1 | 1 | 1 | 1 | 1 | 1 | 1 | 1 |
| 2705 | 55 | 2 | 158 | 53 | 3 | 2 | 1 | 1 | 4 | 2 | 1 | 1 | 1 | 1 | 1 | 1 | 1 | 1 |
| 2706 | 55 | 2 | 158 | 51 | 8 | 2 | 1 | 1 | 4 | 4 | 1 | 1 | 1 | 1 | 1 | 1 | 1 | 1 |
| 2707 | 55 | 2 | 159 | 65 | 8 | 2 | 1 | 1 | 3 | 3 | 1 | 1 | 1 | 1 | 1 | 1 | 1 | 1 |
| 2708 | 55 | 2 | 159 | 65 | 8 | 2 | 1 | 1 | 2 | 2 | 1 | 1 | 1 | 1 | 1 | 1 | 1 | 1 |
| 2709 | 55 | 2 | 159 | 57 | 3 | 2 | 1 | 1 | 4 | 1 | 1 | 1 | 2 | 1 | 1 | 1 | 1 | 2 |
| 2710 | 55 | 2 | 159 | 55 | 8 | 3 | 1 | 1 | 4 | 1 | 1 | 1 | 1 | 2 | 1 | 1 | 1 | 2 |
| 2711 | 55 | 2 | 159 | 55 | 8 | 2 | 1 | 1 | 4 | 4 | 1 | 1 | 1 | 1 | 1 | 1 | 1 | 1 |
| 2712 | 55 | 2 | 159 | 53 | 8 | 2 | 1 | 1 | 3 | 4 | 1 | 1 | 1 | 1 | 1 | 1 | 1 | 1 |
| 2713 | 55 | 2 | 159 | 48 | 8 | 2 | 1 | 1 | 5 | 1 | 1 | 1 | 1 | 1 | 1 | 1 | 1 | 1 |
| 2714 | 55 | 2 | 159.6 | 59.6 | 4 | 2 | 1 | 1 | 4 | 2 | 1 | 1 | 1 | 1 | 1 | 1 | 1 | 1 |
| 2715 | 55 | 2 | 160 | 67 | 6 | 2 | 1 | 2 | 4 | 4 | 1 | 1 | 1 | 1 | 1 | 1 | 3 | 1 |
| 2716 | 55 | 2 | 160 | 62.5 | 4 | 2 | 1 | 1 | 4 | 3 | 1 | 1 | 1 | 1 | 2 | 1 | 1 | 1 |
| 2717 | 55 | 2 | 160 | 62 | 6 | 1 | 1 | 1 | 4 | 2 | 1 | 1 | 1 | 1 | 2 | 1 | 1 | 1 |
| 2718 | 55 | 2 | 160 | 60 | 8 | 2 | 1 | 1 | 3 | 4 | 1 | 1 | 2 | 1 | 1 | 1 | 1 | 1 |
| 2719 | 55 | 2 | 160 | 58 | 8 | 2 | 1 | 1 | 5 | 1 | 1 | 2 | 2 | 1 | 1 | 1 | 1 | 1 |
| 2720 | 55 | 2 | 160 | 57 | 3 | 2 | 1 | 1 | 4 | 4 | 1 | 1 | 2 | 1 | 1 | 1 | 1 | 1 |
| 2721 | 55 | 2 | 160 | 55 | 8 | 2 | 1 | 1 | 6 | 4 | 1 | 1 | 1 | 1 | 1 | 1 | 1 | 1 |
| 2722 | 55 | 2 | 160 | 55 | 8 | 2 | 1 | 1 | 4 | 1 | 1 | 1 | 1 | 1 | 1 | 1 | 1 | 1 |
| 2723 | 55 | 2 | 160 | 55 | 8 | 2 | 1 | 1 | 4 | 1 | 1 | 1 | 1 | 1 | 1 | 1 | 1 | 1 |
| 2724 | 55 | 2 | 160 | 54 | 8 | 2 | 1 | 1 | 4 | 1 | 1 | 1 | 1 | 1 | 2 | 1 | 1 | 1 |
| 2725 | 55 | 2 | 160 | 50 | 8 | 2 | 1 | 1 | 4 | 2 | 1 | 1 | 1 | 1 | 1 | 1 | 1 | 1 |
| 2726 | 55 | 2 | 160 | 50 | 8 | 2 | 1 | 1 | 3 | 2 | 1 | 2 | 1 | 1 | 1 | 1 | 1 | 1 |
| 2727 | 55 | 2 | 160 | 50 | 6 | 2 | 1 | 1 | 4 | 1 | 1 | 1 | 1 | 1 | 1 | 1 | 1 | 2 |
| 2728 | 55 | 2 | 160.5 | 54.9 | 2 | 2 | 1 | 1 | 4 | 1 | 1 | 1 | 1 | 1 | 2 | 1 | 1 | 1 |
| 2729 | 55 | 2 | 161 | 60 | 8 | 2 | 1 | 1 | 3 | 3 | 1 | 1 | 1 | 1 | 2 | 2 | 1 | 1 |
| 2730 | 55 | 2 | 161 | 55 | 8 | 2 | 1 | 1 | 4 | 4 | 1 | 1 | 1 | 1 | 1 | 1 | 1 | 1 |
| 2731 | 55 | 2 | 161.5 | 83 | 8 | 2 | 1 | 1 | 4 | 4 | 1 | 1 | 1 | 1 | 1 | 1 | 1 | 1 |
| 2732 | 55 | 2 | 162 | 67 | 4 | 2 | 1 | 1 | 3 | 2 | 1 | 1 | 1 | 1 | 2 | 1 | 1 | 1 |
| 2733 | 55 | 2 | 162 | 65 | 8 | 2 | 1 | 1 | 4 | 1 | 1 | 1 | 2 | 2 | 1 | 1 | 1 | 2 |
| 2734 | 55 | 2 | 162 | 62 | 1 | 1 | 1 | 2 | 4 | 3 | 1 | 1 | 1 | 1 | 1 | 1 | 1 | 1 |
| 2735 | 55 | 2 | 162 | 59.7 | 8 | 2 | 1 | 1 | 4 | 1 | 1 | 1 | 1 | 1 | 2 | 1 | 1 | 1 |
| 2736 | 55 | 2 | 163 | 77.5 | 3 | 2 | 1 | 1 | 4 | 1 | 1 | 1 | 1 | 1 | 2 | 1 | 1 | 1 |
| 2737 | 55 | 2 | 163 | 67 | 6 | 2 | 1 | 1 | 4 | 1 | 1 | 1 | 1 | 1 | 1 | 1 | 1 | 1 |
| 2738 | 55 | 2 | 163 | 65 | 8 | 2 | 1 | 1 | 3 | 1 | 1 | 1 | 1 | 1 | 1 | 1 | 1 | 1 |
| 2739 | 55 | 2 | 163 | 64 | 8 | 2 | 1 | 1 | 4 | 2 | 1 | 1 | 1 | 1 | 1 | 1 | 1 | 2 |
| 2740 | 55 | 2 | 163 | 62 | 1 | 2 | 1 | 1 | 4 | 1 | 1 | 1 | 1 | 1 | 1 | 1 | 1 | 1 |
| 2741 | 55 | 2 | 164 | 60 | 6 | 2 | 1 | 1 | 4 | 2 | 1 | 1 | 2 | 1 | 1 | 1 | 1 | 1 |
| 2742 | 55 | 2 | 164 | 59.5 | 8 | 2 | 1 | 1 | 3 | 2 | 1 | 1 | 1 | 1 | 1 | 1 | 1 | 1 |
| 2743 | 55 | 2 | 164 | 55 | 8 | 2 | 1 | 1 | 4 | 1 | 1 | 1 | 2 | 1 | 2 | 1 | 1 | 1 |
| 2744 | 55 | 2 | 164 | 47.6 | 8 | 2 | 1 | 1 | 4 | 1 | 1 | 1 | 1 | 1 | 1 | 1 | 1 | 1 |
| 2745 | 55 | 2 | 165 | 72.5 | 8 | 2 | 1 | 1 | 4 | 4 | 1 | 1 | 1 | 1 | 1 | 1 | 1 | 1 |
| 2746 | 55 | 2 | 165 | 55 | 8 | 2 | 1 | 1 | 4 | 1 | 1 | 1 | 1 | 1 | 2 | 1 | 1 | 1 |
| 2747 | 55 | 2 | 168 | 68 | 8 | 1 | 1 | 1 | 5 | 3 | 1 | 1 | 1 | 1 | 1 | 1 | 1 | 1 |
| 2748 | 55 | 2 | 168 | 65 | 8 | 4 | 1 | 1 | 4 | 2 | 1 | 1 | 1 | 1 | 1 | 1 | 1 | 1 |
| 2749 | 55 | 2 | 170 | 80 | 8 | 2 | 1 | 1 | 4 | 3 | 1 | 1 | 1 | 1 | 1 | 1 | 1 | 2 |
| 2750 | 55 | 2 | 176 | 69 | 8 | 2 | 1 | 1 | 6 | 4 | 1 | 1 | 1 | 1 | 1 | 1 | 1 | 1 |
| 2751 | 55 | 1 | 160 | 55 | 8 | 2 | 1 | 1 | 5 | 2 | 1 | 1 | 1 | 1 | 1 | 1 | 1 | 1 |
| 2752 | 55 | 1 | 161.5 | 40 | 8 | 2 | 1 | 1 | 4 | 2 | 1 | 1 | 1 | 1 | 2 | 1 | 3 | 1 |
| 2753 | 55 | 1 | 163 | 80 | 8 | 2 | 1 | 2 | 1 | 1 | 1 | 1 | 1 | 1 | 2 | 1 | 3 | 1 |
| 2754 | 55 | 1 | 163 | 69.5 | 8 | 2 | 1 | 1 | 4 | 4 | 1 | 1 | 1 | 1 | 1 | 1 | 1 | 1 |
| 2755 | 55 | 1 | 163 | 63 | 8 | 2 | 1 | 2 | 3 | 4 | 1 | 1 | 1 | 1 | 2 | 1 | 3 | 1 |
| 2756 | 55 | 1 | 164 | 56 | 8 | 2 | 1 | 2 | 4 | 3 | 1 | 1 | 1 | 2 | 1 | 1 | 3 | 1 |
| 2757 | 55 | 1 | 164 | 54 | 8 | 2 | 1 | 1 | 4 | 4 | 1 | 1 | 1 | 1 | 1 | 1 | 3 | 1 |
| 2758 | 55 | 1 | 165 | 80 | 8 | 2 | 1 | 1 | 3 | 1 | 1 | 1 | 1 | 2 | 1 | 1 | 3 | 1 |
| 2759 | 55 | 1 | 165 | 60 | 4 | 2 | 1 | 1 | 4 | 2 | 1 | 1 | 1 | 1 | 1 | 1 | 2 | 1 |
| 2760 | 55 | 1 | 165 | 55 | 8 | 2 | 1 | 4 | 4 | 1 | 1 | 1 | 1 | 1 | 1 | 1 | 3 | 1 |
| 2761 | 55 | 1 | 165 | 51 | 8 | 2 | 1 | 4 | 4 | 4 | 1 | 1 | 1 | 1 | 1 | 1 | 3 | 1 |
| 2762 | 55 | 1 | 166 | 75 | 8 | 2 | 1 | 1 | 3 | 4 | 1 | 1 | 1 | 1 | 1 | 1 | 1 | 2 |
| 2763 | 55 | 1 | 167 | 64 | 8 | 4 | 1 | 2 | 5 | 1 | 1 | 1 | 1 | 1 | 2 | 1 | 3 | 1 |
| 2764 | 55 | 1 | 168 | 66 | 8 | 1 | 1 | 1 | 1 | 4 | 1 | 1 | 1 | 1 | 1 | 1 | 1 | 1 |
| 2765 | 55 | 1 | 169 | 72 | 1 | 2 | 1 | 2 | 4 | 2 | 1 | 1 | 1 | 1 | 1 | 1 | 3 | 1 |
| 2766 | 55 | 1 | 169 | 65 | 8 | 2 | 1 | 1 | 6 | 4 | 1 | 1 | 1 | 1 | 1 | 1 | 1 | 1 |
| 2767 | 55 | 1 | 170 | 80 | 8 | 2 | 1 | 1 | 4 | 3 | 1 | 1 | 1 | 1 | 1 | 1 | 1 | 1 |
| 2768 | 55 | 1 | 170 | 66 | 1 | 2 | 1 | 1 | 3 | 2 | 1 | 1 | 1 | 1 | 1 | 1 | 1 | 2 |
| 2769 | 55 | 1 | 170 | 65 | 4 | 2 | 2 | 1 | 4 | 1 | 1 | 1 | 1 | 1 | 1 | 1 | 3 | 1 |
| 2770 | 55 | 1 | 171 | 68.8 | 3 | 2 | 1 | 1 | 5 | 1 | 1 | 1 | 1 | 1 | 1 | 1 | 2 | 1 |
| 2771 | 55 | 1 | 171.5 | 66.5 | 8 | 2 | 1 | 2 | 4 | 1 | 1 | 1 | 1 | 1 | 1 | 1 | 3 | 1 |
| 2772 | 55 | 1 | 172 | 73 | 8 | 2 | 1 | 1 | 4 | 4 | 1 | 1 | 1 | 1 | 1 | 1 | 1 | 1 |
| 2773 | 55 | 1 | 173 | 65 | 8 | 2 | 1 | 1 | 4 | 1 | 1 | 1 | 1 | 1 | 1 | 1 | 3 | 1 |
| 2774 | 55 | 1 | 175 | 87.5 | 4 | 2 | 1 | 2 | 4 | 4 | 1 | 1 | 1 | 1 | 1 | 1 | 3 | 1 |
| 2775 | 56 | 2 | 82 | 55 | 8 | 2 | 1 | 1 | 4 | 2 | 1 | 1 | 1 | 1 | 1 | 1 | 1 | 1 |
| 2776 | 56 | 2 | 144.5 | 48.5 | 4 | 3 | 1 | 1 | 2 | 2 | 1 | 1 | 1 | 1 | 2 | 1 | 1 | 1 |
| 2777 | 56 | 2 | 146 | 52.8 | 3 | 2 | 1 | 1 | 3 | 2 | 1 | 1 | 1 | 2 | 2 | 1 | 1 | 2 |
| 2778 | 56 | 2 | 147.5 | 50 | 8 | 2 | 1 | 1 | 2 | 4 | 1 | 1 | 1 | 1 | 1 | 1 | 1 | 1 |
| 2779 | 56 | 2 | 149 | 50 | 8 | 1 | 1 | 1 | 4 | 2 | 1 | 1 | 1 | 1 | 1 | 1 | 1 | 1 |
| 2780 | 56 | 2 | 150 | 65 | 4 | 2 | 1 | 1 | 3 | 2 | 1 | 1 | 1 | 1 | 2 | 1 | 1 | 1 |
| 2781 | 56 | 2 | 150 | 65 | 3 | 2 | 1 | 1 | 4 | 3 | 1 | 1 | 1 | 1 | 1 | 1 | 1 | 1 |
| 2782 | 56 | 2 | 150 | 60 | 4 | 2 | 1 | 1 | 3 | 2 | 1 | 1 | 1 | 1 | 1 | 1 | 1 | 1 |
| 2783 | 56 | 2 | 150 | 55 | 8 | 2 | 1 | 1 | 6 | 4 | 1 | 1 | 1 | 1 | 1 | 1 | 1 | 1 |
| 2784 | 56 | 2 | 150 | 49.5 | 8 | 2 | 1 | 1 | 4 | 1 | 1 | 1 | 1 | 1 | 1 | 1 | 1 | 1 |
| 2785 | 56 | 2 | 150 | 48.5 | 8 | 2 | 1 | 1 | 4 | 1 | 1 | 2 | 1 | 1 | 1 | 1 | 1 | 2 |
| 2786 | 56 | 2 | 151 | 63 | 8 | 2 | 1 | 1 | 3 | 1 | 1 | 1 | 1 | 1 | 2 | 1 | 1 | 2 |
| 2787 | 56 | 2 | 151 | 53 | 4 | 3 | 1 | 1 | 3 | 2 | 1 | 1 | 2 | 1 | 1 | 1 | 1 | 1 |
| 2788 | 56 | 2 | 152 | 67 | 8 | 2 | 1 | 1 | 4 | 1 | 1 | 1 | 1 | 1 | 1 | 1 | 1 | 2 |
| 2789 | 56 | 2 | 152 | 60 | 3 | 2 | 1 | 2 | 4 | 2 | 1 | 1 | 1 | 1 | 1 | 1 | 1 | 1 |
| 2790 | 56 | 2 | 152 | 57.5 | 8 | 2 | 1 | 1 | 3 | 4 | 1 | 1 | 1 | 1 | 1 | 1 | 1 | 1 |
| 2791 | 56 | 2 | 152 | 57 | 3 | 2 | 1 | 1 | 4 | 2 | 1 | 1 | 1 | 1 | 2 | 1 | 1 | 2 |
| 2792 | 56 | 2 | 152 | 55 | 8 | 2 | 1 | 1 | 3 | 2 | 1 | 1 | 1 | 1 | 1 | 1 | 1 | 1 |
| 2793 | 56 | 2 | 152 | 52.5 | 4 | 2 | 1 | 1 | 4 | 1 | 1 | 1 | 1 | 1 | 1 | 1 | 1 | 1 |
| 2794 | 56 | 2 | 152.5 | 59.4 | 8 | 2 | 1 | 1 | 4 | 2 | 1 | 1 | 2 | 1 | 1 | 1 | 1 | 1 |
| 2795 | 56 | 2 | 153 | 66 | 2 | 2 | 1 | 1 | 3 | 1 | 1 | 1 | 1 | 1 | 2 | 1 | 1 | 1 |
| 2796 | 56 | 2 | 153 | 53 | 8 | 2 | 1 | 1 | 2 | 1 | 1 | 1 | 1 | 1 | 1 | 1 | 1 | 1 |
| 2797 | 56 | 2 | 154 | 60.2 | 6 | 2 | 1 | 1 | 4 | 1 | 1 | 1 | 1 | 1 | 1 | 1 | 1 | 1 |
| 2798 | 56 | 2 | 154 | 52 | 8 | 2 | 1 | 1 | 3 | 3 | 1 | 1 | 1 | 1 | 1 | 1 | 1 | 1 |
| 2799 | 56 | 2 | 154 | 46 | 6 | 2 | 1 | 1 | 3 | 1 | 1 | 1 | 1 | 1 | 1 | 1 | 1 | 1 |
| 2800 | 56 | 2 | 154 | 40 | 8 | 2 | 1 | 1 | 5 | 3 | 1 | 1 | 1 | 2 | 1 | 1 | 1 | 2 |
| 2801 | 56 | 2 | 155 | 68 | 8 | 2 | 1 | 1 | 3 | 1 | 1 | 1 | 1 | 1 | 1 | 1 | 1 | 1 |
| 2802 | 56 | 2 | 155 | 57 | 8 | 2 | 1 | 1 | 4 | 1 | 1 | 1 | 1 | 1 | 1 | 1 | 1 | 1 |
| 2803 | 56 | 2 | 155 | 56 | 8 | 2 | 1 | 1 | 4 | 4 | 1 | 1 | 1 | 1 | 1 | 1 | 1 | 1 |
| 2804 | 56 | 2 | 155 | 52 | 8 | 2 | 1 | 1 | 2 | 4 | 1 | 1 | 2 | 1 | 1 | 1 | 1 | 1 |
| 2805 | 56 | 2 | 155 | 51 | 4 | 1 | 2 | 1 | 4 | 2 | 1 | 1 | 1 | 1 | 1 | 1 | 1 | 1 |
| 2806 | 56 | 2 | 155 | 50 | 8 | 3 | 1 | 1 | 4 | 2 | 1 | 1 | 1 | 1 | 1 | 1 | 1 | 1 |
| 2807 | 56 | 2 | 155.5 | 56.3 | 8 | 2 | 1 | 1 | 4 | 2 | 1 | 1 | 1 | 1 | 1 | 1 | 1 | 1 |
| 2808 | 56 | 2 | 156 | 65 | 8 | 2 | 1 | 1 | 2 | 4 | 1 | 1 | 1 | 1 | 1 | 1 | 1 | 1 |
| 2809 | 56 | 2 | 156 | 62 | 6 | 2 | 2 | 1 | 4 | 2 | 1 | 1 | 1 | 1 | 2 | 1 | 1 | 1 |
| 2810 | 56 | 2 | 156 | 59 | 8 | 2 | 1 | 1 | 3 | 1 | 1 | 1 | 1 | 1 | 1 | 1 | 1 | 2 |
| 2811 | 56 | 2 | 156 | 59 | 4 | 2 | 1 | 1 | 4 | 1 | 1 | 1 | 1 | 1 | 1 | 1 | 1 | 1 |
| 2812 | 56 | 2 | 156 | 58 | 8 | 2 | 1 | 1 | 5 | 4 | 1 | 1 | 1 | 1 | 1 | 1 | 1 | 1 |
| 2813 | 56 | 2 | 156 | 58 | 8 | 2 | 1 | 1 | 3 | 1 | 1 | 1 | 1 | 1 | 1 | 1 | 1 | 1 |
| 2814 | 56 | 2 | 156 | 56 | 2 | 2 | 1 | 1 | 4 | 4 | 2 | 1 | 1 | 1 | 1 | 1 | 1 | 1 |
| 2815 | 56 | 2 | 156 | 55 | 8 | 2 | 2 | 1 | 2 | 4 | 1 | 1 | 1 | 1 | 2 | 1 | 1 | 1 |
| 2816 | 56 | 2 | 156 | 54 | 8 | 2 | 1 | 1 | 3 | 4 | 1 | 1 | 1 | 1 | 1 | 1 | 1 | 2 |
| 2817 | 56 | 2 | 156 | 51 | 8 | 2 | 1 | 1 | 4 | 2 | 1 | 1 | 1 | 1 | 1 | 1 | 1 | 1 |
| 2818 | 56 | 2 | 156 | 51 | 8 | 2 | 1 | 1 | 4 | 1 | 1 | 1 | 1 | 1 | 1 | 1 | 1 | 1 |
| 2819 | 56 | 2 | 156 | 45 | 1 | 1 | 1 | 1 | 1 | 2 | 1 | 1 | 1 | 1 | 1 | 1 | 1 | 1 |
| 2820 | 56 | 2 | 156.8 | 50.8 | 6 | 2 | 1 | 1 | 3 | 4 | 1 | 1 | 2 | 1 | 1 | 1 | 1 | 1 |
| 2821 | 56 | 2 | 157 | 82 | 8 | 2 | 1 | 1 | 3 | 4 | 1 | 1 | 1 | 1 | 1 | 1 | 1 | 1 |
| 2822 | 56 | 2 | 157 | 65 | 8 | 2 | 1 | 1 | 3 | 4 | 3 | 1 | 1 | 1 | 1 | 1 | 1 | 1 |
| 2823 | 56 | 2 | 157 | 55 | 8 | 2 | 1 | 1 | 4 | 1 | 1 | 1 | 1 | 1 | 1 | 1 | 1 | 1 |
| 2824 | 56 | 2 | 157 | 54 | 8 | 2 | 1 | 1 | 3 | 4 | 1 | 1 | 1 | 1 | 1 | 1 | 1 | 1 |
| 2825 | 56 | 2 | 157 | 51 | 8 | 3 | 1 | 1 | 5 | 3 | 1 | 1 | 1 | 1 | 1 | 1 | 1 | 1 |
| 2826 | 56 | 2 | 157 | 46 | 8 | 2 | 1 | 1 | 4 | 3 | 1 | 1 | 1 | 1 | 1 | 1 | 1 | 1 |
| 2827 | 56 | 2 | 158 | 66 | 6 | 2 | 1 | 1 | 4 | 1 | 1 | 1 | 1 | 1 | 2 | 1 | 1 | 2 |
| 2828 | 56 | 2 | 158 | 56 | 8 | 2 | 1 | 1 | 4 | 3 | 1 | 1 | 2 | 1 | 1 | 1 | 1 | 1 |
| 2829 | 56 | 2 | 158 | 56 | 8 | 2 | 1 | 1 | 3 | 4 | 1 | 1 | 1 | 1 | 1 | 1 | 1 | 1 |
| 2830 | 56 | 2 | 158 | 55 | 8 | 2 | 1 | 1 | 5 | 1 | 1 | 1 | 1 | 1 | 1 | 1 | 1 | 1 |
| 2831 | 56 | 2 | 158 | 51 | 8 | 2 | 1 | 1 | 4 | 1 | 1 | 1 | 1 | 1 | 1 | 1 | 1 | 1 |
| 2832 | 56 | 2 | 158 | 51 | 8 | 2 | 1 | 1 | 3 | 4 | 1 | 1 | 1 | 1 | 1 | 1 | 1 | 1 |
| 2833 | 56 | 2 | 159 | 75 | 8 | 2 | 1 | 1 | 6 | 2 | 1 | 1 | 1 | 1 | 1 | 1 | 1 | 1 |
| 2834 | 56 | 2 | 159 | 69 | 8 | 2 | 1 | 1 | 4 | 2 | 2 | 1 | 1 | 1 | 1 | 1 | 1 | 1 |
| 2835 | 56 | 2 | 159 | 46.1 | 8 | 2 | 1 | 1 | 4 | 1 | 1 | 1 | 1 | 1 | 1 | 1 | 1 | 1 |
| 2836 | 56 | 2 | 159.5 | 55 | 8 | 2 | 1 | 1 | 4 | 1 | 1 | 1 | 2 | 1 | 1 | 1 | 1 | 1 |
| 2837 | 56 | 2 | 160 | 79 | 8 | 2 | 1 | 1 | 4 | 2 | 1 | 1 | 2 | 1 | 2 | 1 | 1 | 1 |
| 2838 | 56 | 2 | 160 | 71 | 8 | 2 | 1 | 1 | 4 | 2 | 2 | 1 | 1 | 1 | 1 | 1 | 1 | 2 |
| 2839 | 56 | 2 | 160 | 65 | 5 | 2 | 1 | 1 | 4 | 4 | 1 | 1 | 2 | 1 | 1 | 1 | 1 | 1 |
| 2840 | 56 | 2 | 160 | 58 | 4 | 2 | 1 | 1 | 5 | 1 | 1 | 1 | 1 | 1 | 1 | 1 | 1 | 1 |
| 2841 | 56 | 2 | 160 | 55 | 8 | 2 | 1 | 1 | 4 | 3 | 1 | 1 | 1 | 1 | 1 | 1 | 1 | 1 |
| 2842 | 56 | 2 | 160 | 55 | 2 | 2 | 1 | 1 | 4 | 3 | 1 | 1 | 2 | 1 | 1 | 1 | 1 | 1 |
| 2843 | 56 | 2 | 160 | 52 | 2 | 4 | 1 | 1 | 5 | 3 | 1 | 1 | 2 | 1 | 1 | 1 | 1 | 1 |
| 2844 | 56 | 2 | 160 | 50 | 8 | 2 | 1 | 1 | 3 | 4 | 1 | 1 | 1 | 1 | 1 | 1 | 1 | 1 |
| 2845 | 56 | 2 | 160 | 48.1 | 4 | 2 | 1 | 1 | 2 | 2 | 1 | 1 | 1 | 1 | 1 | 1 | 1 | 1 |
| 2846 | 56 | 2 | 160.5 | 56.6 | 6 | 2 | 1 | 1 | 4 | 3 | 1 | 1 | 2 | 1 | 1 | 1 | 1 | 1 |
| 2847 | 56 | 2 | 161 | 59 | 8 | 2 | 1 | 2 | 3 | 2 | 1 | 1 | 1 | 1 | 1 | 1 | 1 | 1 |
| 2848 | 56 | 2 | 162 | 73 | 8 | 2 | 2 | 1 | 4 | 1 | 1 | 1 | 1 | 1 | 2 | 1 | 1 | 1 |
| 2849 | 56 | 2 | 162 | 60 | 8 | 2 | 1 | 1 | 4 | 2 | 1 | 1 | 1 | 1 | 1 | 1 | 1 | 2 |
| 2850 | 56 | 2 | 162 | 53 | 8 | 2 | 1 | 1 | 4 | 1 | 1 | 1 | 1 | 1 | 2 | 1 | 1 | 1 |
| 2851 | 56 | 2 | 164 | 91 | 8 | 2 | 1 | 1 | 2 | 1 | 1 | 1 | 2 | 1 | 1 | 1 | 1 | 1 |
| 2852 | 56 | 2 | 164 | 65 | 4 | 2 | 1 | 1 | 4 | 1 | 1 | 1 | 1 | 1 | 1 | 1 | 1 | 1 |
| 2853 | 56 | 2 | 164 | 58 | 8 | 2 | 1 | 1 | 2 | 4 | 1 | 1 | 1 | 1 | 1 | 1 | 1 | 1 |
| 2854 | 56 | 2 | 164 | 55 | 8 | 2 | 1 | 1 | 4 | 2 | 1 | 1 | 1 | 1 | 1 | 1 | 1 | 1 |
| 2855 | 56 | 2 | 164 | 53 | 4 | 2 | 1 | 1 | 4 | 1 | 1 | 1 | 1 | 1 | 1 | 1 | 1 | 1 |
| 2856 | 56 | 2 | 165 | 71 | 8 | 2 | 1 | 1 | 4 | 1 | 1 | 1 | 1 | 1 | 2 | 1 | 1 | 1 |
| 2857 | 56 | 2 | 165 | 65 | 8 | 2 | 1 | 1 | 4 | 2 | 1 | 1 | 1 | 1 | 1 | 1 | 1 | 1 |
| 2858 | 56 | 2 | 168 | 63 | 4 | 2 | 1 | 1 | 4 | 3 | 1 | 1 | 1 | 1 | 1 | 1 | 1 | 1 |
| 2859 | 56 | 2 | 168 | 60 | 8 | 2 | 1 | 1 | 4 | 1 | 1 | 1 | 2 | 1 | 1 | 1 | 1 | 1 |
| 2860 | 56 | 2 | 174 | 65 | 3 | 2 | 1 | 1 | 6 | 3 | 1 | 1 | 1 | 1 | 2 | 1 | 1 | 1 |
| 2861 | 56 | 1 | 147 | 60.5 | 4 | 2 | 1 | 1 | 3 | 1 | 1 | 1 | 1 | 1 | 1 | 1 | 1 | 1 |
| 2862 | 56 | 1 | 158.2 | 53 | 8 | 3 | 1 | 1 | 3 | 1 | 1 | 1 | 1 | 1 | 1 | 1 | 1 | 1 |
| 2863 | 56 | 1 | 160 | 90 | 6 | 2 | 1 | 2 | 2 | 2 | 1 | 1 | 1 | 1 | 1 | 1 | 3 | 1 |
| 2864 | 56 | 1 | 160 | 58.6 | 8 | 4 | 1 | 1 | 4 | 1 | 1 | 1 | 1 | 1 | 1 | 1 | 3 | 1 |
| 2865 | 56 | 1 | 161 | 59 | 6 | 2 | 1 | 2 | 4 | 1 | 1 | 1 | 1 | 1 | 1 | 1 | 3 | 1 |
| 2866 | 56 | 1 | 162 | 65 | 2 | 2 | 1 | 1 | 4 | 1 | 1 | 1 | 1 | 1 | 1 | 1 | 1 | 1 |
| 2867 | 56 | 1 | 162 | 58 | 8 | 2 | 1 | 1 | 4 | 2 | 1 | 1 | 1 | 1 | 1 | 2 | 3 | 1 |
| 2868 | 56 | 1 | 163 | 57 | 8 | 2 | 1 | 1 | 3 | 4 | 1 | 1 | 2 | 2 | 1 | 1 | 2 | 2 |
| 2869 | 56 | 1 | 164 | 66 | 8 | 2 | 1 | 1 | 4 | 1 | 1 | 1 | 1 | 1 | 1 | 1 | 2 | 1 |
| 2870 | 56 | 1 | 165 | 65 | 8 | 2 | 1 | 3 | 3 | 4 | 1 | 1 | 1 | 1 | 2 | 2 | 3 | 1 |
| 2871 | 56 | 1 | 165 | 61.1 | 3 | 2 | 1 | 1 | 4 | 1 | 1 | 1 | 1 | 1 | 1 | 1 | 3 | 2 |
| 2872 | 56 | 1 | 165 | 58.5 | 2 | 2 | 1 | 1 | 4 | 2 | 1 | 1 | 1 | 1 | 1 | 1 | 1 | 1 |
| 2873 | 56 | 1 | 166 | 62.5 | 8 | 2 | 1 | 1 | 4 | 1 | 1 | 1 | 2 | 1 | 1 | 1 | 2 | 1 |
| 2874 | 56 | 1 | 166 | 60 | 8 | 2 | 1 | 1 | 3 | 3 | 1 | 1 | 1 | 1 | 1 | 1 | 1 | 2 |
| 2875 | 56 | 1 | 167 | 65 | 8 | 2 | 1 | 1 | 4 | 4 | 1 | 1 | 2 | 1 | 2 | 1 | 2 | 1 |
| 2876 | 56 | 1 | 167 | 64 | 8 | 2 | 1 | 1 | 5 | 3 | 1 | 1 | 1 | 1 | 1 | 1 | 3 | 1 |
| 2877 | 56 | 1 | 168 | 76 | 4 | 2 | 1 | 2 | 4 | 4 | 1 | 1 | 1 | 1 | 1 | 1 | 3 | 1 |
| 2878 | 56 | 1 | 168 | 73 | 8 | 2 | 1 | 1 | 4 | 3 | 1 | 1 | 1 | 1 | 1 | 1 | 1 | 1 |
| 2879 | 56 | 1 | 168 | 72 | 8 | 2 | 1 | 2 | 4 | 2 | 1 | 1 | 1 | 1 | 2 | 1 | 3 | 1 |
| 2880 | 56 | 1 | 168 | 70 | 5 | 2 | 1 | 1 | 3 | 1 | 1 | 1 | 1 | 1 | 1 | 1 | 1 | 1 |
| 2881 | 56 | 1 | 168 | 65 | 8 | 2 | 1 | 1 | 2 | 4 | 1 | 1 | 1 | 1 | 1 | 1 | 3 | 1 |
| 2882 | 56 | 1 | 168 | 60 | 8 | 2 | 1 | 2 | 4 | 3 | 1 | 1 | 1 | 1 | 1 | 1 | 2 | 1 |
| 2883 | 56 | 1 | 168 | 55 | 8 | 2 | 1 | 1 | 4 | 4 | 1 | 1 | 1 | 1 | 1 | 1 | 1 | 1 |
| 2884 | 56 | 1 | 168.9 | 64 | 8 | 2 | 1 | 1 | 5 | 4 | 1 | 1 | 1 | 1 | 1 | 1 | 1 | 1 |
| 2885 | 56 | 1 | 169 | 56 | 8 | 2 | 1 | 1 | 3 | 1 | 1 | 1 | 1 | 1 | 1 | 1 | 1 | 1 |
| 2886 | 56 | 1 | 170 | 65 | 2 | 2 | 1 | 1 | 4 | 2 | 1 | 1 | 1 | 1 | 1 | 1 | 1 | 1 |
| 2887 | 56 | 1 | 171 | 104 | 8 | 2 | 1 | 1 | 6 | 1 | 1 | 1 | 1 | 1 | 1 | 1 | 1 | 1 |
| 2888 | 56 | 1 | 172 | 74 | 3 | 2 | 1 | 1 | 5 | 4 | 1 | 1 | 1 | 1 | 1 | 1 | 1 | 1 |
| 2889 | 56 | 1 | 173 | 66 | 8 | 2 | 1 | 1 | 3 | 1 | 1 | 1 | 2 | 1 | 1 | 1 | 1 | 1 |
| 2890 | 56 | 1 | 173 | 64 | 4 | 2 | 1 | 1 | 4 | 1 | 1 | 1 | 1 | 1 | 1 | 1 | 3 | 1 |
| 2891 | 56 | 1 | 173 | 57.6 | 2 | 2 | 1 | 3 | 3 | 4 | 1 | 1 | 1 | 1 | 1 | 1 | 3 | 1 |
| 2892 | 56 | 1 | 174 | 90 | 8 | 2 | 1 | 1 | 4 | 3 | 1 | 1 | 1 | 1 | 1 | 1 | 2 | 1 |
| 2893 | 56 | 1 | 174 | 62 | 4 | 2 | 1 | 1 | 3 | 2 | 3 | 1 | 1 | 2 | 1 | 1 | 1 | 2 |
| 2894 | 56 | 1 | 175 | 90 | 8 | 2 | 1 | 2 | 4 | 3 | 1 | 1 | 1 | 1 | 1 | 1 | 1 | 1 |
| 2895 | 56 | 1 | 175 | 82 | 8 | 4 | 1 | 2 | 4 | 2 | 1 | 1 | 1 | 1 | 1 | 1 | 2 | 1 |
| 2896 | 56 | 1 | 176 | 76 | 2 | 2 | 1 | 2 | 5 | 1 | 1 | 1 | 2 | 1 | 1 | 1 | 3 | 1 |
| 2897 | 57 | 2 | 126 | 37 | 8 | 1 | 1 | 1 | 3 | 4 | 1 | 1 | 1 | 1 | 1 | 1 | 1 | 1 |
| 2898 | 57 | 2 | 143 | 50 | 8 | 2 | 1 | 1 | 4 | 3 | 1 | 1 | 2 | 1 | 1 | 1 | 1 | 1 |
| 2899 | 57 | 2 | 146 | 59.5 | 8 | 2 | 2 | 1 | 3 | 2 | 1 | 1 | 1 | 1 | 1 | 1 | 1 | 1 |
| 2900 | 57 | 2 | 146 | 46.4 | 1 | 2 | 1 | 1 | 4 | 1 | 1 | 1 | 1 | 1 | 2 | 1 | 1 | 1 |
| 2901 | 57 | 2 | 147 | 52.5 | 2 | 2 | 1 | 1 | 3 | 1 | 1 | 1 | 1 | 1 | 1 | 2 | 1 | 1 |
| 2902 | 57 | 2 | 149 | 46.4 | 8 | 2 | 1 | 1 | 3 | 1 | 1 | 1 | 1 | 1 | 1 | 1 | 1 | 1 |
| 2903 | 57 | 2 | 150 | 57 | 4 | 2 | 1 | 1 | 3 | 4 | 1 | 1 | 2 | 1 | 2 | 1 | 1 | 1 |
| 2904 | 57 | 2 | 150 | 56 | 8 | 2 | 1 | 1 | 4 | 2 | 1 | 1 | 1 | 1 | 1 | 1 | 1 | 1 |
| 2905 | 57 | 2 | 150 | 53 | 1 | 2 | 1 | 1 | 2 | 3 | 1 | 1 | 2 | 1 | 1 | 1 | 1 | 1 |
| 2906 | 57 | 2 | 150 | 52 | 8 | 2 | 1 | 1 | 2 | 1 | 1 | 1 | 1 | 1 | 1 | 1 | 1 | 1 |
| 2907 | 57 | 2 | 150 | 51 | 8 | 2 | 1 | 1 | 6 | 3 | 1 | 1 | 1 | 1 | 1 | 1 | 1 | 1 |
| 2908 | 57 | 2 | 150 | 50 | 8 | 2 | 1 | 1 | 3 | 4 | 1 | 1 | 1 | 1 | 1 | 1 | 1 | 2 |
| 2909 | 57 | 2 | 150 | 50 | 4 | 2 | 1 | 1 | 4 | 3 | 1 | 1 | 1 | 1 | 1 | 1 | 1 | 1 |
| 2910 | 57 | 2 | 150 | 46 | 6 | 2 | 1 | 1 | 3 | 1 | 1 | 1 | 1 | 1 | 1 | 1 | 1 | 1 |
| 2911 | 57 | 2 | 150 | 41 | 8 | 2 | 1 | 1 | 2 | 4 | 1 | 1 | 1 | 1 | 1 | 1 | 1 | 1 |
| 2912 | 57 | 2 | 151 | 52 | 4 | 2 | 1 | 1 | 3 | 1 | 1 | 1 | 1 | 1 | 1 | 1 | 1 | 1 |
| 2913 | 57 | 2 | 151.5 | 78 | 6 | 2 | 1 | 1 | 2 | 1 | 1 | 1 | 1 | 1 | 1 | 1 | 1 | 1 |
| 2914 | 57 | 2 | 152 | 67.4 | 8 | 2 | 1 | 1 | 3 | 1 | 1 | 1 | 1 | 1 | 2 | 1 | 1 | 1 |
| 2915 | 57 | 2 | 152 | 55 | 8 | 2 | 1 | 1 | 3 | 4 | 1 | 1 | 1 | 1 | 1 | 1 | 1 | 2 |
| 2916 | 57 | 2 | 152 | 55 | 8 | 2 | 1 | 1 | 3 | 4 | 1 | 1 | 1 | 1 | 1 | 1 | 1 | 1 |
| 2917 | 57 | 2 | 152 | 53.7 | 8 | 2 | 1 | 1 | 4 | 1 | 1 | 1 | 1 | 1 | 1 | 1 | 1 | 1 |
| 2918 | 57 | 2 | 152 | 53 | 8 | 2 | 1 | 1 | 3 | 4 | 1 | 1 | 1 | 1 | 1 | 1 | 1 | 1 |
| 2919 | 57 | 2 | 152 | 51 | 8 | 2 | 1 | 1 | 4 | 3 | 1 | 1 | 1 | 1 | 1 | 1 | 1 | 1 |
| 2920 | 57 | 2 | 152 | 50 | 8 | 2 | 1 | 1 | 4 | 4 | 1 | 1 | 1 | 1 | 1 | 1 | 1 | 1 |
| 2921 | 57 | 2 | 152 | 50 | 1 | 2 | 1 | 1 | 4 | 3 | 1 | 1 | 1 | 1 | 1 | 1 | 1 | 1 |
| 2922 | 57 | 2 | 152 | 48 | 8 | 2 | 1 | 1 | 3 | 3 | 1 | 1 | 1 | 1 | 1 | 1 | 1 | 1 |
| 2923 | 57 | 2 | 152 | 47 | 8 | 3 | 2 | 1 | 3 | 4 | 1 | 1 | 1 | 1 | 1 | 1 | 1 | 1 |
| 2924 | 57 | 2 | 153 | 68.5 | 8 | 2 | 1 | 1 | 3 | 2 | 2 | 1 | 1 | 1 | 1 | 1 | 1 | 2 |
| 2925 | 57 | 2 | 153 | 51 | 8 | 2 | 1 | 1 | 3 | 3 | 1 | 1 | 1 | 1 | 1 | 1 | 1 | 1 |
| 2926 | 57 | 2 | 153 | 51 | 8 | 2 | 1 | 1 | 3 | 2 | 1 | 1 | 1 | 1 | 1 | 1 | 1 | 1 |
| 2927 | 57 | 2 | 153 | 49 | 8 | 2 | 1 | 1 | 3 | 1 | 1 | 1 | 1 | 1 | 1 | 1 | 1 | 1 |
| 2928 | 57 | 2 | 153 | 47 | 6 | 2 | 1 | 1 | 3 | 1 | 1 | 1 | 1 | 1 | 1 | 1 | 1 | 1 |
| 2929 | 57 | 2 | 154 | 80 | 4 | 2 | 1 | 1 | 3 | 1 | 1 | 1 | 1 | 1 | 1 | 1 | 1 | 1 |
| 2930 | 57 | 2 | 154 | 53 | 8 | 2 | 2 | 1 | 4 | 2 | 1 | 1 | 2 | 1 | 1 | 1 | 1 | 1 |
| 2931 | 57 | 2 | 154 | 53 | 4 | 2 | 1 | 1 | 3 | 1 | 1 | 1 | 2 | 1 | 2 | 1 | 1 | 1 |
| 2932 | 57 | 2 | 154 | 51 | 3 | 2 | 1 | 1 | 4 | 4 | 1 | 1 | 1 | 1 | 1 | 1 | 1 | 1 |
| 2933 | 57 | 2 | 155 | 91.4 | 6 | 2 | 1 | 1 | 3 | 2 | 1 | 1 | 1 | 1 | 2 | 1 | 1 | 1 |
| 2934 | 57 | 2 | 155 | 51 | 8 | 3 | 1 | 1 | 3 | 4 | 1 | 1 | 1 | 1 | 1 | 1 | 1 | 1 |
| 2935 | 57 | 2 | 155 | 50 | 8 | 2 | 1 | 1 | 4 | 4 | 1 | 1 | 1 | 1 | 1 | 1 | 1 | 2 |
| 2936 | 57 | 2 | 155 | 50 | 8 | 2 | 1 | 1 | 4 | 1 | 1 | 1 | 1 | 1 | 1 | 1 | 1 | 1 |
| 2937 | 57 | 2 | 155 | 49.5 | 8 | 1 | 1 | 1 | 3 | 1 | 1 | 1 | 1 | 1 | 1 | 1 | 1 | 1 |
| 2938 | 57 | 2 | 155 | 45 | 8 | 2 | 1 | 1 | 3 | 1 | 1 | 1 | 1 | 1 | 2 | 1 | 1 | 1 |
| 2939 | 57 | 2 | 155.5 | 80 | 8 | 2 | 1 | 1 | 4 | 3 | 1 | 1 | 1 | 1 | 1 | 1 | 1 | 1 |
| 2940 | 57 | 2 | 155.5 | 61.2 | 8 | 2 | 1 | 1 | 4 | 2 | 1 | 1 | 1 | 2 | 2 | 1 | 1 | 2 |
| 2941 | 57 | 2 | 156 | 72 | 8 | 2 | 1 | 1 | 4 | 3 | 1 | 1 | 1 | 1 | 1 | 1 | 1 | 1 |
| 2942 | 57 | 2 | 156 | 65 | 8 | 2 | 1 | 1 | 4 | 2 | 1 | 1 | 1 | 1 | 1 | 1 | 1 | 1 |
| 2943 | 57 | 2 | 156 | 61 | 4 | 2 | 1 | 1 | 1 | 4 | 1 | 1 | 1 | 1 | 2 | 1 | 1 | 1 |
| 2944 | 57 | 2 | 156 | 60 | 8 | 2 | 1 | 1 | 4 | 1 | 1 | 1 | 1 | 1 | 2 | 1 | 1 | 1 |
| 2945 | 57 | 2 | 156 | 60 | 8 | 2 | 2 | 1 | 4 | 1 | 1 | 1 | 1 | 1 | 1 | 1 | 1 | 1 |
| 2946 | 57 | 2 | 156 | 60 | 6 | 2 | 1 | 1 | 4 | 3 | 1 | 1 | 1 | 1 | 2 | 2 | 1 | 1 |
| 2947 | 57 | 2 | 156 | 60 | 1 | 2 | 1 | 1 | 4 | 1 | 1 | 1 | 2 | 1 | 2 | 1 | 1 | 1 |
| 2948 | 57 | 2 | 156 | 59.7 | 2 | 2 | 1 | 1 | 4 | 4 | 1 | 1 | 2 | 1 | 1 | 1 | 1 | 1 |
| 2949 | 57 | 2 | 156 | 57.8 | 3 | 1 | 1 | 1 | 4 | 2 | 1 | 1 | 1 | 1 | 1 | 1 | 1 | 1 |
| 2950 | 57 | 2 | 156 | 54 | 8 | 2 | 1 | 1 | 4 | 4 | 1 | 1 | 1 | 1 | 1 | 1 | 1 | 1 |
| 2951 | 57 | 2 | 156 | 52 | 8 | 2 | 1 | 1 | 4 | 3 | 1 | 1 | 1 | 1 | 1 | 1 | 1 | 1 |
| 2952 | 57 | 2 | 156 | 52 | 2 | 2 | 1 | 1 | 4 | 1 | 1 | 1 | 1 | 1 | 1 | 1 | 1 | 1 |
| 2953 | 57 | 2 | 156 | 50 | 8 | 2 | 1 | 1 | 4 | 3 | 1 | 1 | 1 | 1 | 1 | 1 | 1 | 1 |
| 2954 | 57 | 2 | 156 | 44.8 | 6 | 2 | 1 | 1 | 3 | 1 | 1 | 1 | 1 | 1 | 1 | 1 | 1 | 1 |
| 2955 | 57 | 2 | 156.5 | 73.4 | 8 | 2 | 1 | 1 | 4 | 2 | 1 | 1 | 2 | 1 | 1 | 1 | 1 | 1 |
| 2956 | 57 | 2 | 157 | 67 | 8 | 2 | 1 | 1 | 4 | 3 | 1 | 1 | 2 | 1 | 1 | 1 | 1 | 1 |
| 2957 | 57 | 2 | 157 | 66.2 | 8 | 2 | 1 | 1 | 4 | 3 | 1 | 1 | 1 | 1 | 2 | 1 | 1 | 1 |
| 2958 | 57 | 2 | 157 | 62 | 8 | 2 | 1 | 1 | 4 | 4 | 1 | 1 | 2 | 1 | 1 | 1 | 1 | 1 |
| 2959 | 57 | 2 | 157 | 61 | 8 | 2 | 2 | 1 | 6 | 4 | 1 | 1 | 1 | 1 | 1 | 1 | 1 | 1 |
| 2960 | 57 | 2 | 157 | 58 | 4 | 2 | 1 | 1 | 5 | 3 | 1 | 1 | 1 | 2 | 2 | 1 | 1 | 2 |
| 2961 | 57 | 2 | 157 | 42 | 8 | 2 | 1 | 1 | 4 | 3 | 1 | 1 | 2 | 1 | 1 | 1 | 1 | 1 |
| 2962 | 57 | 2 | 157 | 40 | 8 | 3 | 1 | 1 | 4 | 4 | 1 | 1 | 1 | 1 | 1 | 1 | 1 | 1 |
| 2963 | 57 | 2 | 158 | 80 | 8 | 2 | 2 | 1 | 4 | 3 | 1 | 1 | 1 | 1 | 1 | 1 | 1 | 1 |
| 2964 | 57 | 2 | 158 | 63 | 8 | 2 | 1 | 1 | 4 | 1 | 1 | 1 | 1 | 1 | 1 | 1 | 1 | 1 |
| 2965 | 57 | 2 | 158 | 63 | 8 | 2 | 1 | 1 | 1 | 4 | 1 | 1 | 1 | 1 | 1 | 1 | 1 | 1 |
| 2966 | 57 | 2 | 158 | 60 | 8 | 2 | 1 | 1 | 4 | 4 | 1 | 1 | 1 | 1 | 1 | 1 | 1 | 1 |
| 2967 | 57 | 2 | 158 | 60 | 3 | 2 | 1 | 1 | 3 | 1 | 1 | 1 | 1 | 1 | 1 | 1 | 1 | 1 |
| 2968 | 57 | 2 | 158 | 59 | 8 | 2 | 1 | 1 | 4 | 1 | 1 | 1 | 1 | 1 | 1 | 1 | 1 | 1 |
| 2969 | 57 | 2 | 158 | 57 | 8 | 2 | 1 | 1 | 4 | 4 | 1 | 1 | 1 | 1 | 1 | 1 | 1 | 1 |
| 2970 | 57 | 2 | 158 | 55 | 8 | 2 | 1 | 1 | 4 | 3 | 1 | 1 | 1 | 1 | 1 | 1 | 1 | 1 |
| 2971 | 57 | 2 | 158 | 48 | 8 | 2 | 1 | 1 | 3 | 1 | 1 | 1 | 1 | 1 | 1 | 1 | 1 | 1 |
| 2972 | 57 | 2 | 159 | 61 | 2 | 2 | 1 | 2 | 4 | 2 | 1 | 1 | 1 | 1 | 1 | 1 | 1 | 1 |
| 2973 | 57 | 2 | 159 | 60 | 8 | 2 | 1 | 1 | 5 | 1 | 3 | 1 | 2 | 1 | 2 | 1 | 1 | 1 |
| 2974 | 57 | 2 | 159 | 56 | 4 | 2 | 1 | 4 | 4 | 1 | 1 | 1 | 1 | 1 | 1 | 1 | 1 | 2 |
| 2975 | 57 | 2 | 159 | 52.4 | 6 | 2 | 1 | 1 | 4 | 2 | 1 | 1 | 1 | 1 | 1 | 1 | 1 | 2 |
| 2976 | 57 | 2 | 159 | 44.6 | 8 | 4 | 1 | 1 | 3 | 1 | 1 | 1 | 1 | 1 | 2 | 1 | 3 | 1 |
| 2977 | 57 | 2 | 159.2 | 63.5 | 6 | 2 | 1 | 1 | 4 | 1 | 1 | 1 | 1 | 1 | 1 | 1 | 1 | 1 |
| 2978 | 57 | 2 | 160 | 72 | 8 | 2 | 1 | 1 | 6 | 3 | 1 | 1 | 1 | 1 | 1 | 1 | 1 | 1 |
| 2979 | 57 | 2 | 160 | 67.5 | 6 | 2 | 1 | 1 | 4 | 1 | 1 | 1 | 1 | 1 | 1 | 1 | 1 | 1 |
| 2980 | 57 | 2 | 160 | 60 | 8 | 2 | 1 | 1 | 4 | 3 | 1 | 1 | 1 | 1 | 1 | 1 | 1 | 1 |
| 2981 | 57 | 2 | 160 | 60 | 4 | 2 | 1 | 1 | 3 | 1 | 1 | 1 | 1 | 1 | 2 | 1 | 1 | 1 |
| 2982 | 57 | 2 | 160 | 56 | 8 | 2 | 1 | 1 | 3 | 4 | 1 | 1 | 1 | 1 | 1 | 1 | 1 | 1 |
| 2983 | 57 | 2 | 160 | 56 | 4 | 2 | 1 | 1 | 3 | 4 | 1 | 1 | 1 | 1 | 1 | 1 | 1 | 1 |
| 2984 | 57 | 2 | 160 | 55 | 8 | 2 | 1 | 1 | 5 | 4 | 1 | 1 | 1 | 1 | 1 | 1 | 1 | 1 |
| 2985 | 57 | 2 | 160 | 55 | 8 | 2 | 1 | 1 | 5 | 1 | 1 | 1 | 1 | 1 | 1 | 1 | 1 | 1 |
| 2986 | 57 | 2 | 160 | 51 | 8 | 2 | 1 | 1 | 5 | 2 | 1 | 1 | 1 | 1 | 1 | 1 | 1 | 1 |
| 2987 | 57 | 2 | 160 | 51 | 8 | 2 | 1 | 1 | 4 | 2 | 1 | 1 | 2 | 1 | 1 | 1 | 1 | 1 |
| 2988 | 57 | 2 | 160 | 51 | 6 | 2 | 1 | 1 | 3 | 4 | 1 | 1 | 1 | 1 | 1 | 1 | 1 | 1 |
| 2989 | 57 | 2 | 160.5 | 57.8 | 8 | 2 | 1 | 1 | 4 | 1 | 1 | 1 | 1 | 1 | 2 | 1 | 1 | 2 |
| 2990 | 57 | 2 | 161 | 71 | 8 | 2 | 1 | 1 | 3 | 3 | 1 | 1 | 1 | 1 | 1 | 1 | 1 | 1 |
| 2991 | 57 | 2 | 161.5 | 51 | 8 | 2 | 1 | 1 | 3 | 2 | 1 | 1 | 1 | 1 | 2 | 1 | 1 | 1 |
| 2992 | 57 | 2 | 161.5 | 41.6 | 2 | 2 | 1 | 1 | 3 | 2 | 1 | 1 | 1 | 1 | 1 | 1 | 1 | 1 |
| 2993 | 57 | 2 | 162 | 70 | 8 | 2 | 1 | 1 | 5 | 1 | 1 | 1 | 1 | 1 | 1 | 1 | 1 | 1 |
| 2994 | 57 | 2 | 162 | 65 | 8 | 2 | 1 | 1 | 4 | 4 | 1 | 1 | 1 | 1 | 1 | 1 | 1 | 1 |
| 2995 | 57 | 2 | 163 | 75 | 8 | 2 | 1 | 1 | 3 | 4 | 1 | 1 | 1 | 1 | 1 | 1 | 1 | 1 |
| 2996 | 57 | 2 | 163 | 54 | 8 | 2 | 1 | 1 | 2 | 2 | 1 | 1 | 1 | 1 | 1 | 1 | 1 | 1 |
| 2997 | 57 | 2 | 163 | 50 | 2 | 4 | 1 | 1 | 4 | 4 | 1 | 1 | 1 | 1 | 1 | 1 | 1 | 1 |
| 2998 | 57 | 2 | 164.5 | 65.6 | 3 | 1 | 1 | 1 | 6 | 3 | 1 | 1 | 1 | 1 | 1 | 1 | 1 | 1 |
| 2999 | 57 | 2 | 165 | 78.5 | 8 | 2 | 1 | 1 | 6 | 4 | 1 | 1 | 1 | 1 | 1 | 1 | 1 | 1 |
| 3000 | 57 | 2 | 165 | 65 | 8 | 2 | 1 | 1 | 4 | 1 | 1 | 1 | 1 | 1 | 1 | 1 | 1 | 1 |
| 3001 | 57 | 2 | 165 | 55.5 | 4 | 2 | 1 | 1 | 4 | 2 | 1 | 1 | 1 | 1 | 2 | 2 | 1 | 1 |
| 3002 | 57 | 2 | 167 | 71 | 8 | 2 | 1 | 1 | 4 | 2 | 1 | 1 | 1 | 1 | 1 | 1 | 1 | 1 |
| 3003 | 57 | 2 | 167 | 55 | 8 | 2 | 1 | 2 | 4 | 4 | 1 | 1 | 1 | 1 | 2 | 1 | 1 | 1 |
| 3004 | 57 | 2 | 169 | 68 | 2 | 2 | 1 | 1 | 5 | 1 | 1 | 1 | 1 | 1 | 2 | 1 | 1 | 1 |
| 3005 | 57 | 2 | 174 | 73 | 3 | 2 | 1 | 1 | 5 | 1 | 1 | 2 | 1 | 1 | 2 | 1 | 1 | 1 |
| 3006 | 57 | 2 | 175 | 64 | 3 | 2 | 1 | 1 | 4 | 1 | 1 | 1 | 1 | 1 | 1 | 1 | 3 | 1 |
| 3007 | 57 | 1 | 155 | 62.4 | 8 | 2 | 1 | 1 | 3 | 4 | 1 | 1 | 1 | 1 | 1 | 1 | 1 | 1 |
| 3008 | 57 | 1 | 159 | 73 | 8 | 2 | 1 | 1 | 4 | 4 | 1 | 1 | 1 | 1 | 1 | 1 | 1 | 1 |
| 3009 | 57 | 1 | 159 | 54.5 | 8 | 3 | 1 | 1 | 2 | 2 | 1 | 1 | 2 | 1 | 2 | 1 | 1 | 1 |
| 3010 | 57 | 1 | 160 | 67.5 | 8 | 2 | 1 | 1 | 3 | 1 | 1 | 1 | 1 | 1 | 2 | 1 | 1 | 1 |
| 3011 | 57 | 1 | 160 | 65 | 8 | 2 | 1 | 1 | 3 | 2 | 1 | 1 | 1 | 1 | 1 | 1 | 1 | 1 |
| 3012 | 57 | 1 | 161 | 65 | 8 | 2 | 1 | 1 | 4 | 4 | 1 | 1 | 1 | 1 | 1 | 1 | 1 | 1 |
| 3013 | 57 | 1 | 162 | 79 | 8 | 2 | 1 | 1 | 3 | 3 | 1 | 2 | 1 | 1 | 2 | 1 | 1 | 1 |
| 3014 | 57 | 1 | 162 | 69.9 | 2 | 2 | 1 | 1 | 3 | 1 | 1 | 1 | 1 | 1 | 2 | 1 | 3 | 1 |
| 3015 | 57 | 1 | 163 | 62.5 | 8 | 2 | 1 | 1 | 4 | 4 | 1 | 1 | 1 | 1 | 1 | 1 | 1 | 1 |
| 3016 | 57 | 1 | 163 | 59 | 8 | 2 | 1 | 1 | 3 | 1 | 1 | 1 | 1 | 1 | 2 | 1 | 3 | 1 |
| 3017 | 57 | 1 | 164 | 62 | 8 | 2 | 1 | 1 | 5 | 4 | 1 | 1 | 1 | 1 | 1 | 1 | 1 | 1 |
| 3018 | 57 | 1 | 165 | 74.8 | 8 | 2 | 1 | 2 | 5 | 3 | 1 | 1 | 1 | 1 | 1 | 1 | 3 | 1 |
| 3019 | 57 | 1 | 166 | 72 | 8 | 2 | 1 | 1 | 4 | 1 | 1 | 1 | 2 | 1 | 1 | 1 | 3 | 1 |
| 3020 | 57 | 1 | 168 | 87 | 8 | 2 | 1 | 1 | 4 | 3 | 1 | 1 | 1 | 1 | 1 | 1 | 1 | 2 |
| 3021 | 57 | 1 | 168 | 83 | 6 | 2 | 1 | 2 | 3 | 3 | 1 | 1 | 1 | 1 | 1 | 1 | 3 | 1 |
| 3022 | 57 | 1 | 168 | 59 | 8 | 2 | 2 | 1 | 4 | 3 | 1 | 1 | 1 | 1 | 1 | 1 | 3 | 1 |
| 3023 | 57 | 1 | 168 | 59 | 3 | 2 | 1 | 1 | 5 | 3 | 1 | 1 | 1 | 1 | 1 | 1 | 1 | 1 |
| 3024 | 57 | 1 | 168 | 55.5 | 8 | 2 | 1 | 2 | 4 | 1 | 1 | 1 | 2 | 1 | 2 | 1 | 3 | 1 |
| 3025 | 57 | 1 | 168 | 55 | 8 | 2 | 1 | 2 | 3 | 4 | 1 | 1 | 2 | 1 | 1 | 1 | 3 | 2 |
| 3026 | 57 | 1 | 168 | 55 | 8 | 2 | 1 | 2 | 3 | 4 | 1 | 1 | 1 | 1 | 1 | 1 | 3 | 1 |
| 3027 | 57 | 1 | 169 | 73 | 8 | 2 | 1 | 1 | 4 | 1 | 1 | 1 | 1 | 1 | 1 | 1 | 1 | 1 |
| 3028 | 57 | 1 | 169 | 64 | 8 | 2 | 1 | 4 | 4 | 4 | 1 | 1 | 1 | 1 | 1 | 1 | 3 | 1 |
| 3029 | 57 | 1 | 170 | 74 | 3 | 2 | 1 | 1 | 4 | 2 | 1 | 1 | 1 | 1 | 2 | 1 | 3 | 1 |
| 3030 | 57 | 1 | 170 | 70.5 | 3 | 2 | 1 | 4 | 3 | 4 | 1 | 1 | 1 | 1 | 1 | 1 | 3 | 1 |
| 3031 | 57 | 1 | 170 | 70 | 8 | 2 | 1 | 1 | 4 | 4 | 1 | 1 | 1 | 1 | 1 | 1 | 1 | 1 |
| 3032 | 57 | 1 | 170 | 68 | 4 | 2 | 1 | 1 | 4 | 3 | 1 | 1 | 1 | 2 | 1 | 1 | 1 | 2 |
| 3033 | 57 | 1 | 170 | 68 | 2 | 2 | 1 | 1 | 3 | 4 | 1 | 1 | 1 | 1 | 1 | 1 | 3 | 1 |
| 3034 | 57 | 1 | 170 | 52 | 5 | 2 | 1 | 1 | 4 | 1 | 1 | 1 | 1 | 1 | 2 | 1 | 1 | 1 |
| 3035 | 57 | 1 | 171 | 82 | 8 | 2 | 1 | 1 | 4 | 1 | 1 | 1 | 1 | 1 | 2 | 1 | 3 | 1 |
| 3036 | 57 | 1 | 172 | 62.5 | 8 | 1 | 1 | 1 | 6 | 4 | 1 | 1 | 1 | 1 | 1 | 1 | 1 | 1 |
| 3037 | 57 | 1 | 173 | 80.5 | 8 | 2 | 1 | 3 | 4 | 2 | 1 | 1 | 1 | 1 | 1 | 1 | 3 | 1 |
| 3038 | 57 | 1 | 175 | 68 | 6 | 3 | 1 | 2 | 4 | 3 | 1 | 1 | 2 | 1 | 1 | 1 | 3 | 1 |
| 3039 | 57 | 1 | 180 | 69.2 | 2 | 1 | 1 | 4 | 3 | 1 | 1 | 1 | 1 | 1 | 1 | 1 | 3 | 1 |
| 3040 | 57 | 1 | 181 | 87.5 | 4 | 2 | 1 | 1 | 5 | 2 | 1 | 1 | 2 | 1 | 1 | 1 | 1 | 1 |
| 3041 | 58 | 2 | 144 | 45 | 6 | 2 | 1 | 1 | 4 | 1 | 1 | 1 | 1 | 1 | 2 | 1 | 1 | 1 |
| 3042 | 58 | 2 | 144.5 | 48.6 | 6 | 4 | 1 | 1 | 3 | 1 | 1 | 1 | 1 | 1 | 1 | 1 | 1 | 1 |
| 3043 | 58 | 2 | 147.5 | 69.5 | 8 | 2 | 1 | 1 | 5 | 1 | 1 | 1 | 1 | 1 | 1 | 1 | 1 | 1 |
| 3044 | 58 | 2 | 149 | 52 | 6 | 2 | 1 | 1 | 3 | 1 | 1 | 1 | 1 | 1 | 2 | 1 | 1 | 1 |
| 3045 | 58 | 2 | 149 | 48 | 8 | 2 | 1 | 1 | 4 | 4 | 1 | 1 | 1 | 1 | 1 | 1 | 1 | 1 |
| 3046 | 58 | 2 | 149 | 45 | 4 | 2 | 1 | 1 | 4 | 2 | 1 | 1 | 1 | 1 | 1 | 1 | 1 | 1 |
| 3047 | 58 | 2 | 150 | 67 | 8 | 2 | 1 | 1 | 4 | 3 | 1 | 1 | 1 | 1 | 1 | 1 | 1 | 1 |
| 3048 | 58 | 2 | 150 | 63.5 | 8 | 2 | 1 | 1 | 4 | 2 | 1 | 1 | 2 | 1 | 2 | 1 | 1 | 1 |
| 3049 | 58 | 2 | 150 | 61 | 8 | 2 | 1 | 1 | 3 | 2 | 1 | 1 | 1 | 1 | 1 | 1 | 1 | 1 |
| 3050 | 58 | 2 | 150 | 60.3 | 8 | 1 | 1 | 1 | 4 | 1 | 1 | 1 | 1 | 1 | 1 | 1 | 1 | 1 |
| 3051 | 58 | 2 | 150 | 60 | 8 | 2 | 1 | 1 | 4 | 1 | 1 | 1 | 1 | 1 | 2 | 1 | 1 | 1 |
| 3052 | 58 | 2 | 150 | 60 | 4 | 4 | 1 | 2 | 4 | 1 | 1 | 1 | 1 | 1 | 1 | 1 | 1 | 1 |
| 3053 | 58 | 2 | 150 | 60 | 1 | 2 | 1 | 1 | 3 | 1 | 1 | 1 | 1 | 1 | 2 | 1 | 1 | 1 |
| 3054 | 58 | 2 | 150 | 55 | 8 | 2 | 1 | 1 | 2 | 1 | 1 | 1 | 1 | 1 | 2 | 1 | 1 | 1 |
| 3055 | 58 | 2 | 150 | 54 | 4 | 2 | 2 | 1 | 2 | 4 | 1 | 1 | 1 | 1 | 1 | 1 | 1 | 1 |
| 3056 | 58 | 2 | 150 | 48.5 | 8 | 1 | 1 | 2 | 3 | 1 | 1 | 1 | 1 | 1 | 1 | 1 | 1 | 1 |
| 3057 | 58 | 2 | 150 | 33 | 6 | 3 | 1 | 1 | 4 | 1 | 1 | 1 | 1 | 1 | 1 | 1 | 1 | 2 |
| 3058 | 58 | 2 | 152 | 65 | 8 | 2 | 1 | 1 | 4 | 4 | 1 | 1 | 1 | 1 | 1 | 1 | 1 | 1 |
| 3059 | 58 | 2 | 152 | 62.1 | 4 | 2 | 1 | 3 | 4 | 1 | 1 | 1 | 1 | 1 | 1 | 1 | 1 | 1 |
| 3060 | 58 | 2 | 152 | 59 | 8 | 2 | 1 | 1 | 2 | 3 | 1 | 1 | 1 | 1 | 1 | 1 | 1 | 2 |
| 3061 | 58 | 2 | 152 | 59 | 6 | 2 | 1 | 1 | 3 | 2 | 1 | 1 | 2 | 1 | 1 | 1 | 1 | 1 |
| 3062 | 58 | 2 | 152 | 50 | 6 | 2 | 1 | 1 | 1 | 1 | 1 | 1 | 2 | 1 | 1 | 1 | 1 | 1 |
| 3063 | 58 | 2 | 152 | 48 | 8 | 3 | 1 | 1 | 3 | 1 | 1 | 1 | 1 | 1 | 1 | 1 | 1 | 1 |
| 3064 | 58 | 2 | 153 | 54 | 8 | 2 | 2 | 1 | 3 | 1 | 1 | 1 | 1 | 1 | 1 | 1 | 1 | 1 |
| 3065 | 58 | 2 | 153 | 49 | 8 | 2 | 1 | 1 | 4 | 4 | 1 | 1 | 1 | 1 | 1 | 1 | 1 | 1 |
| 3066 | 58 | 2 | 153 | 46.5 | 6 | 2 | 1 | 1 | 3 | 1 | 1 | 1 | 1 | 1 | 1 | 1 | 1 | 1 |
| 3067 | 58 | 2 | 154 | 55.5 | 8 | 2 | 1 | 1 | 3 | 1 | 1 | 1 | 2 | 1 | 1 | 1 | 1 | 1 |
| 3068 | 58 | 2 | 154 | 49 | 3 | 2 | 1 | 1 | 6 | 2 | 1 | 1 | 2 | 1 | 2 | 1 | 1 | 1 |
| 3069 | 58 | 2 | 154 | 46 | 8 | 3 | 1 | 1 | 3 | 1 | 1 | 1 | 1 | 1 | 1 | 1 | 1 | 1 |
| 3070 | 58 | 2 | 154 | 44 | 8 | 2 | 1 | 2 | 4 | 1 | 1 | 1 | 1 | 1 | 2 | 1 | 1 | 1 |
| 3071 | 58 | 2 | 154.2 | 62 | 3 | 2 | 2 | 1 | 3 | 2 | 1 | 1 | 1 | 1 | 2 | 1 | 1 | 1 |
| 3072 | 58 | 2 | 155 | 62 | 8 | 2 | 1 | 1 | 3 | 2 | 1 | 1 | 1 | 1 | 1 | 1 | 1 | 1 |
| 3073 | 58 | 2 | 155 | 55 | 8 | 3 | 1 | 1 | 3 | 3 | 1 | 1 | 1 | 1 | 1 | 1 | 1 | 1 |
| 3074 | 58 | 2 | 155 | 55 | 8 | 2 | 1 | 1 | 5 | 1 | 2 | 1 | 1 | 1 | 1 | 1 | 1 | 1 |
| 3075 | 58 | 2 | 155 | 54 | 4 | 2 | 1 | 1 | 4 | 4 | 1 | 1 | 1 | 1 | 1 | 1 | 1 | 1 |
| 3076 | 58 | 2 | 155 | 53 | 8 | 2 | 1 | 1 | 6 | 4 | 1 | 1 | 1 | 1 | 1 | 1 | 1 | 1 |
| 3077 | 58 | 2 | 155 | 52 | 4 | 2 | 2 | 1 | 4 | 3 | 1 | 1 | 1 | 1 | 1 | 1 | 1 | 1 |
| 3078 | 58 | 2 | 155 | 49 | 8 | 2 | 1 | 1 | 5 | 1 | 1 | 1 | 1 | 1 | 1 | 1 | 1 | 1 |
| 3079 | 58 | 2 | 155 | 46 | 8 | 2 | 1 | 1 | 4 | 1 | 1 | 1 | 1 | 1 | 2 | 1 | 1 | 1 |
| 3080 | 58 | 2 | 155 | 45 | 5 | 2 | 1 | 1 | 3 | 4 | 1 | 1 | 1 | 1 | 1 | 1 | 1 | 1 |
| 3081 | 58 | 2 | 156 | 68 | 3 | 2 | 1 | 1 | 3 | 2 | 1 | 1 | 1 | 1 | 2 | 1 | 1 | 1 |
| 3082 | 58 | 2 | 156 | 53 | 8 | 2 | 1 | 1 | 2 | 1 | 1 | 1 | 1 | 1 | 2 | 1 | 1 | 1 |
| 3083 | 58 | 2 | 156 | 50 | 8 | 2 | 1 | 2 | 3 | 2 | 1 | 1 | 1 | 1 | 1 | 1 | 1 | 1 |
| 3084 | 58 | 2 | 156 | 50 | 8 | 1 | 1 | 1 | 4 | 1 | 1 | 1 | 1 | 1 | 1 | 1 | 1 | 1 |
| 3085 | 58 | 2 | 156 | 50 | 4 | 1 | 1 | 1 | 4 | 1 | 1 | 1 | 1 | 1 | 1 | 1 | 1 | 1 |
| 3086 | 58 | 2 | 157 | 60 | 8 | 2 | 1 | 1 | 4 | 2 | 1 | 1 | 1 | 1 | 1 | 1 | 1 | 1 |
| 3087 | 58 | 2 | 157 | 58 | 4 | 2 | 1 | 1 | 4 | 2 | 1 | 1 | 2 | 1 | 2 | 1 | 1 | 1 |
| 3088 | 58 | 2 | 157 | 55 | 4 | 2 | 1 | 1 | 4 | 3 | 1 | 1 | 2 | 1 | 2 | 1 | 1 | 1 |
| 3089 | 58 | 2 | 157 | 47 | 8 | 2 | 1 | 1 | 3 | 1 | 1 | 1 | 1 | 1 | 1 | 1 | 1 | 1 |
| 3090 | 58 | 2 | 157 | 46.7 | 8 | 2 | 1 | 1 | 3 | 1 | 1 | 1 | 2 | 1 | 1 | 1 | 1 | 1 |
| 3091 | 58 | 2 | 157.5 | 60.9 | 3 | 2 | 1 | 2 | 2 | 1 | 1 | 1 | 2 | 1 | 1 | 1 | 1 | 1 |
| 3092 | 58 | 2 | 157.5 | 60.5 | 8 | 2 | 1 | 1 | 4 | 1 | 1 | 1 | 2 | 1 | 1 | 1 | 1 | 1 |
| 3093 | 58 | 2 | 158 | 68 | 6 | 4 | 1 | 1 | 4 | 4 | 1 | 1 | 1 | 1 | 1 | 1 | 1 | 1 |
| 3094 | 58 | 2 | 158 | 63 | 5 | 2 | 1 | 1 | 3 | 1 | 1 | 1 | 1 | 1 | 2 | 2 | 1 | 1 |
| 3095 | 58 | 2 | 158 | 63 | 4 | 2 | 1 | 1 | 4 | 1 | 1 | 1 | 1 | 1 | 1 | 1 | 1 | 1 |
| 3096 | 58 | 2 | 158 | 60 | 8 | 2 | 1 | 1 | 4 | 1 | 1 | 1 | 1 | 1 | 1 | 1 | 1 | 1 |
| 3097 | 58 | 2 | 158 | 60 | 3 | 2 | 1 | 1 | 4 | 4 | 1 | 1 | 1 | 1 | 1 | 1 | 1 | 1 |
| 3098 | 58 | 2 | 158 | 57 | 8 | 2 | 1 | 1 | 3 | 1 | 1 | 1 | 1 | 1 | 1 | 1 | 1 | 1 |
| 3099 | 58 | 2 | 158 | 52 | 3 | 2 | 1 | 1 | 4 | 1 | 1 | 1 | 1 | 2 | 1 | 1 | 1 | 1 |
| 3100 | 58 | 2 | 158 | 51.7 | 8 | 2 | 1 | 1 | 4 | 2 | 1 | 1 | 2 | 1 | 1 | 1 | 1 | 1 |
| 3101 | 58 | 2 | 158 | 50 | 4 | 2 | 1 | 1 | 5 | 4 | 1 | 1 | 1 | 1 | 1 | 1 | 1 | 1 |
| 3102 | 58 | 2 | 158 | 49 | 2 | 2 | 1 | 1 | 4 | 1 | 1 | 1 | 1 | 1 | 1 | 1 | 1 | 1 |
| 3103 | 58 | 2 | 158 | 45.1 | 8 | 2 | 1 | 1 | 4 | 1 | 1 | 1 | 1 | 1 | 1 | 1 | 1 | 1 |
| 3104 | 58 | 2 | 158.5 | 48 | 2 | 2 | 1 | 1 | 3 | 1 | 1 | 1 | 1 | 1 | 1 | 1 | 1 | 1 |
| 3105 | 58 | 2 | 159 | 58 | 1 | 3 | 1 | 2 | 4 | 1 | 1 | 1 | 1 | 1 | 2 | 2 | 1 | 2 |
| 3106 | 58 | 2 | 159 | 56 | 5 | 2 | 1 | 1 | 6 | 2 | 1 | 1 | 1 | 1 | 1 | 1 | 1 | 1 |
| 3107 | 58 | 2 | 159 | 55 | 8 | 2 | 1 | 1 | 4 | 4 | 1 | 1 | 1 | 1 | 1 | 1 | 1 | 1 |
| 3108 | 58 | 2 | 159 | 48.7 | 4 | 4 | 1 | 1 | 3 | 3 | 1 | 2 | 2 | 1 | 1 | 1 | 1 | 1 |
| 3109 | 58 | 2 | 160 | 75 | 8 | 3 | 1 | 1 | 3 | 4 | 1 | 1 | 1 | 1 | 1 | 1 | 1 | 1 |
| 3110 | 58 | 2 | 160 | 68 | 4 | 2 | 1 | 1 | 4 | 4 | 1 | 1 | 1 | 1 | 2 | 1 | 1 | 1 |
| 3111 | 58 | 2 | 160 | 61 | 8 | 2 | 1 | 1 | 4 | 1 | 1 | 1 | 1 | 1 | 1 | 1 | 1 | 1 |
| 3112 | 58 | 2 | 160 | 58 | 8 | 2 | 2 | 1 | 4 | 1 | 1 | 1 | 1 | 1 | 2 | 1 | 1 | 1 |
| 3113 | 58 | 2 | 160 | 58 | 8 | 2 | 1 | 1 | 4 | 1 | 1 | 1 | 1 | 1 | 1 | 1 | 1 | 1 |
| 3114 | 58 | 2 | 160 | 58 | 3 | 2 | 1 | 1 | 4 | 2 | 1 | 1 | 2 | 1 | 2 | 1 | 1 | 1 |
| 3115 | 58 | 2 | 160 | 57 | 8 | 2 | 1 | 1 | 3 | 3 | 1 | 1 | 1 | 1 | 1 | 1 | 1 | 2 |
| 3116 | 58 | 2 | 160 | 54 | 8 | 2 | 1 | 1 | 3 | 1 | 1 | 1 | 1 | 1 | 1 | 1 | 1 | 1 |
| 3117 | 58 | 2 | 160 | 52 | 4 | 2 | 1 | 1 | 5 | 3 | 1 | 1 | 1 | 1 | 2 | 1 | 1 | 1 |
| 3118 | 58 | 2 | 161 | 76.8 | 3 | 2 | 1 | 1 | 4 | 3 | 1 | 1 | 1 | 2 | 1 | 1 | 1 | 2 |
| 3119 | 58 | 2 | 161 | 61.5 | 4 | 2 | 1 | 1 | 3 | 3 | 1 | 1 | 1 | 1 | 1 | 1 | 1 | 1 |
| 3120 | 58 | 2 | 161 | 60.4 | 8 | 2 | 1 | 1 | 4 | 2 | 2 | 1 | 1 | 2 | 2 | 1 | 1 | 2 |
| 3121 | 58 | 2 | 161 | 47 | 2 | 2 | 1 | 1 | 4 | 1 | 1 | 1 | 1 | 1 | 2 | 1 | 1 | 1 |
| 3122 | 58 | 2 | 162 | 72 | 4 | 2 | 1 | 1 | 4 | 2 | 1 | 1 | 1 | 1 | 1 | 1 | 1 | 1 |
| 3123 | 58 | 2 | 162 | 65 | 8 | 2 | 1 | 1 | 3 | 3 | 1 | 1 | 1 | 1 | 1 | 1 | 1 | 1 |
| 3124 | 58 | 2 | 162 | 55 | 8 | 2 | 1 | 1 | 4 | 1 | 1 | 1 | 1 | 1 | 1 | 1 | 1 | 2 |
| 3125 | 58 | 2 | 162 | 53 | 8 | 2 | 1 | 1 | 4 | 2 | 1 | 1 | 1 | 1 | 1 | 1 | 1 | 1 |
| 3126 | 58 | 2 | 162 | 48.5 | 8 | 2 | 2 | 1 | 5 | 4 | 1 | 1 | 1 | 1 | 1 | 1 | 1 | 1 |
| 3127 | 58 | 2 | 163 | 76 | 8 | 2 | 1 | 1 | 4 | 3 | 1 | 1 | 2 | 1 | 1 | 1 | 1 | 1 |
| 3128 | 58 | 2 | 163 | 71 | 4 | 3 | 1 | 1 | 3 | 3 | 1 | 1 | 1 | 1 | 1 | 1 | 1 | 2 |
| 3129 | 58 | 2 | 163 | 57 | 8 | 2 | 1 | 1 | 3 | 1 | 1 | 1 | 1 | 1 | 2 | 1 | 1 | 1 |
| 3130 | 58 | 2 | 163 | 55 | 3 | 2 | 1 | 1 | 5 | 4 | 1 | 1 | 2 | 1 | 1 | 1 | 1 | 1 |
| 3131 | 58 | 2 | 164 | 71 | 8 | 2 | 1 | 1 | 3 | 3 | 1 | 1 | 1 | 1 | 2 | 1 | 1 | 1 |
| 3132 | 58 | 2 | 165 | 65 | 8 | 2 | 1 | 1 | 4 | 3 | 1 | 1 | 1 | 1 | 1 | 1 | 1 | 1 |
| 3133 | 58 | 2 | 165 | 65 | 3 | 2 | 1 | 1 | 2 | 4 | 1 | 1 | 1 | 1 | 1 | 1 | 1 | 1 |
| 3134 | 58 | 2 | 166 | 65 | 8 | 2 | 1 | 1 | 4 | 1 | 1 | 1 | 1 | 1 | 1 | 1 | 1 | 1 |
| 3135 | 58 | 2 | 169 | 49 | 2 | 2 | 1 | 1 | 5 | 1 | 1 | 1 | 1 | 1 | 1 | 1 | 1 | 1 |
| 3136 | 58 | 2 | 170 | 70 | 1 | 2 | 1 | 1 | 4 | 3 | 1 | 1 | 1 | 1 | 1 | 1 | 1 | 1 |
| 3137 | 58 | 1 | 155 | 67 | 2 | 2 | 2 | 2 | 3 | 2 | 1 | 1 | 1 | 2 | 1 | 1 | 3 | 1 |
| 3138 | 58 | 1 | 159.5 | 57.9 | 6 | 2 | 1 | 1 | 3 | 4 | 1 | 1 | 1 | 1 | 1 | 1 | 3 | 1 |
| 3139 | 58 | 1 | 160 | 51.4 | 8 | 2 | 1 | 1 | 4 | 3 | 1 | 1 | 1 | 1 | 1 | 1 | 1 | 1 |
| 3140 | 58 | 1 | 160.5 | 65 | 5 | 4 | 1 | 1 | 4 | 4 | 1 | 1 | 1 | 1 | 1 | 1 | 3 | 1 |
| 3141 | 58 | 1 | 162 | 90 | 8 | 2 | 1 | 1 | 6 | 4 | 1 | 1 | 1 | 2 | 1 | 1 | 3 | 1 |
| 3142 | 58 | 1 | 162 | 79 | 8 | 2 | 1 | 2 | 3 | 2 | 1 | 1 | 1 | 1 | 1 | 1 | 3 | 1 |
| 3143 | 58 | 1 | 162 | 53.3 | 8 | 2 | 1 | 1 | 3 | 1 | 1 | 1 | 1 | 1 | 1 | 1 | 1 | 1 |
| 3144 | 58 | 1 | 163 | 59 | 6 | 2 | 1 | 1 | 3 | 2 | 1 | 1 | 1 | 1 | 1 | 1 | 3 | 1 |
| 3145 | 58 | 1 | 163 | 58 | 8 | 2 | 1 | 2 | 3 | 1 | 1 | 1 | 1 | 1 | 1 | 1 | 1 | 1 |
| 3146 | 58 | 1 | 163 | 30 | 8 | 2 | 1 | 4 | 3 | 3 | 1 | 1 | 1 | 1 | 1 | 1 | 3 | 1 |
| 3147 | 58 | 1 | 164 | 61.2 | 2 | 2 | 1 | 1 | 3 | 1 | 2 | 1 | 1 | 1 | 1 | 1 | 1 | 1 |
| 3148 | 58 | 1 | 165 | 79.5 | 5 | 2 | 1 | 2 | 4 | 1 | 1 | 1 | 1 | 1 | 1 | 1 | 1 | 1 |
| 3149 | 58 | 1 | 165 | 70 | 8 | 2 | 1 | 2 | 4 | 3 | 1 | 1 | 1 | 2 | 1 | 1 | 3 | 2 |
| 3150 | 58 | 1 | 165 | 70 | 8 | 2 | 1 | 1 | 4 | 4 | 1 | 1 | 1 | 1 | 1 | 1 | 1 | 1 |
| 3151 | 58 | 1 | 165 | 70 | 4 | 2 | 1 | 2 | 3 | 2 | 1 | 1 | 1 | 1 | 1 | 1 | 3 | 1 |
| 3152 | 58 | 1 | 165 | 65 | 8 | 2 | 1 | 1 | 3 | 3 | 1 | 1 | 1 | 1 | 1 | 1 | 1 | 1 |
| 3153 | 58 | 1 | 165 | 48 | 4 | 2 | 1 | 2 | 3 | 3 | 1 | 1 | 1 | 1 | 2 | 1 | 3 | 1 |
| 3154 | 58 | 1 | 166 | 61 | 4 | 2 | 1 | 1 | 5 | 4 | 1 | 1 | 1 | 1 | 1 | 1 | 1 | 2 |
| 3155 | 58 | 1 | 168 | 72 | 8 | 2 | 1 | 1 | 4 | 2 | 1 | 1 | 1 | 1 | 1 | 1 | 1 | 1 |
| 3156 | 58 | 1 | 168 | 68 | 8 | 2 | 1 | 1 | 4 | 1 | 1 | 1 | 1 | 1 | 1 | 1 | 1 | 1 |
| 3157 | 58 | 1 | 168 | 68 | 2 | 2 | 1 | 1 | 4 | 3 | 1 | 1 | 1 | 2 | 1 | 1 | 1 | 1 |
| 3158 | 58 | 1 | 168 | 56 | 8 | 2 | 1 | 4 | 3 | 2 | 1 | 1 | 1 | 1 | 1 | 1 | 3 | 1 |
| 3159 | 58 | 1 | 170 | 73 | 4 | 2 | 1 | 2 | 5 | 1 | 1 | 1 | 1 | 2 | 1 | 1 | 3 | 2 |
| 3160 | 58 | 1 | 170 | 65 | 8 | 2 | 1 | 4 | 3 | 4 | 1 | 1 | 1 | 1 | 1 | 1 | 3 | 1 |
| 3161 | 58 | 1 | 170 | 64 | 8 | 2 | 1 | 1 | 4 | 4 | 1 | 1 | 1 | 2 | 1 | 1 | 1 | 1 |
| 3162 | 58 | 1 | 170 | 62 | 8 | 2 | 1 | 1 | 4 | 4 | 1 | 1 | 1 | 1 | 1 | 1 | 1 | 1 |
| 3163 | 58 | 1 | 170 | 62 | 8 | 2 | 1 | 1 | 3 | 3 | 1 | 1 | 1 | 1 | 1 | 1 | 3 | 2 |
| 3164 | 58 | 1 | 170 | 48 | 8 | 2 | 1 | 1 | 3 | 1 | 1 | 1 | 1 | 1 | 1 | 1 | 1 | 1 |
| 3165 | 58 | 1 | 174 | 70 | 4 | 2 | 1 | 2 | 3 | 4 | 1 | 1 | 1 | 2 | 1 | 1 | 3 | 2 |
| 3166 | 58 | 1 | 175 | 75 | 3 | 2 | 1 | 1 | 4 | 4 | 1 | 1 | 1 | 1 | 1 | 1 | 1 | 1 |
| 3167 | 58 | 1 | 183 | 92 | 6 | 2 | 2 | 4 | 4 | 4 | 1 | 1 | 1 | 1 | 1 | 1 | 1 | 2 |
| 3168 | 59 | 2 | 143 | 42.5 | 2 | 1 | 1 | 1 | 4 | 1 | 1 | 1 | 1 | 1 | 1 | 1 | 1 | 1 |
| 3169 | 59 | 2 | 147.5 | 40 | 6 | 3 | 1 | 1 | 3 | 1 | 1 | 1 | 1 | 1 | 1 | 1 | 1 | 1 |
| 3170 | 59 | 2 | 148 | 60 | 8 | 2 | 1 | 1 | 3 | 1 | 1 | 1 | 1 | 1 | 1 | 1 | 1 | 1 |
| 3171 | 59 | 2 | 149 | 58 | 8 | 3 | 1 | 1 | 2 | 1 | 1 | 1 | 1 | 1 | 1 | 1 | 1 | 1 |
| 3172 | 59 | 2 | 149 | 42 | 8 | 2 | 1 | 1 | 4 | 1 | 1 | 1 | 1 | 1 | 2 | 2 | 1 | 1 |
| 3173 | 59 | 2 | 150 | 59.7 | 2 | 2 | 2 | 1 | 3 | 2 | 1 | 1 | 2 | 1 | 1 | 1 | 1 | 1 |
| 3174 | 59 | 2 | 150 | 58 | 8 | 2 | 1 | 1 | 3 | 2 | 1 | 1 | 1 | 1 | 1 | 1 | 1 | 1 |
| 3175 | 59 | 2 | 150 | 49.5 | 8 | 2 | 1 | 1 | 3 | 1 | 1 | 1 | 1 | 1 | 1 | 1 | 1 | 1 |
| 3176 | 59 | 2 | 150 | 48 | 3 | 2 | 1 | 1 | 4 | 1 | 1 | 1 | 1 | 1 | 1 | 1 | 1 | 1 |
| 3177 | 59 | 2 | 150.5 | 50 | 8 | 1 | 1 | 1 | 1 | 1 | 1 | 1 | 1 | 1 | 1 | 1 | 1 | 1 |
| 3178 | 59 | 2 | 151.5 | 56.7 | 8 | 2 | 1 | 1 | 4 | 1 | 1 | 1 | 1 | 1 | 1 | 1 | 1 | 1 |
| 3179 | 59 | 2 | 151.5 | 53 | 8 | 2 | 1 | 1 | 3 | 1 | 1 | 1 | 1 | 1 | 1 | 1 | 1 | 1 |
| 3180 | 59 | 2 | 151.5 | 48 | 8 | 2 | 1 | 1 | 4 | 1 | 1 | 1 | 1 | 1 | 1 | 1 | 1 | 1 |
| 3181 | 59 | 2 | 152 | 52 | 8 | 2 | 1 | 1 | 4 | 3 | 1 | 1 | 1 | 1 | 1 | 1 | 1 | 1 |
| 3182 | 59 | 2 | 152.2 | 57.2 | 8 | 2 | 1 | 1 | 3 | 2 | 2 | 1 | 1 | 1 | 1 | 1 | 1 | 1 |
| 3183 | 59 | 2 | 153 | 62 | 8 | 2 | 1 | 1 | 4 | 3 | 2 | 1 | 1 | 2 | 1 | 1 | 1 | 1 |
| 3184 | 59 | 2 | 153 | 60 | 2 | 4 | 1 | 1 | 5 | 3 | 1 | 1 | 2 | 1 | 1 | 1 | 1 | 1 |
| 3185 | 59 | 2 | 153 | 57 | 8 | 2 | 1 | 1 | 3 | 1 | 1 | 1 | 1 | 1 | 2 | 1 | 1 | 1 |
| 3186 | 59 | 2 | 153 | 55 | 8 | 2 | 1 | 1 | 4 | 3 | 2 | 1 | 1 | 1 | 2 | 1 | 1 | 1 |
| 3187 | 59 | 2 | 153 | 53 | 4 | 2 | 1 | 1 | 3 | 1 | 1 | 1 | 2 | 1 | 1 | 1 | 1 | 1 |
| 3188 | 59 | 2 | 153 | 53 | 3 | 2 | 1 | 1 | 4 | 1 | 1 | 1 | 1 | 1 | 1 | 1 | 1 | 1 |
| 3189 | 59 | 2 | 153 | 48 | 5 | 2 | 1 | 1 | 4 | 4 | 1 | 1 | 1 | 1 | 1 | 1 | 1 | 1 |
| 3190 | 59 | 2 | 153 | 46 | 8 | 2 | 1 | 1 | 3 | 1 | 1 | 1 | 1 | 1 | 2 | 1 | 1 | 1 |
| 3191 | 59 | 2 | 153.5 | 50 | 4 | 2 | 1 | 1 | 3 | 4 | 1 | 1 | 1 | 1 | 1 | 1 | 1 | 1 |
| 3192 | 59 | 2 | 154 | 61 | 8 | 2 | 1 | 1 | 2 | 4 | 1 | 1 | 1 | 1 | 1 | 1 | 1 | 1 |
| 3193 | 59 | 2 | 154 | 58 | 3 | 2 | 1 | 1 | 3 | 2 | 1 | 2 | 1 | 2 | 2 | 1 | 1 | 2 |
| 3194 | 59 | 2 | 154 | 42 | 8 | 2 | 1 | 1 | 3 | 2 | 1 | 1 | 1 | 1 | 1 | 1 | 1 | 1 |
| 3195 | 59 | 2 | 154.5 | 58 | 8 | 2 | 1 | 1 | 3 | 1 | 1 | 1 | 1 | 1 | 1 | 1 | 1 | 1 |
| 3196 | 59 | 2 | 154.8 | 51 | 4 | 2 | 1 | 1 | 4 | 1 | 1 | 1 | 2 | 1 | 1 | 1 | 1 | 1 |
| 3197 | 59 | 2 | 155 | 78 | 8 | 2 | 1 | 1 | 2 | 3 | 1 | 1 | 1 | 1 | 1 | 1 | 1 | 1 |
| 3198 | 59 | 2 | 155 | 65.2 | 8 | 2 | 1 | 1 | 4 | 2 | 1 | 1 | 1 | 1 | 2 | 1 | 1 | 1 |
| 3199 | 59 | 2 | 155 | 64.5 | 8 | 2 | 1 | 1 | 4 | 4 | 1 | 1 | 1 | 1 | 1 | 1 | 1 | 1 |
| 3200 | 59 | 2 | 155 | 62 | 8 | 2 | 1 | 1 | 3 | 4 | 1 | 1 | 1 | 1 | 1 | 1 | 3 | 1 |
| 3201 | 59 | 2 | 155 | 58.5 | 8 | 2 | 1 | 1 | 2 | 2 | 1 | 1 | 1 | 1 | 1 | 1 | 1 | 1 |
| 3202 | 59 | 2 | 155 | 58 | 8 | 2 | 1 | 1 | 3 | 1 | 1 | 1 | 1 | 1 | 1 | 1 | 1 | 1 |
| 3203 | 59 | 2 | 155 | 57.5 | 8 | 2 | 1 | 2 | 3 | 3 | 1 | 1 | 1 | 1 | 1 | 1 | 1 | 1 |
| 3204 | 59 | 2 | 155 | 57.5 | 4 | 2 | 1 | 1 | 5 | 3 | 1 | 1 | 2 | 1 | 1 | 1 | 1 | 1 |
| 3205 | 59 | 2 | 155 | 50 | 8 | 2 | 1 | 1 | 4 | 3 | 1 | 1 | 1 | 1 | 1 | 1 | 1 | 1 |
| 3206 | 59 | 2 | 155 | 50 | 8 | 2 | 1 | 1 | 3 | 4 | 1 | 1 | 1 | 1 | 2 | 1 | 1 | 1 |
| 3207 | 59 | 2 | 155 | 47 | 8 | 1 | 1 | 1 | 3 | 2 | 1 | 1 | 2 | 1 | 1 | 1 | 1 | 1 |
| 3208 | 59 | 2 | 155 | 45 | 8 | 2 | 1 | 1 | 4 | 1 | 1 | 1 | 1 | 1 | 1 | 1 | 1 | 1 |
| 3209 | 59 | 2 | 155 | 45 | 6 | 3 | 1 | 1 | 3 | 3 | 1 | 1 | 1 | 1 | 1 | 1 | 1 | 1 |
| 3210 | 59 | 2 | 155.5 | 78 | 8 | 3 | 1 | 1 | 3 | 1 | 1 | 1 | 1 | 1 | 1 | 1 | 1 | 1 |
| 3211 | 59 | 2 | 155.5 | 77.9 | 8 | 2 | 1 | 1 | 3 | 4 | 1 | 1 | 1 | 1 | 1 | 1 | 1 | 1 |
| 3212 | 59 | 2 | 155.5 | 47.5 | 8 | 2 | 1 | 1 | 4 | 1 | 1 | 1 | 1 | 2 | 1 | 1 | 1 | 1 |
| 3213 | 59 | 2 | 156 | 69.5 | 8 | 2 | 1 | 1 | 3 | 4 | 1 | 1 | 1 | 1 | 1 | 1 | 1 | 1 |
| 3214 | 59 | 2 | 156 | 62 | 4 | 2 | 1 | 1 | 4 | 4 | 1 | 1 | 1 | 1 | 1 | 2 | 1 | 1 |
| 3215 | 59 | 2 | 156 | 60 | 8 | 2 | 1 | 1 | 3 | 1 | 1 | 1 | 1 | 1 | 1 | 1 | 1 | 1 |
| 3216 | 59 | 2 | 156 | 58 | 8 | 3 | 1 | 1 | 4 | 2 | 1 | 1 | 1 | 1 | 1 | 1 | 1 | 1 |
| 3217 | 59 | 2 | 156 | 54 | 8 | 2 | 1 | 1 | 4 | 2 | 1 | 1 | 1 | 1 | 1 | 1 | 1 | 1 |
| 3218 | 59 | 2 | 156 | 48 | 8 | 2 | 1 | 1 | 3 | 3 | 1 | 1 | 1 | 1 | 1 | 1 | 1 | 1 |
| 3219 | 59 | 2 | 157 | 68 | 6 | 2 | 1 | 1 | 4 | 4 | 1 | 1 | 2 | 1 | 1 | 1 | 1 | 1 |
| 3220 | 59 | 2 | 157.5 | 47.6 | 2 | 2 | 1 | 1 | 4 | 1 | 1 | 1 | 2 | 1 | 1 | 1 | 1 | 1 |
| 3221 | 59 | 2 | 157.9 | 69.5 | 2 | 2 | 1 | 1 | 4 | 3 | 1 | 1 | 2 | 1 | 2 | 1 | 1 | 1 |
| 3222 | 59 | 2 | 158 | 77 | 8 | 2 | 1 | 1 | 2 | 4 | 1 | 2 | 1 | 1 | 1 | 1 | 1 | 1 |
| 3223 | 59 | 2 | 158 | 77 | 2 | 2 | 1 | 1 | 3 | 3 | 2 | 1 | 1 | 2 | 2 | 1 | 1 | 1 |
| 3224 | 59 | 2 | 158 | 60.5 | 8 | 2 | 1 | 1 | 5 | 1 | 1 | 1 | 2 | 1 | 1 | 1 | 1 | 2 |
| 3225 | 59 | 2 | 158 | 60 | 3 | 2 | 1 | 1 | 4 | 1 | 1 | 1 | 2 | 1 | 1 | 1 | 1 | 1 |
| 3226 | 59 | 2 | 158 | 58 | 2 | 2 | 1 | 1 | 3 | 4 | 1 | 1 | 1 | 1 | 1 | 1 | 1 | 1 |
| 3227 | 59 | 2 | 158 | 55 | 8 | 2 | 1 | 1 | 6 | 4 | 1 | 1 | 1 | 1 | 1 | 1 | 1 | 1 |
| 3228 | 59 | 2 | 158 | 52 | 8 | 2 | 1 | 1 | 3 | 3 | 1 | 1 | 2 | 1 | 1 | 1 | 1 | 1 |
| 3229 | 59 | 2 | 158 | 52 | 4 | 2 | 1 | 1 | 4 | 1 | 3 | 1 | 1 | 1 | 1 | 1 | 1 | 1 |
| 3230 | 59 | 2 | 158 | 48 | 8 | 2 | 1 | 1 | 4 | 1 | 1 | 1 | 1 | 1 | 1 | 1 | 1 | 1 |
| 3231 | 59 | 2 | 158 | 45 | 8 | 2 | 1 | 1 | 5 | 3 | 1 | 1 | 1 | 1 | 1 | 1 | 1 | 1 |
| 3232 | 59 | 2 | 159 | 76.8 | 1 | 2 | 1 | 1 | 4 | 4 | 1 | 1 | 2 | 1 | 1 | 1 | 1 | 1 |
| 3233 | 59 | 2 | 159 | 64 | 4 | 2 | 1 | 1 | 4 | 4 | 1 | 1 | 1 | 1 | 1 | 1 | 1 | 1 |
| 3234 | 59 | 2 | 160 | 75 | 8 | 2 | 1 | 1 | 3 | 1 | 1 | 1 | 1 | 1 | 2 | 1 | 1 | 1 |
| 3235 | 59 | 2 | 160 | 71 | 8 | 2 | 1 | 1 | 3 | 1 | 1 | 1 | 1 | 1 | 2 | 2 | 1 | 1 |
| 3236 | 59 | 2 | 160 | 65 | 8 | 2 | 1 | 1 | 4 | 3 | 1 | 1 | 1 | 1 | 2 | 1 | 1 | 1 |
| 3237 | 59 | 2 | 160 | 65 | 8 | 2 | 1 | 1 | 3 | 4 | 1 | 1 | 1 | 1 | 1 | 1 | 1 | 1 |
| 3238 | 59 | 2 | 160 | 65 | 4 | 2 | 1 | 1 | 2 | 2 | 1 | 1 | 1 | 1 | 1 | 1 | 1 | 1 |
| 3239 | 59 | 2 | 160 | 64.1 | 2 | 2 | 1 | 1 | 4 | 2 | 1 | 1 | 1 | 1 | 2 | 1 | 1 | 1 |
| 3240 | 59 | 2 | 160 | 62.5 | 8 | 2 | 1 | 2 | 4 | 3 | 1 | 1 | 2 | 1 | 1 | 1 | 1 | 1 |
| 3241 | 59 | 2 | 160 | 62 | 4 | 2 | 1 | 1 | 4 | 2 | 1 | 1 | 1 | 1 | 1 | 1 | 1 | 1 |
| 3242 | 59 | 2 | 160 | 58 | 6 | 2 | 1 | 1 | 3 | 2 | 1 | 1 | 1 | 1 | 1 | 1 | 1 | 1 |
| 3243 | 59 | 2 | 160 | 57.5 | 2 | 2 | 1 | 1 | 5 | 1 | 1 | 1 | 1 | 1 | 1 | 1 | 1 | 1 |
| 3244 | 59 | 2 | 160 | 55 | 3 | 2 | 1 | 1 | 4 | 3 | 1 | 1 | 1 | 1 | 2 | 1 | 1 | 1 |
| 3245 | 59 | 2 | 160 | 54 | 8 | 2 | 1 | 1 | 3 | 4 | 1 | 1 | 1 | 1 | 1 | 1 | 1 | 1 |
| 3246 | 59 | 2 | 160 | 54 | 8 | 2 | 1 | 1 | 2 | 3 | 1 | 1 | 1 | 1 | 1 | 1 | 1 | 1 |
| 3247 | 59 | 2 | 160 | 51 | 4 | 2 | 1 | 1 | 4 | 1 | 1 | 1 | 1 | 1 | 1 | 1 | 1 | 1 |
| 3248 | 59 | 2 | 161 | 79.5 | 8 | 2 | 1 | 1 | 5 | 1 | 1 | 1 | 1 | 1 | 2 | 1 | 1 | 1 |
| 3249 | 59 | 2 | 161 | 58 | 6 | 2 | 1 | 1 | 4 | 1 | 1 | 1 | 1 | 1 | 2 | 1 | 1 | 1 |
| 3250 | 59 | 2 | 162 | 69 | 8 | 2 | 1 | 1 | 4 | 2 | 1 | 1 | 2 | 1 | 1 | 1 | 1 | 1 |
| 3251 | 59 | 2 | 162 | 59 | 8 | 2 | 1 | 1 | 3 | 4 | 1 | 1 | 1 | 1 | 1 | 1 | 1 | 1 |
| 3252 | 59 | 2 | 162 | 54.4 | 3 | 2 | 1 | 1 | 3 | 4 | 1 | 1 | 2 | 1 | 1 | 1 | 1 | 1 |
| 3253 | 59 | 2 | 162 | 54 | 8 | 1 | 1 | 1 | 3 | 3 | 1 | 1 | 1 | 1 | 1 | 1 | 1 | 1 |
| 3254 | 59 | 2 | 165 | 75 | 8 | 3 | 1 | 1 | 2 | 1 | 1 | 1 | 1 | 1 | 2 | 1 | 1 | 1 |
| 3255 | 59 | 2 | 165 | 66 | 8 | 2 | 1 | 1 | 3 | 2 | 1 | 1 | 1 | 1 | 1 | 1 | 1 | 1 |
| 3256 | 59 | 2 | 166 | 85 | 8 | 2 | 1 | 1 | 3 | 4 | 1 | 1 | 1 | 1 | 2 | 1 | 1 | 1 |
| 3257 | 59 | 2 | 168 | 66 | 8 | 1 | 1 | 1 | 3 | 1 | 1 | 1 | 1 | 1 | 1 | 1 | 1 | 1 |
| 3258 | 59 | 1 | 100 | 29.5 | 8 | 2 | 1 | 1 | 2 | 3 | 1 | 1 | 1 | 1 | 1 | 1 | 1 | 1 |
| 3259 | 59 | 1 | 158 | 44 | 2 | 1 | 1 | 1 | 5 | 4 | 1 | 1 | 2 | 1 | 1 | 1 | 1 | 1 |
| 3260 | 59 | 1 | 160 | 65 | 8 | 2 | 1 | 1 | 4 | 3 | 1 | 1 | 1 | 1 | 1 | 1 | 3 | 1 |
| 3261 | 59 | 1 | 160 | 57 | 8 | 2 | 1 | 1 | 4 | 4 | 1 | 1 | 1 | 1 | 1 | 1 | 1 | 1 |
| 3262 | 59 | 1 | 160 | 50 | 8 | 2 | 1 | 1 | 4 | 4 | 1 | 1 | 1 | 1 | 1 | 1 | 1 | 1 |
| 3263 | 59 | 1 | 160 | 47 | 8 | 2 | 1 | 1 | 4 | 3 | 1 | 1 | 1 | 1 | 1 | 1 | 1 | 1 |
| 3264 | 59 | 1 | 161 | 63 | 8 | 2 | 1 | 2 | 2 | 3 | 1 | 1 | 1 | 1 | 1 | 1 | 1 | 1 |
| 3265 | 59 | 1 | 162 | 69 | 8 | 2 | 1 | 1 | 5 | 1 | 1 | 1 | 2 | 1 | 2 | 1 | 3 | 1 |
| 3266 | 59 | 1 | 162 | 55 | 8 | 2 | 1 | 1 | 5 | 3 | 1 | 1 | 1 | 1 | 1 | 1 | 3 | 1 |
| 3267 | 59 | 1 | 163 | 60 | 8 | 2 | 1 | 1 | 4 | 1 | 1 | 1 | 1 | 1 | 1 | 1 | 1 | 1 |
| 3268 | 59 | 1 | 163 | 57 | 8 | 2 | 1 | 2 | 5 | 1 | 1 | 1 | 1 | 1 | 1 | 1 | 2 | 1 |
| 3269 | 59 | 1 | 165 | 72.5 | 4 | 2 | 1 | 1 | 3 | 4 | 1 | 1 | 1 | 1 | 1 | 1 | 3 | 1 |
| 3270 | 59 | 1 | 165 | 70 | 8 | 2 | 1 | 1 | 4 | 2 | 1 | 1 | 1 | 1 | 2 | 1 | 3 | 1 |
| 3271 | 59 | 1 | 165 | 59 | 8 | 2 | 1 | 1 | 3 | 1 | 2 | 1 | 1 | 1 | 1 | 1 | 1 | 1 |
| 3272 | 59 | 1 | 165.5 | 61.3 | 8 | 2 | 1 | 4 | 3 | 2 | 1 | 1 | 1 | 1 | 2 | 1 | 3 | 1 |
| 3273 | 59 | 1 | 166 | 65 | 8 | 2 | 1 | 1 | 3 | 1 | 1 | 1 | 1 | 1 | 1 | 1 | 1 | 1 |
| 3274 | 59 | 1 | 166 | 64 | 8 | 2 | 1 | 1 | 4 | 1 | 2 | 1 | 1 | 2 | 1 | 1 | 3 | 2 |
| 3275 | 59 | 1 | 167 | 64 | 8 | 2 | 1 | 1 | 6 | 1 | 1 | 1 | 2 | 1 | 1 | 1 | 1 | 1 |
| 3276 | 59 | 1 | 168 | 80 | 8 | 2 | 1 | 2 | 3 | 3 | 1 | 1 | 2 | 1 | 1 | 1 | 3 | 1 |
| 3277 | 59 | 1 | 168 | 66.1 | 8 | 2 | 1 | 2 | 4 | 3 | 1 | 1 | 2 | 1 | 1 | 1 | 3 | 1 |
| 3278 | 59 | 1 | 168 | 60 | 8 | 2 | 1 | 1 | 2 | 3 | 1 | 1 | 1 | 1 | 1 | 1 | 1 | 1 |
| 3279 | 59 | 1 | 168.5 | 64 | 8 | 2 | 1 | 2 | 4 | 2 | 1 | 1 | 1 | 1 | 1 | 1 | 3 | 1 |
| 3280 | 59 | 1 | 169 | 66 | 8 | 2 | 1 | 1 | 3 | 2 | 1 | 1 | 1 | 1 | 1 | 1 | 1 | 1 |
| 3281 | 59 | 1 | 170 | 75 | 4 | 2 | 1 | 1 | 2 | 1 | 1 | 1 | 1 | 1 | 1 | 1 | 1 | 1 |
| 3282 | 59 | 1 | 170 | 74.5 | 8 | 2 | 1 | 1 | 3 | 2 | 1 | 1 | 1 | 1 | 1 | 1 | 1 | 1 |
| 3283 | 59 | 1 | 170 | 70 | 8 | 2 | 1 | 1 | 3 | 4 | 1 | 1 | 1 | 1 | 1 | 1 | 1 | 1 |
| 3284 | 59 | 1 | 170 | 69 | 8 | 2 | 1 | 4 | 4 | 3 | 1 | 1 | 1 | 1 | 1 | 1 | 3 | 1 |
| 3285 | 59 | 1 | 170 | 65 | 8 | 2 | 1 | 1 | 3 | 4 | 1 | 1 | 1 | 1 | 1 | 1 | 1 | 1 |
| 3286 | 59 | 1 | 170 | 63 | 4 | 2 | 1 | 2 | 3 | 1 | 1 | 1 | 1 | 1 | 1 | 1 | 3 | 1 |
| 3287 | 59 | 1 | 172 | 67 | 4 | 2 | 1 | 1 | 3 | 2 | 1 | 1 | 1 | 1 | 1 | 1 | 1 | 1 |
| 3288 | 59 | 1 | 172 | 60 | 8 | 2 | 1 | 2 | 4 | 4 | 1 | 1 | 1 | 1 | 1 | 1 | 3 | 1 |
| 3289 | 59 | 1 | 173 | 70 | 6 | 2 | 1 | 1 | 4 | 2 | 1 | 1 | 1 | 2 | 1 | 1 | 1 | 2 |
| 3290 | 59 | 1 | 173 | 66 | 8 | 2 | 1 | 1 | 3 | 2 | 1 | 1 | 2 | 2 | 1 | 1 | 3 | 2 |
| 3291 | 59 | 1 | 173 | 62 | 8 | 2 | 1 | 1 | 3 | 3 | 1 | 1 | 1 | 1 | 1 | 1 | 1 | 1 |
| 3292 | 59 | 1 | 174 | 87 | 4 | 3 | 1 | 1 | 4 | 2 | 1 | 1 | 1 | 1 | 1 | 1 | 1 | 1 |
| 3293 | 59 | 1 | 179 | 79 | 2 | 2 | 1 | 1 | 5 | 2 | 1 | 1 | 2 | 1 | 1 | 1 | 1 | 1 |
| 3294 | 59 | 1 | 181 | 70 | 6 | 2 | 1 | 1 | 4 | 3 | 1 | 1 | 1 | 1 | 1 | 1 | 2 | 1 |
| 3295 | 60 | 2 | 145 | 51 | 4 | 2 | 1 | 1 | 3 | 2 | 1 | 1 | 1 | 1 | 1 | 1 | 1 | 1 |
| 3296 | 60 | 2 | 146 | 41.5 | 2 | 2 | 1 | 1 | 2 | 4 | 1 | 1 | 1 | 1 | 1 | 1 | 1 | 1 |
| 3297 | 60 | 2 | 147 | 47 | 8 | 2 | 1 | 1 | 5 | 2 | 1 | 1 | 2 | 1 | 2 | 1 | 1 | 1 |
| 3298 | 60 | 2 | 148 | 55.4 | 6 | 2 | 1 | 1 | 3 | 1 | 1 | 1 | 2 | 1 | 2 | 1 | 1 | 1 |
| 3299 | 60 | 2 | 149 | 48 | 8 | 2 | 1 | 1 | 4 | 1 | 1 | 1 | 1 | 1 | 1 | 1 | 1 | 1 |
| 3300 | 60 | 2 | 150 | 71.6 | 8 | 2 | 1 | 1 | 3 | 1 | 1 | 1 | 1 | 1 | 1 | 1 | 2 | 1 |
| 3301 | 60 | 2 | 150 | 61.9 | 8 | 2 | 1 | 1 | 3 | 4 | 1 | 1 | 1 | 1 | 1 | 1 | 1 | 1 |
| 3302 | 60 | 2 | 150 | 60 | 8 | 2 | 1 | 1 | 3 | 1 | 1 | 1 | 1 | 1 | 2 | 1 | 1 | 1 |
| 3303 | 60 | 2 | 150 | 37 | 8 | 2 | 1 | 1 | 2 | 4 | 1 | 1 | 1 | 1 | 1 | 1 | 1 | 1 |
| 3304 | 60 | 2 | 151 | 50.4 | 8 | 2 | 1 | 1 | 4 | 1 | 1 | 1 | 1 | 1 | 1 | 1 | 1 | 1 |
| 3305 | 60 | 2 | 152 | 76 | 8 | 2 | 1 | 1 | 4 | 4 | 1 | 1 | 1 | 1 | 2 | 1 | 1 | 1 |
| 3306 | 60 | 2 | 152 | 60 | 8 | 3 | 1 | 1 | 4 | 4 | 1 | 1 | 1 | 1 | 1 | 1 | 1 | 1 |
| 3307 | 60 | 2 | 152 | 57 | 8 | 4 | 1 | 1 | 3 | 1 | 1 | 1 | 1 | 1 | 1 | 1 | 1 | 1 |
| 3308 | 60 | 2 | 152 | 54 | 8 | 2 | 1 | 1 | 5 | 2 | 1 | 1 | 1 | 1 | 1 | 1 | 1 | 1 |
| 3309 | 60 | 2 | 152 | 44 | 8 | 2 | 1 | 1 | 4 | 2 | 1 | 1 | 1 | 1 | 1 | 1 | 1 | 1 |
| 3310 | 60 | 2 | 152.5 | 49.5 | 3 | 3 | 1 | 1 | 4 | 2 | 1 | 1 | 2 | 1 | 2 | 2 | 1 | 1 |
| 3311 | 60 | 2 | 152.5 | 37.6 | 8 | 2 | 1 | 1 | 4 | 3 | 1 | 1 | 1 | 1 | 1 | 1 | 1 | 1 |
| 3312 | 60 | 2 | 153 | 52 | 6 | 2 | 2 | 1 | 4 | 1 | 1 | 1 | 1 | 1 | 1 | 1 | 1 | 1 |
| 3313 | 60 | 2 | 153 | 50 | 8 | 2 | 1 | 1 | 4 | 4 | 1 | 1 | 1 | 1 | 2 | 2 | 1 | 1 |
| 3314 | 60 | 2 | 153 | 50 | 8 | 2 | 1 | 1 | 2 | 2 | 1 | 1 | 1 | 1 | 1 | 1 | 1 | 2 |
| 3315 | 60 | 2 | 153 | 50 | 8 | 2 | 1 | 1 | 2 | 4 | 1 | 1 | 1 | 1 | 1 | 1 | 1 | 1 |
| 3316 | 60 | 2 | 153 | 35 | 3 | 2 | 1 | 1 | 4 | 4 | 1 | 1 | 2 | 1 | 1 | 1 | 1 | 1 |
| 3317 | 60 | 2 | 154 | 73 | 2 | 2 | 1 | 1 | 5 | 1 | 1 | 1 | 1 | 1 | 1 | 1 | 1 | 1 |
| 3318 | 60 | 2 | 154 | 60 | 6 | 2 | 1 | 1 | 3 | 4 | 1 | 1 | 1 | 1 | 1 | 1 | 1 | 1 |
| 3319 | 60 | 2 | 154 | 57 | 8 | 2 | 1 | 1 | 3 | 3 | 1 | 1 | 2 | 1 | 1 | 1 | 1 | 1 |
| 3320 | 60 | 2 | 154 | 53 | 6 | 2 | 1 | 1 | 4 | 2 | 1 | 1 | 1 | 1 | 1 | 1 | 1 | 1 |
| 3321 | 60 | 2 | 154 | 51 | 3 | 2 | 1 | 1 | 3 | 1 | 1 | 1 | 1 | 1 | 1 | 1 | 1 | 1 |
| 3322 | 60 | 2 | 154 | 50 | 2 | 2 | 1 | 1 | 4 | 4 | 1 | 1 | 1 | 1 | 1 | 1 | 1 | 1 |
| 3323 | 60 | 2 | 154 | 45 | 3 | 2 | 1 | 1 | 4 | 4 | 1 | 1 | 2 | 1 | 1 | 1 | 1 | 1 |
| 3324 | 60 | 2 | 155 | 67 | 8 | 2 | 1 | 1 | 2 | 3 | 1 | 1 | 1 | 2 | 1 | 1 | 1 | 2 |
| 3325 | 60 | 2 | 155 | 66 | 8 | 2 | 1 | 1 | 2 | 2 | 1 | 1 | 1 | 1 | 1 | 1 | 1 | 1 |
| 3326 | 60 | 2 | 155 | 65 | 3 | 2 | 2 | 1 | 3 | 1 | 1 | 1 | 1 | 1 | 1 | 1 | 1 | 1 |
| 3327 | 60 | 2 | 155 | 64 | 4 | 2 | 1 | 1 | 3 | 3 | 1 | 1 | 1 | 1 | 2 | 1 | 1 | 1 |
| 3328 | 60 | 2 | 155 | 62 | 8 | 2 | 1 | 1 | 4 | 2 | 1 | 1 | 1 | 1 | 1 | 1 | 1 | 1 |
| 3329 | 60 | 2 | 155 | 60 | 8 | 2 | 1 | 1 | 4 | 1 | 1 | 1 | 1 | 1 | 1 | 1 | 1 | 1 |
| 3330 | 60 | 2 | 155 | 56 | 6 | 2 | 1 | 1 | 3 | 1 | 1 | 1 | 1 | 1 | 2 | 1 | 1 | 1 |
| 3331 | 60 | 2 | 155 | 54 | 8 | 2 | 1 | 1 | 3 | 4 | 1 | 1 | 2 | 1 | 1 | 1 | 1 | 1 |
| 3332 | 60 | 2 | 155 | 48 | 8 | 2 | 1 | 1 | 3 | 1 | 1 | 1 | 1 | 1 | 1 | 1 | 1 | 1 |
| 3333 | 60 | 2 | 156 | 67 | 8 | 2 | 1 | 1 | 2 | 3 | 1 | 1 | 1 | 1 | 2 | 1 | 1 | 1 |
| 3334 | 60 | 2 | 156 | 65 | 8 | 2 | 1 | 1 | 3 | 2 | 1 | 1 | 1 | 1 | 1 | 1 | 1 | 1 |
| 3335 | 60 | 2 | 156 | 61.5 | 2 | 2 | 1 | 1 | 5 | 4 | 1 | 1 | 1 | 1 | 1 | 1 | 1 | 1 |
| 3336 | 60 | 2 | 156 | 59 | 8 | 2 | 1 | 1 | 4 | 3 | 1 | 1 | 1 | 1 | 1 | 1 | 1 | 1 |
| 3337 | 60 | 2 | 156 | 59 | 6 | 2 | 1 | 1 | 4 | 1 | 1 | 1 | 1 | 1 | 1 | 1 | 1 | 2 |
| 3338 | 60 | 2 | 156 | 55 | 8 | 2 | 1 | 1 | 4 | 1 | 1 | 1 | 1 | 1 | 1 | 1 | 1 | 1 |
| 3339 | 60 | 2 | 156 | 55 | 2 | 2 | 1 | 2 | 5 | 1 | 1 | 1 | 1 | 2 | 2 | 2 | 1 | 2 |
| 3340 | 60 | 2 | 156 | 50.5 | 8 | 3 | 1 | 1 | 4 | 3 | 1 | 1 | 1 | 1 | 1 | 1 | 1 | 1 |
| 3341 | 60 | 2 | 156.5 | 58.3 | 6 | 2 | 1 | 1 | 4 | 4 | 1 | 1 | 1 | 1 | 1 | 1 | 1 | 1 |
| 3342 | 60 | 2 | 157 | 63 | 8 | 2 | 1 | 1 | 4 | 4 | 1 | 1 | 1 | 1 | 1 | 1 | 1 | 1 |
| 3343 | 60 | 2 | 157 | 62.6 | 3 | 2 | 1 | 1 | 4 | 1 | 1 | 1 | 1 | 1 | 1 | 1 | 1 | 1 |
| 3344 | 60 | 2 | 157 | 59 | 6 | 2 | 1 | 1 | 2 | 1 | 1 | 1 | 2 | 1 | 2 | 1 | 1 | 1 |
| 3345 | 60 | 2 | 157 | 54 | 8 | 2 | 1 | 1 | 6 | 1 | 1 | 1 | 2 | 1 | 1 | 1 | 1 | 1 |
| 3346 | 60 | 2 | 157 | 52 | 8 | 2 | 1 | 1 | 2 | 3 | 1 | 1 | 1 | 1 | 2 | 1 | 1 | 1 |
| 3347 | 60 | 2 | 157 | 46 | 8 | 2 | 1 | 1 | 4 | 2 | 1 | 1 | 1 | 1 | 1 | 1 | 1 | 1 |
| 3348 | 60 | 2 | 158 | 73.2 | 4 | 2 | 1 | 1 | 3 | 2 | 2 | 1 | 1 | 1 | 2 | 1 | 1 | 1 |
| 3349 | 60 | 2 | 158 | 60.1 | 8 | 2 | 1 | 1 | 2 | 1 | 1 | 1 | 1 | 1 | 1 | 1 | 1 | 1 |
| 3350 | 60 | 2 | 158 | 55 | 8 | 2 | 1 | 1 | 4 | 1 | 1 | 1 | 1 | 1 | 1 | 1 | 1 | 2 |
| 3351 | 60 | 2 | 158 | 55 | 6 | 2 | 1 | 1 | 3 | 1 | 1 | 1 | 1 | 1 | 1 | 1 | 1 | 1 |
| 3352 | 60 | 2 | 158 | 41 | 4 | 2 | 1 | 1 | 4 | 1 | 2 | 1 | 1 | 1 | 1 | 1 | 1 | 1 |
| 3353 | 60 | 2 | 159 | 65 | 8 | 2 | 1 | 1 | 3 | 3 | 1 | 1 | 1 | 1 | 1 | 1 | 1 | 1 |
| 3354 | 60 | 2 | 159 | 62 | 3 | 2 | 2 | 1 | 4 | 1 | 1 | 1 | 1 | 1 | 1 | 1 | 1 | 1 |
| 3355 | 60 | 2 | 159 | 56 | 6 | 2 | 1 | 1 | 4 | 4 | 1 | 1 | 1 | 1 | 1 | 1 | 1 | 2 |
| 3356 | 60 | 2 | 159 | 55.5 | 8 | 2 | 1 | 1 | 4 | 2 | 1 | 1 | 1 | 1 | 2 | 1 | 1 | 1 |
| 3357 | 60 | 2 | 159 | 51.9 | 6 | 1 | 1 | 1 | 4 | 1 | 1 | 1 | 1 | 1 | 2 | 1 | 1 | 1 |
| 3358 | 60 | 2 | 159 | 49 | 8 | 2 | 1 | 1 | 5 | 2 | 1 | 1 | 2 | 1 | 1 | 1 | 1 | 1 |
| 3359 | 60 | 2 | 160 | 67 | 8 | 2 | 1 | 1 | 3 | 4 | 1 | 1 | 1 | 1 | 2 | 1 | 1 | 1 |
| 3360 | 60 | 2 | 160 | 64 | 8 | 2 | 1 | 1 | 3 | 1 | 1 | 1 | 1 | 1 | 1 | 1 | 1 | 1 |
| 3361 | 60 | 2 | 160 | 58 | 8 | 2 | 1 | 1 | 3 | 2 | 1 | 1 | 1 | 1 | 2 | 1 | 1 | 1 |
| 3362 | 60 | 2 | 160 | 56.4 | 6 | 2 | 1 | 1 | 4 | 2 | 1 | 1 | 1 | 1 | 1 | 1 | 1 | 1 |
| 3363 | 60 | 2 | 160 | 54 | 4 | 2 | 1 | 1 | 4 | 1 | 1 | 1 | 1 | 1 | 1 | 1 | 1 | 1 |
| 3364 | 60 | 2 | 160 | 50 | 3 | 2 | 1 | 1 | 4 | 3 | 1 | 1 | 1 | 1 | 2 | 1 | 1 | 1 |
| 3365 | 60 | 2 | 161 | 65 | 8 | 2 | 1 | 1 | 3 | 4 | 1 | 1 | 1 | 1 | 1 | 1 | 1 | 1 |
| 3366 | 60 | 2 | 161 | 64 | 4 | 2 | 1 | 1 | 4 | 1 | 1 | 1 | 1 | 1 | 1 | 1 | 1 | 1 |
| 3367 | 60 | 2 | 161 | 59.3 | 8 | 2 | 1 | 2 | 4 | 1 | 1 | 1 | 1 | 1 | 1 | 1 | 1 | 1 |
| 3368 | 60 | 2 | 161.5 | 62.5 | 2 | 2 | 1 | 1 | 3 | 1 | 1 | 1 | 1 | 1 | 1 | 1 | 1 | 1 |
| 3369 | 60 | 2 | 162 | 72 | 8 | 2 | 1 | 1 | 4 | 2 | 1 | 1 | 1 | 1 | 1 | 1 | 1 | 1 |
| 3370 | 60 | 2 | 162 | 71 | 8 | 2 | 1 | 1 | 3 | 1 | 1 | 1 | 1 | 1 | 1 | 1 | 1 | 1 |
| 3371 | 60 | 2 | 162 | 55 | 4 | 2 | 1 | 1 | 4 | 1 | 1 | 1 | 1 | 1 | 1 | 1 | 1 | 1 |
| 3372 | 60 | 2 | 163 | 70 | 8 | 2 | 1 | 1 | 5 | 1 | 1 | 1 | 2 | 1 | 1 | 1 | 1 | 1 |
| 3373 | 60 | 2 | 163 | 65 | 8 | 2 | 1 | 2 | 2 | 2 | 1 | 1 | 2 | 1 | 2 | 1 | 1 | 1 |
| 3374 | 60 | 2 | 163 | 59 | 8 | 2 | 1 | 1 | 3 | 1 | 1 | 1 | 1 | 1 | 1 | 1 | 1 | 1 |
| 3375 | 60 | 2 | 164 | 64 | 8 | 2 | 1 | 2 | 2 | 1 | 1 | 1 | 1 | 1 | 1 | 1 | 1 | 1 |
| 3376 | 60 | 2 | 164 | 61 | 8 | 2 | 1 | 1 | 4 | 1 | 1 | 1 | 1 | 1 | 1 | 1 | 1 | 1 |
| 3377 | 60 | 2 | 165 | 88 | 8 | 2 | 1 | 1 | 4 | 4 | 1 | 1 | 1 | 1 | 1 | 1 | 1 | 1 |
| 3378 | 60 | 2 | 165 | 66.4 | 8 | 2 | 1 | 1 | 4 | 3 | 1 | 1 | 2 | 1 | 1 | 1 | 1 | 1 |
| 3379 | 60 | 2 | 165 | 65 | 8 | 2 | 1 | 1 | 5 | 4 | 1 | 1 | 1 | 1 | 1 | 1 | 1 | 1 |
| 3380 | 60 | 2 | 165 | 63 | 8 | 2 | 1 | 1 | 3 | 2 | 1 | 1 | 1 | 1 | 1 | 1 | 1 | 1 |
| 3381 | 60 | 2 | 165 | 55 | 8 | 2 | 1 | 3 | 4 | 3 | 1 | 1 | 1 | 1 | 1 | 1 | 3 | 2 |
| 3382 | 60 | 2 | 165 | 54 | 8 | 2 | 1 | 1 | 4 | 3 | 1 | 1 | 1 | 1 | 2 | 1 | 1 | 1 |
| 3383 | 60 | 2 | 166 | 65 | 2 | 2 | 1 | 2 | 5 | 2 | 1 | 1 | 2 | 1 | 2 | 1 | 1 | 1 |
| 3384 | 60 | 2 | 166 | 59 | 8 | 2 | 1 | 1 | 6 | 1 | 1 | 1 | 2 | 1 | 1 | 1 | 1 | 1 |
| 3385 | 60 | 2 | 166.5 | 62.5 | 8 | 2 | 1 | 1 | 4 | 1 | 1 | 1 | 2 | 1 | 1 | 1 | 1 | 1 |
| 3386 | 60 | 2 | 167 | 62 | 8 | 2 | 2 | 1 | 5 | 4 | 1 | 1 | 2 | 1 | 2 | 1 | 1 | 1 |
| 3387 | 60 | 2 | 168 | 74 | 8 | 2 | 1 | 1 | 3 | 3 | 1 | 1 | 1 | 1 | 1 | 1 | 1 | 1 |
| 3388 | 60 | 2 | 168 | 73 | 8 | 2 | 1 | 1 | 2 | 3 | 1 | 1 | 1 | 1 | 1 | 1 | 1 | 1 |
| 3389 | 60 | 2 | 168 | 64.5 | 4 | 2 | 1 | 1 | 3 | 2 | 1 | 1 | 1 | 1 | 2 | 1 | 2 | 1 |
| 3390 | 60 | 1 | 155 | 72 | 8 | 2 | 1 | 1 | 4 | 2 | 1 | 1 | 1 | 1 | 1 | 1 | 1 | 1 |
| 3391 | 60 | 1 | 156 | 47 | 2 | 2 | 1 | 1 | 5 | 1 | 1 | 1 | 1 | 1 | 1 | 1 | 1 | 1 |
| 3392 | 60 | 1 | 159 | 65 | 8 | 2 | 1 | 2 | 4 | 1 | 1 | 1 | 1 | 1 | 1 | 1 | 1 | 1 |
| 3393 | 60 | 1 | 159.5 | 70 | 8 | 4 | 1 | 2 | 4 | 1 | 1 | 1 | 1 | 1 | 2 | 1 | 1 | 1 |
| 3394 | 60 | 1 | 160 | 90.5 | 8 | 2 | 1 | 1 | 4 | 2 | 1 | 1 | 1 | 1 | 2 | 1 | 2 | 1 |
| 3395 | 60 | 1 | 160 | 73 | 4 | 2 | 1 | 1 | 4 | 1 | 1 | 1 | 2 | 1 | 1 | 1 | 1 | 1 |
| 3396 | 60 | 1 | 160 | 65 | 2 | 2 | 1 | 1 | 5 | 1 | 1 | 1 | 1 | 1 | 1 | 1 | 1 | 1 |
| 3397 | 60 | 1 | 160 | 60.8 | 8 | 2 | 1 | 4 | 2 | 1 | 1 | 1 | 1 | 1 | 1 | 1 | 3 | 1 |
| 3398 | 60 | 1 | 160 | 60 | 8 | 2 | 1 | 1 | 4 | 2 | 1 | 1 | 1 | 1 | 1 | 1 | 1 | 2 |
| 3399 | 60 | 1 | 160 | 56 | 3 | 2 | 1 | 1 | 5 | 4 | 2 | 1 | 1 | 1 | 1 | 1 | 1 | 1 |
| 3400 | 60 | 1 | 160 | 42 | 8 | 2 | 1 | 1 | 4 | 3 | 1 | 1 | 1 | 1 | 1 | 1 | 3 | 1 |
| 3401 | 60 | 1 | 160.5 | 65 | 2 | 2 | 1 | 1 | 1 | 2 | 1 | 1 | 1 | 1 | 1 | 1 | 1 | 1 |
| 3402 | 60 | 1 | 161 | 60 | 8 | 2 | 1 | 1 | 3 | 4 | 1 | 1 | 1 | 1 | 1 | 1 | 1 | 1 |
| 3403 | 60 | 1 | 161.5 | 59 | 4 | 2 | 1 | 1 | 3 | 1 | 1 | 1 | 2 | 1 | 1 | 1 | 1 | 1 |
| 3404 | 60 | 1 | 162 | 54 | 8 | 2 | 1 | 1 | 4 | 1 | 1 | 1 | 1 | 1 | 1 | 1 | 2 | 1 |
| 3405 | 60 | 1 | 164 | 65.7 | 6 | 2 | 1 | 1 | 3 | 1 | 1 | 1 | 1 | 1 | 1 | 1 | 1 | 1 |
| 3406 | 60 | 1 | 165 | 75 | 1 | 1 | 1 | 1 | 4 | 3 | 1 | 1 | 1 | 1 | 1 | 1 | 1 | 1 |
| 3407 | 60 | 1 | 165 | 71 | 4 | 2 | 1 | 4 | 3 | 1 | 1 | 1 | 1 | 1 | 1 | 1 | 2 | 1 |
| 3408 | 60 | 1 | 165 | 64.5 | 8 | 2 | 1 | 1 | 3 | 3 | 1 | 1 | 1 | 1 | 1 | 1 | 1 | 1 |
| 3409 | 60 | 1 | 165 | 57 | 8 | 1 | 1 | 1 | 4 | 4 | 1 | 1 | 2 | 1 | 1 | 1 | 1 | 1 |
| 3410 | 60 | 1 | 165.5 | 57 | 2 | 2 | 1 | 4 | 4 | 1 | 1 | 1 | 1 | 1 | 1 | 1 | 3 | 1 |
| 3411 | 60 | 1 | 166 | 66 | 8 | 2 | 1 | 1 | 4 | 1 | 1 | 1 | 1 | 1 | 1 | 1 | 1 | 2 |
| 3412 | 60 | 1 | 166 | 66 | 8 | 2 | 1 | 1 | 3 | 2 | 1 | 1 | 1 | 1 | 1 | 1 | 3 | 1 |
| 3413 | 60 | 1 | 166 | 65 | 8 | 2 | 1 | 1 | 3 | 2 | 1 | 1 | 1 | 1 | 1 | 1 | 3 | 2 |
| 3414 | 60 | 1 | 166 | 57.5 | 8 | 2 | 1 | 1 | 4 | 1 | 1 | 1 | 1 | 1 | 1 | 1 | 1 | 1 |
| 3415 | 60 | 1 | 167 | 62 | 8 | 2 | 1 | 1 | 4 | 2 | 1 | 1 | 1 | 1 | 1 | 1 | 1 | 1 |
| 3416 | 60 | 1 | 167.5 | 71.6 | 4 | 2 | 1 | 1 | 4 | 3 | 1 | 1 | 1 | 1 | 2 | 1 | 1 | 1 |
| 3417 | 60 | 1 | 168 | 94.5 | 8 | 2 | 1 | 1 | 4 | 2 | 1 | 1 | 1 | 1 | 1 | 1 | 1 | 1 |
| 3418 | 60 | 1 | 168 | 67 | 8 | 2 | 1 | 1 | 4 | 2 | 1 | 1 | 1 | 1 | 2 | 1 | 3 | 1 |
| 3419 | 60 | 1 | 168 | 60 | 8 | 2 | 1 | 4 | 4 | 4 | 1 | 1 | 2 | 1 | 1 | 1 | 3 | 1 |
| 3420 | 60 | 1 | 168 | 58 | 6 | 2 | 1 | 2 | 4 | 2 | 1 | 1 | 1 | 1 | 1 | 1 | 3 | 1 |
| 3421 | 60 | 1 | 168 | 57 | 2 | 2 | 1 | 1 | 6 | 3 | 1 | 1 | 1 | 1 | 1 | 1 | 1 | 1 |
| 3422 | 60 | 1 | 168 | 49 | 8 | 2 | 1 | 1 | 4 | 4 | 1 | 1 | 1 | 1 | 2 | 2 | 3 | 1 |
| 3423 | 60 | 1 | 168.5 | 53.9 | 8 | 2 | 1 | 1 | 4 | 1 | 1 | 1 | 2 | 1 | 1 | 1 | 3 | 1 |
| 3424 | 60 | 1 | 169 | 66 | 8 | 2 | 1 | 1 | 4 | 4 | 1 | 1 | 1 | 1 | 1 | 1 | 3 | 1 |
| 3425 | 60 | 1 | 169 | 63 | 8 | 2 | 1 | 1 | 3 | 1 | 1 | 1 | 1 | 1 | 2 | 2 | 3 | 1 |
| 3426 | 60 | 1 | 169.5 | 69 | 8 | 2 | 1 | 1 | 4 | 4 | 1 | 1 | 1 | 1 | 1 | 1 | 1 | 1 |
| 3427 | 60 | 1 | 170 | 81 | 8 | 2 | 1 | 2 | 3 | 4 | 1 | 1 | 1 | 1 | 1 | 1 | 1 | 1 |
| 3428 | 60 | 1 | 170 | 65 | 6 | 2 | 1 | 2 | 4 | 2 | 1 | 1 | 2 | 1 | 1 | 1 | 2 | 1 |
| 3429 | 60 | 1 | 170 | 60 | 8 | 2 | 1 | 1 | 3 | 4 | 1 | 1 | 1 | 1 | 1 | 1 | 1 | 1 |
| 3430 | 60 | 1 | 170 | 56 | 8 | 2 | 1 | 1 | 3 | 1 | 1 | 1 | 1 | 1 | 1 | 1 | 3 | 1 |
| 3431 | 60 | 1 | 171 | 78 | 8 | 2 | 1 | 1 | 4 | 4 | 1 | 1 | 1 | 1 | 1 | 1 | 3 | 1 |
| 3432 | 60 | 1 | 171 | 68 | 2 | 2 | 1 | 1 | 4 | 1 | 1 | 1 | 1 | 1 | 1 | 1 | 3 | 1 |
| 3433 | 60 | 1 | 171 | 65 | 8 | 2 | 1 | 4 | 2 | 4 | 1 | 1 | 1 | 1 | 1 | 1 | 1 | 1 |
| 3434 | 60 | 1 | 171 | 63.8 | 3 | 2 | 1 | 3 | 4 | 4 | 1 | 1 | 1 | 1 | 1 | 1 | 1 | 1 |
| 3435 | 60 | 1 | 171 | 58 | 3 | 2 | 1 | 1 | 3 | 2 | 1 | 1 | 2 | 1 | 1 | 1 | 2 | 1 |
| 3436 | 60 | 1 | 173 | 90 | 8 | 2 | 1 | 2 | 3 | 2 | 1 | 1 | 1 | 1 | 1 | 1 | 2 | 2 |
| 3437 | 60 | 1 | 173 | 70.4 | 3 | 2 | 1 | 1 | 4 | 2 | 1 | 1 | 1 | 1 | 1 | 1 | 1 | 1 |
| 3438 | 60 | 1 | 173 | 63 | 8 | 2 | 1 | 1 | 5 | 2 | 1 | 1 | 2 | 1 | 1 | 1 | 1 | 1 |
| 3439 | 60 | 1 | 173 | 53 | 3 | 2 | 1 | 1 | 5 | 1 | 1 | 1 | 1 | 1 | 1 | 1 | 1 | 1 |
| 3440 | 60 | 1 | 175 | 75 | 8 | 2 | 1 | 1 | 5 | 1 | 1 | 1 | 1 | 1 | 1 | 1 | 1 | 1 |
| 3441 | 60 | 1 | 175 | 65 | 1 | 2 | 2 | 1 | 3 | 4 | 1 | 1 | 1 | 1 | 1 | 1 | 1 | 1 |
| 3442 | 60 | 1 | 176 | 51 | 8 | 2 | 1 | 1 | 4 | 3 | 1 | 1 | 1 | 1 | 1 | 1 | 1 | 1 |
| 3443 | 60 | 1 | 177 | 74.7 | 6 | 2 | 1 | 1 | 3 | 1 | 1 | 1 | 1 | 1 | 2 | 1 | 1 | 1 |
| 3444 | 61 | 2 | 140 | 46 | 8 | 4 | 1 | 1 | 2 | 1 | 1 | 1 | 1 | 1 | 1 | 1 | 1 | 1 |
| 3445 | 61 | 2 | 144.5 | 41.8 | 2 | 2 | 1 | 1 | 4 | 2 | 2 | 1 | 1 | 1 | 2 | 1 | 1 | 1 |
| 3446 | 61 | 2 | 145.5 | 46.5 | 8 | 2 | 1 | 1 | 2 | 1 | 1 | 1 | 1 | 1 | 1 | 1 | 1 | 1 |
| 3447 | 61 | 2 | 146 | 52 | 8 | 2 | 1 | 1 | 2 | 1 | 1 | 1 | 1 | 1 | 1 | 1 | 1 | 1 |
| 3448 | 61 | 2 | 147 | 58.5 | 1 | 2 | 1 | 1 | 3 | 1 | 1 | 1 | 2 | 1 | 1 | 1 | 1 | 1 |
| 3449 | 61 | 2 | 147 | 50.4 | 6 | 2 | 1 | 1 | 4 | 1 | 1 | 1 | 1 | 1 | 1 | 1 | 1 | 1 |
| 3450 | 61 | 2 | 148 | 52 | 8 | 2 | 1 | 1 | 3 | 1 | 1 | 1 | 1 | 1 | 1 | 1 | 1 | 1 |
| 3451 | 61 | 2 | 149 | 50 | 8 | 2 | 1 | 1 | 4 | 1 | 1 | 1 | 1 | 1 | 1 | 1 | 1 | 1 |
| 3452 | 61 | 2 | 150 | 61 | 8 | 2 | 1 | 1 | 2 | 4 | 1 | 1 | 1 | 1 | 1 | 1 | 1 | 1 |
| 3453 | 61 | 2 | 150 | 56 | 8 | 3 | 1 | 1 | 2 | 4 | 1 | 1 | 1 | 1 | 1 | 1 | 1 | 1 |
| 3454 | 61 | 2 | 150 | 51 | 8 | 2 | 1 | 1 | 3 | 2 | 1 | 1 | 1 | 1 | 1 | 1 | 1 | 1 |
| 3455 | 61 | 2 | 150 | 50 | 8 | 2 | 1 | 1 | 4 | 1 | 1 | 1 | 1 | 1 | 2 | 1 | 1 | 1 |
| 3456 | 61 | 2 | 150 | 50 | 8 | 2 | 1 | 1 | 4 | 1 | 1 | 1 | 1 | 1 | 1 | 1 | 1 | 1 |
| 3457 | 61 | 2 | 150 | 48 | 8 | 2 | 1 | 1 | 3 | 1 | 1 | 1 | 1 | 2 | 1 | 1 | 1 | 1 |
| 3458 | 61 | 2 | 150 | 48 | 4 | 2 | 1 | 1 | 3 | 4 | 1 | 1 | 1 | 1 | 1 | 1 | 1 | 1 |
| 3459 | 61 | 2 | 150 | 45.2 | 8 | 2 | 1 | 1 | 3 | 1 | 1 | 1 | 1 | 1 | 1 | 1 | 1 | 1 |
| 3460 | 61 | 2 | 150 | 44.5 | 8 | 2 | 1 | 1 | 4 | 1 | 1 | 1 | 1 | 1 | 1 | 1 | 1 | 1 |
| 3461 | 61 | 2 | 151 | 58.6 | 3 | 2 | 1 | 1 | 3 | 1 | 1 | 1 | 1 | 1 | 1 | 1 | 1 | 1 |
| 3462 | 61 | 2 | 151 | 55 | 6 | 2 | 1 | 1 | 3 | 1 | 1 | 1 | 1 | 1 | 2 | 1 | 1 | 1 |
| 3463 | 61 | 2 | 151 | 55 | 3 | 2 | 1 | 1 | 4 | 1 | 1 | 1 | 1 | 1 | 1 | 1 | 1 | 1 |
| 3464 | 61 | 2 | 152 | 60 | 8 | 2 | 1 | 1 | 2 | 2 | 1 | 1 | 2 | 1 | 1 | 1 | 1 | 1 |
| 3465 | 61 | 2 | 152 | 56.8 | 8 | 1 | 1 | 1 | 3 | 1 | 1 | 1 | 1 | 1 | 1 | 1 | 1 | 1 |
| 3466 | 61 | 2 | 152 | 56 | 8 | 2 | 1 | 1 | 3 | 2 | 1 | 1 | 1 | 1 | 2 | 1 | 1 | 1 |
| 3467 | 61 | 2 | 152 | 50 | 4 | 2 | 1 | 1 | 4 | 3 | 1 | 1 | 1 | 1 | 1 | 1 | 1 | 1 |
| 3468 | 61 | 2 | 153 | 59.5 | 6 | 2 | 1 | 1 | 3 | 1 | 1 | 1 | 1 | 1 | 1 | 1 | 1 | 1 |
| 3469 | 61 | 2 | 153 | 55 | 8 | 2 | 1 | 1 | 4 | 3 | 1 | 1 | 1 | 1 | 1 | 1 | 1 | 1 |
| 3470 | 61 | 2 | 153 | 55 | 4 | 2 | 1 | 1 | 3 | 4 | 1 | 1 | 1 | 1 | 1 | 1 | 1 | 1 |
| 3471 | 61 | 2 | 153 | 53 | 8 | 2 | 1 | 1 | 4 | 3 | 1 | 1 | 2 | 1 | 1 | 1 | 1 | 1 |
| 3472 | 61 | 2 | 153 | 52.5 | 3 | 3 | 1 | 1 | 6 | 2 | 1 | 1 | 1 | 1 | 2 | 1 | 1 | 1 |
| 3473 | 61 | 2 | 153 | 50 | 8 | 2 | 1 | 1 | 4 | 1 | 1 | 1 | 1 | 1 | 1 | 1 | 1 | 1 |
| 3474 | 61 | 2 | 153 | 50 | 4 | 2 | 2 | 1 | 4 | 1 | 1 | 1 | 1 | 1 | 1 | 1 | 1 | 1 |
| 3475 | 61 | 2 | 153 | 49.5 | 3 | 2 | 1 | 1 | 4 | 1 | 1 | 1 | 1 | 1 | 1 | 1 | 1 | 2 |
| 3476 | 61 | 2 | 153 | 45 | 8 | 2 | 1 | 1 | 3 | 1 | 1 | 1 | 1 | 1 | 1 | 1 | 1 | 1 |
| 3477 | 61 | 2 | 153 | 43 | 8 | 2 | 1 | 1 | 4 | 3 | 1 | 1 | 1 | 1 | 1 | 1 | 1 | 1 |
| 3478 | 61 | 2 | 154 | 60 | 8 | 2 | 1 | 1 | 4 | 2 | 1 | 1 | 1 | 1 | 1 | 1 | 1 | 1 |
| 3479 | 61 | 2 | 154 | 58 | 4 | 2 | 1 | 1 | 3 | 2 | 1 | 1 | 1 | 1 | 1 | 1 | 1 | 1 |
| 3480 | 61 | 2 | 154 | 55.2 | 3 | 2 | 1 | 1 | 3 | 1 | 1 | 1 | 1 | 1 | 2 | 1 | 1 | 1 |
| 3481 | 61 | 2 | 154 | 48 | 3 | 2 | 2 | 1 | 5 | 4 | 1 | 1 | 1 | 1 | 2 | 1 | 1 | 1 |
| 3482 | 61 | 2 | 154 | 46 | 1 | 2 | 1 | 1 | 4 | 2 | 1 | 1 | 1 | 1 | 1 | 1 | 1 | 1 |
| 3483 | 61 | 2 | 154.5 | 68.9 | 3 | 2 | 1 | 1 | 4 | 2 | 1 | 1 | 1 | 1 | 1 | 1 | 1 | 1 |
| 3484 | 61 | 2 | 154.5 | 51 | 3 | 2 | 1 | 1 | 4 | 1 | 1 | 1 | 1 | 1 | 1 | 1 | 1 | 1 |
| 3485 | 61 | 2 | 155 | 78 | 8 | 2 | 1 | 2 | 4 | 3 | 1 | 1 | 1 | 1 | 1 | 1 | 1 | 2 |
| 3486 | 61 | 2 | 155 | 60 | 8 | 2 | 1 | 1 | 4 | 2 | 1 | 1 | 1 | 1 | 1 | 1 | 1 | 1 |
| 3487 | 61 | 2 | 155 | 58 | 8 | 2 | 1 | 1 | 4 | 3 | 1 | 1 | 1 | 1 | 1 | 1 | 1 | 1 |
| 3488 | 61 | 2 | 155 | 57.6 | 3 | 2 | 1 | 1 | 5 | 1 | 1 | 1 | 1 | 2 | 1 | 1 | 1 | 2 |
| 3489 | 61 | 2 | 155 | 57 | 8 | 2 | 1 | 1 | 2 | 1 | 1 | 1 | 2 | 1 | 2 | 1 | 1 | 1 |
| 3490 | 61 | 2 | 155 | 57 | 6 | 2 | 1 | 1 | 3 | 1 | 1 | 1 | 1 | 1 | 2 | 2 | 1 | 1 |
| 3491 | 61 | 2 | 155 | 55 | 3 | 2 | 1 | 1 | 4 | 2 | 1 | 1 | 1 | 1 | 1 | 1 | 1 | 1 |
| 3492 | 61 | 2 | 155 | 52.5 | 8 | 2 | 1 | 2 | 2 | 2 | 1 | 1 | 1 | 1 | 1 | 1 | 1 | 1 |
| 3493 | 61 | 2 | 155 | 50.1 | 3 | 2 | 1 | 1 | 4 | 1 | 1 | 1 | 1 | 1 | 2 | 1 | 1 | 1 |
| 3494 | 61 | 2 | 155 | 50 | 6 | 2 | 1 | 1 | 2 | 1 | 1 | 1 | 1 | 1 | 1 | 1 | 1 | 1 |
| 3495 | 61 | 2 | 155 | 48 | 8 | 2 | 1 | 1 | 2 | 1 | 1 | 1 | 1 | 1 | 1 | 1 | 1 | 1 |
| 3496 | 61 | 2 | 155.3 | 59.6 | 8 | 2 | 1 | 1 | 3 | 2 | 1 | 1 | 1 | 1 | 1 | 1 | 1 | 1 |
| 3497 | 61 | 2 | 156 | 65.5 | 3 | 2 | 1 | 1 | 4 | 1 | 1 | 1 | 1 | 1 | 1 | 1 | 1 | 1 |
| 3498 | 61 | 2 | 156 | 62 | 8 | 4 | 1 | 1 | 4 | 1 | 1 | 1 | 1 | 1 | 1 | 1 | 1 | 1 |
| 3499 | 61 | 2 | 156 | 60 | 6 | 2 | 1 | 1 | 4 | 2 | 1 | 1 | 1 | 1 | 1 | 1 | 1 | 1 |
| 3500 | 61 | 2 | 156 | 58 | 6 | 2 | 1 | 1 | 3 | 1 | 1 | 1 | 1 | 1 | 1 | 1 | 1 | 1 |
| 3501 | 61 | 2 | 156 | 57 | 8 | 1 | 1 | 1 | 2 | 4 | 1 | 1 | 1 | 1 | 1 | 1 | 1 | 1 |
| 3502 | 61 | 2 | 156 | 50 | 3 | 2 | 1 | 1 | 4 | 4 | 1 | 1 | 1 | 1 | 1 | 1 | 1 | 1 |
| 3503 | 61 | 2 | 156 | 45 | 8 | 2 | 1 | 1 | 1 | 2 | 1 | 1 | 1 | 1 | 1 | 1 | 1 | 1 |
| 3504 | 61 | 2 | 157 | 64 | 8 | 2 | 1 | 1 | 3 | 2 | 1 | 1 | 1 | 1 | 1 | 1 | 1 | 1 |
| 3505 | 61 | 2 | 157 | 61 | 8 | 2 | 1 | 2 | 4 | 2 | 1 | 1 | 1 | 1 | 1 | 1 | 1 | 2 |
| 3506 | 61 | 2 | 158 | 54 | 8 | 2 | 1 | 1 | 4 | 1 | 1 | 1 | 1 | 1 | 2 | 1 | 1 | 1 |
| 3507 | 61 | 2 | 158 | 52.5 | 8 | 2 | 1 | 1 | 4 | 1 | 1 | 1 | 2 | 1 | 1 | 1 | 1 | 1 |
| 3508 | 61 | 2 | 158 | 52.5 | 8 | 2 | 1 | 1 | 2 | 1 | 1 | 1 | 2 | 1 | 1 | 1 | 1 | 1 |
| 3509 | 61 | 2 | 158 | 51 | 8 | 2 | 1 | 1 | 6 | 4 | 1 | 1 | 1 | 1 | 1 | 1 | 1 | 1 |
| 3510 | 61 | 2 | 158 | 46 | 8 | 2 | 1 | 1 | 2 | 4 | 1 | 1 | 1 | 1 | 1 | 1 | 1 | 1 |
| 3511 | 61 | 2 | 158.5 | 67.7 | 8 | 2 | 1 | 1 | 4 | 1 | 1 | 1 | 1 | 1 | 1 | 1 | 1 | 1 |
| 3512 | 61 | 2 | 159.5 | 72 | 3 | 2 | 1 | 2 | 3 | 1 | 1 | 1 | 1 | 1 | 1 | 1 | 1 | 1 |
| 3513 | 61 | 2 | 159.5 | 70 | 8 | 2 | 1 | 1 | 3 | 2 | 1 | 1 | 1 | 1 | 1 | 1 | 1 | 1 |
| 3514 | 61 | 2 | 160 | 70 | 6 | 2 | 1 | 1 | 2 | 2 | 1 | 1 | 1 | 1 | 1 | 1 | 1 | 1 |
| 3515 | 61 | 2 | 160 | 63.5 | 2 | 2 | 1 | 1 | 5 | 1 | 1 | 1 | 1 | 1 | 2 | 1 | 1 | 1 |
| 3516 | 61 | 2 | 160 | 60 | 8 | 2 | 1 | 1 | 4 | 1 | 1 | 1 | 1 | 1 | 1 | 1 | 1 | 1 |
| 3517 | 61 | 2 | 160 | 60 | 4 | 4 | 1 | 1 | 3 | 1 | 1 | 1 | 1 | 1 | 1 | 1 | 1 | 1 |
| 3518 | 61 | 2 | 160 | 55 | 3 | 3 | 1 | 1 | 2 | 2 | 1 | 1 | 1 | 1 | 1 | 1 | 1 | 1 |
| 3519 | 61 | 2 | 161 | 62 | 3 | 2 | 1 | 1 | 5 | 2 | 1 | 1 | 1 | 1 | 1 | 1 | 3 | 1 |
| 3520 | 61 | 2 | 161 | 54 | 8 | 2 | 1 | 1 | 4 | 2 | 1 | 1 | 1 | 1 | 1 | 1 | 1 | 1 |
| 3521 | 61 | 2 | 162 | 63 | 8 | 2 | 1 | 1 | 4 | 1 | 1 | 1 | 1 | 1 | 1 | 1 | 1 | 1 |
| 3522 | 61 | 2 | 162 | 62 | 6 | 2 | 1 | 1 | 3 | 2 | 1 | 1 | 1 | 1 | 1 | 1 | 1 | 1 |
| 3523 | 61 | 2 | 162 | 60 | 3 | 4 | 1 | 1 | 4 | 1 | 2 | 1 | 2 | 1 | 2 | 2 | 1 | 1 |
| 3524 | 61 | 2 | 163 | 73 | 8 | 2 | 1 | 1 | 4 | 1 | 1 | 1 | 1 | 1 | 2 | 2 | 1 | 1 |
| 3525 | 61 | 2 | 163 | 70 | 2 | 2 | 1 | 1 | 5 | 2 | 1 | 1 | 2 | 1 | 1 | 1 | 1 | 1 |
| 3526 | 61 | 2 | 163 | 60 | 8 | 2 | 1 | 1 | 2 | 1 | 1 | 1 | 1 | 1 | 1 | 1 | 1 | 1 |
| 3527 | 61 | 2 | 164 | 53 | 8 | 2 | 1 | 1 | 4 | 1 | 1 | 1 | 1 | 1 | 1 | 1 | 1 | 1 |
| 3528 | 61 | 2 | 164 | 52.4 | 6 | 2 | 1 | 1 | 3 | 1 | 1 | 1 | 1 | 1 | 2 | 1 | 1 | 1 |
| 3529 | 61 | 2 | 165 | 79 | 8 | 2 | 1 | 1 | 4 | 2 | 1 | 1 | 1 | 1 | 1 | 1 | 1 | 1 |
| 3530 | 61 | 2 | 165 | 69 | 8 | 2 | 1 | 1 | 4 | 1 | 1 | 1 | 1 | 1 | 1 | 1 | 1 | 1 |
| 3531 | 61 | 2 | 165 | 68.6 | 6 | 3 | 1 | 1 | 3 | 1 | 2 | 1 | 2 | 1 | 1 | 1 | 1 | 1 |
| 3532 | 61 | 2 | 166 | 63 | 8 | 2 | 1 | 1 | 3 | 2 | 1 | 1 | 1 | 1 | 1 | 1 | 1 | 1 |
| 3533 | 61 | 2 | 170 | 60 | 4 | 2 | 1 | 1 | 4 | 1 | 1 | 1 | 1 | 1 | 1 | 1 | 1 | 1 |
| 3534 | 61 | 1 | 147.5 | 38 | 6 | 4 | 1 | 1 | 3 | 1 | 1 | 1 | 1 | 1 | 2 | 1 | 1 | 1 |
| 3535 | 61 | 1 | 150 | 60 | 6 | 2 | 1 | 1 | 3 | 4 | 1 | 1 | 1 | 1 | 1 | 1 | 1 | 1 |
| 3536 | 61 | 1 | 154 | 55 | 6 | 2 | 1 | 1 | 3 | 2 | 1 | 1 | 1 | 2 | 1 | 1 | 1 | 2 |
| 3537 | 61 | 1 | 160 | 77.5 | 8 | 1 | 1 | 1 | 4 | 4 | 1 | 1 | 1 | 1 | 1 | 1 | 1 | 1 |
| 3538 | 61 | 1 | 160 | 65 | 8 | 2 | 1 | 2 | 3 | 1 | 1 | 1 | 1 | 1 | 1 | 1 | 3 | 1 |
| 3539 | 61 | 1 | 160 | 60 | 8 | 2 | 1 | 2 | 3 | 1 | 1 | 1 | 1 | 1 | 1 | 1 | 3 | 1 |
| 3540 | 61 | 1 | 160 | 60 | 8 | 2 | 1 | 1 | 3 | 2 | 1 | 1 | 1 | 1 | 1 | 1 | 3 | 1 |
| 3541 | 61 | 1 | 160 | 58 | 1 | 2 | 1 | 1 | 4 | 4 | 1 | 1 | 1 | 1 | 1 | 1 | 2 | 1 |
| 3542 | 61 | 1 | 160 | 52.5 | 8 | 2 | 1 | 1 | 3 | 1 | 1 | 1 | 1 | 1 | 1 | 1 | 3 | 1 |
| 3543 | 61 | 1 | 160.5 | 81 | 8 | 2 | 1 | 2 | 4 | 4 | 1 | 1 | 2 | 1 | 1 | 1 | 3 | 1 |
| 3544 | 61 | 1 | 161 | 48 | 6 | 2 | 1 | 1 | 4 | 1 | 1 | 1 | 1 | 1 | 1 | 1 | 3 | 1 |
| 3545 | 61 | 1 | 161.5 | 57 | 8 | 2 | 1 | 1 | 5 | 1 | 1 | 1 | 1 | 1 | 1 | 1 | 1 | 2 |
| 3546 | 61 | 1 | 162 | 65 | 8 | 2 | 1 | 1 | 5 | 1 | 1 | 1 | 1 | 1 | 1 | 1 | 3 | 1 |
| 3547 | 61 | 1 | 162 | 59.5 | 8 | 2 | 1 | 1 | 3 | 1 | 1 | 1 | 2 | 1 | 1 | 1 | 1 | 1 |
| 3548 | 61 | 1 | 162 | 58 | 8 | 2 | 1 | 1 | 6 | 3 | 1 | 1 | 1 | 1 | 1 | 1 | 1 | 1 |
| 3549 | 61 | 1 | 163 | 75 | 6 | 2 | 1 | 1 | 6 | 4 | 1 | 1 | 1 | 1 | 1 | 1 | 2 | 2 |
| 3550 | 61 | 1 | 164 | 74 | 4 | 2 | 1 | 1 | 3 | 2 | 1 | 1 | 1 | 1 | 1 | 1 | 2 | 1 |
| 3551 | 61 | 1 | 164 | 67 | 8 | 2 | 1 | 1 | 4 | 1 | 1 | 1 | 1 | 1 | 2 | 2 | 1 | 1 |
| 3552 | 61 | 1 | 164 | 56 | 8 | 2 | 1 | 4 | 4 | 1 | 1 | 2 | 1 | 1 | 1 | 1 | 3 | 2 |
| 3553 | 61 | 1 | 165 | 60 | 8 | 2 | 1 | 1 | 4 | 1 | 1 | 1 | 1 | 1 | 1 | 1 | 3 | 1 |
| 3554 | 61 | 1 | 166 | 70 | 1 | 2 | 1 | 1 | 3 | 3 | 1 | 1 | 1 | 1 | 1 | 1 | 1 | 1 |
| 3555 | 61 | 1 | 166 | 66.4 | 8 | 2 | 1 | 2 | 4 | 1 | 1 | 1 | 1 | 1 | 1 | 1 | 3 | 2 |
| 3556 | 61 | 1 | 166 | 58.2 | 8 | 2 | 1 | 1 | 5 | 1 | 1 | 1 | 1 | 1 | 1 | 1 | 3 | 1 |
| 3557 | 61 | 1 | 166 | 51 | 1 | 1 | 1 | 1 | 3 | 1 | 1 | 1 | 1 | 1 | 1 | 1 | 1 | 1 |
| 3558 | 61 | 1 | 167 | 72.8 | 3 | 2 | 1 | 1 | 4 | 1 | 1 | 1 | 1 | 1 | 1 | 1 | 2 | 1 |
| 3559 | 61 | 1 | 167 | 71 | 8 | 2 | 1 | 1 | 3 | 4 | 1 | 1 | 2 | 1 | 1 | 1 | 1 | 1 |
| 3560 | 61 | 1 | 168 | 70 | 8 | 2 | 1 | 1 | 3 | 1 | 1 | 1 | 1 | 1 | 1 | 1 | 3 | 1 |
| 3561 | 61 | 1 | 168 | 64 | 8 | 2 | 2 | 1 | 3 | 3 | 1 | 1 | 1 | 2 | 1 | 1 | 1 | 2 |
| 3562 | 61 | 1 | 168 | 57 | 8 | 2 | 1 | 1 | 4 | 2 | 1 | 1 | 1 | 1 | 1 | 1 | 2 | 1 |
| 3563 | 61 | 1 | 169 | 65.1 | 3 | 2 | 1 | 1 | 4 | 1 | 1 | 1 | 1 | 1 | 1 | 1 | 1 | 1 |
| 3564 | 61 | 1 | 169 | 62 | 8 | 2 | 1 | 2 | 4 | 2 | 1 | 1 | 1 | 2 | 1 | 1 | 3 | 2 |
| 3565 | 61 | 1 | 169.5 | 57.4 | 8 | 1 | 1 | 1 | 3 | 4 | 1 | 1 | 1 | 1 | 1 | 1 | 3 | 1 |
| 3566 | 61 | 1 | 170 | 75 | 5 | 2 | 1 | 1 | 5 | 3 | 1 | 1 | 1 | 1 | 1 | 1 | 1 | 1 |
| 3567 | 61 | 1 | 170 | 70 | 2 | 2 | 1 | 1 | 4 | 2 | 1 | 1 | 1 | 1 | 1 | 1 | 1 | 1 |
| 3568 | 61 | 1 | 170 | 68 | 2 | 4 | 1 | 1 | 4 | 4 | 1 | 1 | 1 | 1 | 1 | 1 | 1 | 1 |
| 3569 | 61 | 1 | 170 | 59 | 6 | 2 | 1 | 1 | 4 | 1 | 1 | 1 | 1 | 1 | 1 | 1 | 3 | 1 |
| 3570 | 61 | 1 | 171 | 73 | 3 | 2 | 1 | 3 | 3 | 1 | 1 | 1 | 1 | 1 | 1 | 1 | 1 | 1 |
| 3571 | 61 | 1 | 171 | 62.5 | 8 | 2 | 1 | 1 | 4 | 4 | 1 | 1 | 1 | 1 | 1 | 1 | 1 | 1 |
| 3572 | 61 | 1 | 172 | 72 | 8 | 2 | 1 | 1 | 4 | 4 | 1 | 1 | 2 | 1 | 1 | 1 | 1 | 1 |
| 3573 | 61 | 1 | 172 | 70 | 6 | 2 | 1 | 1 | 4 | 1 | 1 | 1 | 1 | 1 | 1 | 1 | 1 | 1 |
| 3574 | 61 | 1 | 174 | 70 | 3 | 2 | 1 | 1 | 5 | 2 | 1 | 1 | 1 | 2 | 2 | 1 | 1 | 1 |
| 3575 | 61 | 1 | 175 | 70 | 8 | 2 | 1 | 1 | 4 | 1 | 1 | 1 | 1 | 1 | 1 | 1 | 1 | 1 |
| 3576 | 61 | 1 | 175.5 | 87.8 | 3 | 2 | 1 | 1 | 4 | 2 | 1 | 1 | 1 | 1 | 1 | 1 | 2 | 1 |
| 3577 | 61 | 1 | 176 | 70 | 8 | 2 | 1 | 2 | 4 | 2 | 1 | 1 | 1 | 1 | 1 | 1 | 1 | 1 |
| 3578 | 61 | 1 | 178 | 75 | 8 | 2 | 1 | 1 | 4 | 4 | 1 | 1 | 1 | 2 | 1 | 1 | 1 | 2 |
| 3579 | 61 | 1 | 180 | 93 | 8 | 2 | 1 | 1 | 4 | 4 | 1 | 1 | 1 | 2 | 1 | 2 | 1 | 1 |
| 3580 | 61 | 1 | 183 | 98 | 4 | 2 | 1 | 3 | 4 | 1 | 1 | 1 | 2 | 1 | 2 | 2 | 1 | 1 |
| 3581 | 62 | 2 | 130 | 47.5 | 8 | 1 | 1 | 1 | 3 | 1 | 1 | 1 | 1 | 1 | 1 | 1 | 1 | 1 |
| 3582 | 62 | 2 | 146 | 54.8 | 4 | 2 | 1 | 1 | 2 | 2 | 1 | 1 | 1 | 1 | 1 | 1 | 1 | 1 |
| 3583 | 62 | 2 | 146 | 41.5 | 2 | 2 | 1 | 1 | 4 | 2 | 1 | 1 | 1 | 1 | 1 | 1 | 1 | 1 |
| 3584 | 62 | 2 | 147 | 49 | 6 | 2 | 1 | 1 | 3 | 1 | 1 | 1 | 1 | 1 | 1 | 1 | 1 | 1 |
| 3585 | 62 | 2 | 147 | 38 | 6 | 2 | 1 | 1 | 3 | 1 | 1 | 1 | 1 | 1 | 1 | 1 | 1 | 1 |
| 3586 | 62 | 2 | 148 | 60 | 8 | 2 | 1 | 1 | 3 | 2 | 1 | 1 | 1 | 1 | 1 | 1 | 1 | 1 |
| 3587 | 62 | 2 | 148 | 57 | 5 | 2 | 1 | 2 | 4 | 1 | 1 | 1 | 1 | 1 | 1 | 1 | 1 | 1 |
| 3588 | 62 | 2 | 149 | 52 | 6 | 1 | 1 | 1 | 3 | 1 | 1 | 1 | 1 | 1 | 1 | 1 | 1 | 1 |
| 3589 | 62 | 2 | 149.5 | 65.5 | 8 | 3 | 1 | 1 | 5 | 3 | 1 | 1 | 1 | 1 | 2 | 1 | 1 | 1 |
| 3590 | 62 | 2 | 150 | 67 | 6 | 2 | 1 | 1 | 3 | 4 | 1 | 1 | 1 | 1 | 1 | 1 | 1 | 1 |
| 3591 | 62 | 2 | 150 | 62 | 8 | 2 | 1 | 1 | 2 | 4 | 1 | 1 | 1 | 1 | 2 | 1 | 1 | 1 |
| 3592 | 62 | 2 | 150 | 60 | 8 | 2 | 1 | 1 | 6 | 4 | 1 | 1 | 1 | 1 | 1 | 1 | 1 | 1 |
| 3593 | 62 | 2 | 150 | 53.5 | 6 | 2 | 1 | 1 | 3 | 1 | 1 | 1 | 1 | 1 | 1 | 1 | 1 | 1 |
| 3594 | 62 | 2 | 150 | 44 | 6 | 2 | 1 | 1 | 3 | 1 | 1 | 1 | 1 | 1 | 1 | 1 | 1 | 1 |
| 3595 | 62 | 2 | 150.5 | 56.2 | 1 | 2 | 1 | 1 | 4 | 1 | 1 | 1 | 1 | 1 | 1 | 1 | 1 | 1 |
| 3596 | 62 | 2 | 151 | 53 | 8 | 2 | 1 | 1 | 4 | 2 | 1 | 1 | 1 | 1 | 1 | 1 | 1 | 1 |
| 3597 | 62 | 2 | 151 | 42 | 8 | 2 | 1 | 1 | 3 | 1 | 1 | 1 | 1 | 1 | 1 | 1 | 1 | 1 |
| 3598 | 62 | 2 | 152 | 50 | 8 | 3 | 1 | 1 | 4 | 2 | 1 | 1 | 1 | 1 | 1 | 1 | 1 | 1 |
| 3599 | 62 | 2 | 153 | 70 | 8 | 2 | 1 | 1 | 3 | 4 | 1 | 1 | 1 | 1 | 1 | 1 | 1 | 1 |
| 3600 | 62 | 2 | 153 | 64 | 8 | 2 | 1 | 1 | 4 | 1 | 1 | 1 | 1 | 1 | 2 | 1 | 1 | 1 |
| 3601 | 62 | 2 | 153 | 60 | 8 | 2 | 1 | 1 | 4 | 4 | 1 | 1 | 2 | 1 | 1 | 1 | 1 | 1 |
| 3602 | 62 | 2 | 153 | 59.8 | 2 | 2 | 1 | 1 | 3 | 1 | 1 | 1 | 1 | 1 | 1 | 1 | 1 | 1 |
| 3603 | 62 | 2 | 153 | 56 | 8 | 2 | 1 | 1 | 3 | 4 | 1 | 1 | 1 | 2 | 1 | 1 | 1 | 2 |
| 3604 | 62 | 2 | 153 | 46.5 | 4 | 2 | 1 | 1 | 3 | 2 | 1 | 1 | 1 | 1 | 1 | 1 | 1 | 1 |
| 3605 | 62 | 2 | 154 | 73.4 | 6 | 2 | 1 | 1 | 2 | 1 | 2 | 1 | 1 | 1 | 2 | 1 | 1 | 2 |
| 3606 | 62 | 2 | 154 | 56 | 3 | 2 | 1 | 1 | 3 | 4 | 1 | 1 | 2 | 1 | 1 | 1 | 1 | 1 |
| 3607 | 62 | 2 | 154 | 55 | 8 | 2 | 1 | 1 | 2 | 4 | 1 | 1 | 1 | 1 | 1 | 1 | 1 | 1 |
| 3608 | 62 | 2 | 154 | 52 | 2 | 1 | 1 | 1 | 3 | 2 | 2 | 1 | 1 | 2 | 1 | 1 | 1 | 2 |
| 3609 | 62 | 2 | 154 | 50 | 8 | 2 | 1 | 1 | 3 | 1 | 1 | 1 | 1 | 1 | 2 | 1 | 3 | 1 |
| 3610 | 62 | 2 | 154.5 | 61 | 8 | 3 | 1 | 1 | 3 | 1 | 1 | 1 | 1 | 1 | 2 | 1 | 1 | 1 |
| 3611 | 62 | 2 | 155 | 69 | 1 | 2 | 1 | 1 | 4 | 4 | 1 | 1 | 1 | 1 | 1 | 1 | 1 | 1 |
| 3612 | 62 | 2 | 155 | 65 | 8 | 2 | 1 | 1 | 1 | 3 | 1 | 1 | 1 | 1 | 1 | 1 | 1 | 1 |
| 3613 | 62 | 2 | 155 | 55 | 3 | 2 | 1 | 1 | 6 | 2 | 1 | 1 | 1 | 1 | 1 | 1 | 1 | 1 |
| 3614 | 62 | 2 | 155 | 50 | 8 | 2 | 1 | 1 | 2 | 1 | 1 | 1 | 1 | 1 | 1 | 1 | 1 | 1 |
| 3615 | 62 | 2 | 155 | 50 | 2 | 2 | 1 | 1 | 4 | 2 | 1 | 1 | 1 | 1 | 1 | 1 | 1 | 1 |
| 3616 | 62 | 2 | 156 | 71 | 8 | 2 | 1 | 1 | 2 | 4 | 1 | 1 | 1 | 1 | 1 | 1 | 1 | 1 |
| 3617 | 62 | 2 | 156 | 60 | 3 | 2 | 1 | 1 | 3 | 3 | 1 | 1 | 1 | 1 | 1 | 1 | 1 | 1 |
| 3618 | 62 | 2 | 156 | 57 | 8 | 2 | 1 | 1 | 4 | 1 | 1 | 1 | 1 | 1 | 1 | 1 | 1 | 1 |
| 3619 | 62 | 2 | 156 | 57 | 4 | 2 | 1 | 1 | 4 | 1 | 1 | 1 | 1 | 1 | 1 | 1 | 1 | 1 |
| 3620 | 62 | 2 | 156 | 56 | 8 | 2 | 1 | 1 | 3 | 1 | 1 | 1 | 1 | 1 | 1 | 1 | 1 | 1 |
| 3621 | 62 | 2 | 157 | 68 | 8 | 3 | 1 | 1 | 6 | 1 | 1 | 1 | 1 | 1 | 1 | 1 | 1 | 1 |
| 3622 | 62 | 2 | 157 | 63.5 | 8 | 2 | 1 | 1 | 3 | 1 | 1 | 1 | 1 | 1 | 1 | 1 | 1 | 1 |
| 3623 | 62 | 2 | 158 | 73 | 4 | 3 | 1 | 1 | 3 | 4 | 1 | 1 | 1 | 1 | 1 | 1 | 1 | 1 |
| 3624 | 62 | 2 | 158 | 62 | 8 | 2 | 1 | 1 | 3 | 2 | 1 | 1 | 1 | 1 | 2 | 2 | 1 | 1 |
| 3625 | 62 | 2 | 158 | 59 | 8 | 2 | 1 | 1 | 2 | 3 | 1 | 1 | 1 | 1 | 2 | 1 | 1 | 1 |
| 3626 | 62 | 2 | 158 | 58 | 8 | 2 | 1 | 1 | 3 | 3 | 1 | 1 | 1 | 1 | 1 | 1 | 1 | 1 |
| 3627 | 62 | 2 | 158 | 58 | 3 | 2 | 1 | 1 | 2 | 2 | 1 | 1 | 1 | 1 | 1 | 1 | 1 | 1 |
| 3628 | 62 | 2 | 158 | 56 | 8 | 2 | 1 | 1 | 4 | 1 | 1 | 1 | 1 | 1 | 2 | 1 | 1 | 1 |
| 3629 | 62 | 2 | 158 | 54.5 | 8 | 2 | 1 | 1 | 4 | 2 | 1 | 1 | 2 | 1 | 1 | 1 | 1 | 1 |
| 3630 | 62 | 2 | 158 | 54 | 8 | 2 | 1 | 1 | 3 | 3 | 1 | 1 | 1 | 1 | 1 | 1 | 1 | 1 |
| 3631 | 62 | 2 | 158 | 52.5 | 8 | 2 | 1 | 2 | 4 | 1 | 1 | 2 | 1 | 1 | 1 | 1 | 1 | 1 |
| 3632 | 62 | 2 | 158 | 47.5 | 8 | 2 | 1 | 1 | 5 | 3 | 1 | 1 | 1 | 1 | 1 | 1 | 1 | 1 |
| 3633 | 62 | 2 | 158.5 | 70.5 | 8 | 2 | 1 | 1 | 3 | 1 | 1 | 1 | 1 | 1 | 2 | 1 | 1 | 1 |
| 3634 | 62 | 2 | 158.5 | 53 | 8 | 1 | 1 | 1 | 3 | 1 | 1 | 1 | 1 | 1 | 1 | 1 | 1 | 1 |
| 3635 | 62 | 2 | 158.5 | 50 | 3 | 2 | 1 | 1 | 3 | 4 | 1 | 1 | 1 | 1 | 1 | 1 | 1 | 1 |
| 3636 | 62 | 2 | 159 | 63 | 1 | 2 | 1 | 1 | 4 | 1 | 1 | 1 | 1 | 1 | 1 | 1 | 1 | 1 |
| 3637 | 62 | 2 | 159 | 60 | 8 | 2 | 1 | 1 | 1 | 1 | 1 | 1 | 1 | 1 | 1 | 1 | 1 | 1 |
| 3638 | 62 | 2 | 159 | 60 | 5 | 2 | 1 | 1 | 4 | 1 | 1 | 1 | 1 | 1 | 1 | 1 | 1 | 1 |
| 3639 | 62 | 2 | 159 | 52 | 8 | 2 | 1 | 1 | 4 | 2 | 1 | 1 | 1 | 1 | 1 | 1 | 1 | 1 |
| 3640 | 62 | 2 | 160 | 77.8 | 5 | 2 | 1 | 1 | 3 | 1 | 1 | 1 | 1 | 2 | 1 | 1 | 1 | 1 |
| 3641 | 62 | 2 | 160 | 70 | 8 | 2 | 1 | 1 | 5 | 3 | 1 | 1 | 1 | 1 | 1 | 1 | 1 | 1 |
| 3642 | 62 | 2 | 160 | 63 | 3 | 2 | 1 | 1 | 4 | 1 | 1 | 1 | 1 | 1 | 1 | 1 | 1 | 1 |
| 3643 | 62 | 2 | 160 | 53 | 8 | 2 | 1 | 1 | 3 | 1 | 1 | 1 | 1 | 1 | 2 | 1 | 1 | 1 |
| 3644 | 62 | 2 | 161 | 69 | 8 | 3 | 1 | 1 | 4 | 4 | 1 | 1 | 1 | 1 | 1 | 1 | 1 | 1 |
| 3645 | 62 | 2 | 161 | 66 | 8 | 2 | 1 | 1 | 4 | 4 | 3 | 1 | 1 | 1 | 1 | 1 | 1 | 1 |
| 3646 | 62 | 2 | 161 | 57.5 | 3 | 2 | 1 | 1 | 4 | 1 | 1 | 1 | 1 | 1 | 1 | 1 | 1 | 1 |
| 3647 | 62 | 2 | 161.5 | 58.5 | 8 | 3 | 1 | 1 | 2 | 4 | 1 | 1 | 1 | 1 | 2 | 1 | 1 | 1 |
| 3648 | 62 | 2 | 162 | 60 | 2 | 2 | 1 | 1 | 3 | 1 | 1 | 1 | 1 | 1 | 1 | 1 | 1 | 2 |
| 3649 | 62 | 2 | 162 | 46.5 | 6 | 2 | 1 | 1 | 4 | 1 | 1 | 1 | 1 | 1 | 1 | 1 | 1 | 1 |
| 3650 | 62 | 2 | 165 | 61 | 8 | 2 | 1 | 1 | 1 | 2 | 1 | 1 | 1 | 1 | 1 | 1 | 1 | 1 |
| 3651 | 62 | 2 | 165 | 60 | 6 | 2 | 1 | 1 | 2 | 4 | 1 | 1 | 2 | 1 | 1 | 1 | 1 | 1 |
| 3652 | 62 | 2 | 165 | 52.5 | 2 | 2 | 1 | 1 | 5 | 4 | 1 | 1 | 1 | 1 | 1 | 1 | 1 | 1 |
| 3653 | 62 | 2 | 168 | 60 | 8 | 2 | 2 | 1 | 4 | 4 | 1 | 1 | 1 | 1 | 1 | 1 | 1 | 1 |
| 3654 | 62 | 2 | 174 | 79 | 8 | 2 | 1 | 1 | 4 | 1 | 1 | 1 | 1 | 1 | 1 | 1 | 1 | 1 |
| 3655 | 62 | 1 | 151 | 57.8 | 2 | 2 | 1 | 1 | 4 | 4 | 1 | 1 | 1 | 1 | 1 | 1 | 1 | 1 |
| 3656 | 62 | 1 | 155 | 73 | 6 | 2 | 1 | 2 | 6 | 1 | 1 | 1 | 1 | 1 | 1 | 1 | 1 | 1 |
| 3657 | 62 | 1 | 156 | 69.5 | 8 | 2 | 1 | 1 | 3 | 4 | 1 | 1 | 1 | 1 | 1 | 1 | 1 | 1 |
| 3658 | 62 | 1 | 157 | 63 | 8 | 2 | 1 | 1 | 3 | 1 | 1 | 1 | 1 | 1 | 1 | 1 | 1 | 1 |
| 3659 | 62 | 1 | 157.5 | 56.1 | 8 | 2 | 1 | 1 | 4 | 1 | 1 | 1 | 1 | 1 | 1 | 1 | 1 | 1 |
| 3660 | 62 | 1 | 158 | 52 | 8 | 2 | 1 | 1 | 3 | 4 | 1 | 1 | 1 | 1 | 1 | 1 | 1 | 1 |
| 3661 | 62 | 1 | 160 | 64 | 8 | 2 | 2 | 1 | 3 | 1 | 1 | 1 | 1 | 1 | 2 | 1 | 3 | 1 |
| 3662 | 62 | 1 | 160.5 | 67 | 8 | 2 | 1 | 1 | 3 | 2 | 1 | 1 | 1 | 1 | 1 | 1 | 1 | 1 |
| 3663 | 62 | 1 | 161 | 58 | 2 | 2 | 1 | 1 | 2 | 1 | 1 | 1 | 1 | 1 | 1 | 1 | 3 | 1 |
| 3664 | 62 | 1 | 161 | 54 | 3 | 2 | 1 | 1 | 4 | 4 | 1 | 1 | 1 | 1 | 1 | 1 | 2 | 1 |
| 3665 | 62 | 1 | 162 | 64 | 8 | 2 | 1 | 1 | 4 | 1 | 1 | 1 | 1 | 1 | 1 | 1 | 1 | 1 |
| 3666 | 62 | 1 | 163 | 65 | 8 | 2 | 1 | 1 | 4 | 4 | 1 | 1 | 1 | 1 | 1 | 1 | 1 | 1 |
| 3667 | 62 | 1 | 163 | 53 | 8 | 2 | 1 | 1 | 3 | 3 | 1 | 1 | 1 | 1 | 1 | 1 | 3 | 1 |
| 3668 | 62 | 1 | 163 | 52 | 2 | 2 | 1 | 1 | 3 | 2 | 1 | 1 | 1 | 1 | 2 | 1 | 1 | 1 |
| 3669 | 62 | 1 | 163.5 | 60 | 8 | 2 | 1 | 2 | 4 | 3 | 1 | 1 | 1 | 1 | 1 | 1 | 1 | 1 |
| 3670 | 62 | 1 | 164 | 82 | 8 | 2 | 2 | 1 | 3 | 4 | 1 | 1 | 1 | 1 | 1 | 1 | 3 | 1 |
| 3671 | 62 | 1 | 164 | 55 | 8 | 2 | 1 | 1 | 4 | 3 | 1 | 1 | 1 | 2 | 1 | 1 | 1 | 2 |
| 3672 | 62 | 1 | 164 | 55 | 8 | 2 | 1 | 4 | 2 | 4 | 1 | 1 | 2 | 1 | 1 | 1 | 3 | 1 |
| 3673 | 62 | 1 | 165 | 78.5 | 1 | 2 | 1 | 1 | 5 | 2 | 1 | 1 | 1 | 2 | 2 | 1 | 1 | 1 |
| 3674 | 62 | 1 | 165 | 69 | 4 | 2 | 1 | 2 | 3 | 4 | 1 | 1 | 1 | 1 | 1 | 1 | 3 | 2 |
| 3675 | 62 | 1 | 165 | 60 | 6 | 2 | 1 | 1 | 4 | 1 | 1 | 1 | 1 | 1 | 1 | 1 | 3 | 1 |
| 3676 | 62 | 1 | 165 | 53 | 2 | 2 | 1 | 1 | 4 | 1 | 1 | 1 | 1 | 1 | 1 | 1 | 3 | 1 |
| 3677 | 62 | 1 | 165.9 | 58.1 | 4 | 2 | 1 | 1 | 4 | 4 | 2 | 1 | 1 | 1 | 2 | 1 | 1 | 1 |
| 3678 | 62 | 1 | 166 | 65 | 8 | 2 | 1 | 3 | 3 | 3 | 1 | 1 | 1 | 1 | 1 | 1 | 3 | 1 |
| 3679 | 62 | 1 | 166 | 61 | 8 | 2 | 1 | 1 | 5 | 1 | 1 | 1 | 1 | 1 | 1 | 1 | 2 | 1 |
| 3680 | 62 | 1 | 166 | 55.5 | 8 | 2 | 1 | 1 | 3 | 2 | 1 | 1 | 2 | 1 | 1 | 1 | 3 | 1 |
| 3681 | 62 | 1 | 166 | 55 | 8 | 2 | 1 | 1 | 4 | 2 | 1 | 1 | 1 | 1 | 1 | 1 | 3 | 1 |
| 3682 | 62 | 1 | 166 | 55 | 1 | 2 | 1 | 1 | 3 | 3 | 1 | 1 | 1 | 1 | 1 | 1 | 1 | 1 |
| 3683 | 62 | 1 | 166.5 | 72 | 6 | 2 | 1 | 3 | 4 | 1 | 1 | 1 | 1 | 1 | 1 | 1 | 3 | 1 |
| 3684 | 62 | 1 | 167 | 67 | 3 | 2 | 1 | 1 | 4 | 1 | 1 | 1 | 1 | 1 | 1 | 1 | 2 | 1 |
| 3685 | 62 | 1 | 167.5 | 69.7 | 1 | 2 | 1 | 1 | 3 | 1 | 1 | 1 | 1 | 2 | 1 | 1 | 1 | 1 |
| 3686 | 62 | 1 | 168 | 75 | 8 | 2 | 1 | 1 | 5 | 4 | 1 | 1 | 2 | 1 | 1 | 1 | 1 | 1 |
| 3687 | 62 | 1 | 168 | 75 | 2 | 2 | 1 | 2 | 5 | 1 | 1 | 1 | 2 | 1 | 1 | 1 | 2 | 1 |
| 3688 | 62 | 1 | 168 | 73.3 | 1 | 2 | 1 | 1 | 4 | 3 | 1 | 1 | 1 | 1 | 2 | 1 | 1 | 1 |
| 3689 | 62 | 1 | 168 | 68 | 8 | 2 | 1 | 1 | 4 | 1 | 1 | 1 | 1 | 1 | 1 | 1 | 1 | 1 |
| 3690 | 62 | 1 | 168 | 63.5 | 8 | 4 | 1 | 2 | 3 | 1 | 1 | 2 | 1 | 1 | 1 | 1 | 3 | 1 |
| 3691 | 62 | 1 | 168 | 60 | 4 | 2 | 1 | 1 | 3 | 1 | 1 | 1 | 1 | 1 | 1 | 1 | 1 | 1 |
| 3692 | 62 | 1 | 169 | 69 | 8 | 2 | 1 | 1 | 4 | 2 | 1 | 1 | 1 | 1 | 1 | 1 | 1 | 1 |
| 3693 | 62 | 1 | 169 | 65 | 8 | 2 | 1 | 1 | 4 | 4 | 1 | 1 | 1 | 1 | 1 | 1 | 1 | 1 |
| 3694 | 62 | 1 | 169.5 | 61 | 8 | 2 | 1 | 1 | 3 | 1 | 1 | 1 | 1 | 1 | 1 | 1 | 1 | 1 |
| 3695 | 62 | 1 | 170 | 70 | 8 | 2 | 1 | 1 | 6 | 3 | 1 | 1 | 1 | 1 | 1 | 1 | 1 | 1 |
| 3696 | 62 | 1 | 170 | 62.5 | 8 | 2 | 1 | 1 | 3 | 1 | 1 | 1 | 1 | 1 | 1 | 1 | 3 | 1 |
| 3697 | 62 | 1 | 170 | 62 | 8 | 2 | 1 | 1 | 3 | 4 | 1 | 1 | 1 | 1 | 1 | 1 | 1 | 1 |
| 3698 | 62 | 1 | 171 | 65 | 8 | 2 | 1 | 1 | 2 | 1 | 1 | 1 | 2 | 1 | 1 | 1 | 1 | 1 |
| 3699 | 62 | 1 | 172 | 74 | 8 | 2 | 1 | 1 | 3 | 1 | 1 | 1 | 1 | 1 | 1 | 1 | 1 | 1 |
| 3700 | 62 | 1 | 172 | 67 | 8 | 3 | 2 | 1 | 3 | 2 | 1 | 1 | 2 | 1 | 1 | 1 | 1 | 1 |
| 3701 | 62 | 1 | 173 | 72 | 8 | 2 | 2 | 1 | 3 | 1 | 1 | 1 | 1 | 1 | 1 | 1 | 1 | 1 |
| 3702 | 62 | 1 | 173 | 61 | 4 | 2 | 1 | 1 | 3 | 1 | 1 | 1 | 1 | 1 | 1 | 1 | 2 | 1 |
| 3703 | 62 | 1 | 174.5 | 71 | 8 | 2 | 1 | 1 | 3 | 1 | 1 | 1 | 1 | 1 | 1 | 1 | 1 | 1 |
| 3704 | 62 | 1 | 177 | 66.5 | 8 | 2 | 1 | 1 | 4 | 1 | 1 | 1 | 2 | 1 | 1 | 1 | 3 | 1 |
| 3705 | 63 | 2 | 143 | 55.5 | 4 | 2 | 1 | 1 | 3 | 1 | 1 | 1 | 1 | 1 | 1 | 1 | 1 | 1 |
| 3706 | 63 | 2 | 147 | 49.5 | 3 | 2 | 1 | 1 | 4 | 1 | 1 | 1 | 2 | 1 | 1 | 1 | 1 | 1 |
| 3707 | 63 | 2 | 147 | 43.4 | 8 | 3 | 1 | 1 | 4 | 1 | 1 | 1 | 1 | 1 | 1 | 1 | 1 | 1 |
| 3708 | 63 | 2 | 148 | 52 | 6 | 2 | 1 | 1 | 3 | 4 | 1 | 1 | 1 | 1 | 1 | 1 | 1 | 1 |
| 3709 | 63 | 2 | 149 | 51 | 3 | 2 | 1 | 1 | 4 | 2 | 1 | 1 | 2 | 1 | 1 | 1 | 1 | 1 |
| 3710 | 63 | 2 | 149.5 | 37 | 2 | 2 | 1 | 1 | 3 | 2 | 1 | 1 | 1 | 1 | 2 | 1 | 1 | 1 |
| 3711 | 63 | 2 | 150 | 60.4 | 2 | 2 | 1 | 1 | 4 | 2 | 1 | 1 | 2 | 1 | 2 | 1 | 1 | 1 |
| 3712 | 63 | 2 | 150 | 60 | 8 | 2 | 1 | 1 | 3 | 4 | 1 | 1 | 1 | 2 | 1 | 1 | 1 | 2 |
| 3713 | 63 | 2 | 150 | 56.5 | 8 | 2 | 1 | 1 | 2 | 1 | 1 | 1 | 1 | 1 | 1 | 1 | 1 | 1 |
| 3714 | 63 | 2 | 150 | 47.1 | 3 | 2 | 1 | 1 | 2 | 4 | 1 | 1 | 1 | 1 | 1 | 1 | 1 | 1 |
| 3715 | 63 | 2 | 150 | 43 | 8 | 2 | 1 | 2 | 3 | 3 | 1 | 1 | 1 | 1 | 1 | 1 | 1 | 1 |
| 3716 | 63 | 2 | 150 | 41 | 2 | 1 | 1 | 1 | 2 | 2 | 1 | 1 | 1 | 1 | 1 | 1 | 1 | 1 |
| 3717 | 63 | 2 | 151 | 49 | 8 | 2 | 1 | 1 | 3 | 1 | 1 | 1 | 1 | 1 | 1 | 1 | 1 | 1 |
| 3718 | 63 | 2 | 152 | 65 | 8 | 2 | 1 | 1 | 4 | 3 | 1 | 1 | 1 | 1 | 1 | 1 | 1 | 1 |
| 3719 | 63 | 2 | 152 | 53 | 6 | 3 | 1 | 1 | 4 | 1 | 1 | 1 | 1 | 1 | 2 | 1 | 1 | 1 |
| 3720 | 63 | 2 | 153 | 62.1 | 2 | 2 | 1 | 1 | 3 | 1 | 1 | 1 | 2 | 1 | 1 | 1 | 1 | 1 |
| 3721 | 63 | 2 | 153 | 56 | 3 | 2 | 1 | 1 | 4 | 4 | 1 | 1 | 1 | 1 | 1 | 1 | 1 | 1 |
| 3722 | 63 | 2 | 153 | 54.5 | 6 | 2 | 1 | 1 | 3 | 4 | 1 | 1 | 1 | 2 | 1 | 1 | 1 | 2 |
| 3723 | 63 | 2 | 153 | 54 | 8 | 2 | 1 | 1 | 3 | 1 | 1 | 1 | 2 | 1 | 2 | 1 | 1 | 1 |
| 3724 | 63 | 2 | 153 | 53 | 8 | 3 | 1 | 1 | 4 | 1 | 1 | 1 | 2 | 1 | 1 | 1 | 1 | 1 |
| 3725 | 63 | 2 | 153 | 48 | 8 | 2 | 1 | 1 | 4 | 1 | 1 | 1 | 1 | 1 | 2 | 1 | 1 | 1 |
| 3726 | 63 | 2 | 153.5 | 55.4 | 8 | 2 | 1 | 1 | 3 | 4 | 1 | 1 | 2 | 1 | 1 | 1 | 1 | 1 |
| 3727 | 63 | 2 | 154 | 52 | 8 | 2 | 1 | 1 | 3 | 3 | 1 | 1 | 1 | 1 | 1 | 1 | 1 | 1 |
| 3728 | 63 | 2 | 155 | 66 | 3 | 2 | 1 | 1 | 3 | 1 | 1 | 1 | 1 | 1 | 2 | 1 | 1 | 2 |
| 3729 | 63 | 2 | 155 | 56.5 | 8 | 2 | 1 | 1 | 3 | 2 | 1 | 1 | 1 | 2 | 1 | 1 | 1 | 2 |
| 3730 | 63 | 2 | 155 | 50 | 8 | 2 | 1 | 1 | 3 | 4 | 1 | 1 | 1 | 1 | 2 | 1 | 1 | 1 |
| 3731 | 63 | 2 | 155 | 49 | 8 | 2 | 1 | 1 | 2 | 2 | 1 | 1 | 1 | 1 | 2 | 1 | 1 | 1 |
| 3732 | 63 | 2 | 156 | 63 | 8 | 3 | 1 | 1 | 4 | 4 | 1 | 1 | 1 | 1 | 1 | 1 | 1 | 1 |
| 3733 | 63 | 2 | 156 | 62 | 8 | 3 | 1 | 1 | 3 | 1 | 1 | 1 | 1 | 1 | 2 | 1 | 1 | 1 |
| 3734 | 63 | 2 | 156 | 50 | 8 | 2 | 1 | 1 | 4 | 1 | 1 | 1 | 1 | 1 | 2 | 1 | 1 | 1 |
| 3735 | 63 | 2 | 156.5 | 57.5 | 8 | 2 | 1 | 1 | 3 | 2 | 1 | 1 | 1 | 1 | 1 | 1 | 1 | 1 |
| 3736 | 63 | 2 | 156.5 | 55 | 8 | 2 | 1 | 1 | 3 | 1 | 1 | 1 | 1 | 1 | 1 | 1 | 1 | 1 |
| 3737 | 63 | 2 | 157 | 67 | 8 | 2 | 1 | 1 | 3 | 2 | 1 | 1 | 1 | 1 | 1 | 1 | 1 | 1 |
| 3738 | 63 | 2 | 157 | 64 | 3 | 2 | 1 | 1 | 4 | 1 | 1 | 1 | 2 | 1 | 1 | 1 | 1 | 1 |
| 3739 | 63 | 2 | 157 | 60 | 8 | 2 | 1 | 1 | 2 | 1 | 1 | 1 | 1 | 1 | 1 | 1 | 1 | 1 |
| 3740 | 63 | 2 | 157 | 59 | 2 | 2 | 1 | 1 | 4 | 4 | 1 | 1 | 1 | 1 | 1 | 1 | 1 | 1 |
| 3741 | 63 | 2 | 157 | 55 | 8 | 2 | 1 | 1 | 2 | 3 | 1 | 1 | 1 | 1 | 1 | 1 | 1 | 1 |
| 3742 | 63 | 2 | 157 | 54 | 6 | 2 | 1 | 1 | 3 | 4 | 1 | 1 | 1 | 1 | 2 | 1 | 1 | 1 |
| 3743 | 63 | 2 | 157.5 | 70 | 3 | 2 | 1 | 1 | 4 | 1 | 1 | 1 | 1 | 1 | 1 | 1 | 1 | 1 |
| 3744 | 63 | 2 | 158 | 63.5 | 8 | 2 | 1 | 1 | 2 | 2 | 1 | 1 | 1 | 1 | 1 | 1 | 1 | 1 |
| 3745 | 63 | 2 | 158 | 60 | 4 | 4 | 1 | 2 | 6 | 1 | 1 | 1 | 1 | 1 | 1 | 1 | 1 | 1 |
| 3746 | 63 | 2 | 158 | 51 | 8 | 2 | 1 | 1 | 4 | 4 | 1 | 1 | 2 | 1 | 1 | 1 | 2 | 1 |
| 3747 | 63 | 2 | 158 | 49.5 | 8 | 2 | 1 | 1 | 4 | 1 | 1 | 1 | 1 | 1 | 2 | 1 | 1 | 1 |
| 3748 | 63 | 2 | 158 | 45 | 6 | 4 | 1 | 1 | 3 | 4 | 1 | 1 | 1 | 1 | 1 | 1 | 1 | 1 |
| 3749 | 63 | 2 | 158.5 | 49.6 | 2 | 2 | 1 | 1 | 6 | 2 | 1 | 1 | 2 | 1 | 1 | 1 | 1 | 2 |
| 3750 | 63 | 2 | 159 | 83 | 8 | 2 | 1 | 1 | 3 | 2 | 1 | 1 | 1 | 1 | 1 | 1 | 1 | 1 |
| 3751 | 63 | 2 | 159 | 50.5 | 4 | 2 | 1 | 1 | 4 | 2 | 1 | 1 | 1 | 1 | 1 | 1 | 1 | 1 |
| 3752 | 63 | 2 | 160 | 77.5 | 8 | 2 | 1 | 1 | 3 | 2 | 1 | 2 | 1 | 1 | 2 | 1 | 1 | 1 |
| 3753 | 63 | 2 | 160 | 65 | 2 | 2 | 1 | 1 | 2 | 3 | 1 | 1 | 1 | 1 | 1 | 1 | 1 | 1 |
| 3754 | 63 | 2 | 160 | 62 | 6 | 2 | 1 | 1 | 3 | 2 | 1 | 1 | 1 | 1 | 1 | 1 | 1 | 1 |
| 3755 | 63 | 2 | 160 | 53.5 | 8 | 2 | 1 | 1 | 3 | 1 | 1 | 1 | 1 | 1 | 2 | 1 | 1 | 1 |
| 3756 | 63 | 2 | 160 | 51 | 6 | 2 | 1 | 1 | 3 | 1 | 1 | 1 | 2 | 1 | 1 | 1 | 1 | 1 |
| 3757 | 63 | 2 | 160 | 50 | 1 | 2 | 2 | 1 | 6 | 3 | 1 | 1 | 1 | 1 | 1 | 1 | 1 | 1 |
| 3758 | 63 | 2 | 160.5 | 62 | 8 | 2 | 1 | 1 | 4 | 2 | 1 | 1 | 2 | 1 | 1 | 1 | 1 | 1 |
| 3759 | 63 | 2 | 162 | 57 | 8 | 2 | 1 | 1 | 6 | 1 | 1 | 1 | 1 | 1 | 1 | 1 | 1 | 1 |
| 3760 | 63 | 2 | 162 | 56.5 | 3 | 2 | 1 | 2 | 4 | 4 | 1 | 1 | 1 | 1 | 1 | 1 | 1 | 1 |
| 3761 | 63 | 2 | 163 | 71.5 | 8 | 2 | 1 | 2 | 3 | 4 | 1 | 2 | 1 | 1 | 2 | 1 | 1 | 1 |
| 3762 | 63 | 2 | 163 | 60 | 8 | 2 | 1 | 1 | 3 | 2 | 1 | 1 | 2 | 1 | 1 | 1 | 1 | 1 |
| 3763 | 63 | 2 | 164 | 68 | 8 | 2 | 1 | 1 | 4 | 4 | 1 | 1 | 1 | 1 | 1 | 1 | 1 | 2 |
| 3764 | 63 | 1 | 150.5 | 62 | 8 | 2 | 1 | 1 | 5 | 1 | 1 | 1 | 1 | 1 | 1 | 1 | 2 | 1 |
| 3765 | 63 | 1 | 151 | 63 | 8 | 2 | 1 | 1 | 2 | 1 | 1 | 1 | 1 | 1 | 1 | 1 | 1 | 1 |
| 3766 | 63 | 1 | 158.5 | 76.3 | 8 | 2 | 1 | 1 | 5 | 4 | 1 | 1 | 1 | 1 | 1 | 1 | 1 | 1 |
| 3767 | 63 | 1 | 161 | 68 | 8 | 2 | 1 | 2 | 5 | 1 | 1 | 1 | 1 | 1 | 2 | 1 | 3 | 1 |
| 3768 | 63 | 1 | 161 | 51 | 8 | 2 | 1 | 1 | 3 | 2 | 1 | 1 | 1 | 1 | 1 | 1 | 1 | 1 |
| 3769 | 63 | 1 | 162 | 72 | 8 | 2 | 1 | 1 | 3 | 1 | 1 | 1 | 1 | 1 | 2 | 1 | 1 | 1 |
| 3770 | 63 | 1 | 163 | 47 | 1 | 2 | 1 | 1 | 3 | 3 | 1 | 1 | 1 | 1 | 1 | 1 | 3 | 1 |
| 3771 | 63 | 1 | 164 | 81.5 | 3 | 2 | 2 | 3 | 5 | 1 | 1 | 1 | 1 | 1 | 1 | 1 | 2 | 1 |
| 3772 | 63 | 1 | 164 | 59 | 6 | 2 | 1 | 3 | 3 | 1 | 1 | 1 | 1 | 1 | 1 | 1 | 3 | 1 |
| 3773 | 63 | 1 | 164.5 | 64.5 | 8 | 1 | 1 | 1 | 4 | 1 | 1 | 1 | 1 | 1 | 2 | 1 | 1 | 1 |
| 3774 | 63 | 1 | 165 | 70 | 8 | 2 | 1 | 2 | 3 | 4 | 1 | 1 | 1 | 1 | 1 | 1 | 1 | 1 |
| 3775 | 63 | 1 | 165 | 70 | 8 | 2 | 1 | 1 | 4 | 3 | 1 | 1 | 1 | 1 | 1 | 1 | 2 | 1 |
| 3776 | 63 | 1 | 165 | 57.5 | 5 | 2 | 1 | 1 | 6 | 2 | 1 | 1 | 1 | 1 | 1 | 1 | 1 | 1 |
| 3777 | 63 | 1 | 165 | 55 | 8 | 2 | 1 | 2 | 3 | 3 | 1 | 1 | 1 | 1 | 1 | 1 | 3 | 1 |
| 3778 | 63 | 1 | 166 | 77.8 | 3 | 2 | 1 | 1 | 4 | 2 | 2 | 1 | 2 | 1 | 1 | 1 | 2 | 1 |
| 3779 | 63 | 1 | 166.5 | 74.5 | 2 | 2 | 1 | 3 | 3 | 2 | 1 | 1 | 1 | 1 | 1 | 1 | 3 | 1 |
| 3780 | 63 | 1 | 167 | 63 | 8 | 2 | 1 | 1 | 3 | 4 | 1 | 1 | 2 | 1 | 1 | 1 | 1 | 1 |
| 3781 | 63 | 1 | 167 | 57 | 8 | 2 | 1 | 1 | 1 | 1 | 1 | 1 | 1 | 1 | 1 | 1 | 1 | 1 |
| 3782 | 63 | 1 | 167 | 54 | 2 | 2 | 1 | 2 | 4 | 4 | 1 | 1 | 1 | 1 | 1 | 1 | 3 | 1 |
| 3783 | 63 | 1 | 168 | 62 | 3 | 2 | 1 | 1 | 4 | 1 | 1 | 1 | 1 | 1 | 1 | 1 | 1 | 1 |
| 3784 | 63 | 1 | 168 | 58 | 6 | 2 | 1 | 1 | 4 | 1 | 1 | 1 | 1 | 1 | 1 | 1 | 2 | 1 |
| 3785 | 63 | 1 | 168 | 52.5 | 8 | 2 | 1 | 1 | 3 | 4 | 1 | 1 | 1 | 1 | 1 | 1 | 2 | 1 |
| 3786 | 63 | 1 | 168.8 | 73 | 4 | 2 | 1 | 1 | 3 | 1 | 1 | 1 | 1 | 1 | 1 | 1 | 1 | 1 |
| 3787 | 63 | 1 | 169 | 79 | 8 | 2 | 1 | 1 | 3 | 1 | 1 | 1 | 1 | 1 | 1 | 1 | 1 | 1 |
| 3788 | 63 | 1 | 169 | 66 | 4 | 2 | 1 | 1 | 3 | 2 | 1 | 1 | 2 | 1 | 2 | 1 | 1 | 1 |
| 3789 | 63 | 1 | 169.5 | 64.5 | 3 | 2 | 1 | 1 | 5 | 1 | 1 | 1 | 2 | 1 | 2 | 1 | 3 | 1 |
| 3790 | 63 | 1 | 170 | 80 | 6 | 2 | 1 | 1 | 3 | 2 | 1 | 1 | 1 | 1 | 2 | 1 | 1 | 1 |
| 3791 | 63 | 1 | 170 | 76 | 8 | 2 | 1 | 1 | 5 | 1 | 1 | 1 | 2 | 1 | 1 | 1 | 1 | 1 |
| 3792 | 63 | 1 | 170 | 72.5 | 8 | 2 | 1 | 2 | 3 | 2 | 1 | 1 | 1 | 1 | 2 | 1 | 2 | 1 |
| 3793 | 63 | 1 | 170 | 68 | 8 | 2 | 1 | 1 | 5 | 2 | 1 | 1 | 1 | 1 | 1 | 2 | 2 | 1 |
| 3794 | 63 | 1 | 170 | 55 | 8 | 2 | 1 | 1 | 3 | 3 | 1 | 1 | 1 | 1 | 1 | 1 | 1 | 1 |
| 3795 | 63 | 1 | 171 | 70 | 8 | 2 | 1 | 2 | 3 | 4 | 1 | 1 | 1 | 1 | 1 | 1 | 3 | 1 |
| 3796 | 63 | 1 | 172 | 74 | 3 | 2 | 1 | 1 | 4 | 3 | 1 | 1 | 2 | 1 | 1 | 1 | 1 | 1 |
| 3797 | 63 | 1 | 173 | 90 | 8 | 2 | 1 | 1 | 4 | 3 | 1 | 1 | 1 | 1 | 1 | 1 | 1 | 1 |
| 3798 | 63 | 1 | 173 | 65 | 5 | 2 | 2 | 1 | 3 | 2 | 1 | 1 | 1 | 1 | 1 | 1 | 1 | 1 |
| 3799 | 63 | 1 | 173 | 65 | 4 | 2 | 1 | 4 | 2 | 3 | 1 | 1 | 1 | 1 | 2 | 1 | 3 | 1 |
| 3800 | 63 | 1 | 174 | 67 | 8 | 2 | 1 | 1 | 3 | 3 | 2 | 1 | 1 | 1 | 1 | 1 | 1 | 1 |
| 3801 | 63 | 1 | 176 | 95 | 8 | 2 | 1 | 2 | 3 | 1 | 1 | 1 | 1 | 1 | 1 | 1 | 1 | 1 |
| 3802 | 63 | 1 | 176 | 80 | 8 | 2 | 1 | 1 | 2 | 3 | 1 | 1 | 1 | 1 | 1 | 1 | 1 | 1 |
| 3803 | 63 | 1 | 177 | 59.5 | 8 | 2 | 1 | 1 | 3 | 1 | 1 | 1 | 1 | 2 | 1 | 1 | 2 | 2 |
| 3804 | 63 | 1 | 178 | 81 | 8 | 2 | 1 | 1 | 3 | 4 | 1 | 1 | 1 | 1 | 1 | 1 | 3 | 1 |
| 3805 | 63 | 1 | 178 | 66 | 8 | 2 | 1 | 1 | 4 | 3 | 1 | 1 | 1 | 1 | 1 | 1 | 1 | 1 |
| 3806 | 63 | 1 | 181 | 84.5 | 8 | 2 | 1 | 3 | 3 | 1 | 1 | 1 | 1 | 1 | 1 | 1 | 3 | 2 |
| 3807 | 64 | 2 | 143 | 45 | 2 | 2 | 1 | 1 | 5 | 1 | 1 | 1 | 1 | 1 | 2 | 2 | 1 | 1 |
| 3808 | 64 | 2 | 145 | 50 | 8 | 3 | 1 | 1 | 2 | 1 | 1 | 1 | 1 | 1 | 1 | 1 | 1 | 1 |
| 3809 | 64 | 2 | 146 | 50 | 3 | 2 | 1 | 1 | 3 | 4 | 1 | 1 | 1 | 1 | 2 | 1 | 1 | 1 |
| 3810 | 64 | 2 | 146 | 48 | 8 | 2 | 1 | 1 | 4 | 1 | 1 | 1 | 1 | 1 | 1 | 1 | 1 | 1 |
| 3811 | 64 | 2 | 148 | 55 | 8 | 2 | 1 | 1 | 3 | 4 | 1 | 2 | 1 | 1 | 1 | 1 | 1 | 1 |
| 3812 | 64 | 2 | 148 | 47.5 | 8 | 2 | 1 | 1 | 3 | 1 | 1 | 1 | 1 | 1 | 1 | 1 | 1 | 1 |
| 3813 | 64 | 2 | 148 | 45 | 2 | 1 | 1 | 1 | 5 | 2 | 1 | 1 | 2 | 1 | 1 | 1 | 1 | 1 |
| 3814 | 64 | 2 | 148 | 31.5 | 8 | 2 | 1 | 1 | 3 | 2 | 1 | 1 | 1 | 1 | 1 | 1 | 1 | 1 |
| 3815 | 64 | 2 | 149 | 59.5 | 6 | 2 | 1 | 1 | 3 | 1 | 1 | 1 | 1 | 1 | 1 | 1 | 1 | 1 |
| 3816 | 64 | 2 | 149 | 52.8 | 8 | 2 | 1 | 1 | 2 | 1 | 1 | 1 | 1 | 1 | 1 | 1 | 1 | 1 |
| 3817 | 64 | 2 | 149 | 51.5 | 8 | 2 | 1 | 1 | 2 | 1 | 1 | 1 | 1 | 1 | 1 | 1 | 1 | 1 |
| 3818 | 64 | 2 | 149.5 | 44.2 | 8 | 2 | 1 | 1 | 2 | 1 | 1 | 1 | 1 | 1 | 1 | 1 | 1 | 1 |
| 3819 | 64 | 2 | 150 | 65 | 8 | 2 | 1 | 1 | 2 | 1 | 1 | 1 | 1 | 1 | 1 | 1 | 1 | 1 |
| 3820 | 64 | 2 | 150 | 60 | 4 | 2 | 1 | 1 | 4 | 1 | 1 | 1 | 1 | 1 | 1 | 1 | 1 | 1 |
| 3821 | 64 | 2 | 150 | 59.6 | 8 | 4 | 1 | 1 | 1 | 1 | 1 | 1 | 1 | 1 | 1 | 1 | 1 | 1 |
| 3822 | 64 | 2 | 150 | 50 | 8 | 3 | 1 | 1 | 3 | 2 | 1 | 1 | 1 | 1 | 1 | 1 | 1 | 1 |
| 3823 | 64 | 2 | 150 | 46 | 3 | 2 | 1 | 1 | 3 | 4 | 1 | 1 | 1 | 1 | 1 | 1 | 1 | 1 |
| 3824 | 64 | 2 | 150 | 43 | 8 | 2 | 1 | 1 | 2 | 4 | 1 | 1 | 1 | 1 | 1 | 1 | 1 | 1 |
| 3825 | 64 | 2 | 152 | 58.4 | 6 | 2 | 1 | 1 | 4 | 1 | 1 | 1 | 1 | 1 | 1 | 1 | 1 | 1 |
| 3826 | 64 | 2 | 152 | 52.8 | 3 | 2 | 1 | 1 | 2 | 1 | 1 | 1 | 1 | 1 | 1 | 1 | 1 | 2 |
| 3827 | 64 | 2 | 152 | 46 | 8 | 2 | 1 | 1 | 4 | 2 | 1 | 1 | 1 | 1 | 1 | 1 | 1 | 1 |
| 3828 | 64 | 2 | 153 | 70 | 8 | 2 | 1 | 1 | 2 | 1 | 1 | 1 | 1 | 1 | 1 | 1 | 1 | 1 |
| 3829 | 64 | 2 | 153 | 50 | 8 | 2 | 1 | 2 | 2 | 4 | 1 | 1 | 1 | 1 | 1 | 1 | 3 | 1 |
| 3830 | 64 | 2 | 154 | 58 | 8 | 2 | 1 | 1 | 2 | 4 | 1 | 1 | 1 | 1 | 1 | 1 | 1 | 1 |
| 3831 | 64 | 2 | 154.2 | 62.4 | 8 | 2 | 1 | 1 | 3 | 3 | 2 | 1 | 1 | 1 | 1 | 1 | 1 | 1 |
| 3832 | 64 | 2 | 154.4 | 60.6 | 3 | 2 | 1 | 1 | 4 | 1 | 1 | 1 | 1 | 1 | 2 | 1 | 1 | 1 |
| 3833 | 64 | 2 | 155 | 64 | 8 | 2 | 1 | 1 | 3 | 1 | 1 | 1 | 1 | 2 | 1 | 2 | 1 | 1 |
| 3834 | 64 | 2 | 155 | 60 | 1 | 2 | 1 | 1 | 5 | 4 | 1 | 1 | 1 | 1 | 1 | 1 | 1 | 1 |
| 3835 | 64 | 2 | 155 | 56.5 | 8 | 2 | 1 | 1 | 3 | 1 | 1 | 1 | 1 | 1 | 1 | 1 | 1 | 1 |
| 3836 | 64 | 2 | 155 | 53 | 8 | 3 | 1 | 1 | 3 | 1 | 1 | 1 | 1 | 1 | 1 | 1 | 1 | 1 |
| 3837 | 64 | 2 | 156 | 78.6 | 8 | 3 | 1 | 1 | 3 | 1 | 1 | 1 | 1 | 1 | 1 | 1 | 1 | 1 |
| 3838 | 64 | 2 | 156 | 73 | 8 | 2 | 1 | 1 | 2 | 1 | 1 | 1 | 1 | 1 | 1 | 1 | 1 | 1 |
| 3839 | 64 | 2 | 156 | 57 | 8 | 2 | 1 | 2 | 2 | 1 | 1 | 1 | 1 | 1 | 1 | 1 | 1 | 1 |
| 3840 | 64 | 2 | 156 | 56 | 8 | 2 | 1 | 1 | 1 | 4 | 1 | 1 | 1 | 1 | 1 | 1 | 1 | 2 |
| 3841 | 64 | 2 | 156 | 45.5 | 8 | 3 | 1 | 1 | 3 | 3 | 1 | 1 | 2 | 1 | 1 | 1 | 1 | 1 |
| 3842 | 64 | 2 | 156.5 | 72 | 3 | 2 | 1 | 1 | 4 | 2 | 1 | 1 | 2 | 1 | 2 | 1 | 1 | 1 |
| 3843 | 64 | 2 | 157 | 65 | 4 | 2 | 1 | 1 | 3 | 2 | 1 | 1 | 1 | 1 | 2 | 2 | 1 | 1 |
| 3844 | 64 | 2 | 157 | 60 | 6 | 2 | 1 | 1 | 2 | 1 | 1 | 1 | 1 | 1 | 2 | 1 | 1 | 1 |
| 3845 | 64 | 2 | 157 | 55 | 8 | 2 | 1 | 1 | 2 | 4 | 1 | 1 | 1 | 1 | 1 | 1 | 1 | 1 |
| 3846 | 64 | 2 | 157.6 | 56 | 2 | 2 | 1 | 1 | 5 | 1 | 1 | 1 | 1 | 1 | 1 | 1 | 1 | 1 |
| 3847 | 64 | 2 | 158 | 60 | 8 | 2 | 1 | 1 | 4 | 1 | 1 | 1 | 2 | 1 | 1 | 1 | 1 | 1 |
| 3848 | 64 | 2 | 158 | 57.8 | 8 | 2 | 1 | 1 | 4 | 2 | 1 | 1 | 2 | 1 | 2 | 1 | 1 | 1 |
| 3849 | 64 | 2 | 158 | 53 | 1 | 2 | 1 | 1 | 4 | 1 | 1 | 1 | 1 | 1 | 2 | 1 | 1 | 1 |
| 3850 | 64 | 2 | 158 | 52.2 | 8 | 2 | 1 | 1 | 3 | 1 | 1 | 1 | 1 | 2 | 1 | 1 | 1 | 2 |
| 3851 | 64 | 2 | 158.5 | 73 | 8 | 2 | 1 | 1 | 4 | 2 | 2 | 1 | 1 | 1 | 2 | 1 | 1 | 1 |
| 3852 | 64 | 2 | 159 | 66.5 | 4 | 2 | 1 | 1 | 2 | 2 | 1 | 1 | 1 | 1 | 1 | 1 | 1 | 1 |
| 3853 | 64 | 2 | 159 | 52 | 8 | 2 | 1 | 1 | 4 | 1 | 1 | 1 | 1 | 1 | 1 | 1 | 1 | 1 |
| 3854 | 64 | 2 | 160 | 65 | 8 | 2 | 1 | 1 | 4 | 1 | 1 | 1 | 1 | 1 | 1 | 1 | 1 | 1 |
| 3855 | 64 | 2 | 160 | 56 | 8 | 2 | 1 | 1 | 3 | 3 | 1 | 1 | 1 | 1 | 2 | 1 | 1 | 1 |
| 3856 | 64 | 2 | 160 | 55 | 8 | 2 | 1 | 1 | 3 | 1 | 1 | 1 | 1 | 1 | 1 | 1 | 1 | 1 |
| 3857 | 64 | 2 | 161 | 69.6 | 2 | 2 | 1 | 1 | 3 | 2 | 1 | 1 | 2 | 1 | 2 | 1 | 1 | 1 |
| 3858 | 64 | 2 | 161 | 60 | 8 | 2 | 1 | 1 | 4 | 1 | 1 | 1 | 1 | 1 | 1 | 1 | 1 | 1 |
| 3859 | 64 | 2 | 162 | 73.4 | 8 | 2 | 1 | 1 | 3 | 1 | 1 | 1 | 1 | 1 | 1 | 1 | 1 | 1 |
| 3860 | 64 | 2 | 162 | 65 | 8 | 2 | 1 | 1 | 4 | 4 | 1 | 1 | 1 | 1 | 1 | 1 | 1 | 1 |
| 3861 | 64 | 2 | 163 | 54 | 8 | 2 | 1 | 1 | 6 | 4 | 1 | 1 | 1 | 1 | 1 | 1 | 1 | 1 |
| 3862 | 64 | 2 | 163 | 53 | 8 | 2 | 1 | 1 | 2 | 1 | 1 | 1 | 1 | 1 | 1 | 1 | 1 | 1 |
| 3863 | 64 | 2 | 165 | 62 | 3 | 2 | 1 | 1 | 4 | 4 | 1 | 1 | 1 | 1 | 1 | 1 | 1 | 1 |
| 3864 | 64 | 2 | 165 | 60 | 6 | 2 | 1 | 1 | 3 | 4 | 1 | 1 | 1 | 1 | 1 | 1 | 1 | 1 |
| 3865 | 64 | 1 | 130 | 23.5 | 8 | 1 | 1 | 1 | 2 | 1 | 1 | 1 | 2 | 1 | 1 | 1 | 1 | 1 |
| 3866 | 64 | 1 | 140 | 48 | 2 | 2 | 1 | 1 | 3 | 3 | 1 | 1 | 2 | 1 | 2 | 1 | 1 | 1 |
| 3867 | 64 | 1 | 153 | 52.5 | 4 | 2 | 1 | 1 | 3 | 1 | 1 | 1 | 1 | 1 | 2 | 1 | 1 | 1 |
| 3868 | 64 | 1 | 153.5 | 45.5 | 8 | 2 | 1 | 1 | 2 | 2 | 1 | 1 | 1 | 1 | 1 | 1 | 3 | 1 |
| 3869 | 64 | 1 | 155 | 60 | 2 | 2 | 2 | 1 | 3 | 3 | 1 | 1 | 1 | 1 | 1 | 1 | 2 | 1 |
| 3870 | 64 | 1 | 157 | 70 | 8 | 2 | 1 | 2 | 4 | 2 | 1 | 1 | 1 | 1 | 1 | 1 | 3 | 1 |
| 3871 | 64 | 1 | 157 | 61.5 | 8 | 2 | 1 | 1 | 4 | 1 | 1 | 1 | 1 | 1 | 1 | 1 | 1 | 1 |
| 3872 | 64 | 1 | 157 | 60 | 2 | 2 | 1 | 1 | 3 | 2 | 1 | 1 | 1 | 1 | 1 | 1 | 1 | 1 |
| 3873 | 64 | 1 | 157 | 56.5 | 8 | 2 | 1 | 1 | 4 | 1 | 1 | 1 | 1 | 1 | 2 | 1 | 3 | 1 |
| 3874 | 64 | 1 | 157.5 | 64 | 1 | 2 | 2 | 1 | 5 | 2 | 1 | 1 | 2 | 1 | 1 | 1 | 1 | 1 |
| 3875 | 64 | 1 | 159 | 64 | 1 | 2 | 1 | 1 | 3 | 1 | 1 | 1 | 1 | 1 | 1 | 1 | 1 | 2 |
| 3876 | 64 | 1 | 159.5 | 39 | 2 | 2 | 1 | 1 | 3 | 1 | 1 | 1 | 2 | 1 | 2 | 1 | 1 | 1 |
| 3877 | 64 | 1 | 160 | 72.5 | 8 | 2 | 1 | 1 | 4 | 1 | 1 | 1 | 1 | 2 | 2 | 2 | 1 | 2 |
| 3878 | 64 | 1 | 160 | 56 | 2 | 2 | 1 | 1 | 4 | 1 | 1 | 1 | 1 | 1 | 1 | 1 | 1 | 1 |
| 3879 | 64 | 1 | 160 | 55 | 8 | 2 | 1 | 1 | 3 | 4 | 1 | 1 | 1 | 1 | 1 | 1 | 1 | 1 |
| 3880 | 64 | 1 | 160 | 55 | 1 | 2 | 1 | 1 | 4 | 4 | 1 | 1 | 1 | 1 | 1 | 1 | 2 | 1 |
| 3881 | 64 | 1 | 162 | 72 | 8 | 2 | 1 | 3 | 4 | 1 | 1 | 1 | 1 | 1 | 1 | 1 | 3 | 1 |
| 3882 | 64 | 1 | 162 | 62.5 | 3 | 2 | 1 | 1 | 4 | 1 | 1 | 1 | 1 | 1 | 1 | 1 | 1 | 1 |
| 3883 | 64 | 1 | 162 | 51 | 2 | 2 | 1 | 1 | 3 | 4 | 1 | 1 | 1 | 1 | 1 | 1 | 3 | 1 |
| 3884 | 64 | 1 | 162.5 | 73.6 | 8 | 2 | 1 | 1 | 3 | 2 | 1 | 1 | 2 | 1 | 1 | 1 | 3 | 1 |
| 3885 | 64 | 1 | 163 | 60 | 8 | 2 | 1 | 1 | 4 | 4 | 1 | 1 | 1 | 1 | 1 | 1 | 1 | 1 |
| 3886 | 64 | 1 | 163 | 59 | 4 | 2 | 1 | 1 | 3 | 4 | 1 | 1 | 1 | 1 | 1 | 1 | 1 | 1 |
| 3887 | 64 | 1 | 163 | 48.2 | 8 | 2 | 1 | 1 | 4 | 1 | 1 | 1 | 1 | 1 | 1 | 1 | 2 | 2 |
| 3888 | 64 | 1 | 163.5 | 61 | 8 | 2 | 1 | 1 | 2 | 1 | 1 | 1 | 1 | 1 | 1 | 1 | 1 | 1 |
| 3889 | 64 | 1 | 163.5 | 58.8 | 6 | 2 | 1 | 1 | 3 | 1 | 1 | 1 | 1 | 1 | 1 | 1 | 1 | 1 |
| 3890 | 64 | 1 | 164 | 70 | 4 | 2 | 1 | 1 | 4 | 2 | 1 | 1 | 1 | 1 | 1 | 1 | 1 | 1 |
| 3891 | 64 | 1 | 164 | 67 | 3 | 2 | 1 | 2 | 3 | 1 | 1 | 1 | 1 | 1 | 1 | 1 | 1 | 1 |
| 3892 | 64 | 1 | 164 | 66 | 8 | 4 | 1 | 1 | 3 | 1 | 1 | 1 | 1 | 1 | 2 | 1 | 1 | 1 |
| 3893 | 64 | 1 | 164 | 60.4 | 4 | 2 | 1 | 4 | 3 | 1 | 1 | 1 | 1 | 1 | 1 | 1 | 1 | 1 |
| 3894 | 64 | 1 | 164 | 51.3 | 8 | 2 | 1 | 1 | 4 | 1 | 1 | 1 | 1 | 1 | 1 | 1 | 3 | 1 |
| 3895 | 64 | 1 | 164.5 | 77 | 3 | 2 | 1 | 2 | 4 | 1 | 1 | 1 | 1 | 1 | 1 | 1 | 3 | 1 |
| 3896 | 64 | 1 | 165 | 80 | 8 | 2 | 1 | 1 | 3 | 2 | 1 | 1 | 1 | 1 | 1 | 1 | 1 | 1 |
| 3897 | 64 | 1 | 165 | 74 | 8 | 2 | 1 | 1 | 4 | 1 | 1 | 1 | 2 | 1 | 1 | 1 | 2 | 1 |
| 3898 | 64 | 1 | 165 | 62.5 | 8 | 2 | 1 | 1 | 2 | 4 | 1 | 1 | 1 | 1 | 1 | 1 | 3 | 1 |
| 3899 | 64 | 1 | 165 | 60 | 4 | 2 | 1 | 1 | 4 | 1 | 1 | 1 | 1 | 1 | 1 | 1 | 1 | 1 |
| 3900 | 64 | 1 | 165 | 52 | 8 | 2 | 1 | 1 | 3 | 1 | 1 | 1 | 1 | 1 | 1 | 1 | 1 | 1 |
| 3901 | 64 | 1 | 166 | 81.2 | 3 | 2 | 1 | 1 | 4 | 1 | 1 | 1 | 1 | 1 | 1 | 1 | 1 | 1 |
| 3902 | 64 | 1 | 166 | 70 | 2 | 2 | 1 | 1 | 3 | 4 | 1 | 1 | 1 | 1 | 1 | 1 | 3 | 1 |
| 3903 | 64 | 1 | 166 | 70 | 1 | 2 | 1 | 1 | 3 | 4 | 1 | 1 | 1 | 1 | 1 | 1 | 1 | 1 |
| 3904 | 64 | 1 | 166 | 69 | 8 | 2 | 1 | 2 | 2 | 3 | 1 | 1 | 1 | 1 | 1 | 1 | 1 | 1 |
| 3905 | 64 | 1 | 166.5 | 71 | 6 | 2 | 1 | 2 | 5 | 4 | 1 | 1 | 1 | 1 | 1 | 1 | 1 | 1 |
| 3906 | 64 | 1 | 167 | 63.5 | 8 | 2 | 1 | 1 | 2 | 2 | 1 | 1 | 1 | 1 | 2 | 1 | 3 | 1 |
| 3907 | 64 | 1 | 167 | 60 | 8 | 2 | 1 | 1 | 4 | 1 | 1 | 1 | 1 | 1 | 1 | 1 | 1 | 1 |
| 3908 | 64 | 1 | 168 | 72.5 | 8 | 2 | 1 | 1 | 4 | 1 | 1 | 1 | 1 | 2 | 2 | 1 | 2 | 2 |
| 3909 | 64 | 1 | 168 | 70 | 1 | 1 | 1 | 2 | 2 | 1 | 1 | 1 | 1 | 1 | 1 | 1 | 3 | 1 |
| 3910 | 64 | 1 | 168 | 59 | 8 | 2 | 1 | 2 | 3 | 3 | 1 | 1 | 1 | 1 | 1 | 1 | 3 | 1 |
| 3911 | 64 | 1 | 168 | 50 | 3 | 2 | 1 | 1 | 5 | 4 | 1 | 1 | 1 | 1 | 1 | 1 | 3 | 1 |
| 3912 | 64 | 1 | 170 | 83 | 8 | 2 | 1 | 2 | 5 | 1 | 1 | 1 | 1 | 1 | 1 | 1 | 3 | 1 |
| 3913 | 64 | 1 | 170 | 80 | 3 | 2 | 1 | 1 | 3 | 1 | 1 | 1 | 1 | 1 | 1 | 1 | 1 | 2 |
| 3914 | 64 | 1 | 170 | 75 | 8 | 2 | 2 | 1 | 3 | 4 | 1 | 1 | 1 | 1 | 1 | 1 | 1 | 1 |
| 3915 | 64 | 1 | 170 | 56 | 8 | 2 | 1 | 1 | 4 | 4 | 1 | 1 | 1 | 1 | 2 | 1 | 1 | 1 |
| 3916 | 64 | 1 | 171 | 78.5 | 8 | 2 | 1 | 1 | 4 | 3 | 1 | 1 | 1 | 1 | 1 | 1 | 1 | 1 |
| 3917 | 64 | 1 | 172 | 78 | 4 | 2 | 1 | 1 | 4 | 4 | 1 | 1 | 1 | 1 | 1 | 1 | 1 | 1 |
| 3918 | 64 | 1 | 173 | 73.5 | 8 | 2 | 1 | 1 | 3 | 1 | 1 | 1 | 1 | 1 | 1 | 1 | 2 | 1 |
| 3919 | 64 | 1 | 175 | 72 | 3 | 2 | 1 | 2 | 3 | 3 | 1 | 1 | 1 | 1 | 1 | 1 | 1 | 1 |
| 3920 | 64 | 1 | 175 | 65 | 3 | 2 | 1 | 1 | 5 | 1 | 1 | 1 | 2 | 1 | 1 | 1 | 3 | 1 |
| 3921 | 64 | 1 | 176 | 80 | 8 | 2 | 1 | 1 | 2 | 1 | 1 | 1 | 2 | 2 | 1 | 1 | 3 | 1 |
| 3922 | 64 | 1 | 178 | 81.8 | 8 | 2 | 1 | 1 | 2 | 1 | 1 | 1 | 1 | 1 | 1 | 1 | 1 | 1 |
| 3923 | 64 | 1 | 178.5 | 77 | 8 | 2 | 1 | 1 | 4 | 1 | 1 | 1 | 1 | 1 | 1 | 1 | 1 | 1 |
| 3924 | 64 | 1 | 180 | 82 | 5 | 2 | 1 | 2 | 3 | 1 | 1 | 1 | 1 | 1 | 1 | 1 | 2 | 1 |
| 3925 | 65 | 2 | 140 | 34.4 | 8 | 2 | 1 | 1 | 3 | 4 | 1 | 1 | 1 | 1 | 1 | 1 | 3 | 1 |
| 3926 | 65 | 2 | 141 | 57 | 8 | 2 | 1 | 1 | 2 | 4 | 1 | 1 | 1 | 1 | 1 | 1 | 1 | 1 |
| 3927 | 65 | 2 | 141 | 47.4 | 8 | 2 | 1 | 1 | 3 | 3 | 1 | 1 | 2 | 1 | 2 | 1 | 1 | 1 |
| 3928 | 65 | 2 | 142 | 39.9 | 8 | 2 | 1 | 1 | 2 | 4 | 1 | 1 | 1 | 1 | 1 | 1 | 1 | 1 |
| 3929 | 65 | 2 | 144.5 | 62.2 | 2 | 2 | 1 | 1 | 3 | 2 | 1 | 1 | 1 | 1 | 1 | 1 | 1 | 1 |
| 3930 | 65 | 2 | 146 | 43.4 | 8 | 2 | 1 | 1 | 1 | 4 | 1 | 1 | 1 | 1 | 2 | 1 | 1 | 1 |
| 3931 | 65 | 2 | 147 | 60 | 8 | 2 | 1 | 1 | 3 | 1 | 1 | 1 | 1 | 1 | 1 | 1 | 1 | 1 |
| 3932 | 65 | 2 | 147 | 51 | 8 | 2 | 1 | 1 | 3 | 3 | 1 | 1 | 1 | 1 | 1 | 1 | 1 | 1 |
| 3933 | 65 | 2 | 147 | 48 | 6 | 2 | 1 | 1 | 3 | 1 | 1 | 1 | 1 | 1 | 1 | 1 | 1 | 1 |
| 3934 | 65 | 2 | 147 | 45.4 | 4 | 2 | 1 | 1 | 6 | 3 | 1 | 1 | 1 | 1 | 1 | 1 | 1 | 1 |
| 3935 | 65 | 2 | 147 | 38.4 | 3 | 2 | 1 | 1 | 4 | 1 | 1 | 1 | 2 | 1 | 1 | 1 | 1 | 1 |
| 3936 | 65 | 2 | 147.5 | 45 | 8 | 2 | 1 | 1 | 4 | 1 | 1 | 1 | 1 | 1 | 1 | 1 | 1 | 1 |
| 3937 | 65 | 2 | 148 | 58 | 8 | 2 | 1 | 1 | 4 | 3 | 1 | 1 | 1 | 1 | 1 | 1 | 1 | 1 |
| 3938 | 65 | 2 | 148 | 46 | 8 | 2 | 1 | 1 | 3 | 1 | 1 | 1 | 1 | 1 | 2 | 1 | 1 | 1 |
| 3939 | 65 | 2 | 149 | 54 | 8 | 2 | 1 | 1 | 1 | 1 | 1 | 1 | 1 | 1 | 1 | 1 | 1 | 1 |
| 3940 | 65 | 2 | 149 | 47 | 8 | 2 | 1 | 1 | 3 | 3 | 1 | 1 | 1 | 1 | 2 | 1 | 1 | 1 |
| 3941 | 65 | 2 | 149.5 | 48 | 2 | 2 | 1 | 1 | 4 | 1 | 1 | 1 | 1 | 1 | 2 | 1 | 1 | 1 |
| 3942 | 65 | 2 | 150 | 61 | 8 | 2 | 1 | 1 | 4 | 4 | 1 | 1 | 1 | 1 | 1 | 1 | 1 | 1 |
| 3943 | 65 | 2 | 150 | 58 | 6 | 2 | 1 | 1 | 4 | 1 | 1 | 1 | 1 | 1 | 1 | 1 | 1 | 1 |
| 3944 | 65 | 2 | 150 | 54 | 6 | 2 | 1 | 1 | 3 | 1 | 1 | 1 | 1 | 1 | 1 | 1 | 1 | 1 |
| 3945 | 65 | 2 | 150 | 52.5 | 8 | 2 | 1 | 1 | 4 | 4 | 1 | 1 | 1 | 1 | 1 | 1 | 1 | 1 |
| 3946 | 65 | 2 | 150 | 52 | 8 | 2 | 1 | 1 | 3 | 1 | 1 | 1 | 1 | 1 | 2 | 1 | 1 | 1 |
| 3947 | 65 | 2 | 150.5 | 52.7 | 6 | 2 | 1 | 1 | 2 | 1 | 2 | 1 | 1 | 1 | 1 | 1 | 1 | 1 |
| 3948 | 65 | 2 | 150.5 | 47.2 | 6 | 2 | 1 | 1 | 2 | 4 | 1 | 1 | 1 | 1 | 1 | 1 | 1 | 1 |
| 3949 | 65 | 2 | 150.8 | 61 | 8 | 2 | 1 | 1 | 3 | 1 | 1 | 1 | 1 | 1 | 2 | 1 | 1 | 1 |
| 3950 | 65 | 2 | 151 | 58 | 8 | 2 | 1 | 1 | 2 | 3 | 1 | 1 | 1 | 1 | 1 | 1 | 1 | 2 |
| 3951 | 65 | 2 | 151.5 | 54.1 | 2 | 2 | 1 | 1 | 5 | 1 | 1 | 1 | 2 | 1 | 1 | 1 | 1 | 1 |
| 3952 | 65 | 2 | 151.6 | 49 | 3 | 2 | 1 | 1 | 4 | 1 | 1 | 1 | 1 | 1 | 1 | 1 | 1 | 1 |
| 3953 | 65 | 2 | 152 | 65 | 8 | 2 | 1 | 1 | 3 | 1 | 1 | 1 | 1 | 1 | 1 | 1 | 1 | 1 |
| 3954 | 65 | 2 | 152 | 55 | 6 | 2 | 1 | 1 | 2 | 3 | 1 | 1 | 1 | 1 | 1 | 1 | 1 | 1 |
| 3955 | 65 | 2 | 152 | 53.5 | 2 | 2 | 1 | 1 | 3 | 2 | 1 | 1 | 2 | 1 | 2 | 1 | 1 | 1 |
| 3956 | 65 | 2 | 152 | 48 | 8 | 2 | 1 | 1 | 2 | 3 | 1 | 1 | 1 | 1 | 1 | 1 | 1 | 1 |
| 3957 | 65 | 2 | 153 | 71.5 | 4 | 2 | 1 | 1 | 3 | 4 | 1 | 1 | 1 | 1 | 2 | 1 | 1 | 1 |
| 3958 | 65 | 2 | 153 | 53.5 | 8 | 2 | 1 | 1 | 3 | 1 | 1 | 1 | 1 | 2 | 2 | 1 | 1 | 2 |
| 3959 | 65 | 2 | 153 | 52 | 8 | 2 | 1 | 1 | 4 | 1 | 1 | 1 | 1 | 1 | 1 | 1 | 1 | 1 |
| 3960 | 65 | 2 | 153 | 52 | 4 | 2 | 1 | 1 | 3 | 4 | 1 | 1 | 1 | 1 | 1 | 1 | 1 | 1 |
| 3961 | 65 | 2 | 153 | 48 | 8 | 2 | 1 | 1 | 2 | 3 | 1 | 1 | 1 | 1 | 2 | 1 | 1 | 1 |
| 3962 | 65 | 2 | 153 | 47 | 2 | 2 | 1 | 1 | 4 | 2 | 1 | 1 | 1 | 1 | 1 | 1 | 1 | 1 |
| 3963 | 65 | 2 | 154 | 71 | 8 | 2 | 2 | 1 | 1 | 3 | 1 | 1 | 1 | 1 | 1 | 1 | 1 | 1 |
| 3964 | 65 | 2 | 154 | 58 | 3 | 2 | 1 | 1 | 4 | 1 | 1 | 1 | 1 | 1 | 1 | 1 | 1 | 1 |
| 3965 | 65 | 2 | 154 | 44 | 8 | 2 | 1 | 1 | 2 | 3 | 1 | 1 | 1 | 1 | 1 | 1 | 1 | 1 |
| 3966 | 65 | 2 | 155 | 71 | 8 | 2 | 1 | 1 | 1 | 3 | 1 | 1 | 1 | 1 | 2 | 1 | 1 | 1 |
| 3967 | 65 | 2 | 155 | 59 | 8 | 2 | 1 | 1 | 4 | 1 | 1 | 1 | 1 | 1 | 2 | 1 | 1 | 1 |
| 3968 | 65 | 2 | 155 | 56 | 2 | 1 | 1 | 1 | 3 | 2 | 1 | 1 | 1 | 1 | 1 | 1 | 1 | 1 |
| 3969 | 65 | 2 | 155 | 53 | 8 | 2 | 1 | 1 | 2 | 2 | 1 | 1 | 1 | 1 | 1 | 1 | 1 | 1 |
| 3970 | 65 | 2 | 155 | 50 | 8 | 2 | 1 | 1 | 3 | 2 | 1 | 1 | 1 | 1 | 1 | 1 | 1 | 1 |
| 3971 | 65 | 2 | 155 | 49 | 8 | 2 | 1 | 1 | 2 | 3 | 1 | 1 | 1 | 1 | 1 | 1 | 1 | 1 |
| 3972 | 65 | 2 | 155 | 47 | 8 | 2 | 1 | 1 | 2 | 2 | 1 | 1 | 1 | 1 | 1 | 1 | 1 | 1 |
| 3973 | 65 | 2 | 156 | 66 | 8 | 2 | 1 | 1 | 3 | 1 | 1 | 1 | 1 | 1 | 1 | 1 | 1 | 1 |
| 3974 | 65 | 2 | 156 | 65 | 8 | 2 | 1 | 1 | 3 | 4 | 3 | 1 | 1 | 1 | 2 | 1 | 1 | 1 |
| 3975 | 65 | 2 | 156 | 60 | 2 | 2 | 1 | 1 | 6 | 1 | 1 | 1 | 1 | 1 | 2 | 2 | 1 | 1 |
| 3976 | 65 | 2 | 156 | 56 | 2 | 3 | 1 | 1 | 5 | 1 | 1 | 1 | 1 | 1 | 1 | 1 | 1 | 1 |
| 3977 | 65 | 2 | 156 | 52.5 | 8 | 2 | 1 | 1 | 3 | 4 | 1 | 1 | 1 | 1 | 1 | 1 | 1 | 1 |
| 3978 | 65 | 2 | 156 | 45 | 8 | 2 | 1 | 1 | 3 | 4 | 1 | 1 | 1 | 1 | 1 | 1 | 1 | 1 |
| 3979 | 65 | 2 | 156.5 | 67 | 8 | 2 | 1 | 1 | 5 | 4 | 1 | 1 | 1 | 1 | 1 | 1 | 1 | 1 |
| 3980 | 65 | 2 | 156.5 | 63.1 | 8 | 2 | 1 | 1 | 3 | 3 | 1 | 1 | 1 | 1 | 1 | 1 | 1 | 1 |
| 3981 | 65 | 2 | 157 | 67.9 | 8 | 2 | 1 | 1 | 2 | 1 | 1 | 1 | 1 | 1 | 1 | 1 | 1 | 1 |
| 3982 | 65 | 2 | 157 | 65.5 | 8 | 2 | 1 | 1 | 5 | 2 | 1 | 1 | 1 | 1 | 1 | 1 | 1 | 1 |
| 3983 | 65 | 2 | 157 | 60 | 8 | 2 | 1 | 1 | 2 | 1 | 1 | 1 | 1 | 1 | 1 | 1 | 1 | 1 |
| 3984 | 65 | 2 | 157 | 57.2 | 8 | 2 | 1 | 1 | 3 | 1 | 2 | 1 | 1 | 2 | 2 | 1 | 1 | 2 |
| 3985 | 65 | 2 | 157 | 53.5 | 8 | 2 | 2 | 1 | 3 | 2 | 1 | 1 | 2 | 1 | 1 | 1 | 1 | 1 |
| 3986 | 65 | 2 | 158 | 65 | 8 | 2 | 1 | 1 | 3 | 1 | 1 | 1 | 1 | 1 | 1 | 1 | 1 | 1 |
| 3987 | 65 | 2 | 158 | 63 | 8 | 2 | 1 | 1 | 5 | 3 | 1 | 1 | 1 | 1 | 1 | 1 | 1 | 2 |
| 3988 | 65 | 2 | 158 | 62 | 8 | 2 | 1 | 1 | 3 | 1 | 1 | 1 | 1 | 1 | 1 | 1 | 1 | 2 |
| 3989 | 65 | 2 | 158 | 60 | 8 | 2 | 1 | 1 | 4 | 2 | 1 | 1 | 2 | 1 | 1 | 1 | 1 | 1 |
| 3990 | 65 | 2 | 158 | 58 | 2 | 2 | 1 | 1 | 4 | 3 | 1 | 1 | 1 | 1 | 1 | 1 | 1 | 1 |
| 3991 | 65 | 2 | 158 | 55.5 | 8 | 2 | 1 | 1 | 3 | 2 | 1 | 1 | 1 | 1 | 1 | 1 | 1 | 1 |
| 3992 | 65 | 2 | 158 | 55 | 8 | 2 | 1 | 1 | 3 | 1 | 1 | 1 | 1 | 1 | 1 | 1 | 1 | 1 |
| 3993 | 65 | 2 | 160 | 74 | 8 | 2 | 2 | 1 | 4 | 4 | 1 | 1 | 1 | 1 | 2 | 1 | 1 | 1 |
| 3994 | 65 | 2 | 160 | 60.2 | 6 | 2 | 1 | 1 | 4 | 1 | 1 | 1 | 1 | 1 | 1 | 1 | 1 | 1 |
| 3995 | 65 | 2 | 160 | 53 | 8 | 2 | 1 | 1 | 3 | 4 | 1 | 1 | 1 | 1 | 1 | 1 | 1 | 1 |
| 3996 | 65 | 2 | 161 | 60 | 8 | 2 | 1 | 1 | 5 | 1 | 2 | 1 | 1 | 1 | 1 | 1 | 1 | 1 |
| 3997 | 65 | 2 | 161 | 60 | 4 | 2 | 1 | 1 | 5 | 4 | 1 | 1 | 1 | 1 | 1 | 1 | 1 | 1 |
| 3998 | 65 | 2 | 161.5 | 63 | 6 | 2 | 1 | 1 | 4 | 1 | 1 | 1 | 1 | 1 | 1 | 1 | 1 | 1 |
| 3999 | 65 | 2 | 162 | 74 | 2 | 2 | 1 | 1 | 4 | 1 | 1 | 1 | 2 | 1 | 1 | 1 | 1 | 1 |
| 4000 | 65 | 2 | 162 | 63 | 2 | 2 | 1 | 1 | 3 | 2 | 1 | 1 | 1 | 1 | 2 | 2 | 1 | 1 |
| 4001 | 65 | 2 | 162 | 62 | 8 | 2 | 1 | 1 | 3 | 1 | 1 | 1 | 1 | 1 | 1 | 1 | 1 | 1 |
| 4002 | 65 | 2 | 164 | 80.5 | 4 | 2 | 1 | 1 | 4 | 1 | 1 | 1 | 1 | 1 | 1 | 1 | 1 | 1 |
| 4003 | 65 | 2 | 165 | 87.5 | 8 | 2 | 1 | 1 | 6 | 1 | 1 | 1 | 1 | 1 | 1 | 1 | 1 | 1 |
| 4004 | 65 | 2 | 165 | 71.5 | 8 | 2 | 1 | 1 | 4 | 1 | 1 | 1 | 2 | 1 | 1 | 1 | 1 | 1 |
| 4005 | 65 | 2 | 165 | 69 | 8 | 2 | 2 | 1 | 4 | 4 | 1 | 1 | 2 | 1 | 1 | 1 | 1 | 1 |
| 4006 | 65 | 2 | 166 | 80 | 8 | 2 | 1 | 1 | 2 | 1 | 1 | 1 | 1 | 1 | 1 | 1 | 1 | 1 |
| 4007 | 65 | 2 | 180 | 85 | 8 | 2 | 1 | 1 | 2 | 4 | 2 | 1 | 1 | 2 | 1 | 1 | 1 | 2 |
| 4008 | 65 | 1 | 151 | 52 | 8 | 2 | 1 | 1 | 1 | 4 | 1 | 1 | 1 | 1 | 1 | 1 | 1 | 2 |
| 4009 | 65 | 1 | 152 | 58.4 | 5 | 2 | 1 | 2 | 2 | 1 | 1 | 1 | 1 | 1 | 1 | 1 | 3 | 1 |
| 4010 | 65 | 1 | 155 | 52 | 8 | 2 | 1 | 1 | 2 | 3 | 1 | 1 | 1 | 1 | 1 | 1 | 1 | 1 |
| 4011 | 65 | 1 | 156 | 60 | 8 | 2 | 1 | 1 | 3 | 3 | 1 | 1 | 1 | 1 | 1 | 1 | 1 | 1 |
| 4012 | 65 | 1 | 156.7 | 48.6 | 8 | 2 | 1 | 1 | 3 | 1 | 1 | 1 | 2 | 1 | 1 | 1 | 1 | 1 |
| 4013 | 65 | 1 | 157 | 60 | 8 | 2 | 1 | 1 | 3 | 1 | 1 | 1 | 1 | 1 | 1 | 1 | 1 | 1 |
| 4014 | 65 | 1 | 158 | 70 | 8 | 2 | 1 | 1 | 2 | 2 | 1 | 1 | 1 | 1 | 2 | 1 | 3 | 1 |
| 4015 | 65 | 1 | 158 | 63.2 | 8 | 2 | 2 | 3 | 3 | 3 | 1 | 1 | 1 | 1 | 1 | 1 | 3 | 1 |
| 4016 | 65 | 1 | 158 | 60.1 | 8 | 2 | 1 | 2 | 3 | 2 | 1 | 1 | 1 | 1 | 1 | 1 | 3 | 1 |
| 4017 | 65 | 1 | 158 | 60 | 8 | 2 | 1 | 1 | 4 | 3 | 1 | 1 | 1 | 2 | 1 | 1 | 1 | 2 |
| 4018 | 65 | 1 | 159 | 79 | 8 | 2 | 1 | 1 | 2 | 3 | 1 | 1 | 1 | 1 | 2 | 1 | 1 | 1 |
| 4019 | 65 | 1 | 160 | 62 | 8 | 2 | 1 | 1 | 4 | 1 | 1 | 1 | 1 | 2 | 1 | 1 | 1 | 1 |
| 4020 | 65 | 1 | 160 | 55.2 | 8 | 2 | 1 | 1 | 4 | 1 | 1 | 1 | 1 | 1 | 1 | 1 | 3 | 1 |
| 4021 | 65 | 1 | 160 | 52 | 3 | 2 | 1 | 4 | 4 | 1 | 1 | 1 | 1 | 1 | 2 | 1 | 3 | 1 |
| 4022 | 65 | 1 | 161 | 67.3 | 2 | 2 | 1 | 1 | 4 | 1 | 1 | 1 | 1 | 1 | 1 | 1 | 1 | 1 |
| 4023 | 65 | 1 | 161 | 51.8 | 2 | 2 | 1 | 2 | 4 | 2 | 1 | 1 | 1 | 1 | 1 | 1 | 3 | 1 |
| 4024 | 65 | 1 | 162 | 62 | 8 | 2 | 1 | 1 | 3 | 3 | 1 | 1 | 1 | 1 | 1 | 1 | 1 | 1 |
| 4025 | 65 | 1 | 163 | 78.3 | 8 | 2 | 1 | 1 | 2 | 2 | 1 | 1 | 1 | 1 | 1 | 1 | 1 | 1 |
| 4026 | 65 | 1 | 163 | 72 | 3 | 4 | 1 | 1 | 4 | 4 | 1 | 1 | 1 | 1 | 1 | 1 | 1 | 1 |
| 4027 | 65 | 1 | 163 | 67.5 | 8 | 2 | 1 | 2 | 3 | 2 | 1 | 1 | 1 | 1 | 1 | 1 | 3 | 1 |
| 4028 | 65 | 1 | 163 | 56.5 | 8 | 2 | 1 | 1 | 2 | 4 | 1 | 1 | 1 | 1 | 2 | 1 | 1 | 1 |
| 4029 | 65 | 1 | 163 | 54.5 | 2 | 2 | 1 | 1 | 2 | 1 | 1 | 1 | 1 | 2 | 2 | 1 | 1 | 1 |
| 4030 | 65 | 1 | 163.5 | 64.2 | 8 | 2 | 1 | 1 | 3 | 3 | 2 | 1 | 2 | 1 | 1 | 1 | 2 | 1 |
| 4031 | 65 | 1 | 164 | 75 | 2 | 2 | 1 | 1 | 4 | 1 | 1 | 1 | 1 | 1 | 1 | 1 | 1 | 1 |
| 4032 | 65 | 1 | 164 | 68 | 4 | 2 | 1 | 1 | 2 | 2 | 1 | 1 | 1 | 1 | 1 | 1 | 3 | 1 |
| 4033 | 65 | 1 | 164 | 67.2 | 8 | 2 | 1 | 1 | 2 | 4 | 1 | 1 | 1 | 1 | 2 | 2 | 2 | 1 |
| 4034 | 65 | 1 | 164 | 59.4 | 2 | 2 | 1 | 1 | 2 | 2 | 1 | 1 | 1 | 1 | 1 | 1 | 1 | 1 |
| 4035 | 65 | 1 | 164 | 58.4 | 6 | 2 | 1 | 1 | 3 | 1 | 1 | 1 | 1 | 1 | 2 | 2 | 1 | 1 |
| 4036 | 65 | 1 | 165 | 83.5 | 8 | 2 | 1 | 1 | 5 | 2 | 1 | 1 | 1 | 1 | 1 | 1 | 1 | 1 |
| 4037 | 65 | 1 | 165 | 75 | 8 | 2 | 1 | 1 | 3 | 4 | 1 | 1 | 2 | 1 | 2 | 1 | 2 | 1 |
| 4038 | 65 | 1 | 165 | 73.5 | 8 | 2 | 1 | 2 | 3 | 1 | 1 | 1 | 1 | 1 | 1 | 1 | 2 | 1 |
| 4039 | 65 | 1 | 165 | 67 | 8 | 2 | 1 | 1 | 2 | 1 | 1 | 1 | 1 | 1 | 1 | 1 | 1 | 1 |
| 4040 | 65 | 1 | 165 | 64 | 8 | 2 | 1 | 1 | 2 | 2 | 1 | 1 | 1 | 1 | 1 | 1 | 1 | 1 |
| 4041 | 65 | 1 | 165 | 55 | 8 | 2 | 1 | 2 | 3 | 2 | 1 | 1 | 1 | 1 | 2 | 1 | 2 | 1 |
| 4042 | 65 | 1 | 165 | 53.8 | 8 | 2 | 1 | 1 | 3 | 3 | 1 | 1 | 1 | 1 | 1 | 1 | 3 | 1 |
| 4043 | 65 | 1 | 165 | 50 | 8 | 2 | 1 | 1 | 4 | 2 | 1 | 1 | 1 | 1 | 1 | 1 | 3 | 1 |
| 4044 | 65 | 1 | 166 | 66 | 8 | 2 | 1 | 1 | 2 | 3 | 1 | 1 | 1 | 1 | 1 | 1 | 1 | 1 |
| 4045 | 65 | 1 | 166.5 | 73 | 8 | 2 | 1 | 3 | 3 | 2 | 1 | 1 | 1 | 1 | 1 | 1 | 3 | 1 |
| 4046 | 65 | 1 | 167 | 71 | 8 | 2 | 1 | 1 | 3 | 1 | 1 | 2 | 1 | 1 | 2 | 1 | 1 | 1 |
| 4047 | 65 | 1 | 167 | 71 | 8 | 2 | 1 | 1 | 3 | 1 | 1 | 1 | 1 | 1 | 1 | 1 | 3 | 1 |
| 4048 | 65 | 1 | 167 | 54 | 8 | 2 | 1 | 2 | 4 | 3 | 1 | 1 | 1 | 1 | 1 | 1 | 3 | 1 |
| 4049 | 65 | 1 | 167.5 | 57.8 | 6 | 2 | 1 | 1 | 4 | 1 | 1 | 1 | 1 | 1 | 1 | 1 | 1 | 1 |
| 4050 | 65 | 1 | 167.5 | 57 | 8 | 1 | 1 | 4 | 3 | 1 | 1 | 1 | 1 | 1 | 2 | 1 | 3 | 1 |
| 4051 | 65 | 1 | 168 | 72 | 8 | 2 | 1 | 1 | 4 | 4 | 1 | 1 | 1 | 1 | 1 | 1 | 3 | 1 |
| 4052 | 65 | 1 | 168 | 70 | 3 | 2 | 1 | 1 | 3 | 1 | 1 | 1 | 2 | 1 | 1 | 1 | 1 | 1 |
| 4053 | 65 | 1 | 168 | 69 | 8 | 2 | 1 | 2 | 3 | 3 | 1 | 1 | 1 | 1 | 1 | 1 | 3 | 1 |
| 4054 | 65 | 1 | 168 | 66 | 2 | 2 | 1 | 1 | 3 | 1 | 1 | 1 | 1 | 1 | 1 | 1 | 1 | 1 |
| 4055 | 65 | 1 | 168 | 65 | 4 | 2 | 1 | 2 | 3 | 1 | 1 | 1 | 1 | 1 | 1 | 1 | 3 | 1 |
| 4056 | 65 | 1 | 168 | 61.8 | 3 | 2 | 1 | 2 | 3 | 1 | 1 | 1 | 1 | 1 | 1 | 1 | 3 | 1 |
| 4057 | 65 | 1 | 168 | 60 | 3 | 2 | 1 | 4 | 5 | 4 | 1 | 1 | 1 | 1 | 1 | 1 | 3 | 1 |
| 4058 | 65 | 1 | 168 | 55.8 | 8 | 2 | 1 | 1 | 3 | 1 | 1 | 1 | 1 | 1 | 1 | 1 | 3 | 1 |
| 4059 | 65 | 1 | 168 | 53 | 3 | 2 | 1 | 1 | 3 | 2 | 1 | 1 | 1 | 1 | 1 | 1 | 1 | 1 |
| 4060 | 65 | 1 | 169 | 70 | 6 | 2 | 1 | 1 | 4 | 1 | 1 | 1 | 1 | 1 | 1 | 1 | 1 | 1 |
| 4061 | 65 | 1 | 169 | 67.4 | 6 | 2 | 1 | 1 | 3 | 1 | 1 | 1 | 2 | 1 | 1 | 1 | 1 | 2 |
| 4062 | 65 | 1 | 169 | 51 | 8 | 2 | 1 | 1 | 2 | 3 | 1 | 1 | 1 | 1 | 2 | 1 | 1 | 1 |
| 4063 | 65 | 1 | 170 | 73 | 2 | 2 | 1 | 1 | 2 | 2 | 1 | 1 | 1 | 1 | 1 | 1 | 1 | 1 |
| 4064 | 65 | 1 | 170 | 69 | 8 | 2 | 1 | 2 | 4 | 1 | 1 | 1 | 1 | 1 | 1 | 1 | 1 | 1 |
| 4065 | 65 | 1 | 170 | 59 | 8 | 2 | 1 | 1 | 4 | 1 | 1 | 1 | 1 | 1 | 2 | 1 | 1 | 1 |
| 4066 | 65 | 1 | 170 | 58 | 8 | 2 | 1 | 1 | 4 | 1 | 1 | 1 | 1 | 1 | 1 | 1 | 1 | 1 |
| 4067 | 65 | 1 | 171 | 59.6 | 6 | 2 | 1 | 1 | 3 | 2 | 1 | 1 | 1 | 1 | 1 | 1 | 3 | 1 |
| 4068 | 65 | 1 | 173 | 72 | 8 | 2 | 1 | 1 | 2 | 2 | 1 | 1 | 1 | 1 | 1 | 1 | 1 | 1 |
| 4069 | 65 | 1 | 173 | 66 | 8 | 2 | 1 | 1 | 5 | 4 | 1 | 1 | 1 | 1 | 1 | 1 | 1 | 1 |
| 4070 | 65 | 1 | 174 | 60 | 2 | 4 | 1 | 4 | 4 | 1 | 1 | 1 | 1 | 1 | 1 | 1 | 3 | 1 |
| 4071 | 65 | 1 | 175 | 92.5 | 8 | 2 | 1 | 1 | 4 | 2 | 1 | 1 | 1 | 1 | 1 | 1 | 1 | 1 |
| 4072 | 65 | 1 | 175 | 73.5 | 8 | 2 | 1 | 1 | 3 | 2 | 1 | 1 | 1 | 1 | 2 | 1 | 3 | 1 |
| 4073 | 65 | 1 | 177 | 79.5 | 8 | 2 | 1 | 2 | 4 | 4 | 1 | 1 | 1 | 1 | 1 | 1 | 3 | 1 |
| 4074 | 66 | 2 | 140 | 47 | 2 | 2 | 1 | 1 | 2 | 2 | 1 | 1 | 2 | 1 | 2 | 1 | 1 | 1 |
| 4075 | 66 | 2 | 141 | 61.7 | 6 | 2 | 1 | 1 | 4 | 1 | 1 | 1 | 1 | 1 | 1 | 1 | 1 | 1 |
| 4076 | 66 | 2 | 145 | 40 | 8 | 2 | 1 | 1 | 2 | 3 | 1 | 1 | 1 | 1 | 1 | 1 | 1 | 1 |
| 4077 | 66 | 2 | 146 | 52 | 8 | 2 | 1 | 1 | 3 | 1 | 1 | 1 | 1 | 1 | 2 | 1 | 1 | 1 |
| 4078 | 66 | 2 | 147 | 69 | 8 | 2 | 1 | 1 | 2 | 1 | 1 | 1 | 1 | 1 | 1 | 1 | 1 | 1 |
| 4079 | 66 | 2 | 148 | 76 | 8 | 2 | 1 | 1 | 6 | 3 | 1 | 1 | 1 | 1 | 1 | 1 | 1 | 1 |
| 4080 | 66 | 2 | 148 | 53 | 6 | 2 | 1 | 1 | 4 | 1 | 1 | 1 | 1 | 1 | 1 | 1 | 1 | 1 |
| 4081 | 66 | 2 | 148 | 53 | 3 | 2 | 1 | 1 | 3 | 1 | 1 | 1 | 1 | 1 | 1 | 1 | 1 | 1 |
| 4082 | 66 | 2 | 148 | 48 | 8 | 2 | 1 | 1 | 3 | 3 | 1 | 1 | 1 | 1 | 2 | 2 | 1 | 1 |
| 4083 | 66 | 2 | 148 | 45 | 8 | 3 | 2 | 1 | 4 | 2 | 1 | 1 | 1 | 1 | 1 | 1 | 1 | 1 |
| 4084 | 66 | 2 | 148 | 43 | 3 | 2 | 1 | 1 | 5 | 3 | 1 | 1 | 1 | 1 | 1 | 1 | 1 | 1 |
| 4085 | 66 | 2 | 150 | 63 | 8 | 2 | 1 | 1 | 2 | 1 | 1 | 1 | 2 | 1 | 1 | 1 | 1 | 1 |
| 4086 | 66 | 2 | 150 | 53 | 8 | 2 | 1 | 1 | 2 | 1 | 1 | 1 | 1 | 1 | 1 | 1 | 1 | 1 |
| 4087 | 66 | 2 | 150 | 50 | 6 | 2 | 1 | 1 | 3 | 2 | 1 | 1 | 1 | 1 | 1 | 1 | 1 | 1 |
| 4088 | 66 | 2 | 151 | 60 | 8 | 2 | 1 | 1 | 4 | 1 | 1 | 1 | 1 | 1 | 1 | 1 | 1 | 1 |
| 4089 | 66 | 2 | 151 | 53.6 | 3 | 2 | 1 | 1 | 4 | 1 | 1 | 1 | 1 | 1 | 1 | 1 | 1 | 1 |
| 4090 | 66 | 2 | 151 | 52 | 4 | 2 | 1 | 1 | 3 | 4 | 1 | 1 | 1 | 1 | 1 | 1 | 1 | 1 |
| 4091 | 66 | 2 | 151 | 46 | 3 | 2 | 1 | 1 | 4 | 1 | 1 | 2 | 2 | 1 | 1 | 1 | 1 | 1 |
| 4092 | 66 | 2 | 151.5 | 63 | 4 | 2 | 1 | 1 | 2 | 1 | 1 | 1 | 1 | 1 | 2 | 1 | 1 | 1 |
| 4093 | 66 | 2 | 151.8 | 48.6 | 8 | 2 | 1 | 1 | 3 | 2 | 1 | 1 | 1 | 1 | 1 | 1 | 1 | 1 |
| 4094 | 66 | 2 | 152 | 66 | 8 | 2 | 1 | 1 | 2 | 3 | 1 | 1 | 1 | 1 | 1 | 1 | 1 | 1 |
| 4095 | 66 | 2 | 153 | 75.5 | 8 | 2 | 1 | 1 | 3 | 1 | 1 | 1 | 1 | 1 | 2 | 1 | 1 | 1 |
| 4096 | 66 | 2 | 153 | 65 | 8 | 3 | 2 | 1 | 5 | 3 | 1 | 1 | 1 | 1 | 2 | 1 | 1 | 1 |
| 4097 | 66 | 2 | 153 | 60 | 8 | 2 | 1 | 1 | 2 | 3 | 1 | 1 | 1 | 1 | 1 | 1 | 1 | 1 |
| 4098 | 66 | 2 | 154 | 61 | 3 | 2 | 1 | 1 | 3 | 1 | 1 | 1 | 2 | 1 | 2 | 1 | 1 | 1 |
| 4099 | 66 | 2 | 154 | 56.4 | 2 | 2 | 1 | 1 | 4 | 1 | 1 | 1 | 2 | 1 | 1 | 1 | 1 | 1 |
| 4100 | 66 | 2 | 154 | 54 | 8 | 1 | 1 | 1 | 5 | 1 | 1 | 1 | 1 | 1 | 1 | 1 | 1 | 1 |
| 4101 | 66 | 2 | 154 | 49 | 8 | 2 | 1 | 1 | 3 | 1 | 1 | 1 | 1 | 1 | 1 | 1 | 1 | 1 |
| 4102 | 66 | 2 | 155 | 66 | 8 | 2 | 1 | 1 | 2 | 3 | 1 | 1 | 1 | 1 | 2 | 1 | 1 | 1 |
| 4103 | 66 | 2 | 155 | 61 | 8 | 2 | 1 | 1 | 2 | 3 | 1 | 1 | 1 | 1 | 1 | 1 | 2 | 1 |
| 4104 | 66 | 2 | 155 | 60 | 1 | 2 | 1 | 1 | 2 | 4 | 1 | 1 | 1 | 1 | 1 | 1 | 1 | 1 |
| 4105 | 66 | 2 | 155 | 59 | 8 | 2 | 2 | 1 | 3 | 3 | 1 | 1 | 1 | 1 | 1 | 1 | 1 | 1 |
| 4106 | 66 | 2 | 155 | 53 | 8 | 2 | 1 | 1 | 2 | 1 | 1 | 1 | 1 | 1 | 1 | 1 | 1 | 2 |
| 4107 | 66 | 2 | 155 | 44 | 8 | 2 | 1 | 1 | 2 | 3 | 1 | 1 | 1 | 1 | 2 | 2 | 1 | 1 |
| 4108 | 66 | 2 | 156 | 56 | 6 | 1 | 1 | 1 | 2 | 1 | 1 | 1 | 1 | 1 | 1 | 1 | 1 | 1 |
| 4109 | 66 | 2 | 156 | 55 | 8 | 2 | 1 | 1 | 3 | 2 | 1 | 1 | 1 | 1 | 2 | 1 | 1 | 1 |
| 4110 | 66 | 2 | 156.1 | 63 | 8 | 2 | 2 | 1 | 2 | 1 | 1 | 1 | 1 | 1 | 1 | 1 | 1 | 1 |
| 4111 | 66 | 2 | 156.5 | 49.7 | 4 | 3 | 1 | 1 | 1 | 1 | 1 | 1 | 1 | 1 | 1 | 1 | 1 | 1 |
| 4112 | 66 | 2 | 157 | 59 | 8 | 2 | 1 | 1 | 2 | 1 | 1 | 1 | 1 | 1 | 1 | 1 | 1 | 1 |
| 4113 | 66 | 2 | 157 | 56 | 8 | 2 | 1 | 1 | 5 | 2 | 1 | 1 | 2 | 1 | 1 | 1 | 1 | 1 |
| 4114 | 66 | 2 | 157 | 52.5 | 8 | 2 | 1 | 1 | 3 | 4 | 1 | 1 | 1 | 1 | 1 | 1 | 1 | 1 |
| 4115 | 66 | 2 | 157 | 47 | 8 | 2 | 1 | 1 | 5 | 1 | 1 | 1 | 1 | 1 | 1 | 1 | 1 | 1 |
| 4116 | 66 | 2 | 158 | 66.5 | 8 | 2 | 1 | 1 | 5 | 1 | 1 | 1 | 1 | 1 | 1 | 1 | 2 | 1 |
| 4117 | 66 | 2 | 158 | 60 | 4 | 2 | 1 | 1 | 3 | 3 | 1 | 1 | 1 | 1 | 1 | 1 | 1 | 1 |
| 4118 | 66 | 2 | 158 | 58.5 | 8 | 2 | 1 | 1 | 3 | 4 | 1 | 1 | 1 | 1 | 2 | 1 | 1 | 1 |
| 4119 | 66 | 2 | 159 | 68 | 8 | 4 | 1 | 1 | 4 | 1 | 1 | 1 | 1 | 1 | 2 | 2 | 1 | 1 |
| 4120 | 66 | 2 | 159 | 60 | 8 | 2 | 1 | 1 | 2 | 2 | 1 | 1 | 1 | 1 | 1 | 1 | 1 | 1 |
| 4121 | 66 | 2 | 159 | 52 | 8 | 2 | 1 | 1 | 4 | 1 | 1 | 1 | 1 | 1 | 2 | 1 | 1 | 1 |
| 4122 | 66 | 2 | 159 | 52 | 8 | 2 | 1 | 1 | 3 | 1 | 1 | 1 | 1 | 1 | 1 | 1 | 1 | 1 |
| 4123 | 66 | 2 | 160 | 70 | 6 | 2 | 1 | 1 | 3 | 1 | 1 | 1 | 1 | 1 | 2 | 1 | 1 | 1 |
| 4124 | 66 | 2 | 160 | 68 | 8 | 3 | 1 | 1 | 1 | 1 | 1 | 1 | 1 | 1 | 1 | 1 | 1 | 1 |
| 4125 | 66 | 2 | 160 | 64 | 8 | 2 | 1 | 1 | 4 | 1 | 1 | 1 | 1 | 1 | 1 | 1 | 1 | 1 |
| 4126 | 66 | 2 | 161 | 51.5 | 8 | 2 | 1 | 1 | 4 | 4 | 1 | 1 | 1 | 1 | 1 | 1 | 1 | 1 |
| 4127 | 66 | 2 | 161.2 | 60.2 | 3 | 2 | 1 | 1 | 5 | 3 | 1 | 1 | 1 | 1 | 1 | 1 | 1 | 1 |
| 4128 | 66 | 2 | 162 | 65 | 8 | 2 | 1 | 1 | 3 | 1 | 1 | 1 | 1 | 1 | 1 | 1 | 1 | 1 |
| 4129 | 66 | 2 | 162 | 61 | 8 | 2 | 1 | 1 | 3 | 4 | 1 | 1 | 1 | 1 | 1 | 1 | 1 | 1 |
| 4130 | 66 | 2 | 162 | 59 | 8 | 3 | 1 | 1 | 4 | 1 | 1 | 1 | 1 | 1 | 1 | 1 | 1 | 1 |
| 4131 | 66 | 2 | 162.5 | 50 | 2 | 3 | 1 | 1 | 4 | 1 | 1 | 1 | 1 | 1 | 1 | 1 | 1 | 1 |
| 4132 | 66 | 2 | 163 | 64 | 2 | 2 | 1 | 1 | 5 | 3 | 1 | 1 | 1 | 1 | 1 | 1 | 1 | 1 |
| 4133 | 66 | 2 | 165 | 62.5 | 8 | 2 | 1 | 1 | 4 | 3 | 1 | 1 | 1 | 1 | 1 | 1 | 1 | 1 |
| 4134 | 66 | 2 | 165 | 55 | 1 | 2 | 1 | 1 | 5 | 2 | 1 | 1 | 1 | 1 | 1 | 1 | 1 | 1 |
| 4135 | 66 | 2 | 167 | 65 | 8 | 3 | 1 | 1 | 2 | 2 | 1 | 1 | 1 | 1 | 1 | 1 | 1 | 1 |
| 4136 | 66 | 1 | 150 | 49 | 1 | 2 | 1 | 2 | 3 | 1 | 1 | 1 | 1 | 1 | 1 | 1 | 1 | 1 |
| 4137 | 66 | 1 | 155 | 70 | 8 | 2 | 1 | 1 | 2 | 3 | 1 | 1 | 1 | 1 | 2 | 1 | 1 | 1 |
| 4138 | 66 | 1 | 155 | 44 | 8 | 2 | 2 | 1 | 4 | 1 | 1 | 1 | 1 | 1 | 2 | 1 | 2 | 1 |
| 4139 | 66 | 1 | 160 | 72 | 6 | 1 | 1 | 1 | 3 | 1 | 1 | 1 | 2 | 1 | 2 | 1 | 1 | 1 |
| 4140 | 66 | 1 | 160 | 63.1 | 2 | 2 | 1 | 4 | 3 | 1 | 1 | 1 | 1 | 1 | 1 | 1 | 3 | 1 |
| 4141 | 66 | 1 | 160 | 62.5 | 8 | 2 | 2 | 1 | 2 | 2 | 1 | 1 | 1 | 1 | 1 | 1 | 1 | 1 |
| 4142 | 66 | 1 | 160 | 60 | 6 | 2 | 1 | 2 | 4 | 1 | 1 | 1 | 1 | 1 | 1 | 1 | 1 | 1 |
| 4143 | 66 | 1 | 162 | 65 | 4 | 2 | 1 | 2 | 3 | 1 | 1 | 1 | 1 | 2 | 2 | 1 | 2 | 2 |
| 4144 | 66 | 1 | 162 | 63.2 | 8 | 2 | 1 | 2 | 4 | 2 | 1 | 1 | 1 | 1 | 1 | 1 | 3 | 1 |
| 4145 | 66 | 1 | 162 | 50 | 2 | 2 | 1 | 1 | 5 | 1 | 1 | 1 | 2 | 1 | 1 | 2 | 1 | 1 |
| 4146 | 66 | 1 | 163 | 75 | 2 | 2 | 1 | 1 | 4 | 3 | 1 | 1 | 1 | 1 | 1 | 1 | 1 | 1 |
| 4147 | 66 | 1 | 163 | 58 | 8 | 2 | 1 | 4 | 4 | 1 | 1 | 1 | 1 | 1 | 1 | 1 | 1 | 1 |
| 4148 | 66 | 1 | 164 | 75.3 | 2 | 2 | 1 | 2 | 2 | 2 | 1 | 1 | 1 | 1 | 2 | 1 | 1 | 1 |
| 4149 | 66 | 1 | 165 | 67 | 8 | 2 | 1 | 1 | 5 | 2 | 1 | 1 | 1 | 1 | 1 | 1 | 1 | 1 |
| 4150 | 66 | 1 | 165.2 | 52 | 8 | 2 | 1 | 1 | 4 | 1 | 1 | 1 | 2 | 1 | 1 | 1 | 1 | 1 |
| 4151 | 66 | 1 | 165.5 | 63.5 | 5 | 2 | 1 | 1 | 5 | 2 | 1 | 1 | 2 | 1 | 1 | 1 | 1 | 1 |
| 4152 | 66 | 1 | 167 | 76 | 2 | 2 | 1 | 1 | 4 | 2 | 1 | 1 | 1 | 1 | 1 | 1 | 1 | 1 |
| 4153 | 66 | 1 | 167 | 71 | 8 | 2 | 1 | 1 | 2 | 3 | 1 | 1 | 1 | 1 | 1 | 1 | 1 | 1 |
| 4154 | 66 | 1 | 167.5 | 69.8 | 2 | 2 | 1 | 2 | 5 | 1 | 1 | 1 | 1 | 1 | 1 | 1 | 3 | 1 |
| 4155 | 66 | 1 | 168 | 70 | 8 | 2 | 1 | 2 | 4 | 4 | 1 | 1 | 1 | 1 | 1 | 1 | 3 | 1 |
| 4156 | 66 | 1 | 168 | 69 | 8 | 2 | 2 | 1 | 3 | 3 | 1 | 1 | 1 | 1 | 1 | 1 | 1 | 1 |
| 4157 | 66 | 1 | 168 | 68.9 | 3 | 2 | 1 | 1 | 4 | 4 | 1 | 1 | 2 | 1 | 1 | 1 | 2 | 1 |
| 4158 | 66 | 1 | 168 | 52 | 8 | 2 | 1 | 1 | 4 | 4 | 1 | 1 | 2 | 1 | 1 | 1 | 1 | 1 |
| 4159 | 66 | 1 | 168.2 | 65 | 2 | 2 | 1 | 2 | 3 | 1 | 1 | 1 | 1 | 1 | 2 | 1 | 2 | 1 |
| 4160 | 66 | 1 | 168.5 | 57 | 3 | 2 | 1 | 3 | 3 | 3 | 1 | 1 | 1 | 1 | 1 | 1 | 2 | 1 |
| 4161 | 66 | 1 | 169 | 80 | 8 | 2 | 2 | 1 | 6 | 4 | 1 | 1 | 1 | 1 | 1 | 1 | 1 | 1 |
| 4162 | 66 | 1 | 169 | 70 | 8 | 2 | 1 | 1 | 4 | 4 | 2 | 1 | 2 | 1 | 1 | 1 | 1 | 1 |
| 4163 | 66 | 1 | 169 | 66.4 | 6 | 2 | 1 | 1 | 3 | 1 | 1 | 1 | 1 | 1 | 1 | 1 | 1 | 1 |
| 4164 | 66 | 1 | 169 | 55 | 2 | 2 | 1 | 2 | 5 | 1 | 1 | 1 | 1 | 1 | 1 | 1 | 2 | 1 |
| 4165 | 66 | 1 | 169.5 | 75 | 8 | 2 | 1 | 1 | 3 | 2 | 1 | 1 | 2 | 1 | 1 | 1 | 1 | 1 |
| 4166 | 66 | 1 | 170 | 73 | 8 | 1 | 1 | 1 | 3 | 3 | 1 | 1 | 1 | 1 | 1 | 1 | 1 | 1 |
| 4167 | 66 | 1 | 170 | 68 | 8 | 2 | 1 | 1 | 2 | 3 | 1 | 1 | 1 | 1 | 1 | 1 | 1 | 2 |
| 4168 | 66 | 1 | 170 | 66.5 | 8 | 2 | 1 | 1 | 5 | 2 | 1 | 1 | 1 | 1 | 1 | 1 | 1 | 1 |
| 4169 | 66 | 1 | 170 | 62.5 | 8 | 2 | 1 | 1 | 3 | 1 | 1 | 1 | 1 | 1 | 1 | 1 | 1 | 1 |
| 4170 | 66 | 1 | 170 | 60 | 2 | 2 | 1 | 1 | 4 | 4 | 1 | 1 | 1 | 1 | 1 | 1 | 1 | 2 |
| 4171 | 66 | 1 | 171 | 68 | 8 | 2 | 1 | 2 | 3 | 1 | 1 | 1 | 1 | 1 | 1 | 1 | 3 | 1 |
| 4172 | 66 | 1 | 171.5 | 58.3 | 6 | 2 | 1 | 2 | 4 | 4 | 1 | 1 | 1 | 1 | 1 | 1 | 3 | 1 |
| 4173 | 66 | 1 | 172 | 75.8 | 4 | 2 | 1 | 1 | 3 | 2 | 2 | 1 | 1 | 1 | 1 | 1 | 2 | 1 |
| 4174 | 66 | 1 | 172 | 75 | 4 | 2 | 1 | 4 | 3 | 4 | 1 | 1 | 1 | 1 | 1 | 1 | 3 | 2 |
| 4175 | 66 | 1 | 173 | 83 | 8 | 2 | 1 | 1 | 2 | 3 | 1 | 1 | 1 | 1 | 1 | 1 | 1 | 1 |
| 4176 | 66 | 1 | 173 | 59 | 8 | 2 | 1 | 1 | 4 | 2 | 1 | 1 | 1 | 1 | 1 | 1 | 3 | 1 |
| 4177 | 66 | 1 | 173.2 | 68 | 8 | 2 | 1 | 1 | 4 | 1 | 1 | 1 | 1 | 1 | 1 | 1 | 3 | 1 |
| 4178 | 66 | 1 | 174 | 70 | 8 | 2 | 1 | 1 | 1 | 1 | 1 | 1 | 1 | 1 | 1 | 1 | 1 | 1 |
| 4179 | 66 | 1 | 175 | 65 | 4 | 2 | 1 | 1 | 4 | 4 | 1 | 1 | 1 | 1 | 2 | 1 | 3 | 1 |
| 4180 | 66 | 1 | 176 | 85 | 8 | 2 | 2 | 1 | 3 | 1 | 1 | 1 | 1 | 1 | 1 | 1 | 1 | 1 |
| 4181 | 66 | 1 | 176 | 63 | 8 | 2 | 1 | 2 | 4 | 2 | 1 | 2 | 2 | 1 | 1 | 1 | 3 | 1 |
| 4182 | 66 | 1 | 177 | 64.4 | 8 | 2 | 1 | 2 | 5 | 1 | 1 | 1 | 2 | 1 | 1 | 1 | 3 | 1 |
| 4183 | 66 | 1 | 178 | 75 | 3 | 2 | 1 | 1 | 3 | 4 | 1 | 1 | 1 | 1 | 1 | 1 | 1 | 1 |
| 4184 | 67 | 2 | 110 | 21 | 8 | 1 | 1 | 1 | 2 | 4 | 1 | 1 | 2 | 1 | 1 | 1 | 1 | 1 |
| 4185 | 67 | 2 | 143 | 43 | 8 | 2 | 1 | 1 | 3 | 1 | 1 | 1 | 1 | 1 | 1 | 1 | 1 | 1 |
| 4186 | 67 | 2 | 145 | 49 | 2 | 2 | 1 | 1 | 4 | 2 | 1 | 1 | 1 | 1 | 2 | 1 | 1 | 1 |
| 4187 | 67 | 2 | 146 | 40 | 8 | 2 | 1 | 1 | 2 | 3 | 1 | 1 | 1 | 2 | 1 | 1 | 1 | 1 |
| 4188 | 67 | 2 | 147 | 88 | 4 | 2 | 1 | 1 | 3 | 1 | 1 | 1 | 1 | 1 | 2 | 2 | 1 | 1 |
| 4189 | 67 | 2 | 147 | 53 | 8 | 2 | 1 | 1 | 2 | 3 | 1 | 1 | 1 | 1 | 1 | 1 | 1 | 1 |
| 4190 | 67 | 2 | 147 | 52.2 | 8 | 2 | 1 | 1 | 3 | 1 | 1 | 1 | 1 | 1 | 1 | 1 | 1 | 1 |
| 4191 | 67 | 2 | 148 | 70 | 8 | 2 | 1 | 1 | 3 | 1 | 1 | 1 | 1 | 1 | 1 | 1 | 1 | 1 |
| 4192 | 67 | 2 | 148 | 52 | 4 | 2 | 1 | 1 | 4 | 4 | 1 | 1 | 2 | 1 | 1 | 1 | 1 | 1 |
| 4193 | 67 | 2 | 148.5 | 43 | 8 | 2 | 1 | 1 | 3 | 4 | 1 | 1 | 1 | 1 | 1 | 1 | 1 | 1 |
| 4194 | 67 | 2 | 149 | 62.5 | 8 | 3 | 1 | 1 | 3 | 4 | 1 | 1 | 1 | 1 | 2 | 1 | 1 | 1 |
| 4195 | 67 | 2 | 149.5 | 50 | 3 | 2 | 1 | 1 | 4 | 1 | 1 | 1 | 1 | 1 | 1 | 1 | 1 | 1 |
| 4196 | 67 | 2 | 149.5 | 45 | 8 | 2 | 1 | 1 | 3 | 1 | 1 | 1 | 1 | 1 | 2 | 1 | 1 | 1 |
| 4197 | 67 | 2 | 150 | 68 | 6 | 2 | 1 | 1 | 3 | 2 | 1 | 1 | 1 | 1 | 1 | 1 | 1 | 1 |
| 4198 | 67 | 2 | 150 | 64.5 | 6 | 2 | 1 | 1 | 3 | 1 | 1 | 1 | 1 | 1 | 1 | 1 | 1 | 1 |
| 4199 | 67 | 2 | 150 | 60.6 | 6 | 2 | 1 | 1 | 3 | 1 | 1 | 1 | 1 | 1 | 2 | 1 | 1 | 1 |
| 4200 | 67 | 2 | 150 | 60 | 8 | 4 | 1 | 1 | 2 | 4 | 1 | 1 | 2 | 1 | 1 | 1 | 1 | 1 |
| 4201 | 67 | 2 | 150 | 54 | 8 | 2 | 1 | 1 | 2 | 3 | 1 | 1 | 1 | 1 | 1 | 1 | 1 | 1 |
| 4202 | 67 | 2 | 150 | 54 | 1 | 2 | 1 | 1 | 5 | 1 | 1 | 1 | 1 | 1 | 2 | 1 | 1 | 1 |
| 4203 | 67 | 2 | 150 | 48 | 5 | 2 | 1 | 1 | 3 | 1 | 1 | 1 | 1 | 1 | 1 | 1 | 1 | 1 |
| 4204 | 67 | 2 | 150 | 41.5 | 8 | 2 | 1 | 1 | 3 | 1 | 1 | 1 | 1 | 1 | 1 | 1 | 1 | 1 |
| 4205 | 67 | 2 | 151 | 75 | 8 | 2 | 1 | 1 | 6 | 2 | 1 | 1 | 1 | 1 | 1 | 1 | 1 | 1 |
| 4206 | 67 | 2 | 151 | 49.5 | 8 | 2 | 1 | 1 | 4 | 1 | 1 | 1 | 1 | 1 | 1 | 1 | 1 | 1 |
| 4207 | 67 | 2 | 151.5 | 63.5 | 6 | 2 | 1 | 2 | 2 | 3 | 1 | 1 | 1 | 1 | 1 | 1 | 1 | 1 |
| 4208 | 67 | 2 | 152 | 68 | 3 | 2 | 1 | 1 | 4 | 1 | 1 | 1 | 1 | 1 | 1 | 1 | 1 | 1 |
| 4209 | 67 | 2 | 152 | 60 | 8 | 2 | 1 | 1 | 4 | 1 | 1 | 1 | 1 | 1 | 2 | 1 | 1 | 1 |
| 4210 | 67 | 2 | 152 | 59.2 | 4 | 2 | 1 | 1 | 4 | 3 | 1 | 1 | 1 | 1 | 1 | 1 | 1 | 1 |
| 4211 | 67 | 2 | 152 | 50 | 8 | 2 | 1 | 1 | 3 | 2 | 1 | 1 | 1 | 1 | 1 | 1 | 1 | 1 |
| 4212 | 67 | 2 | 152 | 46.5 | 8 | 2 | 1 | 1 | 3 | 4 | 1 | 1 | 1 | 1 | 2 | 1 | 1 | 2 |
| 4213 | 67 | 2 | 152 | 45 | 6 | 2 | 1 | 1 | 2 | 1 | 1 | 1 | 1 | 1 | 1 | 1 | 1 | 1 |
| 4214 | 67 | 2 | 153 | 71.5 | 3 | 2 | 1 | 1 | 3 | 1 | 1 | 1 | 1 | 1 | 1 | 1 | 1 | 1 |
| 4215 | 67 | 2 | 153 | 57 | 8 | 2 | 1 | 1 | 2 | 2 | 1 | 1 | 1 | 1 | 2 | 1 | 1 | 1 |
| 4216 | 67 | 2 | 153 | 53 | 8 | 2 | 1 | 1 | 3 | 1 | 1 | 1 | 1 | 1 | 1 | 1 | 1 | 1 |
| 4217 | 67 | 2 | 153.5 | 52 | 8 | 2 | 1 | 1 | 3 | 1 | 1 | 1 | 1 | 1 | 1 | 1 | 1 | 1 |
| 4218 | 67 | 2 | 154 | 70 | 3 | 2 | 1 | 1 | 5 | 2 | 1 | 1 | 1 | 1 | 1 | 1 | 1 | 1 |
| 4219 | 67 | 2 | 154 | 69.2 | 6 | 2 | 1 | 1 | 3 | 1 | 1 | 1 | 1 | 1 | 2 | 1 | 1 | 1 |
| 4220 | 67 | 2 | 154 | 65.9 | 2 | 2 | 1 | 1 | 4 | 2 | 1 | 1 | 1 | 1 | 2 | 1 | 1 | 1 |
| 4221 | 67 | 2 | 154 | 53 | 8 | 3 | 1 | 1 | 2 | 3 | 1 | 1 | 1 | 1 | 1 | 1 | 1 | 1 |
| 4222 | 67 | 2 | 154 | 52.5 | 8 | 2 | 1 | 1 | 3 | 1 | 1 | 1 | 1 | 1 | 1 | 1 | 1 | 1 |
| 4223 | 67 | 2 | 154.5 | 46.8 | 6 | 3 | 1 | 1 | 2 | 2 | 1 | 1 | 1 | 1 | 1 | 1 | 1 | 1 |
| 4224 | 67 | 2 | 155 | 65 | 4 | 2 | 1 | 1 | 2 | 1 | 1 | 1 | 1 | 1 | 2 | 1 | 1 | 1 |
| 4225 | 67 | 2 | 155 | 65 | 2 | 2 | 1 | 2 | 2 | 1 | 1 | 1 | 1 | 1 | 1 | 1 | 1 | 1 |
| 4226 | 67 | 2 | 155 | 58 | 8 | 2 | 1 | 1 | 3 | 1 | 1 | 1 | 2 | 1 | 1 | 1 | 1 | 1 |
| 4227 | 67 | 2 | 155 | 55.5 | 8 | 2 | 1 | 1 | 3 | 2 | 1 | 1 | 1 | 1 | 1 | 1 | 1 | 1 |
| 4228 | 67 | 2 | 155 | 55 | 1 | 2 | 1 | 1 | 4 | 1 | 1 | 1 | 1 | 1 | 1 | 1 | 1 | 1 |
| 4229 | 67 | 2 | 155 | 53 | 8 | 2 | 1 | 1 | 3 | 3 | 1 | 1 | 1 | 1 | 2 | 1 | 1 | 1 |
| 4230 | 67 | 2 | 155 | 52 | 8 | 2 | 1 | 1 | 4 | 1 | 1 | 1 | 1 | 1 | 2 | 1 | 1 | 1 |
| 4231 | 67 | 2 | 155 | 45.1 | 8 | 3 | 1 | 1 | 4 | 1 | 1 | 1 | 1 | 1 | 2 | 1 | 1 | 1 |
| 4232 | 67 | 2 | 156 | 62 | 8 | 3 | 1 | 1 | 4 | 3 | 1 | 1 | 2 | 1 | 1 | 1 | 1 | 1 |
| 4233 | 67 | 2 | 156 | 58 | 8 | 2 | 1 | 1 | 2 | 3 | 1 | 1 | 1 | 1 | 1 | 1 | 1 | 1 |
| 4234 | 67 | 2 | 156 | 57 | 8 | 2 | 1 | 1 | 2 | 1 | 1 | 1 | 1 | 1 | 1 | 1 | 1 | 1 |
| 4235 | 67 | 2 | 156 | 56 | 8 | 2 | 1 | 1 | 2 | 1 | 1 | 1 | 1 | 1 | 1 | 1 | 1 | 1 |
| 4236 | 67 | 2 | 156.5 | 49 | 4 | 3 | 1 | 1 | 2 | 1 | 1 | 1 | 1 | 1 | 2 | 1 | 1 | 1 |
| 4237 | 67 | 2 | 157 | 78 | 8 | 1 | 1 | 1 | 4 | 4 | 1 | 1 | 1 | 1 | 2 | 2 | 1 | 1 |
| 4238 | 67 | 2 | 157 | 61.9 | 6 | 2 | 1 | 1 | 3 | 4 | 1 | 1 | 1 | 1 | 2 | 1 | 1 | 1 |
| 4239 | 67 | 2 | 157 | 60 | 8 | 2 | 1 | 1 | 2 | 3 | 1 | 1 | 1 | 1 | 1 | 1 | 1 | 1 |
| 4240 | 67 | 2 | 157 | 56 | 8 | 3 | 1 | 1 | 3 | 4 | 1 | 1 | 2 | 1 | 1 | 1 | 1 | 1 |
| 4241 | 67 | 2 | 157.5 | 52.8 | 6 | 2 | 1 | 1 | 4 | 2 | 1 | 1 | 1 | 1 | 1 | 1 | 1 | 1 |
| 4242 | 67 | 2 | 158 | 64.6 | 3 | 2 | 1 | 1 | 3 | 1 | 1 | 1 | 1 | 1 | 1 | 1 | 1 | 1 |
| 4243 | 67 | 2 | 158 | 64 | 4 | 2 | 1 | 1 | 6 | 1 | 1 | 1 | 1 | 1 | 1 | 1 | 1 | 1 |
| 4244 | 67 | 2 | 158 | 60 | 6 | 2 | 1 | 1 | 3 | 1 | 1 | 1 | 1 | 1 | 1 | 1 | 1 | 1 |
| 4245 | 67 | 2 | 158 | 58 | 4 | 2 | 1 | 1 | 2 | 1 | 1 | 1 | 1 | 1 | 1 | 1 | 1 | 1 |
| 4246 | 67 | 2 | 158 | 53 | 8 | 2 | 1 | 1 | 4 | 4 | 1 | 1 | 1 | 1 | 1 | 1 | 1 | 1 |
| 4247 | 67 | 2 | 158.5 | 79.1 | 8 | 2 | 1 | 2 | 3 | 1 | 1 | 1 | 1 | 1 | 1 | 1 | 1 | 1 |
| 4248 | 67 | 2 | 159 | 61 | 8 | 2 | 1 | 1 | 2 | 3 | 1 | 1 | 1 | 1 | 1 | 1 | 1 | 1 |
| 4249 | 67 | 2 | 160 | 70 | 8 | 2 | 1 | 1 | 5 | 3 | 1 | 1 | 2 | 1 | 2 | 1 | 1 | 1 |
| 4250 | 67 | 2 | 160 | 58 | 2 | 2 | 1 | 1 | 4 | 3 | 1 | 1 | 1 | 1 | 1 | 1 | 1 | 1 |
| 4251 | 67 | 2 | 160 | 53 | 8 | 2 | 1 | 1 | 2 | 3 | 1 | 1 | 1 | 1 | 1 | 1 | 1 | 1 |
| 4252 | 67 | 2 | 160 | 52 | 8 | 2 | 1 | 1 | 2 | 3 | 1 | 1 | 1 | 1 | 1 | 1 | 1 | 1 |
| 4253 | 67 | 2 | 160 | 43.8 | 8 | 2 | 1 | 1 | 4 | 2 | 1 | 1 | 1 | 1 | 1 | 1 | 1 | 1 |
| 4254 | 67 | 2 | 160.5 | 63.2 | 2 | 2 | 1 | 1 | 4 | 2 | 2 | 1 | 1 | 1 | 1 | 1 | 1 | 1 |
| 4255 | 67 | 2 | 161 | 48 | 1 | 2 | 1 | 1 | 2 | 1 | 1 | 1 | 1 | 1 | 1 | 1 | 1 | 1 |
| 4256 | 67 | 2 | 162 | 67 | 8 | 2 | 1 | 1 | 3 | 4 | 1 | 1 | 1 | 2 | 2 | 1 | 1 | 1 |
| 4257 | 67 | 2 | 162 | 62 | 8 | 2 | 1 | 1 | 3 | 3 | 2 | 1 | 1 | 1 | 2 | 2 | 1 | 1 |
| 4258 | 67 | 2 | 162.5 | 59.4 | 4 | 2 | 1 | 3 | 4 | 1 | 1 | 1 | 1 | 1 | 2 | 1 | 1 | 1 |
| 4259 | 67 | 2 | 164 | 67.5 | 8 | 1 | 1 | 1 | 2 | 1 | 1 | 1 | 1 | 1 | 1 | 1 | 1 | 1 |
| 4260 | 67 | 2 | 164 | 55 | 8 | 2 | 1 | 1 | 6 | 1 | 1 | 1 | 2 | 1 | 1 | 1 | 1 | 1 |
| 4261 | 67 | 2 | 165 | 54 | 8 | 2 | 1 | 1 | 2 | 2 | 1 | 1 | 1 | 1 | 1 | 1 | 1 | 1 |
| 4262 | 67 | 2 | 168 | 58 | 8 | 2 | 1 | 1 | 5 | 2 | 1 | 1 | 1 | 1 | 1 | 1 | 1 | 1 |
| 4263 | 67 | 2 | 169 | 59 | 8 | 2 | 1 | 1 | 2 | 1 | 1 | 1 | 1 | 1 | 1 | 1 | 3 | 1 |
| 4264 | 67 | 1 | 142 | 37 | 8 | 2 | 1 | 1 | 3 | 1 | 1 | 1 | 1 | 1 | 1 | 1 | 1 | 1 |
| 4265 | 67 | 1 | 150 | 35.5 | 4 | 3 | 1 | 1 | 3 | 2 | 1 | 1 | 1 | 1 | 1 | 1 | 1 | 1 |
| 4266 | 67 | 1 | 155 | 52 | 8 | 2 | 1 | 1 | 2 | 3 | 1 | 1 | 1 | 1 | 1 | 1 | 1 | 1 |
| 4267 | 67 | 1 | 157.5 | 55 | 2 | 2 | 1 | 1 | 3 | 4 | 1 | 1 | 1 | 1 | 1 | 1 | 1 | 1 |
| 4268 | 67 | 1 | 158 | 65 | 8 | 2 | 1 | 1 | 2 | 3 | 1 | 1 | 1 | 1 | 1 | 1 | 1 | 1 |
| 4269 | 67 | 1 | 158 | 59 | 6 | 2 | 1 | 3 | 3 | 1 | 1 | 1 | 1 | 1 | 1 | 1 | 3 | 1 |
| 4270 | 67 | 1 | 158.5 | 71.2 | 8 | 2 | 1 | 1 | 4 | 2 | 2 | 1 | 1 | 1 | 1 | 1 | 2 | 1 |
| 4271 | 67 | 1 | 160 | 62.5 | 2 | 2 | 1 | 2 | 4 | 2 | 1 | 1 | 1 | 1 | 1 | 1 | 1 | 1 |
| 4272 | 67 | 1 | 160 | 60 | 1 | 1 | 2 | 1 | 1 | 1 | 1 | 1 | 1 | 1 | 1 | 1 | 1 | 1 |
| 4273 | 67 | 1 | 160 | 59 | 8 | 2 | 1 | 1 | 2 | 3 | 1 | 1 | 1 | 1 | 1 | 1 | 1 | 1 |
| 4274 | 67 | 1 | 160 | 52 | 6 | 2 | 1 | 1 | 4 | 2 | 1 | 1 | 1 | 1 | 1 | 1 | 1 | 1 |
| 4275 | 67 | 1 | 160 | 45.5 | 8 | 2 | 1 | 1 | 4 | 2 | 1 | 1 | 1 | 1 | 1 | 1 | 3 | 1 |
| 4276 | 67 | 1 | 161 | 61 | 8 | 2 | 1 | 1 | 2 | 1 | 1 | 1 | 2 | 1 | 1 | 1 | 1 | 1 |
| 4277 | 67 | 1 | 162 | 53 | 8 | 2 | 1 | 1 | 3 | 3 | 1 | 1 | 1 | 1 | 1 | 1 | 1 | 1 |
| 4278 | 67 | 1 | 162.8 | 55 | 6 | 2 | 1 | 4 | 2 | 2 | 1 | 1 | 1 | 1 | 2 | 1 | 3 | 1 |
| 4279 | 67 | 1 | 163 | 59 | 4 | 2 | 1 | 1 | 3 | 1 | 1 | 1 | 1 | 1 | 1 | 1 | 3 | 1 |
| 4280 | 67 | 1 | 163 | 49 | 8 | 2 | 1 | 1 | 3 | 1 | 1 | 1 | 1 | 1 | 2 | 2 | 1 | 1 |
| 4281 | 67 | 1 | 164 | 65 | 3 | 2 | 1 | 1 | 4 | 1 | 1 | 1 | 2 | 1 | 1 | 1 | 1 | 1 |
| 4282 | 67 | 1 | 164 | 56 | 8 | 2 | 1 | 1 | 4 | 4 | 1 | 1 | 1 | 2 | 1 | 1 | 1 | 2 |
| 4283 | 67 | 1 | 164.5 | 61.5 | 6 | 2 | 1 | 2 | 3 | 1 | 1 | 1 | 1 | 1 | 1 | 1 | 3 | 1 |
| 4284 | 67 | 1 | 165 | 64 | 8 | 2 | 1 | 1 | 4 | 3 | 1 | 1 | 1 | 1 | 1 | 1 | 1 | 1 |
| 4285 | 67 | 1 | 165 | 62 | 6 | 2 | 1 | 2 | 5 | 1 | 1 | 1 | 1 | 1 | 1 | 1 | 2 | 1 |
| 4286 | 67 | 1 | 165 | 58 | 4 | 2 | 1 | 2 | 3 | 1 | 1 | 1 | 1 | 1 | 1 | 1 | 1 | 1 |
| 4287 | 67 | 1 | 165 | 56 | 2 | 2 | 1 | 2 | 5 | 2 | 1 | 1 | 1 | 2 | 1 | 1 | 3 | 2 |
| 4288 | 67 | 1 | 166 | 65.7 | 2 | 2 | 1 | 1 | 2 | 1 | 1 | 1 | 2 | 1 | 1 | 1 | 3 | 1 |
| 4289 | 67 | 1 | 166 | 64.6 | 2 | 2 | 1 | 1 | 4 | 3 | 1 | 1 | 1 | 1 | 1 | 1 | 3 | 1 |
| 4290 | 67 | 1 | 166 | 54 | 8 | 2 | 1 | 1 | 3 | 1 | 1 | 1 | 1 | 1 | 1 | 1 | 1 | 1 |
| 4291 | 67 | 1 | 166.8 | 70.6 | 6 | 2 | 1 | 1 | 4 | 2 | 1 | 1 | 1 | 1 | 1 | 1 | 3 | 2 |
| 4292 | 67 | 1 | 167 | 69 | 8 | 2 | 1 | 1 | 3 | 3 | 1 | 1 | 1 | 1 | 1 | 1 | 1 | 1 |
| 4293 | 67 | 1 | 167 | 67.8 | 2 | 2 | 1 | 1 | 5 | 2 | 1 | 1 | 1 | 1 | 2 | 1 | 1 | 1 |
| 4294 | 67 | 1 | 167 | 64.5 | 8 | 2 | 1 | 1 | 3 | 2 | 2 | 1 | 1 | 2 | 1 | 1 | 1 | 2 |
| 4295 | 67 | 1 | 167 | 55 | 8 | 2 | 1 | 2 | 6 | 1 | 1 | 1 | 1 | 1 | 1 | 1 | 3 | 1 |
| 4296 | 67 | 1 | 167.5 | 58 | 8 | 2 | 1 | 1 | 1 | 4 | 1 | 1 | 1 | 1 | 1 | 1 | 3 | 1 |
| 4297 | 67 | 1 | 168 | 71 | 8 | 3 | 1 | 1 | 4 | 4 | 1 | 1 | 1 | 1 | 1 | 1 | 2 | 1 |
| 4298 | 67 | 1 | 169 | 77 | 8 | 2 | 1 | 1 | 2 | 3 | 1 | 1 | 1 | 1 | 1 | 1 | 1 | 1 |
| 4299 | 67 | 1 | 169 | 70 | 1 | 2 | 1 | 1 | 5 | 2 | 1 | 1 | 1 | 1 | 1 | 1 | 1 | 1 |
| 4300 | 67 | 1 | 169 | 56 | 8 | 2 | 1 | 1 | 2 | 3 | 1 | 1 | 1 | 1 | 1 | 1 | 1 | 1 |
| 4301 | 67 | 1 | 170 | 83.7 | 8 | 2 | 1 | 1 | 4 | 1 | 1 | 1 | 1 | 1 | 1 | 1 | 1 | 1 |
| 4302 | 67 | 1 | 170 | 78 | 8 | 2 | 1 | 1 | 5 | 4 | 1 | 1 | 2 | 1 | 1 | 1 | 1 | 1 |
| 4303 | 67 | 1 | 170 | 67 | 3 | 2 | 1 | 2 | 4 | 1 | 1 | 1 | 2 | 1 | 1 | 1 | 1 | 1 |
| 4304 | 67 | 1 | 170 | 65 | 6 | 2 | 1 | 1 | 3 | 4 | 1 | 1 | 2 | 1 | 1 | 1 | 1 | 1 |
| 4305 | 67 | 1 | 171 | 79.5 | 8 | 2 | 1 | 1 | 3 | 1 | 1 | 2 | 1 | 1 | 1 | 1 | 1 | 1 |
| 4306 | 67 | 1 | 172 | 65 | 2 | 2 | 1 | 1 | 3 | 2 | 1 | 1 | 1 | 1 | 1 | 1 | 1 | 1 |
| 4307 | 67 | 1 | 173 | 78 | 8 | 2 | 1 | 4 | 3 | 2 | 1 | 1 | 1 | 1 | 1 | 1 | 1 | 1 |
| 4308 | 67 | 1 | 174 | 64.4 | 2 | 1 | 1 | 2 | 3 | 3 | 1 | 1 | 1 | 1 | 1 | 1 | 3 | 1 |
| 4309 | 67 | 1 | 174 | 64 | 8 | 2 | 1 | 1 | 4 | 1 | 1 | 1 | 1 | 1 | 1 | 1 | 1 | 1 |
| 4310 | 67 | 1 | 185 | 78.5 | 8 | 2 | 1 | 1 | 3 | 1 | 1 | 1 | 1 | 1 | 1 | 1 | 1 | 1 |
| 4311 | 68 | 2 | 139 | 44 | 8 | 2 | 1 | 1 | 2 | 2 | 1 | 1 | 1 | 1 | 1 | 1 | 1 | 1 |
| 4312 | 68 | 2 | 141 | 63.5 | 8 | 2 | 1 | 1 | 2 | 4 | 1 | 1 | 1 | 1 | 1 | 1 | 1 | 1 |
| 4313 | 68 | 2 | 145.5 | 47.2 | 4 | 2 | 1 | 1 | 2 | 1 | 1 | 1 | 1 | 1 | 1 | 1 | 1 | 1 |
| 4314 | 68 | 2 | 146 | 53 | 8 | 3 | 1 | 1 | 2 | 1 | 1 | 1 | 1 | 1 | 2 | 1 | 1 | 1 |
| 4315 | 68 | 2 | 147 | 63 | 8 | 2 | 1 | 1 | 2 | 1 | 1 | 1 | 1 | 1 | 1 | 1 | 1 | 1 |
| 4316 | 68 | 2 | 147 | 56 | 8 | 2 | 2 | 1 | 2 | 3 | 1 | 1 | 1 | 2 | 1 | 1 | 1 | 1 |
| 4317 | 68 | 2 | 147.5 | 57 | 8 | 3 | 1 | 1 | 3 | 4 | 1 | 1 | 1 | 1 | 1 | 1 | 1 | 1 |
| 4318 | 68 | 2 | 147.8 | 56 | 4 | 2 | 1 | 1 | 3 | 1 | 1 | 1 | 1 | 1 | 1 | 1 | 1 | 1 |
| 4319 | 68 | 2 | 148 | 63 | 8 | 2 | 1 | 1 | 4 | 1 | 1 | 1 | 1 | 1 | 2 | 1 | 1 | 1 |
| 4320 | 68 | 2 | 148 | 58 | 2 | 2 | 1 | 1 | 2 | 2 | 1 | 1 | 1 | 1 | 1 | 1 | 1 | 1 |
| 4321 | 68 | 2 | 148 | 57.5 | 8 | 2 | 1 | 1 | 2 | 2 | 1 | 1 | 1 | 1 | 1 | 1 | 1 | 1 |
| 4322 | 68 | 2 | 148 | 45 | 8 | 2 | 1 | 1 | 6 | 3 | 1 | 1 | 1 | 1 | 1 | 1 | 1 | 1 |
| 4323 | 68 | 2 | 148.6 | 66.9 | 6 | 3 | 1 | 1 | 2 | 1 | 1 | 1 | 1 | 1 | 1 | 1 | 1 | 1 |
| 4324 | 68 | 2 | 149 | 60 | 1 | 2 | 1 | 1 | 4 | 2 | 1 | 1 | 1 | 1 | 1 | 1 | 1 | 1 |
| 4325 | 68 | 2 | 149 | 45 | 8 | 3 | 1 | 1 | 4 | 1 | 1 | 1 | 1 | 1 | 2 | 1 | 1 | 1 |
| 4326 | 68 | 2 | 149.5 | 51.5 | 2 | 2 | 1 | 1 | 3 | 2 | 1 | 1 | 1 | 1 | 2 | 1 | 1 | 1 |
| 4327 | 68 | 2 | 150 | 60 | 8 | 2 | 1 | 1 | 3 | 1 | 1 | 1 | 2 | 1 | 1 | 1 | 1 | 1 |
| 4328 | 68 | 2 | 150 | 58 | 4 | 2 | 1 | 2 | 4 | 1 | 1 | 1 | 1 | 1 | 1 | 1 | 1 | 1 |
| 4329 | 68 | 2 | 150 | 57.5 | 4 | 3 | 1 | 1 | 3 | 1 | 1 | 1 | 1 | 1 | 1 | 1 | 1 | 1 |
| 4330 | 68 | 2 | 150 | 56.5 | 8 | 2 | 1 | 1 | 3 | 1 | 1 | 1 | 2 | 1 | 1 | 1 | 1 | 1 |
| 4331 | 68 | 2 | 150 | 52 | 2 | 2 | 1 | 1 | 3 | 1 | 1 | 1 | 1 | 1 | 2 | 1 | 1 | 1 |
| 4332 | 68 | 2 | 150 | 51 | 8 | 2 | 1 | 1 | 1 | 4 | 1 | 1 | 1 | 1 | 1 | 1 | 1 | 1 |
| 4333 | 68 | 2 | 150 | 42 | 8 | 3 | 1 | 1 | 3 | 2 | 1 | 1 | 1 | 1 | 1 | 1 | 1 | 1 |
| 4334 | 68 | 2 | 152 | 62 | 6 | 2 | 1 | 1 | 2 | 1 | 1 | 1 | 1 | 1 | 1 | 1 | 1 | 1 |
| 4335 | 68 | 2 | 152 | 51 | 3 | 2 | 1 | 1 | 4 | 4 | 1 | 1 | 1 | 1 | 1 | 1 | 1 | 1 |
| 4336 | 68 | 2 | 152 | 45 | 2 | 2 | 1 | 1 | 3 | 1 | 1 | 1 | 2 | 1 | 1 | 1 | 1 | 1 |
| 4337 | 68 | 2 | 152 | 40.5 | 8 | 2 | 1 | 1 | 2 | 2 | 1 | 1 | 1 | 1 | 1 | 1 | 1 | 1 |
| 4338 | 68 | 2 | 153 | 99 | 8 | 2 | 2 | 1 | 2 | 2 | 1 | 1 | 1 | 1 | 1 | 1 | 1 | 1 |
| 4339 | 68 | 2 | 153 | 52.5 | 8 | 2 | 1 | 1 | 4 | 1 | 1 | 1 | 1 | 1 | 2 | 1 | 1 | 1 |
| 4340 | 68 | 2 | 154 | 63 | 8 | 2 | 2 | 1 | 2 | 2 | 1 | 1 | 1 | 1 | 1 | 1 | 1 | 1 |
| 4341 | 68 | 2 | 154 | 56.5 | 8 | 2 | 1 | 1 | 4 | 2 | 1 | 1 | 1 | 1 | 1 | 1 | 1 | 1 |
| 4342 | 68 | 2 | 154 | 55.5 | 8 | 2 | 1 | 1 | 3 | 1 | 1 | 1 | 1 | 1 | 1 | 1 | 1 | 1 |
| 4343 | 68 | 2 | 154 | 55 | 4 | 3 | 1 | 1 | 4 | 1 | 1 | 1 | 1 | 1 | 1 | 1 | 1 | 1 |
| 4344 | 68 | 2 | 154.5 | 67.5 | 8 | 2 | 1 | 1 | 6 | 1 | 1 | 1 | 1 | 1 | 1 | 1 | 1 | 1 |
| 4345 | 68 | 2 | 154.8 | 71.5 | 8 | 2 | 1 | 1 | 3 | 1 | 1 | 1 | 1 | 1 | 1 | 1 | 1 | 1 |
| 4346 | 68 | 2 | 155 | 73.5 | 2 | 2 | 1 | 1 | 6 | 1 | 1 | 1 | 1 | 1 | 1 | 1 | 1 | 1 |
| 4347 | 68 | 2 | 155 | 71 | 8 | 2 | 1 | 1 | 2 | 3 | 1 | 1 | 1 | 1 | 1 | 1 | 1 | 1 |
| 4348 | 68 | 2 | 155 | 67 | 8 | 2 | 1 | 1 | 2 | 3 | 1 | 1 | 1 | 1 | 1 | 1 | 1 | 1 |
| 4349 | 68 | 2 | 155 | 59.5 | 8 | 3 | 1 | 1 | 1 | 1 | 1 | 1 | 1 | 1 | 1 | 1 | 1 | 1 |
| 4350 | 68 | 2 | 155 | 49 | 8 | 2 | 1 | 1 | 2 | 3 | 1 | 1 | 1 | 1 | 1 | 1 | 1 | 1 |
| 4351 | 68 | 2 | 156 | 65 | 3 | 2 | 1 | 1 | 4 | 1 | 1 | 1 | 1 | 1 | 1 | 1 | 1 | 1 |
| 4352 | 68 | 2 | 156 | 57 | 8 | 2 | 1 | 1 | 2 | 2 | 1 | 1 | 1 | 2 | 1 | 1 | 1 | 2 |
| 4353 | 68 | 2 | 156 | 55 | 8 | 2 | 1 | 1 | 4 | 4 | 1 | 1 | 1 | 1 | 1 | 1 | 1 | 1 |
| 4354 | 68 | 2 | 157 | 54 | 8 | 2 | 1 | 1 | 2 | 3 | 1 | 1 | 1 | 2 | 1 | 1 | 1 | 1 |
| 4355 | 68 | 2 | 158 | 80 | 8 | 2 | 1 | 1 | 2 | 2 | 1 | 1 | 1 | 1 | 1 | 1 | 1 | 1 |
| 4356 | 68 | 2 | 158 | 70 | 8 | 2 | 1 | 1 | 2 | 1 | 1 | 1 | 1 | 1 | 1 | 1 | 1 | 1 |
| 4357 | 68 | 2 | 158 | 55 | 8 | 3 | 1 | 1 | 4 | 1 | 1 | 1 | 1 | 1 | 2 | 1 | 1 | 1 |
| 4358 | 68 | 2 | 159 | 44 | 8 | 2 | 1 | 1 | 1 | 4 | 1 | 1 | 1 | 1 | 1 | 1 | 1 | 1 |
| 4359 | 68 | 2 | 159.5 | 67 | 8 | 3 | 1 | 1 | 5 | 4 | 1 | 1 | 1 | 1 | 1 | 1 | 1 | 1 |
| 4360 | 68 | 2 | 159.5 | 63 | 6 | 2 | 1 | 1 | 2 | 4 | 1 | 1 | 1 | 1 | 2 | 1 | 1 | 1 |
| 4361 | 68 | 2 | 160 | 85 | 8 | 2 | 1 | 1 | 2 | 4 | 1 | 1 | 1 | 1 | 2 | 1 | 1 | 1 |
| 4362 | 68 | 2 | 160 | 75 | 8 | 2 | 1 | 1 | 2 | 3 | 1 | 1 | 1 | 1 | 1 | 1 | 1 | 1 |
| 4363 | 68 | 2 | 160 | 70 | 8 | 2 | 1 | 1 | 2 | 4 | 1 | 1 | 1 | 1 | 2 | 1 | 1 | 1 |
| 4364 | 68 | 2 | 160 | 54 | 8 | 3 | 1 | 1 | 3 | 1 | 1 | 1 | 1 | 1 | 1 | 1 | 1 | 1 |
| 4365 | 68 | 2 | 160 | 50 | 8 | 2 | 1 | 1 | 2 | 3 | 1 | 1 | 1 | 1 | 1 | 1 | 1 | 1 |
| 4366 | 68 | 2 | 162 | 65 | 8 | 2 | 1 | 1 | 3 | 2 | 1 | 1 | 1 | 1 | 1 | 1 | 1 | 1 |
| 4367 | 68 | 2 | 162 | 64 | 8 | 2 | 1 | 1 | 4 | 4 | 1 | 1 | 1 | 2 | 2 | 1 | 1 | 1 |
| 4368 | 68 | 2 | 162 | 52 | 8 | 2 | 2 | 1 | 4 | 4 | 1 | 1 | 1 | 1 | 1 | 1 | 1 | 1 |
| 4369 | 68 | 2 | 162 | 45 | 8 | 3 | 1 | 1 | 2 | 1 | 1 | 1 | 1 | 1 | 1 | 1 | 1 | 2 |
| 4370 | 68 | 2 | 168 | 75 | 8 | 2 | 1 | 1 | 4 | 1 | 1 | 1 | 1 | 1 | 1 | 1 | 1 | 1 |
| 4371 | 68 | 2 | 175 | 81 | 8 | 2 | 1 | 1 | 2 | 1 | 1 | 1 | 1 | 1 | 2 | 1 | 1 | 1 |
| 4372 | 68 | 1 | 152 | 42 | 8 | 2 | 1 | 1 | 2 | 4 | 1 | 1 | 1 | 1 | 2 | 1 | 1 | 1 |
| 4373 | 68 | 1 | 155 | 68 | 2 | 2 | 1 | 1 | 2 | 1 | 1 | 1 | 1 | 1 | 1 | 1 | 1 | 1 |
| 4374 | 68 | 1 | 155 | 59 | 8 | 2 | 1 | 1 | 3 | 1 | 1 | 1 | 1 | 1 | 1 | 1 | 1 | 1 |
| 4375 | 68 | 1 | 155 | 53 | 8 | 2 | 1 | 1 | 3 | 4 | 1 | 1 | 1 | 1 | 1 | 1 | 1 | 1 |
| 4376 | 68 | 1 | 155 | 41 | 8 | 2 | 1 | 1 | 2 | 3 | 1 | 1 | 1 | 1 | 1 | 1 | 3 | 1 |
| 4377 | 68 | 1 | 157 | 54 | 4 | 2 | 1 | 1 | 4 | 2 | 1 | 1 | 1 | 1 | 1 | 1 | 1 | 1 |
| 4378 | 68 | 1 | 158 | 63 | 8 | 2 | 1 | 1 | 6 | 4 | 1 | 1 | 1 | 1 | 1 | 1 | 1 | 1 |
| 4379 | 68 | 1 | 158 | 62 | 8 | 2 | 1 | 1 | 3 | 3 | 1 | 1 | 1 | 1 | 1 | 1 | 1 | 1 |
| 4380 | 68 | 1 | 158 | 58 | 8 | 2 | 1 | 1 | 2 | 1 | 1 | 1 | 1 | 1 | 1 | 1 | 2 | 1 |
| 4381 | 68 | 1 | 159.5 | 53 | 8 | 2 | 1 | 1 | 5 | 1 | 1 | 1 | 1 | 1 | 2 | 1 | 2 | 1 |
| 4382 | 68 | 1 | 160 | 71 | 8 | 2 | 1 | 1 | 2 | 3 | 1 | 1 | 1 | 1 | 1 | 1 | 1 | 1 |
| 4383 | 68 | 1 | 160 | 67.4 | 2 | 2 | 1 | 1 | 3 | 3 | 1 | 1 | 1 | 1 | 2 | 1 | 2 | 1 |
| 4384 | 68 | 1 | 160 | 63 | 8 | 2 | 1 | 1 | 3 | 1 | 1 | 1 | 1 | 1 | 1 | 1 | 1 | 1 |
| 4385 | 68 | 1 | 160 | 57 | 8 | 2 | 1 | 1 | 2 | 4 | 1 | 1 | 1 | 1 | 1 | 1 | 1 | 1 |
| 4386 | 68 | 1 | 161 | 57 | 1 | 2 | 1 | 1 | 4 | 1 | 1 | 1 | 1 | 1 | 1 | 1 | 1 | 1 |
| 4387 | 68 | 1 | 162 | 72 | 8 | 2 | 2 | 1 | 4 | 2 | 1 | 1 | 1 | 1 | 1 | 1 | 1 | 1 |
| 4388 | 68 | 1 | 162 | 66 | 8 | 2 | 1 | 1 | 3 | 1 | 1 | 1 | 1 | 1 | 1 | 1 | 1 | 1 |
| 4389 | 68 | 1 | 162 | 64 | 5 | 2 | 1 | 1 | 2 | 1 | 1 | 1 | 1 | 1 | 2 | 1 | 1 | 1 |
| 4390 | 68 | 1 | 162 | 60 | 8 | 2 | 1 | 1 | 2 | 3 | 1 | 1 | 1 | 1 | 1 | 1 | 1 | 1 |
| 4391 | 68 | 1 | 162 | 58 | 8 | 2 | 1 | 2 | 3 | 2 | 1 | 1 | 1 | 1 | 2 | 1 | 1 | 1 |
| 4392 | 68 | 1 | 162 | 55.2 | 8 | 2 | 1 | 1 | 2 | 1 | 1 | 1 | 1 | 1 | 2 | 1 | 1 | 1 |
| 4393 | 68 | 1 | 163 | 66 | 6 | 2 | 1 | 1 | 4 | 1 | 1 | 2 | 2 | 1 | 2 | 1 | 1 | 1 |
| 4394 | 68 | 1 | 164 | 64 | 6 | 2 | 1 | 1 | 3 | 1 | 1 | 1 | 1 | 1 | 1 | 1 | 1 | 1 |
| 4395 | 68 | 1 | 164 | 63 | 8 | 2 | 1 | 1 | 3 | 2 | 1 | 1 | 1 | 1 | 2 | 1 | 1 | 1 |
| 4396 | 68 | 1 | 165 | 59 | 8 | 2 | 1 | 1 | 2 | 2 | 1 | 1 | 1 | 1 | 1 | 1 | 1 | 1 |
| 4397 | 68 | 1 | 165 | 51 | 8 | 2 | 1 | 1 | 1 | 3 | 1 | 1 | 1 | 1 | 2 | 1 | 1 | 1 |
| 4398 | 68 | 1 | 165.5 | 55.9 | 8 | 2 | 1 | 1 | 5 | 1 | 1 | 1 | 1 | 1 | 1 | 1 | 1 | 1 |
| 4399 | 68 | 1 | 166 | 74 | 2 | 2 | 1 | 1 | 4 | 2 | 1 | 1 | 1 | 2 | 1 | 1 | 1 | 2 |
| 4400 | 68 | 1 | 166 | 72 | 2 | 2 | 1 | 1 | 5 | 4 | 1 | 1 | 1 | 1 | 1 | 1 | 1 | 1 |
| 4401 | 68 | 1 | 166 | 69 | 8 | 2 | 1 | 2 | 3 | 1 | 1 | 1 | 1 | 1 | 1 | 1 | 3 | 1 |
| 4402 | 68 | 1 | 166 | 67 | 8 | 2 | 1 | 1 | 3 | 3 | 1 | 1 | 1 | 1 | 1 | 1 | 3 | 1 |
| 4403 | 68 | 1 | 166 | 65 | 2 | 2 | 1 | 1 | 5 | 1 | 1 | 1 | 1 | 1 | 1 | 1 | 3 | 1 |
| 4404 | 68 | 1 | 167 | 79 | 2 | 2 | 1 | 1 | 2 | 2 | 1 | 1 | 1 | 1 | 1 | 1 | 1 | 1 |
| 4405 | 68 | 1 | 168 | 83 | 8 | 2 | 1 | 1 | 4 | 1 | 1 | 1 | 1 | 1 | 1 | 1 | 1 | 1 |
| 4406 | 68 | 1 | 168 | 79.5 | 8 | 2 | 1 | 1 | 4 | 3 | 1 | 1 | 1 | 1 | 1 | 1 | 2 | 1 |
| 4407 | 68 | 1 | 168 | 69 | 3 | 2 | 1 | 2 | 6 | 1 | 1 | 1 | 1 | 1 | 1 | 1 | 1 | 2 |
| 4408 | 68 | 1 | 168 | 68 | 3 | 2 | 1 | 1 | 4 | 3 | 1 | 1 | 2 | 1 | 1 | 1 | 1 | 1 |
| 4409 | 68 | 1 | 169 | 73.4 | 6 | 2 | 1 | 3 | 4 | 2 | 1 | 1 | 2 | 1 | 1 | 1 | 1 | 1 |
| 4410 | 68 | 1 | 170 | 69.5 | 8 | 2 | 1 | 1 | 4 | 2 | 1 | 1 | 1 | 1 | 1 | 1 | 1 | 2 |
| 4411 | 68 | 1 | 170 | 65 | 8 | 2 | 1 | 1 | 3 | 1 | 1 | 1 | 1 | 1 | 1 | 1 | 3 | 1 |
| 4412 | 68 | 1 | 170 | 20 | 8 | 2 | 1 | 1 | 4 | 4 | 1 | 1 | 1 | 1 | 1 | 1 | 1 | 1 |
| 4413 | 68 | 1 | 170.6 | 90.6 | 2 | 2 | 1 | 1 | 4 | 2 | 1 | 1 | 1 | 1 | 1 | 1 | 2 | 1 |
| 4414 | 68 | 1 | 171 | 69 | 8 | 2 | 1 | 1 | 6 | 3 | 1 | 1 | 1 | 1 | 1 | 1 | 1 | 1 |
| 4415 | 68 | 1 | 171 | 67.5 | 2 | 2 | 1 | 1 | 5 | 2 | 1 | 1 | 2 | 1 | 1 | 1 | 1 | 1 |
| 4416 | 68 | 1 | 172 | 83.4 | 4 | 2 | 1 | 3 | 3 | 3 | 1 | 1 | 1 | 1 | 1 | 1 | 3 | 1 |
| 4417 | 68 | 1 | 172 | 83 | 8 | 2 | 1 | 1 | 2 | 3 | 1 | 1 | 1 | 1 | 1 | 1 | 1 | 1 |
| 4418 | 68 | 1 | 173 | 75 | 8 | 2 | 1 | 3 | 3 | 1 | 1 | 1 | 1 | 1 | 2 | 1 | 3 | 1 |
| 4419 | 68 | 1 | 173 | 67 | 8 | 2 | 1 | 2 | 4 | 1 | 1 | 1 | 1 | 1 | 1 | 1 | 2 | 1 |
| 4420 | 69 | 2 | 142 | 33.5 | 8 | 1 | 1 | 1 | 3 | 4 | 1 | 1 | 1 | 1 | 1 | 1 | 1 | 1 |
| 4421 | 69 | 2 | 145 | 48 | 8 | 2 | 1 | 1 | 3 | 1 | 1 | 1 | 1 | 1 | 2 | 1 | 1 | 1 |
| 4422 | 69 | 2 | 145 | 46 | 8 | 2 | 1 | 1 | 6 | 2 | 1 | 1 | 1 | 1 | 1 | 1 | 1 | 1 |
| 4423 | 69 | 2 | 146 | 50 | 4 | 2 | 1 | 1 | 3 | 1 | 1 | 1 | 1 | 1 | 1 | 1 | 1 | 1 |
| 4424 | 69 | 2 | 146 | 46 | 8 | 3 | 1 | 1 | 2 | 2 | 1 | 1 | 1 | 1 | 1 | 1 | 1 | 1 |
| 4425 | 69 | 2 | 147 | 63 | 2 | 2 | 1 | 1 | 3 | 1 | 2 | 1 | 1 | 2 | 1 | 1 | 1 | 2 |
| 4426 | 69 | 2 | 147 | 57.5 | 8 | 2 | 1 | 1 | 2 | 1 | 1 | 1 | 1 | 1 | 1 | 1 | 1 | 1 |
| 4427 | 69 | 2 | 147.5 | 51.5 | 8 | 2 | 1 | 1 | 4 | 1 | 1 | 1 | 1 | 1 | 1 | 1 | 1 | 1 |
| 4428 | 69 | 2 | 148 | 71 | 2 | 2 | 1 | 1 | 4 | 1 | 1 | 1 | 2 | 1 | 1 | 1 | 1 | 1 |
| 4429 | 69 | 2 | 148 | 45.5 | 2 | 3 | 1 | 2 | 5 | 1 | 1 | 1 | 1 | 1 | 1 | 2 | 1 | 1 |
| 4430 | 69 | 2 | 148.5 | 58.1 | 8 | 3 | 1 | 1 | 2 | 3 | 1 | 1 | 1 | 1 | 1 | 1 | 1 | 1 |
| 4431 | 69 | 2 | 149 | 53 | 8 | 3 | 1 | 1 | 3 | 4 | 1 | 1 | 1 | 1 | 2 | 1 | 1 | 1 |
| 4432 | 69 | 2 | 150 | 58.5 | 8 | 3 | 1 | 1 | 2 | 1 | 1 | 1 | 1 | 1 | 1 | 1 | 1 | 1 |
| 4433 | 69 | 2 | 150 | 55 | 8 | 2 | 1 | 1 | 2 | 1 | 1 | 1 | 1 | 1 | 1 | 1 | 1 | 1 |
| 4434 | 69 | 2 | 150 | 50 | 3 | 2 | 1 | 1 | 6 | 1 | 1 | 1 | 1 | 1 | 1 | 1 | 1 | 1 |
| 4435 | 69 | 2 | 150 | 48.5 | 8 | 3 | 1 | 1 | 3 | 2 | 1 | 1 | 1 | 1 | 1 | 1 | 1 | 1 |
| 4436 | 69 | 2 | 151 | 45 | 6 | 2 | 1 | 1 | 3 | 1 | 1 | 1 | 1 | 1 | 1 | 1 | 1 | 1 |
| 4437 | 69 | 2 | 152 | 55 | 8 | 2 | 1 | 1 | 3 | 2 | 1 | 1 | 1 | 1 | 1 | 1 | 1 | 2 |
| 4438 | 69 | 2 | 152 | 50 | 8 | 2 | 1 | 1 | 3 | 1 | 1 | 1 | 1 | 1 | 1 | 1 | 1 | 1 |
| 4439 | 69 | 2 | 152 | 50 | 8 | 2 | 1 | 1 | 2 | 1 | 1 | 1 | 1 | 1 | 1 | 1 | 1 | 2 |
| 4440 | 69 | 2 | 152 | 48 | 8 | 2 | 1 | 1 | 2 | 3 | 1 | 1 | 1 | 2 | 1 | 1 | 1 | 2 |
| 4441 | 69 | 2 | 152 | 45 | 8 | 2 | 1 | 1 | 5 | 2 | 1 | 1 | 1 | 1 | 1 | 1 | 1 | 1 |
| 4442 | 69 | 2 | 152.5 | 51 | 8 | 3 | 1 | 1 | 4 | 1 | 1 | 1 | 1 | 1 | 1 | 1 | 1 | 1 |
| 4443 | 69 | 2 | 153 | 57.2 | 5 | 2 | 1 | 1 | 1 | 2 | 2 | 1 | 1 | 1 | 1 | 1 | 1 | 1 |
| 4444 | 69 | 2 | 153.5 | 58 | 8 | 2 | 1 | 1 | 2 | 4 | 1 | 1 | 1 | 1 | 1 | 1 | 1 | 1 |
| 4445 | 69 | 2 | 154 | 48 | 3 | 2 | 1 | 1 | 3 | 1 | 1 | 1 | 1 | 1 | 1 | 1 | 1 | 1 |
| 4446 | 69 | 2 | 155 | 60 | 8 | 2 | 1 | 1 | 2 | 3 | 1 | 1 | 1 | 1 | 2 | 1 | 1 | 1 |
| 4447 | 69 | 2 | 155 | 57 | 8 | 2 | 1 | 1 | 6 | 3 | 1 | 1 | 1 | 1 | 2 | 2 | 1 | 1 |
| 4448 | 69 | 2 | 155 | 51 | 8 | 2 | 1 | 1 | 4 | 3 | 1 | 1 | 1 | 1 | 1 | 1 | 1 | 1 |
| 4449 | 69 | 2 | 155.5 | 62.5 | 4 | 3 | 1 | 1 | 3 | 1 | 1 | 1 | 1 | 1 | 1 | 1 | 1 | 1 |
| 4450 | 69 | 2 | 156 | 64.5 | 8 | 2 | 1 | 1 | 3 | 1 | 1 | 1 | 1 | 1 | 1 | 1 | 1 | 1 |
| 4451 | 69 | 2 | 156 | 61.5 | 8 | 2 | 2 | 1 | 4 | 1 | 1 | 1 | 1 | 1 | 1 | 1 | 1 | 1 |
| 4452 | 69 | 2 | 156 | 56 | 6 | 2 | 1 | 1 | 3 | 4 | 1 | 1 | 1 | 1 | 1 | 1 | 1 | 1 |
| 4453 | 69 | 2 | 156 | 52 | 8 | 3 | 1 | 1 | 3 | 1 | 1 | 1 | 1 | 1 | 2 | 1 | 1 | 1 |
| 4454 | 69 | 2 | 157 | 78.5 | 8 | 3 | 1 | 1 | 4 | 2 | 1 | 1 | 2 | 1 | 1 | 1 | 1 | 1 |
| 4455 | 69 | 2 | 158 | 69.5 | 8 | 2 | 1 | 1 | 4 | 1 | 1 | 1 | 1 | 1 | 1 | 1 | 1 | 1 |
| 4456 | 69 | 2 | 158 | 55 | 8 | 2 | 1 | 1 | 4 | 4 | 1 | 1 | 1 | 1 | 1 | 1 | 1 | 1 |
| 4457 | 69 | 2 | 158 | 45 | 8 | 1 | 1 | 1 | 3 | 4 | 1 | 1 | 1 | 1 | 1 | 1 | 1 | 1 |
| 4458 | 69 | 2 | 159 | 52 | 6 | 2 | 1 | 1 | 3 | 1 | 1 | 1 | 1 | 1 | 1 | 1 | 1 | 1 |
| 4459 | 69 | 2 | 160 | 73 | 8 | 2 | 1 | 1 | 4 | 3 | 1 | 1 | 1 | 1 | 1 | 1 | 1 | 1 |
| 4460 | 69 | 2 | 160 | 56.5 | 8 | 2 | 1 | 1 | 2 | 2 | 1 | 1 | 1 | 1 | 1 | 1 | 1 | 1 |
| 4461 | 69 | 2 | 160.5 | 57.7 | 3 | 2 | 1 | 1 | 4 | 1 | 1 | 1 | 1 | 1 | 2 | 2 | 1 | 1 |
| 4462 | 69 | 2 | 162 | 65 | 4 | 2 | 1 | 1 | 4 | 1 | 1 | 1 | 1 | 1 | 1 | 1 | 1 | 1 |
| 4463 | 69 | 2 | 162 | 59 | 2 | 4 | 1 | 1 | 4 | 1 | 1 | 1 | 1 | 1 | 1 | 1 | 1 | 1 |
| 4464 | 69 | 2 | 162 | 53 | 8 | 3 | 1 | 1 | 2 | 1 | 1 | 1 | 1 | 1 | 2 | 1 | 1 | 1 |
| 4465 | 69 | 2 | 163 | 53 | 8 | 3 | 1 | 2 | 3 | 1 | 1 | 1 | 1 | 1 | 1 | 1 | 1 | 1 |
| 4466 | 69 | 2 | 163 | 51.8 | 6 | 2 | 1 | 1 | 2 | 1 | 1 | 1 | 1 | 1 | 1 | 1 | 1 | 1 |
| 4467 | 69 | 2 | 164 | 44.5 | 8 | 3 | 1 | 1 | 3 | 1 | 1 | 1 | 1 | 1 | 1 | 1 | 1 | 1 |
| 4468 | 69 | 2 | 165 | 76.8 | 8 | 2 | 1 | 1 | 1 | 4 | 1 | 1 | 1 | 1 | 2 | 2 | 1 | 1 |
| 4469 | 69 | 2 | 165 | 60 | 8 | 3 | 1 | 1 | 2 | 1 | 1 | 1 | 1 | 1 | 1 | 1 | 1 | 1 |
| 4470 | 69 | 2 | 167 | 61 | 6 | 2 | 1 | 1 | 3 | 1 | 1 | 1 | 2 | 1 | 2 | 1 | 1 | 1 |
| 4471 | 69 | 2 | 170 | 60 | 8 | 2 | 1 | 1 | 4 | 1 | 2 | 1 | 1 | 1 | 1 | 1 | 1 | 1 |
| 4472 | 69 | 1 | 152 | 58 | 6 | 3 | 1 | 1 | 3 | 1 | 1 | 1 | 1 | 1 | 1 | 1 | 3 | 1 |
| 4473 | 69 | 1 | 153 | 57 | 2 | 2 | 1 | 1 | 2 | 1 | 1 | 1 | 1 | 1 | 1 | 1 | 1 | 1 |
| 4474 | 69 | 1 | 157.5 | 68 | 8 | 3 | 1 | 1 | 3 | 2 | 1 | 1 | 1 | 1 | 2 | 1 | 1 | 1 |
| 4475 | 69 | 1 | 158 | 75 | 8 | 2 | 1 | 2 | 2 | 2 | 1 | 1 | 1 | 1 | 2 | 1 | 2 | 1 |
| 4476 | 69 | 1 | 158 | 67.6 | 3 | 2 | 1 | 3 | 3 | 1 | 1 | 1 | 1 | 1 | 1 | 1 | 1 | 1 |
| 4477 | 69 | 1 | 158 | 51.8 | 6 | 2 | 1 | 1 | 3 | 1 | 1 | 1 | 1 | 2 | 1 | 1 | 1 | 2 |
| 4478 | 69 | 1 | 159 | 60.2 | 3 | 2 | 1 | 1 | 4 | 1 | 1 | 1 | 1 | 1 | 1 | 1 | 1 | 1 |
| 4479 | 69 | 1 | 159 | 58 | 8 | 2 | 1 | 2 | 3 | 1 | 1 | 1 | 1 | 1 | 1 | 1 | 2 | 1 |
| 4480 | 69 | 1 | 160 | 63 | 8 | 2 | 1 | 3 | 5 | 3 | 1 | 1 | 1 | 1 | 1 | 1 | 3 | 1 |
| 4481 | 69 | 1 | 161 | 67.5 | 2 | 2 | 1 | 1 | 3 | 1 | 1 | 1 | 1 | 1 | 1 | 1 | 1 | 2 |
| 4482 | 69 | 1 | 162 | 63.9 | 6 | 2 | 1 | 1 | 2 | 1 | 1 | 1 | 1 | 1 | 1 | 1 | 3 | 1 |
| 4483 | 69 | 1 | 162 | 56 | 8 | 2 | 1 | 4 | 4 | 1 | 1 | 1 | 1 | 1 | 2 | 2 | 2 | 1 |
| 4484 | 69 | 1 | 162.5 | 72.5 | 1 | 2 | 1 | 2 | 4 | 1 | 1 | 1 | 1 | 1 | 1 | 1 | 1 | 1 |
| 4485 | 69 | 1 | 162.5 | 44 | 8 | 2 | 1 | 1 | 4 | 1 | 1 | 1 | 1 | 1 | 1 | 1 | 1 | 1 |
| 4486 | 69 | 1 | 163 | 55.5 | 8 | 2 | 1 | 1 | 5 | 1 | 1 | 1 | 2 | 1 | 2 | 1 | 1 | 1 |
| 4487 | 69 | 1 | 165 | 70 | 8 | 2 | 1 | 1 | 3 | 1 | 1 | 1 | 1 | 1 | 1 | 1 | 3 | 1 |
| 4488 | 69 | 1 | 165 | 67 | 8 | 2 | 1 | 2 | 3 | 2 | 1 | 1 | 1 | 1 | 2 | 1 | 3 | 1 |
| 4489 | 69 | 1 | 165 | 62 | 8 | 2 | 1 | 1 | 5 | 3 | 1 | 1 | 1 | 1 | 1 | 1 | 1 | 1 |
| 4490 | 69 | 1 | 166 | 73.9 | 8 | 2 | 1 | 1 | 4 | 4 | 2 | 1 | 1 | 1 | 2 | 2 | 3 | 1 |
| 4491 | 69 | 1 | 167 | 70 | 1 | 2 | 1 | 1 | 5 | 2 | 1 | 1 | 1 | 1 | 1 | 1 | 1 | 1 |
| 4492 | 69 | 1 | 167 | 69 | 3 | 2 | 1 | 2 | 4 | 1 | 1 | 1 | 1 | 1 | 2 | 1 | 1 | 1 |
| 4493 | 69 | 1 | 168 | 63 | 6 | 2 | 1 | 1 | 4 | 1 | 1 | 1 | 1 | 1 | 1 | 1 | 1 | 1 |
| 4494 | 69 | 1 | 168 | 60 | 4 | 2 | 1 | 1 | 4 | 1 | 1 | 1 | 1 | 1 | 1 | 1 | 2 | 1 |
| 4495 | 69 | 1 | 168 | 58.5 | 8 | 2 | 1 | 4 | 3 | 2 | 1 | 1 | 1 | 1 | 1 | 1 | 3 | 1 |
| 4496 | 69 | 1 | 169 | 73.5 | 8 | 2 | 1 | 1 | 3 | 1 | 1 | 1 | 1 | 1 | 2 | 1 | 1 | 1 |
| 4497 | 69 | 1 | 169 | 64.5 | 8 | 2 | 1 | 1 | 3 | 1 | 1 | 1 | 1 | 1 | 1 | 1 | 3 | 1 |
| 4498 | 69 | 1 | 169.8 | 68 | 8 | 2 | 1 | 1 | 3 | 1 | 1 | 1 | 1 | 1 | 1 | 1 | 1 | 1 |
| 4499 | 69 | 1 | 170 | 66 | 3 | 2 | 1 | 1 | 4 | 1 | 1 | 1 | 1 | 1 | 1 | 1 | 2 | 1 |
| 4500 | 69 | 1 | 170 | 63 | 8 | 2 | 1 | 1 | 3 | 1 | 1 | 1 | 1 | 1 | 1 | 1 | 3 | 1 |
| 4501 | 69 | 1 | 170 | 60 | 8 | 2 | 2 | 1 | 5 | 1 | 1 | 1 | 1 | 1 | 2 | 1 | 1 | 1 |
| 4502 | 69 | 1 | 170.8 | 78 | 3 | 2 | 1 | 1 | 5 | 1 | 1 | 1 | 1 | 1 | 1 | 1 | 3 | 1 |
| 4503 | 69 | 1 | 172 | 77 | 8 | 2 | 1 | 1 | 4 | 1 | 1 | 1 | 1 | 1 | 1 | 1 | 1 | 1 |
| 4504 | 69 | 1 | 175 | 63 | 8 | 2 | 1 | 1 | 3 | 1 | 1 | 1 | 1 | 1 | 1 | 1 | 1 | 1 |
| 4505 | 69 | 1 | 178 | 77.5 | 8 | 1 | 1 | 4 | 3 | 1 | 1 | 1 | 1 | 1 | 2 | 1 | 3 | 2 |
| 4506 | 69 | 1 | 178.5 | 61.6 | 8 | 2 | 1 | 1 | 3 | 1 | 1 | 1 | 1 | 1 | 1 | 1 | 3 | 1 |
| 4507 | 70 | 2 | 141 | 59.9 | 2 | 2 | 1 | 1 | 4 | 1 | 1 | 1 | 1 | 1 | 1 | 1 | 1 | 1 |
| 4508 | 70 | 2 | 144 | 50 | 8 | 2 | 1 | 1 | 4 | 2 | 1 | 1 | 1 | 1 | 1 | 1 | 1 | 1 |
| 4509 | 70 | 2 | 145 | 38 | 3 | 3 | 1 | 1 | 2 | 2 | 1 | 1 | 1 | 1 | 1 | 1 | 3 | 1 |
| 4510 | 70 | 2 | 146 | 40.5 | 8 | 2 | 1 | 1 | 6 | 1 | 2 | 1 | 2 | 1 | 1 | 1 | 1 | 1 |
| 4511 | 70 | 2 | 148 | 65 | 8 | 2 | 1 | 1 | 2 | 1 | 1 | 1 | 1 | 1 | 1 | 1 | 1 | 1 |
| 4512 | 70 | 2 | 148 | 62 | 8 | 2 | 1 | 1 | 1 | 1 | 1 | 1 | 1 | 1 | 1 | 1 | 1 | 1 |
| 4513 | 70 | 2 | 148 | 53.2 | 8 | 3 | 1 | 1 | 3 | 1 | 1 | 1 | 1 | 1 | 2 | 1 | 1 | 1 |
| 4514 | 70 | 2 | 148.5 | 53.4 | 4 | 2 | 1 | 1 | 3 | 1 | 1 | 1 | 1 | 1 | 1 | 1 | 1 | 1 |
| 4515 | 70 | 2 | 149 | 70 | 8 | 3 | 1 | 1 | 1 | 4 | 1 | 1 | 1 | 1 | 1 | 1 | 1 | 1 |
| 4516 | 70 | 2 | 149 | 65 | 1 | 2 | 1 | 1 | 4 | 1 | 1 | 1 | 1 | 1 | 1 | 1 | 1 | 1 |
| 4517 | 70 | 2 | 149 | 44 | 8 | 2 | 1 | 1 | 2 | 4 | 1 | 1 | 1 | 1 | 1 | 1 | 1 | 1 |
| 4518 | 70 | 2 | 150 | 61 | 8 | 2 | 1 | 1 | 6 | 4 | 1 | 1 | 1 | 1 | 1 | 1 | 1 | 1 |
| 4519 | 70 | 2 | 150 | 45 | 1 | 2 | 1 | 1 | 3 | 2 | 1 | 1 | 1 | 1 | 1 | 1 | 1 | 1 |
| 4520 | 70 | 2 | 152 | 56 | 8 | 2 | 1 | 1 | 3 | 1 | 1 | 1 | 1 | 1 | 1 | 1 | 1 | 1 |
| 4521 | 70 | 2 | 152 | 53 | 8 | 2 | 1 | 1 | 4 | 4 | 1 | 1 | 1 | 1 | 1 | 1 | 1 | 1 |
| 4522 | 70 | 2 | 152 | 47.5 | 8 | 2 | 1 | 1 | 2 | 4 | 1 | 1 | 1 | 1 | 2 | 1 | 1 | 1 |
| 4523 | 70 | 2 | 153 | 60 | 8 | 2 | 1 | 1 | 3 | 1 | 1 | 1 | 1 | 1 | 1 | 1 | 1 | 1 |
| 4524 | 70 | 2 | 153 | 55 | 8 | 2 | 1 | 1 | 1 | 4 | 1 | 1 | 1 | 1 | 1 | 1 | 1 | 1 |
| 4525 | 70 | 2 | 153 | 45 | 8 | 2 | 1 | 1 | 2 | 4 | 1 | 1 | 1 | 1 | 1 | 1 | 1 | 1 |
| 4526 | 70 | 2 | 154 | 58.5 | 8 | 2 | 1 | 1 | 2 | 4 | 1 | 1 | 1 | 1 | 2 | 1 | 1 | 2 |
| 4527 | 70 | 2 | 154 | 57 | 8 | 3 | 1 | 1 | 3 | 1 | 1 | 1 | 1 | 1 | 2 | 1 | 1 | 1 |
| 4528 | 70 | 2 | 155 | 48.5 | 8 | 2 | 1 | 1 | 2 | 2 | 1 | 1 | 1 | 1 | 1 | 1 | 1 | 1 |
| 4529 | 70 | 2 | 155 | 47 | 8 | 2 | 1 | 1 | 2 | 1 | 1 | 1 | 2 | 1 | 1 | 1 | 1 | 1 |
| 4530 | 70 | 2 | 156 | 70 | 4 | 2 | 1 | 1 | 2 | 1 | 1 | 1 | 1 | 1 | 1 | 1 | 1 | 1 |
| 4531 | 70 | 2 | 156 | 59 | 5 | 2 | 1 | 1 | 4 | 1 | 1 | 1 | 1 | 1 | 1 | 1 | 1 | 1 |
| 4532 | 70 | 2 | 156 | 50 | 3 | 2 | 1 | 1 | 3 | 2 | 1 | 1 | 1 | 1 | 1 | 1 | 1 | 1 |
| 4533 | 70 | 2 | 156.2 | 55.8 | 2 | 2 | 1 | 1 | 4 | 2 | 1 | 1 | 1 | 1 | 1 | 1 | 1 | 1 |
| 4534 | 70 | 2 | 158 | 70 | 8 | 2 | 1 | 1 | 2 | 4 | 1 | 1 | 1 | 1 | 1 | 1 | 1 | 1 |
| 4535 | 70 | 2 | 158 | 60.9 | 2 | 2 | 1 | 1 | 4 | 1 | 1 | 2 | 2 | 1 | 2 | 1 | 1 | 1 |
| 4536 | 70 | 2 | 158 | 41.5 | 3 | 2 | 1 | 1 | 2 | 1 | 1 | 1 | 1 | 1 | 2 | 1 | 1 | 1 |
| 4537 | 70 | 2 | 159 | 67.5 | 8 | 2 | 1 | 1 | 6 | 3 | 1 | 1 | 1 | 1 | 1 | 1 | 1 | 1 |
| 4538 | 70 | 2 | 161 | 68.5 | 4 | 3 | 1 | 1 | 3 | 2 | 1 | 1 | 1 | 1 | 1 | 1 | 1 | 2 |
| 4539 | 70 | 2 | 165 | 55 | 8 | 2 | 1 | 1 | 2 | 4 | 1 | 1 | 1 | 1 | 1 | 1 | 1 | 1 |
| 4540 | 70 | 1 | 149.5 | 43.2 | 8 | 2 | 1 | 1 | 4 | 1 | 1 | 1 | 1 | 1 | 1 | 1 | 1 | 1 |
| 4541 | 70 | 1 | 155 | 70 | 8 | 2 | 1 | 1 | 2 | 2 | 1 | 1 | 1 | 1 | 1 | 1 | 1 | 1 |
| 4542 | 70 | 1 | 155 | 67 | 8 | 3 | 1 | 1 | 3 | 4 | 1 | 1 | 1 | 1 | 1 | 1 | 3 | 1 |
| 4543 | 70 | 1 | 156 | 68 | 8 | 2 | 1 | 1 | 2 | 3 | 1 | 1 | 1 | 1 | 1 | 1 | 1 | 1 |
| 4544 | 70 | 1 | 156 | 55 | 8 | 2 | 1 | 2 | 3 | 1 | 1 | 1 | 1 | 1 | 2 | 2 | 1 | 1 |
| 4545 | 70 | 1 | 157.5 | 53.5 | 2 | 2 | 1 | 1 | 2 | 2 | 1 | 1 | 1 | 1 | 2 | 1 | 1 | 1 |
| 4546 | 70 | 1 | 158 | 71 | 8 | 2 | 1 | 1 | 4 | 4 | 1 | 1 | 2 | 1 | 1 | 1 | 1 | 1 |
| 4547 | 70 | 1 | 160 | 73.4 | 2 | 2 | 1 | 3 | 4 | 1 | 1 | 1 | 1 | 1 | 1 | 1 | 3 | 1 |
| 4548 | 70 | 1 | 160.5 | 63.5 | 2 | 2 | 1 | 4 | 3 | 1 | 1 | 1 | 1 | 1 | 2 | 1 | 3 | 1 |
| 4549 | 70 | 1 | 162 | 65 | 8 | 2 | 1 | 1 | 3 | 4 | 1 | 1 | 1 | 1 | 1 | 1 | 1 | 1 |
| 4550 | 70 | 1 | 162 | 61 | 8 | 2 | 1 | 1 | 4 | 2 | 1 | 1 | 1 | 1 | 1 | 1 | 1 | 1 |
| 4551 | 70 | 1 | 162 | 55.6 | 2 | 2 | 1 | 1 | 5 | 1 | 2 | 2 | 1 | 1 | 1 | 1 | 1 | 1 |
| 4552 | 70 | 1 | 162.8 | 63.9 | 3 | 2 | 1 | 1 | 5 | 1 | 1 | 1 | 1 | 1 | 1 | 1 | 3 | 1 |
| 4553 | 70 | 1 | 163 | 66 | 8 | 2 | 1 | 1 | 4 | 1 | 1 | 1 | 1 | 1 | 1 | 1 | 1 | 1 |
| 4554 | 70 | 1 | 164 | 76.2 | 8 | 2 | 1 | 1 | 4 | 1 | 1 | 1 | 2 | 1 | 1 | 1 | 1 | 1 |
| 4555 | 70 | 1 | 164 | 64 | 8 | 2 | 1 | 4 | 3 | 1 | 1 | 1 | 1 | 1 | 2 | 2 | 2 | 1 |
| 4556 | 70 | 1 | 165 | 75 | 8 | 2 | 1 | 2 | 4 | 2 | 1 | 1 | 1 | 1 | 1 | 1 | 1 | 1 |
| 4557 | 70 | 1 | 165 | 70 | 8 | 2 | 1 | 4 | 2 | 4 | 1 | 1 | 1 | 1 | 1 | 1 | 3 | 1 |
| 4558 | 70 | 1 | 165 | 68 | 8 | 2 | 1 | 2 | 3 | 1 | 1 | 1 | 1 | 1 | 1 | 1 | 3 | 1 |
| 4559 | 70 | 1 | 165 | 65 | 1 | 2 | 1 | 4 | 5 | 1 | 1 | 1 | 1 | 1 | 1 | 1 | 1 | 1 |
| 4560 | 70 | 1 | 165 | 62.5 | 8 | 2 | 1 | 1 | 5 | 4 | 2 | 1 | 1 | 2 | 2 | 1 | 1 | 1 |
| 4561 | 70 | 1 | 167 | 70 | 8 | 2 | 1 | 1 | 3 | 3 | 1 | 1 | 1 | 1 | 1 | 1 | 1 | 1 |
| 4562 | 70 | 1 | 167 | 70 | 8 | 2 | 1 | 1 | 3 | 1 | 1 | 1 | 1 | 1 | 1 | 1 | 1 | 1 |
| 4563 | 70 | 1 | 168 | 80 | 8 | 2 | 1 | 2 | 2 | 2 | 1 | 1 | 1 | 1 | 1 | 1 | 2 | 1 |
| 4564 | 70 | 1 | 168 | 58.5 | 8 | 2 | 1 | 1 | 4 | 4 | 1 | 1 | 1 | 1 | 1 | 1 | 2 | 1 |
| 4565 | 70 | 1 | 170 | 67 | 6 | 2 | 1 | 1 | 4 | 1 | 1 | 1 | 1 | 1 | 1 | 1 | 1 | 1 |
| 4566 | 70 | 1 | 171 | 59 | 8 | 2 | 1 | 1 | 2 | 1 | 1 | 1 | 1 | 1 | 1 | 1 | 1 | 1 |
[truncated: 67,768 more chars]
